# “Expecting the unexpected?” Uncovering role expectation differences in a Dutch hospital

**DOI:** 10.3389/fpsyg.2022.951359

**Published:** 2022-09-30

**Authors:** Milan Wolffgramm, Joost Bücker, Beatrice Van der Heijden

**Affiliations:** ^1^Research Group Employability Transition, Saxion University of Applied Sciences, Enschede, Netherlands; ^2^Institute for Management Research, Radboud University, Nijmegen, Netherlands; ^3^Faculty of Management, Open University of the Netherlands, Heerlen, Netherlands; ^4^Department of Marketing, Innovation and Organisation, Ghent University, Ghent, Belgium; ^5^School of Business, Hubei University, Wuhan, China; ^6^Kingston Business School, Kingston University, London, United Kingdom

**Keywords:** devolution, human resource management, front-line managers, healthcare management, qualitative research, role theory, role expectations, role stress

## Abstract

The aim of this study was to empirically investigate differences in role expectations, among the stakeholders involved, about the devolved personnel management role of front-line managers (FLMs). In particular, we researched the role expectation differences between FLMs, their middle managers, and Human Resource (HR) practitioners. In total, nineteen semi-structured interviews have been conducted involving eleven FLMs, eight middle managers, and two HR practitioners working at the same Dutch hospital. Most discovered role expectation differences were related to how FLMs should execute their HR tasks (i.e., process ambiguity). FLMs were often uncertain if their role enactment met those of their middle managers and/or HR practitioners, herewith indicating role stress. Our findings underline the importance of paying attention to role expectations’ differences in aligning components of the HRM-performance relationship. Future research could include the role expectations of other important stakeholders, such as: subordinates and top management. The outcomes of this empirical work are translated into four interventions to diminish FLMs’ role stress.

## Introduction

Front-line managers (FLMs) play a crucial role in healthcare organizations’ Human Resource Management (HRM; McConville and Holden, 1999; Kabene et al., 2006; Op de Beeck et al., 2016). Hales (2005) defined the function of ‘first-line manager’ as “the position representing the first level of management to whom non-managerial employees report” (p. 473). In terms of hierarchy, FLMs, in turn, are managed by middle managers. FLMs’ importance in HRM can be ascribed to the increasing number of Human Resource (HR) tasks (or responsibilities) they have been entrusted with (Lowe, 1992; Keen and Vickerstaff, 1997; Papalexandris and Panayotopoulou, 2005).

The increase in FLMs’ HR-related responsibilities goes back to the 1980’s wherein Fombrun et al. (1984) stated that “any attempt to redesign the role of the HR function requires the line’s participation since most of the activities of selection, appraisal, reward, and development are prerogatives of the line organization” (p. 236). Building upon this notion, in the 1990’s, Hoogendoorn and Brewster (1992) introduced the term ‘devolution’ which they defined as “the allocation of tasks formerly undertaken by the personnel specialists to front-line managers” (p. 4). Building on this notion of devolution, Brewster and Larsen (2000) focused upon investigating the organizational rationale behind devolution and discovered five reasons for it: (1) to reduce costs; (2) to meet the need for a more comprehensive approach towards HRM; (3) to speed up decision-making processes; (4) to reflect changes in philosophy and organizational structure; and (5) to launch an alternative for outsourcing the HR function. Besides, devolving HR tasks to line managers leaves more time for HR practitioners to implement sound HR practices that foster sustainable competitive advantage through people management (Boxall, 1996; Sisson and Storey, 2000; Finegold and Frenkel, 2006). However, whether these benefits will be achieved strongly depends on the alignment between components of the Process Model of Strategic HRM (Wright and Nishii, 2013).

The Process Model of Strategic HRM (Wright and Nishii, 2013) showcases the causal chain between HR initiatives and organizational performance and departs from the formulated HR strategy and designed HR practices, that is the intended HR practices. These intended HR practices have to be implemented in practice. The Process Model of Strategic HRM was already empirically confirmed by Bos-Nehles et al. (2013) who stated: “even if the intended HR practices are well designed, they will be ineffective if they are not properly implemented” (p. 862).

FLMs are considered to be the key players who should implement these intended HR practices (Hales, 2005; Nehles et al., 2006; Purcell and Hutchinson, 2007; Townsend et al., 2015), due to their close interaction with operational employees. This close interaction allows FLMs to steer the perceptions of their subordinates about the implemented HR practices, which will affect their behavior (employee reaction) and, subsequently, organizational outcomes (organizational performance) accordingly (Bowen and Ostroff, 2004; Nehles et al., 2006; Andersen et al., 2007; Purcell and Hutchinson, 2007; Hutchinson and Purcell, 2010; Boon et al., 2011). The FLMs’ key position in the Process Model of Strategic HRM (Wright and Nishii, 2013), and thus in the HR landscape, resulted in increased attention for the scholarly topic of HR devolution in the last two decades (Qadeer, 2011).

This increased attention has resulted into various studies on the different aspects of HR devolution (Cascón-Pereira and Valverde, 2014), for instance, research on the areas of HRM that have been devolved (e.g., recruitment and selection), and research on how these areas are distributed among organizational members (e.g., Conway and Monks, 2010). Furthermore, scientists studied the consequences that come with HR devolution (e.g., Sheehan, 2012), its impact (e.g., Morley et al., 2006), and its overall usefulness (e.g., Gilbert et al., 2011a). In addition, within the HR devolution literature, a few authors investigated the problems FLMs experience regarding their ascribed HR role (Hutchinson and Purcell, 2010; Gilbert et al., 2011a; Dewettinck and Vroonen, 2017; Evans, 2017).

A role refers to “the boundaries and sets of expectations applied to role incumbents of a particular position, which are determined by the role incumbent and the role senders within and beyond the organization’s boundaries” (Banton, 1965, p. 6). In the context of this study, FLMs are the role incumbents. Examples of the FLMs’ role senders are: senior managers, middle managers, HR practitioners, subordinates, colleagues, and customers (Evans, 2017). That roles are important in organizational contexts is known for a longer time. To illustrate, roles are considered to be the “building blocks of social systems” (Katz and Kahn, 1978, p. 219) and help to conceptualize human behavior in organizations (Dougherty and Pritchard, 1985).

It is important to note that, although highly related, roles are different from duties and competencies. Hodgson (1995) defined a duty as “a course of action that is required of one by position” (p. 80). He further explains how one’s duties are related to social customs, for honoring moral obligations, or to perform assigned tasks. These driving forces could motivate someone to act without having a complete understanding why action should be taken. On the other hand, roles are different from duties in that they solely focus on the expected activities that are associated with a particular position or job (Katz and Kahn, 1978). We argue, therefore, that a role, in essence, resembles a more clear-cut concept as it focusses on objective activities (i.e., who, what, where, when, and how) and, initially, leaves out the complexity coming with social and moral norms.

Analogously, roles and competencies also differ from one another. More specifically, competencies are the capacities one should have to execute a particular role. In other words, knowledge, skill, ability, and attitude requirements in existing HR competency models (e.g., Ulrich et al., 1995, 2007, 2013, 2015; Brockbank and Ulrich, 2003; The RBL Group, 2015) are only relevant when these are in line with the ascribed HR roles of FLMs. Therefore, it is of crucial importance to have a thorough understanding about the role expectations both FLMs and role senders (i.e., their surrounding stakeholders) attach to the FLMs’ HR role. We argue that adopting a social work environment perspective [Beijer et al., 2019 (*cf.*, a multiple-stakeholder or systemic perspective; Colakoglu et al., 2006)] is highly relevant in order to gain more insight into possible differences in role expectations among the parties (stakeholders) involved.

The so-called role expectations embody the “norms, beliefs, and preferences concerning the performance of any individual in a social position, relative to individuals occupying other positions” (Thies, 2013, p. 33). Role expectations are relevant as these do not only determine the competencies one should master to meet them, yet, and even more important in the light of our empirical work, role expectations can differ between FLMs and role senders. For instance, Morley et al. (2006) and Hutchinson and Purcell (2010) found that middle managers held different expectations about what the FLMs’ HR role exactly comprises.

We contend that it is of utmost importance to increase our understanding of possible differences in role expectations, as the absence of information about the FLMs’ HR role, or lack of role clarity, results in increased role ambiguity and, subsequently, role stress for FLMs. Role ambiguity is a serious threat for FLMs, and for the effectiveness of their HR implementation. Not only is role ambiguity negatively related with job satisfaction (Jong, 2016) and job performance (Bauer and Simmons, 2000; LePine et al., 2016), it also undermines the FLMs’ ability to implement HRM tasks as intended. As a result, it is likely that optimal levels of organizational performance will not be achieved in case of role ambiguity, as the latter disrupts the causal chain between intended, implemented, and perceived HRM (Khilji and Wang, 2006; Do et al., 2018). That is to say, to mitigate role ambiguity, it is important to align the role expectations between FLMs and role senders.

Mat and Barrett (2015) conducted empirical research on the alignment between role expectations of middle managers and HR practitioners regarding FLMs’ HR role in two Malaysian airports. In their study, middle managers and HR practitioners were asked to determine which HR tasks FLMs should execute in their opinion. While their work provided fruitful insights in the role expectations of both parties, only one side of the story was witnessed as researchers did not pay attention to the expectations of the role incumbents, that is the FLMs themselves. Instead, the work of Mat and Barrett (2015) relied on the assumption that “the role holder enacted their role based on what was expected and required by others in a similar role set” (p. 123). With this assumption, that they based on Katz and Kahn (1978), the authors assumed that middle managers’ and HR practitioners’ role expectations would be equal to the role expectations of FLMs.

Notwithstanding the importance of their work for the domain of HR devolution, there is still a serious lack of scholarly research in this field. Harris et al. (2002) explored the concerns that HR devolution entail and concluded that it is far more complex than commonly assumed. In order to optimize the process of HR devolution, and to more effectively prepare FLMs for their new HR responsibilities, these authors advocated to stress the importance of joint ownership and to pay more attention to the individual development needs of the different parties involved, in order to enhance the quality of HR processes. Our research is aimed to move the work in this field forward and we posit that joint ownership and working towards the common goal of high-quality HR processes is only possible if one is better able to understand differences in role expectations. Kurdi-Nakra et al. (2022) already called for more scholarly work to better understand the mechanisms behind the social dynamics among various HR actors that shape the HR implementation process. Obvious differences in expectations about the FLMs’ role shape the social dynamics between these actors, their HR enactment and determine whether joint ownership is practiced.

Kou and associates (2022), in their exemplary theorizing on FLMs’ HR role identity to articulate FLMs’ sense-making process toward their devolved HR duties, set the stage for more empirical work into (differences) in role expectations. However, with the exceptions of the work by Mat and Barrett (2015; see above) and the work by Op de Beeck et al. (2016), who studied differences in perceptual discrepancy between line managers and HR professionals on the degree of HR devolution, to the best of our knowledge, no previous scholarly work with a focus on differences in role expectations has been conducted, let alone incorporating several categories of stakeholders. In addition, no previous empirical work has compared the role expectations of FLMs themselves, on the one hand, and other stakeholders, on the other hand.

Therefore, in this contribution we will further disentangle the concerns that HR devolution entails by an in-depth qualitative approach that focuses on differences in role expectations among three distinguished categories of stakeholders (i.e., FLMs, middle managers, and HR practitioners). As such, this study adopts a multiple-stakeholder perspective (Colakoglu et al., 2006) which should resonate in aggregated views allowing the identification of complementary and competing expectations regarding the FLMs’ devolved HR role. In doing so, possible tensions will emerge, which can form the basis for a much better alignment across the stakeholders and, as a result, a better HR implementation process.

For our theoretical framing, our study builds on Evans (2017) who claimed that role theory (Biddle, 1979, 1986) may be useful to shed more light on the importance of role expectations within the HRM-performance link. More specifically, two distinctive, yet complementary, frameworks for uncovering role expectations will be combined: devolution dimensions (Cascón-Pereira and Valverde, 2014) to roughly identify what FLMs’ HR role entails and role ambiguity dimensions (Bedeian and Armenakis, 1981) to study how the HR role should be enacted. We deliberately combine these frameworks as they bare the potential to help us collect more detailed role expectations and allow us to allocate role expectation differences better. We explain both frameworks in our literature review.

Finally, based on the identified role expectation differences, practical solutions will be formulated to overcome possible role stress resulting from these. Bridging role expectation differences is highly beneficial as this is the first step in decreasing FLMs’ role conflict, role ambiguity, and role overload (Bauer and Simmons, 2000), which, we argue, will positively impact the implementation of HR policies and, subsequently, organizational performance (Richard and Johnson, 2001). Although the FLMs’ senior managers, subordinates, colleagues, and customers are also considered to be important holders of role expectations regarding the HR function of FLMs (Evans, 2017), this research will focus on the role expectations of FLMs, middle managers, and HR practitioners. This is for two reasons. First, comparing the role expectations of middle managers and HR practitioners with those of the FLMs themselves responds to closing the previously mentioned knowledge gap, and making sure that we contribute to the domain of knowledge in an incremental way. Second, due to the position of the middle managers and HR practitioners in the HRM-performance chain, these actors are most likely to have certain expectations regarding the FLMs’ HR role enactment. Therefore, our central research question is as follows: *How do the individual role expectations of FLMs, middle managers, and HR practitioners regarding the FLMs’ devolved HR function differ from one another?*

## Literature review

First, we will elaborate on the devolution dimensions. Second, we will explain the role expectations and role ambiguity dimensions. We close this section by portraying an image visualizing the central notion behind this research.

HR devolution comprises a multi-dimensional concept (Cascón-Pereira and Valverde, 2014), containing four dimensions (p. 155): (1) the implementation of tasks which concerns the HR activities line managers are involved in; (2) the decision-making power of line managers which concerns the freedom to take decisions about the execution of the devolved HR tasks, without the interference of their middle manager; (3) the financial power which refers to the financial resources a line manager is allowed to allocate autonomously when executing the devolved HR tasks; and (4) the knowledge that entails all HR and non-HR information line managers need to possess for properly enacting their HR role. In other words, by filling in the devolution dimensions, one can define an FLM’s HR role.

Despite the existing body of HR devolution literature, only a few studies have distinguished the hierarchical layers of line management, such as senior managers, middle managers, and FLMs (McConville, 2006). Especially the role of FLMs was considered to be overlooked in previous HR literature (Sanders and Frenkel, 2011; Townsend et al., 2012, 2015; Brewster et al., 2013). In their more recent literature reviews, Kurdi-Nakra et al. (2022) and Townsend et al. (2022) elaborate on the various research attempts that were made over time to further clarify the role of the FLM in the HR landscape. However, we sensed that in these inquiries, although they often adapt a multi-actor perspective, overstep a systematic, in-depth analysis of the content and differences in role expectation (e.g., Blayney et al., 2020; Tyskbo, 2020; Kou et al., 2022).

Role expectations closely align with role theory (Biddle, 1979, 1986) in the sense that individuals hold expectations for their own behaviors, and of those of others in particular positions. Specifically (Thies, 2013) stated that individuals in certain positions are expected to behave in a specific way and to perform at an expected time and place. Moreover, role expectations define what behavior is being tolerated and what behavior is not. In this sense, the set of role expectations that are related to one’s specific position guides and directs an individual’s behavior (Solomon et al., 1985). Role expectations are related to role ambiguity as they can be “vague, indefinite, or ambiguous” (Biddle, 1986, p. 83), leaving the individual in confusion about what is actually being expected from him or her (Hill, 2005). Ambiguity constraints choice as desirable and undesirable alternatives can hardly be distinguished (McLain et al., 2015).

To specifically determine the boundaries and enactment of the role expectations that FLMs, middle managers, and HR practitioners ascribe to the FLMs’ HR function, four dimensions of role ambiguity, that are distinguished in earlier scholarly work, will be used for our empirical work (see Bedeian and Armenakis, 1981; McConville, 2006). Each of these dimensions holds specific expectations in regards to what, when, and how a person executes particular tasks. Thus, where the devolution dimensions define the contours of the FLM’s HR role, the role ambiguity dimensions could add additional details on how the defined HR role should be executed. When carefully comparing in role expectations that the three distinguished actors adhere to the FLMs’ HR function, possible misconceptions about the FLMs’ HR roles will become visible (Hill, 2005).

Following (Bedeian and Armenakis, 1981), the four role ambiguity dimensions that are incorporated in our study, are: first, goal/expectation/responsibility ambiguity, which refers to the clarity about what the individual is expected to do and where the boundary of their tasks is located; second, process ambiguity, which deals with the clarity on how to execute the ascribed tasks; third, priority ambiguity, which comprises clarity about when, and in which order, tasks should be executed; and fourth, behavior ambiguity, which refers to the clarity about the behavior an individual is expected to enact.

In a similar vein, various studies that described the different HR roles of FLMs (McConville, 2006; Hutchinson and Purcell, 2010; Gilbert et al., 2011a; Evans, 2017; Tamayo-Verleene, 2021) concluded that FLMs run a high risk of experiencing role stress (Sawyer, 1992), in terms of not knowing how to combine their HR roles with other ascribed roles (i.e., experiencing role conflict), not being able to cope with job demands accompanying the HR roles (i.e., experiencing role overload), or not knowing what the HR roles entail and how to enact the roles according to expectations (i.e., experiencing role ambiguity). As we focus on the role expectation differences in regards to the FLMs’ HR role, role ambiguity is the focal point of attention in our study. Lyons (1971) reported that individuals will experience role clarity when having access to unvaried, role-relevant information, and when they experience a feeling of having enough role-relevant information. Obviously, individuals will face role ambiguity when they do not experience role clarity (Smith and Brannick, 1990; Chang et al., 2021), and the resulting role stress may negatively influence their job performance (Miner, 1971; Tubre and Collins, 2000; Wu et al., 2019), in our case, the effective implementation of HR policies.

**Figure 1:**
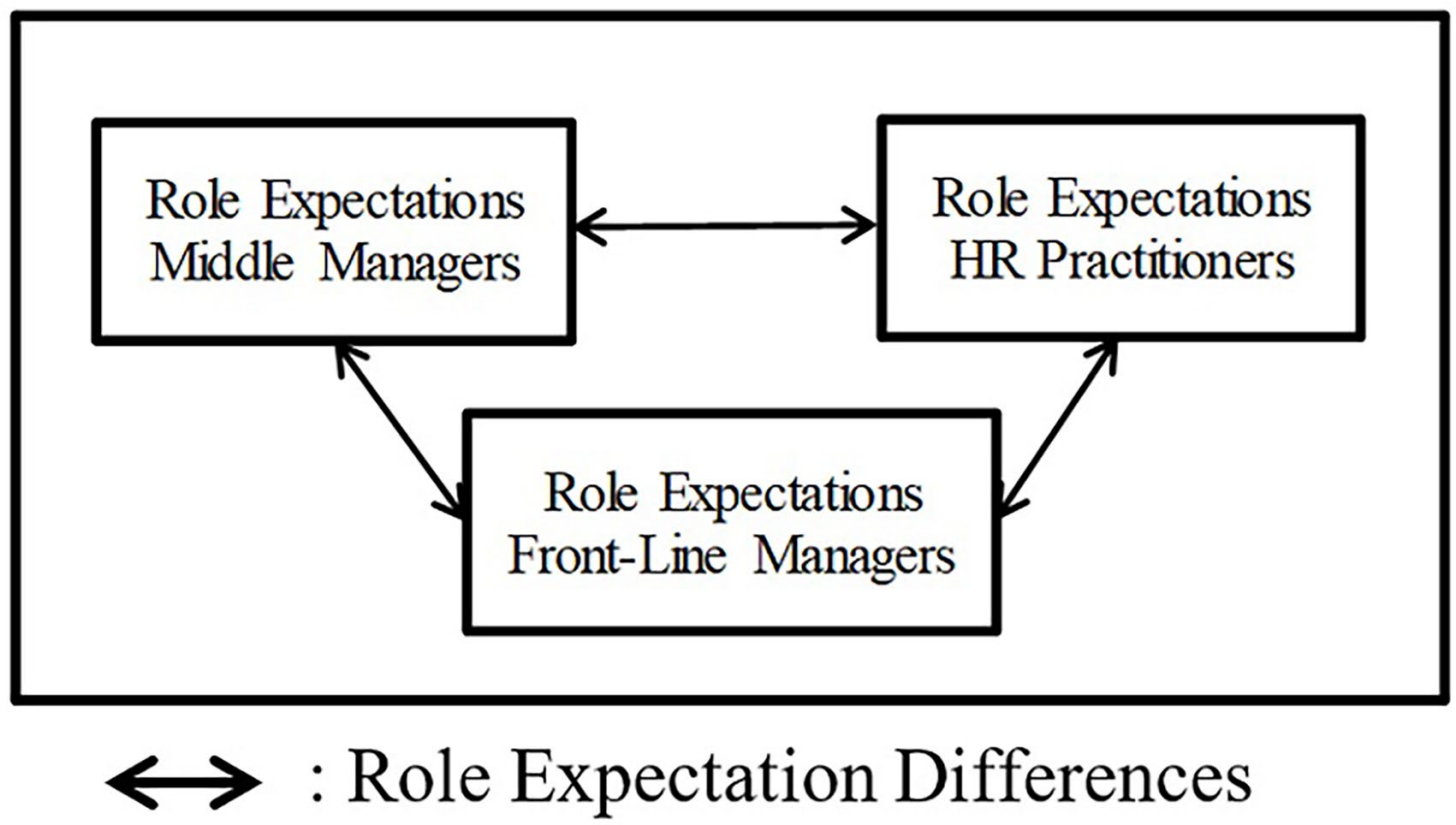
Role expectation differences amongst stakeholders’ role expectations.

Figure 1 summarizes this study’s theoretical background. FLMs, middle managers, and HR practitioners are the individuals under study, who are holding certain role expectations concerning the FLMs’ devolved HR role. These expectations bear elements of the devolution dimensions and role ambiguity dimensions. Inspired by McDermott et al. (2015), a double-headed arrow is drawn between the distinguished stakeholder categories, as this study’s aim is to explore the differences in the role expectations of the distinguished actors.

## Materials and methods

An exploratory qualitative research method was used as this study focuses on theory-building by identifying in-depth role expectations of multiple parties about the HR role of FLMs and finding differences amongst these expectations. Since role expectations are complex as they are highly diverse in terms of content and execution, as explained in our literature review, it makes them hard to capture effectively through survey or experiment strategies (Maxwell, 2009). Therefore, qualitative research fit our research question best as it mostly allows us to generate an answer to the question how individual role expectations of FLMs, middle managers, and HR practitioners regarding the FLMs’ devolved HR function differ from one another.

After tremendous efforts to find willingness to participate in our study, one Dutch non-academic hospital was ready to participate in this study. We contend that this Dutch hospital is particularly suitable in the light of our study, as the Netherlands is one of the leading countries in devolving HR tasks to front-line managers (Andolsek and Stebe, 2005). Moreover, the healthcare sector was chosen as devolution is a proven phenomenon in hospitals (see Harris et al., 2002; Dorenbosch et al., 2006; McConville, 2006; Stanton et al., 2010; Cascón-Pereira and Valverde, 2014) while at the same time there is a lack of research concerning the role expectations regarding the HR role of FLMs (Genrich et al., 2020). Finally, in the Dutch hospital sector, FLMs are often promoted team members that spend their time on both HR tasks and operational tasks (e.g., providing healthcare). Being positioned between different managerial layers (middle management and operations) and work demands (HR tasks versus operational tasks) comes with the risk of role ambiguity and, thus, role expectation differences.

The participating hospital has between 300 and 400 occupied beds on a daily basis, and employs around 1,750 employees. Furthermore, the hospital holds around 25 healthcare units (e.g., Intensive Care) and about five support units (e.g., Facility Services). The FLMs in the hospital are known as ‘Team Leaders’ and manage operational staff (subordinates), such as nurses (in case of healthcare units) and cleaners (in case of support units). FLMs are managed by the so-called Business Leaders, who are part of the hospital’s middle management. Each healthcare and support unit is being supported by one of the five HR advisors – each HR advisor is linked to its own units and knowledgeable about the actualization of HR practices in those particular units. The HR advisors constitute this study’s HR practitioner population.

For this qualitative study, nineteen semi-structured, open-ended interviews were conducted across six healthcare units and two support units. In total, eleven FLMs and eight respective middle managers were interviewed. We also interviewed two HR practitioners that were supporting half of the units under study. Moreover, seventeen interviews involved one interviewee, whereas two interviews involved multiple interviewees due to scheduling issues (one interview with two FLMs, another one with an FLM and a middle manager). All interviewees provided their informed consent verbally. The interviews themselves lasted 46 min on average (with a standard deviation of approximately 17 min), and were conducted in Dutch. More information on the sample characteristics and the duration of the interviews is presented in Table 1.

**Table 1:** Sample characteristics.

Unit ID	Unit type	Middle manager ID	Sex middle manager	Duration middle manager interviews (in minutes) mean = 38	FLM ID	Sex FLM	Years of experience in FLM position mean = 2.9	Duration FLM interviews (in minutes) mean = 50	HR practitioner 1 (sex: female; duration: 56 min)	HR practitioner 2 (sex: female; duration: 71 min)
1	Healthcare	1	Female	15	1	Female	5.7	40	No	No
					2	Female	1.8	-(1)	No	No
2	Healthcare	2	Female	28	3	Female	4	35	No	No
3	Healthcare	3	Female	24	4	Male	4	55	No	No
4	Support	4	Male	29	5	Male	0.8	54	Yes	No
					6	Female	3.4	76	Yes	No
5	Healthcare	5	Female	61	7	Female	1.8	44	No	Yes
					8	Female	4	51	No	Yes
6	Support	6	Male	46	9	Male	1.5	30	No	Yes
7	Healthcare	7	Female	62	10	Female	1	66	No	Yes
					11	Female	4	-(1)	No	Yes
8	Healthcare	8	Female	34	n.a.	n.a.	n.a.	n.a.	No	No

(1) - particiated in a duo interview.

The interviews were conducted based on a semi-structured interview protocol comprising open questions and additional explanations. We choose for semi-structured interviews due to the nature of our research question. To answer our research question, we needed highly detailed yet comparable data to not only define role expectations but also to uncover role expectation differences. While open interviews are ideal for collecting very detailed data as it allows the interviews to freely ask questions, it comes with the risk of getting off-topic and making data difficult to compare (Corbin and Morse, 2003). Fully structured interviews, however, come with the opposite advantage and disadvantage: it helps to stay close to the research topics of interest but leave no room for emerging, in-depth questions nor the opportunity to clarify answers (Bryman, 2016). In that sense, a semi-structured interviews embodied in our eyes the best trade-off between data richness on the one hand and data comparability on the other.

The questions constituting the semi-structured interview protocol (see Supplementary material) originated from previous, scholarly operationalization of the differentiated HR devolution dimensions (Cascón-Pereira and Valverde, 2014) and the role ambiguity dimensions (Bedeian and Armenakis, 1981), herewith securing internal validity (i.e., construct measurement). To further increase the study’s internal validity and to help interviewees to define the FLMs’ HR roles, 22 frequently-devolved HR tasks [i.e., recruitment, selection, induction, and maintaining staff records (Hutchinson and Purcell, 2010)] were shown to interviewees to prime them for further discussing the HR role expectations. Stressed by Glegg (2019), using visual tools generates opportunities to collect richer data.

Although the same interview protocol was used for each interview, the exact formulation of specific questions was adjusted to the respective category the interviewee belonged to (e.g., “What do you expect from the FLM’s HR role” versus “What do you think is being expected from your HR role”). All interviews took place in the hospital, were conducted in Dutch by the first author, were fully recorded, and were translated into full verbatim transcripts by the first author.

For the data analysis procedure, a deductive thematic approach (Fereday and Muir-Cochrare, 2006) was taken. We used the devolution dimensions (implementation of tasks, decision-making power, financial power, and knowledge) and role ambiguity dimensions (goal/expectancy/responsibility ambiguity, process ambiguity, priority ambiguity, and behavior ambiguity) as labels. The labels were operationalized based on the theory presented in the literature review above. The combination of labels and operationalisations comprised this research’s code book. Based on these operationalisations, the transcripts were filtered for relevant texts by the first author. Relevant texts were summarized in a code of one or a few words and connected to the label. The number of times a similar code was used was administered. To illustrate, the devolution dimension label ‘financial power’ comes with the code ‘flowers’. The code was retraced in eleven interviews and was expressed in quotes alike: “My FLMs should buy the flowers themselves.” The first author reported about the preliminary coding to the second author for the sake of safeguarding internal validity. In order to uncover role expectation differences, the outcomes of the coding processes were first compared within healthcare and support units and later across the actors (FLMs, middle managers, HR practitioners).

In terms of quality, our method checks the four criteria Guba (1981) introduced to assess the so-called trustworthiness of qualitative research endeavors: credibility, transferability, dependability, and confirmability. We based our argumentation on Shenton’s (2004) strategies for ensuring trustworthiness. Our work is credible as we used suitable and evidence-based operational measures to study the topics of interest. Transferability was secured by providing plenty of details about the context and the individuals under study. By carefully describing the build-up, execution, and analysis of our study, we aimed to enable future researchers to repeat our work and meet the dependability criterium. Finally, in terms of confirmability, we did our utter best to reduce researcher bias by using multiple coders, showcasing our coding approach, and staying close to the data (i.e., limiting interpretation).

## Results

Based on the collected empirical data, the differences between FLMs’, middle managers’, and HR practitioners’ role expectations regarding the FLMs’ HR role were made salient. We structured the results as following: this section starts off with the devolution dimensions (Hutchinson and Purcell, 2010; Cascón-Pereira and Valverde, 2014) and gives a broad overview regarding FLMs’ expected HR tasks, decision-making power, financial power, and knowledge. Next, we present the role ambiguity dimensions (Bedeian and Armenakis, 1981) and give a more detailed impression regarding the role expectations’ boundaries, how and when the FLMs are expected to enact their HR role, and what behavior is expected.

### Devolution dimension: Implementation of HR tasks

As Table 2 showcases, FLMs are involved in a wide range of HR tasks. However, small nuances were found. Most interviewees expected FLMs to occupy themselves with conducting performance appraisals and development reviews, managing absences, coordinating the work of teams by creating work planning schedules, and communicating top-down and bottom-up. However, different from what was reported by the middle managers and HR practitioners, FLMs also considered themselves to be involved in HR tasks related to the health and safety, and the improvement of the subordinates’ working lives.

**Table 2:** HR duties mentioned by middle managers, HR practitioners, and FLMs.

HR duty	Middle manager		HR		FLM	
Recruitment	Healthcare:	2	HR Practitioner 1:	1	Healthcare:	8
	Support:	1	HR Practitioner 2:	1	Support:	3
Selection	Healthcare:	4	HR Practitioner 1:	1	Healthcare:	8
	Support:	1	HR Practitioner 2:	1	Support:	3
Induction	Healthcare:	2	HR Practitioner 1:	1	Healthcare:	7
	Support:	4	HR Practitioner 2:	1	Support:	2
Maintaining staff records	Healthcare:	2	HR Practitioner 1:	1	Healthcare:	7
	Support:	1	HR Practitioner 2:	1	Support:	3
Deciding and planning training and development needs of staff	Healthcare:	4	HR Practitioner 1:	1	Healthcare:	6
	Support:	2	HR Practitioner 2:	0	Support:	3
Providing formal training	Healthcare:	1	HR Practitioner 1:	1	Healthcare:	1
	Support:	1	HR Practitioner 2:	0	Support:	0
Providing informal training including coaching and guidance	Healthcare:	4	HR Practitioner 1:	1	Healthcare:	8
	Support:	2	HR Practitioner 2:	1	Support:	3
Mentoring	Healthcare:	4	HR Practitioner 1:	1	Healthcare:	5
	Support:	0	HR Practitioner 2:	0	Support:	0
Performance appraisal/development reviews	Healthcare:	6	HR Practitioner 1:	1	Healthcare:	8
	Support:	2	HR Practitioner 2:	1	Support:	3
Agreeing performance development plans	Healthcare:	2	HR Practitioner 1:	1	Healthcare:	3
	Support:	2	HR Practitioner 2:	1	Support:	2
Discipline and grievance handling	Healthcare:	2	HR Practitioner 1:	1	Healthcare:	3
	Support:	2	HR Practitioner 2:	1	Support:	2
Absence management	Healthcare:	6	HR Practitioner 1:	1	Healthcare:	8
	Support:	2	HR Practitioner 2:	1	Support:	3
Giving recognition	Healthcare:	2	HR Practitioner 1:	1	Healthcare:	5
	Support:	0	HR Practitioner 2:	1	Support:	2
Pay banding decisions	Healthcare:	0	HR Practitioner 1:	0	Healthcare:	0
	Support:	0	HR Practitioner 2:	0	Support:	0
Upward communication	Healthcare:	6	HR Practitioner 1:	1	Healthcare:	8
	Support:	2	HR Practitioner 2:	1	Support:	3
Downward communication	Healthcare:	6	HR Practitioner 1:	1	Healthcare:	8
	Support:	2	HR Practitioner 2:	1	Support:	3
Listening and responding to staff suggestions	Healthcare:	3	HR Practitioner 1:	0	Healthcare:	5
	Support:	1	HR Practitioner 2:	1	Support:	2
Coordinating the work of teams/shifts	Healthcare:	6	HR Practitioner 1:	1	Healthcare:	8
	Support:	2	HR Practitioner 2:	1	Support:	3
Maintaining effective teamwork	Healthcare:	2	HR Practitioner 1:	0	Healthcare:	5
	Support:	1	HR Practitioner 2:	1	Support:	2
Counselling staff	Healthcare:	4	HR Practitioner 1:	1	Healthcare:	5
	Support:	2	HR Practitioner 2:	1	Support:	1
Health and safety	Healthcare:	3	HR Practitioner 1:	1	Healthcare:	8
	Support:	1	HR Practitioner 2:	0	Support:	3
Improving working lives	Healthcare:	4	HR Practitioner 1:	1	Healthcare:	8
	Support:	1	HR Practitioner 2:	0	Support:	3

### Devolution dimension: Decision-making power

The expectations that FLMs, middle managers, and HR practitioners have about the FLMs’ decision-making power partially overlap. All three actors agreed that the FLMs’ decision-making power is concentrated at the operational level. This includes decision-making regarding: the content of the work schedule, the autonomous conduct of selection interviews, performance appraisals, development reviews, absenteeism interviews, coaching conversations (subordinates’ work behavior), and informal conversations (subordinates’ private life and well-being):

“I regularly talk with subordinates about their private lives …. Once I know that, I can support them better and help them doing a better job” (FLM 5, male, support unit 4, ten months of experience as FLM).

Despite these similarities, we discovered a few differences as well. Compared to middle managers, all HR practitioners and support FLMs and the majority of healthcare FLMs (*N* = 7; 88%) stressed the FLMs’ administrative decision-making power. This includes: writing summaries about the conducted conversations, uploading these summaries to the HR system, creating work schedules, approving incoming requests (absences, working hours, holidays, address changes, and declarations). All FLMs also underlined that the time that is needed for the coordination of their teams and shifts goes beyond the work schedule. They not only decide who works when, they allocate tasks to subordinates as well. FLMs do this by making subordinates responsible for the execution of healthcare, support, or HR tasks. The latter refers to deciding which subordinate will guide a newly hired subordinate or organize a formal training. FLMs do this to intellectually challenge their subordinates and, herewith, improve their working life experiences as well.

### Devolution dimension: Financial power

Two different types of purchases were found that indicate the amount of money FLMs are allowed to spend autonomously. First, there are general purchases that directly support the primary healthcare or support process (e.g., the purchase of bandages and tools). FLMs’ financial power for these purchases ranges from €1.000 to €2.500. Second, there are staff purchases which comprise human capital investments. With regard to these purchases, FLMs are allowed to autonomously purchase flowers in case of hiring a new subordinate or a long-term absence. One healthcare middle manager and both HR practitioners included the purchase of post cards in the FLMs’ financial power as well. The healthcare middle manager and healthcare FLM of healthcare unit 7 underlined that the FLM is allowed to purchase a team dinner when subordinates are working overtime. The middle manager and FLMs of healthcare unit 5 are allowed to purchase gifts, such as gift cards, magazines, candy, or celebration cake, for subordinates that leave the unit or in case of an anniversary. Finally, a third of all interviewees mentioned that FLMs are allowed to autonomously hire flex workers in case of understaffing.

### Devolution dimension: Knowledge

FLMs were expected to possess specific knowledge about various topics to execute the devolved HR tasks as intended. However, the relevance of certain types of knowledge varies from topic to topic. The majority of the interviewees (N = 13; 62%) considered expert knowledge about the subordinates’ field of expertise essential for FLMs to successfully execute their devolved HR tasks. This type of knowledge, which also includes the standards for desired and undesired work behavior, will help FLMs to better understand complicated work-related issues, properly manage subordinates, and to support middle managers who often do not possess these expert insights.

Despite this overlap, different role expectations were reported regarding the FLMs’ knowledge about conducting proper conversations. The middle managers and HR practitioners underlined that FLMs should know how they can motivate their subordinates for organizational changes. The HR practitioners added that FLMs should know how they can constructively address undesired work behavior as well. FLMs themselves, however, did not mention these roles, yet indicated that they should know how to successfully conduct in-depth conversations to find the reasoning behind their subordinates’ actions.

A minority of the interviewees, mainly middle managers and HR practitioners, underlined the necessity for FLMs to possess business and labor law knowledge. Business knowledge includes cognitive insights regarding the hospital’s HR and non-HR workflows, how to interpret the unit’s analytical performance reports, and where to find corporate and HR policies. Labor law knowledge captures the basic principles regarding paternity leave, contracts, reintegration, and the collective labor agreement.

### Role ambiguity dimension: Goal/expectation/responsibility ambiguity

This first role ambiguity dimension reveals where the FLMs’ HR responsibilities end. Most healthcare middle managers (*N* = 7; 88%), both HR practitioners, and all healthcare.

FLMs agreed that the healthcare middle manager is responsible for the unit’s finances and performance, setting the unit’s strategic direction by creating the annual strategic plan, and deciding on pay banding (pay banding is the range of pay established by organizations to pay employees performing a particular job or function), and the distribution of bonuses. A slight majority of the interviewees (*N* = 11; 52%) also reported that middle managers are the ones who are responsible for handing out official warnings for intolerable work behavior. The selection of a new subordinate is considered to be a shared responsibility between a certain middle manager and FLM, where the middle manager decides who to hire. The same applies to deciding who goes on training. A handful of healthcare middle managers and FLMs stated that FLMs are in charge of analyzing the training and development needs of their subordinates and communicating these to the middle manager, who decides about who participates to which training:

“*We have to look at that. These decisions affect the training budget … I have to deliberate with my middle manage*r” (FLM 11, female, healthcare unit 7, four years of experience as FLM).

In addition to the HR tasks being conducted by the middle manager, a few HR tasks were conducted by FLMs’ subordinates. To illustrate this, four healthcare FLMs and two support FLMs reported that their subordinates were formally training other subordinates. Five healthcare FLMs and one support FLM used this practice in the light of induction of new subordinates. Although subordinates executed these HR tasks, FLMs were still held accountable by their middle managers for the proper execution of these. Two examples of Goal/Expectation/Responsibility Ambiguity were reported by FLMs. They experienced this type of role ambiguity when they started working as a FLM. One FLM shared:

“*For me, new things [HR tasks] keep appearing … When I arrived here there were no strict rules regarding ‘you do this and I do that’ or ‘who is responsible for what’ … That is sometimes stressful*” (FLM 10, female, healthcare unit 7, one year of experience as FLM).

FLMs reported that they prevent this type of role ambiguity by reading their online accessible job description, indicating the main goal of the FLM position, hierarchical position, attention areas, results to accomplish and maintain, required knowledge, and behavior to enact. Other FLMs overcame their role ambiguity by conducting a short calibration session with the middle manager to clarify who conducts which HR task.

### Role ambiguity dimension: Process ambiguity

For this second role ambiguity dimension, various role expectations were found about how HR tasks should be executed. The first expectation of this kind was regarding the FLMs’ participation in primary healthcare or support processes. A minority of the interviewees expected FLMs to spend 25 percent of their time in these operational processes. The second, frequently mentioned role expectation of this kind was regarding the FLMs’ accomplishment of so-called ‘HR task performance agreements’. These were mentioned by the majority of the interviewees (*N* = 19, 85%). Middle managers and FLMs should formally meet once per 2 weeks. During these bilateral meetings, FLMs are asked to provide a status update on the HR task performance agreements – as set in prior meetings – and to clarify if they can accomplish these in time. An example of such an update would be about the number of conducted performance appraisals. All middle managers stated that the timely accomplishment of these agreements is more important to them than the actual execution:

“*They [FLMs] are free to do it [the execution of devolved HR tasks] the way they want, as long as the result is there*” (Middle Manager 1, female, healthcare unit 1).

Most examples of role ambiguity that were found in this empirical study were related to this specific type of role ambiguity. Most of the ambiguousness was attributed to the unclear wording used in the FLMs’ job description. One support FLM and three healthcare FLMs reported that they did not know when an FLM is ‘participating’ in the healthcare processes. Another example of this type of role ambiguity referred to the absence of HR policies. This absence caused role stress for FLMs as there were no guidelines on how to properly act. A final example of process ambiguity was reported by a healthcare FLM who was obliged by her middle manager to execute a new HR task on a short term, causing her role stress:

“*And now, from April first you will be doing that [HR task]. Oh. Ok. How? Saturday it is April first, meaning that I have to go somewhere next week. And have to do something. I don’t know*” (FLM 8, female, healthcare unit 5, four years of experience as FLM).

FLMs prevented or solved this type of role ambiguity by looking up the hospital’s HR policies if available or by consulting their middle manager or HR practitioner on how to execute their HR task best. These questions would be asked during the bilateral meeting with the middle manager, during the monthly meetings each FLM has with their unit’s affiliated HR practitioner, or by stepping into the middle manager’s or HR’s office.

### Role ambiguity dimension: Priority ambiguity

Expectations regarding the moment or sequence FLMs have to execute their HR tasks encompasses the third role ambiguity dimension. Four healthcare FLMs and one support middle manager stated that the HR task performance agreements, made between middle manager and FLM, determine when HR tasks should be executed. Each of these agreements has a strict deadline, herewith making it perfectly clear for the FLM when to finish an HR task. The HR system also helps FLMs with determining when HR tasks should be executed. FLMs would receive digital announcements stating when and which actions should be taken (conduct of performance appraisals and management of absences). Finally, the work schedules dictate when FLMs work on their HR tasks. Three healthcare FLMs explained how they start their day by determining if the work schedule needs readjustments or not (e.g., absenteeism or unexpected work demands). For this third type of role ambiguity, no examples of role ambiguity were found.

“*The red line is that I have my briefing in the morning. Then I look at the work schedule of that day and the upcoming ones. Then I know what my priorities are. The continuation of the unit, that is top priority*” (FLM 4, male, healthcare unit 3, four years of experience as FLM).

### Role ambiguity dimension: Behavior ambiguity

The behaviors FLMs have to enact during the execution of devolved HR tasks comprise the final role ambiguity dimension. The majority of the interviewees (*N* = 17; 81%) agreed that FLMs should enact role model behavior. This means that the FLM acts according to the behavioral standards prescribed on the hospital and unit level. Enacting these behaviors is considered to be crucial as subordinates are assumed to copy their FLM’s behavior. Another behavioral expectation that was frequently reported dealt with the FLMs’ leadership style. According to a slight majority of the interviewees (*N* = 11, 52%), it is important that FLMs dare to take decisions that could be considered unfavorable by subordinates as well as to address undesired work behavior. This would require a strict, direct, and autocratic leadership style. For some FLMs, enacting this leadership style could be challenging as they were promoted from subordinate to FLM:

“*At first, the FLM was subordinate. One of the gents. Now, the challenge is for this FLM to know how to hierarchically position himself*” (Middle manager 6, male, support unit 6).

Middle managers and HR practitioners held additional role expectations regarding the FLMs’ behavior. They expect FLMs to motivate subordinates for organizational change processes. To do so, FLMs should inform subordinates about the upcoming developments in the unit, explain why these developments are relevant for the unit, and how subordinates could participate in the change process. Furthermore, they underlined the FLMs’ display of personal integrity. This means that FLMs should be capable of fostering and maintaining a trustworthy relationship with both their subordinates and middle manager.

“*I think you [FLM] have to be positive. You must have a positive vibe. You must be able to motivate people. But also take people along … Showing that change can be fun*” (HR practitioner 1, female, affiliated with support unit 4).

One example of behavior ambiguity has been found where a healthcare FLM did not exactly know how to display personal integrity when starting as FLM, causing role stress:

“*Because it is, to be honest, by far one of the toughest positions in the organization. You are sandwiched between everything*” (FLM 4, male, healthcare unit 3, four years of experience as FLM).

For this FLM, having leadership experiences built up in previous jobs helped him overcoming his role ambiguity. Other interviewees mentioned the hospital’s ‘FLM training’ as an important factor in this regard. This training was provided by the HR department and was followed by both healthcare and support unit FLMs. During this training, FLMs received information about leadership, conversation skills, labor law developments, and reading analytical reports. However, the training also brought FLMs together which ignited knowledge and experience sharing:

“*They [the FLMs] started talking with each other about their job. And by doing that, they discovered things like ‘oh, that is great you are doing it like that. I will start doing that in my unit too*” (HR practitioner 1, female, affiliated with support unit 4).

## Discussion

This exploratory interview study aimed at answering the following research question: how do the individual role expectations of FLMs, middle managers, and HR practitioners regarding the FLMs’ devolved HR function differ from one another? By conducting nineteen in-depth semi-structured interviews with 21 respondents (see the Methodology section for more details), the role expectations of eleven FLMs, eight middle managers, and two HR practitioners from a Dutch non-academic hospital were identified. This study relied on theoretical frameworks with respect to frequently-devolved HR tasks (Hutchinson and Purcell, 2010), devolution dimensions (Cascón-Pereira and Valverde, 2014) and role ambiguity dimensions (Bedeian and Armenakis, 1981).

Various role expectation differences were found. Most of these differences concerned the knowledge FLMs need to possess to execute their HR tasks as intended. Each party expects FLMs to possess a different type of knowledge. These expectations appeared to vary from conducting proper conversations with subordinates (FLMs), to the possession of business-related knowledge (middle managers), to knowledge about labor laws (HR practitioners). Furthermore, multiple role expectation differences were directed towards the FLMs’ decision-making power and behavior to enact. Compared to middle managers, FLMs and HR practitioners expect FLMs to underline a larger (administrative) decision-making power. Middle managers and HR practitioners, however, seemed to hold more expectations regarding the behavior FLMs should enact during the execution of their HR tasks.

Another role expectation difference was found in the frequently-devolved HR tasks (Hutchinson and Purcell, 2010). FLMs expect to be involved in a wider range of HR tasks in comparison with middle managers and HR practitioners. FLMs expect to take part in HR tasks related to health and safety and the improvement of working lives as well. Final role expectation differences were found regarding when and how FLMs should execute their HR tasks. Middle managers and HR practitioners reported no specific expectations other than accomplishing set agreements. This in contrast to FLMs themselves, who shared very detailed and hands-on expectations on the actual accomplishment of these agreements.

In their article, Mat and Barrett (2015) relied on an assumption by Katz and Kahn (1978), stating that the role expectations of middle managers and HR practitioners – regarding the devolved HR role of FLMs – are equal to those of FLMs by default. From our empirical work, we conclude that the assumption made by Katz and Kahn (1978) should be nuanced as role expectations regarding FLMs’ HR role, interpreted by the distinguished actors involved, cannot be assumed to be equal. Therefore, stakeholders in working organizations should not deny the presence of role expectation differences and should, instead, actively intervene to mitigate these, as explained in the following section.

In line with scholars, such as Evans (2017), who previously researched role stress among FLMs, various examples of FLMs experiencing role ambiguity and, subsequently, role stress have also been discovered in this empirical work. Most examples of role ambiguity were related to how FLMs should execute their assigned HR tasks. Reasons for experienced role ambiguity were related to unspecific wording in FLMs’ job descriptions, FLMs’ unfamiliarity with newly devolved HR tasks, or absent HR policies. Role stress also appeared to be related to Goal/Expectation/Responsibility Ambiguity and to Behavior Ambiguity. These types of ambiguity occurred most often when operational workers started working as FLM, still being relatively unaware of the HR tasks to execute and behavior to enact. Furthermore, previous devolution literature underlines that decreasing FLMs’ role ambiguity is not only a matter of aligning expectations. Organizational support is also an important factor in the light of reducing role ambiguity (Hutchinson and Purcell, 2010; Showail et al., 2013; Gilbert et al., 2015). In particular, organizational support, as provided by the HR department, is relevant for FLMs as these are expected to possess certain HR knowledge and skills to enact their ascribed HR role as intended (Bos-Nehles et al., 2013).

This study reveals the role clarification interventions that FLMs use to prevent or cope with their role ambiguity. They read their job description, have frequent meetings with their middle manager and HR practitioner, work with clear and timely performance agreements, ask questions, use the HR systems to keep them alert, rely on their previous leadership experiences, and inform themselves with organizational and HR policies. We believe that these interventions contribute to a relatively low level of role ambiguity regarding the execution of HR tasks.

In this study, FLMs were free to divide the workload amongst their subordinates. This also included HR-related tasks on training and development (providing formal training) and induction (showing new subordinates around). This means that the HR role is further cascading down to the work floor. Bondarouk et al. (2018) referred to this phenomenon as the second wave in devolution. The so-called first wave was about the devolution of HR tasks from HR or middle management to FLMs. In the second wave, the HR tasks are further devolved, namely, in the direction of subordinates. However, the purpose for the FLMs (the population under study in our research) to devolve their HR tasks to subordinates slightly differed from the purpose of Bondarouk et al. (2018). In their book Organizational Roadmap to Teal Organizations, Bondarouk et al. (2018) explained how the second wave of devolution can help organizations to enable and strengthen self-managing teams, while our study revealed that FLMs are engaged in HR devolution to intellectually stimulate their sub-ordinates and to improve their working lives. Despite this difference between the scholarly work by Bondarouk et al. (2018) and the empirical work that is reported in this contribution, the upcoming second wave of devolution could have serious implications for the HR role of FLMs and their subordinates. A plausible future would be one wherein the subordinates execute most of the HR tasks themselves and wherein FLMs supervise the process to assure that the role expectations of middle managers and HR practitioners are being realized. This further cascading down to the work floor of HR tasks is further strengthened by the input from “HR intelligence – tools, instruments and data – for Self-Managing Teams which they can use to manage themselves, such as e-HRM systems, dashboard and team development tools” (Renkema et al., 2018, p. 83). This asks from HR professionals a more mature digital mindset (Isari et al., 2019). The above described second wave also relates to the term ‘Strategic Partnerships’, discussed by Jackson et al. (2003), based on the recognition that managing human resources is everyone’s responsibility. They stressed a concept called the ‘HR Triad’, referring to three stakeholders all together responsible for managing human resources: Managers, Employees, and HR Professionals, being useful for understanding the different roles used when managing HRM.

Based on this study’s insights, we propose four interventions for mitigating unwanted role ambiguity. These interventions are focused on FLMs, but in the light of the second wave of devolution (Bondarouk et al., 2018), they are just as well applicable to subordinates. *Firstly*, organizations could help their FLMs by providing access, preferably online, to their job description and the intended HR policies of the specific organization. Middle managers and HR practitioners should embed their role expectations into these documents. Operationalizing the role expectations through clear wording and practical examples could help FLMs to better understand what is expected from them in their HR role enactment. *Secondly*, organizations could use their HR systems and the SMART method (Bovend'Eerdt et al., 2009) for clarifying when FLMs should execute which HR practice. The HR system should direct (ICT-supported) the FLMs by timely notifying when certain actions should be taken. The SMART method, a goal-setting method aimed at specifying results and targets, assures that the HR task performance agreements between middle manager and FLM are Specific, Measurable, Achievable, Relevant, and Timely. *Thirdly*, to ensure that new and experienced FLMs, from skills and behavioral point of view, can enact their ascribed HR tasks, HR practitioners and middle managers should organize recurring ‘FLM workshops’. Inspired by the supervisory intervention of Uduma et al. (2017), these workshops should learn FLMs how to execute their HR tasks, what experience and behavior is expected when enacting their HR role, how the HR systems can be leveraged, where important documents can be found, and how to get access to knowledge and experiences from other FLMs. Role plays, group discussions, and live demonstrations of the HR systems could be suitable methods for these workshops. Presenting an authority continuum, as explained in the work of Blank (2016), could help to clarify the distribution of HR-related tasks amongst middle managers, FLMs, HR practitioners, and other parties. *Fourthly*, in line with publications from Hutchinson and Purcell (2010) and Evans (2017), FLMs’ feedback-seeking behavior helps to reduce role ambiguity effectively. To encourage FLMs to seek valuable feedback when experiencing role ambiguity, Kraut et al. (2015) suggested organizations to reinforce feedback as a habit. This means that organizations, across all hierarchical layers, should integrate feedback into their daily routine. FLMs have to learn about the added value of feedback-seeking behavior and should seek and receive feedback properly through complimenting and correction exercises. Organizations are recommended to include the feedback-seeking behavior aspect in the recurring FLM workshops mentioned in the previous intervention, and to provide FLMs with ample opportunities to enact feedback-seeking behavior (Van Waeyenberg and Decramer, 2018) and thus increase professionalism (Delgado, 2021). The hospital under study in our scholarly work did this by having formal meetings between middle managers and FLMs once every 2 weeks, and between HR practitioners and FLMs once a month. Furthermore, FLMs had the opportunity to informally address questions to the middle manager and their HR practitioner outside these meetings. Managers’ awareness about their HR role contributes not only to their role performance, it also enhances the quality of the relationship with their subordinates (Lam et al., 2015). In case of conflicting role expectations, parties could resort to goal-setting theory (Locke and Latham, 2002) and use practical methods, such as the aforementioned SMART technique, as a starting point for realigning their expectations.

Although this study offers ample insights in the different role expectations of FLMs, middle managers, and HR practitioners, in regards to the FLMs’ HR role, three limitations underlie this study, and urge the need for more empirical work in this field. Firstly, only certain categories of role senders have been included in this study. We do not know whether other types of role senders (such as senior managers, subordinates, colleagues, customers, and suppliers) would have comparable or different role expectations and could add to the knowledge that we have gained – suppliers, in particular, are important role senders given their impact on organizational performance (Lee et al., 2011). Secondly, only two HR practitioners have been included in this study whom only covered halve of the units under study. This raises the question whether outcomes would have become different when additional HR practitioners would have been interviewed. Third, the duration of our interviews was, due to practical and incidental occurrences, highly diverse and varied from 15 to 71 min. Although the quality of our data collection has never been at stake, we do recommend future researchers to schedule at least 40 min for conducting each interview as it provides sufficient time for addressing all necessary questions and asking follow-up questions.

In line with these limitations, researchers could expand the current field of HR devolution research by measuring the role expectations of unstudied role senders. For instance, the role expectations of senior managers and subordinates. By comparing these with existing findings, undiscovered overlap and differences in role expectations can be made salient (Evans, 2017). Researchers are advised to pay close attention to the upcoming HR role of subordinates (Bondarouk et al., 2018) as this could have implications for the HR tasks being executed by FLMs and, thus, for the role expectations in this regard. We also advice researchers to use the devolution dimensions (Cascón-Pereira and Valverde, 2014) and role ambiguity dimensions (Bedeian and Armenakis, 1981) as a combined measure for exploring role expectation differences. These two frameworks are, in our eyes, complementary and highly valuable for the identification of the FLMs’ HR role (devolution dimensions) and the execution of these (role ambiguity dimensions). The frequently devolved HR tasks that were distinguished by Hutchinson and Purcell (2010) and used in our research helped our respondents to define the FLMs’ HR role and to specify their role expectations which, we believe, enhanced internal validity. As Mat and Barrett (2015) considered the content of role expectations to be dependent on organizational size, researchers are being advised to replicate this research in an organization, preferably healthcare, of similar size, and to investigate the generalizability and/or differences in comparison with organizations with different sizes. Nonetheless, generalizability challenges mentioned by Carminati (2018) must be accounted for (i.e., using a congruent terminology and following systematic procedures).

## Conclusion

FLMs fulfil a crucial, mediating role within the causal relationship between intended HR practices and organizational performance. They are the managerial layer closest to the workforce (Nehles et al., 2006), they fulfil an HR role worth mentioning (Cascón-Pereira and Valverde, 2014), and they are located in a paradoxical, intermediate, “piggy in the middle” position (Gilbert et al., 2011b, p. 565), that is between strategic and operational layers. Therefore, a rising number of studies, including this one, underline that FLMs are running considerable risks on experiencing role stress in regards to the execution of their HR role (Evans, 2017). It is up to the middle managers and HR practitioners to define, align, and properly communicate their role expectations regarding the FLMs’ HR role and, through this, to secure the relationship between HRM and organizational performance (Wright and Nishii, 2013). This study was meant to come up with some sound, evidence-based recommendations in this regard.

## Data availability statement

The anonymized data supporting the conclusions of this article and the interview protocol will be made available by the authors, without undue reservation.

## Ethics statement

Ethical review and approval was not required for the study on human participants in accordance with the local legislation and institutional requirements. Written informed consent for participation was not required for this study in accordance with the national legislation and the institutional requirements. The participants were informed and verbal consent was obtained prior to the data collection.

## Author contributions

MW and JB: conceptualization, methodology, validation, and writing—original draft preparation. MW: software, formal analysis, investigation, resources, data curation, visualization, and project administration. MW, JB, and BVdH: writing—review and editing. JB and BVdH: supervision. All authors contributed to the article and approved the submitted version.

## Conflict of interest

The authors declare that the research was conducted in the absence of any commercial or financial relationships that could be construed as a potential conflict of interest.

## Publisher’s note

All claims expressed in this article are solely those of the authors and do not necessarily represent those of their affiliated organizations, or those of the publisher, the editors and the reviewers. Any product that may be evaluated in this article, or claim that may be made by its manufacturer, is not guaranteed or endorsed by the publisher.

## Supplementary material

The Supplementary material for this article can be found online at: https://www.frontiersin.org/articles/10.3389/fpsyg.2022.951359/full#supplementary-material

## References

[ref1] Andersen K. K. Cooper B. K., and, Zhu C. J. (2007). The effect of SHRM practices on perceived firm financial performance: some initial evidence from Australia. Asia Pac. J. Hum. Resour. 45, 168–179. doi: 10.1177/1038411107079111

[ref2] Andolsek D. M. Stebe J. (2005). Devolution or (de) centralization of HRM function in European organizations. Int. J. Hum. Resour. Manag. 16, 311–329. doi: 10.1080/0958519042000339525

[ref3] Banton M. (1965). Roles: an Introduction to the Study of Social Relations. London: Tavistock.

[ref4] Bauer J. C. Simmons P. (2000). Role ambiguity: a review and integration of the literature. J. Mod. Bus. 3, 41–47.

[ref5] Bedeian A. G. Armenakis A. A. (1981). A path-analytic study of the consequences of role conflict and ambiguity. Acad. Manag. J. 24, 417–424. doi: 10.5465/255852, PMID: 10251671

[ref6] Beijer S. Van De Voorde K. Tims M. (2019). An interpersonal perspective on HR attributions: examining the role of line managers, coworkers, and similarity in work- related motivations. Front. Psychol. 10:1509. doi: 10.3389/fpsyg.2019.01509, PMID: 31312161 PMC6614339

[ref7] Biddle B. J. (1979). Role Theory: Expectations, Identities, and Behaviors. New York, NY: Academic.

[ref8] Biddle B. J. (1986). Recent developments in role theory. Annu. Rev. Sociol. 12, 67–92. doi: 10.1146/annurev.so.12.080186.000435

[ref9] Blank W. (2016). “Principles of management,” in Management and Leadership Skills for Medical Faculty eds. Viera A. J. Kramer R. (New York: Springer), 65–76.

[ref10] Blayney C. Cormier-MacBurnie P. Young J. D. (2020). The devolution of human resource management to line managers: a preliminary examination of the hotel industry in Halifax, Canada. J. Hum. Resour. Hosp. Tour. 19, 443–472. doi: 10.1080/15332845.2020.1763760

[ref11] Bondarouk T. Bos-Nehles A. Renkema M. Meijerink J. De Leede J. (2018). Organisational Roadmap Towards Teal Organisations. Bingley: Emerald Group Publishing.

[ref12] Boon C. Den Hartog D. N. Boselie P. Paauwe J. (2011). The relationship between perceptions of HR practices and employee outcomes: examining the role of person- organisation and person-job fit. Int. J. Hum. Resour. Manag. 22, 138–162. doi: 10.1080/09585192.2011.538978

[ref13] Bos-Nehles A. C. Van Riemsdijk M. J. Looise J. C. (2013). Employee perceptions of line management performance: applying the AMO theory to explain the effectiveness of line managers’ HRM implementation. Hum. Resour. Manag. 52, 861–877. doi: 10.1002/hrm.21578

[ref14] Bovend'Eerdt T. J. Botell R. E. Wade D. T. (2009). Writing SMART rehabilitation goals and achieving goal attainment scaling: a practical guide. Clin. Rehabil. 23, 352–361. doi: 10.1177/0269215508101741, PMID: 19237435

[ref15] Bowen D. E. Ostroff C. (2004). Understanding HRM-firm performance linkages: the role of “strength” of the HRM system. Acad. Manag. Rev. 29, 203–221. doi: 10.2307/20159029

[ref16] Boxall P. (1996). The strategic HRM debate and the resource-based view of the firm. Hum. Resour. Manag. J. 6, 59–75. doi: 10.1111/j.1748-8583.1996.tb00412.x

[ref17] Brewster C. Gollan P. J. Wright P. M. (2013). Guest editors’ note: human resource management and the line. Hum. Resour. Manag. 52, 829–838. doi: 10.1002/hrm.21594

[ref18] Brewster C. Larsen H. H. (2000). “Responsibility in human resource management: the role of the line,” in Human Resource Management in Northern Europe. eds. Brewster C. Larsen H. H. (Oxford: Blackwells), 195–218.

[ref19] Brockbank W. Ulrich D. (2003). Competencies for the New HR. Arlington: Society of Human Resource Management.

[ref20] Bryman A. (2016). Social Research Methods (5th Edn). Oxford: Oxford University Press.

[ref21] Carminati L. (2018). Generalizability in qualitative research: a tale of two traditions. Qual. Health Res. 28, 2094–2101. doi: 10.1177/1049732318788379, PMID: 30043686

[ref22] Cascón-Pereira R. Valverde M. (2014). HRM devolution to middle managers: dimension identification. BRQ Bus. Res. Q. 17, 149–160. doi: 10.1016/j.brq.2013.05.001

[ref23] Chang S. M. Budhwar P. Crawshaw J. (2021). The emergence of value-based leadership behavior at the frontline of management: a role theory perspective and future research agenda. Front. Psychol. 12:635106. doi: 10.3389/fpsyg.2021.635106 34113282 PMC8185066

[ref24] Colakoglu S. Lepak D. P. Hong Y. (2006). Measuring HRM effectiveness: considering multiple stakeholders in a global context. Hum. Resour. Manag. Rev. 16, 209–218. doi: 10.1016/j.hrmr.2006.03.003

[ref25] Conway E. Monks K. (2010). The devolution of HRM to middle managers in the Irish health service. Pers. Rev. 39, 361–374. doi: 10.1108/00483481011030548

[ref26] Corbin J. Morse J. M. (2003). The unstructured interactive interview: issues of reciprocity and risks when dealing with sensitive topics. Qual. Inq. 9, 335–354. doi: 10.1177/1077800403009003001

[ref27] Delgado J. (2021). Vulnerability as a key concept in relational patient-centered professionalism. Med. Health Care Philos. 24, 155–172. doi: 10.1007/s11019-020-09995-8, PMID: 33423192

[ref28] Dewettinck K. Vroonen W. (2017). Antecedents and consequences of performance management enactment by front-line managers. Evidence from Belgium. Int. J. Hum. Resour. Manag. 28, 2473–2502. doi: 10.1080/09585192.2015.1137608

[ref29] Do H. Budhwar P. S. Patel C. (2018). Relationship between innovation-led HR policy, strategy, and firm performance: a serial mediation investigation. Hum. Resour. Manag. 57, 1271–1284. doi: 10.1002/hrm.21903

[ref30] Dorenbosch L. De Reuver R. Sanders K. (2006). Getting the HR message across: the linkage between line–HR consensus and “commitment strength” among hospital employees. Manag. Rev. 17, 274–291. doi: 10.5771/0935-9915-2006-3-274

[ref31] Dougherty T. W. Pritchard R. D. (1985). The measurement of role variables: exploratory examination of a new approach. Organ. Behav. Hum. Decis. Process. 35, 141–155. doi: 10.1016/0749-5978(85)90032-9

[ref32] Evans S. (2017). HRM and front line managers: the influence of role stress. Int. J. Hum. Resour. Manag. 28, 3128–3148. doi: 10.1080/09585192.2016.1146786

[ref01] Fereday J. Muir-Cochrane E. (2006). Demonstrating rigor using thematic analysis: a hybrid approach of inductive and deductive coding and theme development. Int. J. Qual. Methods 5, 80–92.

[ref33] Finegold D. Frenkel S. (2006). Managing people where people really matter: the management of human resources in biotech companies. Int. J. Hum. Resour. Manag. 17, 1–24. doi: 10.1080/09585190500366169

[ref34] Fombrun C.J. Tichy M.M. Devanna M. A. (1984). Strategic Human Resource Management, New York: John Wiley.

[ref35] Genrich M. Worringer B. Angerer P. Müller A. (2020). Hospital medical and nursing managers’ perspectives on health-related work design interventions. A qualitative study. Front. Psychol. 11:869. doi: 10.3389/fpsyg.2020.00869, PMID: 32431651 PMC7214727

[ref36] Gilbert C. De Winne S. Sels L. (2011a). The influence of line managers and HR department on employees' affective commitment. Int. J. Hum. Resour. Manag. 22, 1618–1637. doi: 10.1080/09585192.2011.565646

[ref37] Gilbert C. De Winne S. Sels L. (2011b). Antecedents of front-line managers’ perceptions of HR role stressors. Pers. Rev. 40, 549–569. doi: 10.1108/00483481111154432

[ref38] Gilbert C. De Winne S. Sels L. (2015). Strong HRM processes and line managers' effective HRM implementation: a balanced view. Hum. Resour. Manag. J. 25, 600–616. doi: 10.1111/1748-8583.12088

[ref39] Glegg S. M. (2019). Facilitating interviews in qualitative research with visual tools: a typology. Qual. Health Res. 29, 301–310. doi: 10.1177/1049732318786485, PMID: 29986623

[ref41] Guba E. G. (1981). Criteria for assessing the trustworthiness of naturalistic inquiries. Educ. Commun. Technol. J. 29, 75–91. doi: 10.1007/BF02766777

[ref02] Hales C. (2005). Rooted in supervision, branching into management: continuity and change in the role of first‐line manager. J. Manag. Stud. 42, 471–506.

[ref42] Harris L. Doughty D. Kirk S. (2002). The devolution of HR responsibilities– perspectives from the UK’s public sector. J. Eur. Ind. Train. 26, 218–229. doi: 10.1108/03090590210424894

[ref43] Hill E. J. (2005). Work-family facilitation and conflict, working fathers and mothers, work- family stressors and support. J. Fam. Issues 26, 793–819. doi: 10.1177/0192513X05277542

[ref44] Hodgson J. (1995). Blind duty versus moral duty. Valpo Core Read. 249, 80–82.

[ref45] Hoogendoorn J. Brewster C. (1992). Human resource aspects: decentralization and devolution. Pers. Rev. 21, 4–11. doi: 10.1108/00483489210009075

[ref46] Hutchinson S. Purcell J. (2010). Managing ward managers for roles in HRM in the NHS: overworked and under-resourced. Hum. Resour. Manag. J. 20, 357–374. doi: 10.1111/j.1748-8583.2010.00141.x

[ref47] Isari D. Bissola R. Imperatori B. (2019). “HR devolution in the digital era: what should we expect? in HRM 4.0 For Human-Centered Organizations Advanced Series in Management. *Vol*. 23. (Bingley: Emerald Publishing Limited), 41–61.

[ref48] Jackson S. E. Luo Y. Schuler R. S. (2003). Managing Human Resources in Cross-Border Alliances. London: Routledge.

[ref49] Jong J. (2016). The role of performance feedback and job autonomy in mitigating the negative effect of role ambiguity on employee satisfaction. Public Perform. Manag. Rev. 39, 814–834. doi: 10.1080/15309576.2015.1137771

[ref50] Kabene S. M. Orchard C. Howard J. M. Soriano M. A. Leduc R. (2006). The importance of human resources management in health care: a global context. Hum. Resour. Health 4, 1–17. doi: 10.1186/1478-4491-4-20 16872531 PMC1552082

[ref51] Katz D. Kahn R. L. (1978). The Social Psychology of Organizations. Vol. 2. New York: Wiley.

[ref52] Keen L. Vickerstaff S. A. (1997). ‘We’re all human resource managers now’: local government middle managers. Public Money Manag. 17, 41–46. doi: 10.1111/1467-9302.00081

[ref53] Khilji S. E. Wang X. (2006). ‘Intended’ and ‘implemented’ HRM: the missing linchpin in strategic human resource management research. Int. J. Hum. Resour. Manag. 17, 1171–1189. doi: 10.1080/09585190600756384

[ref54] Kou X. Kurdi-Nakra H. Pak J. (2022). The framework of first-line manager’s HR role identity: a multi-actor HR involvement perspective. Hum. Resour. Manag. Rev. 32:100898. doi: 10.1016/j.hrmr.2022.100898

[ref55] Kraut A. Yarris L. M. Sargeant J. (2015). Feedback: cultivating a positive culture. J. Grad. Med. Educ. 7, 262–264. doi: 10.4300/JGME-D-15-00103.1, PMID: 26221448 PMC4512803

[ref56] Kurdi-Nakra H. Kou X. Pak J. (2022). The road taken and the path forward for HR devolution research: an evolutionary review. Hum. Resour. Manag. 61, 239–258. doi: 10.1002/hrm.22091

[ref57] Lam M. O’Donnell M. Roberson D. (2015). Achieving employeee commitment for continuous improvement initiatives. J. Operat. Prod. Manag. 35, 201–215. doi: 10.1108/IJOPM-03-2013-0134

[ref58] Lee S. M. DonHee L. Schniederjans M. J. (2011). Supply chain innovation and organizational performance in the healthcare industry. Journal of Operations & Production Management 31, 1193–1214. doi: 10.1108/01443571111178493

[ref59] LePine M. A. Zhang Y. Crawford E. R. Rich B. L. (2016). Turning their pain to gain: charismatic leader influence on follower stress appraisal and job performance. Acad. Manag. J. 59, 1036–1059. doi: 10.5465/amj.2013.0778

[ref60] Locke E. A. Latham G. P. (2002). Building a practically useful theory of goal setting and task motivation: A 35-year odyssey. Am. Psychol. 57, 705–717. doi: 10.1037/0003-066X.57.9.705, PMID: 12237980

[ref61] Lowe J. (1992). Locating the line: the front-line supervisor and human resource management. Reassess. Hum. Resour. Manag. 20, 148–168.

[ref62] Lyons T. F. (1971). Role clarity, need for clarity, satisfaction, tension, and withdrawal. Organ. Behav. Hum. Perform. 6, 99–110. doi: 10.1016/0030-5073(71)90007-9

[ref63] Mat N. H. N. Barrett R. (2015). Understanding the line managers’ HRM role expectations: does size matter? Asian Soc. Sci. 11:118. doi: 10.5539/ass.v11n16p118

[ref64] Maxwell J. A. (2009). “Designing a qualitative study,” in The SAGE Handbook of Applied Social Research Methods. eds. Bickman L. Rog D. J. (SAGE Publications, Inc.), 214–253.

[ref65] McConville T. (2006). Devolved HRM responsibilities, middle-managers and role dissonance. Pers. Rev. 35, 637–653. doi: 10.1108/00483480610702700

[ref66] McConville T. Holden L. (1999). The filling in the sandwich: HRM and middle managers in the health sector. Pers. Rev. 28, 406–424. doi: 10.1108/00483489910286738

[ref67] McDermott A. M. Fitzgerald L. Van Gestel N. M. Keating M. A. (2015). From bipartite to tripartite devolved HRM in professional service contexts: evidence from hospitals in three countries. Hum. Resour. Manag. 54, 813–831. doi: 10.1002/hrm.21728

[ref68] McLain D. L. Kefallonitis E. Armani K. (2015). Ambiguity tolerance in organizations: definitional clarification and perspectives on future research. Front. Psychol. 6:344. doi: 10.3389/fpsyg.2015.00344 25972818 PMC4411993

[ref69] Miner J. B. (1971). Management Theory. London: Macmillan.

[ref03] Morley M. J. Gunnigle P. O’Sullivan M. Collings D. G. (2006). New directions in the roles and responsibilities of the HRM function. Pers. Rev. 35, 609–617. doi: 10.1108/00483480610702683

[ref70] Nehles A. C. Van Riemsdijk M. J. Kok I. Looise J. C. (2006). Implementing human resource management successfully: the role of first-line managers. Manag. Rev. 17, 256–273. doi: 10.5771/0935-9915-2006-3-256

[ref71] Op de Beeck S. Wynen J. Hondeghem A. (2016). HRM implementation by line managers: explaining the discrepancy in HR-line perceptions of HR devolution. Int. J. Hum. Resour. Manag. 27, 1901–1919. doi: 10.1080/09585192.2015.1088562

[ref72] Papalexandris N. Panayotopoulou L. (2005). Exploring the partnership between line managers and HRM in Greece. J. Eur. Ind. Train. 29, 281–291. doi: 10.1108/03090590510597133

[ref73] Purcell J. Hutchinson S. (2007). Front-line managers as agents in the HRM-performance causal chain: theory, analysis and evidence. Hum. Resour. Manag. J. 17, 3–20. doi: 10.1111/j.1748-8583.2007.00022.x

[ref74] Qadeer F. (2011). An overview of HR-line relationship and its future directions. Afr. J. Bus. Manag. 5, 2512–2523.

[ref04] Renkema M. Bondarouk T. Bos-Nehles A. (2018). Transformation to self-managing teams: lessons learned: a look at current trends and data. Strategic HR Review 17, 81–84. doi: 10.1108/SHR-10-2017-0072

[ref75] Richard O. C. Johnson N. B. (2001). Strategic human resource management effectiveness and firm performance. Int. J. Hum. Resour. Manag. 12, 299–310. doi: 10.1080/09585190121674

[ref76] Sanders K. Frenkel S. (2011). HR-line management relations: characteristics and effects. Int. J. Hum. Resour. Manag. 22, 1611–1617. doi: 10.1080/09585192.2011.565644

[ref77] Sawyer J. E. (1992). Goal and process clarity: specification of multiple constructs of role ambiguity and a structural equation model of their antecedents and consequences. J. Appl. Psychol. 77, 130–142. doi: 10.1037/0021-9010.77.2.130

[ref78] Sheehan M. (2012). Devolvement of HRM and perceived performance within multinational corporations (MNCs). Eur. J. Int. Manag. 6, 101–127. doi: 10.1504/EJIM.2012.044760

[ref79] Shenton A. K. (2004). Strategies for ensuring trustworthiness in qualitative research projects. Educ. Inf. 22, 63–75. doi: 10.3233/EFI-2004-22201

[ref80] Showail S. J. McLean Parks J. Smith F. L. (2013). Foreign workers in Saudi Arabia: a field study of role ambiguity, identification, information-seeking, organizational support and performance. Int. J. Hum. Resour. Manag. 24, 3957–3979. doi: 10.1080/09585192.2013.781521

[ref81] Sisson K. Storey J. (2000). Realities of Human Resource Management: Managing the Employment Relationship. London: McGraw-Hill Education.

[ref82] Smith C. S. Brannick M. T. (1990). A role and expectancy model of participative decision-making: a replication and theoretical extension. J. Organ. Behav. 11, 91–104. doi: 10.1002/job.4030110202

[ref83] Solomon M. R. Surprenant C. Czepiel J. A. Gutman E. G. (1985). A role theory perspective on dyadic interactions: the service encounter. J. Mark. 49, 99–111. doi: 10.1177/002224298504900110

[ref84] Stanton P. Young S. Bartram T. Leggat S. G. (2010). Singing the same song: translating HRM messages across management hierarchies in Australian hospitals. Int. J. Hum. Resour. Manag. 21, 567–581. doi: 10.1080/09585191003612075

[ref85] Tamayo-Verleene K. G. (2021). *Line Manager Involvement in HRM: Advancing Theory and Evidence from Philippine Call Centers*. Ph.D. theses, Ku Leuven, Leuven.

[ref86] The RBL Group (2015). *HRCS Round 7: Creating HR Value from the Outside-In*. Available at: https://rbl.net/s3/?rblip/HRCS/pdf/hrcs-7-report.pdf (Accessed September 24, 2021).

[ref87] Thies C. G. (2013). The United States, Israel and the Search for International Order: Socializing States. New York: Routledge

[ref88] Townsend K. Dundon T. Cafferkey K. Kilroy J. (2022). Victim or master of HRM implementation: the frontline manager conundrum. Asia Pac. J. Hum. Resour. 60, 79–96. doi: 10.1111/1744-7941.12311

[ref89] Townsend K. Dundon T. Loudoun R. (2015). The front-line manager’s role in informal voice pathways. Empl. Relat. 37, 475–486. doi: 10.1108/ER-06-2014-0060

[ref90] Townsend K. Wilkinson A. Bamber G. Allan C. (2012). Accidental, unprepared, and unsupported: clinical nurses becoming managers. Int. J. Hum. Resour. Manag. 23, 204–220. doi: 10.1080/09585192.2011.610963

[ref91] Tubre T. C. Collins J. M. (2000). Jackson and Schuler (1985) revisited: a meta-analysis of the relationships between role ambiguity, role conflict, and job performance. J. Manag. 26, 155–169. doi: 10.1177/014920630002600104

[ref92] Tyskbo D. (2020). Line management involvement in performance appraisal work: toward a practice-based understanding. Empl. Relat. 42, 818–844. doi: 10.1108/ER-06-2019-0236

[ref93] Uduma O. Galligan M. Mollel H. Masanja H. Bradley S. McAuliffe E. (2017). The impact of a human resource management intervention on the capacity of supervisors to support and supervise their staff at health facility level. Hum. Resour. Health 15, 1–16. doi: 10.1186/s12960-017-0225-0 28854937 PMC5577784

[ref94] Ulrich D. Brockbank W. Johnson D. Younger J. (2007). Human resource competencies: responding to increased expectations. Employ. Relat. Today 34, 1–12. doi: 10.1002/ert.20159

[ref95] Ulrich D. Brockbank W. Ulrich M. Kryscynski D. Slade J. (2015). *Human Resource Competency Conference 2016*. Available at: http://www.apg.pt/downloads/file954_pt.pdf (Accessed January 24, 2017).

[ref96] Ulrich D. Brockbank W. Yeung A. Lake D. (1995). Human resource competencies: an empirical assessment. Hum. Resour. Manag. 34, 473–495. doi: 10.1002/hrm.3930340402

[ref97] Ulrich D. Younger J. Brockbank W. Ulrich M. D. (2013). The state of the HR profession. Hum. Resour. Manag. 52, 457–471. doi: 10.1002/hrm.21536

[ref98] Van Waeyenberg T. Decramer A. (2018). Line managers’ AMO to manage employees’ performance: the route to effective and satisfying performance management. Int. J. Hum. Resour. Manag. 29, 3093–3114. doi: 10.1080/09585192.2018.1445656

[ref99] Wright P. M. Nishii L. H. (2013). “Strategic HRM and organizational behavior: integrating multiple levels of analysis,” in 2012 *HRM and Performance: Achievements and Challenges*. eds. Guest D. E. Paauwe J. Wright P. M. (Chichester: Wiley), 97–110.

[ref100] Wu G. Hu Z. Zheng J. (2019). Role stress, job burnout, and job performance in construction project managers: the moderating role of career calling. Int. J. Environ. Res. Public Health 16:2394. doi: 10.3390/ijerph16132394, PMID: 31284496 PMC6651169

