# Supplementary material for: “Expecting the unexpected?” Uncovering role expectation differences in a Dutch hospital
Source: Front Psychol. 2022 Sep 30;13:951359. doi: 10.3389/fpsyg.2022.951359 (PMC9561906; doi:10.3389/fpsyg.2022.951359)
Supplement: Supplementary file 1 [file Data_Sheet_1.docx]

Contents

[Transcript Interview Middle Manager 1 2](#_Toc112244231)

[Transcript Interview Middle Manager 2 6](#_Toc112244232)

[Transcript Interview Middle Manager 3 12](#_Toc112244233)

[Transcript Interview Middle Manager 4 18](#_Toc112244234)

[Transcript Interview Middle Manager 5 25](#_Toc112244235)

[Transcript Interview Middle Manager 6 31](#_Toc112244236)

[Transcript Interview Middle Manager 7 and FLM 11 43](#_Toc112244237)

[Transcript Interview Middle Manager 8 58](#_Toc112244238)

[Transcript Interview HR practitioner 1 67](#_Toc112244239)

[Transcript Interview HR practitioner 2 81](#_Toc112244240)

[Transcript Interview FLM 1 and FLM2 95](#_Toc112244241)

[Transcript Interview FLM 3 103](#_Toc112244242)

[Transcript Interview FLM 4 110](#_Toc112244243)

[Transcript interview FLM 5 123](#_Toc112244244)

[Transcript Interview FLM 6 135](#_Toc112244245)

[Transcript Interview FLM 7 150](#_Toc112244246)

[Transcript Interview FLM 8 163](#_Toc112244247)

[Transcript Interview FLM 9 175](#_Toc112244248)

[Transcript Interview FLM 10 183](#_Toc112244249)

# Transcript Interview Middle Manager 1

**Duur interview: 15 minuten en 05 seconden**

**Functie: Bedrijfsleider**

**Afdeling(en) onder supervisie: Healthcare**

**Gender: vrouw**

**Werkervaring in functie: n.a.**

**Interviewer**: Dan is eigenlijk mijn eerste vraag: wat zijn de dagelijkse werkzaamheden van een teamleider hier binnen het ziekenhuis?

**Respondent:** Dat is een goede vraag. Ik heb op dit moment geen teamleider. Ik werf er eentje op dit moment. De teamleider op dit moment met name alles operationeel op de afdeling te regelen. Roosters, projectjes, het waarnemen van de bedrijfsleider als die op vakantie is en dan in de korte zin van het woord en niet in de eindverantwoordelijke zin want daar moeten we altijd een collega bedrijfsleiding voor regelen, maar de teamleider kan wel in alle operationele zaken de afstemming doen. Kan ook alle ziek- en herstelmeldingen doen, jaargesprekken doen. Er zijn dus wel een tal van taken die een teamleider wat dat betreft kan oppakken.

**Interviewer**: Zoals ik het dan goed hoor, zit daar ook veel HR-gerelateerde taken in het pakket van zo'n teamleider. Wat zit u als HR taken van deze persoon?

**Respondent:** Klopt. Jaargesprekken, eindproeftijdbeoordelingen, selectiegesprekken. Dat zou nog een discussiepunt kunnen zijn om officiële waarschuwingen, dat is normaal gesproken iets van een bedrijfsleider. Ik weet alleen wel dat er bepaalde collega's van mij zijn die daar ook teamleiders bij betrekken. Maar dat is ook afhankelijk van het niveau van de teamleider. Zo zou ik er ook in staan. Ik zou niet dit per definitie niet iets vinden dat een teamleider zou moeten doen.

**Interviewer**: Als we dan kijken naar welke verantwoordelijkheden zo'n teamleider dan heeft, met die HR taken, waarover zou zo'n iemand dan mogen beslissen volgens u. Waar ligt de grens?

**Respondent:** Dat is wel een hele lastige vraag denk ik. Ja dat is wel lastig. Want zoals hier zit ik op een hele specifieke afdeling, dat ik is heel hoog gekwalificeerd personeel. Dat is heel anders dan een afdeling interne. De vraag is of iemand op een intensive care dezelfde beoordelingen zou kunnen doen dan op een algemene afdeling. Dat hoeft niet per definitie zo te kunnen zijn. In principe denk ik dat je prima een teamleider kan in eerste lijn keuzes maken, maar zal niet eindverantwoordelijk zijn. Dat zal ten aller tijden een bedrijfsleider moeten zijn. En dat is ook wel waarom we het ingericht hebben dat als er tijdens bijvoorbeeld mijn afwezigheid in een vakantie iets zou gebeuren met een van mijn medewerkers, dat die bijvoorbeeld een officiële waarschuwing zou moeten krijgen, dan kan diegene wel het traject inzetten maar kan diegene uiteindelijk niet de officiële waarschuwing uitdelen.

**Interviewer**: Dat komt dan uiteindelijk toch weer bij u terug?

**Respondent:** Ja, of bij een waarnemer van mij.

**Interviewer**: Als we dan kijken naar teamleiders die minder geschoold zijn dan hier op de afdeling, waar zou dan het verschil liggen. Zou u dan aan kunnen geven waar ongeveer het verschil ligt in verantwoordelijkheden.

**Respondent:** Ik denk juist dat mensen hier meer geschoold zijn. Maar dat het lastige stuk in de verschillende afdelingen zit dat je te maken hebt met ander soort personeel. Daar moet je als teamleider dus wat standvastiger in je schoenen staan. Als we het namelijk hebben over het aansturen van een poliassistente of iemand aan het aansturen bent die ver boven HBO-niveau zit aan het aansturen bent. De manier waarop een teamleider, stel dat die er zit, met mijn dokters te maken heeft of met een assistent van de chirurgie, dat is toch een andere verhouding.

**Interviewer**: Dus u kijkt dat even wat de situatie is, wat voor soort personen zich in de situatie bevinden en op basis daarvan zou u dan verantwoordelijkheden bij een teamleider neerleggen?

**Respondent:** Ja, en waarschijnlijk zou ik ook, dat is waarom bij mij misschien de functie ook nog niet is ingevuld, omdat ik wel wat andere eisen heb aan een teamleider dan in een afdeling interne, >AFDELING< of >AFDELING<.

**Interviewer**: Wat maken uw eisen anders? Zijn uw eisen hoger?

**Respondent:** Ik weet dat er nog wel eens concessies worden gedaan over het opleidingsniveau. Dat is tegenwoordig steeds minder. We willen echt HBO-geschoold personeel hebben. Maar ik zoek met recht iemand die managementervaring heeft. Ik weet dat hier ook wel mensen in huis aangenomen worden die niet per definitie ervaring te hoeven hebben als teamleider of managementervaring, terwijl ik dat eigenlijk wel een pré vindt als mensen dat hebben omdat je hier toch tegen personeel aankijkt dat echt wel redelijk zelfstandig en standvastig zijn en meer kennis van zaken hebben.

**Interviewer**: Zou zo'n teamleider, afgezien van verantwoordelijkheden, eventueel ook nog een financiële verantwoordelijkheid krijgen. Of komt uiteindelijk alles wat kosten met zich meebrengt bij u terecht voor een akkoord?

**Respondent:** Daar hebben we onderscheid in. Op dit moment is het zo dat teamleiders voor 2.500 euro tekenbevoegd zijn. Daarboven alleen maar een bedrijfsleider. Dus zoals op dit moment neem ik ook de poli >AFDELING< en >AFDELING< waar, en voor morgen is een teamleider van de >AFDELING< gekomen want er moest iets getekend worden met een hoger bedrag dan 2.500 euro. Dan mag zij niet zelf tekenen, dan komt ze bij mij, als waarnemer van haar eigen bedrijfsleider, en teken ik voor haar.

**Interviewer**: Als we dan kijken naar de uitvoer van de HRM activiteiten, wanneer is het naar uw mening naar behoren uitgevoerd. Heeft u daar een bepaalde graadmeter voor, of een soort standaard waar u naar refereert: nu vind ik het naar behoren?

**Respondent:** Dat een teamleider zijn of taken uitvoert?

**Interviewer**: Ja.

**Respondent:** Ik heb mijn organisatie hier redelijk strak ingericht dus ik heb, ter vergelijking een kwaliteitsmedewerker en een praktijkopleider. Dit moeten bijvoorbeeld, aan de hand van standaard formats, met mij evalueren. Daar ik heb ik bilateraal overleggen mee en degene die net haar hoofd door de deur stak dat is de kwaliteitsmedewerker. Dit heeft om de week een overleg met mij aan de hand van een vaste agenda. En dan moet ze over haar voortgang rapporteren. Op die manier borgen en monitoren we de taken en zaken die zij onder haar hoede heeft. De planning is ook om dat zo met een teamleider te doen. Die teamleider krijgt ook een eigen takenpakket zodat het ook weer niet strookt met wat een kwaliteitsmedewerker doet. Anders krijg je daar weer dubbelingen in zegmaar. En ook daarvan zullen we een vaste structuur afstemmen over hoe we de afstemming samen besluit, bekijken en beoordelen. En ook kijken hoe is de voortgang daarin.

**Interviewer**: Die standaarden, kijkt u dan al gauw naar medische standaarden? Waar houdt u zich dan aan vast.

**Respondent:** Nee, zeker geen medische standaarden. Want opzicht zal een teamleider ten aanzien van medisch eigenlijk niet iets doen. Dat is meer ten aanzien van het totale takenpakket en afspreken die je daarin samen formuleert. Naar mijn idee zou een teamleider echt wel jaargesprekken moeten gaan doen. De frequent-verzuimgesprekken, het rooster onder zijn of haar beheer, een aantal projecten moeten gaan draaien. Afhankelijk van hoe ver iemand is zou die ook projectmatig ingezet kunnen worden. En daarop zou ik zo iemand ook betoetsen. Zo iemand kan daar ook inzage in geven middels een dashboard wat de voortgang is ten aanzien van jaargesprekken of verzuimgesprekken. Er zijn ook, ik heb één re-integrant. Zo'n iemand zou een teamleider prima kunnen begeleiden. Dit zou mij de tijd geven om weer op andere zaken te focussen.

**Interviewer**: Is er dan nog bepaalde kennis die u belangrijk vindt waarover zo'n teamleider dan beschikt voor het uitvoeren van de HR-taken. Zijn er bepaalde dingen die echt 'need-to-knows' zijn?

**Respondent:** Als het gaat om HRM moet je echt wel de processen weten. Hoe lopen nou eigen de processen ten aanzien van de aanname van personeel. Wat komt daarbij kijken? Op deze afdeling ben ik al compleet gedigitaliseerd. Dus stel dat zij een nieuwe medewerker door het gehele proces zouden willen helpen, moeten ze eerst het hele proces zelf kennen en weten. Dat is ook waarom wij in huis een intern teamleidertraject hebben ontwikkeld. Iedereen die hier nieuw teamleider wordt, ongeacht enige ervaring op managementniveau of als je bedrijfskunde hebt gestudeerd of iets anders, moet je toch het traject volgen. Dat helpt je ook door de processen van het >ZIEKENHUIS< heen.

**Interviewer**: Is dit een organisatie-breed programma?

**Respondent:** Ja. Dus of je nou teamleider wordt op een klinische afdeling of op een ondersteunde afdeling, of op een poli, of zelfs bij een >AFDELING<-afdeling, dat maakt niet uit. Dan volg je het programma.

**Interviewer**: Zijn er nou eigenlijk ook nog bepaalde activiteiten, personeels-gerelateerde activiteiten, waarvan u zoiets heeft dat is echt niet de taak van de teamleider. Dat ligt bijvoorbeeld bij HR of bij een andere orgaan in het ziekenhuis. Dat is niet iets waar ik mijn teamleider mee wil belasten.

**Respondent:** Dat is een goede vraag. Ik vind sowieso dat stuk waar ik vertelde over de officiële waarschuwing, daar zou een teamleider misschien bij betrokken kunnen zijn, maar dat vind ik echt een aan bedrijfsleider. Dat moet echt een bedrijfsleider doen. Als het gaat om, hebt ook HR-gerelateerde zaken die in een begroting voorkomen of in een business cases. Dat vind ik echt wel zaken voor een bedrijfsleider. Dat wil niet zeggen dat een teamleider daar niet in kan meedenken of mee kan helpen. Want ik weet dat, afhankelijk van de persoon, zijn er best teamleiders die het ongelofelijk interessant vinden om ook eens mee te kijken van 'hoe werkt zo'n begroting' of 'hoe werkt zo'n investeringsaanvraag'. Maar dat moet niet per definitie de eindverantwoordelijkheid zijn van een teamleider. Dat vind ik echt wel de taak van een bedrijfsleider.

**Interviewer**: Zijn er dan ook nog bepaalde manieren hoe u de taken uitgevoerd wil hebben. Of zijn teamleiders daarin vrij om zelf een eigen weg te vinden?

**Respondent:** Ik zou het het leukste vinden als ze zelf hun weg daarin zouden kunnen vinden. Maar in the end komt het erop neer dat het dezelfde procedures zijn die ze daarin volgen. Soms kun je ook niet anders. Onze aanstellingsprocedure is daarin hetzelfde. Dan zou je wel kunnen denken van 'ik wil nu een hele andere vacature gaan maken, samen met HR', dat kan. Zou dan vooral HR op en ga samen kijken naar vacature tekst. Of wil je in de externe media daarin zelf wat doen. Of op social media iets ondernemen. Be my guest en doe me een voorstel, dan is het alleen maar leuk dat mensen daar zelf over nadenken. Maar in the end kom je op dezelfde stromen terug.

**Interviewer**: Zijn er dan bepaalde workflows gebouwd, een soort stappenplan waar de teamleider doorheen kan werken? Of is het vooral de ene medewerker die de andere medewerker uitlegt van 'zo gaat het hier'.

**Respondent:** Ja, dat is een leuke vraag. Ik ben vier jaar geleden hier begonnen als bedrijfsleider. Toen liep ik er tegen aan dat er geen draaiboek aanwezig was waar ik als bedrijfsleider aan moest voldoen. En als ik dan dat draaiboek volg, dan weet ik wat ik moet doen. Helaas is dat draaiboek er niet. Zo is dat er ook niet voor teamleiders. We hebben een kwaliteitshandboek gemaakt die als leidraad van de afdeling dient. Waar je moet voldoen. Dat je zeker weet als je deze stappen hebt gedaan, dat dan je hele kwaliteitsverantwoording en EVT-verantwoording geregeld is. Dat zou je prima uit kunnen splitsen van 'dit doet een bedrijfsleider' en 'dit doet een kwaliteitsmedewerker' en 'dat doet een teamleider'. Daar zou je prima onderscheid tussen kunnen maken.

**Interviewer**: Is dat onderscheid ook gemaakt in het kwaliteitshandboek?

**Respondent:** We zijn momenteel met de uitsplitsing bezig. Hebben we nog niet kunnen volmaken omdat die teamleider er nog niet is. En dat we zeiden, 'ik heb nu een kwaliteitsmedewerker en ik wil niet dat zij nu al verplicht dingen gaat kwijtraken aan iemand die er nog niet is. Dan ligt het weer op mijn bord. Dus daar zijn we nog een beetje zoekende in. Maar dat gaat er zeker komen.

**Interviewer**: In dat handboek staat dan ook wat, wanneer gedaan zou moeten worden?

**Respondent:** Ja, en hoe vaak per jaar en of er een standaard format is waarin zoiets verslagen wordt. Naar mijn mening zou dat voor deze organisatie een groot winstpunt zijn voor deze organisatie als je zoiets uniform voor alle EVRs instelt. Dat is meer mijn mening. Ik zou het bijvoorbeeld ook heel goed vinden als teamleiders uitwisseling hebben over verschillende afdelingen. Omdat er namelijk enorme diversiteit binnen de organisatie is. Dat zal overigens overal het geval zijn, maar je kunt deste meer leren door het uitwisselen van functies of het uitwisselen van mensen binnen bepaalde functies.

**Interviewer**: Is er dan ook nog bepaald gedrag dat u verwacht dat vertoond wordt door teamleiders als ze personeelsactiviteiten uitvoeren?

**Respondent:** Ja, zeker ja. Een teamleider zou zich zeker moeten gedragen als iemand die een voorbeeldfunctie heeft ten aanzien van bepaalde professionalisering die die heeft. Bepaald professioneel gedrag. We hebben bepaalde kernwaarden binnen het ziekenhuis. Daar zou die zich zeker aan moeten identificeren en daarop voor moeten staan ten opzichte van de rest. Dat zal inherent zijn aan dat soort functie. Ik denk dat iedereen die onderdeel uitmaakt van een managementlaag, zich daaraan moet conformeren.

**Interviewer**: Zijn er nog zaken die u zou willen toevoegen aan het interview?

**Respondent:** De afstemming tussen bedrijfsleider, teamleider, en HR is ontzettend belangrijk. Zo moet duidelijk zijn welke taken tot de bedrijfsleider, de teamleider, en HR behoren: een duidelijke taakverdeling. Zo zou een nieuwe medewerker IC aan een opleiding beginnen. Maar nu blijkt dat deze persoon geen opleidingsovereenkomst heeft. We zouden ook wel kunnen spreken van een trilaterale overlegstructuur.

**Interviewer**: Als ik het goed begrijp is het hierin belangrijk om de duidelijke verwachtingen te hebben van iedereen zijn rol hierin?

**Respondent:** Ja, dat klopt.

*Einde transcript*

# Transcript Interview Middle Manager 2

**Duur interview: 27 minuten en 41 seconden**

**Functie: Bedrijfsleider**

**Afdeling(en) onder supervisie: Healthcare**

**Gender: vrouw**

**Werkervaring in functie: n.a.**

**Interviewer**: Dan is mijn eerste vraag eigenlijk: wat zijn volgens u de dagelijkse werkzaamheden van teamleiders?

**Respondent:** De dagelijkse werkzaamheden van teamleiders is dat zij operationeel sturing geven aan een onderdeel van de EVR. In dit geval kan dat een afdeling zijn of een kliniek of een functieafdeling of een specialistische afdeling. Zij sturen de dagelijkse gang, de dagelijkse operationele gang van zaken aan. Dat is eigenlijk hun voornaamste taak. En daarna, nemen zij taken over die de bedrijfsleider delegeert. Dat kunnen leidinggevende taken zijn waarvoor zij het mandaat krijgen, maar dat kunnen ook andere taken zijn. Bijvoorbeeld, het deelnemen aan projectengroepen, ziekenhuis-brede projectgroepen, of kwaliteitsprojecten binnen de afdeling.

**Interviewer**: Heeft u voorbeelden van taken die u dan overdraagt aan teamleiders?

**Respondent:** Jazeker, ik draag een deel van de jaargesprekken over aan de teamleiders. Formeel is het zo dat iedere medewerker recht heeft op een jaargesprek met zijn leidinggevende. Dat is in dit geval de bedrijfsleider. De teamleider geeft ook voor een deel leiding aan de afdeling, maar onder eindverantwoordelijkheid van de bedrijfsleider. Ik schrijf iedere maand nieuwsbrieven, die stuur ik aan mijn medewerkers en aan het begin van het jaar, of ergens daar, staat in de nieuwsbrief wanneer we weer met de jaargesprekken beginnen en daar schrijf ik dan bij dat wij de jaargesprekken verdelen. Ik moet bijna honderd gesprekken voeren, dat is een beetje veel. Ik heb dat in het begin ook gedaan, maar op een gegeven moment waren de teamleiders zo ver dat zij dat ook kunnen doen. Ik zeg dan wel tegen medewerkers 'als je echt een gesprek met mij wil hebben, geef dat dan aan. Dan houden we daar in de planning rekening mee'. Dus zo is het naar beide kanten gewaarborgd, dat werkt heel erg goed. Teamleiders kunnen prima jaargesprekken voeren. Daar zijn ze in getraind. Daar zijn ze ook in geschoold. Anders gaat het namelijk niet. Wij voeren jaargesprekken volgens een bepaald format. Voordat wij met medewerkers in gesprek gaan, dan vragen we altijd nog aan elkaar 'goh, moeten we nog iets met elkaar bespreken, of is er iets dat jullie is opgevallen?'. Ik zie niet alles, en zij zien ook niet alles. Dus op die manier is dat redelijk goed gedekt. Soms weten teamleiders het beter dan ik te benoemen wat er goed gaat en wat er niet goed gaat omdat zij op een andere manier werken. Dat is een voorbeeld van wat ik hen laat doen. Andere dingen die ik hen laat doen, ik ben heel actief in het delegeren van taken omdat ik vind dat teamleiders in een soort, als ze er geschikt voor zijn, bij gebleken geschiktheid, in een soort kweekvijver, een soort biotoop waarin ze kunnen leren hoe ze leiding kunnen geven en hoe ze, ze moeten het ergens gaan leren anders kunnen ze het nooit doen. Dus ik laat het hun dat doen en ik laat hen ook deelnemen aan projecten in mijn naam, met mijn mandaat. Dat gaat heel goed.

**Interviewer**: Aan wat voor projecten zouden teamleiders deelnemen?

**Respondent:** Kwaliteitsprojecten. Zo hebben we de NIAS gehad. Dat is ondergebracht bij een van de teamleiders. We doen dat wel in overleg. We hebben eens in de twee weken MT overleg en daarin bespreken we dingen. Wat een andere teamleider, die heeft een project gedaan die bij mij lag, dat was het ziekenhuis-breed vervangen van ECG-apparatuur. Dit is >NAAM<afdeling, dus hebben we het meeste te maken van ECG’s. Hoe ga je dat nou doen en op welke wijze ga je het opslaan? Moet het digitaal of toch nog steeds analoog. Die vragen zijn er nog wel. Dat heeft de teamleider allemaal gedaan. Die heeft een plan van aanpak gemaakt en uiteindelijk wordt het een implementatieplan. Dus zij heeft ook de gesprekken met de leverancier. Ik zit daar wel bij. Niet altijd, maar meestal als het kan of als er besluiten genomen moeten worden dan lopen die altijd wel via mij. Maar zij informeren mij. Mij teamleiders informeren mij heel goed van alles wat er op de afdeling speelt. Andere voorbeelden van wat ik hen laat doen is, soms bij langdurige verzuimtrajecten, laat ik hen die trajecten doen, waarbij zij mij wel constant blijven informeren en ik zeg 'nu wil ik wel dat Jantje, Pietje, of Klaasje eens een keer in de week komt koffie drinken, want ik wil ze wel blijven zien'. En dat zo eigenlijk wel prima.

**Interviewer**: En dan nemen die teamleider het voortouw in dat proces om diegene uit te nodigen of iets dergelijks.

**Respondent:** Ja, ze onderhouden het contact. Dus ze moeten er wel wat voor doen.

**Interviewer**: Dat zijn sterk personeels-gerelateerde taken die u bij de teamleider neerlegt. Zijn er dan nog meer taken die met personeelsmanagement te maken hebben die een teamleider zou kunnen doen?

**Respondent:** Ja. Werving en selectie. Gesprekken voeren, brieven lezen.

**Interviewer**: Doen ze dat dan alleen, of zit u er dan bij, bij zo'n gesprek.

**Respondent:** Afhankelijk van de kandidaat die komt solliciteren, zijn er gesprekken. Dus stel het is iemand die voor een specialistische afdeling komt, voor de CCU-opleiding, dan zit daar altijd een opleidingscoördinator bij om te beoordelen of iemand ook aan een opleiding mee zou willen gaan doen. Als het een interne sollicitant is die al vaker rondgelopen heeft, en het is meer een formaliteit, dan zitten de teamleider en ik erbij. Als het iemand van buiten is, dan hebben we twee gesprekken: een met leidinggevenden, en een met het team.

**Interviewer**: Ligt er ook nog een soort coachende rol bij een teamleider?

**Respondent:** Ja, de teamleider heeft ook een coachende rol. Bij medewerkers. Bijvoorbeeld bij de jaargesprekken die we houden, dat zit een beoordelingscomponent aan vast. Er wordt beoordeeld 'voldoende' of 'onvoldoende' of 'meer dan voldoende'. Dus of iemand werkt naar verwachting, boven verwachting, of onder verwachting werkt. Dus dat betekent ook dat je het gesprek met medewerkers aan moet gaan over wat je ziet, wat je van hem verwacht, waar die nog kan groeien, of waar hij zichzelf al overtreft. Dan vraag je ook 'waar kunnen we je mee helpen' en dan horen we van de medewerker waar zijn behoefte ligt van die medewerker. Dan gaan we daarmee aan de slag. Dus coachen doen we ook. Soms worden er scholingstrajecten opgesteld met mensen. Daar komt ook de teamleider in. De teamleider coacht ook de leerlingen van de leer-werkplaats.

**Interviewer**: Dat dan toch een aardig actieve personeelsrol die zo'n teamleider heeft.

**Respondent:** Ja, ze zijn er druk mee.

**Interviewer**: En voeren ze daarnaast ook nog medische taken uit?

**Respondent:** Werken aan het bed?

**Interviewer**: Juist.

**Respondent:** Ja, dat doen ze ook nog. Want ik wil dat de teamleiders voor vijftig procent aan het bed staan omdat ik vind dat de teamleiders operationeel het team aansturen. De EVR >AFDELING< heeft een aantal onderdelen, een stuk of vijf, waar ik leiding aan geef, samen met de cardioloog, en ik werk niet mee op de afdeling. Dat gaat ook niet omdat ik vind dat ik meer beleidsmatig aan het werk moet zijn. Dus dat operationele, dat doen de teamleiders. En ze moeten ook weten hoe het gaat, anders zitten we dadelijk alleen nog maar in kantoortjes te vertellen hoe ze het op de werkvloer moeten doen. Dat gaat niet werken. Dus zij werken mee. Op papier staat vijftig procent, maar dat lukt niet altijd. Soms moeten ze echt veel meewerken, en soms is het iets minder. De teamleider van de afdeling werkt soms mee. De teamleider van de polikliniek werkt mee op de katheterisatiekamer. Die staat daar dus ook echt operationele taken uit te voeren.

**Interviewer**: En als we kijken naar die personeel-gerelateerde activiteiten, wat voor beslissingsbevoegdheid zou zo'n teamleider dan hebben? Zou u aan kunnen geven waar de grens zou liggen van 'hierover mag je beslissen, maar als je daarbuiten komt, dan kom je naar mij of iemand die daar het laatste woord over heeft'.

**Respondent:** Ik kan niet echt zwart-wit of heel scherp een grens trekken van dit mag je wel, en dit mag je niet. Ze hebben bijvoorbeeld geen tekenbevoegdheid voor investeringen of grote uitgaven. Die moeten ze echt met mij overleggen. Aanname- en ontslagbeleid dat kunnen ze ook niet zelf beslissen. Dat gaat via mij, maar hebben ze wel een hele sterke stem in. Ze kunnen we beslissen of iemand die zich ziekmeld, of die ziekteverlof vraagt, of ze dat goedkeuren. Daar kunnen ze vrij ver in gaan: dat moeten ze ook kunnen beoordelen. Zo zijn er ook dingen op operationeel gebied. Soms besluit ik iets waar ze het helemaal niet mee eens zijn en dan moeten ze mij overtuigen. En dat doen ze dan ook, en dan pas ik mij aan.

**Interviewer**: Over die tekenbevoegdheid. Als er dus iets aangekocht moet worden, dan komen ze uiteindelijk bij u. Bijvoorbeeld een opleiding of iets dergelijks.

**Respondent:** Juist. Voor opleidingen, die inventariseren we ieder jaar en bekijken we wat iemand wil. Die komt in de opleidingsbegroting welke weer bij HR ingediend wordt.

**Interviewer**: We hebben dan die personeelsactiviteiten van een teamleider in kaart, zijn ook nog activiteiten waarvan u zoiets heeft van 'dat valt buiten de teamleider, daar hoeft de teamleider zich niet mee bezig te houden, maar HR of jou bijvoorbeeld'?

**Respondent:** Ik heb het overstijgende overleg met cardiologen of met andere specialismes. Ook het bedrijfsleidersoverleg doe ik. Maar als ik bijvoorbeeld voor langer weg ben, dan regel ik, dat ik al vaker gedaan heb, of tijdens mijn vakantie dat er een belangrijk punt op de agenda staat van HR of bedrijfsleiders, dan vraag ik of de teamleider bij dat punt aanwezig kan zijn omdat ze daar iets over te zeggen heeft of, namens mij, daar iets moet gaan halen. Dat kan dan ook. Dat gebeurt dan ook. Bedden sluiten, dat vind ik ook nog zo'n verhaal. We blokkeren in principe geen bedden. Daar ben ik fel op tegen.

**Interviewer**: Wat houdt dat precies in?

**Respondent:** Dat kun je geen patiënten opnemen omdat er te weinig personeel is bijvoorbeeld of er teveel zieken zijn of te zware patiënten liggen of omdat het te druk is. Ik vind dat als we een patiënt hebben en we hebben een bed, dan moeten die patiënt in een bed. Er kunnen situaties zijn waarin je geen patiënt kunt opnemen omdat je de zorg niet kunt geven omdat er een paar ernstig zieke patiënten liggen en er te weinig personeel ligt. Dat kan natuurlijk ook in een weekend, of 's avonds, of op een vrijdag dat ik er niet ben. Dan heeft die teamleider het mandaat om die bedden te blokkeren. Maar daar hebben we wel afspreken over gemaakt. We weten van elkaar hoe we daarin staan. Dan mogen zij dat doen.

**Interviewer**: Dat is een aardig vergaande beslissingsbevoegdheid.

**Respondent:** Ja. Dat overleggen ze dan ook met de cardioloog die dienst heeft. Dat is de procedure zoals het gaat, en zo doen zij die dan. Daar hoeven zij mij niet voor te gaan bellen.

**Interviewer**: U zegt het net al, procedures. Zijn er bepaalde manieren waarop u wil dat een teamleider zijn werkzaamheden uitvoert of mag een teamleider dan zelf beslissen hoe hij dit doet?

**Respondent:** Dat mag hij helemaal zelf weten. Dat mag hij echt zelf weten. Ik werk op basis van vertrouwen, en niet op basis van wantrouwen. Ik ga er vanuit, en dat checken we ook en dat blijkt ook uit de praktijk, dat zij doen wat zij moeten doen. Soms moet ik wel eens wat bijsturen of vragen of ik daar antwoord op kan krijgen of 'hoe zit dat, wanneer ga je dat doen?' en dan geven ze daar antwoord op. Dan weet ik dat en dat is prima. Maar ik vind dat zij de vrijheid moeten hebben om hen werk zelf in te delen. Ik let wel op het tempo en of ze het werk afkrijgen en of er omstandigheden zijn waardoor ze er niet aan toe zouden komen. Dat kan natuurlijk ook zo zijn. Maar ik ga er niet naast staan met een stopwatch van 'het is nu tien over tien en nu moet je met die en nu moet je met dat beginnen'.  Dat vind ik niet want zij hebben hun eigen verantwoordelijkheid en die moeten ze ook kunnen dragen en aan mij goed uit kunnen leggen.

**Interviewer**: Is het dan wel eens voorgekomen dat die teamleider zoveel vrijheid ervaart dat hij het dan op een andere manier aanpakt dan u voorstaat?

**Respondent:** Ja, dat kan ja.

**Interviewer**: En heeft u dan zoiets van 'daar ga ik in mee' of is het dan juist..

**Respondent:** Nee, dan ga ik daar in mee. Kijk, als het werk, dan werkt het. Hoe zij het dan maakt niet uit. Als het resultaat er maar is. Hoe zij dat resultaat bereiken, de ene doet het op een andere manier dan de ander. Ik heb twee teamleiders die totaal verschillend zijn van elkaar, in aanpak en in karakter, en ik zie dat zij elkaar aanvullen. Uiteraard, soms zijn daar wel eens irritaties. De ene is tot zes cijfers precies achter de komma en de andere is heel situationeel en lost het gelijk op. Samen komen ze daar goed. Soms komen ze eens in elkaars irritaties. Dan spreken ze dat uit, en is het goed. Daar heb ik eigenlijk nog nooit in hoeven te grijpen. Ik geloof dat we ooit eens een gesprek over gehad hebben, heel erg in het begin. Je moet ook elkaars kwaliteiten kunnen waarderen. Dat doen zij ook. Wij hebben een hele open cultuur. Dat heb ik ook naar de teamleiders en andersom ook. Dus ja, we mogen ook mopperen en ook op elkaar of op de afdeling, als de deur maar dicht is. Zodra we naar buiten komen, moeten alle neuzen dezelfde kant op staan. Dat is wel iets wat ik eis: naar buiten toe verkondigen we dezelfde boodschap. Dan kunnen medewerkers ons niet uit elkaar spelen. Dat wordt wel geprobeerd, maar dat lukt niet.

**Interviewer**: Daar heb je wel een hele sterke overlegband voor nodig?

**Respondent:** Ja, dat klopt.

**Interviewer**: Hoe doen jullie zoiets dan? Hebben jullie veel mailcontact met elkaar, of lopen jullie constant bij elkaar binnen?

**Respondent:** We hebben heel veel informeel contact. We zien elkaar iedere dag. Een keer in de twee weken hebben een formeel overleg: het MT overleg. Verder zien wij elkaar iedere dag en bespreken we wel de dingen die belangrijk zijn. Ik zie iedere teamleider niet iedere dag als ze bijvoorbeeld druk zijn op de werkvloer, of als ik zelf een hele drukke agenda heb. Het kan niet zo zijn dat er dingen lopen waarvan we niet op de hoogte zijn. We informeren elkaar door middel van overdrachten. Stel het is weekend en het is heel erg druk geweest, dan schrijft een van de teamleiders een overdrachtje met daarin de dingen van die dag zodat we allemaal goed geïnformeerd zijn.

**Interviewer**: Als we kijken naar hetgeen dat een teamleider moet weten, dus echt de kennis die een teamleider moet bezitten op het gebied van personeelsmanagement. Wat vind u dan belangrijk dat zo'n teamleider weet.

**Respondent:** Kennis op het gebied van personeelsmanagement vind ik minder belangrijk. Die kennis heb ik namelijk zelf ook niet: deze kennis ga ik halen. HR is er uiteindelijk ook nog. Zij moeten ons kunnen adviseren over de dingen die we niet weten, dan gaan we daarmee aan de slag. Dat is eigenlijk zoals het werkt. Wat vooral belangrijk is: ze moeten goed met mensen om kunnen gaan. En mensen kunnen motiveren, enthousiasmeren, en met weerstanden om kunnen gaan. Dat is eigenlijk heel belangrijk. Wij merken natuurlijk uit medewerkersonderzoeken wat daar naar voren komt en welke knelpunten er zijn. Daar maken we dan een plan voor en daar gaan we achteraan. Dat plan evalueren we dan weer en bepalen we of het resultaat heeft gehad. En zo ja, wat fijn. En zo nee, wat moeten we dan gaan doen? Daar betrekken we ook medewerkers. Alle teamleiders en ik zitten bij elkaars teamoverleggen. We hebben de teamoverleggen in drie verschillende afdelingen gesplitst: poli-functie, >AFDELING<, en >AFDELING< omdat het niet altijd dezelfde onderwerpen zijn waar het over gaat. Daar zitten we alle drie bij.

**Interviewer**: Ik hoorde net iets van onderzoeken. Zijn er nog bepaalde graadmeters om te bepalen wanneer het werk van een teamleider naar behoren is uitgevoerd.

**Respondent:** Het is meer zo, als ik iets van ze zie dit is voldoet niet aan mijn verwachtingen of dit stuk is niet goed geschreven ofzo, dan geef ik daar feedback op.

**Interviewer**: Heeft u bepaalde verwachtingen van teamleiders?

**Respondent:** Ja zeker. Ze moeten overstijgend kunnen denken. Ze moeten een helicopterview kunnen hebben. Ik heb wel eens horen zeggen in de sollicitatie, waar ik toen vroeg: heb je ook een helicopterview? Toen zij iemand tegen mij 'dat weet ik niet, ik heb nog nooit in een helikopter gezeten'. Dan denk ik 'ja leuk antwoord, maar je hebt de vraag niet begrepen: dat gaat 'm niet worden.' Zij moeten verbanden kunnen leggen, en goed kunnen kijken naar oorzaak en gevolg. Bij medewerkers spelen heel vaak dingen die oppervlakkig lijken, maar toch ergens vandaan komen. Ik wil dat ze daarnaar op zoek gaan.

**Interviewer**: Ze moeten de diepte in kunnen gaan?

**Respondent:** Ze moeten de diepte in kunnen gaan. Dat doen ze ook. Zij kunnen mij voeden met dingen die ik niet weet. Andersom kan dat ook. En zo hebben we dat met medewerkers. Als het iemand is waar het heel moeilijk mee gaat worden, dan halen ze mij erbij. Soms hebben we hele problematische periodes met medewerkers. Dan kun je dat niet laten doen door één medewerker. Dan verdelen we het. Die is dan voor jou en als ik iets kan doen, dan hoor ik het. Dan pakken we het gezamenlijk op. Wat heel belangrijk is dat ik de signalen van medewerkers krijg. Als ik van medewerkers signalen krijg van 'ik zie mijn teamleider niet', dan ga ik daar achteraan en meteen mee aan de slag.

**Interviewer**: Het komt dan ook wel eens voor dat signalen de teamleiders passeren en bij u terecht komen?

**Respondent:** Ja, dat komt voor.

**Interviewer**: Als we dan eens kijken naar het gedrag van een teamleider. Dus op de werkvloer. Hoe moet een teamleider zich dan gedragen?

**Respondent:** Hij heeft een voorbeeldfunctie. Een teamleider moet onder een vergrootglas kunnen liggen. Een teamleider moet echt het goede voorbeeld geven. Hij mag niet iets doen wat ook niet geaccepteerd zou worden van medewerkers. Omdat je het voorbeeld geeft. Je hebt een voorbeeldfunctie. Daar moet je goed op letten. Als medewerkers het signaal afgeven 'ik zie mijn teamleider nooit' of 'komt binnen en is weer weg', dan gaan we daarover in gesprek van 'ik hoor die van de medewerkers'. Dit is wat zij zien en wat zij ervaren. Ga daarmee aan slag. Let daarop en werk daaraan. Anders ga je daar problemen mee krijgen. Dat soort dingen. De teamleider is een zorgspecialist en een manager. Hij moet problemen tot op bepaalde hoogte kunnen managen. Als een probleem hier komt, omdat de teamleider het niet heeft kunnen managen, terwijl hij dat wel had kunnen doen, dan is daar een leerpunt. Hij moet het leren. Het is niet erg als je het niet kan. Dat gebeurt ook. Dan ga je daarmee met elkaar in gesprek. En wat wij daarmee gedaan hebben in het begin is dat zij bij heel veel moeilijke gesprekken hebben gezeten om te kijken en om te luisteren hoe ik dat nu deed en wat nou belangrijk was. Wij hebben dan ook echt geleerd om door te vragen.

**Interviewer**: U heeft uw manier van gespreksvoering overgedragen op de teamleiders?

**Respondent:** Wat ik vooral aan hun heb overgedragen is van 'haal een leidinggevende voor ogen die je nooit had willen zijn, waar je zelf last van hebt gehad'. Laat dat het voorbeeld zijn van zo moet je het niet doen. Ik heb zelf het meeste gehad aan een leidinggevende die ik heb gehad van 'nou, zo ga ik het absoluut nooit doen'.

**Interviewer**: Dan werk je daar van af, richting de leidinggevende die je wel zou willen zijn?

**Respondent:** Juist ja. Het blijft altijd dat je soms op basis van de functie meer overredingskracht hebt of meer zeggenschap of dat soms diensten niet opgelost kunnen worden. Situaties als 'ik krijg dat niet opgelost, die wil niet werken'. Dan vragen ze het aan mij of ik het op wil lossen, en dan los ik het op en dat vinden ze heel frustrerend. Dan hebben ze hetzelfde gevraagd en dan kan die medewerker niet. Dan kom ik en dat lukt het wel. Dan vragen ze aan mij 'hoe heb je dit voor elkaar gekregen?' Het is op dat moment gewoon mijn functie die er staat en dat maakt dat die medewerker niet kan weigeren: ga je het zelf oplossen of wil je dat ik het ga oplossen?

**Interviewer**: Het is dan eerder een hiërarchische kwestie?

**Respondent:** Het is eerder een hiërarchische kwestie ja.

**Interviewer**: Ik weet niet of u nog dingen aan dit interview toe wil voegen?

**Respondent:** Ik ben benieuwd naar je onderzoek. Heb je hier iets aan?

**Interviewer**: Het is lastiger om verwachtingen te bepalen omdat u uw teamleiders vrijlaat. U heeft wel een sterke band. Als het wat is, rapporteren jullie aan mij.

**Respondent:** Dat is ook de basis waarop wij werken.

**Interviewer**: Ik ben benieuwd wat voor verwachtingen dat schept bij teamleiders als alles diegene in principe vrij staat om te doen. Ik ben benieuwd wat voor verwachtingen dat schept.

**Respondent:** Het staat ze vrij om te doen op de manier waarop zij willen, als het resultaat er maar is. Dat is heel belangrijk. Dat weten zij ook. Ik verkeer in de veronderstelling, en als dat niet zo is, dan ga je dat horen, dat de teamleiders een goede kans hebben gekregen om zich te ontwikkelen. Ik ben namelijk uit het operationele werk gestapt. Hierdoor moet je als teamleider echt gaan kijken van 'hoe lopen mijn processen' en 'waar kan ik nog efficiëntie uit behalen?' en 'wat kunnen we daaraan verbeteren'. In plaats van kunnen Jantje en Pietje de dienst wel samen doen? Dat wil ik helemaal niet weten. Als er iets is waarom dat niet kan, dan moet dat probleem dat er onder ligt aangepakt worden. Ik wil echt overstijgend werken. Ik kan veelste weinig tijd daaraan besteden door het hoge ziekteverzuim en het moeilijk invullen van vacatures. Als zaken van teamleiders bij mijn komen, kan ik mijn werk niet doen. Dat vinden zij ook heel vervelend. Ik ben benieuwd of mijn beeld overeenkomt met dat van mij. Als het sterk afwijkt wil ik dit graag weten. *Lacht.*

**Interviewer**: *Lacht.*

*Einde transcript*

# Transcript Interview Middle Manager 3

**Duur interview: 23 minuten en 37 seconden**

**Functie: Bedrijfsleider**

**Afdeling(en) onder supervisie: Healthcare**

**Gender: vrouw**

**Werkervaring in functie: n.a.**

**Interviewer:** Dan is mijn eerste vraag: wat zijn de dagelijkse werkzaamheden van een teamleider in uw ogen?

**Respondent:** De dagelijks in de kliniek >AFDELING< is operationele gedeelte, dat is het stuk kijken naar de kwantiteit-kwaliteit verhoudingen op de afdeling gerelateerd aan het type patiënten dat er ligt en dat >TEAMLEIDER< observeert van 'is het in evenwicht?', dat is iets dat >TEAMLEIDER< in de gaten houdt en de dagelijkse werkzaamheden zijn ook de kwaliteitsaspecten. De kwaliteitsaspecten dat betekent of dossiers compleet zijn en compleet worden ingevuld, ook een stukje begeleiding ook die >TEAMLEIDER< oppakt. >TEAMLEIDER< loopt ook in uniform. Dus dat betekent dat >TEAMLEIDER< ook patiëntencontact heeft. Geen eigen patiënten, wel in begeleiding van verpleegkundigen als er tekorten zijn. Dan kunnen ze op >TEAMLEIDER< terugvallen.

**Interviewer:** U noemde net de kwaliteit-kwantiteit verhouding. Wat bedoelt u daar precies mee?

**Respondent:** Daar bedoel ik mee, daar gaat het om de typen patiënten dat er ligt dat varieert met de dag en zijn dan de juiste verpleegkundigen bij de juiste patiënt geplaatst? We doen aan patiënttoewijzing hier. We maken altijd een planning voor de hele week dat je al weet voor de hele week bij welke patiënt je komt staan. Maar, de situatie kan veranderen. >TEAMLEIDER< kijkt wat is geschikt. Je hebt verpleegkundigen met een >AFDELING< opleiding en basisverpleegkundigen. >TEAMLEIDER< is dan ook verantwoordelijk voor de garantie van de kwaliteit van zorg die gegeven wordt.

**Interviewer:** Dat is heel sterk de medische kant van de teamleider. Er zit ook nog een hele bedrijfskundige, managementkant aan een teamleider. Zou u daar iets over kunnen vertellen?

**Respondent:** Samen, wij kijken samen ook naar het stuk van 'hoe wordt er gewerkt, wordt er ook wel efficiënt gewerkt?'. Het jaarplan maak ik, >TEAMLEIDER< kijkt dit jaarplan na. >TEAMLEIDER< kijkt ook of dingen aangevuld moeten worden. Alles wat wij plannen voor het komende jaar of voor het meerjarig beleid eigenlijk, gaan we ook kijken van 'wat voor poppetjes heb je nodig', 'wat voor scholing heb je nodig?'. Daar heeft **﻿**>TEAMLEIDER< ook een rol in. Om te kijken 'wat hebben er voor nodig?'. Gaan we uitbreiden, hebben we extra bedden nodig in verband met de productie die wel of niet toeneemt, gaan we iets heel anders doen? Dat stuk pakt zij ook op.

**Interviewer:** Ik heb eens nagekeken waar een teamleider allemaal mee bezig zou kunnen zijn, naast de zorgtaken, daar komen dan deze onderwerpen of werkzaamheden uit. Als u het lijstje zo ziet, zijn er dan nog bepaalde aspecten waarvan u denkt 'dat zie ik >TEAMLEIDER< toch heel vaak mee bezig zijn?'

**Respondent:** De functioneringsgesprekken of jaargesprekken dan delen we het team in tweeën: ik doe de helft, zij doet de helft. Ik pak de iets complexere jaargesprekken om het zo te zeggen. Werving en selectie doen we samen, introductie voor nieuwe medewerkers dat doet zij voornamelijk. Zorgt ook dat de juiste verpleegkundige aan de juiste medewerker gekoppeld wordt om te zorgen dat de inwerking gewoon goed gaat lopen. Personeelsbestanden houd ik grotendeels bij, maar zij ook. Zij heeft ook inzage daarin, uiteraard. De trainings- en ontwikkelingsbehoeften van medewerkers bespreken we met elkaar. >TEAMLEIDER< geeft mij de inhoud eigenlijk van 'wie moet wat gaan doen'. Want >TEAMLEIDER< signaleert wat hen trainingsbehoefte is. Dat ziet >TEAMLEIDER< op de werkvloer. Informele training, nee weinig. Mentoren, ja grotendeels. Een teamleider heeft ook een mentorrol natuurlijk, naar de teamleden toe. Jaargesprekken hebben we het al over gehad. Ontwikkelingsplannen plannen die ontwikkelen we, die maken we samen en beoordelen we samen. Het hangt er vanaf want we verdelen dat. Disciplineren en corrigeren is een rol, maar het hangt er vanaf op welk niveau het zit.

**Interviewer:** Kunt u aangeven waar het verschil ligt?

**Respondent:** Voor de verpleegkundigen doet >TEAMLEIDER< het grotendeels. Voor de AIOS-en en de artsen komt het bij mij terecht.

**Interviewer:** Is er nog een reden voor deze scheiding waarom deze gemaakt is?

**Respondent:** Ook omdat het soms lastiger is. Verpleegkundigen, >TEAMLEIDER< ziet wat zij operationeel precies doen. Op de werkvloer ziet **﻿**>TEAMLEIDER< wat er gebeurt. Dus als er iets gebeurt waardoor je in moet grijpen of wat dan ook of iemand feedback moet geven, dat is wat duidelijker. Met de artsen weer ander soort processen. Dat heeft ook te maken met de samenwerking met de medisch specialist. Die rol pak ik vaak op. Sowieso ook de gesprekken met de arts-assistenten. Klachtenfunctionaris ook de teamleidersrol. Niet alleen van de klachten die ingediend zijn, maar ook als er een signaalfunctie komt, dat er vanuit patiënten komt, dat je al vanuit patiënten hoort, dat je die patiënt ergens over hoort klagen, dan is de bedoeling dat >TEAMLEIDER< met de patiënt gaat praten om te voorkomen dat er een officiële klacht wordt ingediend.

**Interviewer:** Op organisatieniveau bedoelt u?

**Respondent:** Ja, als te heftig is, dan kom ik erbij. Als de situatie te enorm heftig is, dan pak ik hem over. ... Ja, de absentie valt ook onder haar. Beslissen over salarisschalen, dat ligt bij mij. Maar ik laat mij wel adviseren door >TEAMLEIDER<. Bottom-up communicatie ook. Top-down, dat ligt eraan wat het is. In principe informeer ik >TEAMLEIDER< over alles wat top-down komt en >TEAMLEIDER< geeft dit door aan het team. Aanhoren en reageren op personeel doet **﻿**﻿>TEAMLEIDER< ook. Coördineren doet >TEAMLEIDER<, effectief teamwerk zorgt >TEAMLEIDER< voor. Ja, eigenlijk bijna alle aspecten komen naar voren. De ene wat meer dan de andere, dat hangt ook van de situatie af.

**Interviewer:** En als we dan eens kijken naar de bevoegdheden van >TEAMLEIDER<, waar mag >TEAMLEIDER< dan over beslissen? Wat voor een onderwerpen?

**Respondent:** In grote lijnen. Behalve. In grote lijnen mag >TEAMLEIDER< beslissen. Aanname van personeel. Als ik er niet zou zijn geef ik >TEAMLEIDER< de rol. Als je alle items langs gaat, dan ligt de beslissingsbevoegdheid vrij hoog, maar wel in overleg met mij.

**Interviewer:** Heeft u een idee waardoor deze bevoegdheid van >TEAMLEIDER< zo hoog ligt.

**Respondent:** Ik denk niet dat dat anders is dan voor de andere teamleiders. Uiteindelijk ben ik degene die verantwoordelijk is, die een eindbesluit neemt. Stel dat hier iemand is die niet goed functioneert, dan ben ik degene die het contract beëindigd.

**Interviewer:** U durft wel veel uit handen te geven. Dat zegt dan iets over >TEAMLEIDER< capaciteiten?

**Respondent:** Ja. >TEAMLEIDER< is een hele goede teamleider. >TEAMLEIDER< is al heel lang op de afdeling. >TEAMLEIDER< is echt gegroeid in de rol. Ik zie als ik naar >TEAMLEIDER< kijk, naar de persoon en niet naar de rol als teamleider. Dan zie ik >TEAMLEIDER< ook in een soort kweekvijver voor een volgende functie.

**Interviewer:** Wat zou deze volgende functie dan inhouden?

**Respondent:** De functie van bijvoorbeeld bedrijfsleider. >TEAMLEIDER< zit eigenlijk al een stapje verder, in een kweekvijver, voor als er iemand vrij komt dat >TEAMLEIDER< ook zou moeten kunnen doen. Daarom ook dat ik >TEAMLEIDER< in alle aspecten meenemen: als de begroting gemaakt moet worden. Ik maak de begroting samen met de EVR-voorzitter. Maar ik neem >TEAMLEIDER< in het hele proces mee. Zodat >TEAMLEIDER< op de hoogte is waarom bepaalde besluiten genomen worden.

**Interviewer:** Kunnen we dan ook zeggen dat >TEAMLEIDER< plaatsvervangend bedrijfsleider is als u er niet bent? Of is dit een stap te ver?

**Respondent:** Nee, zo is het hier ook niet ingericht in de organisatie. Op het moment dat ik er niet ben. Bijvoorbeeld als ik op vakantie ben of voor meer als een paar dagen, dan moet ik een collega-bedrijfsleider, die is degene die mijn rol dan waarneemt. En heeft dan ook te maken met rekeningen die betaald moeten worden. Die rol is nog niet toegewezen aan de teamleiders, terwijl >TEAMLEIDER< dat wel zouden moeten kunnen als je ze maar goed informeert.

**Interviewer:** Dan komt het uiteindelijk allemaal op de communicatie neer?

**Respondent:** Ja, klopt.

**Interviewer:** Heeft >TEAMLEIDER< ook nog een financiële bevoegdheid? Dat zij investeringen zou mogen doen. Bijvoorbeeld de aankoop van materialen of het toewijzen van een training aan iemand?

**Respondent:** Nou dat aankopen van materialen daar is een hele procedure voor. Dat is niet zo makkelijk. Investeringen boven een bepaald bedrag moet aangevraagd worden bij de Investeringscommissie. Dat ligt bij ons bedrijfsleiders want wij moeten dat ook echt verdedigen. Kleinere dingen. Stel dat hier iets kleins stuk gaat, een douchestoel ofzo wat onder dat budget valt, mag zij ook voor vervanging zorgen. Omdat zij weet dat wij er een vast aantal moeten hebben, en >TEAMLEIDER< weet ook het moet vervangen op het moment dat het nodig is. Die bevoegdheid heeft >TEAMLEIDER<.

**Interviewer:** Geldt dat dan ook voor investeringen in het personeel?

**Respondent:** Ook dat is weer. We maken een vaste scholingsbegroting jaarlijks. En dan een bepaalde scholingen toegekend, met een bepaald budget wat je krijgt. Dus dat staat eigenlijk wel vast. Zij mag het toewijzen. Op het moment dat we zeggen van nou 'we hebben 5.000 euro voor symposia vrijgemaakt voor de verpleegkundigen', dan mag >TEAMLEIDER< toewijzen welke verpleegkundigen daarvoor in aanmerking komen.

**Interviewer:** Als we dan kijken hoe >TEAMLEIDER< de taken uitvoert. Heeft u dan nog een bepaalde standaard in uw achterhoofd waarvan u zoiets heeft 'als >TEAMLEIDER< het werk zo verzet heeft, dan naar mijn mening naar behoren gedaan.'

**Respondent:** Dat is een hele brede vraag die je stelt. Wat ik verwacht. Als het in evenwicht is. Als de veiligheid van de patiënt gegarandeerd is, de veiligheid van de medewerker. Als aan alle kwaliteitseisen wordt voldaan. Als >TEAMLEIDER< kan zorgen dat bijvoorbeeld alle protocollen op tijd gemaakt zijn. Die hoeft >TEAMLEIDER< niet zelf te maken, maar dat >TEAMLEIDER< degene aanstuurt die daarmee bezig zijn. Dat is dus alle afgesproken processen op de afdeling naar volledige tevredenheid verlopen. Dat geldt dus eigenlijk voor alle processen.

**Interviewer:** Vooral gedoeld op die personeelsactiviteiten die>TEAMLEIDER< uitvoert, daar heb je bepaalde kennis en vaardigheden voor nodig ...

**Respondent:** Ja, juist.

**Interviewer:** ... om deze naar behoren te kunnen uitvoeren. Wat zou >TEAMLEIDER< moeten kunnen en weten?

**Respondent:** Je bedoelt vanuit de opleidingskant, vanuit haar niveau?

**Interviewer:** Ja, bijvoorbeeld.

**Respondent:** >TEAMLEIDER< heeft scholing gevolgd natuurlijk, voor de teamleidersfunctie. >TEAMLEIDER< is ook met een vervolgstudie aan de slag gegaan. Want je moet ook inhoud hebben. Je moet bedrijfsprocessen kunnen, bedrijfseconomisch moet je, en ook het personeelswerk, moet je een stukje geschoold worden. **﻿Dus**>TEAMLEIDER< moet sowieso op de hoogte zijn van de processen en protocollen vanuit de organisatie.

**Interviewer:** En heeft zij dan die opleiding die u net noemde, is dat een bedrijfskunde opleiding of is het een soort master class?

**Respondent:** Nee, ik zou het geen eens een master class noemen. Er is een interne opleiding geweest en daarin zijn alle facetten van het teamleiderschap die zijn allemaal daar besproken, uitgetest en zijn getoetst. Dus daaraan heeft >TEAMLEIDER< voldaan. Daarna heeft >TEAMLEIDER< een vervolgopleiding gedaan 'Middenmanagement-opleiding'. Deze is niet volledig afgerond door >TEAMLEIDER<. Maar >TEAMLEIDER< heeft anderhalf jaar van deze opleiding gedaan. Deze heb je ook weer nodig om je vervolgstappen te kunnen zetten.

**Interviewer:** Kijkende naar taken die >TEAMLEIDER<, zijn er nog bepaalde taken waarvan u zoiets heeft 'dat hoort niet bij >TEAMLEIDER<, maar bijvoorbeeld bij mij of een HR afdeling'?

**Respondent:** Ik zit heel even na te denken hoor. Sowieso werk je nauw samen natuurlijk. Zeker als je kijk naar het stukje personeelsbeleid, werk je samen. Ook met de HR afdelingen. Je werkt samen bijvoorbeeld over het aannamebeleid, et cetera. Je zit in een samenwerkingsverband dus je kijkt samen naar kwaliteitsaspecten op het gebied van kwaliteit en veiligheid. Dan werk je nauw samen met de adviseur. Die betrek je er nauw bij. Datzelfde geldt voor opleidingen. Je zit niet op de stoel van opleidingen. Maar, je zet afdeling Opleidingen in als je een nieuw soort opleiding wil opzetten of wat dan ook. Het is niet zo dat je het werk van de faciliterende afdelingen doet, het is dat je daarmee samenwerk.

**Interviewer:** Je gebruikt de faciliterende afdelingen als een soort verlengstuk om het allemaal mogelijk te maken?

**Respondent:** Ja.

**Interviewer:** Heeft u voor ogen hoe u wil dat >TEAMLEIDER< de taken uitvoert of is >TEAMLEIDER< daar heel vrij in om zelf in te richten?

**Respondent:** Daar heeft >TEAMLEIDER< vrijheid in. >TEAMLEIDER< weet welke taken voor haar zijn weggelegd eigenlijk. Wij hebben sowieso dagelijks contact. Elke ochtend bespreken wij in het kort even wat de prioriteiten op dit moment kunnen zijn. Het kan zijn dat >TEAMLEIDER< komt met verzuim van personeel dan ineens heel veel verzuim is. Dan kan daar de focus op liggen. Dat ik moet zorgen dat voldoende personeel in de avonddienst zit. Maar, het kan ook zo zijn dat, we zijn nu bezig met het verpleegkundig dossier. Dat digitaal gaat worden. Nu heeft >TEAMLEIDER< de focus om te zorgen dat alle verpleegplannen erin gehangen worden. Dat dit >TEAMLEIDER< prioriteit in taken is eigenlijk. Daar krijgt >TEAMLEIDER< wel een vorm van vrijheid in. Ik bedoel ik heb met >TEAMLEIDER<, een mooi voorbeeld, besproken binnen deze tijdskader moet het klaar zijn. En hoe >TEAMLEIDER< dat dan vervolgens inricht moet >TEAMLEIDER< zelf weten. Ik houd een vinger aan de pols door te vragen 'kan je laten zien hoe ver het is, ben je op schema ermee?'. Maar >TEAMLEIDER< mag zelf bepalen hoe de dag ingevuld gaat worden.

**Interviewer:** U zei net '**﻿**>TEAMLEIDER< is zich bewust van alle taken die >TEAMLEIDER< heeft'. Enig idee, als ik >TEAMLEIDER< dan zou zijn, hoe ik dan op de hoogte kan zijn van al mijn taken?

**Respondent:** Door te overleggen en door te communiceren.

**Interviewer:** Er is geen document aanwezig dat stapsgewijs uitleg geeft.

**Respondent:** Nee, nee, nee, nee. >TEAMLEIDER< is van alles op de hoogte dat moet gebeuren. Sowieso deel ik heel veel informatie vanuit de organisatie met >TEAMLEIDER<. Via de mail stuur ik alles aan >TEAMLEIDER< door zodat >TEAMLEIDER< op de hoogte is van alles wat gebeurt en wat prioriteit heeft en wanneer iets opgeleverd moet worden. >TEAMLEIDER< is van alles op de hoogte.

**Interviewer:** Dat zijn het uiteindelijk de zaken die spelen die dan bepalen wat er moet gebeuren en wanneer het moet gebeuren?

**Respondent:** De prioriteiten. Stel, ik zou van mijn fiets af vallen, dan moet zij het even tijdelijk kunnen overnemen. Zij moet dus ook volledig op de hoogte kunnen zijn. Dus ook over gesprekken die ik heb met development medewerkers. Dat is lastig. En ook daar informeer ik haar over en over de inhoud van die gesprekken. Zodat zij ook op de hoogte is en alle dingen kan opvangen.

**Respondent:** Ik denk dat je de teamleidersrol, >TEAMLEIDER< zit nu op de kliniek. We hebben ook nog een polikliniek. Daar zit geen teamleider, daar zit een senior. Maar, zoals morgen ben ik er niet. Dus >TEAMLEIDER< loopt ook lang de polikliniek om te kijken 'jongens lopen de zaken daar?'.

**Interviewer:** Wat maakt naar uw mening het verschil tussen een senior en een teamleider?

**Respondent:** Hiërarchisch. Een senior heeft geen hiërarchische rol en die is ook minder opgeleid daarvoor. Die hoeft geen personeelswerk te doen. Die hoeft alleen maar als een soort aanspreekpunt te zijn, de seniorrol, om te zorgen dat alle lopende processen goed lopen. Of als er nieuwe processen opgestart moeten worden, dan moet met de senior besproken worden hoe dat geregeld moet worden. De rol is heel anders. In de teamleidersrol zit meer vrijheid. Meer de kans om ook innovaties in te zetten.

**Interviewer:** Duidelijk. U bent dus ook voorstander van het gebruikmaken van teamleiders?

**Respondent:**  Ja, ja dat klopt.

**Interviewer:** Zijn er dan ook nog bepaalde gedragingen die u van uw >TEAMLEIDER< verwacht die >TEAMLEIDER< laat zien tijdens het uitvoeren van de personeelstaken.

**Respondent:** Teamleider is een rottige functie eigenlijk. Want je zit als een soort buffertje tussen mij in en het team in. Het lastige dat als de teamleider uit het team kom, is dat het nog moeilijker is. Want je haalt jezelf uit de groep eigenlijk. Want je hebt altijd groepsgedrag. Die rol kan soms ook heel erg moeilijk zijn. Dus ik verwacht ook van **﻿**>TEAMLEIDER< dat >TEAMLEIDER< loyaal is naar allebei de kanten toe. Dat stukje respect verwacht ik zeer zeker van **﻿**>TEAMLEIDER<. De dingen die ik met >TEAMLEIDER< bespreek, dat die niet meteen het team in gaan. En andersom, als een medewerker iets met >TEAMLEIDER< bespreekt, wat ze liever nog niet met mij willen delen, om wat voor reden dan ook. Dat is daarom een beetje een lastige rol.

**Interviewer:** Er wordt een groot beroep gedaan op elkaars integriteit?

**Respondent:** Ja, ja zeer zeker.

**Interviewer:** Heeft u dan ook nog het idee dat >TEAMLEIDER< een voorbeeldfunctie heeft naar het team toe?

**Respondent:** Ik denk het wel. De teamleider op zich die moet ook zorgen dat >TEAMLEIDER< voor de verpleegkundigen het voorbeeld eigenlijk is. Als je wil dat ze zich aan afspraken houden, dan moet jij je ook aan afspraken houden. Als teamleden met >TEAMLEIDER< een afspraak maken, of het nou gaat om vrije dagen of wat dan ook. Dan moet je ook zorgen dat je, als een verpleegkundige aan >TEAMLEIDER< vraagt 'wil je even kijken of ik het weekend vrij kan zijn?', en je kijkt er die dag gewoon helemaal niet naar en je komt er never-nooit meer op terug, dat kan niet. Want je verwacht dat als je aan een verpleegkundige vraagt 'wil je je aan de afspraak houden?' dat het ook gebeurt. Dus je zit ook echt in een soort voorbeeldfunctie en ook in gedrag, maar ook in bejegeningen naar elkaar toe. Daar heb je ook een voorbeeldfunctie in.

**Interviewer:** En als u >TEAMLEIDER< als leider zou omschrijven, is zij dan een hele hiërarchische leider, of staat zij midden in het team en gaat er boven staan waar nodig?

**Respondent:** Dat is een lastige. Dat is namelijk iets wat >TEAMLEIDER< ook als een lastig iets ervaart. Dat is wat haar rol ook lastig maakt. Ik denk dat >TEAMLEIDER< zeker de hiërarchische rol pakt waar het nodig is. Voor de rest laat >TEAMLEIDER< altijd merken dat >TEAMLEIDER< in het team zit. Dit hangt dus van de situatie af.

**Interviewer:** Ik ben door mijn vragen heen. Ik weet of u nog informatie wil toevoegen aan het interview?

**Respondent:** Wat ik best zou willen toevoegen is dat deze organisatie nu deze organisatiestructuur, EVRs. Stel dat straks een hele andere structuur zou komen in het ziekenhuis. Dat kan natuurlijk altijd gebeuren. En dat je zegt van 'we gaan het niet meer met EVRs doen, we gaan weer clustermanagement invoeren'. Een heel ander niveau. Dan denk ik zeker dat de huidige teamleider de oude hoofdenfuncties over kunnen nemen. Zeer zeker. Die zijn dan echt voldoende daarvoor opgeleid en kundig genoeg om dat te kunnen doen. Eigenlijk kun je van een teamleider verwachten van wat vroeger eigenlijk de hoofdenfunctie was. En zeker hoe die hier eigenlijk uitgevoerd wordt.

**Interviewer:** Als u een nieuwe teamleider aan zou moeten nemen, zou u dan meteen dezelfde verantwoordelijkheden overdragen zoals deze nu aan >TEAMLEIDER< heeft overgedragen?

**Respondent:** Niet meteen. Dan zou ik eerst moeten weten 'is iemand er wel geschikt voor'. Misschien stel je dan ook weer een heel ander soort eisen.

**Interviewer:** Dus het komt uiteindelijk op de persoon zelf neer?

**Respondent:** Ja, dat klopt.

*Einde transcript*

# Transcript Interview Middle Manager 4

**Duur interview: 28 minuten en 41 seconden**

**Functie: Hoofd**

**Afdeling(en) onder supervisie: Support**

**Gender: man**

**Werkervaring in functie: n.a.**

**Interviewer:** Oké, laten we dan maar beginnen. Mijn eerste vraag zou dan zijn: wat is de rol van een teamleider hier binnen het ziekenhuis.

**Respondent:** Voor de ondersteunende dienst?

**Interviewer:** Ja.

**Respondent:** De rol van teamleider is er voor zorgen dat de afdeling waar de teamleider verantwoordelijk voor is, gewoon integraal aangestuurd wordt. Dat betekent verantwoordelijkheid voor een stukje HR, verantwoordelijk voor de operaties, verantwoordelijk voor een stukje communicatie met alle stakeholders. Dus ook met zorg. Een stukje financieel. Een deelverantwoordelijkheid ten aanzien van het budget. Dat zijn wat mij betreft taken en verantwoordelijkheden.

**Interviewer:** Om hoeveel teamleiders hebben we het die onder u vallen?

**Respondent:** Onder mij vijf.

**Interviewer:** Dan hebben we ook nog een paar coördinerende personen. Hoeveel zouden dat er ongeveer zijn?

**Respondent:** Dat zijn er zes. Drie afdelingen, per afdeling twee coördinatoren.

**Interviewer:** U zei het net al, er zit een stukje HR in de taken van de teamleider. Wat is stuk HR?

**Respondent:** Dat is heel breed. Eigenlijk alles wat met personeel te maken heeft. Er zit een stukje roostering in, een stukje DRP, ziekmeldingen, gesprekken op het moment dat mensen vaker ziek zijn, jaargesprekken zitten daarin. Dat zijn voor name de belangrijkste aspecten van HR-zijn.

**Interviewer:** Laat u dit volledig aan de teamleider over of houdt u op een of andere manier een vinger aan de pols? Zijn er bijvoorbeeld situatie die zo extreem zijn dat u deze overneemt van de teamleiders?

**Respondent:** Overnemen niet. Dan zou ik er eerder naast gaan zitten dan deze over te nemen. Ik heb wel situatie. Als er bijvoorbeeld een belangrijke klacht is over een medewerker, dat we dan, dat het bijvoorbeeld tot een waarschuwing zou kunnen leiden, dan ben ik daar wel bij. Dan doen we samen het gesprek. Of iemand met frequent verzuim, waar we al vaker mee gesproken is, dan zit ik daar bij. Met name om wat extra dingen te benadrukken van 'goh, het is nu wel echt serieus'. Het escalatiemodel schrijf je aan als je als teamleider al meerdere malen dingen hebt geprobeerd. Dat lukt dan niet, en dan gaan we opschalen. Dan vraagt men of ik er bij wil zitten en of ik een deel van het gesprek zou willen doen.

**Interviewer:** Dat is dan eerder omdat de mogelijke uitkomst, dat dan de risico's groter zijn?

**Respondent:** Ja klopt.

**Interviewer:** Als we dan kijken naar die HR-taken en dan naar de beslissingsbevoegdheid van zo'n teamleider, waarover zou zo'n iemand dan mogen beslissen?

**Respondent:** Wat mij betreft heel veel. Ze mogen sowieso roosters beslissen, ziekteverzuim zijn ze sturend in en mogen ze zelf de gesprekken doen, eerstelijns confl>AFDELING<en dat wel. Bijvoorbeeld niet over het aannemen van personeel. Daar hebben ze een deeltaak in. Een deel-verantwoordelijkheid. Ook over de inschaling van nieuwe medewerkers hebben ze geen zeggenschap over.

**Interviewer:** Dat loop dan via u?

**Respondent:** Ja, en via HR. Daar vinden we wat van. En ook minder over functiebeschrijvingen. Ze gaan minder over functiebeschrijvingen. Zodra een functiebeschrijving aangepast moet worden dan moeten we een stukje input leveren. Het totaalplaatje wordt meer bepaald tussen mij en HR.

**Interviewer:** Heeft de teamleider dan een adviserende rol hierin?

**Respondent:** Ja, klopt.

**Interviewer:** En heeft een teamleider dan ook een financiële bevoegdheid. Om een bijvoorbeeld een aankoop te doen?

**Respondent:** Beperkt. Precieze bedragen ken ik niet. Ik weet zelf dat ik maar voor een bepaald bedrag mag investeren en boven een bepaald bedrag mag moet ik ook de manager, en die mag ook weer tot een bepaald bedrag. Dus daar zit een model. De teamleider mag beperkt, autonoom aanvragen doen. Alles boven 2.500 euro moet dan een handtekening hebben. Er komt dan een inkooporder en die moet een handtekening hebben.

**Interviewer:** Als we dan kijken naar de kennis die teamleider moet hebben. Komt er dan iets in u naar boven waarvan u zoiets heeft 'dit is belangrijk om te weten?'.

**Respondent:** Hoe bedoel je?

**Interviewer:** Bijvoorbeeld ik krijg bijvoorbeeld de taak om de roosters rond te maken, is er dan nog bepaalde kennis die ik moet weten om die taak naar behoren te kunnen doen?

**Respondent:** Je moet je personeelsbestand kennen, alle afspraken die met individuele medewerkers gemaakt zijn. Iedereen moet aan zijn uren komen. Er zijn afspraken. Ook moet je de CAO wel kennen. De rusttijdenwetgeving van 'als je in de avond hebt gewerkt, mag je dan in de ochtend ingeroosterd worden', dat zijn wel dingen die je moet weten.

**Interviewer:** Hoe kan ik daar als teamleider van op de hoogte zijn? Verwacht je dat ik zelf die CAO in duik en dat ik weet wat daar in staat. Of staat zoiets in een documentje op Idoc?

**Respondent:** Sowieso staat op Idoc heel veel. En wat mij betreft, als je het niet weet, je hebt een bila met de HR adviseur. Dat zijn dan vragen die je aan de HR adviseur stelt. Dat je dan zegt van 'ik heb iets gelezen maar ik weet het niet helemaal zeker'. Om het dan even te toetsen bij een HR adviseur. Die heeft natuurlijk een adviserende rol.

**Interviewer:** Alles wat de kennis van teamleiders overstijgt kan dan weer aan de HR adviseur gevraagd worden.

**Respondent:** Ja, ik heb ook regelmatig bila’s. Dat werkt heel goed.

**Interviewer:** Als we dan kijken naar de uitvoer van die taken, wanneer vindt u dan dat deze naar behoren zijn uitgevoerd volgens u?

**Respondent:** Dat is een beetje een gevoelskwestie. Daar heb je geen harde normeringen voor of iemand het goed doet of niet goed doet. Kijk, goed doet wat mij betreft is dat iemand zelfstandig is en als teamleider kan functioneren. Mensen kan aanspreken. Dat we het gewoon hebben over, als opdracht van mij  naar een teamleider dat we een bepaald doel gaan bereiken. Hoe die teamleider tot dat doel komt, maakt mij minder uit. Ik vind het belangrijker dat we samen een doel bepalen. Ik wil die teamleider op dat doel aanspreken en niet over de weg daar naartoe. Ik probeer meer aan te sturen en ik merk dat de ene die makkelijk vind dan de ander. De ene heeft meer vrijheid nodig dan de ander. De andere heeft meer duidelijkheid nodig en wil meer weten over de weg daar naar toe. Bij welke bocht moet ik links af en waar moet ik rechts af? Daar heb je dan wat meer gesprekken mee. En of iemand het wel goed doet, ik vind zelfstandigheid gewoon heel belangrijk. Iemand die gewoon zijn doelen haalt en zonder heel veel moeite daar bij nodig heeft. Dat vind ik prettig. Iemand die wat mee zoekend is, die probeer ik wat meer te coachen of wat meer te begeleiden. Dat vinden we ook prima, dat is ook fijn, maar dan ben je nog wel iets minder ver op het gebied van leidinggevende.

**Interviewer:** Je stuur wel echt aan op autonomie, zelfredzaamheid, zelfsturend?

**Respondent:** In het ideale plaatje wel. Als je echter kijkt naar de teamleider waar we momenteel mee samenwerken is dat nog niet voor iedereen mogelijk. Sommige zitten ook al een tijdje hier en hebben op een bepaalde manier gewerkt of instructies gekregen. Als je andere dingen verwacht, dan is het moeilijk om op een andere manier te gaan werken.

**Interviewer:** Is er dan echt een moment geweest dat het verwachtingspatroon heel er aangepast is. Dat die medewerkers dan moeilijker meekomen?

**Respondent:** Nee, dat gaat geleidelijk. Ik ben wel iemand van de geleidelijke weg en iemand die zeg van 'we deden het zo, nu doen we het helemaal andersom'. Maar iemand die steeds meer, ook vertrouwen geven dat iemand dingen kan en daarom ook zelf die stap gaat maken. En als ik merk dat die stap een beetje te langzaam gaat, toch iedere keer weer een beetje masseren en duwen.

**Interviewer:** Voor dat vertrouwen, hebben jullie dan regelmatig overleg met elkaar? Dat je dan terugkoppeling krijgt over alles wat er speelt binnen de teams?

**Respondent:** We hebben sowieso gemiddeld elke twee weken een bila met de teamleiders. Dan zit ik elke twee weken en hebben we het over een bepaald aantal dingen. Operationeel, financieel, over ziekte, wat losse dingen. Dan kijken we even 'waar staan we, hoe staan bepaalde projecten'. Of de voortgang er een beetje in zit.

**Interviewer:** Als we dan eens kijken naar takenpakket, dat HR-takenpakket van de teamleider, waar kunnen we de grens trekken qua verantwoordelijkheden? Vanaf welk moment heeft u zoiets van 'dit is niet meer de taak van een teamleider, dit is bijvoorbeeld een taak van mij of van HR?

**Respondent:** ... Het aansturen van dat team. Het dagelijks er mee bezig zijn en de mensen in beweging krijgen en houden. Dat is typisch iets van de teamleider. Daarnaast hebben we standaard iets ten aanzien van ziekteverzuim, ten aanzien van jaargesprekken, dat stukje loopbaanbegeleiding hoort daar ook bij, maar dat komt uit de jaargesprekken. Als ik praat over de aanname van personeel, functiebeschrijvingen, dat overstijgt de beslissingsbevoegdheid van een teamleider. Maar ik denk dat teamleiders over het algemeen veel kunnen en mogen.

**Interviewer:** Als we kijken naar de uitvoer van de taken, heeft u dan nog zoiets van 'ik wil dat het zo uitgevoerd wordt' of zijn teamleiders daar juist heel vrij in om zelf een weg in te vinden?

**Respondent:** Ik geef ze redelijk veel vrijheid. Iedereen heeft een andere stijl van leidinggeven. En nogmaals wat ik zeg, ik vind het belangrijk dat er doelen behaald worden, niet hoe het doel behaald wordt. Uiteindelijk wel, niet allemaal mensen uit de wagen vallen. Op een normale manier moeten de doelen behaald worden, zonder dat er allemaal klachten van de medewerkers die bij mij komen van 'goh, die teamleider wil van alles en dat kan allemaal niet'. Het moet dus allemaal binnen de redelijkheid van de wet zeg maar. Ik wil met name toetsen op de uitvoer van de afspraken. De kaders zijn wat mij betreft wel redelijk breed.

**Interviewer:** De doelen die dan gesteld worden, stellen jullie die dan samen op? Of is dit juist iets dat vanuit de manager komt?

**Respondent:** Natuurlijk zijn het allemaal afgeleiden van afgeleiden. Het meerjarenbeleidsplan wat richting geeft aan het gehele ziekenhuis wordt dan vertaald naar een jaarplan voor het afdelingsbeleid, gastvrijheid in dit geval, dat wordt dan weer vertaald naar deelplannen voor de komende jaren voor bijvoorbeeld voor een Foodservice of voor Keuken jaarplan. Daar hebben ze zelf een soort van invloed op omdat ik dan vraag van 'joh, we gaan samen het jaarplan maken. Kom met een voorzet, ik kom met een voorzet gaan we samen zien wat we samen het komend jaar willen bereiken'. Dat is uiteindelijk, het jaarplan dat er ligt voor de afdeling is in samenwerking in met teamleider en met mij. Idealiter heeft de teamleider er ook nog eens met zijn team over gesproken. Maar dat vind ik nog te weinig. Dan draag je hem helemaal tot aan het team toe zeg maar.

**Interviewer:** Enig idee waarom die vertaalslag niet wordt gemaakt richting het team?

**Respondent:** We hebben redelijk wat grote teams hier. Het is moeilijk om die allemaal te spreken te krijgen. Dan zal je daar echt een aparte werkoverleg voor moeten organiseren en werkoverleg is schaars. We zitten dan wel een paar keer per jaar bij elkaar. Want het is een grote groep en moeilijk om iedereen bij het ziekenhuis te krijgen. Mensen werken ook in het weekend of in de avond en moeten daar voor terugkomen. Waar een HR of een financiële afdeling gewoon om vijf uur of om vier uur een vergadering heeft waar iedereen kan komen. Dat is hier wat lastiger. De mensen moeten ook doorwerken. Iedereen hier bij elkaar krijgen, dat wordt lastig. Het is ook nieuw. Er is nooit echt gevraagd aan medewerkers om mee te denken over dit soort plannen. Ik heb dus niet de ervaring dat er in het verleden vaak gevraagd is. Het zou idealiter, ik zou vinden dat ze dat vaker, in betrokken moeten worden. Maar in de praktijk gebeurt dat nog te weinig.

**Interviewer:** Zie je daar een rol voor de teamleiders in?

**Respondent:** Ja, en mezelf om dat nog beter te faciliteren.

**Interviewer:** Het communiceren of echt het bij elkaar krijgen van de groep?

**Respondent:** Beiden. Enerzijds het communiceren en anderzijds, het is een kwestie van halen en brengen. dus je wil graag dat je samen met team iets gaat doen. Dat betekent dat je wat moet geven en wat moet ophalen. Daar zit nu heel veel communicatie in. Maar ook eens faciliteren van 'hoe krijg je je team bij elkaar dat je nog eens zinnige input krijg?'. Of ga je zeggen van 'ik stuur een mailtje en vraag iedereen om input te leveren?'. Dat kan ook. Dat gebeurt nog te weinig. Het is vooral nu van 'goh, we hebben een jaarplan, dit is het'.

**Interviewer:** En als ik, kan ik dan als teamleider me de dan ook heel er vasthouden aan het jaarplan, dat ik weet wanneer ik wat moet doen, qua doen? Of is het jaarplan juist een heel breed iets?

**Respondent:** Het zijn kaders. We proberen ze wel zo SMART mogelijk te maken want dat is de opdracht van de Raad van Bestuur, maar je probeer ook in de tijd richting te geven. Dus je probeer dingen in, teraal 1, tertaal 2, tertaal 3, zeg maar. Dus over een jaar, wanneer gebeurt wat? Grofweg staat dat er wel in. We willen bijvoorbeeld een business case maken in het eerste tertaal. Dus wordt het ook in het eerste tertaal een business case maken. Dus er staat wel een tijd aan gekoppeld. Maar, teamontwikkelingen zijn natuurlijk wel het hele jaar door.

**Interviewer:** Het is niet zo dat ik als teamleider dan ergens een bestand zou kunnen vinden met wanneer ik welke taken zou moeten voeren. Bijvoorbeeld, wanneer de jaargesprekken er weer aankomen of wanneer er echt weer bepaalde gebeurtenissen aan zitten te komen dat ik weet van 'dat kan ik dan verwachten'? Een soort draaiboek als het ware.

**Respondent:** Draaiboek is er niet en ik betwijfel ook of je dat moet willen. Dan ga je heel erg voorschrijven. Dat is het tegenovergestelde van de vrije rol die ik graag wil geven. Bij bijvoorbeeld jaargesprekken weet je bijvoorbeeld dat je 31 december, of zelf in januari nog wel eens, 95 procent van jaargesprekken gedaan moet hebben. Dat is een norm die we als ziekenhuis hebben bepaald, de norm die we ook uitdragen. Dus ik roep in januari al 'weet dat je in december je jaargesprekken moet hebben gedaan'. Zeker die dame die 72 jaargesprekken moet voeren, dat zijn er heel wat. Als je in januari begint, heb je er al twee in de week, voor de rest van het jaar. En ben je op vakantie, dan wordt het alleen maar meer en zit je in december vier per dag te doen. Dat moet je niet willen. Dus ik zeg ook nu gewoon van 'goh, gewoon nu beginnen met het inplannen van die jaargesprekken, anders zit je de tweede helft van het jaar alleen maar gesprekken te voeren en kom je niet meer aan normaal werk toe'.

**Interviewer:** Is het voor de teamleiders goed duidelijk wat er van hen verwacht wordt? Zoals bijvoorbeeld die 95 procent die voor de jaargesprekken gehaald moet worden. Zijn er nog meer van dat soort verschillende eisen waar een teamleider aan moet voldoen?

**Respondent:** Belangrijke onderwerpen zijn die jaargesprekken, je budget. Je moet je aan je budget houden. Een norm hebben we ten aanzien van het ziekteverzuim hebben. Een norm ten aanzien van jaargesprekken die hebben we bepaald als ziekenhuis. Daarnaast heb je op afdelingsniveau afspraken qua ontwikkelingen. Het kan zijn dat bepaalde trainingen door het team gevolgd moeten worden. Of bepaalde veranderingen gedaan moeten worden. Of bepaalde business cases opgelegd moeten worden.

**Interviewer:** Wat voor rol pakt een teamleider daar dan in? Die is dan de kartrekker? Wat is de rol van een teamleider?

**Respondent:** Die rol wordt verschillende opgepakt. Dus dat is een lastige.

**Interviewer:** U mag ook de verschillen uitleggen?

**Respondent:** Je hebt teamleiders die heel zelfstandig de stukken pakken en op basis daarvan aan de slag gaan. Je hebt ook teamleiders die wat meer begeleiding vragen en wat meer voorschrijvend moet zijn. Zij hebben meer een communicerende rol naar het team zeg maar. Dan ben je minder teamleider, dan voer je meer uit. Teamleider is iemand die weet wat die moet doen en gaat daarmee aan de slag.

**Interviewer:** Heb je dan ook nog het idee dat de teamleider bepaalde waardigheden moet bezitten om zo zelfsturend aan het werk te kunnen zijn.

**Respondent:** Ja goed, valt natuurlijk allemaal onder het kopje leidinggevende vaardigheden. Je hebt communicatie nodig, je hebt overtuigingskracht nodig, je hebt een soort politiek gevoel, of een soort gevoel van 'wat speelt er nu binnen de organisatie en hoe krijg ik nou mijn mensen daarin mee?', een soort gevoel. Mensenkennis heb je nodig. Teamleiders hebben hele diverse medewerkers in hun team. Hoe kan ik de mensen nu aanspreken dat ze het ook gaan doen, zonder dat ik alleen maar politieagentje moet gaan spelen en een lijstje moet gaan maken om af te vinken. Dat je mensen kunt overtuigen om bepaalde dingen te gaan doen en bepaalde veranderingen uit te voeren.

**Interviewer:** Als je dan gedragsmatig naar zo'n teamleider kijken, wat voor gedrag verwacht je dan dat door zo'n persoon wordt uitgestraald?

**Respondent:** Ik vind het belangrijk dat de teamleider een soort voorbeeldrol heeft. Iedereen kijkt naar een teamleider en meet daar zijn eigen prestaties een beetje aan af van 'goh op het moment dat die teamleider onzichtbaar is of vaak ziek, dan hebben medewerkers vaker zoiets van 'oké, dat is blijkbaar niet zo belangrijk, dat kunnen wij ook'. Dan heb je wel een belangrijke voorbeeldrol. Dat je zichtbaar bent. Dat je die kartrekkersrol vervult. Dat je de boel enthousiasmeert. Dat zijn wel de belangrijkste. Met name om je team mee te krijgen. Laat gewoon zien wat je doet en wat je ook op papier hebt geschreven. Dus houd je aan je eigen afspraken.

**Interviewer:** Zo'n teamleider, heeft die naast het managen van personeel, voert die nog een ondersteunende taak uit? Bijvoorbeeld een teamleider Keuken, kookt die dan bij wijze van mee of is het puur iemand die constant aan het managen is?

**Respondent:** Het mooie is, de teams die we hebben, dat zijn hele operationele teams. Teamleider Keuken bijvoorbeeld, die kookt gewoon mee. Dat is een redelijk klein team. Dat is een te klein team om iemand die fulltime op kantoor zit. Je hebt geen 36 uur kantoortijd voor zo'n iemand. Ik denk dat hij, naast het bestellen de HR taken, voor de helft meekookt. Foodservice is een grote club, in vergelijking met de Keuken-club, die redelijk klein is, met veel meer taken. Als je bijvoorbeeld kijkt naar rooster, HR, en gesprekken, dat vraagt veel meer tijd. Dus dat is iemand die wel de hele tijd op een kantoorfunctie heeft of in ieder geval niet operationeel meedraait. Alleen in geval van calamiteiten eigenlijk van 'goh, er is echt helemaal niemand, er is paniek'. Dan een jasje aan en meedoen. Dat is bij >AFDELING< ook. Dan heb je >AFDELING<. Daar hebben we zelfs een halve FTE omdat we ervaarden van 'één FTE is te veel'. Dat kan prima in een paar halve dagen. Dat lukt nu ook, dus die hoeft eigenlijk niet mee te draaien. Dan heb je nog bij DIV, dat is digitalisering, dat is een kleiner team, die zit dan compleet in het team. Die draait ook mee met projecten en scant ook.

**Interviewer:** Dus puur afhankelijk van teamgrootte?

**Respondent:** Ja, teamgrootte is belangrijk en de zelfstandigheid.

**Interviewer:** Heeft u het idee dat het voor een teamleider duidelijk is wat van hem of haar verwacht wordt? Door u eventueel een manager of nog hoger?

**Respondent:** Die illusie heb ik wel. We spreken met elkaar doelen af en als het niet duidelijk is hoor ik graag wat er nier duidelijk is. Dan leg ik dat graag uit. Wat mij betreft is het wel duidelijk welke kan we op gaan.

**Interviewer:** U heeft dus ook het idee als het niet duidelijk is voor teamleiders wat er verwacht wordt, dat er dan zelf gezocht wordt of dat er dan contact opgenomen wordt met jou?

**Respondent:** Eerder het laatste. Met name het laatste. Met name het opzoeken is minder. Eerder van 'hoe moet ik nu verder, hoe gaan we dit nu aanvliegen'. Dan is opzicht het doel wel duidelijk waar we graag heen willen. Dan ziet de teamleider in dit geval niet de route daar naar toe, zit even vast, en gaan we dan samen kijken hoe we dan weer uit die modder kunnen komen. Weer richting dat doel.

**Interviewer:** Enig idee hoe het dan komt dat een teamleider die route moeilijker of helemaal niet ziet? Is het een gebrek aan kennis en vaardigheden?

**Respondent:** Ja. Het is nieuw. Het is anders. Maar dan heeft de teamleider misschien net niet de kennis en vaardigheden om daarmee om te gaan, met weerstand om te gaan, of bepaalde verantwoordlijkheden voor elkaar te krijgen.

**Interviewer:** Is jouw rol dan uiteindelijk om dan te instrueren van 'zo zou ik het aanpakken', of stuur je ze op cursus? Hoe reageer jij op zo'n vraag?

**Respondent:** Specifiek een cursus voor een casus niet. Moeten mensen opgeleid worden bijvoorbeeld worden. De huidige teamleiders hebben vorig jaar allemaal een teamleidertraject gedaan hier in het ziekenhuis. Daardoor zijn ze geëquipeerd om weer aan de slag te gaan. Opleiden dus wat minder, maar gaan we meer samen kijken. Ik probeer het eerst bij de teamleider zelf neer te leggen van 'hoe zou je het doen?'. Op basis van die uitkomst dan sturen van 'goh, misschien kan dat ook anders' of 'goh, helemaal goed'. Zoveel mogelijk in overleg te doen en die ideeën bij de teamleider te laten ontstaan. En als het niet lukt dan probeer ik daarbij te helpen. Maar eerst iemand laten nadenken van 'wat zou de oplossing kunnen zijn?'. Als het niet slim is, dan hebben we het daar over.

**Interviewer:** Die teamleidertraining was een goede zet? Een goede basis gelegd?

**Respondent:** Ja, zeker. Daar moeten ze vooral mee doorgaan.

**Interviewer:** Ik heb in principe alles wel wat ik nodig heb. Ik weet niet of er nog bepaalde punten zijn die u toe zou willen voegen?

**Respondent:** Ik denk dat het heel duidelijk is. Je hebt de teamleider. Die zit in een soort spagaatfunctie. Tussen een klein team of een groot team en allerlei opdrachten die van boven komen. En daarmee is het meteen een hele lastige functie. Dan merk je dat sommige al jaren op zo'n functie zitten en het moeilijk vinden om daar verandering in aan te brengen. Over het algemeen denk ik dat ze best weten wat er van ze verwacht wordt en wat men wel mag en wat men niet mag. Als je een tijdje rondloopt dat heb je vanzelf wel een idee van 'dit zijn mijn kaders, dit zijn mijn grenzen ten aanzien van alle HR dingen'. Ik denk dat het wel duidelijk is voor ze wat ze moeten en wat ze mogen.

**Interviewer:** Dat komt dan door alle document die beschikbaar zijn of het contact tussen u en teamleider?

**Respondent:** Een combinatie en jarenlange ervaring die meegedragen wordt. Die jaargesprekken heb ik niet bedacht. Die standaard is al jaren hetzelfde. Men weet de normen, men weet de deadlines. Men weet ook wanneer men ziekteverzuimgesprekken moet houden. Dat zijn vaste afspraken die we hebben. Die zijn bekend. Althans, daar ga ik van uit. Ik moet ook zeggen, de jaarplannen die elk jaar weer ontstaan. Dat is mijn taak om het daar over te hebben. Dat is elk jaar weer anders.

**Interviewer:** Zie je nog verbeteringen hoe we de verwachtingen nog scherper zouden kunnen stellen voor een teamleider.

**Respondent:** Ik denk dat het altijd beter kan. We hebben het hier over communicatie over meerdere lagen. Ik denk wel dat de laag beter betrokken kan worden. Er zit een hoop kennis en kunde bij die laag en denk dat die wel beter betrokken kan worden. Dat merk ik bij mezelf, maar dat merk ik ook breder. Dat hoor ik van andere hoofden ook. Soms moet je meer vanuit je teamleiders informatie halen. Daar zit een hoop ervaring en kennis.

*Einde transcript*

# Transcript Interview Middle Manager 5

**Duur interview: 61 minuten en 04 seconden**

**Functie: Bedrijfsleider**

**Afdeling(en) onder supervisie: Healthcare**

**Gender: vrouw**

**Werkervaring in functie: n.a.**

**Interviewer:** Wat zie jij als de dagelijkse werkzaamheden van de teamleider?

**Respondent:** Voor is een teamleider een soort aanstuur. Dat betekent personele planning. Dat is een belangrijke taak. Het beoordelen van medewerkers. Het coachen van medewerkers vind ik een hele belangrijke taak en je hebt ook verantwoordelijkheid voor de kwaliteit en tevredenheid. Dat zijn voor mij de grootste, of nou, zoals ik het voor mij zie.

**Interviewer:** Dus dat is zowel een zorgpet, als een HR-pet als ik het goed begrijp.

**Respondent:** Ja. Ja. Want personeelsmanagement vind ik echt belangrijk omdat zeg maar zij staan relatief dicht het team zeg maar. Zij zien ze ook wel veel. En ja dat is wel de positie waar je vanuit echt goede coaching kunt aanvoelen. Dat kan namelijk niet, ik ben daarin een wassen neus voor medewerkers.

**Interviewer:** Maak jij ook nog gebruik van seniors?

**Respondent:** Die hadden wij wel zeg maar binnen de EVR. Ik heb even wel, ben wel terughoudend geweest en heb toen afscheid genomen van die functie. En toen ben ik even terughoudend geweest om nieuwe seniors te werven omdat ik, bij ons, de EVR-structuur, ik sta nog redelijk dicht bij de werkvloer. Maar met mij en teamleiders en seniors, ik vind het dan te gelaagd worden. Ik ga nu twee EVRen doen. Ik vind dat nu iets om wellicht voor de komende periode te gaan heroverwegen omdat ik uiteindelijk toch iets meer afstand ga krijgen van de teams. Maar een senior heeft aparte taken zeg maar.

**Interviewer:** En als jij straks twee EVRen gaat leiden, wat voor rol zie jij dan voor die teamleider voor je? Gaat er iets veranderen?

**Respondent:** Deels. Zeg maar he. Wat ik wel doe is eigenlijk nog iets steviger positioneren, maar ik heb mijn teamleiders als echt in de lead gezet op de afdelingen en op het primaire proces. Dus dat heb ik eigenlijk al gedaan. En vandaar heb ik ook ruimte om twee EVRen aan te sturen. Dat zal nog steviger doen waarbij ik met name mijn teamleiders zeg 'kies van joh'. Ik geloof er namelijk niet zo in dat je alle taken bij een persoon moet beleggen want uiteindelijk, je kunt in je eentje heel hard werken, maar als je de groep niet mee krijgt dan bereik je niets. Ik vind veel meer dat we ook, ook echt zeg maar taken en aandachtsgebieden bij professionals neer moeten leggen.

**Interviewer:** Dus nog lager in de organisatie?

**Respondent:** Ja. Je ziet ook zeg maar vanuit studies, waar worden mensen ongelukkig van? Als je geen regelruimte hebt en geen invloed op iets hebt. En als je een teamleider alles laat doen in zijn/haar eentje, ja dan wordt je team daar niet perse gelukkiger en beter van, de teamleider zelf gewoon vet overbelast. En als je dat doortrekt naar de lijn gebeurt bij mij eigenlijk hetzelfde. Als je bij mij alles neer zou leggen om in mijn eentje te fixen. Dus wat nu met name aan het doen zijn is zorgen dat juist taken beter belegd worden in de organisatie, bij de professionals, maar dat ze er wel tijd voor krijgen. Dat kan nu omdat ik twee EVRen aan ga sturen. Het is geen bezuiniging, dus we hebben daar ook iets meer budgettaire ruimte voor. En dat is dus een slag die we willen maken om echt die HBO-verpleegkundigen ook echt daadwerkelijk de tijd en ruimte te geven om een project op te pakken en naast gewoon de direct patiëntenzorg met succes te gaan regisseren. Dat is wat ik wil, wat zeg maar mijn visie is op hoe je een EVR moet aansturen zeg maar. En je ziet zeg maar in al die thuiszorgorganisaties dat ze die leidinggevende helemaal wegsaneren. Ik dat dat binnen een complexe ziekenhuisorganisatie niet te doen is. Maar ik geloof wel dat je kritisch moet zijn op hoeveel leidinggevenden je inzet en wat mensen zelf moeten doen.

**Interviewer:** Juist. Heb je voorbeelden van taken die je graag bij zo'n Hbo’er neer zou willen leggen?

**Respondent:** Ik denk, het hele protocolbeheer. Dat is ook verder denken. Niet alleen het ontwikkelen van een protocol maar ook zorgtaken. Dat is iets wat die Hbo’er heel goed kan en sterker nog, ik kan zo'n zorgpad helemaal niet bedenken en denk ook dat een teamleider soms ook te veel afstand heeft om echt goed zo'n proces, zo'n zorgpad, in te zetten. Want het is denk ik erg inhoudelijk vaak ligt dat bij verpleegkundigen. We zijn nu bijvoorbeeld verpleegkundigen aan het opleiden om hun collega's te scholen in acute verloskunde. Dat vind ik echt iets van teamleden. Maar dan moeten ze ook echt wel willen van 'ga hier planmatig mee om' en ga niet zomaar even een traingkje geven, zeg het echt planmatig goed op. Ik denk met scholing, het medisch convenant, dat dat echt iets is dat niet een teamleider hoeft te doen maar bij het teamlid kan liggen. Die het gewoon goed kan oppakken, die snapt met welke apparatuur die werkt en ook snapt welk onderwijs van toegevoegde waarde is. DIM-commissie. Er moet iemand van de leiding in participeren, maar ik denk dat sommige fouten die gemaakt worden ook op de werkvloer opgelost kunnen worden. De best oplossingen komen ook van de werkvloer. Die komen echt niet van mij of van een teamleider. Dat zijn taken. Efficiëntie, klanttevredenheid, patiënttevredenheid, het doen van enquêtes en ook een goede analyse erop zetten en maatregelen erop zetten. Dat is zeker iets wat teamleden kunnen doen. Eigenlijk heel veel.

**Interviewer:** Juist. Dus het takenpakket van de teamleider die gaat meer, die lager neergelegd worden, of stukjes ervan als ik dat goed begrijp?

**Respondent:** Ja. Stukjes daarvan gaan lager neergelegd worden ja.

**Interviewer:** Heb jij op die moment nog concrete verwachtingen van je teamleiders?

**Respondent:** Ja, ik vind wel, als kijk op dit moment naar de manier waarop mijn teamleiders functioneren, dan vind ik dat ze die slag, zeg maar he, van 'challenge nou je teamleden om zelf dingen te gaan doen' die moeten ze nog wel maken. Ik vind ook dat ze nog procesmatiger moeten gaan denken. Denk in procenten en ga wel checken, check veel meer hoe dingen geregeld zijn. Dan dat je alleen maar aan het werk gaat en dingen maar snel gaat inregelen. Je kan dan wel denken dat je je medisch convenant helemaal super hebt geregeld, maar ik nu bij wijze van spreken het LMS hopen dan zeg ik 'he >TEAMLEIDER<', nog niet alles is afgetekend. Dus zo goed als dat jij zegt dat het is, dat is het nog niet. Dat geeft niets, maar wat heb je er nu voor nodig om het wel goed te laten verlopen? En wat ik merk is dat zeker mijn teamleiders, dat zijn helemaal geen slechte teamleiders, maar zijn wel heel er als solist werkt hij/zij en ik denk dat hij/zij nog wel moet nadenken over 'hoe zorg ik dat ik daar een niet te kwetsbare positie in krijg?'. Wie betrek ik bij mijn besluitvorming? Wie betrek ik bij mijn beoordelingen? Wie betrek bij mijn planning? Ik denk dat zij nog wel in een hele hiërarchische manier van denken zitten. Dan denk ik, ik geloof dat in 2017, hiërarchisch leiderschap past niet echt meer in deze tijd.

**Interviewer:** Juist. Dus echt samen als ik het goed begrijp?

**Respondent:** Ja. Ik zie het heel erg als een voetbalspel. Er moet iemand de coach zijn en iemand moet soms besluiten nemen en een knoopje doorhakken, maar je ben niet de traditionele leidinggevende neer die het voor het zeggen heeft.

**Interviewer:** Heb jij het idee dat je, om als teamleider goed te kunnen functioneren, nog bepaalde dingen moet kunnen?

**Respondent:** Ja. Ik denk dat het belangrijk dat een teamleider echt iets van HR af weet. Dat die dus ook echt weet van 'goh, zo zit het met personeelsmanagement'. Hoe coach je mensen, wat zijn je kaders, wat zijn dingen die wel kan zeggen, wat zijn dingen die je absoluut niet omdat ze een heel slecht effect hebben. Dat vind ik echt wel iets waarin teamleiders goed in opgeleid moeten worden. Ook hoe je beoordelingen doet. Wat zijn de do's en de dont's daarin? Daar kan je echt wel missertjes in maken als je iemand beoordeeld van horen zeggen, nou dat kan echt wel hele nare gevolgen hebben voor teamleden waar ook heel lang en naar effect in zit. Dus dat vind ik dat teamleiders daarin echt goed in opgeleid moeten worden want dat kan nog iets meer zeg maar. Ik vind ook in gedrag, ik heb mijn teamleiders naar zo'n cursus 'stoppen met leidinggeven'. Hoe zorg je dat je mensen nu meekrijgt zonder dat je de leidinggevende uithangt zeg maar. Dat vind ik wel echt heel erg belangrijk dat mensen dat ze dat leren. Ik denk dat je procesdenken en projectmatig kunnen werken, dat vind ik ook heel erg belangrijk.

**Interviewer:** Is het voor jou ook duidelijk waar zeg maar de scheidslijn ligt? Dus waar teamleiders wel over verantwoordelijk zijn en waar teamleiders geen verantwoordelijkheid over hebben?

**Respondent:** Ik denk dat in de huidige situatie met een EVR, die is niet altijd even duidelijk in onze organisatie. Je hebt toch echt wel situaties waarin dik 90, want dat zit er in mijn EVR, 90 tot 100 man. Nou, dat is te klein om mensen te kennen, maar te groot om echt iets op te bouwen met je teamleden. Dat maakt verlof- en ziekteverzuim en beleid verschuiven toch wel wat, dat teamleden soms bij mij komen en soms naar de teamleider gaan. Dat vind ik onplezierig, maar dat gebeurt toch. Het overkomt me nog wel, ondanks dat ik er heel kritisch in ben, maak ik daar zelf soms fouten in. Dat ik toch te ver in dat gesprek ga en dat ik dan later zie 'daar had ik me toch echt niet mee moeten bemoeien'.

**Interviewer:** Juist. Die had lager neergelegd moeten worden?

**Respondent:** Ja. Ja. Ik vind opzicht is hij aardig duidelijk als het gaat om, ik ben echt iemand die voor het beleid is, die de koers uit moet zetten hier op de afdeling, die moet checken of processen goed ingeregeld zijn, die ook echt een verantwoordelijkheid heeft bij de vakgroep. Ik ben budgethouder, ik maar begrotingen, investeringsaanvragen doe ik, ik kijk ook hoe we het doen. Maar ik vind, soms schuurt hij ook wel dicht op elkaar. Ik hoop straks met twee EVRen iets minder gaat zijn. Dat het qua verantwoordelijkheden duidelijk gaat worden.

**Interviewer:** Heb je het idee dat dat realistisch is?

**Respondent:** Nou het is gewoon, het is spannend. Ik denk het wel, anders was ik er niet aan begonnen. Maar is wel een proces dat je met elkaar, wat een spannend proces is waar ik ook coaches bij gevraagd heb zeg maar.

**Interviewer:** Maar hoe ga je dat straks aanpakken bijvoorbeeld als je straks twee EVRen gaat leiden, dan zal je verwachting van de teamleider ook gaan veranderen, hoe ga je zoiets aanvliegen?

**Respondent:** Ik heb sowieso in de nieuwe EVR dat ik maar even 100 dagen om mee heen moet gaan kijken. Dus ik zal eerst gewoon even met name de teamleiders laten zeggen wat ze denken, en dat echt volgen. Maar wat ik nu zeg maar met al op >AFDELING< doe is dat we opnieuw ons organogram hebben opgesteld met elkaar een aantal sessie hebben gedaan van 'wat doe jij, wat doe ik en hoe gaan we het met elkaar vormgeven?'. Op welke momenten is dat belangrijk voor het team dat ik echt zichtbaar ben? Dat kwam nog wel eens uit het medewerkerstevredenheidsonderzoek dat teamleden nog wel eens dingen van mij willen zien. Bijvoorbeeld er is een besluit genomen dat ik de nieuwsbrieven weer ga schrijven, maar ik ook wat meer gevoel krijg bij wat ik denk en vind en doe. Maar daar gaan we dus echt met elkaar over in gesprek van goh 'wie doet nu wat?'. Wat zijn onze rollen? Daar hebben we nu sessies over gehouden. Hoe gaan we vergaderingen indelen? Voorheen was het ook mogelijk om heel veel infomeel met elkaar af te stemmen, zonder dat je het formaliseert. En dat lijkt prettig, maar nu denk ik wel 'zo prettig was dat helemaal niet'.

**Interviewer:** Het is voor jou storend?

**Respondent:** Ik kan later heel slecht op afspraken terug komen. Hebben we dat nu wel of niet afgesproken? En ik merk dat daar soms wel spanningen over zijn ontstaan. Dat ik heb gezegd joh 'dat praatje pot en dat sparren dat gaan we gewoon lekker tussendoor doen en onze werkoverleggen gaan we echt de afspraken goed vastleggen'. Want dat is toch zeker als ik twee EVRen doe, kunnen we het ons niet permitteren om even snel iets met elkaar af te spreken.

**Interviewer:** Je vraagt dus echt van je teamleider om alle administratie goed op orde te houden als ik het goed begrijp?

**Respondent:** Ja. En daar help ik hun mee. Maar dat is ook mezelf zeg maar. En met de >AFDELING< ga ik hetzelfde doen. Dus ik pak het organogram. Waar ben ik zichtbaar, wat doe ik, wat doen jullie en ook 'wat moeten jullie gaan delegeren?'. Het kan niet zo zijn dat zij aan het eind van de rit overbelast zijn en met een burn-out thuis zitten.

**Interviewer:** Merk jij trouwen als bedrijfsleider nog verschillen tussen jou teamleiders? Misschien binnen jou EVR, maar misschien ook in huis?

**Respondent:** Zeker. De ene teamleider binnen dit ziekenhuis heeft veel minder verantwoordelijkheid als dan dat ze dat bij mij hebben. Dat is natuurlijk ook 'hoe zit je als bedrijfsleider in elkaar?'. Waar leg je de accenten op? Ik probeer de EVR op grote lijnen te sturen en voor mij is echt de focus 'daar gaan we het op orde brengen', dat vind ik echt belangrijk en een andere teamleider zit daar soms gewoon veel minder boven op.

**Interviewer:** Hebben jullie dat daar als bedrijfsleider onderling ook wel eens over?

**Respondent:** Ja, wel eens. Ik merk dat dat echt wel verschilt, dat we echt wel van mening verschillen.

**Interviewer:** Juist. Ben jij van mening dat er meer gelijkheid getrokken moet worden tussen teamleiders?

**Respondent:** Dat vind ik een lastige vraag want ik zie gewoon verschillende profielen bedrijfsleider zijn we eindelijk. Ik ook echt de implementatie van EVRen, zijn er bedrijfsleiders die gewoon doorgeklommen zijn, ook ooit een assessment hebben gehad. Die eigenlijk teamleider waren, hoofd zijn geworden en toen ineens bedrijfsleider. Dat vind ik niet perse verkeerd zeg maar, maar dat maakt wel, als je hier ook nog eens honderd jaar werkt en nog nooit ergens anders hebt gewerkt, dan zit je er anders in. Ik zit hier net twee jaar. Dit is mijn derde organisatie en dan zie je verschillende manieren en heb je een denkwijze die met name bij jou past.

**Interviewer:** De verschillen hoeven dus niet eens verkeerd te zijn in dat opzicht?

**Respondent:** Ik zou wel oppassen om daar direct een oordeel aan te geven. Er zijn ook bedrijfsleiders die er voor gekozen om helemaal geen teamleider te hebben omdat zij zeggen 'ik doe het allemaal zelf'. Is dat fout? Dat weet ik niet of dat fout is.

**Interviewer:** Duidelijk. Je zegt 'verschillen hoeven helemaal niet verkeerd te zijn in dat opzicht'.

**Respondent:** Ik durf dat niet zo hard te stellen.

**Interviewer:** Nee, precies. Maar als we de functieomschrijving van teamleider kijken, die wordt dan wel weer organisatiebreed getrokken. Moet die naar jou idee meer op maat gemaakt worden of is de fubes goed op dit moment?

**Respondent:** Kun je de vraag nog een keer herhalen?

**Interviewer:** Er zijn verschillen tussen teamleider. Dat hoeft niet goed of fout te zijn.

**Respondent:** Ja.

**Interviewer:** Maar we gebruiken wel dezelfde fubes voor de teamleiders. Dezelfde functiebeschrijving. Wat vind jij daar van?

**Respondent:** Ja. Phoe, dat vind ik lastig. Even kijken hoor. Je zou daarin kunnen differentiëren natuurlijk. Dat kan. Phoe, lastige vraag hoor als ik heel eerlijk ben.

**Interviewer:** Wat maakt hem lastig?

**Respondent:** In principe, hij is redelijk algemeen geschreven. Dus ik denk in grote lijn dan mensen er zeker wel aan voldoen. Maar de invulling verschilt wel heel erg per EVR, denk ik he? Maar kan je dat in een fubes ook wel weer aanpassen. Ik weet niet of dat zo makkelijk kan.

**Interviewer:** Nee precies.

**Respondent:** Ga in de organisatie daar dan heel veel discussie over hebben? Dan moet je ook weer in honorering gaan verschillen. Mensen willen wel aanpassing op hun fubes als je minder hoeft te doen. Maar als honorering meer of minder wil doen, daar zullen minder mensen om te springen staan. Ja.

**Interviewer:** Dat is een lastig onderwerp, absoluut. Ik kan me namelijk goed voorstellen dat jij als teamleider een andere teamleider spreek die in dezelfde FWG zit, maar veel minder doet, dat dat wrijving kan veroorzaken. Heb jij dat wel eens ervaren?

**Respondent:** Niet echt wrijving. Het is meer dat ze onderling wel eens gesprekken hebben van 'ik veel meer doen dat als jij dat doe'. Daar hebben ze dan de gesprekken met elkaar over. Of 'ik heb het veel beter geregeld dan jou'. Dat ligt aan de bedrijfsleider. Dat kan je nooit helemaal voorkomen. Dus ja, ik weet nou niet of je daar heel veel tijd en energie in zou moeten steken.

**Interviewer:** Het is dus iets vooral iets wat bij de teamleiders ligt om aan te geven van 'goh, ik hoor dit, zou ik dit ook mogen'?

**Respondent:** Ja, dat denk ik.

**Interviewer:** Dus echt zelf een poging doen om de functie zelf iets meer in te kleden?

**Respondent:** Ja.

**Interviewer:** Ik had ook als laatste vraag, zeker gezien de tijd, heb jij nog een bepaald toekomstbeeld van de teamleider. Hoe bijvoorbeeld er over nu en een paar jaar uit zal zien?

**Respondent:** Ja. Wat mij betreft iemand die nog echt meer het proces volgt van 'hoe doen we het op onze afdeling?'. Teamleden coacht en dat jij je als teamleider uiteindelijk ook bereid bent jezelf boos te maken. Terwijl ik ook denk dat het niet helemaal kan in deze complexe organisatie. Dat er toch wel een heleboel dingen over blijven. Idealiter, denk ik, is het team heel self supportive is, dat ze echt dingen zelf regelen. En dat het team zelf met initiatieven komt. Dat het team zelf kritisch is als dingen niet goed lopen. Dat ze echt dingen zelf oppakken. Dat is wat idealiter. Ik denk dat het een utopie is, maar hoe meer mensen samen als team kunnen oppakken en kunnen realiseren, hoe meer successen ze met zichzelf kunnen vieren, hoe gelukkiger je wordt in je werk. Wat uiteindelijk wil iedereen toch invloed hebben in zijn eigen werk. Het is goed als je niet alleen gecoacht wordt door je teamleider, maar ook echt door, juist, de professionele organisatie waarin je hoort van collega van 'dit moet je anders doen'. Dat is wel een utopie, maar ik denk wel dat het echt, naar mijn mening, een streven moet zijn dat je als teamleider jezelf zoveel mogelijk overbodig maakt.

**Interviewer:** Juist, dus als de teamleider niet meer nodig is dan is goed werk geleverd in dat opzicht?

**Respondent:** Dat denk ik ja.

**Interviewer:** Moet een teamleider naar jou mening ook nog een meewerkende functie hebben? Dus echt zorg leveren?

**Respondent:** Vind ik wel belangrijk. Vind ik wel echt belangrijk dat je dat kan. Want anders heb je en een bedrijfsleider die niet meewerkt, een teamleider die niet meewerkt. Ik vind wel dat je in ieder geval mee kan werken, zeg maar he. Dat dat mogelijk is dat je dat doet en dat je dat af en toe doet. Anders denk ik echt dat je niet echt goed kunt coördineren.

**Interviewer:** Het is dus ook belangrijk dat teamleiders ook competent blijven in dat opzicht? Dus ook hun vakgebied blijven bijhouden? Dus ieder moment kunnen bijspringen als het ware?

**Respondent:** Ja, want anders ga je namelijk, echt een soort luchtballon om je heen. Ik kan het ook al niet. Ik zeg altijd 'nou een beetje feedback mag geven, dat kan ik maar verder houd het op'. En doe ik ook overigens waar het moet, dat vind ik ook helemaal geen probleem. Maar dan ga je echt een waterhoofd in je management creëren. Wie kan nog echt coachen en wie kan dan nog echt zeggen tegen teamlid 'je functioneert niet naar behoren'. Dat kan je namelijk echt niet goed zeggen als het werk echt niet goed kent.

**Interviewer:** Duidelijk, ik denk dat we het hierbij kunnen laten. Ik weet niet of er zelf nog dingen zijn die jij toe zou willen voegen?

**Respondent:** Nee, ik heb denk ik wel gezegd wat ik wilde zeggen. Ik kijk nog even naar de vragen. Welke werkzaamheden vallen er buiten jou teamleiders en welke financiële verantwoordelijkheid hebben jou teamleiders?

**Interviewer:** Juist.

**Respondent:** Nou financieel denk ik, ik stel de kaders. En ik zeg ook, de vrije FTE-ruimte mogen ze zelf opvullen. Maar ik vind het wel fijn om erover te sparren hoe ze dat gaan doen. Maar ik heb met >TEAMLEIDER< wel eens een discussie gehad dat hij/zij zei 'ik heb een halve verpleegkundeformatie over, en ik wil er een hele ad-interim assistent voor inzetten'. Nou, dan vind ik wel dat we een goed gesprek moeten hebben samen van 'waarom en wat levert je dat op, waarom zou je dat moeten doen?'. Dat gesprek moeten we wel hebben, maar dan is het ook aan hem/haar als hij/zij zegt 'dit helpt echt het operationele proces'. En we hebben alle vragen afgedicht, dan gaan we dat ook echt doen. Dat is wel aan hem/haar om dat zo in te regelen. En voor mij is het om kritisch te zijn op de begroting. Want stel je voor, er komt weer een begrotingsproces aan, we willen altijd meer dan dat we krijgen. We hebben het hier helemaal niet slecht, maar we willen altijd meer dan dat we krijgen. Dan moet ik zeggen tegen >TEAMLEIDER<, bij wijze van, we gaan er een half FTE'tje van af halen en hij/zij moet mij dan echt challengen. En daar wil ik het in een pittig gesprek met elkaar over hebben. Uiteindelijk heb ik daar de eindverantwoordelijkheid in en stel ik de kaders waarbinnen de teamleider binnen moet blijven. Er is een budget voor uitjes. Als er meer nodig is dan kan de teamleider dat vragen, maar hij/zij heeft wel budgettaire ruimte om dat geld uit te geven.

**Interviewer:** Hebben jouw teamleider nog meer financiële verantwoordelijkheid, als ik bijvoorbeeld denk aan het doen van kleinere aankopen voor het personeel? Denk bijvoorbeeld een bloementje.

**Respondent:** Daar heeft hij/zij volledige vrijheid in. In de kaders die wij stellen. Dus als we zeggen 'een zieke moet altijd een bloementje krijgen'. En ik krijg te horen 'hij/zij strooit met bloementjes, die krijgt iedereen iedere dag', dan gaat het niet goed. Daar hebben we gewoon wat kaders voor afgesproken 'wanneer doe je dat nu wel en wanneer doe je dat nu niet'? Laatst hadden we teamvergadering en toen duurde het wat langer en toen hadden ze honger. Toen heeft de teamleider wat brood en een soepje besteld. Dat zie ik dat. Daar hoef je voor mij echt niet voor te overleggen. Als je meer doet moet je echt aan de bel trekken. Maar ik wil daar niet elke keer over hoeven te overleggen. Anders heb je als teamleider ook niet meer te zeggen.

**Interviewer:** Nee, precies. Merk je dat dat wel eens lastig is, dat zoiets niet zwart-wit is?

**Respondent:** Nee. Dat heb ik eigenlijk nooit gehad. Wat ik wel altijd zeg 'je deelt geen eigen geld uit'. Het is niet mijn geld, het is geen geld van het ziekenhuis, het is geld van de maatschappij. Ik vind dat je daar zuinig en zinnig mee om moet gaan. Ik vind dat je het goed moet investeren. Ik heb hun ook nog nooit dingen zien doen waarvan ik denk 'ik vind dit totaal krankzinnig, dit kun je niet doen, dit had je niet mogen doen'. Dat heb ik nog nooit gehad. Kennelijk voelen wij met elkaar goed genoeg aan waar de grens ligt.

*Einde transcript.*

# Transcript Interview Middle Manager 6

**Duur interview: 46 minuten en 24 seconden**

**Functie: Hoofd**

**Afdeling(en) onder supervisie: Support**

**Gender: man**

**Werkervaring in functie: n.a.**

**Interviewer:** Oke, laten we beginnen. Hoeveel teamleiders heb jij momenteel onder je?

**Respondent:** Haal die die s maar, dus 1 teamleider en dat is >TEAMLEIDER<. Dus 1.

**Interviewer:** Als we naar de werkzaamheden van >TEAMLEIDER< kijken, wat moet ik mij daar dan bij voorstellen, waar houdt >TEAMLEIDER< zich dagelijks mee bezig?

**Respondent:** >TEAMLEIDER< houdt zich bezig met het leiden van het team van de Helpdesk. Dus >TEAMLEIDER< geeft sturing. >TEAMLEIDER< functie was eerst eigenlijk coördinator. >TEAMLEIDER< regelde gewoon wie wat deed. Nu is >TEAMLEIDER< sinds anderhalf jaar teamleider. Wat de functie is, je zei het net al een beetje, personeelsmanagement. Dus >TEAMLEIDER< doet de jaargesprekken, daar is >TEAMLEIDER< verantwoordelijk voor. >TEAMLEIDER< stuurt de mensen aan. En ik, en wij, verwachten ook van >TEAMLEIDER< dat >TEAMLEIDER< de Helpdesk op een hoog niveau brengt in Itilprocessen. Ik weet nog je dat kent?

**Interviewer:** Nee, dat zegt mij niets.

**Respondent:** Itilprocessen is echt gericht op de IT. Dat is gewoon, als je itel-cursus doet, dan krijg je, incident management, change management. Hoe kun je het incidentmanagementproces, waarin verschillende processen worden beschreven van 'zo kun je het inrichten'. Heel veel organisaties gebruiken dat ook en wij verwachten van >TEAMLEIDER< dat >TEAMLEIDER< ook in de sturing richting de Helpdesk ook meeneemt.

**Interviewer:** Is >TEAMLEIDER< dan ook eindverantwoordelijk voor dat itilproces.

**Respondent:** Ja, >TEAMLEIDER< heeft een incidentmanagement-rol zoals dat heet. Die heeft >TEAMLEIDER< ook. Maar dat heeft >TEAMLEIDER< niet alleen voor de rol van de helpdesk, waar >TEAMLEIDER< sturing aan geeft, dat heeft >TEAMLEIDER< ook als rol binnen heel >AFDELING<.

**Interviewer:** Dus als ik het goed begrijp is >TEAMLEIDER< zowel een >AFDELING<-persoon, maar ook een personeelspersoon met personeelstaken als onderdeel van de functie?

**Respondent:** Ja, dat klopt.

**Interviewer:** Is >TEAMLEIDER< ook meewerkend op afdeling? Meedraaiend in het team?

**Respondent:** Grappig. Ik heb vorige week met >TEAMLEIDER< gezeten omtrent de functiebeschrijving van >TEAMLEIDER<. Ik vond in de praktijk dat >TEAMLEIDER< gewoon heel veel uitvoerend werk deed. Ik zei 'ik wil daar eigenlijk een maximum aan hebben'. In de functieomschrijving staat dat >TEAMLEIDER< in tijden van pieken meer dan 25 procent uitvoerend mag zijn. Heb je dat gezien?

**Interviewer:** Ja, dat heb ik gezien.

**Respondent:** En toen zei ik 'ik wil daar een maximaal van maken'. Ik vind ook, voor de dingen die we neer willen zetten, 25 procent nog te veel. Dus max 25 procent. Daar was >TEAMLEIDER< het mee eens. >TEAMLEIDER< zei dat >TEAMLEIDER< geen 25 procent wil, >TEAMLEIDER< wil meer op 10 zitten.

**Interviewer:** Zou dit dan inhouden dat andere teamleden dan meer zouden moeten doen en taken van >TEAMLEIDER< over moeten nemen om dat percentage naar beneden te brengen?

**Respondent:** Dat zou kunnen. Wat >TEAMLEIDER< doet, moet nu iemand anders doen. Maar ik, zoals ik het zie, dat het ook zonder >TEAMLEIDER< moet kunnen.

**Interviewer:** Straks heeft >TEAMLEIDER< meer tijd over. Waar wil je dat die tijd naartoe gaat?

**Respondent:** Wat ik voor wil is een stukje incidentmanagementproces. >TEAMLEIDER< heeft een aantal. Kijk, in het incidentmanagementproces komen een aantal informatiestromen. Een stukje telefonie, mensen bellen. Mensen kloppen in. Mensen komen aan de balie. Om daar te kijken van 'hoe kunnen we dit nu efficiënter indelen?'. Een stukje automatisering. Een stukje 'hoe dring je de calls terug?'. Daar wil ik eigenlijk dat >TEAMLEIDER< zich op focust.

**Interviewer:** Meer overstijgend als ik het goed begrijp?

**Respondent:** Juist.

**Interviewer:** Als we eens kijken naar de personeelsrol die >TEAMLEIDER< heeft. Je noemde net al jaargesprekken. Zijn er nog andere onderwerpen waar >TEAMLEIDER< zich mee bezig houdt op het gebied van mensenmanagement?

**Respondent:** Ik weet niet of planningen daar toe behoren?

**Interviewer:** Ik kan je wel een beetje op weg helpen.

**Respondent:** >TEAMLEIDER< doet namelijk best wel veel. Ziektemeldingen en zo.

**Interviewer:** Kijk, dit zijn voorbeelden van onderwerpen waar een teamleider zich mee bezig zou kunnen houden. We zouden hem door kunnen gaan en dan bekijken wat >TEAMLEIDER< zowaar doet?

**Respondent:** Werving doet **﻿**>TEAMLEIDER< zeker. Selectie doet >TEAMLEIDER<. Introductie...

**Interviewer:** Dus als er een nieuwe medewerker aangenomen wordt, is >TEAMLEIDER< dan degene die die persoon gaat inwerken en wegwijs gaat maken?

**Respondent:** Gedeeltelijk. >TEAMLEIDER< geeft wel een intro, maar iemand anders werkt hem in. Bijhouden van personeelsbestanden.

**Interviewer:** Muteert >TEAMLEIDER<?

**Respondent:** Ja, >TEAMLEIDER< zet het in het systeem, maar ik accordeer het. Bijvoorbeeld >NAAM< is als laatste binnen gekomen. Dat heb ik wel geïnitieerd en toen kreeg >TEAMLEIDER< het binnen en verwerkte >TEAMLEIDER< het. En dan moet ik er nog een laatste klap op geven. Dus ja, dat doet >TEAMLEIDER< zeker. Ook als er iets gewijzigd moet worden. Plannen van trainingsdoeleinden doet >TEAMLEIDER< zeker.

**Interviewer:** Wat doet >TEAMLEIDER< dan precies? Gaat >TEAMLEIDER< met medewerkers in gesprek van 'goh, wat heb je nodig qua training'?

**Respondent:** Ik was bezig met een opleidingsplan voor beide afdelingen. Dus ik heb tegen >TEAMLEIDER< gezegd 'ik wel weten van jou waar de behoefte ligt'. Dat heeft >TEAMLEIDER< ook besproken in het team en dat inzichtelijk gemaakt voor mij. En >TEAMLEIDER< beslist. Een goed voorbeeld voor jou. >TEAMLEIDER< was er niet. >TEAMLEIDER< was een week ziek ofzo. Toen had ik een gesprek met een medewerker over een cursus. Ik had toevallig opleidingsdagen over, die kopen we in. Toen heb ik met die medewerker afgesproken van 'ik denk dat het goed is als jij dat gaat doen'. Toen kwam >TEAMLEIDER< terug en zei 'nee, ik denk dat we niet die persoon'. Dat het oneerlijk was. En >TEAMLEIDER< beslist dan over de uiteindelijke.

**Interviewer:** Uiteindelijk is die persoon dus niet op cursus gegaan?

**Respondent:** Nee, iemand anders.

**Interviewer:** Dat zegt wel iets over hoe jij met >TEAMLEIDER< input en adviezen omgaat. Je neemt het wel degelijk in overweging?

**Respondent:** Ik vind dat >TEAMLEIDER< verantwoordelijk is voor z’n team. Zo probeer ik hem ook te sturen. Ik zit ook niet constant op zijn huid. >TEAMLEIDER< is verantwoordelijk en als >TEAMLEIDER< zijn verantwoordelijkheid neemt, en dat doet >TEAMLEIDER<, dan doet >TEAMLEIDER< goed. En ik vind dat >TEAMLEIDER< het goed doet. En als ik daarin iets zie van 'dat gaat verkeerd', of >TEAMLEIDER< neemt de verkeerde beslissing, dan zeg ik daar ook iets van en moet ik daar ook iets van vinden. Maar weet je, voor 99 procent gaat het goed. Dus ja. Geven van formele training. Ik denk het niet.

**Interviewer:** Dat besteed je dan uit?

**Respondent:** Ja. Geven van informele training, coachen en ondersteunen. Je bedoelt iemand aansturen?

**Interviewer:** Bijvoorbeeld, misschien als iemand het niet snapt. Of even kort instrueren van 'zo kun je het ook doen'.

**Respondent:** Dat doet >TEAMLEIDER< zeker. Mentoren, dat doet >TEAMLEIDER<. Functioneringsgesprekken, dat doet >TEAMLEIDER<.

**Interviewer:** Verdelen die nog onder of doet >TEAMLEIDER< de functioneringsgesprekken voor alle Helpdesk medewerkers?

**Respondent:** Ja.

**Interviewer:** Hoeveel zijn dat er ongeveer?

**Respondent:** Dat zijn er vijf.

**Interviewer:** Te overzien?

**Respondent:** Ja zeker.

**Interviewer:** Doe je verder nog iets met de uitkomsten uit de functioneringsgesprekken?

**Respondent:** Ja zeker. Ik zie ze sowieso in. Ik moet er akkoord op geven. Als ik zie 'er gaat iets mis', dan zeg ik daar iets van. Akkoord geven op ontwikkelingsplannen. Dat is een beetje opleidingsplannen?

**Interviewer:** Ja.

**Respondent:** Dat heb ik. Ik zit hier pas sinds augustus. Toen was het opleidingsplan al een beetje bestempeld. Dus dat voortraject heb ik niet echt meegemaakt. Maar hoe zou ik er in zitten? Dan zou >TEAMLEIDER< er zeker een beslissende rol in hebben. Dan geeft >TEAMLEIDER< akkoord op wat wij met zijn allen dan bedenken. Ik geef akkoord op het uiteindelijke plan. Dat doe ik, maar zijn input verwacht ik wel. Dus akkoord geven vanuit zijn team, zeker. Ja, disiplineren en corrigeren, dat doet >TEAMLEIDER< zeker. Afhandelen van tuchtprocedures. Tja. Klachtprocedures dat ken ik wel.

**Interviewer:** Maar mocht het nou eens voorkomen dat een medewerker, dat de veiligheid van systemen in gevaar zou komen, zou jij dat dan bij >TEAMLEIDER< neerleggen, of zou je het eerder zelf, als hoofd, oppakken? Of ligt dat aan de zwaarte van het incident?

**Respondent:** Dat ligt aan het zwaarte van het incident. Het is ook een stukje Helpdesk. Dus dan is het >TEAMLEIDER<. Maar als het een stukje overstijgend is, dus het raakvlak heeft, zal ik het zijn.

**Interviewer:** Dat klink vrij vergaand dat >TEAMLEIDER< daarin meegenomen wordt?

**Respondent:** Vind je?

**Interviewer:** Het klinkt als een vrij grote verantwoordelijkheid?

**Respondent:** Ja, >TEAMLEIDER< heeft ook de rol van incidentmanagement en daarvoor ook vanuit die rol, moet >TEAMLEIDER< op dat niveau kunnen acteren. En >TEAMLEIDER< kan zelf overstijgen. Als >TEAMLEIDER< vind dat bij >COLLEGA< een incident ligt dat gigantisch impact kan hebben, kan >TEAMLEIDER< zelfs tegen >COLLEGA< zeggen '>COLLEGA<, ik neem die, die, die mensen mee en ga de aankomende uren'. Dat mag >TEAMLEIDER<. Maar in de praktijk gebeurt dat niet zo vaak, maar het zou wel kunnen.

**Interviewer:** Enig idee hoe >TEAMLEIDER< het vindt om die autoriteit eventueel te kunnen gebruiken.

**Respondent:** Ik denk dat gewoon bij systeem- en netwerkbeheer hoort. Ik weet niet hoe >TEAMLEIDER< dat overstijgende vindt, omdat we dat nooit in de praktijk meemaken, dus nee geen idee wat >TEAMLEIDER< daar van vindt. Voor hoe ik >TEAMLEIDER< ken, denk ik dat >TEAMLEIDER< daar wel zijn weg in kan vinden. Het is nooit gebeurt dus vind ik lastig om te bepalen. Absentiemanagement, dat doet >TEAMLEIDER< voor het eigen team. Waardering geven, dat doet >TEAMLEIDER< ook.

**Interviewer:** Enig idee hoe >TEAMLEIDER< dat aanpakt?

**Respondent:** Dan heb ik het alleen over wat >TEAMLEIDER< zegt. >TEAMLEIDER< benoemt het als er iets goed.

**Interviewer:** Dat is een manier van waardering geven.

**Respondent:** Ja. Zo ken ik hem. Dat doet >TEAMLEIDER< richting mij en ook richting anderen. Dat doet >TEAMLEIDER< niet soms, maar echt regelmatig. Beslissen over salarisschalen, dat ben ik eigenlijk. >TEAMLEIDER< kan er wel een inbreng over hebben, maar de uiteindelijke klap geef ik. Uiteindelijk ben ik budgettair verantwoordelijk voor de twee budgets, de twee kostenplaatsen. Daar kan ik overstijgend in zijn. Als >TEAMLEIDER< zoiets van heeft 'daar ben ik het niet mee eens', maar ik ben het er wel mee eens, dan kan zeggen dat het toch gaat gebeuren. Ik neem zijn input wel mee, maar ik ben beslissingsbevoegd. Bijvoorbeeld over >NAAM<. HR zei van 'er is nog nooit een klap op gegeven in de functiewaardering, zit >NAAM< in salarisschaal 40 normaal. Maar omdat de functiewaardering nog niet afgerond is vind ik dat we die persoon in 35 moeten doen. >TEAMLEIDER< zei van '40'. Maar weet je, ik moet een beslissing doen. Dan gaan we toch 40 doen. Maar als blijkt dat we uiteindelijk toch naar 35 gaan. Dus dan doet >TEAMLEIDER< het eigenlijk niet hè?

**Interviewer:** Nee.

**Respondent:** Bottom-up communicatie wat moet ik mij daar bij voorstellen?

**Interviewer:** Als we bijvoorbeeld dingen die in het Helpdesk-team spelen, is >TEAMLEIDER< dan de persoon die die informatie naar jou brengt? Of moeten medewerkers >TEAMLEIDER< direct naar jou toe komen en >TEAMLEIDER< overstijgen? Hoe zie je dat?

**Respondent:** Helpdeskmedewerkers gaven aan >TEAMLEIDER< feedback of terugkoppeling. >TEAMLEIDER< rapporteert dat dan weer door naar mij.

**Interviewer:** Hoe vindt dat plaats? Hebben jullie veel overleg samen of veel mailcontact?

**Respondent:** Toevallig hebben we net. Het was eerst niet zo zichtbaar. Wat we nu doen is een evening call bij de Helpdesk. Het is eigenlijk begonnen bij de Systeem en Helpdesk. Daar ben ik gestart met de morning call. Dat is een stand-up. Weet je wat dat is?

**Interviewer:** Nee, dat ken ik niet.

**Respondent:** Dan ga je om negen uur staan met het team en ga je één minuut per persoon vertellen wat je die dag gaat doen. >COLLEGA< doet dat nu ook. >TEAMLEIDER< heeft besloten de evening call te doen. Morning call evening call. Zij kunnen het niet doen om acht uur want dan krijgen ze de meeste belletjes. Dus dat is heel moeilijk in het proces. Maar vijf uur, ze werken allemaal door tot half zes, dan doet >TEAMLEIDER< de evening call.

**Interviewer:** Dus wat heb ik gedaan?

**Respondent:** Ja, wat heb ik gedaan en wat ga ik morgen doen.

**Interviewer:** Wat >TEAMLEIDER< dan opvangt wat er dan speelt gaat dan eventueel weer door naar jou.

**Respondent:** Ja, het is de bedoeling dat negen uur in de ochtend >TEAMLEIDER< bij mijn morning call staat. Soms kan dat niet, maar het is wel de bedoelding. Bij escallatie kan het helaas niet. Het is wel de bedoeling dat dat iedere dag gebeurt.

**Interviewer:** Nog nooit van gehoord dat dat op zo'n manier kan.

**Respondent:** Zo doen we dat. *﻿Lacht*﻿. Top-down.

**Interviewer:** Dat is dan de andere kant op. Van jou naar >TEAMLEIDER< om vervolgens weer naar het team te gaan.

**Respondent:** Ja, dat gebeurt ook.

**Interviewer:** Is het dan zo dat datgene wat >TEAMLEIDER< de morning calls meekrijgt, dat gaat dan weer naar de Helpdesk?

**Respondent:** Ook in mijn bila's. Ik heb iedere week een bila met >TEAMLEIDER<. Daarin wordt ook gesproken over alles wat besloten is. Ook vanuit mijn hoger management. Ook dat bespreek ik met **﻿**﻿>TEAMLEIDER<. Dat gaat dan weer door naar het team. Aanhoren van suggesties en reageren op suggesties team. Coördineren van het werk van teams. Ja dat doet >TEAMLEIDER<. Onderhouden van effectie teamwerk. Ja, een van de speerpunten die we genomen hebben is dat de effectiviteit omhoog moet. Dat houdt in dat we beter moeten kijken, dat is dan weer onderdeel van het medewerkertevredenheidsonderzoek.

**Interviewer:** Waar druk je efficiency in uit?

**Respondent:** De hoeveelheid werk. Stel dat we 36 uur werk hebben tien applicaties voorbereiden voor de omgeving. En dat straks door die tien applicaties 24 uur staat. Dus hoe kunnen we nou zo goed mogelijk inrichten? Dus eigenlijk meer werk in minder tijd. Dat doen we bijvoorbeeld door vroegtijdig documenteren, dingen automatiseren, en bijvoorbeeld zoals we net zeiden, het incidentmanagementproces dat terugdringen van telefoontjes, hoe doen we dat? Daar zijn we nu dus steeds mee bezig. De self-service portal zijn we mee bezig. Dat mensen zelf een incident inkloppen in plaats van gaan bellen. Dan zijn er minder mensen aan de telefoon, dan hoeven minder mensen het op te gaan lossen. Maar, daar is >TEAMLEIDER< dan verantwoordelijk voor. Voor zijn team is >TEAMLEIDER< daar verantwoordelijk voor. Ik ben dan wel eindverantwoordelijk. Maar ik stuur hem aan dat >TEAMLEIDER< dat waarmaakt. Personeel consulteren. Daar bedoel je gewoon mee dat **﻿**>TEAMLEIDER< naar het team loopt voor advies?

**Interviewer:** Ja. Mocht >TEAMLEIDER< advies uitbrengen richting jou dat >TEAMLEIDER< nog even langs het teamleden loopt voor wat extra input of een blik op de zaak.

**Respondent:** Ja, dat doet >TEAMLEIDER< zeker. Gezondheid en veiligheid. ...

**Interviewer:** Heb je een idee dat hierin een rol voor >TEAMLEIDER< ligt?

**Respondent:** Van >TEAMLEIDER< verwacht ik dat als een medewerker ziek is dat >TEAMLEIDER< het personeel belt en op de hoogte houdt hoe het met deze persoon gaat. Veiligheid ... In het werk >TEAMLEIDER< is het wel zo dat als er iets onveiligs gebreurt dat >TEAMLEIDER< dat oplost. Ik heb niet echt een voorbeeld. Maar ik kan me wel voorstellen dat als er iets wordt neergezet en iemand loopt er constant tegen aan, kan vallen ofzo.

**Interviewer:** Of snoertjes of iets dergelijks of objecten.

**Respondent:** Daar is >TEAMLEIDER< verantwoordelijk voor. Verbeteren van werklevens. ... Ik denk nog niet zo hebben gekeken naar het team. Maar ik denk wel dat het een hele goede is. We kijken natuurlijk wel naar werkplezier.

**Interviewer:** Gaan kijken of hebben gekeken?

**Respondent:** Gaan kijken.

**Interviewer:** Enig idee wat je dan zou willen doen?

**Respondent:** We zijn nu eerst bezig met werkdruk. Mensen ervaren veel werkdruk. Dat resulteert dat het team minder werkplezier beleeft. Dus daar hebben we gezegd 'we focussen ons eerst op werkdruk, en als de werkdruk lager is'. Ik heb vorige week gezeten met het team en gevraagd, en dat heeft >TEAMLEIDER< ook aan het Helpdesk-team gevraagd, om drie punten op te schrijven hoe zij, zonder personeelsuitbreiding, kunnen zorgen voor minder werkdruk. Daar gaan we volgende week woensdag weer voor zitten. Dan gaan we die punten verzamelen en gaan we bekijken wat de oplossingen zijn wat het team heeft gemaakt. Dat ga ik ook nog doen, drie punten. En wat gaan we als eerste aanpakken en wat kunnen we aanpakken? En dat zou kunnen resulteren in de situatie dat er we ook meer werkplezier beleven. Hoeft niet. Maar als dat het wel zo is, zou dat betekenen dat we werkplezier niet echt hoeven aan te pakken. Dat is even te staven dan met het team.

**Interviewer:** Als eerst de druk van de ketel af gaat?

**Respondent:** Ja. We hebben bij ons allebei werkdruk. Bij Helpdesk zit het volgens de medewerker in de communicatie die niet goed loopt. Dus eerst de werkdruk.

**Interviewer:** Ligt daar nog een rol voor >TEAMLEIDER< als het aankomt op die communicatie?

**Respondent:** Ja, het is eigenlijk. Wat ze bedoelen is eigenlijk teamoverstijgend. Dus het is eigenlijk een zaak van >TEAMLEIDER<, >COLLEGA<, >MANAGER<, en ik, Dat we overstijgend goed de informatie brengen. Wat de Helpdesk ervaart is dat zij gewoon weinig input van andere teams krijgen. Wat we willen, wat we doen. Ook de communicatie van het team.

**Interviewer:** Als we dan terug gaan naar die Helpdesk-afdeling en >TEAMLEIDER< en we kijken naar de verantwoordelijkheden van >TEAMLEIDER<, wat voor verantwoordelijkheden heeft >TEAMLEIDER<? In termen van beslissingsbevoegdheid?

**Respondent:** Ik vind als teamleider dat je ook echt verantwoordelijkheid moet nemen voor jouw team. Budgettair-technisch kan >TEAMLEIDER< weinig. Ik heb het budget. Maar als het om processen gaat en die zijn team aangaan, is >TEAMLEIDER< helemaal vrij. Natuurlijk laat >TEAMLEIDER< mij weten wat het voor hem betekent. Dat rapporteert >TEAMLEIDER< bij mij. Ik kan daar wel overstijgend in zijn en dat ik zeg 'dat gaan we niet zo doen'. In de praktijk zie ik en verwacht ik dat >TEAMLEIDER< dat goed doet en ook die verantwoordelijkheid pakt.

**Interviewer:** Heb je voorbeelden van situaties of onderwerpen waar >TEAMLEIDER< over mag beslissen zonder jouw bijkomstigheid?

**Respondent:** Manier van hoe ze printers uitleveren, manier hoe ze computer uitleveren, manier hoe ze de inrichting doen van de computers inrichten. Daar hangt een hele structuur aan vast. De architect geeft bepaalde kaders aan. >TEAMLEIDER< mag binnen de kaders eigenlijk beslissen hoe die inrichting eruit ziet. Als het maar binnen de kaders past. Binnen die kaders mag >TEAMLEIDER< beslissen.

**Interviewer:** Heb je dan ook zo'n kader als het aankomt op het management van de personeelsleden?

**Respondent:** Vanuit mij bedoel je?

**Interviewer:** Ja vanuit jou. Hoeveel vrijheid >TEAMLEIDER< heeft om de mensen te managen.

**Respondent:** Ik geeft >TEAMLEIDER< heel veel vrijheid. Dat vind ik ook belangrijk. Kijk, het gaat goed. Ik vind het belangrijk dat ik **﻿**>TEAMLEIDER< de vrijheid geef omdat ik >TEAMLEIDER< ook de verantwoordelijkheid geef. Als >TEAMLEIDER< niet met die verantwoordelijkheid om kan gaan, wat ik niet zie, dan is het een ander verhaal. Maar >TEAMLEIDER< kan goed met die verantwoordelijkheid om gaan. Ik vind dat ook bij>TEAMLEIDER< thuishoren. Kijk, ik ben eindverantwoordelijk dus ik geef sturing als het echt mis gaat en geef ik richting. Zo heb ik veel richting gegeven aan van 'hé, volgens mij', toen heb ik mij met zijn gebied bemoeid. Ook een stukje. Ik vind. Heb je wel eens een call aangemeld?

**Interviewer:** Ja.

**Respondent:** Is die ook afgesloten?

**Interviewer:** Ja.

**Respondent:** Dan krijg je een automatische afsluitmail.

**Interviewer:** Juist.

**Respondent:** Dat ik heb gezegd 'ik wil daar verbetering in'. Ik wil dat iemand van jou team, van jullie team gaat terugbellen. Dus jij hebt een incident. Dan gaat iemand die uitvoeren en sluit die call. Als toevoeging, dat wil ik graag. Dat een call, die wordt dan uitgevoerd en gaat er iemand van de Helpdesk jou bellen van 'is het naar behoren uitgevoerd?'.

**Interviewer:** Customer satisfaction die omhoog moet gaan?

**Respondent:** Ja. Waar ging het nou eigenlijk over. Die input. >TEAMLEIDER< is dan verantwoordelijk voor het incidentmanagementproces, maar ik geeft daar ook wel input aan en sturing aan.

**Interviewer:** Duidelijk. Als we het hebben over budgetten. In hoeverre kan >TEAMLEIDER< zelfstandig financiële aankopen doen? Van een bos bloemen tot een training. Zitten daar mogelijk in?

**Respondent:** Bos bloemen nee. Bos bloemen niet. Dat moet ik allemaal accorderen. Wat **﻿**>TEAMLEIDER< wel heeft, dat heb ik dat al. >TEAMLEIDER< heeft wel een budget voor een investering. Elk jaar. Daar is **﻿**>TEAMLEIDER< verantwoordelijk voor. Dat gaat om een fors bedrag. Dit jaar was het 250.000 euro of zo, waar >TEAMLEIDER< verantwoordelijkheid voor had.

**Interviewer:** Maar echt investeringen in het personeel dat loop allemaal via jou?

**Respondent:** Ja, dat klopt. Die werkstations moeten om de drie jaar vervangen worden. Dit jaar moest er veel vervangen worden. In ieder geval veel geld.

**Interviewer:** Wanneer ben je tevreden met >TEAMLEIDER< presteren?

**Respondent:** Daar hebben we het wel eens over gehad. Ik vind het allerbelangrijkste dat iemand zich aan de gemaakte afspraken houdt. Dus ja, wat we afspreken dat we dat ook nakomen. Dan ben ik al gauw tevreden. Als we bepaalde doelen hebben gesteld, dan wil ik dat >TEAMLEIDER< zich daar aan vast houdt.

**Interviewer:** Zijn deze doelen voor meerdere jaren, of is dit voor één jaar?

**Respondent:** Ik heb een éénjaarplan.

**Interviewer:** En ook als het aankomt op het maken van die afspraken, doel je dan ook op afspraken die jullie maken omtrent personeel? Bijvoorbeeld afspraken omtrent opleidingen?

**Respondent:** Dat zou een toevoeging zijn. Ik heb wel input en ik doe ook de opleidingsplannen voor de teams. Dan weet ik ongeveer wie wanneer gaat. Eindverantwoordelijkheid voor de opleidingen ligt bij mij. Dus daar geef ik wat sturing aan. Daar heb ik nog geen concrete afspraken met >TEAMLEIDER< over gemaakt. Ik denk dat het wel een toevoeging zou kunnen zijn.

**Interviewer:** Heb je het idee dat >TEAMLEIDER< nog bepaalde kennis om het managen van personeel naar tevredenheid te kunnen doen?

**Respondent:** Waar de uitdaging van >TEAMLEIDER< in zit is dat. Eerst was >TEAMLEIDER< een coördinator. Een van de heren. De uitdaging van >TEAMLEIDER< is om meer kennis te hebben om overstijgend te zijn.

**Interviewer:** Er komt een hiërarchische relatie tot stand?

**Respondent:** Daar zijn de groei van >TEAMLEIDER<.

**Interviewer:** Heb je het idee dat er bepaalde taken zijn die buiten takenpakket van de teamleider vallen?

**Respondent:** Er zijn heel veel dingen. >TEAMLEIDER< heeft een team, ik heb een team. >TEAMLEIDER< zal alleen bij mijn vervanging mijn team leiden. Maar voor het team >TEAMLEIDER< is het veel wat >TEAMLEIDER< doet. Budgettair-technish. Ik zit even te zoeken wat niet bij >TEAMLEIDER< ligt.

**Interviewer:** Dus als ik het goed begrijp, alles wat binnen de muren van die Helpvalt, dat ligt bij >TEAMLEIDER<, op het geld na?

**Respondent:** >TEAMLEIDER< kan ook niet gebeld worden in de avond voor het team, ik wel. Dus als het Helpdeskteam ingezet moet worden, dan wordt ik gebeld. Dat is ook zo afgesproken. Dat is ook weer zo gescheiden tussen teamleider en hoofd. Hoofd moet ook in de avond aanspreekbaar zijn. Teamleider niet.

**Interviewer:** Komt het vaak voor dat er gebeld wordt?

**Respondent:** Voor het team van >TEAMLEIDER< minder, maar wel eens. Actie zetten op het team van >TEAMLEIDER< als er gebeld is ligt bij mij. Er zijn een aantal vaktechnische focusgebieden die niet bij Systeem- en Netwerkbeheer liggen. Daar, technische kennis moeten we dan uit het team van >TEAMLEIDER< halen. Dan bel ik.

**Interviewer:** Zou je eens een onder woorden kunnen brengen wat je van >TEAMLEIDER< verwacht in de rol van teamleider.

**Respondent:** Wat ik verwacht is dat >TEAMLEIDER< overstijgend het team leidt. Dat is ook de leercurve van >TEAMLEIDER<. Dat >TEAMLEIDER< overstijgend het team leidt en daar verantwoordelijkheid voor neemt.

**Interviewer:** Om dat waar te maken, heb je dan nog bepaalde manier voor in je hoofd hoe >TEAMLEIDER< dat moet doen? Of mag **﻿**>TEAMLEIDER< juist heel vrij handelen, als maar aan de verwachting voldaan wordt?

**Respondent:** We hebben natuurlijk wel afspraken met elkaar over wat we de komende maanden gaan doen. Ik vind wel dat we over de afspraken die we gemaakt hebben dat >TEAMLEIDER< daar naar kijkt en hoe deze afspraken in de tijden gehaald kunnen worden. Als >TEAMLEIDER< merkt dat die afspraken niet op tijd gehaald gaan worden, dat >TEAMLEIDER< escaleert naar mij. Of actie zet en en optie van om het wel te halen.

**Interviewer:** Als de afspraken, of afgesproken resultaten, maar gehaald worden en veiliggesteld worden, dan is de weg daar naartoe door >TEAMLEIDER< in te vullen?

**Respondent:** Ja, dat vind ik wel.

**Interviewer:** Heb je het idee dat >TEAMLEIDER< goed op de hoogte is van jouw verwachtingen?

**Respondent:** Ja, dat heb ik wel.

**Interviewer:** Hoe? Hoe zou >TEAMLEIDER< kunnen weten wat jij van >TEAMLEIDER< verwacht?

**Respondent:** Ik heb iedere week bila's met >TEAMLEIDER<. Daar spreek ik ook mijn verwachtingen uit. In die optiek denk ik dat >TEAMLEIDER< daar op terug kan pakken.

**Interviewer:** Kan >TEAMLEIDER< dan ook nog vasthouden aan het jaarplan?

**Respondent:** Het jaarplan is afdelingsbreed, van Syteem- en Netwerkbeheer en Helpdesk.

**Interviewer:** Daar zou >TEAMLEIDER< niet veel verantwoordelijkheden uit af kunnen leiden?

**Respondent:** Er staan wel gebieden op het afdelingsniveau van de Helpdesk. Dan verwacht ik dat zijn gebieden, daar heb ik dan ook over gesproken met >TEAMLEIDER<, daar ligt de verantwoordelijkheid van **﻿**>TEAMLEIDER<.

**Interviewer:** Uit nieuwsgierigheid. Als we een jaarplan hebben. Is daar dan ook een stuk HR in verwerkt? Dat je als teamleider bijvoorbeeld iets met opleiden moet doen of met jaargesprekken?

**Respondent:** Wil je hem zien, het jaarplan?

**Interviewer:** Ja, waarom niet.

**Respondent:** Ik heb ook een stukje personeel in het jaarplan opgenomen. Ik heb één sheet met allemaal personeelszaken erop. Dan hebben we het over een stukje team work, een stukje team building, dat we borrels gaan organiseren met elkaar. Een stukje halfjaarlijkse evaluatie en een stukje jaarlijkse evaluatie. Toevallig heb ik net voor jou een halfjaarlijkse evaluatie gedaan met een redelijk nieuw iemand. Dat gebeurde hier nooit. Nu voor de eerste keer gedaan. Wat ik ervaarde is dat het jaargesprek een keer per jaar werd gedaan. Dan werd over een jaar weer een jaargesprek gedaan en werd er helemaal niet meer gerefereerd naar het jaar daarvoor. En dan van 'oh, weer goed. Oke, prima'.

**Interviewer:** Meer een formaliteit?

**Respondent:** Precies. Ja, we moeten dat maar doen. Dat vind ik iets raars. Daarom probeer ik nu ook om afspraken in november en december gemaakt te hebben met het personeel, ook wat zij zouden doen, en dat we dan over een half jaar het er met elkaar over hebben. En de ene zal meer aan de afspraken werken dan de ander.

**Interviewer:** Maar daar ben je dan wel een half jaar eerder achter.

**Respondent:** Ja, precies.

*﻿Respondent laat PowerPointpresentatie zien aan interviewer*﻿.

**Respondent:** Niet op >AFDELING<-breed. Maar op Systeem- en Netwerkbeheer en Helpdesk. Het is echt mijn gebied zeg maar. Ik heb daarvoor een gesprek met medewerkers gehouden. Wat vinden jullie nou waar we aan moeten werken? En is er een rapport geweest over de helpdesk en een jaarplan >AFDELING<. Daar is weer een >AFDELING<-beleidsplan uit voortgekomen. Dat wil zeggen dat we niet groot innoveren, maar meer een midde modus.

**Interviewer:** Een soort constante verandering en blij blijven benen?

**Respondent:** Ja. Dit dingetje vallen sommige mensen. Maar wat ik bedoel is. Continuïteit, dus het beheer en zorgen dat applicaties en systemen draaien, en dus niet als we een nieuw project hebben dat dat voorrang krijgt. Dat is mijn, snapje?

**Interviewer:** Ja.

**Respondent:** Evaluaties na grote changes. Gebruikmakend van scrum. Ken je scrum?

**Interviewer:** Nee.

**Respondent:** Scrum komt voor in projectmanagement gericht op >AFDELING< en zeg van 'elk ding, elke change, elk project'. Dat je zegt van 'we hangen er een proces in en zo gaan we het doen'. Het is niet echt een grote planning voor langere tijd, maar meer wat er nu gedaan moet worden. Dat doe je regelmatig met elkaar. Ze noemen het ook wel eens de postid. Scrum is echt van 'wat heb ik nog allemaal te doen?'. Dat pakken we dan op en dat is dan wat we moeten toen.

**Interviewer:** In plaats van dat je een vijfjarenplan uitlegt, ga je terug en bepaal je op hele korte termijn wat er gedaan moet worden?

**Respondent:** Ja, klopt. En dan ga ik met een medewerker op cursus daarvoor. Werkplekverbetering is echt dat we kijken van 'hoe kunnen we werkplekken nu verbeteren?'. Bijvoorbeeld over geluid. En ik kan het ze niet helemaal beloven want er komt een hoop geld bij kijken.

**Interviewer:** Wat bedoelen ze precies met geluid?

**Respondent:** Dat als ze aan het werk zijn, dat ze aan de overkant dan geluid horen. Mensen kunnen aan het kletsen zijn en dat haalt mensen uit hun concentratie. Teambuilddagen gaan we extern doen. Op tijd komen. Ik vind het vervelend als mensen te laat komen.

**Interviewer:** Merk je dat mensen al minder vaak te laat komen?

**Respondent:** Het is echt een cultuur van jaren. Mensen vinden het heel lastig. >TEAMLEIDER< kwam ook af en toe te laat. Daar zie ik nu verbetering in. Ik zie dat als cultuur. Wij hebben als ziekenhuis een cultuur van 'oh dan maar vijf minuten te laat'.

**Interviewer:** Dat duurt heel lang om dat omgezet te hebben in iets nieuws?

**Respondent:** Ja, precies. Die bewustwording. Dat hoort allemaal bij aandacht voor de mens.

*﻿Respondent laat een overzicht van projecten zien.*﻿

**Interviewer:** Dat zijn bijvoorbeeld projecten waar >TEAMLEIDER< zich mee bezig houdt, maar daar ben ik ook bij betrokken. Dit is >TEAMLEIDER<. Dit is een stukje dat overkoepelend is. Dat zijn wel de dingen die bij >TEAMLEIDER< horen. >TEAMLEIDER< heeft relaties met veel verschillende partijen. De planning, daar is >TEAMLEIDER< ook verantwoordelijk voor. Dat ligt ook echt bij >TEAMLEIDER<. Waar >TEAMLEIDER< eerst heel erg de planning deed, vind ik dat het team ook de planning moet doen.

**Interviewer:** Bedoel je dan de personeelsplanning. Wie wanneer werkt. Of bedoel je met planning de inzet van middelen?

**Respondent:** Stel je voor dat er iets nieuws binnenkomt. Bijvoorbeeld een nieuw type werkstation dat binnenkomt, dat ook de werknemers zelf kunnen plannen.

**Interviewer:** Is dit een soort competentie om dat te kunnen?

**Respondent:** Zo zou je het kunnen zien. Het is uiteindelijk de bedoeling dat medewerkers zelf kunnen plannen. >TEAMLEIDER< maakt de planning en ik bewaak de planning. Ik leg het ook wel bij het team neer hoor. Dat ze zien van 'ik heb te veel gepland'. Om te zorgen dat de planning weer goed komt.

**Interviewer:** Aan >TEAMLEIDER< straks de taak om de medewerkers te laten plannen?

**Respondent:** Ja, dat doen ze momenteel niet. Dat doet >TEAMLEIDER<.

**Interviewer:** Hoe zou je willen dat >TEAMLEIDER< dat doet? Wil je dat >TEAMLEIDER< deze medewerkers op cursus stuurt of wil je dat >TEAMLEIDER< zelf een soort training gaat organiseren?

**Respondent:** Ik denk eerder gewoon overleggen van 'goh, hoe gaan we dat nou doen?'. >TEAMLEIDER< weet hoe het moet. >TEAMLEIDER< moet dat gaan overdragen door middel van een klein workshopje.

*﻿Respondent laat een tabel zien. Op de horizontale as zijn de maanden weergeven. Op de vertical as zijn de projecten weergeven. In de tabel staan kruisjes die aangeven welke projecten in welke maanden worden uitgevoerd.*

*﻿*﻿ **Respondent:** En dan de tijdplanning. Alle acties die gedaan worden. En dan is het mijn sturing van 'goh, wie loopt er nou achter?'. Daar loop ik op te sturen.

**Interviewer:** Om de uitloop te beperken?

**Respondent:** Ja, klopt.

**Interviewer:** Dan had ik eigenlijk als laatste vraag, wat verwacht je van >TEAMLEIDER< als het aankomt op het gedrag van >TEAMLEIDER< als teamleider? Hoe moet >TEAMLEIDER< zich gedragen naar jouw mening.

**Respondent:** ... Goeie. Goeie vraag. Hoe >TEAMLEIDER< zich moet gedragen. Dat zei ik net al een beetje. Dat >TEAMLEIDER< zich bewust is dat >TEAMLEIDER< overstijgend moet werken. Dat **﻿**>TEAMLEIDER< bewust is dat >TEAMLEIDER< een relatie heeft met het team en relatie heeft met mij en het hoger management. Dus dat wat verteld wordt aan >TEAMLEIDER<. Wat vroeg je precies?

**Interviewer:** Wat voor een gedrag?

**Respondent:** Dat vind ik een moeilijke vraag.

**Interviewer:** Vind je bijvoorbeeld dat >TEAMLEIDER< een voorbeeldfunctie moet hebben?

**Respondent:** Oh, ja zeker. En dat bedoel ik ook net met overstijgend. >TEAMLEIDER< heeft een voorbeeldfunctie voor de Helpdesk en daar moet >TEAMLEIDER< ook op acteren.

**Interviewer:** En als voorbeeldfunctie, hoe vervul je die?

**Respondent:** Representatief zijn. Ja, hoe vervul je dat kun je dan vragen. Eigenlijk vind ik het, even heel gechargeerd gezegd, de eerste klap opvangen en bij een klacht, en dat goed vertalen. Een klacht goed ontvangen, representatief zijn van 'goh, ik hoor je, ik voel je, ik begrijp je'.

**Interviewer:** Een stukje empathisch vermogen?

**Respondent:** Empathisch vermogen ja. Ook al begrijp je het niet. Dat je wel een houding hebt. Je bent jezelf. En dat >TEAMLEIDER< dat goed vertaalt richting het team. En dan zonder de emoties verteld. Gewoon van 'dit en dit heb ik gehoord, dit is wat ik van jullie verwacht'.

**Interviewer:** Heel strak heel zakelijk?

**Respondent:** Nou, dat hoeft niet heel strak en zakelijk. Soms dan weer wel. Maar ik denk wel zakelijk richting de klant en begrip vol. En ik denk dat >TEAMLEIDER< dat per situatie moet verzinnen. Soms is wel strak nodig als iemand niet mee wil werken of bewegen, of het er niet mee eens is. Dan moet >TEAMLEIDER< strak zijn. Maar, als ik het team van >TEAMLEIDER< ken, dat ze niet altijd strak aangesproken hoeven worden.

**Interviewer:** Je noemde net empatisch vermogen. Denk je dat >TEAMLEIDER< nog bepaalde competenties nodig heeft om goed het kunnen functioneren als teamleider.

**Respondent:** >TEAMLEIDER< heeft wel een overwicht. Maar ik denk toch echt wel het stukje overstijgen. Die leercurve is echt nodig.

**Interviewer:** Denk je dat dat met leiderschap te maken heeft? Dat je in plaats van in het team, boven het team gaat staan. Komt dat neer onder leiderschap?

**Respondent:** Ja, zeker. Het is leiding geven aan. Eerst stond je in het team. Nu sta je boven het team.

**Interviewer:** Is het ook een stukje autoriteit opeisen?

**Respondent:** Ja, en dat kan >TEAMLEIDER<. Ik denk alleen dat het een stukje bewust moet laten zien. >TEAMLEIDER< zit af en toe nog in het team.

**Interviewer:** Zal **﻿**﻿>TEAMLEIDER< die leercurve meer te pakken krijgen zodra die operationele taken losgelaten worden? Die scheiding wordt dan denk ik groter. Je werkt nog steeds mee, een stukje, maar je staat er nu voornamelijk naast en boven.

**Respondent:** Ja, en ik denk dat >TEAMLEIDER< daarmee vooral in de rol van teamleider wordt gezet. Eigenlijk wilde ik gewoon zeggen dat >TEAMLEIDER< nul procent uitvoerende taken heeft. Maar op dit moment kan dat nog niet. Daarom zeg ik max 25 procent. En afbouwen.

**Interviewer:** Afbouwend richting nul.

**Respondent:** Ja, ik zei ook. Eigenlijk wil ik dat het nu procent is.

**Interviewer:** Even de fubes ombouwen?

**Respondent:** Nu hebben afgesproken max 25. Misschien doen we het jaar erna of het jaar erna misschien niets. Omdat we nu nog wel een efficiencyslag te slaan hebben op de Helpdesk. Als we dat gedaan hebben, dan kunnen we verder kijken.

**Interviewer:** Ik heb alles gevraagd wat ik jou wilde vragen. Ik weet niet of je zelf nog dingen aan het interview wilde toevoegen?

**Respondent:** Nee, ik vond het een heel fijn gesprek.

*Einde transcript*

# Transcript Interview Middle Manager 7 and FLM 11

**Duur interview: 62 minuten en 10 seconden**

**Functie: Bedrijfsleider (Respondent B) en Teamleider (Respondent
 A)**

**Afdelingen onder supervisie: Healthcare**

**Gender: vrouw, vrouw**

**Werkervaring in functie: n.a. / 4 jaar**

**Interviewer:** Wat zijn de dagelijkse werkzaamheden van een teamleider?

**Respondent A:** Teamleiders in het algemeen?

**Interviewer:** Vooral wat jij doet.

**Respondent A:** Wat ik doe. Ik ben teamleider van twee poli's. Van >AFDELING< en >AFDELING< en mijn grootste taak is, op >AFDELING< doe ik de operationele aansturing. Eigenlijk alles wat rechtstreeks op de werkvloer gebeurt. Dat gaat over de planning. De planning van dokters, de paramedici die aanwezig zijn. Ik moet zorgen dat het rooster klopt. De assistentes plannen zelf. Daar ben ik wel degene als er problemen zijn, dat je daarin springt. Ik moet kunnen meewerken. Dat is ook wat gezegd wordt, 25 procent. Ik ben van oorsprong, heb ik een paramedische opleiding in de >AFDELING<. Ik ben technisch >OPLEIDING< assistent. Dus als het nodig is, mijn witte jas hangt hier, trek ik een witte jas aan en kan ik spreekuur doen. Maar ik moet zeggen, stel je niet voor dat dat dagelijks of drie keer in de week voorkomt. Dat is vooral bij ziekte of bij uitval.

**Interviewer:** Wanneer is de laatste keer dat je meegewerkt hebt, denk je?

**Respondent A:** Oh. Dat was toen >NAAM< ziek was. Ik denk dat dat nu. Wat is dus meewerken? Spreekuur heb ik gedaan. Ik denk dat ik twee weken. In jou vakantie.

**Respondent B:** Twee weken geleden.

**Interviewer:** Dat is vrij recent nog?

**Respondent B:**  Het is echt wel af en toe hoor.

**Respondent A:** Het wisselt enorm. En ik vind, je kunt een enorme discussie gaan voeren 'wat is meewerken?'. Is dat als je een witte jas aan hebt en over de poli loopt? Ja, dan werk ik mee in mijn oude vak. Of is meewerken als ik de planning doe die op heel veel poli's goed gestroomlijnd is en waard de polikliniek-assistent of doktersassistent de planning doorzet? Voor >AFDELING< doe ik het omdat het een heel complex gebeuren is. Alles is meewerken want alles is...

**Interviewer:** Achter de schermen bedoel je? Dat valt eigenlijk ook onder meewerken?

**Respondent A:** Ja.

**Interviewer:** Van al die dingen die je moet doen. Daar zit ook een stukje HR in verscholen?

**Respondent A:** Ja.

**Interviewer:** Wat herken je zelf? Wat vind jij gerelateerd aan HR?

**Respondent A:** Bijvoorbeeld de jaargesprekken. De verzuimgesprekken. De voortgangsgesprekken. En werkoverleg. Dat is ook iets dat je moet doen. Het werkoverleg van de paramedici, de assistentes. Ook als het nodig is muteren, van jou toch geleerd? Muteren van medewerkers als er iets verandert aan een contract of zo. Dat is echt heel erg HR. In Youforce bijhouden van dingen die binnenkomen. Dat kunnen zijn wijzigingen in adres tot declaraties. Dat is heel erg HR vind ik. Dat zijn hele simpele dingen, maar het is wel. Verzuimsignaal dus ook het bijhouden van ziekteregistraties of als iemand zich ziek meld of contact houden. Dat soort dingen.

**Interviewer:** Maken jullie ook nog gebruik van seniors?

**Respondent A:** Nee. Wij hebben op >AFDELING< en >AFDELING< op beide poli's hebben we geen seniors.

**Respondent B:** Meer

**Respondent A:** Dat zou ook wel veel zijn toch?

**Respondent B:** Nou vind ik ook. Nee. Wij. Nee, die hebben we niet meer.

**Interviewer:** Is er nog een reden waarom jullie zoiets hebben van 'wij kunnen zonder senior uit de voeten?'.

**Respondent B:** Ik denk dat >AFDELING< een behoorlijk volwassen team is. Die hebben een mega crisis achter de rug en zijn daar enorm van gegroeid zelf ook. En iedereen heeft sowieso bij andere poli's eigen werk of taken als, of soms heet het klussen, en dat is min of meer gewoon een administratieve taak of een inhoudelijke taak die iemand voor zijn rekening neemt. Dus ik ben heel van de verantwoordelijkheid en werkzaamheden zo laag mogelijk drukken. Naar de werkvloer. Die hebben de meeste kennis, hebben er de meeste last van. En er is natuurlijk altijd iets van leiding en coördinatie nodig, maar wij proberen daar toch een coachende rol in aan te nemen. En senior is wat naar mijn idee voornamelijk uit de behoefte komt van de medewerker, dan wel een deel van het team of het team. En die behoefte is er niet.

**Respondent A:** En je merkt wel. Je hebt mensen. Talenten komen altijd boven drijven en in het team, bijvoorbeeld de een is heel goed in plannen. De ander is heel goed om in de computer even een bestandje te maken of een Excel. En zo heeft ieder eigenlijk is misschien dan senior, of een heel klein stukje. Met elkaar is een gelijk. En dat is ook bij de paramedici zo.

**Respondent B:** Ik vind dat je echt een coördinerende taak moet geven over. Stel je voor ik heb drie buitenpoli's met >AFDELING<, dan zou het misschien best handig zijn om daar dan eens echt een coördinerende rol op te zetten. En dan heb je niet zoveel mogelijkheden. Je gaat niet nog een teamleider aannemen in de omvang van dit team. Dat zou dan een senior kunnen zijn.

**Respondent A:** Maar ze hebben zelf. Er was iemand die de ambitie gehad om senior te worden. Toen had het team heel erg aangegeven, heel open 'wij hebben helemaal geen behoefte aan, wij regelen nu het ook met elkaar en onderling'.

**Respondent B:** We hebben >TEAMLEIDER< en we hebben >BEDRIJFSLEIDER<.

**Respondent A:** Dat was voor hun voldoende.

**Interviewer:** Is er dan een centraal aanspreekpunt van de dag?

**Respondent A:** Ja. Er is. Daar is poli >AFDELING< is daarin heel strikt. Die hebben een dagcoördinator. Ik hoef daar echt niet de poli op te komen en het even vergeten te zijn of ik zie >BEDRIJFSLEIDER<, ik noem maar wat, die is die dag de dagcoördinator. Dat is heel handig. Op Oog is dat was later, een beetje uit nood geboren, en dat komt de ene dag beter dan de andere dag uit de verf. Opzicht is het wel helder bij wie je moet zijn. Dat proberen we dan ook zo te regelen dat. Er zijn nieuwe dokters die nog heel veel moeten leren en in ieder geval moeten weten naar wie ze toe moeten gaan en ook niet nog, in alle hectiek van de dag, moeten gaan zoeken 'naar wie moet ik gaan?'. Nou, dat de ene keer beter dan de andere keer.

**Interviewer:** Als teamleider kun je voor heel veel verschillende onderwerpen verantwoordelijk zijn, zitten hier onderwerpen tussen die jij herkent en waar jij je mee bezig ben?

**Respondent A:** Heb je zelf het lijstje ook? Kijk jij met mij mee. Nou werving, toch eigenlijk ook wel. Dokters werven. *﻿*﻿*﻿Respondent A lacht*﻿. Nee, dan de werving is echt iets wat. Bedoel je echt de hele procedure?

**Interviewer:** Het zoeken van nieuwe mensen als het ware?

**Respondent A:** Is eigenlijk iets wat wij samen doen he? Ze is er wel bij betrokken, maar meestal doen we daar even samen in kijken en overleggen van 'goh, er gaat iemand weg', want we werken vooral binnen de paramedici met verschillende disciplines en mensen die meer dan een discipline hebben. Dus het aantrekken van personeel heb ik het dan over. Hoe gaan we het aanpakken, wat gaan we doen? Daar zijn we dan samen. Dat we kortsluiten 'wat is handig, was is wijs?'. En de vacature, ik heb er een of jij hebt er nog een.

**Respondent B:** ik beslis bij voorkeur nooit alleen. Nee. Ik ben zeker. Het feit dat je iemand moet werven. Dat start heel logisch en formatieberekening dat doe ik wel. Ik doe het altijd open. En we beslissen het niet eens met z'n tweeën, we hebben altijd de sollicitatiecommissie. Maar voor het aanjagen, dat komt of uit de begroting, of komt uit de operatie en dan zegt >TEAMLEIDER< van 'er komt toch een nieuwe flexer' of 'die gaat weg'. En dan maken we samen een plan. Maar ik ben eindeverantwoordelijk hoeveel geld er aan uitgegeven wordt. Dus daar beslis ik dan wel over. Maar altijd in samenspraak.

**Interviewer:** De begroting, stellen jullie die samen op?

**Respondent B:** Dat is wel het plan. Ik heb het tot nu toe alleen gedaan. Daar was wel een goede reden voor. Er viel niets te begroten. Ik had geen dokters, ik had geen idee wat voor omzet ze dit jaar zouden draaien, echt geen benul, dus daar heb ik niet al te veel tijd aan besteed om heel kort gezegd. Maar normaal is dat iets waar in ieder geval een deel van de input komt van de >TEAMLEIDER< en ik vind het ook voor de ontwikkeling van de teamleider vind ik het belangrijk om dat samen te doen. Er zit wel vaak een enorme squeeze op, waardoor je wel eens flink door moet pakken. De begroting is eigenlijk ook maar een momentopname. Het wordt allemaal heel groot gemaakt, omdat het alleen maar nodig is, maar op het moment dat je nieuw voortschrijdend inzicht hebt, is dat alweer anders.

**Interviewer:** Maar wel iets waar een teamleider in betrokken hoort te worden?

**Respondent A:** Ja. Absoluut. Als ik het even langs kijk...

**Interviewer:** Welke dingen vallen je op?

**Respondent A:** Sorry?

**Interviewer:** Welke onderwerpen vallen je op?

**Respondent A:** Selectie, hoort ook bij werving, dat is een geheel voor ons. Introductie doe ik zelf weer minder. Ik zorg dat de introductie kan verlopen.

**Interviewer:** Je faciliteert?

**Respondent A:** Ja. Ik faciliteer dat en ik zorg wel, dat is wel mijn stukje van de introductie, dat is weet je dat iemand een pasje heeft, een jasje krijgt. Maar de introductie, eigenlijk het ziekenhuis verzorgt een deel. Moeten een stukje zelf doen via...

**Interviewer:** Met die tablet geloof ik?

**Respondent A:** Ja. Nee. Ze moeten thuis al iets doen geloof ik. Ze krijgen een link naar een aantal filmpjes, dat is ook iets dat in het LMS, het leermanagementsysteem, zit. Dat wordt thuis gedaan. De echt introductie is op de afdeling en dat proberen we zoveel mogelijk te laten doen door de naaste collega's. Dus komt er een nieuwe optometrist, dan zorgen we dat de optometrist, die eigenlijk va volwassen is, maar wie wel dezelfde taken, dat die gaat helpen en ook tijdens gewoon de introductie. Ja. Als je introductie inwerken noemt. Introductie is echt dat stukje dat ik net noemde. Inwerken is iets dat daarna komt en dat is iets wat door naasten, gelijken, zoveel mogelijk op de werkvloer gebeurt. Maar ik faciliteer het dan wel. Bijhouden van personeelsbestanden. Ja. Dat gaat eigenlijk...

**Respondent B:** HR. >TEKST GESCHRAPT<. Ik vind dat de bedrijfsleider ook nog altijd wel tot een zeker mate op de werkvloer betrokken moet zijn. Dus een altijd van de jaargesprekken doe ik altijd nog zelf. We doen ook wel eens een gesprek samen. Het is dan wel geen jaargesprek, maar voortgangsgesprekken met iemand. Degene die het verslag maakt, zorgt dat het in het personeelsdossier komt. En op het moment zeg maar dat iemand, stel je voor dat iemand, dat hebben we nog helemaal niet aan de hand gehad, of echt een beoordelingstraject zou krijgen, of het gewoon heel slecht doet, dan zou dat voor >TEAMLEIDER< een taak zijn. Om dat dan ook bij te houden, verslagen te maken.

**Interviewer:** Ben jij dan in de lead?

**Respondent B:** Nou. Ik. Bij >AFDELING< nog niet aan de hand gehad. Ja dit is natuurlijk ook niet helemaal waar. We hebben natuurlijk die gekke uit >NAAM ZIEKENHUIS<. Soms ben ik wel in de lead. Gaat ook een beetje. Wij zoeken ook qua tijd en qua impact wat de beste keuze zou zijn.

**Respondent A:** Sowieso overleggen we ook van. Soms is het handig als ik iets doe omdat diegene dan nog kan escaleren naar >BEDRIJFSLEIDER<. Als >BEDRIJFSLEIDER< het doet, kan diegene niet mee escaleren. Dat overleggen we ook wel eens van 'ik doe het' en als het dan escaleert, dan kan het naar >BEDRIJFSLEIDER<. Dat is eigenlijk gewoon een mate van afstemmen.

**Interviewer:** Tactisch?

**Respondent A:** Ja.

**Respondent B:** Het is wel zo, als je bij mij op gesprek moet komen, dan ben je onmiddellijk aan de beurt hoor, over het algemeen.

**Interviewer:** Zie ik het dan ook zo voor me dat met de verdeling van de jaargesprekken, dat de zwaardere bij jou liggen?

**Respondent B:** Nee. Dat doe ik juist niet. Want dan zou het zijn. Kijk. Het jaargesprek is niet echt zoals een functioneringsgesprek he? Het is moment in het jaar waarop je terugkijkt en er zit wel een component aan, een beoordelingscomponent, dat bespreken wij al van te voren. Zeker als iemand enorm afwijkt, naar onder of naar boven, maar dat doe je dus juist. We hebben toch een beetje onze vast mensen, mensen die eraan gewend zijn om met ons het jaargesprek te voeren.

**Respondent A:** Wij maken eerder de verdeling dat je op de inhoud over het vak kunt praten, niet over de beoordeling. Maar wel 'waar staat iemand, wat voor ambities heeft iemand, wat heeft iemand voor scholing, waar wil die naar toe?'. Ik heb wat meer >MEDISCHE SPECIALISATIE< kennis. >BEDRIJFSLEIDER< heeft een heleboel andere kennis. Dus komt het vaker dat we daar een afweging in maken, dat ik wat meer paramedici doe en >BEDRIJFSLEIDER< eventueel wat van de dokters en de polikliniekassistenten wat. De verdeling is dus niet op 'die is zwaar of dat'. Nee.

**Interviewer:** Maar maakt jullie dat een sterk team? Het feit dat jij veel medische kennis hebt en jij wat meer bedrijfskundige, management kennis?

**Respondent B:** Gisteren gaf iemand ons een heel mooi compliment vind ik zelf. Je zou dat ook als een belediging op kunnen vatten, maar ik zag het als een compliment. We waren op een congres en iemand vroeg 'hoe lang werken jullie al samen?'. Ik moest even denken. Vanaf 2011. Ik dacht echt dat jullie al twintig jaar samenwerkten. Hier in het ziekenhuis zeggen ze 'daar gaan Jit en Jul van >AFDELING<'. Ik denk dat wij behoorlijk complementair zijn qua kennis, maar ook hoe we erin staan. Ik ben van de grote lijnen, >TEAMLEIDER< is erg van details. Dat is echt super fijn. En >TEAMLEIDER< probeert mij bij de details te houden en ik probeer >TEAMLEIDER< naar de grote lijn toe te trekken. Ik denk dat dat echt een hele goede aanvulling is.

**Respondent A:** Ja.

**Interviewer:** Denken jullie dat de setting hier, jullie zitten met z'n tweeën in een ruimte, dat zie je denk ik niet heel veel in het ziekenhuis?

**Respondent A:** Dat is ook hopeloos want ik heb ook nog twee andere afdelingen. Iemand wil ook wel eens wat alleen met >TEAMLEIDER< bespreken, maar er is geen ruimte. Dus uh. Het heeft ook voordelen. We kunnen heel snel schakelen daardoor.

**Interviewer:** Ja. Daar was ik een beetje naar op zoek. Zoals jullie bij elkaar zitten, zouden andere EVRs dat ook moeten doen?

**Respondent A:** Ik denk van niet. Dat is ook wel leuk. Jij vertelt wat en dat kun je ook niet voorbespreken. >BEDRIJFSLEIDER< had in het verleden een werkplek ergens anders in het ziekenhuis en ik had een werkplek op de poli. Goed je zit niet dan op een kamer, maar je spreekt elkaar wel. Je zoekt elkaar op. Ik heb niet eens het gevoel alleen nu merk ik, je overlegt sneller even dingen. Ik merk ook dat ik soms dingen vraag waarvan ik denk 'dat weet ik zelf ook wel, maar ik heb nu toch >BEDRIJFSLEIDER< tegenover'. Ik meld soms een wat waarvan ik denk...

**Respondent B:** Moet dat nu?

**Respondent A:** Dan zou je denken 'dat komt als >TEAMLEIDER< voorbijkomt' of dat komt wel. Ik denk niet dat dat een negatieve impact heeft.

**Respondent B:** Nee. Je hebt als leidinggevende op z'n minst een rustige werkplek nodig, die hadden we eigenlijk allebei niet. Het gevecht om de ruimte is altijd groot en iedereen had commentaar dat >TEAMLEIDER< in deze grote kamer zat. Toen zei ik 'zet mij er maar bij in'. We moeten er nu weer uit want iemand anders wil er in.

**Interviewer:** Dus dan wordt het weer een beetje uit elkaar getrokken?

**Respondent B:** Ze moeten eerst verzinnen waar naartoe.

**Respondent A:** Ik denk dat het glamping wordt ergens. Dat weten we niet.

**Interviewer:** Ergens bij de flexplekken?

**Respondent B:** Als leidinggevende, je moet echt een laag niveau hebben qua inloop vind ik. Mensen moeten makkelijk bij je terecht kunnen, zeker als er iets is. Als iemand van mijn andere afdelingen komt is dat toch lastig. Mensen willen soms wel eens wat met >TEAMLEIDER< bespreken of soms wel eens wat met mij bespreken. Als je allebei zit te bellen. We werken er nu nog aardig omheen.

**Respondent A:** Het scheelt wel, wij hebben een goede werkrelatie onderling. Een goede verstandhouding. Wij zijn het niet altijd eens. Niet altijd even aardig naar elkaar. Maar wij weten wel wat wij bedoelen en kunnen elkaar, daardoor, ook alles zeggen en vinden we altijd de modus en zijn wij gewoon eerlijk tegen elkaar. Als ik iets moet bespreken, dan jij eruit of ik raadpleeg jou agenda alvast als ik hier een gesprek plan. Het voors en tegens maar je hebt wel van die setjes hier in huis waarvan ik denk 'als je die in een kamer gaat zetten dan leggen we een dweiltje voor het bloed bij de deur'. Misschien wel of niet.

**Respondent B:** Ik denk wel. Ik zou met al mijn teamleiders in een ruimte kunnen werken. Ik ben wat dat aangaat bijzonder transparant. Voor de medewerker vind ik het beslist niet ideaal. Voor het contact met mijn collega-bedrijfsleiders is het soms ook lastig. Dat geldt voor jou als teamleider ook. Als je wat als teamleider wil bespreken zit je bedrijfsleider erbij. Dan voelen wij dat vaak wel aan en gaan wij.

**Respondent A:** Gaan wij er gewoon uit.

**Interviewer:** Maar mocht er straks een nieuwe ruimte beschikbaar komen, zouden jullie dan voorstander zijn om dichter naar de afdeling toe te gaan qua locatie?

**Respondent B:** Eigenlijk moeten we op de afdeling zitten.

**Respondent A:** Voor mij. Het is ook eigenlijk gebruikelijk dat de teamleider op de afdeling. Maar ik had bij >AFDELING< dus een plekje. Maar dat was een dermate vervelende plek. Ten eerste was hij nog als werkplek voor de assistentes. En daarbij was het een plek die alleen toegankelijk was langs de balie, achter de balie, door het secretariaat. En als je dan ook nog teamleider van een andere afdeling bent, iedereen komt binnen en begint al bij de balie te roepen 'waar is >TEAMLEIDER<' en het kleppert achter elkaar door. Het was onrust aan alle kanten. En door de crisis die we op >AFDELING< hadden had ik de planklus en dat was iedere keer zo veel. Daarbij komt mijn slechthorendheid dat storend is voor mij, maar ook storend is voor mijn omgeving. Dus daardoor is gekozen voor een andere werkplek dat mij enorm veel gescheeld heeft met een boel dingen. Nu moeten we kijken. Zoals het nu is, ik kan niet terug naar de poli want er is geen plek. Er komen als werkruimte voor de paramedici, dokters, AIOS, ANIOS, tekort. Ik zeg 'dat wordt glampen of op de WC met een laptop op mijn schoot, iets dergelijks moet het worden'.

**Respondent B:** Ik heb mijn afdelingen dan verspreid door het huis en ik heb dan wel dat het niet veel uitmaakt waar ik zit. Ik heb bij de >AFDELING< gezeten, ik heb bij >AFDELING< gezeten. Ik word al jaren lang verplaatst.

**Interviewer:** Zien jullie ook, ik spring een beetje door het lijstje heen, zien jullie ook dat bij de teamleider een communicatierol ligt?

**Respondent A:** Hoe bedoel je in welke vorm een communicatierol?

**Interviewer:** Ik kan me voorstellen dat als er zo tussen zit tussen medewerker en hoger management, dat je een soort doorgeeffunctie hebt om van wat hier besproken wordt...

**Respondent B:** Een hele grote rol.

**Respondent A:** Dat is wel een hele grote rol. Maar niet. Kijk, als er een overleg is geweest, dan ben ik degene die met de punten gaat naar >BEDRIJFSLEIDER<. Punten waar ik zelf niet mee of waar geld aan verbonden is wat beslissingen vraagt wat buiten mijn bevoegd heden vallen. Dan ga ik naar >BEDRIJFSLEIDER<. En dan zeg ik '>BEDRIJFSLEIDER<, dit is besproken, dat zijn de vragen'. Maar als iemand bij mij komt van 'goh, wil jij van mij aan >BEDRIJFSLEIDER< vragen of...

**Respondent B:** Nee. Nee. Nee.

**Respondent A:** Dat gebeurt dus niet. Je weet waar >BEDRIJFSLEIDER< zit. Je weet het telefoonnummer van >BEDRIJFSLEIDER<. Dat is wederzijds. Ik denk wel de communicatie naar, af en toe als het nodig is dus wel.

**Respondent B:** En het vertalen naar de werkvloer van dingen als 'dit moet er gedaan worden, de inspectie komt eraan, die moet worden geregeld'. Vandaag hadden we het nog over informatieveiligheid. Hoe is dat eigenlijk ingeregeld op de poli >AFDELING<? Er moeten altijd tien dingen tegelijkertijd. Dus af en toe verlies je wel eens iets uit het oog. Dat gaat per voorkeur via >TEAMLEIDER< dat doe ik niet rechtstreeks.

**Interviewer:** Hoe doe je dat dan, dat communiceren? Hoe gaat dat?

**Respondent A:** Ik probeer zo veel mogelijk. Zoals nu kwam >BEDRIJFSLEIDER< bij mij. De informatieveiligheid. Ik was net op de poli en vraag ik wel en person 'komt dit of dit vaak voor, hoe doen we dat'. Dan hoor ik het even, maar bij voorkeur zet ik het toch, om iedereen te bereiken en dezelfde boodschap naar iedereen uit te dragen, zeg ik het het liefst op een werkoverleg waar ze allemaal zitten. Er is er altijd wel een niet of twee niet. Als ik alle communicatie daar moet neerleggen krijg je dus de one woman show en zittende medewerkers. Dus ik doe nu zo dat in week van het overleg, stuur ik aan het begin van de week een mail met allemaal punten. Dan weten ze, als ze er vragen over hebben, dat is het begin van het overleg, waarna ze hun eigen zaken kunnen bespreken. Dus dan zet ik het op de mail om te voorkomen. Soms denk je dat je iets heel duidelijk gecommuniceerd hebt en dan je er achter, vanochtend hadden we hier twee mensen zitten waarvan we dachten 'ja'. Er was een akkefietje geweest en je denkt 'het is goed uitgepraat', maar die communicatie heeft dit of dit of dat gezet. Als je iedereen dezelfde boodschap wil geven moet je of de hele groep of het op papier zetten. Anders wordt het net zo'n doorpraatspelletje. En dan wordt het verhaal heel anders dan dat het was.

**Interviewer:** Je gebruikt dan het werkoverleg, de mail vooraf het werkoverleg. Zijn er ook nog andere manieren hoe je boodschappen...

**Respondent A:** Als het echt een hele belangrijke boodschap is, dan roepen we beneden ook de mensen bij elkaar. Ik ziet niet op een groepsapp of zo. Maar dan roep je de mensen bij elkaar en dan is het even de deur van het secretariaat dicht en 'ik wil even iets meedelen'. Als het echt heel belangrijk, zoals in 2015 kwam iets heel belangrijks, als alle dokters zeggen 'we gaan weg', dat wordt verteld. Maar op dat moment waren er een aantal mensen niet. Dan vraag je ook 'willen jullie nu niet gaan appen'. Ik heb iedereen die dag persoonlijk gebeld zodat iedereen dezelfde dag dezelfde boodschap ontvangt.

**Respondent B:** Je zit met al die parttimers he? Echt Nederland parttime-land.

**Respondent A:** Ik heb op heel >AFDELING<, nu is het dan met die PA's is dat dan, dan heb je maar twee. Ik heb maar een fulltime doktersassistente en een fulltime paramedicus.

**Respondent B:** En bottom-up niet te vergeten he? Communicatie. Dat men iets wil of wat dan ook. Over heel veel dingen beslist >TEAMLEIDER< zelf, maar als het geld kost of als >TEAMLEIDER< twijfelt of het een goed plan is, dan hebben we het er even over. En dan gaat het weer terug.

**Interviewer:** Als we kijken naar jou beslissingsbevoegdheid, waarover zou jij allemaal beslissingen mogen nemen?

**Respondent A:** Waarover zou ik beslissingen mogen nemen? Nou, aankomen tot 1.000 euro. Dat is heel pragmatisch en heel aantoonbaar. In ieder geval over de hele planning. De planning van de artsen en de paramedici. Of nou beslissen, ik ga niet met >BEDRIJFSLEIDER< overleggen van 'goh'...

**Respondent B:** Die gaat extra werken...

**Respondent A:** Gewoon inzet van personeel. Wij werken met een detacheringsbureau of ZZP'ers voor de paramedici en het flexburo eventueel voor de inzet van assistentes en polikliniekdokters. Als ik vind dat het nodig is, ga ik het inzetten. Daar heb ik wel volledige beslissingsbevoegdheid over. En weetje over wijzigingen in. Over. Niet zomaar in uren. Als iemand minder wil werken wel. Dat moet je ook altijd wel honoreren. Dat overleggen we natuurlijk wel. Als iemand zegt 'ik wil van drie naar vier dagen'. Dan moet ik wel naar >BEDRIJFSLEIDER< gaan van 'hebben we nog ruimte dat we kunnen uitbreiden?'. Als iemand vraagt 'ik wil in plaats van maandag dinsdag werken', dat soort dingen worden gewoon geregeld. En verder.

**Respondent B:** Operationeel eigenlijk alles.

**Respondent A:** Operationeel eigenlijk heel veel.

**Interviewer:** Bijvoorbeeld opleidingen. Je hoort vaak dat er een opleidingsplan wordt gemaakt. Ben jij degene die dat opleidingsplan maakt?

**Respondent A:** Eigenlijk is er wel een verschil. Alle paramedici staan allemaal in een kwaliteitsregister. Die moeten verplicht punten halen bij hun nascholing. En die dienen eigenlijk allemaal, dat zit zo ingesleten, hun plan in van 'ik wil dit, dit, dit en dit'. Voor dokters een polikliniekassistentes is dat toch een ander verhaal. Er is minder scholing. Alle scholing die aangeboden wordt door het ziekenhuis in het LMS promoot ik bij hun en ga ik ook niet bij >BEDRIJFSLEIDER< van 'goh, er wilde een de training werkbegeleiding'.

**Interviewer:** Maar mocht er iemand een externe training willen volgen?

**Respondent A:** Een externe training. Dan zouden we moeten kijken. Want dat gaat van het opleidingsbudget dus dat ga ik wel met >BEDRIJFSLEIDER< overleggen. Meestal overleg ik dan al van 'die wil dit'. Ik vind er soms wat van. Soms vind ik het een heel goed plan en soms denk je ook van 'is dat wel zinnig?' en ik zie dit of dit en verwacht dat. Ik moet dat wel met >BEDRIJFSLEIDER< bespreken en dan koppel ik dat aan de medewerker terug.

**Respondent B:** Dingen die uit de jaargesprekken komen die bespreken wij altijd wel.

**Respondent A:** Dingen die uit het jaargesprek komen ja.

**Respondent B:** En dat is. Als je het hebt over opleidingsplan, dan is zeker bij die dokters en polikliniekassistentes, dan ben je al lang blij dat je ze iets kunt bieden. En dat gaat relatief over weinig geld want er zijn weinig mogelijkheden. Dus dan kan het ook altijd wel.

**Respondent A:** Kijk zo'n VOVZ-dag dat is extern een dag. Dat is een scholing. Dat is een van de weinige dagen waar hun heen kan. Ik meld dat dan wel bij **﻿**>BEDRIJFSLEIDER< van 'ik heb gezegd, er mogen er twee'...

**Respondent B:** Oh ja. Er gaan er twee naartoe.

**Respondent A:** Er gaan er twee heen. Dat ga ik niet vooraf uitgebreid toestemming voor halen. Wetende dat.

**Interviewer:** Je zei net al dat er een klein stukje financiële bevoegdheid bij jou ligt. Tot die 1.000 euro.

**Respondent A:** 1.000 euro.

**Interviewer:** Zijn dat dan voornamelijk materialen die je koopt, of zijn het ook wel eens dingen zoals een bloemetje...

**Respondent A:** Een bloemetje. *﻿Respondent lacht*﻿.

**Interviewer:** Bijvoorbeeld dus geld die je in het personeel investeert?

**Respondent A:** Dat kan dus inderdaad zijn, bloemen voor een medewerker die ziek is. Bloemen voor een medewerker die een diploma heeft behaald. Als ik oor alle bloemen ging overleggen, dan kunnen we per week nog wel een extra week inlassen denk ik. Daar stellen we eigenlijk heel breed. Dat kan materiaal zijn, maar dat kan inderdaad ook iets van personeel. Nou de scholing, omdat dat dat anders is dan die 1.000 euro waar ik voor bevoegd ben, scholing is iets anders, wat ik net aangaf. Als er iets op de poli moet komen voor het algemeen belang, dat het echt materiaal is dat nodig is in patiëntenbelang.

**Interviewer:** Wordt er vaak geld uitgegeven aan dat individuele belang?

**Respondent A:** Ik denk dat wij daar van >AFDELING< best...

**Respondent B:** Als er iets te vieren is? Dan vieren we dat.

**Interviewer:** Zijn er nog andere voorbeelden dan dat bloemetje?

**Respondent A:** Een stagiaire die weg gaat en een cadeautje krijgt. Nou dan zeggen we 'degene die begeleidt heeft zegt dan 'die zorgt voor een bloemetje'. Als het een stagiaire is geweest die het goed gedaan heeft heb je ook voordelen van. Stagiaires zijn je potentiele collega's. Dan zeggen we ook van 'een cadeautje een aardigheidje'. Dat kan een cadeaubon, een tijdschriftenbon, dat kan een bon van de Rituals zijn.

**Respondent B:** De jubileumtaart he?

**Respondent A:** Jublieumtaart met foto. Bloemen voor een nieuwe medewerker. Dat is wel een HR dingetje op het inwerkformulier nieuwe medewerker. Maar is bijna een poli die dat doet.

**Respondent B:** Wij wel.

**Respondent A:** Wij doen dat wel.

**Respondent B:** Dat wordt echt gewaardeerd.

**Respondent A:** Dat doe ik op >AFDELING<.

**Interviewer:** Dat doe je op eigen houtje? Dat verzorg je allemaal op eigen houtje?

**Respondent A:** Dat regel ik gewoon. Ik heb op >AFDELING< ook een bedrijfsleider. Dus dan meld ik het gewoon, als ik het dan meld, van 'uh, ik heb dit dat dat, de accounts aangevraagd, bloemen voor de eerste werkdag'. Bloemen voor de eerste werkdag? Ja. Bloemen voor de eerste werkdag. Dat zijn de eerste punten die we verdienen, de eerste credits die we hebben.

**Interviewer:** Wat maakt naar jou een goede teamleider, wanneer levert een teamleider goed werk?

**Respondent A:** Allemachtig. Ik vind dat wel een beetje een gewetensvraag. Wanneer ben je een goede teamleider. Ik vind een goede teamleider. Vanavond weet ik het heel goed he? Dat je een goede teamleider bent als je je team zelf de ruimte geeft om dingen te ontdekken, om misschien een keer op je pet te gaan, maar wel een hand reiken om op te staan. Je resultaten toch halen. Op de afdeling toch zorgen dat je met je team het goed kunt verantwoorden naar EVR-leiding. Dus naar bedrijfsleider, dus naar vakgroepvoorzitter, naar de leiding, wat je doet. Dus of dat nou het beste is, als het maar goed kunt verantwoorden. Dus eigenlijk kunt verantwoorden wat je doet en waar je voor staat en dat je het team zo zelfstandig...

**Respondent B:** Laat ontwikkelen he? En dat je de randvoorwaarden van kwaliteit en veiligheid goed in de gaten houdt. Met die mini-veiligheidsrondes, met die audit van hygiëne en infectiepreventie en wat voor instrumenten we dan ook allemaal hebben. En dus die laten we dus ook zo veel mogelijk zelf doen. Zoals die mini-veiligheidsronde en wel meekijken over de schouder en af en toe een buitenstaander uitnodigen om mee te kijken. Om te zorgen dat de kwaliteit van zorg goed op orde is.

**Respondent A:** En ik denk dat je als teamleider, maar ik denk dat je dat op elke leiding, dat je hard op de feiten moet zijn, maar zacht op de persoon.

**Interviewer:** Dus echt een harde kan en een zachte kant?

**Respondent A:** Ja. Dat vind ik niet altijd makkelijk hoor en dat gaat niet altijd vlekkeloos. Dat is wel iets dat ik zou willen en dat in mijn ogen ook wel moet zijn.

**Interviewer:** Heb je het idee dat je ook nog bepaalde kennis nodig hebt als teamleider om goed te kunnen functioneren?

**Respondent A:** Ja.

**Interviewer:** Wat moet je weten?

**Respondent A:** Ik vind sowieso als teamleider, omdat je operationeel, en dat vind ik het grote onderscheid met bedrijfsleider, als teamleider moet je vakkennis hebben. Ik merk, ik heb geen >AFDELING<-kennis en ik ben maar voor een heel klein deel op >AFDELING<, daar kan ik me wel redden. En toch heb ik het gevoel dat ik altijd een buitenstaander voor mijn gevoel blijf en ik vind dat je als teamleider, omdat er operationele beslissingen worden gevraagd, operationeel moet je dingen kunnen kanaliseren, kristalliseren, dan vind ik vakkennis wel heel belangrijk. Dat is wel de eerste kennis. Dat je daarnaast kennis hebt van allerhand managementmodellen, allerhand gesprekstechnieken. Ja. Dat is ook wel heel prettig, ik vind ook dat je al teamleider vakkennis, zodat ze je nooit een oor aan kunnen naaien. Of dat je denkt 'ik trap ergens in, ik doe het goed'.

**Respondent B:** Ik ben het daar niet mee eens. Ik ben het daar niet mee eens.

**Respondent A:** Nou ja dat is leuk?

**Interviewer:** Waarom ben je het er niet mee eens?

**Respondent B:** Ik ben teamleider geweest bij de >AFDELING<, met geen enkele vakinhoudelijke kennis. Ik ben ook denk ik wat naïef, ik vertrouw mensen altijd. Dus ze kunnen je een keer een oor aannaaien. Maar als ik er achter kom dan heb je toch echt een dijk van een probleem. Die heeft het zich bij mij in een andere richting ontwikkeld. Ik vind op een gegeven moment wel, ik zou als polikliniekassistente taken kunnen doen als ik daar teamleider gebleven was. Ik heb toen ook ooit een SAP-training gevolgd. Dat je afspraken kunt maken, dat je aan de balie kunt zitten. Dat is ook meewerken. Ik denk dus ook dat een teamleider kan functioneren als hij een groot organisatorisch vermogen heeft en bereid is zich te verdiepen in het vak. Ik moet er wel een kanttekening bij plaatsen want >AFDELING< is behoorlijk complex zeg maar qua substitutie van taken en hoe dingen aan elkaar gelinkt zijn. Daar is het dus echt wat lastiger. Dan is het wel heel handig als je iemand heb met inhoudelijke kennis. Je moet wel een bepaalde affiniteit hebben met het vak en als je zelf uit het vak komt, dan weet je dat al. Toen ik hier kwam als teamleider bij de >AFDELING<, ik had geen idee. Ik dacht het wel, nou ik heb een affiniteit met zorg, dan kan ik ook affiniteit krijgen met de >AFDELING<. Als dat niet het geval is, dan is het dus een stuk lastiger.

**Respondent A:** Op >AFDELING< heb ik die kennis niet. Dan vind ik het wel lastiger. Ik kan me wel redden. Ik kan wel jaargesprekken voeren. Ik kan wel operationeel, als er zaken zijn en het team is er ook wat vakkennis betreft en alles wat er moet gebeuren. Misschien ben ik dan gewoon te veel gelinkt aan Oog. Op Oog vind ik als teamleider, maar ook door alles wat wij meegemaakt hebben, zonder vakkennis denk ik dat je het wel heel zwaar zult hebben gehad en dat er dan...

**Respondent B:** Dan zou ik het met name een heel stuk zwaarder hebben gehad. Dat ja. Ik denk dat het goed mogelijk is dat jij als teamleider, maar dan moet je je wel verdiepen. En moet je dus bereid zijn om je te verdiepen in het vak.

**Interviewer:** Is het ook heel duidelijk voor jullie hoe de verantwoordelijkheden zijn verdeeld? Wie doet wat?

**Respondent B:** Voor mij wel.

**Respondent A:** Voor mij ook. Er was wel een tijdje dat ik had 'ik weet het even niet meer'. Toen hebben we het er over gehad en toen hebben we het opeens weer. Het staat ook op papier. Mijn functieomschrijving, wat bij mij hoort. Die hebben we toen nog min of meer samen doorgelezen voordat we hem ingediend hebben.

**Respondent B:** Ja.

En toen we dat op een rijtje hadden van 'ja'. Dat is ook wel voor mij...

**Interviewer:** Jouw functieprofiel is voor jou, mocht er ooit weer een moment komen dat je denkt van 'goh, zou het bij mij horen of niet', dan is jouw functieprofiel een goede houvast?

**Respondent A:** Nou. Ik moet je wel heel eerlijk zeggen dat ik dan denk 'hoort dit bij mij?', dan denk ik ook heel vaak 'jeetje, >BEDRIJFSLEIDER< heeft het harstikke druk, ik weet niet of het bij mij hoort, maar als ik er tijd voor heb dan kan ik er in ieder geval aan beginnen'. Dat gebeurt dan eigenlijk best wel vaak. Ik ga niet mijn functieprofiel helemaal uitspitten. Ik behoor niet tot de groep. Als ik het niet weet of het bij mij hoort, maar ik weet wel waar het over gaat, of ik kan daar een stap in zetten, dan ga ik het doen.

**Interviewer:** Een stukje eigen initiatief?

**Respondent A:** Het lijkt met niet dat je moet gaan zitten wachten tot.

**Respondent B:** Ik kom dus zeg maar uit het operationele niveau van het moment dat je overal nog seniors had, dus voor mij was het wel een stap, en soms nog wel hoor, om gewoon los te laten. Want ik hou heel erg van, soms ben ik er ook wel klaar mee hoor, van het regelen en het crisisjes oplossen. Maar goed ik ben, wordt nu toch geacht dat ik de jaarverslagen, alle verslaglegging, rapportages naar de Raad van Bestuur, de begroting. Nu bezig met het meerjarenbeleidsplan voor de >AFDELING< in te vullen. Business cases te schrijven en zo.

**Interviewer:** Dit zijn echt bedrijfsleiderstaken?

**Respondent B:** Ja.

**Interviewer:** Heb jij daar nog een rol in?

**Respondent A:** Sorry?

**Interviewer:** Heb jij nog een rol in de taken die >BEDRIJFSLEIDER< net noemde?

**Respondent A:** Met name in het meerjarenbeleidsplan, of nou een rol. Maar daarover sparren we nog wel eens of vraag je eens of zeg je wat. Ik heb wel altijd aangegeven, maar daar kwam het niet altijd van, dat ik daar bij betrokken wil zijn. Ik ben zelf wel ambitieus. Ik vind het gewoon wel leuk. En voor nu ook voor >AFDELING< ook heel spannend omdat we weer aan het bouwen zijn met een heel nieuw team. Om ook te kijken van 'hoe gaat dat, waar staan we nu, wat wordt dat meerjarenbeleidsplan, waar gaan we naartoe werken?', want niets was meer zeker. Dus ja, een rol. Je hoopt het en >BEDRIJFSLEIDER< geeft mij dat stukje rol ook wel.

**Respondent B:** Ik laat alles checken door >TEAMLEIDER< vanwege >TEAMLEIDER< inhoudelijke kennis. PA's, die leest >TEAMLEIDER< eerst voordat die naar de raad van bestuur gaan. Alle investeringsaanvragen of wat dan ook, zeg dat zij overvolle kennis heeft is voor mij een groot gemak. Ik hoef er niet mee naar een ander of naar een dokter.

**Interviewer:** Handig?

**Respondent A:** Ja.

**Interviewer:** Is er naar jouw idee ook nog een bepaalde manier hoe, en dan voornamelijk kijkend naar die HR-taken, hoe dit uitgevoerd moeten worden?

**Respondent A:** Hoe bedoel je je vraag?

**Interviewer:** Je krijgt allemaal HR-taken als je naar je functieprofiel kijkt. Was het toen voor jou ook helemaal duidelijk wat er van jou verwacht werd, hoe je dat moest invullen?

**Respondent A:** Een aantal taken, zoals op mijn vorige werkplek, heb ik jaargesprekken en verzuimgesprekken. Dat was wel een ander format. Dus ik, met dat soort dingen, ik ben niet bij jou aangehaakt. We kwamen er eigenlijk achter dat de jaargesprekken zoals jij ze voerde, was ook zo als op mijn vorige werkplek. Een beetje zo gewend. Dat was voor mij vrij snel duidelijk. Eerder de systemen. Zoals in huis hebben we ook nieuwe systemen gekregen dus opeens is er een nieuw systeem voor verzuim. Dan heb ik zoiets van 'uh'. Voor >BEDRIJFSLEIDER< was het ook niet duidelijk, dus zijn we maar samen gaan kijken en zoeken van 'waar zetten we dit, hoe doen we dat'. Als iets niet duidelijk is of hoe ik het moet uitvoeren, of wat. Ik ben teamleider geworden toen >BEDRIJFSLEIDER< als bedrijfsleider geworden was. Dan ga ik naar >BEDRIJFSLEIDER< van 'goh, het is mij niet duidelijk'.

**Respondent B:** Ja. Dat zeker.

**Respondent A:** Dan was het van 'ik denk dat het zo moet, maar heb ik dit wel goed begrepen, vragen ze dit wel van mij?'. Ik heb niet het gevoel dat ik ontzettend knel ben geraakt.

**Respondent B:** Helemaal niet.

**Interviewer:** Maar mocht er dus geen plek zijn waar jij die vragen beantwoord zou krijgen, dan was het een heel stuk lastiger?

**Respondent A:** Ik vind. Als ik het niet bij >BEDRIJFSLEIDER< beantwoord zou krijgen, dan ga ik naar HR of iemand anders. Voor mij is telefoneren vrij lastig met die slechte oren. Maar dat maakt wel dat ik overal kom of denk van 'hm, daar ga ik heen'. Of dat nou is van communicatie, >AFDELING<, HR, Arbo. Ik ga er wel heen.

**Interviewer:** Gebruikmakend van de kennis van anderen?

**Respondent A:** Ik ga altijd vragen 'bij wie moet ik zijn'. Als ik zelfs dat niet weet, ook dat gebeurt wel eens'. Vraag ik ook aan >BEDRIJFSLEIDER< 'wie gaat daar over, wie zou ik daarover kunnen benaderen?'.

**Respondent B:** De dames van HR zijn altijd zeer welwillend. Die hebben een harde kluif gehad aan ons afgelopen jaar met al die waarnemend paramedici en waarnemend oogartsen. Nou niets dan lof.

**Respondent A:** Absoluut. Dat zijn wij ook altijd naar hun. Dat merkte ik ook. Vorig jaar was een roerig jaar. We gingen aan het eind van het jaar een presentje brengen voor alles wat ze voor ons dus steeds weer. Het is een wisselwerking. Als je begrip hebt voor hun, zij zijn naar ons toe alle welwillend, ik stap daar ook met alles binnen zo van 'hallo, daar ben ik weer. Ik heb nu een graag, bij wie moet ik zijn?'. En het komt altijd goed.

**Interviewer:** Heb je als teamleider het idee dat je nog bepaalde vaste verplichtingen hebt?

**Respondent A:** Vaste verplichtingen? Dan moet je me even helpen wat je daarmee...

**Interviewer:** Zoals die jaargesprekken, dat zijn best wel een moetje. Iedere medewerker heeft recht op een jaargesprek ieder jaar. Dat klinkt iets wat er gewoon standaard bij hoort?

**Respondent B:** Super leuk.

**Respondent A:** Maar dat is juist een van de leukste dingen. Het jaargesprek is juist het moment om eens even rustig met iemand te zitten en te praten. Soms hoor je dan dingen waarvan je denkt 'goh, had ik nooit achter diegene gezocht' of 'he, nu snap ik waarom het afgelopen twee maanden niet zo lekker loopt'. Ik voel het niet. Kijk, het moet gebeuren, je moet gewoon, je jaargesprekken moeten gevoerd worden. Er moet ook elke dag poli gedraaid, er moet ook de elke de dag planning kloppen. Ik voel dat niet als een verplichting, zeker niet de jaargesprekken.

**Interviewer:** Zeker. Het is meer gericht op dat het iets is dat standaard terugkomt. Als jee een soort draaiboek zou maken, dan het jaargesprek wel iets dat daarin thuis hoort. Zijn er nog meer van dat soort voorbeelden?

**Respondent B:** Verzuimgesprekken.

**Respondent A:** Ja. Frequent-verzuimgesprekken.

**Respondent B:** Werkoverleg.

**Respondent A:** Ja. Voortgangsgesprekken die daar weer bij horen. Dat soort gesprekken komt altijd terug. Verder als je. Ieder jaar moet weer je veiligheidrondes gelopen zijn. Onze auditcertificaten, dat is niet elk jaar maar...

**Respondent B:** Om de zoveel jaar.

**Respondent A:** Er zijn gewoon een heleboel dingen die elk jaar weer terugkomen. Je eigen BLS-training, je reanimatietraining, die komt ook ieder jaar weer terug.

**Interviewer:** Het is goed duidelijk voor jou wanneer dat er allemaal weer aan zit te komen. Heb je daar overzicht over?

**Respondent A:** Ik weet bijvoorbeeld. En altijd gaat dat even in mijn nek hijgen. De jaargesprekken beginnen bij mij rond mei. Ik weet gewoon 'voor 31 december moet ik ze gewoon allemaal gevoerd hebben'. Dat lukt.

**Respondent B:** Je hebt er zelf invloed op. Dus als je het wil veranderen...

**Respondent A:** Soms trek ik er een naar voren. Ik moet er nu een, dat is dan in zo'n cyclus gepland, dat kwam nu op tweede paasdag uit, dat is niet zo handig, haal ik wat naar voren. Vind ik ook wel lekker. Vooral dit jaar heb ik het bewust. Twee jaar geleden dacht ik al 'ik moet dit anders gaan plannen, nog meer verdelen'. Dit jaar ben ik nu wel blij dat ik dat nog niet gedaan heb omdat op >AFDELING< nog zoveel andere dingen eerst even aandacht. Dan denk ik 'dan is de eerste vier vijf maanden voorbij met die dokters en gaan we met die jaargesprekken'. Je weet dat het komt. De frequent-verzuimgesprekken. Ja. Je weet niet aan het begin van het jaar 'oh, die ga ik dan voeren', want je weet niet wat het brengt.

**Interviewer:** Maar eenmaal in zo'n traject, dan kun je wel enigszins vooruit plannen van 'dan kan ik dat'?

**Respondent A:** Dan weet je ook als iemand langdurig ziek is ook, weet je, dan is die zes weken ziek, dan moet dit en dan moet dat. Dat is wel helder hoor.

**Interviewer:** Ik ben er bijna hoor.

*﻿Respondent 1 lacht*﻿.

**Interviewer:** Heb je het idee dat een teamleider nog bepaald gedrag uit moet stralen.

**Respondent A:** In ieder geval vind ik dat je als teamleider, of gedrag, wel moet conformeren aan de richtlijnen die het ziekenhuis stelt over bijvoorbeeld kleding waar ik, als ik geen spreekuur doe, heb ik sieraden om. Als ik spreekuur doe is het eerste wat ik doe sieraden hier af doen en een witte jas aan, witte jas dicht. Dat. In bejegening. Communicatie en bejegening. Ik vind het een naar woord voorbeeldgedrag.

**Respondent B:** Ja. Maar dat is wel zo. Teamleider heeft een hele grote voorbeeldfunctie. Een bedrijfsleider ook. Heiliger dan de paus. En als je iets doet wat niet mag, zorg dat niemand het ziet. Ja. Dat is hier gewoon de conclusie.

**Respondent A:** Ik vind het zo'n naar woord voorbeeldfunctie.

**Interviewer:** Wat maakt het naar voor jou?

**Respondent A:** Nou. Wat maakt het naar voor je? Het is niet zo dat ik beter ben of weet hoe het hoort. Je weet wel wat het huis, wat er van je verwacht wordt, wat de richtlijnen zijn. En je ziet dat mensen toch de hand wat mee lichten. En daar moet je soms toch. Dat zijn hele simpele dingen. Een spijker moet scheuren. Iemand die spreekuurhoudend is een spijkerbroek met van die scheuren draagt, kan die nog zo'n dure merkbroek zijn, hoort niet. Voor mij betekent dat, stond ik vanochtend he >BEDRIJFSLEIDER<?, stond ik van 'kan dit eigenlijk wel?'. *﻿Respondent wijs naar gedragen kledingstuk*﻿. Ik ben dan ook voor de medewerkers op de poli een voorbeeld in. Niet met je boterham in de hand over de poli lopen. Hele simpele dingen.

**Respondent B:** Ben ik helemaal met je eens.

**Interviewer:** En bepaalde dingen die je als teamleider nog moet kunnen, competenties die je zou moeten hebben?

**Respondent A:** Wat moet je hebben. Dat je toch wel enig organisatietalent. Dat je moet kunnen organiseren. Dat je goed moet kunnen luisteren. Er komt ontzettend veel op je af. Luisteren met name ook leren luisteren wat er onder ligt en wat ze je vertellen. Dat is het leren filteren. Er komt iemand vertellen dat hij een naar weekend heeft gehad, niet goed geslapen heeft, of dat het spreekuur niet lekker liep. En dan zit daar een verhaal achter omdat er iets gebeurt is, omdat er iets niet loopt. Dat bedoel ik luisteren. Niet alleen zoals jij nu naar ons luistert, maar ook...

**Interviewer:** Net een laagje dieper kunnen gaan?

**Respondent A:** Ja. Dat vind ik eigenlijk wel dat je moet kunnen. Je werk organiseren. Je moet kunnen plannen, prioriteiten stellen, flexibel zijn en schakelen. Dat vind ik ook wel heel belangrijk. Ik heb zo vaak dat ik me voorneem 'dit, dit en dit ga ik vandaag regelen of afhandelen vandaag', weet je dan gaan er tien brandjes 'daar een zieke, daar is iets gebeurt'. Schakelen tussen dingen.

**Respondent B:** Daarnaast moet je nog al een motivator zijn he? Mensen motiveren. Je bent ook een voorbeeld in energieniveau en werklust en hoe leuk je het vindt, ook qua uitstraling. Je moet een bezielend leider zijn.

**Respondent A:** De betrokken en bevlogenheid uitstralen. Ja.

**Interviewer:** Dan zit ik er eigenlijk wel doorheen. Ik heb jullie gevraagd wat ik jullie wilde vragen. Misschien als laatste vraag nog, zien jullie veel verschillen hier tussen teamleiders in huis?

**Respondent B:** Dat kan ik niet beoordelen.

**Respondent A:** Ik, wij hebben een tijdje teamleiders. Iemand heeft dat opgezet. De bedrijfsleiders hebben bedrijfsleidersoverleg. Dat heeft ook een status in huis. Toen heeft iemand gezegd van 'goh, een teamleidersoverleg om onderling eens te sparren', dat had geen status, en eens te kijken 'waar lopen we tegen aan'. En dan merk je wel, er zit wel verschil in teamleiders en dat heeft enerzijds ook te maken dat we allemaal andere mensen zijn. Dat is overal dat is prima. Merken een duidelijk verschil tussen die teamleiders alleen in de kliniek, dus >AFDELING<en, polikliniek, en daarnaast heb je ook nog een ploeg teamleiders zoals de teamleiders >AFDELING<, teamleider van de voeding, teamleider van de >AFDELING<t en dat is niet anders, maar er zitten wel verschillen in. Als ik het. Ik heb het met de teamleider van de Voeding, waar ik ook te maken mee heb. Als je jaargesprekken moet voeren met mensen die echt niet in staat zijn om op een computer een afspraak te accepteren of eigenlijk niet in staat zijn het jaargesprekformulier te lezen, dat maakt...

**Respondent B:** Ja. Dat is echt wel andere koek.

**Respondent A:** Ik weet nog. Er was eens een teamleider, dat zeg ik heel plat, nou ik snap niet wat die teamleider bij ons aan moeten schuiven? En dan bedoelde men vooral de teamleiders van alle faciliterende, ondersteunende diensten die niet rechtstreeks in de zorg. Dus van de niet-zorg afdelingen. Nou doe je petje er maar voor af hoor. Je zult maar 60 mensen van de >AFDELING< aansturen die amper Nederlands spreken of. Petje af. Er zit dus wel verschil tussen teamleiders.

**Respondent B:** Ja. In huis zitten er verschillen in. Ik zat naar mijn eigen teamleiders te kijken. Die teamleiders, dat zijn er maar twee. Een teamleider in opleiding noem ik dat maar, maar dat zijn hele verschillende mensen. Ook heel verschillend in hoe lang ze teamleider zijn. In aanpak en hoe ze dingen oppakken en wat belangrijk is en qua taakopvatting. Er zijn ook heel veel overeenkomsten?

**Interviewer:** Dus het feit dat we zelfde functieomschrijving gebruiken voor alle EVR teamleiders, is dat naar jullie idee oké?

**Respondent B:** Ik kan dat alleen maar voor mijn eigen poli's kan ik dat beoordelen. Ik vind dat het om de MKA, daar is nog geen teamleider want die zijn nog wat te klein. Maar als ik kijk naar jou en naar >TEAMLEIDER<, dan liggen de accenten verschillend vanwege de prioriteiten binnen de club, maar qua indeling van het werk en qua taakopvatting is het precies hetzelfde.

**Respondent A:** Ja. Ik vraag me af. Ik zat net te denken. Je kunt op Idoc staan alle functieomschrijvingen via het HR-portal. Er is dus een functieomschrijving teamleider die zo'n beetje door iedereen gebruikt wordt. Dat is de teamleider in de zorg en dergelijke...

**Respondent B:** Teamleider EVR volgens mij.

**Respondent A:** Maar voor de teamleider van bedrijven die faciliteren, hebben volgens mij weer een andere omschrijving.

**Interviewer:** Die zijn op maat gemaakt.

**Respondent B:** Dat snap ik ook wel.

**Interviewer:** Waarom snap je dat?

**Respondent B:** Vakinhoudelijk heeft het te maken met de span of control denk ik, met het type mens dat je aan moet sturen, en het type bedrijf. Het bedrijfsonderdeel zal ik het maar even noemen. Toevallig kom ik van heel lang geleden uit de facilitaire hoek. Ik vind dat in de zorg, met hoogopgeleide professionals, dat is anders. Daar onderscheidt >AFDELING< zich ook heel erg mee vind ik. Ik vind wat dat aangaat ook dat doktersassistenten, zijn ook hoogopgeleide professionals. Naar dat niveau moet je zo ook echt toetrekken. Die doen gewoon heel veel zelf. Het nemen van beslissingen, het afhandelen van een klacht, bijsturen, angstige patiënten. In een >AFDELING< of in een Voeding is dat toch echt anders.

**Interviewer:** Terecht?

**Respondent B:** Ik vind dat de accenten daar anders liggen. En ook denk ik qua controle. Ik ben zelf, dat pretendeer ik ook een heel faciliterend leider te zijn. Een zekere mate van controle is dan wel nodig. En toch is het de verantwoordelijkheid zo laag mogelijk.

**Respondent A:** Ik weet niet of jullie zelf nog dingen willen voegen?

**Respondent B:** Nee. Ik ben blij dat je dit onderzoek doet. Ik vind dat teamleiders de backbone zijn van de organisatie. Ik vind dat we die goed podium moeten geven en goed ruimte om zich te ontwikkelen, daar waar de wens er is en de mogelijkheden er zijn. Ik kijk met belangstelling uit naar de uitslag van je onderzoek.

*Einde transcript*

# Transcript Interview Middle Manager 8

**Duur interview: 33 minuten en 36 seconden**

**Functie: Bedrijfsleider**

**Afdeling(en) onder supervisie: Healthcare**

**Gender: vrouw**

**Werkervaring in functie: n.a.**

**Interviewer:** Om te beginnen. Wat zijn de algemene dagelijkse werkzaamheden van een teamleider hier op de afdeling.

**Respondent:** Ik heb twee teamleiders binnen de afdeling Interne. Een in de polikliniek en een in de kliniek. >TEAMLEIDER< is werkzaam in de kliniek. Daar valt eigenlijk ontzettend veel onder. Alle operationele zaken die aangestuurd moeten worden. Niet alleen van de 28 klinische bedden die we hebben. Ook van de Kurenkamer die we hebben en van de dagbehandeling. Dus dat is best heel vors. Heel veel personeel. Veel leerlingen ook. Arts-assistenten, medisch specialisten. Dus je hebt van allerlei gradaties loop er wat rond. Daarnaast hebben we ook de >AFDELING< en de voedingsassistenten. Die hebben allemaal contact met de patiënt en vallen daarom allemaal wel onder de supervisie van >TEAMLEIDER<. Sommige stuur >TEAMLEIDER< niet direct aan. Maar als er wat is. Bijvoorbeeld, een schoonmaker komt ergens binnen en is ergens van onder de indruk, dan is >TEAMLEIDER< degene die ze eruit vist en gesprek aangaat of zorgt dat ze goed opgevangen worden. Dus >TEAMLEIDER< is voor mij. >TEAMLEIDER< is voor mij mijn oren en ogen. >TEAMLEIDER< ziet veel meer wat er direct op de werkvloer gebeurt en als het nodig is koppelt >TEAMLEIDER< dat terug naar mij. Heel veel kan >TEAMLEIDER<  zelf goed oplossen. Een aantal dingen koppelt >TEAMLEIDER< terug naar mij en spreken we samen 'pak jij dat op of pak ik dat op?'.

**Interviewer:** Heeft u een voorbeeld van dat soort gevallen die terugkoppeling nodig hebben?

**Respondent:** Bijvoorbeeld wanneer iemand. Langdurig verzuim. Slecht functioneren. Ruzie. Wanneer er een gebeurtenis is geweest waardoor iemand niet goed functioneert of daarmee zit toch aandacht nodig heeft om opgevangen te worden. Wij hebben geen hele strakke verdeling van 'dit hoort bij jou, dat hoort bij mij'. Het is veel meer natuurlijk. Dus die dingen waarvan we denken van 'nou'. Bijvoorbeeld >TEAMLEIDER< is heel goed in coachen. Dus als er meer een coachvraag ligt, dan pakt >TEAMLEIDER< dat op. Dat vindt >TEAMLEIDER< ook leuk, daar krijgt >TEAMLEIDER< energie van en >TEAMLEIDER< kan mensen ook echt weer goed op weg helpen daarmee. We hebben de jaargesprekken verdeeld. Hoewel ik wel eerlijk moet zeggen dat >TEAMLEIDER< iets meer jaargesprekken doet dan ik. Maar ik vind het ook heel belangrijk om jaargesprekken te blijven voeren omdat je ook op die manier direct contact hebt met je medewerker en even samen een uur zit en daar ook goede afspraken over maakt. Maar ik merk wel dat er een lading aan hangt, want als je bij >BEDRIJFSLEIDER< moet komen, dan is er misschien wel wat. Die vindt dat soms wel spannender van 'oh, dan heb ik ineens een jaargesprek met de bedrijfsleider in plaats van de teamleider'.

**Interviewer:** Het is geen pragmatische scheiding om de groep letterlijk in tweën te delen?

**Respondent:** Nee.

**Interviewer:** Er zit een reden achter?

**Respondent:** Voor ons een natuurlijke verdeling. Maar ik zeg niet tegen >TEAMLEIDER< 'dit zijn de lastige gesprekken, die doe ik'.

**Interviewer:** Dat is ook niet het geval dan?

**Respondent:** Nee.

**Interviewer:** Eerder de gevallen die echt aandacht nodig hebben?

**Respondent:** Omdat wij denken dat dit een betere verdeling is. Allerlei strategische zaken, als het gaat om financiën, als het gaat om FTE's, als het gaat om materialen. Aanschaf van grote dingen. Dat gaat altijd via mij. Ik vind het wel altijd belangrijk een klankbord te hebben. En dat is voor mij, mijn teamleider. Dus ik stem wel altijd af. Maar uiteindelijk neem ik de eindbeslissing. De eindverantwoording ligt bij mij. Maar ik vind het belangrijk dat je als teamleider een klankbord bent.

**Interviewer:** Een soort adviserende rol dan?

**Respondent:** Ja. Precies.

**Interviewer:** Je noemde net al een paar HR-gerelateerde taken. Ik hoorde jaargesprekken. Coachende rol hoorde ik. Zijn er eigenlijk nog meer dingen die met personeelsmanagement te maken hebben die bij >TEAMLEIDER< liggen?

**Respondent:** Ja. Een heleboel dingen. Iemand meld zich ziek en er moet een dienst opgelost worden. Dat is echt weer heel operationeel. Is de bezetting wel naar behoren voor de volgende dienst. Ook de verdeling zeg maar van verpleegkundigen met patiënten zeg maar zorgzwaarte. Verloop dat een beetje evenredig? Dat zijn dingen. Daar houdt >TEAMLEIDER< zich mee bezig. Wij stemmen wel af met elkaar. >TEAMLEIDER< is er niet altijd. Ik ben er niet altijd. Dus wij dragen het ook moeiteloos naar elkaar over. Dat is ook wel heel belangrijk. Anders gaan we dingen dubbel doen. Dan gaat >TEAMLEIDER< wat regelen bijvoorbeeld.

**Interviewer:** Hoe vindt die afstemming plaats?

**Respondent:** We spreken elkaar dagelijks. Waarin we dingen afstemmen. Van 'nou, wat ligt er. Wat pak jij op, wat pak ik op?'. En als ik een paar dagen vrij ben geweest krijg ik altijd een overdracht van >TEAMLEIDER< en dat doe ik ook naar >TEAMLEIDER< toe. Als >TEAMLEIDER< een paar dagen vrij is geweest of op vakantie is geweest, maak ik altijd even een overdracht met de highlights.

**Interviewer:** En als je dan een paar dagen weg bent, wat is dat de rol van >TEAMLEIDER<?

**Respondent:** Ik heb altijd een collega-bedrijfsleider die mij vervangt. Dus >TEAMLEIDER< heeft geen andere rol als ik weg ben. >TEAMLEIDER< doet exact hetzelfde en weet dat >TEAMLEIDER< moet afstemmen met mijn vervangend bedrijfsleider als het gaat om 'ik loop vast', 'er kan geen flex ingezet worden, maar ik heb ook geen personeel mogen we bedden sluiten?'. Dingen die dus impact hebben op het financiële resultaat. Dus ﻿>TEAMLEIDER< bewaakt wel de kwaliteit van zorg garanderen. En als >TEAMLEIDER< daar gevaar in ziet dan stemt >TEAMLEIDER< met mij of met een vervangend bedrijfsleider.

**Interviewer:** Ik heb eens opgezocht wat een bedrijfsleider als personeelstaken zou kunnen hebben. Daar komt ongeveer zo een lijstje uit. Zouden we eens kunnen kijk welke onderwerpen door >TEAMLEIDER< gedaan worden. En op welke manier dan. En wat dat de rol van >TEAMLEIDER< is.

**Respondent:** Als we kijken naar werving. De vacaturetekst moet ik maken. Dat is omdat ik als bedrijfsleider daar mijn handtekening op moet zetten. Omdat er plek is binnen de begroting of omdat het een uitbreiding is. Dat moet ik verantwoorden. Dus dat doe ik. Selectie kan >TEAMLEIDER< heel goed zelf. Dat stemmen we ook heel goed af van. Bij sommige functies wil ik er graag zelf bij zitten. Bij andere denk ik 'daar vertrouw ik helemaal op. Dat komt helemaal goed'.

**Interviewer:** Dan mag >TEAMLEIDER< dus ook beslissen wie er aangenomen wordt?

**Respondent:** Ja. >TEAMLEIDER< stemt het wel altijd af met mij en kan het wel altijd goed onderbouwen. Ik heb nog nooit meegemaakt dat >TEAMLEIDER< daarin verkeerde beslissingen neemt. Dus daar heb ik absoluut vertrouwen in en dat kan ik loslaten. Introductie kan >TEAMLEIDER< goed zelf doen. Bijhouden van personeelsbestanden dat doen we samen omdat het een hele grote EVR is met heel veel mutaties. Dus dat doen we gezamenlijk. We checken ook met elkaar van 'joh, wat is nu de stand van zaken'. Daar hebben we een Excelbestand voor. Even aanpassen en dan hebben we weer de up-to-date informatie. Beslissen over en plannen van trainings- en ontwikkelingsdoeleinden. Er is een opleidingsbudget. Dat heb ik vastgesteld voor in de begroting. Zo lang daar binnen gebleven wordt heet >TEAMLEIDER< daar wel beslissingsbevoegdheid toe. Zodra we daar overheen moeten gaan, dan stemt >TEAMLEIDER< daar over af. Dat moet goed gemotiveerd. Daar kan >TEAMLEIDER< niet zelfstandig over beslissen.

**Interviewer:** Als ik het dan goed begrijp. Er is iemand die een training nodig heeft. Is >TEAMLEIDER< dan degene die daar 'ja of nee' op zegt? Akkoord of geen akkoord.

**Respondent:** Er zijn standaard trainingen in huis die iedereen volgt. Dan ziet >TEAMLEIDER< er op toe dat iedereen ook echt gaat die dat nodig heeft.

**Interviewer:** En externe training dan?

**Respondent:** Voor externe training, zolang het binnen de mogelijkheden zit. Is het bijvoorbeeld één symposiumdag of zo. Dan mag >TEAMLEIDER< dat zeker beslissen. Als het gaat om een intensieve cursus waardoor iemand een paar weken van de werkvloer is, dan stemt >TEAMLEIDER< dat af met mij. Het geven van formele training dat doet >TEAMLEIDER< en kan >TEAMLEIDER< ook goed. Het geven van informele training. Nou coachen en ondersteunen dat doet >TEAMLEIDER<. Het mentoren dat neemt >TEAMLEIDER< ook van nature op zich. >TEAMLEIDER< vindt het ook leuk om onderwijs te geven en is wat dat aangaat heel laagdrempelig voor medewerkers. Ook omdat >TEAMLEIDER< in het wit loopt en meewerkt. >TEAMLEIDER< springt bij. Coaching on the job.

**Interviewer:** Vind u de verdeling fijn van meewerken en managen? >TEAMLEIDER< heeft een dubbele rol, twee petten. Bent u tevreden met de verdeling zoals die nu is?

**Respondent:** Ik zie dat >TEAMLEIDER< af en toe te veel door de zorg opgeslokt wordt en dat het lastig is. Andere taken blijven dan wel liggen, waardoor >TEAMLEIDER< overloopt.

**Interviewer:** Is >TEAMLEIDER< dat zo'n iemand die dan wel de taken doet ten koste van zichzelf?

**Respondent:** Ja. Dus daar moet >TEAMLEIDER< echt voor waken. Dat heeft hij genoeg inzicht daarin. En >TEAMLEIDER< weet ook dat >TEAMLEIDER< altijd bij mij kan aankloppen, dat ik weer wat taken van zijn bordje haal en dat oppakt als we het aan de voorkant niet beter kunnen regelen. Want het liefst wil ik dat >TEAMLEIDER< dan wat minder meewerkt in de zorg en wat meer met de teamleiderstaken bezig is. En dat is ook wel de spagaat waar een teamleider in zit. Van beide kanten wordt er aan je getrokken. Ik trek aan >TEAMLEIDER< omdat ik wil dat >TEAMLEIDER< dingen doet. De verpleegkundigen, het team, trekt aan >TEAMLEIDER< omdat zij willen dat >TEAMLEIDER< dingen doet.

**Interviewer:** Mocht het te veel worden voor >TEAMLEIDER<, dan zou u voorstander zijn om iets minder zorgtaken te doen?

**Respondent:** Ja. Functioneringsgesprekken. Die doe ik. Beoordelingsgesprekken doen we vaak samen. Of doet hij vaak alleen. Maar als het echt is dat iemand in een functioneringstraject zit is dat vaak van die zwaarte. Je spreekt een periode af van dingen die je moet behalen. Als dat niet lukt, nemen we afscheid van elkaar. Dus dat. Dat ligt niet bij een teamleider vind ik. Dat hoort echt bij een bedrijfsleider. Ik heb wel de input van >TEAMLEIDER< nodig over het functioneren omdat >TEAMLEIDER< veel dichter bij de werkvloer zit dan ik zit. Dus we doen het samen, maar de verantwoording ligt bij mij. Akkoord geven op ontwikkelingsplannen. Dat mag >TEAMLEIDER< zelf en doet >TEAMLEIDER< zelf. Disciplineren en corrigeren doet >TEAMLEIDER< zelf. Nou weet ik niet zo goed wat je onder disciplineren verstaat?

**Interviewer:** Als er echt bijvoorbeeld onwenselijk gedrag vertoond wordt. Dat dat dan rechtgezet wordt.

**Respondent:** Ja. Spreekt >TEAMLEIDER< collega's direct aan en geeft >TEAMLEIDER< aan wat gewenst gedrag is.

**Interviewer:** En indien te extreem, dan kom het hier te recht?

**Respondent:** Ja. Zeker. Afhandelen van tuchtprocedures en klachtprocedures. Afhandelen van tuchtprocedures doen we altijd in samenwerking met de advocaat erbij. Dus dat handelt >TEAMLEIDER< niet zelf af. Klachtenprocedures. We hebben hier een decentrale klachtenafhandeling. Dus een patiënt is ontevreden en die ligt er nog, dan gaat >TEAMLEIDER< daar mee aan de slag. Als wij een klacht krijgen achteraf, dus als de patiënt is al thuis, en de klacht gaat over het operationele proces van 'nou, ik heb niet de zorg gekregen die ik wenselijk vond', dat soort klachten, dan duikt >TEAMLEIDER< daarin en antwoord ook richting de klager toe. En klachten van andere aard handel ik weer af. We kijken dus altijd een beetje van 'wat is de aard van de klacht?'. Als het te maken heeft met het operationele proces, als we daarin te kort zijn geschoten, dan pakt >TEAMLEIDER< dat op. Absentiemanagement dat doet >TEAMLEIDER<. Ziek en herstelmeldingen houdt >TEAMLEIDER< bij, dat doe ik ook. We krijgen ook beide telefoontjes van mensen die ziekteverlof aanvragen. Want dat doen ze hier en wij voeren ook frequent-verzuimgesprekken.

**Interviewer:** Is dat weer per case apart?

**Respondent:** Ja. Per case apart. Met sommige zit je al in een traject en heb je al meerdere gesprekken gehad. Dan is het wat natuurlijker om dat op te pakken dan dat ze weer met anderen om tafel moeten. En met anderen is het voor de eerste keer dat je zo'n gesprek voert en kun je dat prima verdelen met elkaar. Waardering geven doet >TEAMLEIDER< zeker. Beslissen over salarisschalen doe ik. Bottom-up communicatie. Ja. Als je daarmee bedoelt naar het team communiceren, dan hebben wij, of een vertaalslag van de Raad van Bestuur naar het team toe, dat doen we gezamenlijk. We hebben teamvergaderingen, werkbesprekingen, en nieuwsbrief. Dat was ﻿>TEAMLEIDER< initiatief. Om eigenlijk alles wat we in huis aan informatie krijgen om dat te verzamelen en wekelijks door te sturen.

**Interviewer:** Is het ook de rol van >TEAMLEIDER< om informatie van de werkvloer weer naar jou door te spelen?

**Respondent:** Ja. Dat doet >TEAMLEIDER< ook. >TEAMLEIDER< is mijn ogen en oren. Dus wanneer er onrust is, of wanneer er dingen spelen, of een hoge werkdruk is. Dat soort zaken. Dan is >TEAMLEIDER< degene die als eerste bij mij aanklopt en maken wij samen een plan de campagne hoe wij dat samen gaan oplossen. Top-down communicatie, nou daar hebben we het over gehad. Aanhoren van en reageren op suggesties van het personeel, dat doet >TEAMLEIDER< zeker. Werkbesprekingen zitten wij altijd gezamenlijk voor omdat daar de suggesties naar voren komen.

**Interviewer:** Is dit vanwege voorgaande functies van >TEAMLEIDER<?

**Respondent:** Het zit in >TEAMLEIDER< genen. Die vindt het leuk en is daar goed in. En die kan het ook nuanceren. Want er spelen ook beelden bij mensen die ze niet getoetst hebben. Snap je? Dan worden het van die wandelgangverhalen. Dat kan >TEAMLEIDER< heel goed tackelen.

**Interviewer:** Ook fijn voor u dan om zo een teamleider te hebben?

**Respondent:** Ja. Heel fijn. Een paar jaar geleden had ik geen teamleider. En dan probeer je zelf alle ballen in de lucht te houden en dan merk je gewoon dat dat niet lukt. Dan merk je dat het team wat mist. Echt die tussenlaag waar ze op terug kunnen vallen als het druk is, of als het moeilijk gaat met een patiënt. Dan hebben ze een extra handje erbij. Iemand die de rust brengt. En ook iemand waar ze de noden bij kwijt kunnen. Want bij mij moeten ze wachten tot ik tijd heb in mijn agenda. Wat op dat moment dan speelt he.

**Interviewer:** Heeft u ook gebruik gemaakt van seniors?

**Respondent:** Ja. Ik had vier seniors. Maar senior is echt veel meer onderdeel van het team. Vinden het moeilijk om collega's aan te spreken. Krijgen dan ook gelijk terug 'zo hoe je dat niet te zeggen, je hoeft dat nu niet te denken nu je senior bent'. Het team doet ook heel veel moeite om iemand zijn gelijke te maken. En wanneer het te spannend wordt, dan haakt de senior echt af.

**Interviewer:** Dus de teamleider is dan echt een uitkomst?

**Respondent:** Ja. De teamleider staat echt boven het team, maar is wel dichter bij het team dan een bedrijfsleider. Coördineren van teams en ploegen. Dat doet >TEAMLEIDER<. Onderhouden van effectief teamwerk, daar is >TEAMLEIDER< heel goed in. Personeel consulteren en raadplegen doet >TEAMLEIDER<. >TEAMLEIDER< vraagt ook heel vaak wat mensen er van vinden als er ideeën zijn. >TEAMLEIDER< betrekt ze erbij en creëert daarmee gelijk een groter draagvlak. Gezondheid en veiligheid. Ja. Als dingen niet kloppen, dan regelt >TEAMLEIDER< dat. >TEAMLEIDER< is ook echt van het regelen dat is >TEAMLEIDER< degene die dat oppakt. We hebben pas het noro hier gehad. Dan is >TEAMLEIDER< gelijk op de bres en iedereen wordt goed gefaciliteerd zodat iedereen goed met alle veiligheid en isolatiemaatregelen veilig naar binnen kunnen. En >TEAMLEIDER< is heel helder in ﻿de communicatie. Dat vind ik de kracht van >TEAMLEIDER< Daar is geen ruis omheen. Verbeteren van werklevens. Daar zet >TEAMLEIDER< zich enorm voor in. Daar is niet altijd tijd voor. Soms hebben mensen toch wensen waar je niet tegemoet in kunt komen. Ik ben misschien dan iets korter door de bocht en denk 'past niet, kan niet, los het zelf op'. >TEAMLEIDER< gaat dan toch net even wat extra moeite doen. En dat is goed. Want dat betekent dat ze zich gehoord voelen, dat ze zich gezien voelen, zich geen nummer voelen, maar voelen dat iemand die er iets over mag zeggen moeite voor je doet.

**Interviewer:** Als we dan eens kijken naar de bevoegdheden van >TEAMLEIDER<. Die lijken best wel ver te gaan. >TEAMLEIDER< lijkt over best veel dingen te mogen beslissen. Enige idee waar deze bevoegdheid ophoudt en dat we dan hier bij de bedrijfsleider hier uitkomen?

**Respondent:** Die is er natuurlijk wel. Waar ligt hij dan wil jij dan weten.

**Interviewer:** Of welke richting?

**Respondent:** Als het gaat over het financiële deel of wat impact heeft op de financiën, dan moet >TEAMLEIDER< dat altijd afstemmen. Als het gaat om strategie. Als het gaat om het maken van keuzes. Gaat we links af of gaan we rechts af? De input die >TEAMLEIDER< geeft is zeer waardevol en wordt zeer serieus genomen. Maar >TEAMLEIDER< mag nooit de eindbeslisser daarover zijn. Dus ja dat is goed geregeld tussen ons. En als >TEAMLEIDER< een beslissing maakt waarvan ik denk 'dit had je eigenlijk niet mogen nemen'. Want soms he. Dan is de scheidslijn wat minder duidelijk. Dan zal ik >TEAMLEIDER< altijd steunen. En dat vind ik ontzettend belangrijk. >TEAMLEIDER< krijgt namelijk ontzettend veel verantwoording. En >TEAMLEIDER< voelt zicht heel verantwoordelijk, maar dat moet ook wel in een veilige omgeving gebeuren. Als je het gevoelt hebt dat je onmiddellijk een schop onder je kont krijgt als het niet goed gaat, dan doe je dat niet meer. Dan pak je die verantwoording niet meer. Dat vind ik heel belangrijk. En uiteindelijk ja, wat dat betreft is het eigenlijk best griezelig, maar zitten wij zo op een lijn, dat het naar mijn idee nog nooit gebeurt is.

**Interviewer:** Het is altijd wel duidelijk geweest wat van >TEAMLEIDER< verwacht werd?

**Respondent:** Ja. Of we gaan die kant op. Ja dat dacht ik al. Zo. We hebben daar nooit discussie over. Wij staan nooit tegenover elkaar. En dat is wel uniek denk ik.

**Interviewer:** En heel prettig?

**Respondent:** Ja. Zeker.

**Interviewer:** Als we dan een kijk. U zei net al iets over financiën. Stel >TEAMLEIDER< wil een bos bloemen kopen voor iemand van het personeel...

**Respondent:** Dat mag >TEAMLEIDER< altijd doen.

**Interviewer:** Dat kan ook zonder toestemming?

**Respondent:** Nee. Kan zeker zonder toestemming. >TEAMLEIDER< vertelt het altijd wel van 'ik heb daar en daar een bloemetje laten bezorgen'. Dan zeg ik van 'oh, fijn dat je er aan gedacht hebt'. Want >TEAMLEIDER< is daar attenter in en weet sneller dat er wat speelt en denk ik ook van 'daar moeten ook bloementjes naar toe'.

**Interviewer:** Er ligt dus wel een kleine financiële verantwoordelijkheid bij >TEAMLEIDER<. >TEAMLEIDER< mag wel iets van kleine aankopen doen.

**Respondent:** Ja. >TEAMLEIDER< mag van mij ook zeggen dat een dienst naar TMI moet. TMI is natuurlijk veel duurder qua personeel. Maar ik vind dat je wanneer je verantwoordelijk bent voor de kwaliteit van zorg, dat je ook die beslissing kunt nemen. Als je het maar goed uit kunt leggen. Op het moment dat ik denk 'het wordt te pas en te onpas gedaan om het team te pleasen'. He, er wordt heel snel geroepen dat er een hoge werkdruk is. Ja, dan ben je populair als teamleider. Dan zou ik het niet doen. Maar die verantwoordelijkheid heb ik doorgeschoven. >TEAMLEIDER< mag dat doen. >TEAMLEIDER< geeft ook altijd terugkoppeling naar mij waarom >TEAMLEIDER< dat heeft gedaan. En dan had ik dat ook gedaan.

**Interviewer:** Zijn er nog meer aankopen die >TEAMLEIDER< zou mogen of kunnen doen zonder jouw tussenkomst?

**Respondent:** Ja. Kijk. Als meubilair stuk is dan mag >TEAMLEIDER< dat gewoon aanschaffen en vervangen. Mensen moeten hun werk kunnen doen. Dus als apparaten stuk gaan moet dat vervangen worden. Wat dat betreft is >TEAMLEIDER< ook best zuinig dus neemt ook geen gekke beslissingen daarover. De grote uitgaven mag ﻿>TEAMLEIDER< niet zelf tekenen. Dan schrijft >TEAMLEIDER< wel de bestelbon kant-en-klaar voor mij. Dan moet ik tekenen. Dat is natuurlijk helemaal helder. Dan doe dat.

**Interviewer:** Misschien voor jullie heel vanzelfsprekend, maar wanneer ben je tevreden met >TEAMLEIDER< functioneren.

**Respondent:** Ik weet niet of ﻿>TEAMLEIDER< jou dat verteld heeft, maar >TEAMLEIDER< heeft van mij een boven-verwachting beoordeling gekregen op het jaargesprek. Dat komt omdat >TEAMLEIDER< zo meedenkt en zo faciliterend is en zoveel rust brengt in het team en ook overstijgend mee kan denken. De senior is onderdeel van het team en die heeft ook last van die hoge werkdruk. Dus die reageer vanuit andere waarden die op dat moment spelen. >TEAMLEIDER< kan dat ook doen met de bril op van het organisatiebelang. En dat vind ik wel heel belangrijk van een teamleider dat. Soms zijn dingen spannend en kun je daar ook niet volledig achter staan. Maar snap je we dat er een organisatiebelang is waarom je dit moet doen. En dan moet je het uit kunnen leggen aan je medewerkers en erachter kunnen staan. En dat vind ik heel belangrijk dat bij een teamleider moet passen. Een teamleider moet geen allermansvriendje zijn. Die heeft toch een functie in de hierarchie. Dat betekent dat mensen soms ook boos op je zijn en niet zo leuk vinden dat ze een gesprek met je hebben. Dat moet je wel aan durven gaan.

**Interviewer:** Denk je dat er ook nog bepaalde kennis is die >TEAMLEIDER< als teamleider bij zich zou moeten dragen om het werk van teamleider goed te kunnen doen?

**Respondent:** Ja. Zeker. Wat dat betreft heeft >TEAMLEIDER< ook een managementopleiding gedaan. Dat rugzakje van >TEAMLEIDER< is dan ook goed gevuld om de functie te kunnen vervullen. Ik zie in huis wel teamleiders die dat wat minder mee hebben.

**Interviewer:** Dat zou een goede overweging zijn voor andere teamleiders om ook te doen?

**Respondent:** Zeker. Wat ik de kracht vind van >TEAMLEIDER< is dat >TEAMLEIDER< en veel kennis heeft van de zorg, op het moment dat spannend wordt dat >TEAMLEIDER< kan bijspringen en rust kan brengen, maar hij heeft ook veel verstand van HR-zaken. En dat is een groot gedeelte van je functie als teamleider. En >TEAMLEIDER< heeft het verstand om mensen coachen.

**Interviewer:** En verstand van HR-zaken over wat voor verstand zou ik dan kunnen denken?

**Respondent:** Over, binnen welke marges kun je bewegen. Wet en regelgeving. Hoe vlieg je bepaalde zaken aan? Waar kun je bepaalde formulieren vandaan toveren die nodig zijn voor een frequent-verzuimgesprek of voor een jaargesprek. Of dat soort zaken. Daar is >TEAMLEIDER< heel goed van op de hoogte. En volgt dat altijd goed op. En wat dat betreft is dat altijd goed in orde. En >TEAMLEIDER< is volledig zijn verslaglegging en die is duidelijk. Dus het is voor mee volledig duidelijk waar >TEAMLEIDER< mee bezig is. Een dat stukje inzage daarin, dat stukje transparantie, ook al werk je niet heel veel samen, we hebben natuurlijk wel spreekmomenten met elkaar maar werkt ook een groot deel van de dag zelfstandig, dat hoor ook zo. Een van de kwaliteiten van teamleider vind ik ook dat je die niet heel direct hoeft aan te sturen. Je moet echt zelfstandig kunnen werken. Dus ja weet je. Het is helder. Ik weet waar >TEAMLEIDER< mee bezig is. En dat vind ik heel belangrijk.

**Interviewer:** En als we dan eens kijken naar uw visie op de teamleider. Wat is naar uw idee de verantwoordelijkheid van een teamleider en wat overstijgt een teamleider? Dat hoort bijvoorbeeld bij iemand anders. Bijvoorbeeld een bedrijfsleider.

**Respondent:** Nou, we hebben net het lijstje doorgenomen. Wat hoort bij een teamleider. Een bedrijfsleider is eindverantwoordelijk voor al deze zaken. Dus wat bij mij hoort is bewaken dat >TEAMLEIDER< dit goed kan en goed gefaciliteerd wordt hierin. Wat bij hoort is bewaken dat >TEAMLEIDER< goed de functie kan uitvoeren. Als >TEAMLEIDER< dus te veel door de zorg wordt opgeslokt, moet ik dat anders organiseren. Dat >TEAMLEIDER< toch met de teamleidertaken bezig kan houden. De gesprekken met de Raad van Bestuur. >TEAMLEIDER< is vaak aangeschoven omdat ik het belangrijk vind dat >TEAMLEIDER< ook hoort hoe dat soort gesprekken gaan. Dat hoort wel bij de bedrijfsleider. Het maken van een jaarplan. Daar ben je als bedrijfsleider verantwoordelijk voor. Het maken van een begroting ben je als bedrijfsleider verantwoordelijk voor. Maar ik doe dat altijd samen met mijn teamleider. Ja. Je doet dat altijd met elkaar. Dat kan niet alleen maar mijn kokervisie zijn van 'hier gaan we naartoe'. Je doet het met elkaar en je voed elkaar. Dus een teamleider moet ook kritisch kunnen zijn. Moet tegengas durven geven want dat is je klankbord.

**Interviewer:** Heeft u ook een visie op hoe een teamleider de functie moet uitvoeren?

**Respondent:** Ja. We hebben het er eigenlijk net al over gehad. Ik vind het belangrijk dat een teamleider heel goed tussen de bedrijfsleider en het team kan manoeuvreren. Ik vind het belangrijk dat een teamleider kennis van zaken heeft en klaargestoomd wordt om uiteindelijk bedrijfsleider te worden. Dat je ook op dat niveau presteert. En daarom doe je ook je teamleiderschap onder supervisie van een bedrijfsleider. Maar je bent uiteindelijk een kweekvijver voor de nieuwe bedrijfsleiders.

**Interviewer:** Zijn er nog bepaalde competenties waarover een teamleider zou moeten beschikken?

**Respondent:** Wat ik zie is dat een ontzettende grote plus is, is dat >TEAMLEIDER< het coachen van teamleiders heel leuk vindt. Dus als medewerkers zijn vastgelopen. De aandacht. Even een-op-een aandacht geven aan de medewerker. Dat vind ik ontzettend belangrijk. Maar daarnaast vind ik het ook belangrijk dat je strategisch kan meedenken en input kan geven. En je moet gewoon feeling hebben voor HR-zaken. Je kunt alles leren. Maar als je er geen feeling voor hebt, straal je dat ook uit. En ja weet je het is fijn als iemand heel nauwkeurig is. Maar goed dat zijn geen hoofdkwaliteiten. Het heeft impact als je fouten maakt, of als je dingen laat liggen, maar ik vind het veel belangrijker dat je in grote lijnen op een niveau zit waar je klaargestoomd wordt om bedrijfsleider te worden. En er is in huis gewoon een groot verschil zie ik.

**Interviewer:** Kunt u dat verschil eens onder woorden brengen?

**Respondent:** Ja. We hebben natuurlijk teamleiders vanuit de polikliniek en vanuit de kliniek. En een teamleider vanuit de polikliniek is van oudsher een polikliniek-assistent. Die heeft al een ander opleidingsniveau. Een andere vooropleiding genoten. Hebben geen HBO managementopleiding gedaan. Hebben vaak ook een veel kleiner clubje om aan te sturen en kunnen ook slechter afdeling-overstijgend kijken naar de organisatie.

**Interviewer:** Die worden veel eerder in de groepsdynamiek gezogen?

**Respondent:** Ja. Die zitten veel meer in dat poligebeuren. En ik zie ook wel dat collega-bedrijfsleiders anders met hun teamleiders omgaan. Ik betrek >TEAMLEIDER< heel erg bij het opstellen van de begroting, het maken van het jaarplan, kwartaalgesprekken met de Raad van Bestuur. Dat doet niet iedere bedrijfsleider. Die vinden 'dat hoort bij de bedrijfsleider en daar hoeft een teamleider niets van te weten'.

**Interviewer:** Oke. Het is dus de transparantie hier, de openheid?

**Respondent:** Ja. Niet iedere teamleider is daar nieuwsgierig naar en wil zich daarin ontwikkelen. Dat is natuurlijk ook wel een verschil. Sommigen vinden dat heel spannend die financiën, met de bestuurder praten over zaken. Dus die blijven daar ook weg van.

**Interviewer:** Ik hoor ook best wel veel vrijheid als we naar >TEAMLEIDER< kijken. Is het voor ﻿>TEAMLEIDER< ook duidelijk wanneer wat gedaan moet worden? Qua prioriteiten. Hoe kan een teamleider op de hoogte zijn?

**Respondent:** De jaargesprekken. Ik weet niet of die hier nu hangt. Maar normaal hebben we hier een Excel-sheet hangen. Van mij hoeven ze niet alleen maar in de maand mei gevoerd te worden, want we hebben daar het hele jaar voor. Maar het moet wel een keer per jaar gevoerd worden dus dat is een kwestie van plannen. En dat gebeurt ook.

**Interviewer:** Jullie kijken dus samen van qua werk 'wat moet er allemaal verzet worden en wanneer' en dan maken jullie een soort planning?

**Respondent:** Ja.

**Interviewer:** Valt het jaarplan daar ook onder?

**Respondent:** Ja. We hebben een prachtig mooie planning voor gemaakt.

**Interviewer:** Dat schept duidelijkheid?

**Respondent:** Ja. >TEAMLEIDER< maakt dan van dit soort mooie lijstjes. Dat helpt mij enorm omdat wij natuurlijk weer een heel ambitieus jaarplan hebben en dat is voor mij weer een richting van 'oh, dan moeten we dat klaar hebben en dan moeten we op dat aansturen'. Dus die checklist van 'zijn we nog in control, zijn we nog op orde, of moeten we gas geven?'.

**Interviewer:** Is er eigenlijk nog bepaald gedrag dat een teamleider uit zou moeten stralen?

**Respondent:** Je moet heel integer zijn omdat je natuurlijk ontzettend veel hoort. Niet delen daar waar het niet gewenst is. Je moet ook tegen een bedrijfsleider durven zeggen dat die te snel gaat of te veel wil of dat het team het echt nodig heeft op pas op de plaats te maken. Een beetje bewaken dat iedereen zich er goed bij voelt, aangehaakt blijft. Maar ja, met name de integriteit vind ik wel heel belangrijk. En ja je moet boven het team staan. Toch leiderschap uitstralen en wat dat betreft geleerd hebben dat je ook soms impopulaire maatregelen moet nemen en die doe je vanuit je functie en niet vanuit je persoon. En daar zie ik beginnend teamleiders nog wel een mee worstelen omdat die het heel graag zo goed willen doen dat ze met iedereen vrienden willen blijven. Dus ja. ik wil niet zeggen dat je een olifantshuid moet kweken maar je moet wel leren hoe je daar mee omgaat. Zonder jezelf geweld aan te doen.

**Interviewer:** Merkte u dat toen >TEAMLEIDER< hier begon als teamleider daar ook nog zoekende in was? Of was >TEAMLEIDER< heel standvastig wat dat aangaat. Om dus echt die impopulaire beslissingen te durven maken?

**Respondent:** Ja. >TEAMLEIDER< was er iets meer zoekende naar dan nu. Ik moet wel zeggen. Dat toen >TEAMLEIDER< de bedrijfsleider op >EVR< heeft vervangen voor langere tijd, >TEAMLEIDER< is >FUNCTIE< geweest. Dus >TEAMLEIDER< heeft al wel geleerd daar wat minder nou door van slag te zijn. Wat enorm geholpen heeft is dat een teamleider van een andere afdeling is gekomen. Dus die krijgt dan ook meteen die positie in het team. Want het is lastig als je als lid van het team ineens de teamleider wordt want 'wat weet jij er nou van, maandag was je nog een van ons?'. Dus je ziet dat dat veel minder soepel gaat. Het is niet onmogelijk, maar het is wel een lastige.

**Interviewer:** U heeft een duidelijk visie op de teamleider met verwachtingen. Waren die vanaf het begin voor >TEAMLEIDER< duidelijk toen >TEAMLEIDER< hier startte of was >TEAMLEIDER< nog zoekende?

**Respondent:** We hebben samen een beetje gezocht. Dat zie je dan. Dan gebeurt er iets in het team en dan denk ik 'oh, het is opgelost'. Daar moet ik ook even aan wennen. >TEAMLEIDER< moet daar aan wennen van 'tot hoe ver kan ik gaan, waar liggen de grenzen?'. We hadden nog seniors die ineens hadden van 'we horen niets meer, het wordt overgenomen'. Aan het begin was het echt wel een beetje een zoektocht met elkaar naar 'hoe liggen de verhoudingen?'.

**Interviewer:** Wat heeft daarbij geholpen in die zoektocht?

**Respondent:** Uitspreken naar elkaar. Als ik dacht 'ik ga dat even oplossen' en het is al gedaan, terwijl ik dat graag zelf had willen doen, uitgesproken. >TEAMLEIDER< heeft uitgesproken als er te veel op >TEAMLEIDER< bordje kwam of als >TEAMLEIDER< dingen naar zich toe wilde trekken terwijl ik die nog moeilijk vond om los te laten, en seniors het uitgesproken. Want >TEAMLEIDER< trok alles naar zich toe.

**Interviewer:** Toen waren de seniors een beetje buiten spel gezet?

**Respondent:** Ja. Die volgde dat heel erg zo. Dus 'prima, willen jullie dat doen, ga het doen, ga het oppakken, ga het regelen'. Dus het vooral uitspreken naar elkaar. Maar minder overal vakjes omheen willen plaatsen want het is natuurlijk een heel dynamisch bedrijf. Geen dag is hetzelfde. Dus het is soms nodig dat het als is opgepakt zonder dat het met iedereen is afgestemd. Zo loopt dat.

**Interviewer:** Ik ben er wel doorheen wat de vragen betreft. Ik weet niet of er dingen zijn die u nog toe zou willen voegen?

**Respondent:** Sommige bedrijfsleiders hebben geen teamleiders omdat het een bedreiging kan zijn. Als het zo goed gaan.

**Interviewer:** Dan zet die de bedrijfsleider buiten spel?

**Respondent:** Dat zou kunnen. Als je je als teamleider zo ontwikkelt dat je de functie van bedrijfsleider uit kan voeren. Er is ook wel een stukje angst van 'laat ik het zelf maar regelen, laat ik het zelf maar doen. Dan kan niemand een poot onder mijn stoel vandaan zagen'. Terwijl ik het iedereen zou gunnen om zo'n goede teamleider te hebben. Dat zorgt ervoor dat je zelf je werk veel beter kunt doen.

**Interviewer:** Dat je meer op de strategische dingen kunt focussen zonder constante verstoring door de dagelijkse operaties?

**Respondent:** Ja. En anders heb je het gevoel 'oh, ik had er toch meer aandacht aan moeten besteden'. En nu is het van 'oh, lekker, >TEAMLEIDER< is er al in gedoken'.

**Interviewer:** Dat het nog maar voorlopig zo door mag gaan?

**Respondent:** Ja. Ja. Ja.

*Einde transcript*

# Transcript Interview HR practitioner 1

**Duur interview: 55 minuten en 55 seconden**

**Functie: HR adviseur**

**Afdeling(en) onder supervisie: Zorg- en niet-zorg**

**Gender: vrouw**

**Werkervaring in functie: n.a.**

**Interviewer:** Misschien is het de bedoeling van het interview en waar het onderzoek over gaat. Het gaat over de teamleiders in het ziekenhuis hier. Vanuit de theorie is weinig bekend over deze mensen, maar voor HR zijn ze wel heel erg belangrijk. Ze staan namelijk ontzettend dicht bij de operationele medewerkers. Dus alles wat wij qua beleid maken, dat moet eerst lang die teamleider om goed uitgevoerd te worden. Ik ben vooral benieuwd hoe jij naar de rol van de teamleider aankijkt en dus ook wat je van zo'n teamleider verwacht. Daar komen we gaandeweg het interview op uit. Zoals we dus al weten, jij zit vooral op de facilitaire afdelingen qua HR advies, als ik het goed begrijp?

**Respondent:** Ja, ik heb wel twee zorgafdelingen waar eentje een teamleider heeft en een ander dat niet heeft.

**Interviewer:** Je zei hiervoor al iets van een soort kerstboomconstructie. Met, voor de ondersteunende afdelingen die maken dan gebruik van coördinatoren. Sommige dan, en sommige zorgafdelingen maken weer gebruik van seniors. Mag ik die vergelijken met elkaar? Zouden die qua verantwoordelijkheden op hetzelfde uit komen?

**Respondent:** Ik denk het haast wel. Ik moet even nadenken hoor. Als je bijvoorbeeld kijkt op het facilitair heb je op elke afdeling een coördinator. Sommige heetten eerst verantwoordelijken, maar dat is aangepast naar coördinator. Want eerste verantwoordelijke klinkt als zoals in het leger. Als een soort rang of zo. Dus zij zijn nu coördinatoren geworden en die sturen aan op de werkvloer. Die richten zich wel op het kwaliteitsdeel vaak of op het operationele deel. En dat doen de seniors ook wel. Zo heb je de senior OKE. Ik weet niet of je weet waar de OKE voor staat?

**Interviewer:** Ja.

**Respondent:** In basis betekent dat wel dat ze hetzelfde doen als coördinatoren.

**Interviewer:** Zouden zij ook iets van HR-taken hebben? Zoals jaargesprekken of iets dergelijks?

**Respondent:** Sommige zijn wel eens bij werving en selectie aanwezig. Dat gebeurt in de zorg ook. Dat verschilt denk ik per EVR. Ze spreken mensen wel aan, maar dat heeft dan meer te maken met taakinhoud. Verzuimgesprekken doen ze volgens mij ook niet. Maar als mensen ziekgemeld worden, dan nemen ze de ziekmelding wel aan. Dus dat HR aspect ...

**Interviewer:** Hebben ze een hiërarchische rol naar jou idee?

**Respondent:** Nee.

**Interviewer:** Ze staan in meer in het team en hebben een extra taak?

**Respondent:** Ja, het is een rol. Uiteindelijk is het net zoals de senior OKE hebben ze een rol. Allen bij facilitair is het allemaal van oudsher bestaan dat ze wel een functie als eerste verantwoordelijken hebben. Maar in basis verschilt het niet veel. Ze worden wel meer verloond dan normale medewerkers. Kijk bij afwas, zitten ze in 20. De eerste verantwoordelijke zit dan in 25 bijvoorbeeld. Terwijl in de zorg, krijgen ze een toeslag. Dus daar zit wel een verschil in.

**Interviewer:** Als we dan eens kijken naar de taken van de teamleiders, wat zijn dan naar jouw idee de algemene verantwoordelijkheden van de teamleider hier in het ziekenhuis?

**Respondent:** Voor zorg of voor facilitair?

**Interviewer:** Misschien eerst zorg en dan facilitair? Ik merk dat er volgens jou een verschil tussen zit?

**Respondent:** Ja, zeker. Er zit een verschil sowieso bij facilitair en bij zorg. Facilitair zijn, weet ik, verantwoordelijk voor eigen jaargesprekken, ze stellen eigen budget op en daar zijn ze verantwoordelijk voor. Ze zijn mede verantwoordelijk, niet eindverantwoordelijk. Ze doen alle arbo-aspecten. Je ziet bij de zorg, dat is wel anders. Daar laten zijn ze naarmate de jaren wel jaargesprekken gaan doen, maar dat is denk ik de laatste twee, drie jaren gekomen dat ze dat nu morgen doen. Maar, ze zijn niet verantwoordelijk voor het budget. Daar houden ze zich ook niet mee bezig. Dat doen ze bij het facilitair dan weer wel. Dat is dan wel echt een verschil.

**Interviewer:** Dus als ik het dan goed hoor zijn de taken voor een teamleider bij facilitair net iets breder dan voor zorg?

**Respondent:** Ja. Breder weet ik niet. Je heb net iets meer verantwoordelijkheid denk ik. Maar je ziet dat daar de inschaling ook weer anders is omdat de verantwoordelijkheden daarin anders zijn.

**Interviewer:** Enig idee waarom er een verschil zit?

**Respondent:** Bij EVR-zorg heb je één functiebeschrijving dat geldt voor een teamleider poli, maar ook voor een teamleider verpleeg. Dus daar zit geen onderscheid in. Terwijl het niveau kan verschillen. Die worden wel allemaal in hetzelfde FEG betaald: FWG 55. Bij facilitair is gewoon gekeken 'oké, wat is jouw aandachtsgebied en waar ben jij verantwoordelijk voor? Wie stuur jij aan?'.

**Interviewer:** Dus dan zijn de functiebeschrijvingen van facilitair op maat gemaakt?

**Respondent:** Ja. Niet op de persoon, maar gewoon 'wat vraag je'? En die FWG loopt dan van 45 tot 55.

**Interviewer:** Heb je zoiets van 'dat zouden ze ook bij de zorg moeten doen' of is één functiebeschrijving voor de zorg prima?

**Respondent:** Dat weet ik niet. Dat ligt aan hoe die mensen worden ingezet.

**Interviewer:** Puur op basis van de vraag daar is?

**Respondent:** Wat ik zelf zou doen, wat mijn advies zou zijn is om, een teamleider voor de verpleeg is echt wel anders dan een teamleider poli. Op de poli heb je doktersassistenten en polikliniekassistenten zitten in een lagere schaal, zijn op een andere manier verantwoordelijk, hebben andere risico's die dat met zich meebrengt. Ook in de aansturing. En dat ga je ook in een weging zien. Zoals mensen die nu ik de kliniek werken, op de >AFDELING< zijn vaak 45 tot 50 worden ze betaald en maken dan een sprong naar 55. Bij de poli maken ze een sprong van 35 naar 55 en dat is groot.

**Interviewer:** Dat is best een groot gat?

**Respondent:** Ja, zeker. En volgens mij moet je daar twee stromingen in gaan maken. Teamleider poli, teamleider kliniek. En dan wel dat iedereen hetzelfde doet. Dat vraag ik me nu dus af, of dat zo is. Maar goed, dat onderzoek jij nu zo mooi.

**Interviewer:** Ik hoorde net al wat HR-dingetjes voorbij komen. Als we dan kijken naar de teamleider, wat voor HR-taken liggen er dan bij zo'n teamleider? Als het het voor jou makkelijker maakt kun je weer een afscheiding maken tussen zorg en niet-zorg.

**Respondent:** Ik denk dat dat niet zo heel veel verschilt. Ik denk wel dat het ligt aan de leidinggevende als ik kijk bij mij bij de zorg is dat wel een verschil. Ik weet bijvoorbeeld wel dat de jaargesprekken worden verdeeld en dan in het begin werden de lastige gesprekken door de bedrijfsleider gedaan en minder-lastige gesprekken door de teamleider. Dat is bij de zorg. Hoe dat nu is in huis, dat weet ik niet. Bij facilitair is er maar één teamleider die verantwoordelijk is voor zijn club en die doet ook het jaargesprek. En de bedrijfsleider, dat is het hoofd. En die is hoofd van een hele club. En niet van een EVR dat één specialisme is, maar heeft verschillende specialisme onder zich. Dat is wel een verschil. En de span of control is veel groter. Een teamleider doet dan op HR-gebied: de jaargesprekken, de ARBO-verzuimgesprekken, de POP wordt besproken dus de ontwikkeldingen worden besproken, dus verzuim, ontwikkeling, en jaargesprekken, dat denk ik even in het kort. En alles wat er nog bij komt op het gebied van HR is een fube kan het natuurlijk zijn. Dat je samen een fube gaat schrijven. Of dat je gewoon eens een arm om iemand heen slaat. Dat soort gesprekken. Van 'nou, ik zie dat je niet lekker in je vel zit'.

**Interviewer:** Dat je dan ook echt een soort coachende, gespreksvoerende rol pakt?

**Respondent:** Ja, dat hoop je dan.

**Interviewer:** Dat is een verwachting die je hebt?

**Respondent:** Ja.

**Interviewer:** Oké. Ik had eens gekeken naar wat voor onderwerpen of taken er allemaal bij een teamleider zouden kunnen liggen, als je het lijstje zo ziet...

**Respondent:** Werving en selectie.

**Interviewer:** ... onderwerpen waarvan je denkt 'daar houden jouw teamleiders zich mee bezig?'

**Respondent:** Ik denk ook dat het afhangt van het team dat ze aansturen. Want ik denk ook, dat mentoren. Dan bedoel je mentoren op vakinhoud?

**Interviewer:** Ja, erbij staan of hulp bieden.

**Respondent:** Dat verschilt wel heel erg. Zoals bij >AFDELING<. Ik zie >TEAMLEIDER< nou niet er naast lopen van 'zo moet je een bed duwen'. Dus op vakinhoud vind ik dat voor sommige afdelingen lastig. Maar dat is puur vanwege het vak. Want voor zorg denk ik 'ja logisch, natuurlijk kijk je dan even met iemand want jij bent de teamleider en daar moet je dan ook voor zorgen'. Dat zou een senior ook prima kunnen doen.

**Interviewer:** Hoe gecompliceerder de taak, hoe groter de kans is dat een mentorende rol wordt gepakt?

**Respondent:** Nou, dat ligt er aan. Voeding bijvoorbeeld. Het is niet heel spannend, niet heel moeilijk. Maar ik kan me wel voorstellen dat je op een gegeven moment zegt van 'dat vind ik niet handig, dat vind ik niet verstandig hoe jij nu aan het tillen bent'. Dat hoeft dan niet heel ingewikkeld te zijn. Het gaat eerder om de bewustwording. Bijhouden van personeelsbestanden. Ik vind dat iets van HR. Want daar bedoel je mee? De dossiers?

**Interviewer:** Als er wijzigingen zijn of iets dergelijks.

**Respondent:** Ja, zij doen de mutaties. Zij hebben daar de meeste zicht op. HR voert uit. Introductie is wel tweeledig. Vanuit HR doen we hier de centrale introductie. Je hebt natuurlijk ook de introductie op de werkvloer. Moet je teamleider dat doen? Ik denk het niet. Ik denk dat een medewerker dat ook prima kan. Inwerken. Informatie, informele training. Nou, ontwikkelplannen moet je sowieso bespreken met je leidinggevende dus ja, een teamleider zeker. Disciplineren en corrigeren, ook. Afhandelen van tuchtprocedures en klachtenprocedures. Ja, meestal zie je dat de teamleider dat doet of de bedrijfsleider. Ik denk dat bij de zorg dat de bedrijfsleider vaker ingeroepen wordt met hulp van de teamleider, maar ik denk niet dat dat een ding is dat meteen bij de teamleider wordt neergelegd bij de zorg.

**Interviewer:** Heb je een idee waarom dat zo werkt?

**Respondent:** Omdat de bedrijfsleider de eindverantwoordelijke is.

**Interviewer:** Is het vanwege het risico dat met zo'n klacht komt?

**Respondent:** Ook. Ik denk ook dat het met hiërarchie te maken heeft. Als jij een klacht hebt ingediend als patiënt, dan heeft de bedrijfsleider je gehoord in plaats van de teamleider. Dat werkt dan zo. Bij facilitair wordt het meestal samen gedaan. Teamleider en hoofd. Die trekken daarin samen op. Ik denk ook dat dat logisch is omdat de teamleider van de inhoud weet. Het hoofd is eindverantwoordelijk en kan niet alles weten. Die doen dat vaker gewoon samen. Ik zie dat dat bij facilitair gewoon vaker samen gedaan wordt. Absentiemanagement dat is arbo. Kan een teamleider prima doen. Wel altijd in overleg. Bij een EVR zou ik zeggen, samen met de bedrijfsleider, maak daar afspraken over. Facilitair, kan een teamleider dat prima doen en het hoofd heeft het er over tijds de bila's. Waardering geven, volgens mij is dan niet functiegebonden. Volgens mij moet iedereen elkaar waardering geven.

**Interviewer:** Heb je een idee hoe teamleiders dat zouden kunnen doen?

**Respondent:** Zoals ze dat nu doen gaat dat via complimenten geven, waardering, maar ook benoemen 'goh, wat vervelend dat je thuis het zwaar hebt, maar top dat je bent gekomen'. Dat soort dingen.

**Interviewer:** Een stukje persoonlijke aandacht?

**Respondent:** Ja. Terwijl je ziet dat de waardering toch vaak uitgedrukt wil worden in geld.

**Interviewer:** Maar je zit een ziekenhuis, dat gaat minder makkelijk? Wat zijn de opties?

**Respondent:** Ze geven wel een gratificaties. Dat komt dan wel echt vanuit het management. Als je zoiets hebt van 'ik wil echt iemand belonen, wat kan ik doen?'. Je kunt een gratificatie geven. Maar als je weet dat iemand verlof te kort komt dan is het eerder van 'goh, geef hem een extra dag of schrijf een extra uurtje op'. Dat kan natuurlijk ook. Wees creatief daarin. Op zicht is er wel heel veel mogelijk. Een training en cursus is ook waardering. Zo zien ze het vaak niet. Of een paar nieuwe schoenen, een paar nieuwe klompen. Dat mensen dan denken 'dat hoef ik nu niet zelf te kopen, dat krijg ik van mijn werk'. Zien mensen dat als waardering? Nee. Maar, ik vind wel dat mee moeten uitdragen. Beslissen over salarisschalen. In deze CAO, nee. Alles is vastgelegd. Je kunt er soms creatief, maar we hebben een functieloongebouw en daar geloof ik heel erg in. Als je afwijkt moet je ook kunnen uitleggen waarom je er van af wijkt ook voor de ander. Daarom heb ik soms wel moeite met de teamleider EVR in 55 en bij facilitair een hele erge spoeling van op de functie gericht. Bottom-up, top-down, ja. Verbeteren van werklevens. Dat is het werkklimaat?

**Interviewer:** Misschien iets met vitaliteit of duurzame inzetbaarheid?

**Respondent:** Ja. Dat doe je samen met HR. Dit onderwerp is wel een hot item binnen het facilitair. Daar help ik ze ook bij en daar ondersteun ik ze ook in, maar het blijft moeilijk.

**Interviewer:** Enig idee waarom het zo'n hot item is?

**Respondent:** Het is fysiek zwaar. Het is een ouder personeelsbestand. Als we kijken naar >AFDELING<, dat zijn redelijk oude dames. Het is fysiek zwaar werk. Mensen werken hier al ugh jaar. Die kennen de teamleider dus al een langere tijd. En de teamleider vind het moeilijk om dat gesprek aan te gaan. Maar dat is wel echt de basis. Durf dat gesprek aan te gaan.

**Interviewer:** Daar ligt dan ook een rol voor de teamleider?

**Respondent:** Ja dat klopt. Die moet dat doen, maar dat doet hij niet. Of ze vinden het moeilijk, die moeten daarin geholpen worden. Die vitaliteit, die duurzame werktoekomst is echt wel tweeledigheid. Teamleider en het hoofd, maar ook HR. Samen. Zorg of niet-zorg. Ziekenhuisbreed.

**Interviewer:** Als we dan kijken naar de bevoegdheden die zo'n teamleider heeft. Waarover zou zo iemand naar jou idee dan mogen beslissen?

**Respondent:** Ja, lastig. Over opleidingen. Sowieso. Want die staan sowieso dichter bij de werkvloer dan een hoofd of een bedrijfsleider. Dat is gewoon zo. Dus die weten ook gewoon 'iemand wil een cursus doen, past dat ook, hebben we dat nodig?'. Ik denk dat ze daar wel wat meer beslissingsbevoegdheid voor mogen krijgen. Of in ieder geval wel iets meer stem. Arbo-verzuim doen ze natuurlijk altijd al samen met mij. Maar ik denk wel dat het goed is om in de zorg wat meer naar voren te laten komen omdat wij ook. Ja, zij zitten er gewoon echt tussen. Zij zijn echt die tussenlaag. En weetje, die brug is best wel ver tussen medewerker en bedrijfsleider. Dan heb je gewoon iemand nodig die die vertaling maakt. Dat is wel heel erg mooi. Je merkt wel dat dat anders is bij facilitair. Dan is het echt dé leidinggevende.

**Interviewer:** Hoe  bedoel je dé leidinggevende?

**Respondent:** Als ik verpleegkundige ben en ik moet naar mijn leidinggevende, dan is dat laagdrempeliger. Want natuurlijk daarboven zit de bedrijfsleider. Die is de baas van mijn hele EVR. Als ik kijk naar facilitair, dan moet naar mijn leidinggevende van >AFDELING<. Dan ga ik niet direct denken van 'goh, daar zit nog een manager boven die verantwoordelijk is voor Voeding, >AFDELING<, >AFDELING<, Keuken'. Daar ga je dan niet zo snel heel.

**Interviewer:** Die drempel is dan te hoog?

**Respondent:** Te hoog. Dat is dan hetzelfde dat ik nu naar >LID RAAD VAN BESTUUR< zou gaan. Dan sla ik >HOOFD< en >MANAGER< over. Dat doe je niet zo snel. Dat is mijn gevoel. Terwijl de mensen met het hoofd best wel kunnen en vinden hoor. Ik denk dat dat het is waar ze over kunnen beslissen. Ik denk dat niet echt waarvan ik denk 'dat is iets'.

**Interviewer:** Hebben ze meer een adviserende stem of mogen ze ook echt harde beslissingen maken?

**Respondent:** Ja. Toch wel over ontwikkeling denk ik. Ook over opleidingsbudgetten. Over opleidingen. Over misschien nog wel het roosteren. Dat weet ik niet precies. De processen op de werkvloer, volgens mij mogen ze daarin wel wat meer stem krijgen. Maar goed, praktisch gezien snap ik dat dat niet altijd kan.

**Interviewer:** Heb je het idee dat die stem bij de manager of het hoofd ligt?

**Respondent:** Of bij de voorzitter zelf. Het is dan echt een kwestie van doen wat je gezegd wordt. Wat ik ook wel weer begrijp. Je zit hier midden in een zorgproces met 'de dokter'. En die heeft het ook gewoon voor het zeggen. Is dat goed? Nee. Je moet het namelijk met elkaar doen. Je bent gelijkwaardig aan elkaar. Daar zie je dan nog wel het verschil.

**Interviewer:** Heb je wel het idee dat de stem van de teamleider goed gehoord wordt? Dat als een teamleider een idee heeft, dat deze gehoord wordt. Er moeten namelijk vind wat lagen doorboord worden.

**Respondent:** Dat weet ik niet. Ik heb >EVR<. Nee, dat weet ik niet. Voor >EVR< is het meer voor de aansturing van het personeel en de bedrijfsleider doet meer het bedrijfsmatige. Die hebben daarin wel een mooie verdeling gemaakt. Procesmatig, daar heb ik geen zicht op. Dat vind ik lastig.

**Interviewer:** Zou een teamleider dan ook nog een financiële bevoegdheid hebben. Mogen ze aankopen doen?

**Respondent:** Tot een bepaald bedrag wel denk ik. Een bos bloemen. Die waardering, daar hebben we het dan over. Die worden dan door de bedrijfsleider besteld. Volgens mij zijn ze niet tekenbevoegd. Budgettair, dat weet ik eigenlijk niet. Dat zou wel mooi zijn.

**Interviewer:** Waarom zou je dat mooi vinden?

**Respondent:** Nou ja, als jij teamleider bent en jij wil iemand waarderen en je moet eerst langs vijf verschillende schijven om een bloemetje te krijgen. Dan zou ik denken of 'laat maar' of de bedrijfsleider loopt er mee weg. Daar gaat dan jouw waardering.

**Interviewer:** Heb je voor jezelf nog een idee, als het aankomt op de uitvoer van de HR-taken, wanneer dat naar tevredenheid gedaan is?

**Respondent:** Hoe bedoel je?

**Interviewer:** Stel ik ben een teamleider en moet jaargesprekken voeren. Dat kan ik heel slecht doen, maar dat kan ik ook goed doen. Heb je daar een mening over ...

**Respondent:** Wanneer je een geslaagde teamleider bent?

**Interviewer:** ,.. zeker op het gebied van die HR-taken, dat is best moeilijk om daar een waardeoordeel aan vast te hangen.

**Respondent:** Dat is inderdaad moeilijk. Je begeeft je dan op glad ijs. Er zullen ook genoeg bedrijfsleiders en hoofden zijn die echt slecht zijn. Volgens mij is het veel breder te trekken: wat maakt een goede leidinggevende?

**Interviewer:** Heb je daar een mening over?

**Respondent:** Ja, zeker. Ik denk gewoon als je het gesprek openlijk met je medewerkers aan durft te gaan. Dus niet alleen in tijden dat het goed gaat, maar ook in slechte tijden. Dat je het daarover kan en mag hebben. Zonder aanvallend te zijn. Uiteindelijk zijn we een bedrijf en heb je je doelen te behalen en daar heb je je beste mensen voor nodig. En waarom mag je daar mensen niet op aan mogen spreken. Natuurlijk op een verbindende manier, en op een normale manier. Maar wel met elkaar. En daar kan een teamleider misschien net iets meer in betekenen dan een bedrijfsleider omdat die wat dichter bij de werkvloer is. Dus als je die verbinding kan maken, dat je dan al een heel eind bent voor je EVR. Maar wanneer schrijf je een goed verslag? Dat is een lastige. En wie corrigeert je daarin?

**Interviewer:** Heb je als HR adviseur wel eens een moment gehad dat je moest ingrijpen?

**Respondent:** Ja, zeker. Ik zit niet veel bij jaargesprekken, maar ik kreeg toen een stapeltje jaargesprekken voor mijn neus en dat was letterlijk copy paste. Medewerker A, B, C, en D hadden alle vier hetzelfde verhaal en daar stond de handtekening onder van de teamleider en het hoofd. En toen had ik echt zoiets van 'dit kan gewoon echt niet'. Dit is gewoon een belediging voor de medewerker. Je neemt een medewerker dan niet serieus. Ga gewoon het gesprek aan. Iedereen verdient gewoon even dat momentje. Eén keer per jaar, een uurtje.

**Interviewer:** Als je dan de reactie peilt van zo'n teamleider, is die dan verrast van 'goh, dit is een verassing. Die verwachting van jou kende of wist ik helemaal niet?'.

**Respondent:** Dat is heel wisselend. Sommige kunnen het prima verantwoorden van 'goh, dit heb ik dan daar en daarom gedaan'. Dat is dan duidelijk, maar laten we voor een volgende keer daar dan duidelijke afspraken over maken dat we dat wel anders kunnen doen. En als je het moeilijk vind, dan wil ik je daar graag in ondersteunen. Ik heb ook mijn bila's. Dus ik hoor ook wel eens dingen en vraag ook wel eens dingen 'wat vind je?'.

**Interviewer:** Wie zitter er bij de bila's?

**Respondent:** De teamleider en ik. En ik heb dan ook eens in de zoveel tijd ook met het hoofd een bila en dan bespreken we gewoon over alle afdelingen en ook even over alle teamleiders van 'wat vind jij daarvan?' of 'let even op >TEAMLEIDER< van >AFDELING< want ik zie dat >TEAMLEIDER< te hard wil' of 'let even op >TEAMLEIDER<'. Dan probeer ik het hoofd daarin ook een beetje te faciliteren. Maar ik geef het ook terug aan de teamleider zelf.

**Interviewer:** Daar moet het uiteindelijk gedaan worden?

**Respondent:** Ja, ik doe het uiteindelijk wel aan twee kanten. Want uiteindelijk moeten de teamleiders daar ook wat mee. En dat vind ik wel eens moeilijk aan mijn rol daarin.

**Interviewer:** Dat je er midden in zit?

**Respondent:** Ja, want ze vinden ook wel echt wat van elkaar.

**Interviewer:** Dan ben je echt mediator als het ware?

**Respondent:** Ja. Ik vind daar ook wat van en dan moet je maar kijken van 'tja, wat doe je er mee?'. Want je wil de ene helpen, maar aan de andere kant zie je dat de teamleider stagneert en die moet dan ook geholpen worden. En daar moet je dan een weg in vinden. Dat is lastig.

**Interviewer:** Heb je ook het idee dat een teamleider ook nog bepaalde kennis nodig heeft om zijn of haar taken uit te kunnen voeren?

**Respondent:** Ik denk dat ze wel iets van leiderschap moeten hebben gedaan. Als is het dan maar een training of een cursusje. Dat vind ik een beetje oneerbiedig gezegd, maar ik heb bij Voeding nu twee nieuwe Eerste verantwoordelijken. Die vinden het gewoon heel moeilijk om mensen aan te spreken. Wat mag ik zeggen? Wat mij betreft mag je alles zeggen, als je het maar gewoon respectvol doet. Maar het zou wel helpen als je een soort van kader meekrijgt van te voren. Hoe doe je dat gesprek met mensen aangaan dat lastig is? Want waardering uitspreken dat vinden we allemaal niet zo moeilijk. Het gaat om het benoemen van de dingen die je ziet die niet goed gaan en dan niet feitelijk op inhoud, maar op houding en gedrag. Ik denk dat ze wel getraind moeten worden van 'hoe doe je dat'. En zeker als je van de vloer komt. Dat is helemaal moeilijk. Dan was je eerst een van hen en nu sta je daar boven. Dat doet ook wat met jou.

**Interviewer:** Heb je het idee dat teamleiders er echt boven staan? Of zijn het eerder mensen die in het team staan en dan er boven gaan staan als het nodig is?

**Respondent:** Ik denk het tweede. Ik denk dat het tweede het geval is, maar dat het anders wordt gezien. Want dat is toch wel hoe het gezien wordt na een tijdje. Je bent dé teamleider. Je staat er boven. En niet iedereen komt uit het team en wordt teamleider. Bij Facilitair zijn het gewoon teamleider die niet uit het team komen. Dat is dan makkelijker, lijkt mij.

**Interviewer:** Oke. Dus leiderschap. Zijn er nog andere dingen. Heb je nog HR-kennis nodig of kun je die juist weer bij jou halen?

**Respondent:** Bij mij inderdaad. Ik denk wel dat je iets moet weten van ARBO. Van arbeidsrecht is ook wel handig. Ik merk wel nu als ik kijk naar teamleiders, die ik het afgelopen jaar heb aangenomen dat teamleiders die gepokt en gemazeld zijn op het gebied van HR, dat werkt voor mij wel een stuk prettiger. Ik zie dan ook dat het in het team ook beter gaat. Ik heb ook teamleiders die dat niet hebben en dan komen van 'hoe zit het met ouderschapverlof'. Dat soort basisdingen. Ik vind wel dat je dat moet weten.

**Interviewer:** Denk je dat teamleiders weten  dat jij dat van hen verwacht, dat zij een stukje basis moeten bezitten?

**Respondent:** Ja, dat maak ik ze wel duidelijk. Met het aannamebeleid. Dus met het aannemen van mensen, let ik daar nu wel steeds meer op. In het begin lette ik daar niet heel erg op. Je ben nu al zo lang leidinggevende, dus dan weet je dat. In de praktijk blijkt dat tocht wel tegen te vallen. Als we mensen aannemen dan kijk ik daar wel specifiek naar en vraag ik daar specifiek op door.

**Interviewer:** Merk je dan ook dat teamleiders die meer HR-kennis hebben, in vergelijking tot degene die minder HR-kennis hebben, minder vaak voor wissewasjes bij jou aan het bureau staan?

**Respondent:** Of juist niet. Juist niet denk ik. Dat verschilt heel erg per afdeling. Soms denk ik 'ik zie jou heel erg weinig en hoe kan dat?'. Vanuit mijn ivoren toren, ik zie en hoor een hele boel en denk ik 'gaat het daar wel goed'. En soms laat ik het gebeuren. Want dan adviseer ik wel van 'daar hebben we het over' en dan krijg ik een soort van nee. Dan laat ik het soms gebeuren. En soms grijp ik wel in en, als ik tot kwellens aan toe, het er over te hebben. Er moet wel echt iets gebeuren, want de mensen zijn ontevreden of er gebeurt wat op zo'n afdeling.

**Interviewer:** Komen er wel  eens van dat soort klachten bij jullie terecht? Dat het de afdeling overstijgt?

**Respondent:** Niet zo snel bij mij moet ik zeggen. Wel bij het hoofd. Bij >AFDELING< en Zorg is het Hoofd snel gevonden. Daar heb ik ook van gezegd 'dat moet je niet doen'. Daar ondermijnt dan eigenlijk je eigenlijk je eigen teamleider. Dus dat gebeurt dan ook nu. Maar dat kost tijd.

**Interviewer:** Als we dan eens kijken. Je noemde net al een soort samenwerking tussen jou, bedrijfsleider, en teamleider. Hoe zie je die samenwerking voor. Waar moet ik, als ik naar de taken van de teamleider kijk, de grens trekken. Wat overstijgt een teamleider?

**Respondent:** Ja, goede vraag. Ik denk dat de bedrijfsleider of het hoofd wil loslaten. Ik denk dat deze vraag voor Zorg meer relevant is dan voor Facilitair omdat het echt wel in Facilitair gekaderd is. Dit is waar teamleider verantwoordelijk voor is, en daar bemoeit het hoofd zich niet mee. Bij Zorg is het best wel een grijs gebied en moet ook de bedrijfsleider dingen loslaten. Kijk we hebben de functiebeschrijving wel. Maar er zijn ook een heleboel dingen die de mensen niet doen die wel in de functiebeschrijving staan. Er zijn ook een heleboel dingen die de mensen wel doen, maar niet in de functiebeschrijving staan. Ik denk wel dat er een grijs gebied is. Waar trek je de lijn. Daar moet je het ook echt met elkaar over hebben.

**Interviewer:** Is het dan voor teamleiders wel duidelijk wat ze moeten doen?

**Respondent:** Ja, dat denk ik wel. We hebben natuurlijk allemaal onze functiebeschrijving waarin staat wat we moeten doen. En bij teamleider staat dan ook dat hij of zij jaargesprekken moet doen. Maar vanuit mijn verleden weet ik dat teamleiders dat niet deden omdat bedrijfsleiders dat niet wilde. Dan kun je het wel op papier hebben, maar als het niet gebeurt, dan, en de teamleider vindt het prima, dat kan natuurlijk ook, wie ben ik dan om te zeggen 'het staat op papier, je moet het doen'.

**Interviewer:** Maar in principe zijn functiebeschrijvingen wel een goede houvast voor nieuwe teamleiders om, bijvoorbeeld, hun rol in kaart te brengen?

**Respondent:** Ja. Zo is het stuk kwaliteit aan toegevoegd. Dat teamleiders zich ook daarmee bezig houden. Dat is nu iets wat nu nog niet heel specifiek opschreven staat, maar wel van essentieel belang is voor een ziekenhuis.

**Interviewer:** Wat moet ik mij daar dan bij voorstellen?

**Respondent:** Kwaliteit van de zorg. Dus meer kijken naar het werkproces. Kijk, niet alle teamleiders zijn verpleegkundigen. Dus het is vrij lastig als je niet verpleegkundige bent om je dan te bemoeien met een verpleegkundig proces. Want je kunt wel een soort van 'ik heb daar een idee bij, jij hebt daar een idee bij'. Hoe dat in praktijk werkt, geen idee. Dan kan juist een teamleider van toegevoegde waarde zijn omdat die van de vloer komt en kan zeggen 'jongens, dit is helemaal niet handig hoe we dit doen, we moeten hier wat anders mee doen' en dan samen met de bedrijfsleider optrekken om dat te realiseren, als dat nodig is.

**Interviewer:** Maar, niet alle teamleiders komen uit het werkveld, of wel?

**Respondent:** Bij facilitair speelt het kwaliteitsaspect op een heel ander niveau.

**Interviewer:** Het zou dan niet lastig zijn voor een teamleider een uitspraak te kunnen doen van 'ik zou het zo en zo doen'?

**Respondent:** Ik denk het niet. Dat komt omdat je niet met protocollen te maken hebt. Bijvoorbeeld bij Voeding moet je je voedingsvoorschriften weten, maar opzicht als je inzicht hebt, want het heeft ook met inzicht te maken, over werkprocessen en logica, dan kom je ook wel een heel eind denk ik.

**Interviewer:** Heb je ook nog voor ogen hoe je de HR-taken uitgevoerd wil hebben, of wordt dit juist heel vrij gelaten?

**Respondent:** Als zij dat prima vinden en de bedrijfsleider vindt dat prima, wie ben ik dan om te zeggen 'dat moet je niet doen'? Volgens mij moet je het zo makkelijk mogelijk maken voor jezelf en je functie zo leuk mogelijk maken. Laat de teamleider de dingen doen waar de bedrijfsleider geen tijd voor heeft. De bedrijfsleider is uiteindelijk verantwoordelijk voor de toko.

**Interviewer:** Dit zou uiteindelijk geen stress opleveren voor de teamleiders, dat ze zoveel vrijheid krijgen. Dat ze bijvoorbeeld een jaargesprek moeten voeren. Ze hebben een format, maar weten verder niet hoe het gesprek te voeren.

**Respondent:** De ene zal dat fijn vinden, en de ander zal dat minder fijn vinden. Volgens mij begint het dan ook echt om samen met elkaar te kijken van 'wat verwachten we van elkaar?'. Wederzijdse verwachtingen. Ik verwacht als bedrijfsleider dit van jou, wat verwacht je van mij. En het dan met elkaar te hebben over 'hoe bedienen wij de EVR?'. Hoe gaan we doelen halen, wat is jouw rol, wat is mijn rol?

**Interviewer:** Zijn daar dan bila's voor?

**Respondent:** Nee, dat is echt wel iets tussen bedrijfsleider en teamleider vind ik. Daar kan HR in ondersteunen waar nodig of waar gewenst. We kunnen mijn z'n drieën het er over kunnen hebben. Dat zou ik wel adviseren omdat je dan een objectief derde persoon hebt die zegt. Nou ja, je ziet soms dingen gebeuren waarvan je denk 'misschien spreek het zichzelf niet uit' of 'is dat wel handig?'. Dan treedt je als een soort mediator op. Ik zet even te denken hoe dat er bij Facilitair er aan toe gaat. Dat is allemaal al gekaderd. En als ze er niet uit komen, dan gaan ze of naar het hoofd of naar mij. Of wij hebben het er met z'n drieën over. Dat gaat gewoon zo.

**Interviewer:** Als vanzelfsprekend?

**Respondent:** Ja, vanzelfsprekend. Omdat je verantwoordelijk bent voor je eigen club en omdat je nooit iemand hebt waar, ja je moet wel verantwoording afleggen, maar uiteindelijk is het wel jouw clubje, jouw budget, jij bent voor alles verantwoordelijk.

**Interviewer:** Heb je het idee dat teamleiders bepaalde competenties nodig hebben?

**Respondent:** Ja, je moet sowieso sociaalvaardig zijn. Empathisch vermogen. Dat zijn al die HR-termen. Je moet menselijk zijn. Ik vind ook dat je een soort zakelijk inzicht moet hebben. Dat je ook, sommige dingen zijn gewoon zakelijk en moet je ook gewoon zakelijk kunnen aanpakken.

**Interviewer:** Bedoel je dan met zakelijk dat je echt weet hoe de organisatie in elkaar zit qua hiërarchie, processen, hoe je dingen gedaan krijgt?

**Respondent:** Ja, het is niet alleen maar kijken naar de patiënt. Uiteindelijk ben je een bedrijf die gerund moet worden. Natuurlijk staat je patiënt bovenaan. Maar uiteindelijk moet je ook kijken van 'hoe kunnen we dat het beste bedienen en dat zo goed mogelijk met het personeel dat wij hebben'. Wees ook niet bang om mensen aan te spreken. Is dan een competentie, mensen aanspreken? Duidelijkheid, durf.

**Interviewer:** Lef hebben?

**Respondent:** Ja, durven. Leiderschap tonen.

**Interviewer:** Daar komt het dan toch weer op neer.

**Respondent:** Ja, zoals ik het altijd zeg 'in goede en in slechte tijden'.

**Interviewer:** Zijn er voor teamleiders ook nog bepaalde momenten dat bepaalde taken uitgevoerd moeten worden? Zijn er deadlines die aan teamleiders gesteld worden?

**Respondent:** Nee. Uiteindelijk moet je in een jaar al je jaargesprekken hebben gedaan. Het MO wordt voor ze gedaan en verwachten we dat er binnen een jaar toch wel terugkoppeling is geweest. Maar dat zijn geen harde eisen. Ik adviseer daar wel over. Zeker voor de grote clubs. Dat ik zoiets heb 'ga nou niet 80 vrouwen aan het einde van het jaar doen. Dat haal je niet. Spreidt het uit over een jaar'. Maar harde deadlines zijn er niet zo ver ik weet.

**Interviewer:** Weten teamleiders dan van hun bedrijfsleiders dan wat ze moeten doen voor de aankomende tijd qua taken? Of zeg jij dat er wat moet gebeuren en wanneer dit moet worden verzet?

**Respondent:** Ik informeer wel dat als ik zie van 'dit gaat niet goed, ik zie dat je nog helemaal geen jaargesprekken hebt gedaan'. Ik houd wel een vinger aan de pols. Maar dat is niet mijn verantwoordelijkheid. Die moeten ze zelf pakken. Ik denk ook dat ze dat prima kunnen en dat je dat niet in beton hoeft te gieten. Dat gaat organisch.

**Interviewer:** Zoals die jaargesprekken. Dat is best wel een hard iets. Je moet gewoon binnen een jaar met iedere medewerker een gesprek hebben gehad. Zijn er meer van dit soort dingen?

**Respondent:** Ja. Frequent-verzuimgesprekken. Als mensen vaker dan drie keer ziek zijn per jaar. Dat is wel iets dat moet. Wordt het gedaan, dat vraag ik mij af. POP bespreek je tijdens je jaargesprek. Heb je het over een training of een cursus die je wil ontwikkelen, dan komt daar een POP uit voort. Wie pakt daar de regie in? POP is van een medewerker. Maar als die niet af komt, gaat daar dan een teamleider achter aan of een bedrijfsleider? Dat zijn dingen die moeten, maar of die ook gedaan worden. Daar hebben we geen harde afspraken over gemaakt.

**Interviewer:** Denk je dat het nodig is die afspraken?

**Respondent:** Weet je wat is. Nu bijvoorbeeld in de CAO staat dat je een POP moet maken, dat iedereen een PLB-plan moet maken. We doen het nu omdat het voorgeschreven is. Omdat het moet. Maar dan is het meer een doel, dan een middel. En daar geloof ik niet zo in. Ik denk gewoon dat je het moet doen omdat je het wil en niet omdat het moet. Dan streef je het doel voorbij. PLB-plan heb je voor hoe je je PLB-uren wil inzetten. Daar hebben we een mooi format voor. Geef maar aan wat je wil. Dan staan er twee woorden van 'ik wil mijn uren dus ik schrijf nu dit op'. Je gaat dan niet het resultaat behalen wat wil. Dus ik geloof in het organisch. En soms even harde sturing erop.

**Interviewer:** Heb je eigenlijk nog verwachtingen van het gedrag dat een teamleider moet vertonen?

**Respondent:** Ik denk dat je positief moet zijn. Je moet een positieve vibe hebben. Je moet mensen kunnen enthousiasmeren. Waardering uitspreken. Echt wel een positieve noot proberen te zijn. Maar ook mensen meenemen. Soms vinden mensen het moeilijk om dingen aan te pakken of te zien qua verandering. Dat je ook mensen daarin mee kunt nemen. Dat je laat zien dat veranderen leuk is. Het beste proberen uit mensen te halen. Volgens mij is dat wel een goede eigenschap.

**Interviewer:** Heeft HR nog een rol daarin om dat gedrag aan te brengen?

**Respondent:** Ik vind het heel erg lastig dat je met zo'n bila maar éénrichtingsverkeer hebt. Je ziet maar één kleur. Ik praat met de teamleider of met het hoofd en ik krijg de kleur van het hoofd of van de teamleider en niet van de medewerker. Wat ik wel dan soms lastig vind is dat ik dan, ik hoor van deze persoon zwart. Maar tussen de regels door lees ik toch iets anders. Hoe kan ik er nou voor zorgen dat ik niet helemaal mee ga met die teamleider? Hoe kan ik objectief blijven?

**Interviewer:** Hoe doe je dat?

**Respondent:** Ik ga dan sowieso even op onderzoek uit bij de medewerkers. Ik probeer her en der wat informatie op te snorren. En het zit ook hier he. Het zit ook in je onderbuik. Je hebt ook wel mensenkennis. Op een gegeven moment zie ook wel van 'jij bent gewoon niet zuiver'. Of je hoort dingen. Dat leer je vanzelf. Maar het is wel moeilijk hoor. En soms zit je dan aan tafel en ga je dan het gesprek aan met de medewerker en dan komt het op tafel en geeft die medewerker de kleur oranje. Ho, wacht even. We gingen er zwart in, nu is het oranje. Dan ben ik ook wel zo stoer genoeg om te zeggen van 'ik heb  nu deze kant van het verhaal gehoord, ik hoor nu dit, daar zit wel een verschil tussen. Wat gaan we nu doen?'. Daar moet je dan heel eerlijk in zijn. En dat is heel moeilijk. Eerlijk niet...

**Interviewer:** Maar objectief?

**Respondent:** Ja. Je bent er uiteindelijk voor de medewerker. En weet je. Voor 99 procent spreek ik de medewerker niet. Dus hoe objectief ben ik dan?

**Interviewer:** Vind je dat jammer?

**Respondent:** Ja. Dat doe ik natuurlijk zelf ook. En waarvoor zou ik naar de werkvloer gaan. Dan neem ik eigenlijk alweer de rol van de leidinggevende over.

**Interviewer:** Dan ga je er wel echt midden in staan.

**Respondent:** Ja. Het is ook meer mijn nieuwsgierigheid dan he. Dit was off-topic.

*﻿Interviewer lacht.*

﻿*﻿Respondent lacht.*

﻿**Interviewer:** Zwaar off-topic. Ik was nog wel benieuwd. Toen ik hier voor het eerst kwam, hoor ik al gelijk over die teamleidertraining waar iedereen toch best wel erg enthousiast over was...

**Respondent:** Dat teamleiderstraject?

**Interviewer:** Ja. Kun je daar misschien wat meer over vertellen?

**Respondent:** Volgens mij hebben we er drie gedaan. De laatste was vorig jaar of twee jaar terug. We hebben er verschillende gehad. We hebben er twee of drie gehad. En eigenlijk zijn die iedere keer weer anders geworden. De eerste keer was het een soort persoonlijke effectiviteit, leiderschap, dat soort dingen werden dan aangedragen. Terwijl er eigenlijk veel meer behoefte was aan 'hoe doe ik nu zo een spannend gesprek', 'hoe doe ik nu een frequent-verzuimgesprek', 'hoe zit het nou met die Arbowetgeving?', 'hoe lees ik nou in godsnaam zo'n budget of zo'n begroting?'. Die vragen kwamen en we hebben het traject dus steeds meer op maat gemaakt. Is het als succesvol ervaren? Dat weet jij nu.

*﻿Interviewer knikt*

﻿**Respondent:** Oke.

**Interviewer:** Dat is dus het leuke. Juist omdat het nu zo over verwachtingen gaat, die training schept juist hele duidelijke verwachtingen van wat er van die teamleider verwacht wordt...

**Respondent:** Ze hebben dat zelf aangedragen volgens mij hoor. Wat er is ook niet gezegd. Dat kan ook niet. Want het is Zorg en Facilitair en >AFDELING<, whatever. Dat is allemaal bij elkaar gezet. Die eerste lading was wel alleen maar zorg. Die laatste was een heel gemixt gezelschap. Toen hebben ze het echt met elkaar erover gehad 'oke, wat doe jij, oh nou dat doe ik niet, oh dat doe jij wel'. Er zijn geen kaders gegeven.

**Interviewer:** Het was dan eerder dat deze mensen bij elkaar hebben gezeten, dan dat jullie een presentatie hebben gegeven van 'je bent teamleider. Dit doe je en die dit dit dit'.

**Respondent:** Nee. Vanuit HR hebben we wel een training gegeven. Of een workshop. Waar ging het nou over? Volgens mij de visie 'wat wil je nou met je afdeling?'.

**Interviewer:** Dat is dus eigenlijk best wel bijzonder dan. Want terwijl je in eerste instantie dus zou denken dat zo'n training dan heel erg...

**Respondent:** Zenden?

**Interviewer:** ... ja. Het is eerder op vaardigheden ingegaan in plaats van wat jullie hebben duidelijk gemaakt wat er nou bij zo'n teamleider komt kijken.

**Respondent:** Ja. Klopt. Omdat het ook geen eenheidsworst is. Natuurlijk heb je verwachtingen. En tuurlijk moet iedereen een jaargesprek doen en worden mensen daarin getraind. Want ook zij hebben allemaal de Training Jaargesprek gehad. Net als een bedrijfsleider en een voorzitter. Dus daar zijn ze wel echt in meegenomen. Alleen is er niet gezegd 'jij moet dat doen'. Volgens mij werkt dat wel het beste. En tijdens het teamleiderstraject denk ik dat er is gebeurt dat ze het er met elkaar over hebben gehad ook hebben gezien 'wat leuk dat jij dat daar doet, nou dat neem ik dan ook mee naar mijn EVR' of 'wat gek dat jij dat nou moet doen, want wij doen dat niet zo. Hoe zit dat?'. Dat je dat met elkaar bespreekt en met elkaar zelf de grenzen bepaalt wat ze wel en niet hebben gedaan?

**Interviewer:** Merk je grote verschillen tussen teamleiders?

**Respondent:** Nee, bij mij in ieder geval.

**Interviewer:** Dus het is niet het geval dat als we twee teamleiders bij elkaar zetten, en ze gaan het over elkaars werkzaamheden hebben, dat er een enorm gat tussen zit.

**Respondent:** Dat weet ik niet. Een paar jaar geleden had ik >AFDELING< met >TEAMLEIDER< die mocht niets van >BEDRIJFSLEIDER<. Dat was >AANTAL< jaar geleden. Het is ook moeilijk. Als je als bedrijfsleider verantwoordelijk bent geweest voor je club, en je moest alles doen, en je moet nu alles gaan loslaten, terwijl dat ook nog dingen zijn die jij super leuk vond aan je werk, dat is best lastig. Dus dat heeft ook wel zo moeten ontstaan Daarom dan ook dat ene bedrijfsleider zegt 'doe jij die jaargesprekken, daar heb ik helemaal geen zin in en geen tijd voor', terwijl de ander zegt 'laten we samen dat met elkaar doen, fifty fifty, of zo'. Daar is geen goed of fout geweest.

**Interviewer:** Was het vroeger dan zo dat alle HR-taken die nu bij de teamleider liggen allemaal bij een bedrijfsleider lagen?

**Respondent:** Ja.

**Interviewer:** En het was te veel voor de bedrijfsleider? Dat daarom de taken nu bij de teamleider liggen?

**Respondent:** En omdat de behoefte er was voor meer sturing op de werkvloer. Ik denk als bedrijfsleider zit je er gewoon enorm boven. Als ik kijk naar >HOOFD< als hoofd. Die kan niet al die mensen. Wat zijn het 200 of 300 man in totaal. Die kan a niet alle jaargesprekken doen. En b heeft >HOOFD< er geen zicht op. Dus het is vrij logisch dat je daarom er een laagje tussen maakt. Ik merk dat daar de behoefte is gekomen.

**Interviewer:** Merk je dat die laag belangrijk is voor HR?

**Respondent:** Ja. Dat merk ik.

**Interviewer:** Waaraan merk je dat?

**Respondent:** Omdat ze dicht bij de werkvloer staan. Zij zien en beleven meer. Wat ik jammer vind, als ik naar mezelf kijk. Heb ik met >EVR< geen bila. Alleen met de bedrijfsleider. Maar ik denk, ook daar moet een bila voor komen. Om ook met die teamleider te helpen.

**Interviewer:** Van de teamleider krijg je dan toch weer waardevolle informatie binnen?

**Respondent:** Ja. En de helpt ook de teamleider ook in de ontwikkeling.

**Interviewer:** Heb je het idee dat er een reden is waarom je geen bila hebt met die teamleider?

**Respondent:** Die behoefte was er gewoon even niet. Die EVR is best een bijzondere EVR. Maar dit is wel iets wat ik later nog ga oppakken.

**Interviewer:** Op het to do lijstje.

**Respondent:** Ja. Het er met elkaar over hebben.

**Interviewer:** Ik denk dat ik er wel doorheen ben. Ik ben alleen nog wel benieuwd naar jouw visie op de functie van de teamleider voor nu en in de toekomst.

**Respondent:** Ik denk dat je de teamleider moet behouden. Het is een functie. Die heeft ook een soort van status. Als je een rol hebt, zoals die seniors OKE. Ja, ik geloof daar gewoon niet zo in.

**Interviewer:** Denk je dat seniors OKE wat gedaan krijgen?

**Respondent:** Weet ik niet. Sommige wel. Sommige worden ook alleen maar gebruikt voor het rooster. Dus dan denk ik 'is dat nou het doel van een senior?'. Nee, die zou zich dan als dagcoördinator moeten bezighouden weet je wel? Echt van die puntje op de werkvloer waar ook een teamleider eigenlijk te duur voor is of geen tijd voor heeft. Bijvoorbeeld als het verband op is dan moet een teamleider dat niet doen. Dat kan een senior dat prima doen. Dat soort praktische puntjes. En bijvoorbeeld bij >AFDELING< 'je tilt niet handig'. Die loopt mee over de werkvloer en kijk wat je ziet. Dat moet je een teamleider niet laten doen. Die moet er boven staan.

**Interviewer:** Ik weet niet of je zelf nog dingen toe zou willen voegen?

**Respondent:** Nee, eigenlijk niet. Ik hoop dat je er wat uit kan halen.

**Interviewer:** Ik denk dat dat wel goed moet komen. Eerst maar eens uitwerken.

**Respondent:** Het is lastig hoor.

**Interviewer:** Het is een moeilijke positie waar je als teamleider in zit als een soort buffertje die er tussen zit tussen bedrijfsleider en medewerkers?

**Respondent:** Ja. Precies. En het ligt ook echt wel aan de relatie de je hebt met je bedrijfsleider of hoofd. Heb je een back-up. Stel je voor ik ga naar mijn teamleider en het antwoord staat me niet aan. Ga ik dan weer een trapje hoger? Hoe gaat dat dan weer. Dat vind ik dan weer interessante dingen om over na te denken. Ga die bedrijfsleider dan ook terug naar de teamleider of is het van 'ik ben de baas, het gebeurt zoals ik zeg'. Geloof ik niet, maar het kan. Het was vroeger de bedoeling dat de teamleider een soort mini-bedrijfsleider zou worden en dat ze in een soort kweekvijver zaten op later bedrijfsleider te worden.

**Interviewer:** Heb je het idee dat dat nog het geval is?

**Respondent:** Nee.

**Interviewer:** Wordt de teamleider weggehouden van de belangrijkere activiteiten?

**Respondent:** Ik weet ook niet of de huidige teamleiders geschikt zijn als bedrijfsleider/hoofd.

**Interviewer:** De meeste teamleiders zijn hun huidige operationele functie overstegen toch?

**Respondent:** Ja. Die gaan het nu leren. En dan misschien de stap richting bedrijfsleider. Dat vraagt ook een bepaald niveau. En dan is teamleider het plafond en dat is prima. Maar we moeten dan ook niet verwachten dat iedere teamleider een bedrijfsleider gaat worden. Als dit het plafond is, is het prima. Als jij daar gelukkig van wordt.

**Interviewer:** Ligt er nog een verantwoordelijkheid bij HR om de teamleider naar bedrijfsleider te trekken?

**Respondent:** Phoe. Deels. Samen met de bedrijfsleider.

**Interviewer:** Maar dan ook op een bepaald moment kunnen zeggen 'dit gaan hem niet worden'?

**Respondent:** Neem bijvoorbeeld >EVR<. >BEDRIJFSLEIDER< gaat een stap doen omdat >BEDRIJFSLEIDER< minder gaat werken. >BEDRIJFSLEIDER< zegt 'ik heb die en die op het oog, want die doen het zo leuk'. Dan zeg ik 'nou dat kan maar: is die geschikt om leidinggevende te zijn, wil diegene het wel', dan ga je daarover vragen en samen een plan maken. Bijvoorbeeld eerst een papi doen om te kijken of diegene wel leidinggevende capaciteiten heeft en vertrouwd is met zijn eigen leiderschap en ga daarna samen met elkaar een plan maken. Trek daar de tijd voor uit. En wees dan ook gewoon eerlijk met elkaar als je het niet ziet zitten. Van alle kanten. Want jij kan het wel willen. Maar als je er na vier jaar achter komt dat je niet geschikt bent. Daar moeten we het samen over hebben. Als leidinggevenden en HR. Daar geloof ik in. Ik geloof in een heleboel.

*Interviewer lacht.*

**Respondent:** Een soort utopia dit altijd.

**Interviewer:** Thanks dat je mee wilde doen.

*Einde transcript.*

# Transcript Interview HR practitioner 2

**Duur interview: 71 minuten en 07 seconden**

**Functie: HR Adviseur**

**Afdeling(en) onder supervisie: Zorg en niet-zorg**

**Gender: vrouw**

**Werkervaring in functie: n.a.**

**Interviewer:** Misschien is het handig om dit interview te beginnen met het onderscheiden van seniors van teamleiders. Waar ligt volgens jou het onderscheid tussen seniors en teamleiders?

**Respondent:** Seniors OKE is destijds ontstaan om een aantal taken die zeg maar buiten je normale werk vallen, bijvoorbeeld doktersassistenten of verpleegkundigen, buiten de zorgtaken die je heb ook wat operationele taken, zoals het rooster op te pakken of iets met kwaliteit op de afdeling. Daar moet je natuurlijk ook protocollen voor schrijven en dingen in Idoc. Daar is die rol voor ontstaan, dat er tijd wordt vrijgemaakt om dat soort dingen te doen. En dat is ook een beetje de senior OKE rol, waar stond dat voor?, organisatie, kwaliteit en educatie, die drie. En daar kon je een van die drie onderdelen deed je dan of een combi daarvan. Dat was eerst senior OKE. Ook nog steeds zo, maar daar zie je ook steeds meer verschillen in. Net zoals bij teamleiders. Daar zitten ook verschillen in en dat moet ook haast wel denk ik want je heb op poli vaak geen teamleider, maar alleen een senior. Ik heb het dan even over >BEDRIJFSLEIDER< met >AFDELING<. Daar is gewoon heel duidelijk, er is geen teamleider maar daar hebben we senior >SENIOR< en die doet alle operationele dingen op de werkvloer qua coördinatie. Dat regelt >SENIOR< allemaal. >BEDRIJFSLEIDER< zit meer in het strategische stuk en >SENIOR< regelt gewoon dat alles op de werkvloer gewoon soepeltjes verloopt en die regelt alles met vrije dagen van mensen en roostering en alles om er omheen.

**Interviewer:** Wat maakt het verschil dat >BEDRIJFSLEIDER< er voor kies om een senior op de poli te zetten, terwijl bij >AFDELING< een teamleider op poli >EVR< staat?

**Respondent:** Dan zie je daar het verschil ontstaan. >BEDRIJFSLEIDER< is nog steeds direct leidinggevende van de mensen van >AFDELING< dat is >SENIOR< niet. Dus >SENIOR< heeft geen hiërarchische positie. Wel heel erg operationeel, maar >SENIOR< is geen leidinggevende van die mensen, waar >TEAMLEIDER< dat wel echt is. Die is wel echt verantwoordelijk voor de mensen en spreek mensen aan op hun functioneren. Die heeft ook jaargesprekken met ze en heeft ook een veel formelere rol daarin. Die voert ook de functioneringsgesprekken, de tussentijdse gesprekken met mensen. Ook als er een verbeterplan gemaakt moet worden is >TEAMLEIDER< degene die daarin de lead heeft. In het ziekteverzuim zie je dat >TEAMLEIDER< daar prominenter in is. Op alle punten is het echt iemand zijn leidinggevende, dat is >SENIOR< niet. Die voed >BEDRIJFSLEIDER< wel met heel veel informatie natuurlijk en die zit soms om te leren soms ook bij een jaargesprek, maar die heeft niet die verantwoordelijkheid. Daar zit het verschil in denk ik.

**Interviewer:** En als je kijkt naar de algemene werkzaamheden van een teamleider, wat zie jij diegene dan doen?

**Respondent:** Dat vind ik ook nogal verschillend. Als we kijken naar >TEAMLEIDER< die heeft nu aan >AFDELING< heel veel werk aan, we hebben een nieuwe oogarts, aan de roosters, zorgen dat er genoeg formatie is, dat de spreekuren goed verlopen. Daar moet >TEAMLEIDER< heel veel mee puzzelen. En op het gebied van kwaliteit en protocollen daar is >TEAMLEIDER< heel druk mee. En dat is bij anderen weer net anders. Daar ligt het zwaartepunt heel anders. Als ik naar >TEAMLEIDER< kijk, en ook bij >TEAMLEIDER< die werkt af en toe ook mee in het werk dus die moet af en toe weer als >FUNCTIE< werkzaamheden doen omdat dan gewoon de formatie krap is. Nou >TEAMLEIDER< heeft gewoon een veel grotere scope aan medewerkers en heeft op jaarbasis gewoon 60 jaargesprekken te voeren en die heeft al veel meer afstand genomen van de >AFDELING< en die gaat nu wel weer een beetje terug. Die is een beetje te weinig in het wit. Maar die zit nu veel meer al en wat rol dat je veel meer in het functioneren en het ziekteverzuim. Die met veel meer met dat soort dingen bezig is.

**Interviewer:** Wat is jou idee daarover? Teamleiders hebben een managementrol en een zorgrol...

**Respondent:** En een HR-rol.

**Interviewer:** ... Hoe vind je dat dat verdeeld zou moeten worden?

**Respondent:** We hebben destijds in de functieomschrijving staat er. Wat staat er? Een derde of zo?

**Interviewer:** Een kwart?

**Respondent:** Een kwart van je tijd. Ja, ik vind dat heel moeilijk. Ik weet niet of je daar perse een percentage aan moet hangen als teamleider.

**Interviewer:** Jij zou niet heel sec zeggen van 'ik zou het fijn vinden als iedere teamleider standaard een dag in de week zou meewerken'?

**Respondent:** Ik weet niet of dat een standaard dag in de week moet zijn. Ik denk wel dat ze mee moeten werken om. Juist als teamleider is het om die verbinding met die werkvloer is heel belangrijk. Een bedrijfsleider staat er veel verder vanaf. Om die verbinding te houden met de werkvloer denk ik dat voor een teamleider heel belangrijk is dat die meewerkt. Of dat iedereen dat dan een eenheidsworst moet zijn van een kwart, dat weet ik niet. Ik denk dat je dan heel erg moet kijken 'wat is voor mijn rol handig?'. En is dat dan een keer in de week is of twee keer in de week is of een keer in de drie weken. ik denk dat dat heel verschillend is. Ik denk ook bij een poli. Dat is veel kleinschaliger en zoals >TEAMLEIDER< die komt daar elke dag dus die spreekt ook de mensen daar. Dan zit je er ook tussenin. Dan is het misschien wat minder nodig om mee te werken dan op een >AFDELING< met 60 man waar je niet iedereen ziet elke dag, dan het wel handig als je af en toe even feeling houdt met de werkvloer en daar wat vaker aan het werk gaat. Ik vind ook soms een poli en een >AFDELING< niet te vergelijken met elkaar qua teamleiderschap.

**Interviewer:** Maar ze hebben we dezelfde fubes?

**Respondent:** Ja. Ja. Ja. En daar gaat die soms wel een beetje scheef ja dat je dan denk daar...

**Interviewer:** Zouden we dat dan uit elkaar moeten trekken?

**Respondent:** Iets van een functiedifferentiatie is wel eens geopperd in het verleden, dat heb ik nooit heel gek gevonden. Ook het salarisniveau lig ook zo ver uit elkaar. Nou hoeft het niet altijd zo te zijn dat je de mensen die je aanstuurt dat je daarop ongeveer op gelijke salarishoogte hoeft te zitten. Je hebt een groep van 35 mensen FWG 30 FWG 35 en op een >AFDELING< is dat 45 of hoger en daar zitten de mensen dan al vaak in met een gespecialiseerde verpleegfunctie dan blijf je in salaris ook nog gelijk en ga je weinig vooruit. En bij de poli is het zo dat het ineens een stap van 35 naar 55. Dus dan is de overstap ineens weer heel groot. Dus dan is ook de vraag 'is dat ook wel fair?'. Het ligt ook wel aan de verantwoordelijkheden want soms heeft een teamleider wel dezelfde verantwoordelijkheden als op een >AFDELING<. Want je doet soms en het jaargesprek. En goed, als we dan kijken naar facilitair, daar zitten mensen die ook weer een hele begroting doen.

**Interviewer:** Dat zou dan weer een stap verder zijn, nog breder?

**Respondent:** Dan heb je misschien een nog bredere. Daar zou je gewoon heel zorgvuldig naar moeten kijken volgens mij. Want dat is volgens mij gewoon heel lastig.

**Interviewer:** Enig idee waarom die. Want je zegt 'in het verleden was het al geopperd om die functiedifferentiatie misschien toe te passen', enig idee waarom het toen niet doorgevoerd is?

**Respondent:** Dat was toen heel erg van 'he, we willen het zo gelijk mogelijk voor iedereen'. Dat was volgens mij ook door de Raad van Bestuur uitgesproken dat dat gewoon gelijk moest zijn voor die teamleidersfunctie. Eigenlijk is het ook wel weer heel gek dat we dat hebben gezegd. Want als je naar facilitair kijkt en bij >AFDELING< hebben we ook allemaal verschillende FWG-niveaus.

**Interviewer:** Bij het facilitair is het heel erg gedifferentieerd?

**Respondent:** Precies. En dat is hier weer anders.

**Interviewer:** Waarom zou het bij een ondersteunende afdeling wel kunnen, maar niet voor de EVR's?

**Respondent:** Nou ik denk dus niet dat je weer moet. Dat is dus het verschil. Dat we elkaar even goed begrijpen. Ik denk niet dat we weer terug moeten. Zoals bij facilitair hebben we een teamleider Keuken, een teamleider Foodservice voor ieder apart is er een functiebeschrijving. Ik denk niet dat we daar weer naar terug moeten. Dus niet dat we moeten zeggen 'een teamleider >AFDELING< een teamleider Oog, teamleider'. We dat we moeten kijken naar 'zitten er qua verantwoordelijkheden, zitten daar differentiatie in?' en op basis daarvan generieke wel generieke functie. Dus het kan wel algemeen teamleider zijn, maar op verschillende niveaus. Dus meer een functiestraat, maar dan teamleiders op meerdere niveaus. En dan zou ik niet op basis van afdeling doen, niet weer allemaal aparte functieomschrijvingen maken. Daar willen we echt niet meer naar terug. *﻿Respondent lacht*﻿. Dat is natuurlijk, net als de functiefamilie verpleegkundige, dat wil willen we gewoon niet meer dat allemaal apart beschrijven.

**Interviewer:** Dus dan zou je een functiebeschrijvingstraat krijgen voor de poli en...

**Respondent:** Of echt alleen een functiestraat teamleider, en dan op verschillende niveaus. Ja. Dan zo ja.

**Interviewer:** Een lastig spel?

**Respondent:** Ja. Het is ook ingewikkeld. Dus daarom is uiteindelijk ook het besluit genomen van, denk ik, 'van ja, dat moet maar één functiebeschrijving worden op basis van'. Toch zie je in de praktijk dat daar weer heel anders mee wordt omgegaan. Ik weet ook niet of dat verkeerd is waarschijnlijk. Op elk moment en op elke plek is er weer iets anders nodig. Dat hangt dus ook heel sterk af van tijdsbestek af. Dat zie je ook heel sterk bij >AFDELING<. Daar is nu tijd over en wordt er heel dedicated tijd voor kwaliteit en indicatoren, daar gaat even heel veel tijd naartoe. En op een ander moment heb je misschien op een ander vlak de nadruk. Dus dat blijft ook lastig.

**Interviewer:** Maar we kunnen nog wel steeds stellen dat het gat tussen teamleiders groot is?

**Respondent:** Ja. Ook als je alleen al de span of control. Alleen daar zit al een heel groot verschil in. Dus ja.

**Interviewer:** Het is lastig?

**Respondent:** Ja. *﻿Respondent lacht*﻿. Dat zie je ook wel in de FWG zie je dat ook wel terug in 'hoeveel mensen stuur je aan?'. Bij tien tot vijftien FTE zie je daar natuurlijk ook al weer de zwaarte in terugkomen. En dat is voor iedereen in 55 geldt dat, is die zwaarte voor iedereen zo, terwijl die in de praktijk ook niet zo is. Dus dat is wel gek.

**Interviewer:** Als we eens kijken naar de HR-taken die bij een teamleider liggen, dan zou dit er bijvoorbeeld uit kunnen komen qua onderwerpen. Zou wij het lijstje eens door kunnen lopen en waarvan je denkt 'dat zie ik die teamleiders' wel doen, dat je dat dan aangeeft en daar een voorbeeld van geeft van 'zo vervullen ze het'?

**Respondent:** Ja. Werving. Daar vervullen ze zeker een rol in. Werving, selectie, en introductie.

**Interviewer:** Hoe zie je die rol voor je?

**Respondent:** Ze zitten bij sollicitatiegesprekken, en ze selecteren, wel altijd met collega's, een kandidaat. En met die introductie houden zij zich ook bezig op de afdeling van 'dan wordt er een mentor toegewezen' of een introductieprogrammatje. Dat zijn wel dingen waar ze zich mee bezig houden. Ik zit even te denken of ze dat allemaal doen. Denk het haast wel.

**Interviewer:** Hier zit niet veel verschil tussen?

**Respondent:** Ik denk dat ze hier wel allemaal een rol in hebben. Bijhouden van personeelsbestanden. Wat bedoel je daarmee?

**Interviewer:** Als er bijvoorbeeld aanvragen binnen komen of dingen gemuteerd moeten worden?

**Respondent:** Nee. Zij zitten nog niet. Nou. Alhoewel. In Youforce krijgen we natuurlijk. De bedrijfsleider kan altijd doorzetten omdat zij niet gemachtigd zijn of ge-...

**Interviewer:** Geautoriseerd?

**Respondent:** Geautoriseerd zijn om uiteindelijk zelf helemaal die mutatie helemaal door te zetten. Ik zit even te denken. Volgens mij doet >TEAMLEIDER< wel mutaties indienen dat die vervolgens door naar >BEDRIJFSLEIDER< gezet worden. Of doen ze nou helemaal geen mutaties? Wel ziekteverzuim volgens mij. Een verzuimsignaal. Nou, ik weet nou niet zelf of zij ook mutaties doen. Ik weet niet of zij daar bevoegdheden voor hebben om mutaties in te voeren. Alles wat wij aan mutaties krijgen is van >BEDRIJFSLEIDER<.

**Interviewer:** Is het makkelijk als zij zelf mutaties kunnen doen?

**Respondent:** Ja.

**Interviewer:** Waarom zou het het makkelijker maken?

**Respondent:** Ik denk dat het ontlastend is voor sowieso de bedrijfsleider en dat zij sowieso degene zijn die daarvoor input moeten geven aan de bedrijfsleider. Dus ja. Dan zouden ze dat net zo goed gewoon zelf kunnen doen. Het is niet altijd zo hoor, maar in heel veel gevallen gaat het toch ook met de input van de teamleider en stemmen ze het met elkaar af. En soms is het dan net zo handig dat de teamleider het zelf invoert. In die zin zou het het sneller maken. Plannen van training en ontwikkelingsbehoeften. Dat is meer de bedrijfsleider die daar. Dat merk ik ook. Wij hebben nu de SPO gesprekken en dan is het vooral de gesprekken met de bedrijfsleider daarover en de opleidingsbegroting wordt ook met de bedrijfsleider afgestemd.

**Interviewer:** SPO staat voor strategische personeelsontwikelling? *﻿Interviewer lacht.*

*﻿* **Respondent:** *Respondent lacht.*﻿Ja, precies. Dus dan is het ook iets van 'dan kijk je naar de toekomst en wat denk je daarvoor nodig te hebben?'.

**Interviewer:** Echt een bedrijfsleideronderwerp?

**Respondent:** Ja. Is meer een bedrijfsleideronderwerp. Het geven van formele training bedoel je dan...

**Interviewer:** Dat ze echt voor de groep gaan staan en iets uitleggen over bijvoorbeeld 'we krijgen nu dit binnen'. Als er bijvoorbeeld een nieuwe wet zou komen, zoals Wet Werk en Zekerheid. Dat zij dan zoiets hebben van 'ik ga even voor de groep staan en dat delen wij dan met de groep'.

**Respondent:** Dan denk ik meer aan formele training in zorginhoudelijke onderwerpen misschien. Dat weet ik niet zo goed. Kijk, coaching en ondersteuning doen ze natuurlijk wel. Daar zijn zij de aangewezen personen voor die ook wel tussentijds de mensen aangaan en het aanspreekpunt voor de mensen zijn. Als medewerker en teamleider er niet uitkomen kunnen ze altijd nog de bedrijfsleider inschakelen. Wat is soms ook wel eens zie gebeuren dat bijvoorbeeld bij >TEAMLEIDER< op >AFDELING< die heeft dan een tijdje met iemand gesproken in een verbetertraject en dan wil het niet verder komen en dan stagneert het en dan schalen ze op naar een >BEDRIJFSLEIDER< om de boel weer recht te trekken.

**Interviewer:** Is dat een efficiënte methode naar jou idee? Werkt het?

**Respondent:** Ja. Ik denk wel dat het werk. Het is ook prettig voor de medewerkers dat ze altijd nog bij >BEDRIJFSLEIDER< terecht kunnen, maar ik merk wel dat. Uh. Ik denk dat het in eerste instantie goed is dat je eerst het gesprek aangaat met je direct-leidinggevende. Vanuit daar moet het gesprek altijd plaatsvinden en zou het helpen als >BEDRIJFSLEIDER< erbij zit om beide partijen een beetje te ondersteunen op een aantal punten en tot nieuwe inzichten te laten komen. Dat werkt wel. Ik heb ook wel gezien, en dat ervaart >BEDRIJFSLEIDER< dan zelf, dat niet het hele verhaal van A tot Z meekrijgen en ergens halverwege moeten bijspringen en ergens achterwege denkt van 'ik had soms eerder moeten aanhaken', dat is dus wat moeilijk he voor >BEDRIJFSLEIDER<.

**Interviewer:** Wat is mijn rol?

**Respondent:** Wat is mijn rol. Maar nu ook merkt >BEDRIJFSLEIDER< met de medewerkerstevredenheidsonderzoeken dat betrokkenheid van >BEDRIJFSLEIDER< en de zichtbaarheid van >BEDRIJFSLEIDER< van 'oké, je zit wel op de afdeling, maar je zit altijd in gesprek'. Dat klopt ook. >BEDRIJFSLEIDER< zit ook op een ander niveau dan dat >TEAMLEIDER< zit. >TEAMLEIDER< begeeft zich natuurlijk ook meer op die werkvloer, dus dat is voor >BEDRIJFSLEIDER< een soort van 'oh, misschien moet ik mij weer wat vaker laten zien bij de medewerkers en wat vaker zichtbaar op het teamoverleg'. Dus dat is het lastige ook gelijk van. Maar ook weer het stukje verwachtingsmanagement dat je richting medewerkers moet doen 'wat kunnen ze nu verwachten van een bedrijfsleider en wat van een teamleider?'. Daar moet je natuurlijk ook altijd heel helder in zijn.

**Interviewer:** Heb je het idee dat het voor medewerkers goed duidelijk is?

**Respondent:** Ik denk niet altijd. Daar zijn dan soms wel eens vragen over en daar wordt op het MO. Daar zijn dan wel eens vragen over. En dat is dan nu op het MO dat wordt dan weer besproken.

**Interviewer:** Waar vallen ze dan over? Over wat voor een onderwerpen?

**Respondent:** Inderdaad voor wat voor vragen moeten we dan bij wie zijn. En niet specifiek overigens bij >AFDELING< maar toch ook weer bij >AFDELING< kwam dat naar boven. Dat ze dus ook aangeven 'soms vinden we het gewoon lastig met wat we nou bij wie moeten zijn'. >TEAMLEIDER< is natuurlijk onze teamleider. Dat is ook nog niet zo lang overigens. Dus daarin moet je de weg vinden met elkaar. Dus dat is wel, en dat merk je dan merken hun ook weer van 'oh ja, het is ook weer iets wat we even aandacht moeten geven en met jullie moeten communiceren'. En gaandeweg ga je aan de slag met elkaar en kom je daarachter.

**Interviewer:** Denk je dat dat vaker het geval is als een nieuwe teamleider aangenomen wordt, dat er dus zo een periode is van afstemming 'wat doe jij, wat doe ik'. Of is het eerder als een nieuwe teamleider komt dat we weten wat we van diegene mogen verwachten?

**Respondent:** Ik denk dat dat. In het geval van >BEDRIJFSLEIDER< en >TEAMLEIDER< hebben we dat echt omschreven van 'wat worden jouw resultaatgebieden voor >TEAMLEIDER<'. Maar in hoeverre dat nou met de medewerkers gedeeld wordt en in hoeverre zij daar dan weer in meegenomen worden, dat weet ik dan weer niet. Dan denk ik van, dat is dan wat ik afleid van wat ik nu hoor, van 'oh ja, is dat dan weer even vergeten?' of is dat in het proces gegaan van 'dat is vanzelfsprekend, dat snappen we wel'. Weet je wel?

**Interviewer:** Zo'n rolverdeling komt natuurlijk ook niet uit de lucht vallen, dat is iets wat je samen doet?

**Respondent:** Ja. Precies. Wat je samen vorm wil geven. Daar wordt over nagedacht.

**Interviewer:** Een stukje mentoren heb je aangegeven. Dat zit een beetje bij het coachen in?

**Respondent:** Ja. Klopt. Ja. Functionerings en beoordelingsgesprekken. Ja. Het jaargesprek. En dat doen ze allemaal. Ik zit even te denken >TEAMLEIDER< die. Die zal daar ook mee. Die doet dat ook. Ja, volgens mijn doen ze dat allemaal.

**Interviewer:** Wordt er dan nog een afscheiding gemaakt tussen moeilijkere jaargesprekken en makkelijker jaargesprekken?

**Respondent:** Ja. Dat zie je ook. Ze hebben er eerst nog een paar samen gedaan. Dat is ook het groeimodel waar je natuurlijk een beetje. >BEDRIJFSLEIDER< die heeft daar wel ervaring mee. Die doet dat al jaren. Dus die heeft ook gezegd 'we doen er eerst een paar samen'. En soms wat je zeg, de iets ingewikkeldere, ja, doen ze dan ook wel eens samen of daar wordt wel eens van gezegd van 'jij doet deze dit keer, deze heb ik al een paar keer gedaan en ik heb het idee dat ik vastloop, nou dan kan jij hem een volgende keer doen'. Dus daar wordt wel afstemming voor gezorgd.

**Interviewer:** Het akkoord geven op ontwikkelingsplannen, dat ligt dan echt bij de bedrijfsleider?

**Respondent:** Ja. Ja. Echt voor het definitieve akkoord. Als je ook altijd kijk naar studieformulieren dan moet daar het akkoord van de bedrijfsleider onder staan. Dat heeft te maken met die procuratie en dat zij voor het budget verantwoordelijk zijn.

**Interviewer:** Dan ben ik wel nieuwsgierig naar het volgende onderwerp, het disciplineren en corrigeren van medewerkers. In hoeverre ligt dat een rol voor teamleiders?

**Respondent:** Ik denk het zeker wel. Dat is wel iets. Zij spreken en zien die mensen ook in het dagelijkse werk werken dus zij zijn ook de eerste die die mensen aanspreken op wat ze zien. Dus dat gebeurt zeker wel.

**Interviewer:** Ook wel iets dat we van een teamleider zouden mogen verwachten, dat ze daar als teamleider mee bezig zijn?

**Respondent:** Ja. Omdat dat ook iets is dat sowieso in het jaargesprek naar voren komt. Maar goed als je iemand verrast bij het jaargesprek met het feit dat het niet goed of dat er dingen niet goed gaan. Dus tussendoor worden mensen daar ook op aangesproken. Dus ja dat vind ik echt wel een rol van teamleider.

**Interviewer:** Zouden zij dit dan op eigen houtje kunnen doen of is het ook iets waar de bedrijfsleider in meegenomen wordt?

**Respondent:** Nou dat ligt eraan. Als het echt gaat om discipline en echt een disciplinaire maatregel, zoiets als een officiële waarschuwing, dan is het iets waar de bedrijfsleider echt in meegenomen moet worden en wij, wij ook vaak zeggen van 'betrek HR daar ook in'. Wij hoeven niet bij het gesprek aanwezig te perse te zijn. Wij weten wel wat de regels daaromtrent zijn, en wat je boodschap allemaal moet bevatten en waar je iemand op gewezen moet hebben. Dus dan is het wel. Vind ik wel dat de bedrijfsleider daarin betrokken moet zijn. Dat dat gaat gebeuren. Ik zeg ook altijd 'ga met z'n tweeën altijd een officiële waarschuwing, doe dat nooit in je eentje', want uiteindelijk gaat het ook om hoor en wederhoor overigens, maar dan heb je wel altijd een getuige van wat iemand gezegd heeft. Dus dan gaat het echt om zware. Maar als het om corrigeren gaat over het werkinhoudelijke dingen of het gedrag dat iemand vertoond, dat is anders denk ik.

**Interviewer:** Een stukje tucht noemde je. Een stukje arbeidsrecht.

**Respondent:** Ja. Dan komt de bedrijfsleider erbij.

**Interviewer:** En als het over klachten gaat?

**Respondent:** Klachten. Volgens mij behandelen de klachten de teamleiders gewoon. Van >TEAMLEIDER< weet ik zeker dat >TEAMLEIDER< zich daar mee bezighoudt. Van >TEAMLEIDER< heb ik daar ook wel eens over gehoord. Volgens is dat wel iets dat teamleiders over het algemeen wel kan oppakken. Als het vaak iets is, iets een klacht is van een patiënt of iets dat op de werkvloer gebeurt is dan is het even horen van de medewerker 'hoe is het precies gegaan?' en dan even kijken 'hoe gaan we dat nu doen richting de patiënt?'. En soms heeft >TEAMLEIDER< ook wel eens gezegd van 'medewerker misschien moet jij zelf even contact opnemen met de patiënt, dat is veel krachtiger'. Dat gebeurt. Dat pakt de teamleider meestal op. In ieder geval. Dat hangt ook weer van de klacht af. Soms is het nodig dat de bedrijfsleider ook betrokken wordt omdat het gaat over bredere dingen dan. Soms zijn klachten natuurlijk, heeft het invloed op de hele afdeling. Sommige klachten wel. En als je daar organisatorisch iets mee wil, dat je denkt van 'we moeten iets in het proces veranderen', dan komt de bedrijfsleider er wel.

**Interviewer:** Een stukje absentiemanagement noemde jij net al. Krijgen zij dan ook de ziekmelding binnen?

**Respondent:** Ja. Ze melden zich ook wel vaak bij de teamleider ziek en die onderhoud ook het contact met de medewerker.

**Interviewer:** Waardering geven, dat klinkt heel breed. Hoe zie jij het voor je dat een teamleider waardering geeft?

**Respondent:** Ik denk dat het ook wel degene is die mensen moet enthousiasmeren en de waardering moet geven omdat de bedrijfsleider toch iets verder weg zit, en niet dagelijks daar is. Dus ik denk wel zeker dat het bij een teamleider past ja. Dat is natuurlijk ook weer iets dat in het jaargesprek heel erg terugkomt van dingen waar mensen goed in zijn en een schouderklopje voor verdienen. Dus dat hoort er zeker bij ja.

**Interviewer:** Ik hoorde een stukje complimenten? Kan het ook verder gaan dan alleen het geven van complimenten?

**Respondent:** Oh je bedoelt een gratificatie?

**Interviewer:** Bijvoorbeeld. Of zijn er nog andere opties die die teamleiders gebruiken?

**Respondent:** Nee. Waar zit jij dan aan te denken?

**Interviewer:** Gratificatie is dan een mogelijkheid...

**Respondent:** Maar daar zijn zij dan weer niet bevoegd voor. Dat is wel echt iets wat de teamleider...

**Interviewer:** Dus die valt af?

**Respondent:** Die valt af.

**Interviewer:** Tja. Je zou complimenten kunnen geven, wat zou je nog meer kunnen doen? Bijvoorbeeld een bos bloemen of een paar klompen? Gebeurt dat?

**Respondent:** Ja. Ik denk dat dat wel. Ik denk zeker wel dat teamleiders zo attent zijn. Ik denk dan bijvoorbeeld aan >TEAMLEIDER< die is, dat is ook heel er persoonsafhankelijk, die is heel erg juist van het attente en het denken aan de mensen en zo ja, een bloemetje. Dus ja ik denk dat >TEAMLEIDER< dat bijvoorbeeld doet.

**Interviewer:** Denk je dat daar ruimte voor is voor dat soort dingen, of is dit heel uniek als iemand een bloemetje zou krijgen?

**Respondent:** Die ruimte is er. Als het maar niet over exorbitante dingen gaat. Een bloemetje of een kaartje, zeker.

**Interviewer:** Als het een beetje normaal blijft...

**Respondent:** Is hij er. Zeker weten.

**Interviewer:** In termen van communicatie. Hoe zie je daarin de rol van teamleider. Van zowel medewerker naar bedrijfsleider als misschien andersom?

**Respondent:** Kan je nog wat specifieker zijn?

**Interviewer:** Ik kan me voorstellen dat de teamleider echt een soort doorgeefluik is. Dus dat een teamleider zowel dingen van de medewerker naar bedrijfsleider communiceert als van bedrijfsleider naar een medewerker communiceert?

**Respondent:** Ja. Dat is wel waar. Wel waar. En soms is dat wel een worsteling natuurlijk omdat je dingen vanuit bedrijfsleider uiteindelijk moet communiceren aan de werkvloer en dat je ook weet waar de werkvloer mee worstel. Maar ik vind opzicht wel dat bedrijfsleiders en teamleiders daar best wel over op een lijn zitten. Dus proberen daar wel een front in te vormen zal ik maar zeggen, dat daar geen ruis tussen komt. Dat wil je niet.

**Interviewer:** Dan kun je tegen elkaar uitgespeeld worden?

**Respondent:** Ja. Dat zie ik namelijk wel gebeuren. Dat gebeurt natuurlijk dan wel. Dan is het van >TEAMLEIDER< zei dit en >BEDRIJFSLEIDER< zei dat. Zo gaat dat dan. Dat probeer je natuurlijk zoveel mogelijk te voorkomen dat het uit een mond. Dat dat goed zit. Dat is wel. Ik denk dat het bij de meeste wel goed zit je. Ik zie bij de >AFDELING< nog wel, dat is >TEAMLEIDER< nog echt wel meewerkend voorman. Dus die moet nog in zijn rol van teamleider groeien en wat meer. Dat is het lastige ook. Je bent dichter bij de mensen als een >TEAMLEIDER< die zit echt in het team en ook letterlijk als werkplek. Dan is het ook lastig van als er dingen spelen, dat je je dan niet laat leiden door de afdeling maar dat je zelf een zekere mate van afstand voor jezelf creëren naar de mensen toe. Omdat je ook wel iets vindt van wat een >HOOFD< of een >MANAGER< vind, maar dat kan je niet constant ventileren. Je eigen mening...

**Interviewer:** Je moet er echt boven gaan staan?

**Respondent:** Je eigen mening moet je voor jezelf houden en ook voor de sake van het team. Het is natuurlijk een best wel lastige rol. Als >TEAMLEIDER< kan ik mij dat ook voorstellen. Die was eerst meewerkend voorman en als je dan opeens teamleider bent, moet je daar afstand van gaan nemen.

**Interviewer:** Blijft >TEAMLEIDER< ook op de afdeling werken of gaat >TEAMLEIDER< ergens een kantoortje krijgen?

**Respondent:** Nee. Dat weet ik niet wat daar de plannen van zijn. Maar ik merk wel, ik denk dat dat lastig is. Ik zie dat ook... Wat wilde ik nou zeggen? ... Ik weet niet meer wat ik wilde zeggen. Maar dat is dan wel dat je denkt van. Oh ja, mijn andere afdeling, daar zal ik niet te veel over uitwijden, als een bedrijfsleider en teamleider niet op een lijn zitten dan praat je niet met een mond naar de medewerkers en gaan de medewerkers aan de haal met de ene vind en wat de andere vind.

**Interviewer:** Dan krijg je een tweesplitsing?

**Respondent:** Ja. En dat is niet fijn. Als je dan gaat roepen dat je het ook niet eens bent met wat jij vind als bedrijfsleider, ja, dan geeft dat geen positief beeld van jou omdat jij als eerste iets zegt, maar ook over iets zegt dan de ander.

**Interviewer:** Dus echt een unit vormen?

**Respondent:** Ja. Want anders komt het bij de medewerkers ook niet goed over. Dus dat is met die communicatie net zo.

**Interviewer:** Heb je het idee dat als het aankomt op het aanhoren van suggesties, dat teamleiders gebruik maken van de input van medewerkers?

**Respondent:** Ja. Denk het wel.

**Interviewer:** Door wat voor manier zijn zij van die input op de hoogte.

**Respondent:** De medewerker zelf?

**Interviewer:** Nee. De teamleider. Heb jij een idee over hoe een teamleider op de hoogte zijn van wat medewerkers denken en vinden?

**Respondent:** Dat verzamelen ze sowieso in het jaargesprek. Een onderdeel van het jaargesprek is ook van 'dingen om aan te geven, aan te merken, ook op mij als leidinggevende'. Dat is ook een plek waar ze er naar vragen. En in het MO wordt er weer expliciet naar gevraagd. Ik weet niet of ze het veel tussendoor doen.

**Interviewer:** Het zijn van die momenten die ze pakken?

**Respondent:** Ja. Zoals het jaargesprek he? Precies. Ja. Ik denk wel. Ik denk dat het dan wel expliciet terug van, als ze dan iets terughoren van 'oh wat gek want ik heb in het jaargesprek, he wat gek heb ik daar niemand over gehoord?'. Weet je wel? Ik denk niet dat ze tussentijds daar de thermometer in hangen van. Maar dan over eigen functioneren he? Wel over andere dingen.

**Interviewer:** Heb jij ook het idee dat zij nog verantwoordelijk zijn voor het teamwerk op de afdeling en zorgen dat het team goed met elkaar samenwerkt?

**Respondent:** Uhm. ... Dat is ook weer heel verschillend denk ik. Dat is met als die onderwerpen vind ik wel lastig hoor. Dat ligt er ook maar net aan wat voor team je hebt natuurlijk. Je merkt gewoon, sommige teams zijn echt zelfsturende teams. Die behandel je dan ook echt anders dan teams waar dat veel meer anders is. Die hebben veel meer directe aansturing nodig.

**Interviewer:** Wat zou een verschil kunnen zijn tussen een team dat wel zelfsturend is en een team dat niet zelfsturend is?

**Respondent:** Ik denk dat je bij een zelfsturend team, dan faciliteer je veel meer he. Dan ga je veel meer zorgen dat de randvoorwaarden, dat ze hun werk op een goede wijze, dan ga je veel meer kijken. Ik weet bijvoorbeeld wel. >TEAMLEIDER< dan, die is heel erg bezig juist daarmee. >TEAMLEIDER< trok dingen best wel naar zichzelf toe. Voelde zich daar verantwoordelijk voor. Nu zie je wel een verschuiving. Het zijn zorgprofessionals die willen zelf ook dingen ondernemen. Die weten zelf soms ook niet hoe ze een project moeten aanpakken, van 'oké, ik geef jullie de tijd en de dingen die jullie daarvoor nodig hebben, ik organiseer dat, maar jullie mogen daar zelf de inhoud aan geven'. Weet je? Dus daarin ben gewoon veel meer faciliteren. Dan neem je even een stapje terug. Dat heb ik dan heel mooi gezien bij >AFDELING< dat >TEAMLEIDER< die beweging maakt. Bij >AFDELING<, >TEAMLEIDER< is nog niet op dat punt beland. Die is nog veel meer met dat operationeel in het team bezig, omdat er gewoon nog heel veel te doen is en omdat het een net-geformeerd team is, dat moet je weer vanaf nul opbouwen, dat heb je daar, dan zit je daar veel meer operationeel in.

**Interviewer:** Die pakken meer een kartrekkersrol?

**Respondent:** Ja. Die zijn veel meer bezig daar te coördineren. Dus dat ligt heel er aan het team. En >TEAMLEIDER< ook, die werkt ook nog best wel heel veel mee in het werk. Dat is dan ook van, die coördinatierol die zou >TEAMLEIDER< veel meer zelf moeten oppakken. Niet dat >TEAMLEIDER< al het werk zelf doet...

**Interviewer:** Maar meer afgeven?

**Respondent:** Ja. Gaat zorgen dat het werk juist om de meest efficiënte wijze wordt ingevuld. Maar dat is ook groeien in je functie en ook waar je team aan toe is. Die moet je daar heel goed in meenemen.

**Interviewer:** Merk je ook dat teamleiders bezig zijn met onderwerpen als 'kwaliteit van arbeid, duurzame inzetbaarheid, veiligheid?'.

**Respondent:** Ja. Dat is denk ik nog te weinig dan. Het is dan net die rol. Ze zijn er wel mee bezig en zijn komen eens in de tijd bij elkaar, de teamleider, en delen daar onderwerpen met elkaar, en daar is toen ook de vraag uit gekomen van 'willen we iets met die duurzame inzetbaarheid?'

**Interviewer:** Kwam vanuit hun die vraag?

**Respondent:** Ja. Vanuit die groep toen. En dan ga je wel doorvragen 'wat bedoel je dan precies?'. Maar ik denk dat het daar dan ook bij blijft. Is niet heel actief daar iets met dat onderwerp...

**Interviewer:** Wordt niet heel concreet gemaakt?

**Respondent:** Nee. Dat idee heb ik een beetje. En dan is het ook meer ook van. Uh. Volgens mij ook altijd van 'ga het gesprek met je medewerker daarover aan'. Voor iedereen is het vaak maatwerk en is daar een andere oplossing voor nodig. Dus ja. Ik weet niet of ze met dat soort thema's echt aan de slag zijn.

**Interviewer:** Maar je bent er wel voor jezelf uit dat dit thema's zijn die teamleiders bezig zouden moeten houden?

**Respondent:** Ja. Ja, maar het houdt ons ook bezig hoor. Het is ook dat HR daarmee bezig is dat wij hun daarin kunnen faciliteren en waarmee, wat is dan de behoefte? Maar je merkte wel dat de vragen die er kwamen, die waren niet heel specifiek en zelf ook niet heel erg verder over nagedacht. Dus dat is nog wel iets en staat ook in het strategische personeelsontwikkeling. Het stukje kijken naar de toekomst hebben we gemerkt, dat is voor de bedrijfsleiders al heel lastig, dus dat is ook iets waarin we met elkaar een weg aan het vinden zijn. Dus laat staan de teamleider. Het is nog wel een overgang. De bedrijfsleiders zijn het al niet gewend, sommige wel hoor, dus daarin is het al een hele diverse club, andere nog helemaal niet. Dat is voor teamleiders net zo.

**Interviewer:** Als we dan eens kijken naar de beslissingsbevoegdheid van een teamleider, wat voor besluiten zouden zij dan mogen nemen, of juist niet mogen nemen?

**Respondent:** Dat vind ik wel een moeilijke vraag. Welke besluiten zouden ze mogen nemen en welke niet? Ja...

**Interviewer:** Of onderwerpen waarvan je zoiets hebt 'dat ligt sowieso niet bij een teamleider?'.

**Respondent:** Dat is alles wat met de begroting en met geld te maken heeft en de formatie. Daar is de bedrijfsleider in de lead en eindverantwoordelijk. Wat ook terecht is.

**Interviewer:** Waarom?

**Respondent:** Omdat die uiteindelijk ook eindverantwoordelijk is en draagt daar ook de eindverantwoordelijkheid voor. Dus ik denk dat dat wel terecht is. Dat heeft natuurlijk ook wel weer een doorwerking op het personeel.

**Interviewer:** Ligt er financiële bevoegdheid bij de teamleider?

**Respondent:** Ja. Uh. Nee. Sorry, ik dacht je het over de bedrijfsleider had. Volgens mij is daar wel wat over gevonden ja. Waar zij voor mogen tekenen. Tot een bepaald bedrag of zo.

**Interviewer:** Heb je ook het idee dat er geld wordt vrijgemaakt door teamleiders om in het team te investeren? Ik bedoel dan niet perse opleidingen, maar juist dingen zoals een bloemetje of iets anders?

**Respondent:** Nee. Daar is niet perse een potje voor of zo. Maar ik denk wel dat dat iets is wat heel erg in overleg gaat met de bedrijfsleider en waarvan de bedrijfsleider en de teamleider vaak wel op een lijn zitten en daar iets samen over hebben afgesproken. Of het ligt heel erg aan de bedrijfsleider die zegt 'ik heb dat echt gedelegeerd aan mijn teamleider en ik vind het prima, die krijgt van mij alle ruimte om daarmee te doen wat hij of zij wil'. Het zijn dan wel echt de bloementjes en de kaartjes hoor. Het gaat niet om de gratificaties dat ik wel weer echt bij de bedrijfsleider horen. Ik bedoel, zodra een bedrijfsleider daar geen zicht meer op heeft en een teamleider allemaal dingen gaat bedenken, maar je moet wel tekenen voor een begroting, en wel moet weten wat er in en uit jouw EVR gaat en zelf verantwoordelijk voor bent, dan vind ik dat je dat niet moet doen. Dat moet je dan niet willen.

**Interviewer:** Heb jij ook nog een mening over wanneer een bedrijfsleider goed werk aflevert. Wanneer zo iemand naar behoren functioneert?

**Respondent:** Wanneer een bedrijfsleider naar behoren functioneert?

**Interviewer:** Komt er dan iets in je op. Ik vind dat, dat, dat.

**Respondent:** Ja. Als je goed naar je mensen weet te luisteren, als je goed weet wat er speel, als je goed weet waar de ontwikkelingsmogelijkheden van mensen zitten en dat je mensen op de juiste plek inzet. En ook dat je inderdaad kijkt van 'waar gaat mijn EVR naartoe, wat is de toekomst?'. Ook wel het stukje, dat zie je ook niet iedereen doen, 'wat is het bestaansrecht van mijn EVR en waar kan ik nog in de markt'. Meer het strategische denken van 'wat is mijn markt, waar moet ik mij gaan begeven en waar moet ik mij juist niet op begeven?'. Dat ze daar over na gaan denken en, zoals ik zei, goed naar de ontwikkelingsmogelijkheden van medewerkers kijkt en hoe zet je die nu het beste in? Ik denk dat dat het belangrijkste is je.

**Interviewer:** Denk je dat teamleiders ook nog over bepaalde kennis moeten beschikken om goed te kunnen functioneren?

**Respondent:** Bepaalde kennis. Ja. Ik denk wel, gespreksvaardigheden en het hele financiële begroting van je EVR. Het lijkt mij wel goed dat je daar inzicht in hebt. Dat je dat begrijpt. Dat je ook weet hoe je rapportages moet lezen en weet van 'hoe staat de EVR er voor', maar ook een stukje zachte kant dus echt in termen van gespreksvaardigheden en hoe je met mensen in gesprek gaat. Hoe doe je dat met een jaargesprek, hoe doe je dat met ziekteverzuim. Ja, ik denk dat dat wel belangrijk is. En opzicht hebben we de mensen daarin ook wel ondersteunt en denken we daar ook over na van 'wat kan daar nog meer in helpen?'.

**Interviewer:** Heb je het idee dat teamleiders ook nog over bepaalde HR-kennis moeten beschikken om hun werk te kunnen doen?

**Respondent:** Nou ja. Je merkt wel aan het begin dat teamleiders die nog nooit leidinggevende zijn geweest, en dat heb ik in het begin ook wel een aantal keer gehad, die begeleid je in het begin veel intensiever omdat ze gewoon veel dingen niet weten. Dat gaat dan over praktische dingen, over dingen die gaan over Wet Verbetering Poortwachter, het ziekteproces, of hoe werkt het nu precies met verbetertrajecten, hoe werkt het nu met een officiële waarschuwing? Of hoe werkt het met de begeleiding of met de studieovereenkomsten? Met al dat soort praktische vragen, HR-vragen, het je in het begin heel veel mee te maken. Dus dan merk je dat ze die kennis echt ontberen en dat ze die heel erg bij jou op komen doen?

**Interviewer:** Hoe ga je daar mee om?

**Respondent:** Dat vind ik niet erg. Ik denk dat dat in het begin heel logisch is dat ze je daar veel meer voor consulteren en dat je ziet dat in de tijd ook wel afnemen. Dus dat is alleen maar goed. En soms. Je kunt je ook afvragen 'moet ik daar dan wat mee', kijk wij doen vanuit HR hebben wij dan de roadshow gedaan met vooral de wet- en regelgeving op de hoogte te houden, want je merkt gewoon dat dingen houden ze niet bij. Die dingen die wijzigen en op gebied van wetgeving. Ik weet ook niet of je dat van ze moet verwachten, dat is hun rol ook niet denk ik.

**Interviewer:** Dat is niet hun specialisatie?

**Respondent:** Dat is niet hun specialisatie. Dat ze die bij ons komen halen of dat wij die bij hun komen brengen. Dus in dat. I merk wel als het gaat over werving en selectie, dan hebben wij ook wel met elkaar er over nagedacht van 'moeten we ze daar niet meer in equiperen, dat zij beter die sollicitatiegesprekken in gaan. Maar daar zie je ook als je er een paar keer bij gezet hebt dat dat beter gaat. Dan horen zij de vragen die jij stelt en dat kan ik ook doen, dat helpt dan al. Want het is ook niet zo dat wij bij elk gesprek willen gaan zitten. Maar het is vooral voor hun willen overbrengen van 'hoe kun je nu zo een gesprek doen en wat zijn de handigheidjes daarin?'. Zodat je vooral het gesprek zelf kunt doen. Dat geldt ook voor gespreks en verslaglegging. Vooral in verbetertrajecten gaat daar zaten wij ook heel veel bij. Dat merkte wij ook dat slokte wel veel tijd weg. Die gespreksverslagen schrijven en zo. Is het niet veel handiger als zij die gespreksverslagen schrijven, en dat wij meelezen en daar input op geven. Dan is het ook al heel gauw 'oh, zo moet ik hem insteken'.

**Interviewer:** Dus echt zelfredzaam maken?

**Respondent:** Ja echt die zelfredzaamheid in plaats van tekens maar over nemen. Want voor je het weet heb je allemaal taken die tot de lijn behoren, terwijl de eerste verantwoordelijkheid toch echt wel bij hun ligt om dat gesprek te voeren. Dat is wel iets waar wij mee bezig zijn. Dat is ook wel weer moeilijk hoor want soms merk je ook wel weer dat als je erg moet bijsturen als je zoiets hebt van 'oe, dat gaat niet helemaal de goede kant op'.

**Interviewer:** Heb je dan ook voor je hoe teamleiders, bijvoorbeeld die gesprekvoering dan, moeten uitvoeren? Of mogen ze dat dan helemaal zelf invullen?

**Respondent:** Wat wij niet weten, dat weten wij dan niet. Dat is het ook een beetje. Als zij ons niet vragen dan weten wij ook niet wat er allemaal aan gesprekken plaatvind. Ik denk wel over het algemeen, en dat zeg ik dan ook, ga altijd eerst het gesprek medewerker leidinggevende, niet gelijk HR erbij halen want dan geeft je mensen ook geen veilig gevoel. Dat is ook niet prettig. Maar als het gaandeweg niet loopt en je komt op een punt van 'ja, ik weet niet of wij verder met elkaar moeten', dan komen wij wel daarbij en kijken wij hoe wij daarin kunnen. Dat is dan vooral een rol van, wel beide kanten horen om te kunnen ondersteunen. Dan zit je daar niet alleen voor de leidinggevende, dan zit je daar ook voor de medewerker. Dus ja. Dat is dan het bemiddelingsrol die je dan pakt.

**Interviewer:** Heb je ook nog een idee hoe een teamleider zich zou moeten gedragen ten opzichte van medewerkers of collega's? Wat voor gedragingen laat zo iemand zien?

**Respondent:** Hoe die zich zou moeten gedragen.

**Interviewer:** Ja.

**Respondent:** Een voorbeeldrol. Dus ik denk dat dat wel heel belangrijk is. Als je bijvoorbeeld kijkt naar >TEAMLEIDER< op de poli en hoe zij >TEAMLEIDER< met de patiënten omgaat, dingen op een hele goede manier weet af te handelen als een patiënt ontevreden is. Nou dat is wel wat >TEAMLEIDER< uitdraagt en wat anderen zien. Dat vind ik heel belangrijk, dat wat je verwacht van je mensen dat je dat ook zelf uitstraalt. Dus ik denk dat dat het voornaamste is.

**Interviewer:** Dus als ik het goed begrijp is het zowel voor hoe je her moet doen als wanneer je het moet doen, dat eigenlijk zelf door de teamleider in te vullen als het uiteindelijke resultaat maar daar is?

**Respondent:** Of de hoe en wanneer zelf in te vullen is bedoel je daarmee. Dat wij daar geen richtlijn voor geven?

**Interviewer:** Juist. Teamleiders hebben wel een functiebeschrijving, maar hoe zij invulling geven aan de functiebeschrijving, is dat volledig vrij?

**Respondent:** Ik denk niet dat dat volledig vrij is. Want uiteindelijk zijn er wel een paar taken in de functieomschrijving die iedereen doet en waar je ook niet van kunt zeggen 'daar heb ik geen zin in om dat te doen'.

**Interviewer:** Heb je een voorbeeld van die taken?

**Respondent:** De jaargesprekken bijvoorbeeld.

**Interviewer:** Niet aan te ontkomen?

**Respondent:** Nee. Als teamleider heb je die verantwoordelijkheid en daar kun je dan niet van zeggen 'daar heb ik geen zin in'.

**Interviewer:** De functieomschrijving is dus wel echt de harde ondergrond van 'dit moet er gebeuren?'.

**Respondent:** Dat vind ik ook wel weer een lastige omdat wij daar dingen in gezet hebben die niet iedereen doet volgens mij, als ik dat goed voor ogen heb. Dus dat is dan wel van, dat zeg ik dan, afdelingsafhankelijk. Dus daar staan dan ook weer dingen in die niet iedereen doet.

**Interviewer:** Het is dus als teamleider belangrijk om je afdeling door en door te kennen?

**Respondent:** Ja. Dan is het wel wat jij zegt. Dat je dan samen met de bedrijfsleider kijkt 'wat is nodig, hoe doen we de taakverdeling, wat pak jij op?'. Sommige dingen zijn heel obvious dat doet een bedrijfsleider, dat doet een teamleider, er is dan geen ontkomen aan. Andere dingen zijn veel meer in afstemming van 'invulling op een afdeling'.

**Interviewer:** Dus het is aan de teamleider om die actieve rol te pakken en die verduidelijking te maken?

**Respondent:** Ja. In samenwerking. Dat doen ze samen. Ja, ik heb ook de functiebeschrijving niet voor ogen. Volgens mij staan er ook dingen in die niet iedereen doet.

**Interviewer:** Zowel generieke dingen staan erin als iets context-specifiekere dingen?

**Respondent:** Ja. Ik denk het wel. Misschien kun jij dat onderzoeken. Ik heb het niet op het vizier. Maar zo zie ik wel. Dat het op afdelingen dus zo verschillend wordt ingezet. Ik kan me dus ook zo voorstellen dat het dus inderdaad verschillend is en dus niet dat wat in de functieomschrijving staat iedereen doet. Maar dat is misschien ook wel grappig om in je onderzoek mee te nemen?

**Interviewer:** En misschien als laatste vraag, wat is jouw visie op de teamleider?

**Respondent:** Mijn visie op de teamleider?

**Interviewer:** Hoe zie jij de teamleider voor je. Moeten we van de teamleider af, of moet er veel meer door een teamleider gedaan worden, of juist meer naar seniors geschoven worden?

**Respondent:** Ja. Dat is steeds een leuke discussie die we ook hier hebben. Als de seniors OKE wordt afgeschaft, nee, als je een teamleider hebt, heb je geen senior OKE meer nodig. En als je een HBO verpleegkundige hebt, heb je geen teamleider meer nodig. Dan is het maar net aan wie ga je bepaalde taken en verantwoordelijkheden toebedelen? In het HBO-profiel zitten ook taken en verantwoordelijkheden die ook bij een teamleider, en ook andersom. En dan ga je ook weer die afstemming op afdelingsniveau. Hoe ga je dat dan invulling geven? Hebben we dan inderdaad geen teamleider meer nodig? Ik denk wel, dat zie ik ook heel duidelijk bij >AFDELING<. Kijk, je kunt zeggen ik doe daar helemaal geen senior OKE meer, of geen teamleider, dan mis je toch even die tussenlaag omdat >BEDRIJFSLEIDER< toch een verantwoordelijkheid heeft voor meerdere poli's, dan zou >BEDRIJFSLEIDER< zich weer heel operationeel moeten begeven op die poli's.

**Interviewer:** Dat is misschien niet iets voor een bedrijfsleider?

**Respondent:** Dat is niet iets voor een bedrijfsleider en ik weet ook niet of je dat wil als organisatie. Daar is >BEDRIJFSLEIDER< net te duur voor om zich met dat soort dingetjes bezig te houden. Ik denk dat je altijd wel een vorm houd van of teamleider of senior OKE. En dan is het maar net afhankelijk van 'wat heb je zitten'. Heb je al een senior OKE, wat is de grootte van de poli? Want misschien heb je dan inderdaad helemaal geen teamleider nodig en dan werkt het prima zo.

**Interviewer:** Dat is ook waar jullie nu mee zitten toch?

**Respondent:** Dat is ook lastig hoor. Wat is nou wijsheid. Ik heb toen ook gezegd, want toen wilde ze die senior OKE's afschaffen. Daar was ik op tegen omdat ik weet dat het op een aantal poli's gewoon heel goed werkt omdat daar geen teamleider is...

**Interviewer:** Ook niet nodig is?

**Respondent:** Ook niet nodig is. Dat heb ik ook gezegd, dan komt er in plaats van een senior OKE een teamleider EVR in schaal 55, dat wil ook niet. En er is geen tussenstap dus dan is zo'n senior OKE prima. Dat is dus ook net maar hoe je er tegen aan kijkt. Want op een grote >AFDELING< denk ik ook dat een teamleider onmisbaar is voor een teamleider want een bedrijfsleider, want ja, >BEDRIJFSLEIDER< heeft net een EVR erbij. Die kan dat niet allemaal zelf managen.

**Interviewer:** Dan raak je het overzicht kwijt?

**Respondent:** Dan is het te groot. Het aantal gesprekken dat je dan al moet gaan voeren is dan al te veel. En dat moet je dan op gaan delen en dat geldt ook voor de grote verantwoordelijkheden.

**Interviewer:** Heb jij een ideale oplossing in je hoofd?

**Respondent:** Ideale oplossing. Nee. Vind ik een hele lastige.

**Interviewer:** Dus als jij het nu voor het zeggen had zou je het lastig vinden één, twee, drie met een oplossing te komen.

**Respondent:** Ja, ook. Sommige dingen lopen goed zoals ze lopen. Als je dan nu weer een hele hoop over hoop gaat halen, wat levert je dat dan uiteindelijk op? Daar zat ik ook aan te denken. Dat maakt het ook lastig. Dat ik denk van. Dat vind dus moeilijk. We hebben toen geopperd 'doe functiedifferentiatie'. Dat was nog steeds waar ik van denk 'dat had moeten gebeuren voor de poli's'. Daar stond >BEDRIJFSLEIDER< toen ook achter want die vond die stap naar 55 ook erg groot en niet nodig. Maar dat hebben we wel allemaal doorgevoerd. Dus om nu te zeggen 'we stappen daar weer vanaf en gaan wel functiedifferentiatie toepassen', dan ga je die mensen wel weer demotiveren en terugbrengen. Dan denk ik wel van 'ja, we hebben wel een weg gekozen. We zijn ergens ingestapt.' Dat vind ik wel lastig. Dat is met senior OKE ook zo. Ik merk wel, voordat we iets doen, laten we dan wel heel goed...

**Interviewer:** Scenario's gaan plannen?

**Respondent:** Ja. Scenario's. Precies.

**Interviewer:** Je bent een beetje in een glazen bol aan het kijken als het ware?

**Respondent:** Ja. Dan denk ik 'als je nu weer gaat besluiten wat anders, dan het was'.

*Einde transcript*

# Transcript Interview FLM 1 and FLM2

**Duur interview: 40 minuten en 15 seconden**

**Functie: Teamleider**

**Afdeling(en) onder supervisie: Healthcare**

**Gender: vrouw, vrouw**

**Werkervaring in functie: 5,7 jaar / 1,8 jaar**

**Interviewer:** Laten we beginnen met de vraag wat jullie dagelijkse werkzaamheden binnen het ziekenhuis zijn.

**Respondent A:** Zal ik beginnen? Ik ben leidinggevende van de >AFDELING< en de >AFDELING< en daarnaast ben ik ook werkzaam als verpleegkundige op de afdeling. Dit betekent dat ik de rooster regel, het verzuim regel, de verzuimbegeleiding, opleiden van mensen, jaargesprekken voer, ik ben interne auditter. Ik zit nu na te denken wat ik eigenlijk allemaal doe. Ik ben interne auditter in het ziekenhuis. Dit betekent dat ik ook nog interne audits door het ziekenhuis heen houd. Daarnaast een stukje kwaliteit expliciet als aandachtsgebied heb. Daarnaast hebben we een leerwerkplaats op de afdeling.

**Interviewer:** Deze valt dan ook onder leiding van u?

**Respondent A:** Ja, dat klopt.

**Respondent B:** Ik ben leidinggevende over de poli-functie en de katkamer, de interventieruimte, daarnaast ook verpleegkundige die meedraait op de katkamer omdat ik de interventiemodule gedaan heb. Indien nodig spring ik ook nog bij voor de >AFDELING< want mij achtergrond is IC CCU verpleegkundige. Als teamleiders heb ik voor de poli en de functie de roostertaken, het goedkeuren van de vakanties, de verzuimgesprekken, de ziekmeldingen, scholing accorderen, binnen het LMS dingetjes doen, het fileteren van declaraties.

*Respondent A humt.*

**Respondent B:** Ik ben naar HR-dingetjes aan het zoeken. Jaargesprekken. Gewoon persoonlijke gesprekken met mensen, noem maar op. Voor de interventiekamer, naast het meewerken op de katkamer de planning van de patiënten en de informatiestroom naar patiënten, bestellingen binnen een inkoopprogramma hier in huis, contacten met de disciplines die van toepassing zijn op de katkamer, dat zijn de firma’s voor de devices en dat soort dingetjes. Daarnaast ben ik betrokken bij ziekenhuisbrede projecten en zat ik bij het meedenken van het digitale dossier, het verpleegkundig digitale dossier. In het verleden heb ik ook in de verpleegkundige staf gezeten. Daarnaast zit ik ook veel contacten met medische techniek en >AFDELING< rondom de nodige applicaties die gebruikt worden.

**Interviewer: Z**oals ik het nu hoor zit er best een behoorlijk stuk personeels-management in jullie functie. Hoe moet ik mij dit voorstellen? Ik kan het niet mooier verwoorden als wat ik voor mij zie: ik zie een zorgspecialist, maar ook een manager voor mij.

**Respondent A:** Je krijgt twee in een.

**Respondent B:** Je hebt beide petten op.

**Interviewer:** Hoe gaat dat in de praktijk?

**Respondent B:** Soms letterlijk in één. Of je nou op de katkamer staat, dan is het iets lastiger om sommige dingen tegelijkertijd te doen omdat je nu eenmaal in zo'n afgesloten ruimte bezig bent. Maar de dingen gaan wel gewoon door. Ook als ik in de zorg sta. Je hebt die pet van leidinggevende nooit af. Tuurlijk is mijn focus dan de patiëntenzorg, maar sommige dingen die kan of mag ik alleen als leidinggevende doen en mijn collega-verpleegkundigen niet. Dan schuif je wat met taken.

**Interviewer:** Dus dan komen ze uiteindelijk, ondanks dat u volledig in zorgfunctie bent...

**Respondent A:** Ja, dat is prima. Ik blijf hetzelfde. Of het nu vijf voor vier is of vijf over vier, om vier uur eindigt de dagdienst, dan heb ik liever dat ze het me eerder zeggen. Soms kan het niet als ik te druk ben met bepaalde patiënten, kom daar dan later bij me op terug. Ik denk dat het helpt, dan praat ik even voor ons allebei, dat we daar duidelijk in zijn in verwachtingen, ook naar medewerkers toe, wanneer kun je waarmee waar terecht. Datzelfde geldt ook nu ik met jou in gesprek ben. Dan zien mensen dat de deur dicht is. Normaal is de deur open en kunnen mensen te pas en te onpas binnenlopen. Als de deur dicht is, dan is er iets. En als er nood aan de man is, dan moet je nog steeds binnen komen, maar dat is wel een drempeltje om duidelijk te maken 'ik zit nu in gesprek'. Zo hebben we er ook voor gekozen om als we in zorg zijn, dat we dan in het wit zijn en als we niet in zorg zijn om dan niet in het wit te zijn. Dat maakt dan voor medewerkers duidelijk van 'wat is je rol vandaag en kan ik mij bestoken met je vragen, of moet ik die even achterwege laten?'. Dat helpt in duidelijkheid scheppen.

**Interviewer:** Als we dan kijken naar een soort verdeling, zie ik dan een fifty fifty verdeling voor me: vijftig procent zorg en 50 procent management? Of ligt die verdeling heel anders?

**Respondent B:** Dat is heel wisselend.

**Respondent A:** Heel wisselend. Afhankelijk van het rooster. Dus de planning. Omdat je toch, de zorg gaat voor te allen  tijde. Die verpleegkundige hoort aan het bed te staan en als je het rooster niet rond krijgt, ga je toch bijspringen als er geen uitzendkracht aanwezig is of er is een zieke ad hoc. Dat heeft dan wel weer een invloed op je agenda. Dan blijven er dingen liggen omdat de patiënt even voor gaat. Vanuit het functieprofiel heb je een deel van je uren die je meedraait in de zorg. Maar, het is heel wisselend. Het ene moment. Bijvoorbeeld deze week, sta ik drie dagen beneden op de katkamer. En deze week stond jij ineens op de >AFDELING< omdat we een zieke hadden.

**Respondent A:** Volgens de functieomschrijving is het 25 procent meewerkend. Dat meewerkend kun je zo breed en zo smal mogelijk zien als dat je zelf wil. Is dat werkelijk in het wit aan het bed om iemand te wassen bij wijze van spreken. Of valt meewerken ook onder protocollen in orde maken, zorgen dat de roosters gemaakt worden. Dat zijn maar net de dingen die je onder 'meewerken' verstaat. Het is een breeds begrip.

**Respondent B:** De katkamer is momenteel wat krap. Daar zit bijna een vacatureruimte van bijna zes FTE totaal. Dus daar zit wat krapte. Dan nijg je sneller naar meedraaien. Maar ik denk dat doordat we vijf jaar gewerkt hebben om duidelijkheid te creëren voor mensen vanuit de goede zin van het woord. Je heb invloed op je werk, dat maakt het werk leuker. En ook door duidelijk te zijn. Zoals gisteren ik trek mijn pak aan, ik kom erbij, maar ik wel graag mijn afspraak van half twaalf door laten gaan. Kunnen we dat regelen? Dat doe ik 's morgens bij de dagstart. Kan het niet, dan is het jammer. Maar mensen weten ook 'het zou heel fijn zijn voor haar als die afspraak door zou kunnen gaan'. En eigenlijk kan het altijd. Dat is dus het mooie wat je met elkaar kunt regelen. Maar dat gaat niet vanzelf. Daar moet je wel in investeren om dat voor elkaar te krijgen.

**Interviewer:** Ik heb eens opgezocht wat voor een taken allemaal bij een teamleider neergelegd kunnen worden. Als je het lijstje doorgaat, zie je dan nog bepaalde punten waarvan je denkt 'die springen er echt uit, hier ben ik een groot stuk van mijn tijd mee bezig'.

**Respondent A:** Eigenlijk met heel veel dingen. Het verbeteren van werklevens, gezondheid en veiligheid, personeel raadplegen. Ik zeg wel eens gekscherend als ik thuis kom 's avond en ze vragen 'heb je een drukke dag gehad?', dan zeg ik 'ik heb alleen maar koffie gedronken'. Maar voor mij zijn dat functionele koffiegesprekken. Natuurlijk vind ik het ook lekker dat ik contact heb met de mensen, verbinding, verbinding vind ik een naar woord. Dat als er geen druk op de ketel staat dat ik ook dat gesprek met mensen heb. Zodat als het wel een keer kort door de bocht moet. Dat mensen dan gewoon weten, dan hoef ik daar niet zoveel tijd aan te besteden op momenten dat ik die eigenlijk niet heb, om dingen geregeld te krijgen. Effectief teamwerk, coördineren, daar ben ik veel mee bezig. Dit zijn allemaal, dit gebeurt allemaal door elkaar heen. En sommige dingen zijn nu eenmaal nodig top-down omdat die informatiestroom zo gaat. Maar juist ook door die functionele kopjes koffie zegmaar, probeer ik ook die bottom-up gevoed te houden. Beslissen over salarisschalen, dat liggen allemaal wel vrij vast hier. Waardering geven zit hem voor mij voornamelijk in het geven van aandacht. Mensen zien, gezien worden, en soms iets voor ze kunnen regelen, en ze daarentegen ook iets kunnen vragen. Om daar een balans in te houden. Absentiemanagement, ja, de hele dag door. De ene keer meer dan de andere. Tuchtprocedures heb ik niet zoveel mee van doen. Klachten wel.

**Interviewer:** Komen die dan ook bij jullie terecht?

**Respondent A:** In principe wel.

**Respondent B:** Dat hebben wij bij ons wel zo geregeld. In principe heeft de bedrijfsleider daar ook een rol in. Soms is het maar net, wat voor een klacht is het. Wie pakt het op?

**Respondent A:** Ligt ook aan het soort klacht dat het is.

**Respondent B:** Ja, wat voor soort klacht het is. Maar meestal komt die bij ons te liggen. Ontwikkelingsplannen, meestal bespreek ik die met de medewerker en voor akkoord stuur ik hem naar de bedrijfsleider. Maar dat is dan eerder van 'zie ik iets over het hoofd, heb ik overal aan gedacht?'. Even een tweede blik erop werpen. Dat zouden we eventueel ook met z'n tweeën kunnen doen. Jaargesprekken houd ik ook met iedereen. Mentoren ook. Dat zijn dan meer persoonlijke situaties. Informele training valt een beetje samen het coachen en mentoren. Formele training geef ik niet. Dat is ook meer omdat ik me afvraag of het van toepassing is. Ik probeer dat namelijk meer bij de medewerker neer te leggen omdat ze vaak op inhoudsniveau de uitdaging kunnen vinden. Plannen van training en ontwikkelingsbehoeften zeker. Personeelsbestanden ja. Werving en selectie ja. Eigenlijk wel het hele rijtje.

**Interviewer:** Er is dus eigenlijk best veel herkenning? Ook bij jou?

**Respondent B:** Ja, daar sluit ik mij bij aan.

**Interviewer:** Geen bepaalde punten waarvan je denkt 'die springen er uit'?

**Respondent B:** Nee, ik dat dat we binnen de EVR >AFDELING< op die manier, ongeacht of je nou op een >AFDELING< of op de >AFDELING< of op de polifunctie werkt, op dezelfde manier behandeld wordt. Waarbij we toch zoveel mogelijk proberen de mensen zelfsturend verantwoording te geven. Voor de polifunctie hebben we dan bijvoorbeeld aandachtsvelden ingericht, dus de taken onderverdeeld per onderdeel wat daar speelt. Dat ze daar dus ook een stukje verantwoording in hebben en ook gewoon meer betrokkenheid laten zien. De manier van oppakken is uniform. Dat houdt ook in dat op het moment dat ik vrij ben dat >RESPONDENT A< ja op dezelfde manier de poli, de functie, of de katkamer benader. Als er specifieke vragen zijn of brandjes die op dezelfde dag geblust moeten worden, dan los je dat op. En anders zeg je 'morgen is >RESPONDENT A< er weer of morgen is >RESPONDENT B< er weer, dan kun je het daarmee bespreken.'

**Respondent A:** Om te voorkomen ook dat je tegen elkaar uitgespeeld wordt.

**Interviewer:** En we dan eens kijken naar de bevoegdheid er dan komt kijken bij zo'n HR taak, hebben jullie dan een idee tot hoe ver jullie bevoegdheid rijkt? Bijvoorbeeld, tot dit kunnen wij doen en voor alles daarbuiten gaan we naar >BEDRIJFSLEIDER< of HR.

**Respondent B:** Ik denk dat dat wel duidelijk is. Dat staat niet zwart-wit op papier. Voor mezelf loop ik niet op grijze gebieden waarvan ik onzeker wordt of 'dat weet ik nu even niet'. En mocht dat toch gebeuren, of in het verleden gebeurt zijn, dan is onze bedrijfsleider zo laagdrempelig bereikbaar dat een overleg te allen tijde mogelijk is.

**Interviewer:** Kun je proberen aan te geven waar de grens ligt? Wat mag door jou afgehandeld worden en wat moet door de bedrijfsleider afgehandeld worden?

**Respondent A:** Sommige dingen zitten in een systeem. Een bevoegdheid in systemen. Dan stuur ik een mailtje, of bel ik even op van 'wil je dit even doen?'. Wat dat kan ik dan niet in het systeem. Of het nu een mutatie is of iets qua uren, dan zit het meer daar in...

*Respondent B knikt.*

**Respondent A:** ... Verder denk ik dat we. We hebben ook een tijdje, door ziekte, zonder bedrijfsleider, of nouja niet helemaal zonder bedrijfsleider gewerkt. Maar waar kom je dan op uit en waar loop je dan tegen aan als die persoon er niet is waar je het normaal mee geregeld hebt, dan is het vaak systemen en informatiestromen. Wordt je goed op de hoogte gehouden van dingen die normaal via je bedrijfsleider hoort? We horen namelijk heel veel van onze bedrijfsleider. Daar mis je dan soms iets in. Dan moet je heel actief op zoek van 'hoor ik alles dat van belang is om te horen?' Krijg ik alle mailtjes door die van belang zijn om te horen. Kijk, nu weet dat alles wat van toepassing is, dat krijg ik doorgestuurd. Of verteld of wat dan ook. Als diegene er niet is, dan weet je niet altijd of dat gebeurt.

**Respondent B:** Kijk, je hebt gewoon bepaalde overlegvormen hier in huis. Daar zit de bedrijfsleider en eventueel de EVR voorzitters, maar als teamleider ben je daar niet bij aanwezig. Dat is een bewuste keuze hier in huis. Met als gevolg dat je als teamleider de informatie van jouw bedrijfsleider moet ontvangen. Dat is hier binnen de >AFDELING< goed geregeld. We hebben honderd procent vertrouwen dat alles wat nodig is via de mail bij ons binnen komt of onderling verteld wordt. Maar goed, ik weet natuurlijk niet of dat op alle plekken zo is.

*Teamleider 1 knikt.*

**Interviewer:** Houdt dit dan ook in dat jullie over ieder HR-onderwerp een besluit mogen nemen of is het ook zo dat je voor bepaalde onderwerpen eerst voor afstemming gaat zoeken, voordat je een akkoord geeft?

**Respondent A:** We zoeken veel afstemming. Maar dat ligt eerder aan de manier waarop we samenwerken denk ik. Dan in formeel 'wie mag wat beslissen?'. Kijk, mensen aannemen, we kunnen het niet eens voor elkaar krijgen in het systeem. Maar, het zijn wel onze medewerkers. We moeten wel meedenken. Maar dat is allebei even zwaar invloed hebben.

**Respondent B:** Formeel, de sancties, formele handtekening, dat is gebonden aan de functie. Dat is bij ons gebonden aan >BEDRIJFSLEIDER<. Maar >BEDRIJFSLEIDER< vindt onze input wel belangrijk. Zoals bij sollicitatiegesprekken, dan zitten we er altijd bij afhankelijk van de werklocatie. Of als er iets niet lekker gaat met een persoon, dan wordt er altijd wel overlegd van 'goh, wat vinden jullie er van?'.

**Respondent A:** Vaak samen. Dat je met z'n tweeën dat soort gesprekken houdt. Dan maakt de ene de notulen en neemt de ander de leiding in het gesprek.

**Interviewer:** Is dat het zelfde voor het doen van financiële aankopen?

**Respondent A:** Grote bedragen gaan via >BEDRIJFSLEIDER<. Kleine bedragen...

**Respondent B:** Voor de Katkamer heb ik via >BEDRIJFSLEIDER< een account gekregen dus ik kan voor materialen voor de Katkamer kan ik via inkoop gewoon bestellen.

**Respondent A:** Meer de hoge bedragen gaan via >BEDRIJFSLEIDER<. En hoe hoog het bedrag is, dat zou ik niet weten. Als zou het 10.000 euro zijn, als het daaronder ligt zou ik nog steeds zeggen 'goh, >BEDRIJFSLEIDER< wat vind je er van?'. Als we dat allemaal vier keer per jaar doen. Ja, je runt de tent samen.

**Interviewer:** Dat komt dan toch weer terug op het overleg en de manier van samenwerken?

**Respondent B:** Ja, klopt. We hebben nieuwe echoapparatuur aangeschaft. Dan hebben het over bedragen van tienduizenden euro’s tot een paar ton. Dan heb je gewoon haar handtekening nodig. Maar je mag wel het project draaien. >BEDRIJFSLEIDER< kijkt op de achtergrond mee en is tevreden als je >BEDRIJFSLEIDER< meeneemt in de cc omdat ze uiteindelijk de handtekening moet zetten. Dat geldt natuurlijk ook voor hier.

**Respondent A:** Ja, klopt. Wat is recent... Recent, dat klinkt alsof het vorige week is. Maar recent, hebben we een verslavingsprobleem gehad op de afdeling bij een medewerker. Daar miste ik echt beleid in. Dat maakte dat wij dat samen hebben bedacht.

**Respondent B:** Ziekenhuisbreed beleid.

**Respondent A:** Maar we miste  ziekenhuisbreed beleid waardoor we dit zelf hebben moeten uitzoeken naar eer en geweten. Dat zijn wel lastige problemen. We hebben het hier over een medewerker die verslaafd is aan alcohol in ieder geval. Zonder beleid is het heel lastig om te bepalen wie wat mag qua sancties of gesprekken überhaupt. Wat mag je, wat kun je? Kijk, dat heeft ons wel doen groeien met elkaar. Maar dat maakt wel het stukje HR, bedrijfsleiding, teamleiding. Wat doe je als je een zakje wiet in een kleedkamer vind? Ik lig niet wakker van dat zakje wiet. Maar, er is geen beleid. Wat gaan we hiermee doen?

**Interviewer:** Precies, de kaders zijn belangrijk wat dat aangaat?

**Respondent A:** Ja, de kaders zijn heel er belangrijk. Het moet namelijk niet uitmaken of dat zakje wiet op mijn afdeling ligt, of op een andere afdeling ligt. Dat soort problematiek. Ik weet dat er nu gewerkt wordt om daar beleid op te maken. Maar dat hebben we heel erg gemist.

**Interviewer:** Als we kijken naar jullie HR-taken, hebben jullie dan eigenlijk nog een bepaalde standaard in jullie hoofd van 'oké, deze taak heb ik nu afgerond of dit is het resultaat, dan is het naar mijn idee naar behoren uitgevoerd'. Hebben jullie een soort graadmeter?

**Respondent B:** Ik denk dat die graadmeter heel persoonlijk is. Ik denk niet dat er een ziekenhuisbrede standaard is. Neem als voorbeeld de jaargesprekken. Daar is een standaard formulier voor. Maar het gesprek op inhoud en de uitkomst, die vul je samen in met de medewerker.

**Respondent A:** Die vul je samen in.

**Respondent B:** Zelfs het formulier kun je nog heel creatief invullen. Niemand van HR zou zeggen 'goh dit is goed, goh dit is niet goed'. Althans dat heb ik nog nooit gehoord.

**Respondent A:** Wat eigenlijk alleen teruggegeven wordt is het percentage gehouden jaargesprekken. Los van het feit of het een goed gesprek was.

**Interviewer:** Hoeveel zegt jullie zo'n percentage?

**Respondent A:** Wij voeren de gesprekken. Het zegt meer over de aantallen dan over de kwaliteit van de gesprekken.

**Respondent B:** Die terugkoppeling. Dat zijn dan weer andere systemen...

*Teamleider 1 humt.*

**Respondent B:** ... waar je als teamleider mee moet dealen. De terugkoppeling is niet real-time. Als wij bijvoorbeeld in onze lijst kijken en we zien 80 procent. Dan kan het zomaar zijn dat een HR afdeling aangeeft dat we op 30 procent zitten. Dat is wel frustrerend.

**Interviewer:** Dat kan ik mij voorstellen. Hebben jullie het idee dat er nog bepaalde kennis nodig is om jullie werk uit te voeren? Dan doel ik op de personeelstaken. Zijn er bepaalde dingen die standaard zou moeten weten?

**Respondent B:** Als teamleider hiervoor was je senior zorgeenheid. Toen kwam de functie van teamleider in huis. Toen hebben we intern een teamleidercursus voor gedaan. Wat ik zie hier in huis is dat het woord 'teamleider' is standaard het functieprofiel teamleider is standaard, er komt één profiel uit. Alleen de invulling is zo divers als wat. Als je ook kijkt van 'goh hoe gaat HR daar mee om?'. Toen zeiden ze destijds van 'goh, we hebben hele goede teamleiders in huis en vanuit HR focussen we ons daarom en gaan we kijken wat we de mensen kunnen aanbieden. Dat je in een soort kweekvijver. Er zitten een hele hoop in die kweekvijver. Maar de vervolgstappen vanuit HR die mis je. Die deuren blijven dicht. Dat je echt het gevoel hebt van 'goh, ik ben nu zo erg gegroeid ik barst die vijver uit'. Dan barst je letterlijk die vijver uit en ga je extern wat zoeken want hier in huis kom je niet veel verder. Dat soort dingen zou HR, dat zou een stap zijn om aandacht aan te besteden in huis. Hoe zorg je er voor dat...

**Respondent A:** Dat je genoeg doorgroeimogelijkheden hebt.

**Respondent B:** ... je A zegt en B doet. We hebben een kweekvijver, we hebben potentie, alleen B moet nog komen.

**Respondent A:** Ik denk dat, niet alle EVRs hebben teamleiders. Sommige werken zonder teamleider. Ik denk dat je dan ook verschil krijgt in bedrijfsleiderfuncties krijgt, ik denk dat je dus ook verschil krijgt in teamleidersfunctieinvulling. Iedere bedrijfsleider mag is volledig vrij om de functie teamleider in te vullen. En dan ook de vraag, wat heb je dan nodig als teamleider om HR taken te doen. Het ligt er maar net aan wat je van je bedrijfsleider mag doen. Ik ben heel blij dat ik bij deze bedrijfsleider terecht ben gekomen. We mogen heel veel doen. We mogen ons heel erg ontwikkelen en de bedrijfsleider probeert aan ons te delegeren waarvan >BEDRIJFSLEIDER< denkt dat wij daar aan toe zijn. Soms ook om het te proberen. En dan gaan we op ons snuit. Ja, dat gebeurt. Maar je leert het door het te doen. Dus ja wat heb je nodig. Je hebt kennis van de wetten nodig, je hebt kennis van de CAO nodig. Dit kun je zelf opzoeken, maar soms helpt het om even gewezen te worden op 'hoe werkt dat dan?', financiële kaders kennen, maar ook vooral gespreksvoering. Hoe doe je dat, hoe werkt dat, hoe voelt dat, als jij op een bepaalde manier je gesprek voert.

**Interviewer:** Dan ben ik nu wel benieuwd, als we in de schoenen gaan staan van >BEDRIJFSLEIDER<, wat verwacht >BEDRIJFSLEIDER< van jullie in termen van die HR taken? Wat valt wel binnen jullie takenpakket en wat valt niet binnen jullie takenpakket?

**Respondent A:** Ik denk dat >BEDRIJFSLEIDER< een grote rol heeft in de werving en selectie. Uiteindelijke besluitvorming. Tucht- en klachtprocedures in eerste instantie, voor het grootste gedeelte onder haar verantwoording valt. Voor de rest denk ik er heel veel gedelegeerd is. Uiteindelijk is >BEDRIJFSLEIDER< verantwoordelijk voor alles. Maar als ik vanuit haar kijk, dat >BEDRIJFSLEIDER< die dingen als grootste verantwoordelijkheid noemt. Natuurlijk, wij zijn haar signaalfunctie van 'wat gebeurt er met medewerkers?'. Maar dat is een soort doorgeefsysteem. De belangrijkste dingen, die geven wij door.

**Respondent B:** De bedrijfsleider houdt de formatie bij, houdt de financiële situatie in de gaten, heeft contact met de EVR-voorzitter, ik denk meer op dat niveau. Maar input voor die onderwerpen leveren wij weer aan. Mochten er dingen zijn, dan wordt het weer naar ons teruggekoppeld. Maarja, zoals >TEAMLEIDER 1< net al zei, ze is vrij transparant en open richting ons. Dat geeft ons de gelegenheid om ons ding te doen waarbij het voor ons goed voelt. Destijds heeft ze ook ons zodanig geselecteerd. Wij zijn plus en min, Yin en Yang. Dus dat vult elkaar goed aan. En dat heeft ze goed gezien.

**Interviewer:** Hebben jullie een idee dat >BEDRIJFSLEIDER< bepaalde standaarden in haar hoofd heeft hoe de HR-taken uitgevoerd moeten worden?

**Respondent B:** De kreet die >BEDRIJFSLEIDER< als tijd heeft en waar >BEDRIJFSLEIDER< 's nachts mee wakker wordt is: doe wat je zegt en zegt wat je doet. Wat ik zelf, qua mijn persoonlijkheid, erg belangrijk vind is vertrouwen en beloofd is beloofd. Als je iets beloofd dat moet je dat ook waarmaken. En oprecht zijn.

**Respondent A:** Met name dat wij een signaalfunctie hebben. Zodra dingen af dreigen te wijken van wat we vooraf bedacht hadden of vooraf afgesproken hadden, dat we >BEDRIJFSLEIDER< daarbij helpen met die signaalfunctie. Want daar komt eerder bij ons binnen dan bij >BEDRIJFSLEIDER<binnen. Dat is niet, dat is gewoon omdat het een grote EVR is. En transparant naar elkaar zijn.

**Interviewer:** Hebben jullie een idee dat >BEDRIJFSLEIDER< ook nog bepaalde taken op een bepaald moment uitgevoerd willen hebben?

**Respondent A:** Zoals we het afspreken. We spreken het vrij SMART af. Dus volgens afspraak, tenzij, geef dan aan dat het niet gaat lukken. Er is geen vaagheid over 'goh nu zou ik iets moeten doen, maar geen idee wanneer ik dat zou moeten doen.'

**Respondent B:** Een heleboel dingen liggen ook vast in een bepaalde cyclus. Je weet dus ook bijvoorbeeld dat je teamvergaderingen die zijn een bepaalde tijd van te voren vast gelegd, voor het hele jaar ingepland voor alle afdelingen. We bedenken ook de agenda voor ieder vergadering.

**Respondent A:** Jaargesprekken plannen we zelf. Wat >BEDRIJFSLEIDER< zegt is 'regel het'. Ieder jaar moet een medewerker een jaargesprek. Regel het.

**Interviewer:** Is er dan een soort draaiboek?

**Respondent A:** Ja, zoiets. Ik heb een bestandje met daarin wie een jaargesprek heeft gehad en wanneer.

**Respondent B:** Kijk, >BEDRIJFSLEIDER< maakt dan bijvoorbeeld de nieuwsbrief en dan weet je ook gewoon einde van de maand 'heb je input voor de nieuwsbrief, lever het aan' dus die is dan voor de hele EVR. Dan heeft >BEDRIJFSLEIDER< die overleggen waar >BEDRIJFSLEIDER< naartoe gaat. Die bereiden we met z'n drieën voor.

**Interviewer:** Dus als ik het goed begrijp valt of staat alle succes van deze EVR met communicatie?

**Respondent A:** Ik denk voor iedere EVR.

**Respondent B:** Voor ons geldt dat wel. Communicatie en transparantie.

**Interviewer:** Mijn laatste vraag: wat voor gedrag zou >BEDRIJFSLEIDER< van jullie verwachten op de werkvloer?

**Respondent B:** Daar heb ik maar één woord voor: voorbeeldfunctie.

**Respondent A: J**a, voorbeeldgedrag. Je ligt onder een vergrootglas. Ook als je meewerkt. Alles wat jij zegt, doet of laat, is aanleiding van de gemiddelde medewerker om dat ook niet te doen of meestal niet te doen, of wel te doen. Dus wees je bewust van je voorbeeldfunctie, want je ligt onder een vergrootglas. Daar hoef je geen last van te hebben, maar ben je daar wel van bewust. Als we afspreken dat we geen sieraden dragen, dan doe je dat ook niet.

**Respondent B:** Dan heb je geen horloge om. *Respondent twee laat polsen zien. Teamleider 2 draagt geen horloge om de polsen.*

**Respondent A:** In de zorg dan. Uniform aan, is sieraden af. Je heb je registratie op orde en de boel kan uit elkaar klappen. Maar in principe doe je helemaal volgens het boekje zoals je het doet. Zoals we samen met elkaar hebben afgesproken. Want ja, wat verwacht je? Als ik het niet doe, wat verwacht je dan van je medewerker?

**Respondent B:** Nu zijn wij van die typen die er 100 procent achter staan, dus we hebben er ook geen last van. Wees je ervan bewust. Dat werkt wel omdat je dit dan mag verwachten van medewerkers.

**Interviewer:** Ik heb al mijn vragen gesteld. Zijn er misschien nog dingen die jullie aan het interview willen toevoegen?

**Respondent A:** Probeer alsjeblieft de functie van teamleider te behouden. De stappen tussen verpleegkundigen en bedrijfsleider zijn best wel groot en die seniorverpleegkundigen die doen soms best wel rottige klussen maar dat doet >BEDRIJFSLEIDER<, maar soms ook hij, natuurlijk omdat ze het leuk vinden of omdat ze de uitdaging erin zien of omdat ze willen bepalen of zo'n leidinggevende rol echt iets voor mij is. Iets om daarmee te oefenen. Maar ze kregen een gratificatie van tachtig euro bruto per maand. Is dat het nou? Is zonde als je die doorstroom, dat carrièreperspectief niet meer hebt. Een hoop van die leidinggevenden in de kweekvijver. Natuurlijk, je hoeft geen plekken te creëren, maar je wil wel je betere mensen en je ambitieuze mensen behouden. Het gevolg is dat je mensen die achterover leunen die blijven, en de ambitieuze mensen die gaan weg. Dat is heel er sonde. Je blijft maar aan die kar trekken en die mensen gaan niet ineens intrinsiek gemotiveerd raken, die mensen die achteroverleunen.

**Interviewer:** Dus het zou zonde zijn om de rol van teamleider, als laag, weg te knippen?

**Respondent A:** Zelfs senior zou ik zonde vinden.

**Respondent B:** Je hoort nu toch al weer geluiden dat die terug zou moeten komen. We zijn nu bezig met HBO-V'ers. Die moet je ook uitdaging bieden. Dan moet je werk anders gaan indelen dan voor de MBO'ers op de >AFDELING< of waar dan ook. Dan laat je die HBO'er als senior optreden. Maak beleid.

**Respondent A:** En handel daarnaar.

**Respondent B:** Het blijft nu in het ongewisse. Het worden wandelganggesprekken. Maar er is geen daadkracht. Net als die teamleiderfunctie. Die is heel divers en wordt overal anders ingevuld van 'ik ben teamleider van twee personen' tot 'ik ben teamleider van twee >AFDELING<en'. Dat maakt niet, het komt op hetzelfde neer zeg men. Maar doe er wat meer mee, ga één keer per jaar met alle teamleiders, vanuit HR, een bijeenkomst organiseren of ga één-op-één in gesprek van 'waar sta je, wat heb je nodig, waar ga je naartoe?'.

**Respondent A:** Dat gebeurt nu middels het jaargesprek met je bedrijfsleider. Maar verder dan dat komt het niet. Dat is heel er sonde.

**Respondent B:** De insteek nu is 'ik moet gaan halen'. Het zou mooi zijn als HR ook komt brengen.

**Interviewer:** Duidelijk.

*Einde transcript.*

# Transcript Interview FLM 3

**Duur interview: 34 minuten en 34 seconden**

**Functie: Teamleider**

**Afdeling(en) onder supervisie: >AFDELING<**

**Gender: vrouw**

**Werkervaring in functie: 4**

**Interviewer:** Zou je over je dagelijkse werkzaamheden in het ziekenhuis kunnen vertellen?

**Respondent:** Ja, dat wisselt heel er per dag. Zoals je weet staat de teamleider ook af en toe in de zorg. Dus het zou kunnen dat ik in de zorg sta en richt op de patiënten. Maar goed, dan houd je alsnog je functie als teamleider dus ontkomt je er niet aan dat er dingen op de werkvloer operationeel of vanuit de organisatie, dat je telefoontjes krijgt dat je toch dingen moet regelen. Het is heel breed. Het is belangrijk dat het personeel zich prettig voelt op de afdeling dus dat is echt het stukje HR. Dat gaat tot het houden van gesprekken, het geven feedback, maar ook in gesprek gaan over functioneren of dingen die mij opvallen. Maar ook over samenwerking. Dat is heel breed. Ik doe ook functioneringsgesprekken met de verpleegkundigen hier. Het gaat ook over de kwaliteit van de zorg die ze leveren. Zijn ze geschoold? Dus in dat opzicht kijk ik ook naar personeel en ook naar kwaliteit op de afdeling. Ook dat is heel breed. Dan heb je het dus over scholing van personeel. Dus ook daar, daar stuur ik op. Maar dat houd ik ook in de gaten door je kan het zien in ons leermanagementsysteem. Maar ook hoe dat in de praktijk gebeurt zeg maar. Je hebt ook toetsers die toetsen in de praktijk, maar ook het organiseren van klinische lessen. Dat we hier in ieder geval werken met mensen die goed geschoold zijn en bijblijven met de kennis. Maar goed, dat doe ik ook wel veel samen met >BEDRIJFSLEIDER< hoor. We bespreken veel en ik bespreek veel met >BEDRIJFSLEIDER< van 'joh, wat voor scholingen gaan we doen dit jaar, en wat is de behoefte van de afdeling en van het personeel, waar zullen voor kiezen, wat voor budget hebben we?'. Dus dat, ik heb wel hele nauwe contacten met >BEDRIJFSLEIDER<. We spreken elkaar bijna elke dag. We starten de dag samen even op kantoor op door even te vertellen wat er allemaal gebeurt is. Dus ook al ben ik een dag vrij, dan weet ik wat er gisteren gebeurt is. Dus dat is heel prettig. Heel veel doen we dus ook samen. Het is niet zo dat ik hier alles op de werkvloer regel. >BEDRIJFSLEIDER< speelt daar ook een rol in. Dat overlapt elkaar dus een stukje. Maar over het algemeen ben ik operationeel wel op de werkvloer en >BEDRIJFSLEIDER< wat meer de organisatie in. Nog mee over mijn. Kwaliteit op de afdeling hadden we het over. Dan heb je het echt over protocollen en over procedures en over bijvoorbeeld Idoc, het systeem waar alle documenten in staan. Dus ik zorg, ik hoef dat niet allemaal inhoudelijk zelf te doen, maar dat doe ik wel deels samen met een collega. Ik kijk dus of al die documenten op orde zijn. Of de signalen komen van de werkvloer van 'goh, die arts dat loop niet, dat moeten we anders doen'. Dus kijken naar procedures of die anders kunnen. Dat delegeer ik ook aan verpleegkundigen. Dan hoef ik dat niet allemaal zelf op te lossen. Maar wel een soort helicopterview over de afdeling dat wat er allemaal loopt. We hebben dan verschillende werkgroepen. Dus aandachtsvelders. Dus sommige storten zich op het stukje leerlingen, sommigen storten zich op scholing en het toetsen van collega's. Daar krijg ik terugkoppeling van hoe dat loopt. Mocht ik dingen krijgen vanuit >BEDRIJFSLEIDER< of vanuit de organisatie dat er iets van ons verwacht wordt, dan geef ik dat weer terug naar de verpleegkundigen. Even kijken hoor, nog meer mijn. Bijvoorbeeld ziekenhuisbreed is nu bijvoorbeeld het EVD een hot item, het digitaal verpleegkundig dossier. Daar speel ik wel een grote rol in, want dat betekent een hele grote verandering hier voor de afdeling. Dus ik ben ook samen met >BEDRIJFSLEIDER< aan het kijken, hoe kunnen we dit implementeren? Nouja, heel veel communicatie richting de verpleegkundigen van 'jongens, het komt eraan'. Mensen een beetje warm houden en vooral collega's vertellen wat er in de organisatie gebeurt dat dat ook hier leeft zeg maar, op de afdeling. Ook dat is een hele belangrijke rol, die soms best wel lastig is hoor, want weet je, verpleegkundigen zijn heel druk hier en nouja, er is best veel informatie om te vertellen vanuit de organisatie, maar in hoeverre je ze niet overlaad met informatie maar ze wel geïnformeerd houd is wel een leuke uitdaging. Dat is wel belangrijk voor >BEDRIJFSLEIDER< en mij die communicatie. Samenwerking met de artsen is belangrijk. Dus ik heb ook contacten met de artsen, daar zit ik ook structureel mee van 'hoe gaat die samenwerking en waarin kunnen we verbeteren?'. Dat een beetje in het algemeen.

**Interviewer:** Duidelijk. Je zit hier nu in werkkleding. Komt het ook wel eens voor dat je in burger bent.

**Respondent:** Dat kan ik heel vaak doen. Maar het is mijn keuze omdat ik eigenlijk ook denk 'het is ook makkelijk', dus ik doe dat dan 's ochtends aan. Dat betekent niet dat ik vandaag aan het bed ben geweest. Dat ben ik namelijk helemaal niet. Ik had hier nu ook in gewone kleding kunnen zitten. Maar goed, dat is een beetje gemakzucht vanuit mijn kant. Dat ik denk 'dan hoef ik niet na te denken over wat ik aantrek' en opzicht, in het begin, toen was de functie nog niet heel duidelijk en toen was het nog wel zo dat collega's zoiets hadden van 'wil jij die patiënt even doen, wil jij even helpen tillen?'. Toen werd ik iedere keer weggetrokken en toen dacht ik 'misschien is het goed om af ten toe even gewone burgerkleding aan doen, zodat die rol duidelijk is'. Maar de rol is nu zo duidelijk dat het niet uitmaakt wat voor kleren ik aan heb om te laten zien wat voor een functie ik heb.

**Interviewer:** Hoe is die rol duidelijker geworden over tijd? Wat is daarvoor gedaan?

**Respondent:** Ook omdat we voorheen seniorrol hadden, en dat was niet een functie, maar dat was een rol, je zat er niet schaal hoger, dus daar kregen we wel wat tijd voor, maar goed dat was ook de praktijk dat moet uitwijzen of dat lukte en dat was samen met twee andere collega's. Dan had eentje het aandachtsveld kwaliteit, eentje het aandachtsveld opleidingen. Dus dat was dan echt een rol. En toen kwam er opeen de functie teamleider. En toen dacht iedereen ook van 'wat is nu het verschil?'. Ook ik moest even mijn plek hier vinden en ook >BEDRIJFSLEIDER< moest ook even kijken van 'wat ga ik nou doen, en wat laat ik los?'. Dat is denk ik ook gewoon een natuurlijk proces. Dat dat in het begin nog even duidelijk moest worden. Ook ik had in het begin van 'hoe ga ik die functie inkleden?'. Natuurlijk kun je een functieprofiel hebben. Dan nog zal je heel goed moeten kijken naar de behoeften van het team en wat past bij mij en wat wordt er verwacht van de bedrijfsleider en wat wordt er verwacht van de organisatie?

**Interviewer:** Uiteindelijk zijn jullie stukje bij beetje de taken gaan verdelen, uiteindelijk is er een pakket taken voor de teamleider gekomen?

**Respondent:** Ja, ja.

**Interviewer:** Als we dan kijken naar je tijdsbesteding in een week. Wat zou de verhouding zijn dat je aan het bed staat en ondersteunt?

**Respondent:** Ja, dat is een beetje raad verdeeld. Ik ben vooral in de vakantie meer inzetbaar in de zorg. Waardoor ik de andere delen van het jaar eigenlijk weinig aan het bed sta. De organisatie zegt volgens mij, d'r staat echt beschreven hoeveel uur per week zou moeten zijn. Dat weet ik niet uit mijn hoofd, dat is ook een beetje wat de afdeling toelaat en hoe het eruit ziet en waar de prioriteiten ligt en of dat haalbaar is met de collega's die aan het werk zijn. Maar dat wisselt ook een beetje met drukke en minder drukke perioden, dat ik meer in de zorg sta met vakantieperioden of met ziekte, dan dat ik standaard een halve dag per week standaard in de zorg sta. Zo hebben we dat niet ...

**Interviewer:** Dus het is meer incidenteel. En voor de vakanties en ziektes meer stelselmatig?

**Respondent:** Ja, zo kom ik wel redelijk aan zorguren. En ik vind dat redelijk belangrijk ook, ik vind dat ook wel. Ik vind het heel goed om met je collega’s zeg maar weer, natuurlijk ben je naaste collega, om zelf maar echt in de zorg te staan, door dingen te merken, te ontdekken, te horen. En opzicht doe ik dat hoor. Ik zit wel heel veel hier, maar ik zit ook heel veel op kantoor, dus ik weet wel precies wat er speelt op de afdeling, maar daadwerkelijk aan het bed staan is toch echt wel anders. Dan kom je toch weer dingen tegen.

**Interviewer:** Weer net een laagje dieper dan ...

**Respondent:** Ja, ja. Dat onderhoudt ook weer het contact me je collega's. Dus dat.

**Interviewer:** We hebben dan net, tussen neus en lippen door, wat HR-gerelateerde taken voorbij horen komen. Zou je eens kunnen opschrijven wat jouw HR-takenpakket is?

**Respondent:** Het is ook niet dat ik dat helemaal op mij neem, maar dat overlapt ook wel met >BEDRIJFSLEIDER<. Ik overleg ook wel heel veel met >BEDRIJFSLEIDER< , ook wat HR betreft. Dan kunnen we dan ook nog bepalen van 'ik leg dit nu bij jou neer, of ik doe dit'. Dat doen we ook veel in overleg. Ik doe jaargesprekken samen met >BEDRIJFSLEIDER<. >BEDRIJFSLEIDER<  doet de helft van de gesprekken, ik de andere helft. En ja, zoals ik al zei, als we het hebben over functioneren, dan ben je constant feedback aan het geven en aan het kijken hoe collega's functioneren. Maar collega's komen ook bij mij als ze of privé niet lekker zitten, of met samenwerking niet lekker zitten, of nu met heel veel ontwikkelingen met digitaal zeg maar, dat wat oudere verpleegkundigen die lopen daar tegen aan en die vinden dat best wel moeilijk. Dus dat we daar echt wel aandacht aan besteden. Dus daar luister ik naar en kijk ik van 'joh, wat kan ik jullie bieden of hoe kun je zorgen dat je het nog wel redt tot je pensioen, zeg maar?'. Want ook dat elektronisch verpleegkundig dossier komt er aan. Dus die hebben echt nog wel wat voor de kiezen. Dus zulke dingen ook. Echt de oudere verpleegkundigen, dat we daar rekening mee houden van 'goh, lukt het allemaal, kunnen we ze anders inzetten?'. Dus daar speel ik een rol in. Noem nog eens een HR-dingetje?

**Interviewer:** Ik zal je kunnen helpen. Ik heb eens opgezocht waar een teamleider zich mee bezig zou kunnen houden op HR-gebied.

**Respondent:** Werving en selectie natuurlijk.

**Interviewer:** Wat doe je dan precies?

**Respondent:** Samen met, de vacature die stellen >BEDRIJFSLEIDER< en ik samen op. We zitten samen bij het sollicitatiegesprek. Dus dat doen we eigenlijk samen. Geen idee of dat op andere afdelingen anders is, maar dat vinden wij prettig zo. Dat doen we zo. Introductie van nieuwe collega's, dat hebben we weggelegd bij, ik wijs dan twee coaches aan op de afdeling. En tuurlijk, heet ik wel iemand welkom en is het vaak de eerste dag de afdeling laten zien. Maar vooral, wijzen we twee coaches aan van >AFDELING< samen. Die hebben dan nauw contact. En tuurlijk vraag ik 'hoe gaat het' en houd ik het in de gaten. Maar die introductie hoef ik niet helemaal zelf te doen. Bijhouden van personeelsbestanden, dat doen we eigenlijk samen ook. >BEDRIJFSLEIDER<  en ik. Wat is de formatie, zitten we er boven of er onder, hebben we veel flex ingezet of niet? Dus dat doen we ook wel samen. Ook de roosters doen we nog wel samen, omdat we een nieuw systeem hebben. Ik weet niet of >BEDRIJFSLEIDER< jou dat verteld heeft, zelfroosteren.

**Interviewer:** *Interviewer lacht*

**Respondent:** Waarom lach je?

**Interviewer:** >tekst geschrapt vanwege identiteit ziekenhuis<.

**Respondent:** Ik heb ook wel eens vakantie en kan niet alleen weten hoe het werk. Daarom doen we dat ook samen. In het begin moesten we allebei natuurlijk even wegwijs worden en weten hoe het werkt. Nu wisselen we met het rooster een beetje af en we zitten ook vaak samen aan het rooster om het af te ronden. Maar ja, het rooster wordt eigenlijk samen gemaakt door de verpleegkundige zelf. En wij tikken hem af. Dus dat doen we vaak samen. En het kan ook zo zijn dat er diensten missen, of, over het algemeen als ik er ben kijk ik hier naar het personele bestand: zijn er morgen genoeg poppetjes, de rest van de week, moet ik wat aanvragen bij de flex, moet ik wat ruilen? Dan kijk ik hier naar de personele bezetting. Maar, ik ben ook wel eens vrij dus dan doet >BEDRIJFSLEIDER< dat. Mochten er dingen zijn, dan overleg ik met >BEDRIJFSLEIDER<  'denk jij dat het morgen kan met een poppetje minder?'. We overleggen wel heel veel. Over het algemeen is het wel mijn verantwoordelijkheid om daar naar te kijken. We hebben ook een heel rooster-planning-bord die is ook aan mij om die te maken. Dus dat wat betreft wat betreft de personeelsbestanden. En ook die trainings- en ontwikkelingsbehoeften, dat is ook in overleg met >BEDRIJFSLEIDER< . En tuurlijk hoor ik wel veel van de werkvloer. Het betekent niet dat >BEDRIJFSLEIDER< niets hoort van collega's. Ze komen ook wel bij van 'joh, dit lijkt me heel leuk om te doen, of ik zie bijvoorbeeld dat we iets met communicatie moeten doen, want daar hebben we dan de zoveelste klacht van'. Een punt wat ik doe, dat zijn de klachten. In overleg met patiënten en familie. En dan zeg ik 'ik denk dat het handig is als we eens een communicatietraining gaan doen'.

**Interviewer:** Komt het dan wel eens voor dat een medewerker jou overstijgt en gelijk door gaat naar Annie.

**Respondent:** Ja, dat komt voor. Maar dat is dan bijvoorbeeld als ik er niet ben. Maar dat betekent ook dat >BEDRIJFSLEIDER< nauw betrokken is bij de afdeling. Het is allemaal heel laagdrempelig. Als ik er niet ben, dan lopen ze bij >BEDRIJFSLEIDER< binnen en roepen ze het alvast. Maar ja, dat hoor ik dan gelijk van >BEDRIJFSLEIDER<. Dan weet ik dat ook. Dan zegt >BEDRIJFSLEIDER< tegen mij 'Pietje was er'. Dus dat gaat eigenlijk nooit langs elkaar heen. Maar over het algemeen ben ik aanspreekpunt op de afdeling en komen ze vaak bij mij, maar dat betekent niet dat ze niet bij >BEDRIJFSLEIDER< komen.

**Interviewer:** Als we het lijstje verder aflopen zijn er dan nog bepaalde punten?

**Respondent:** Nee, communicatie hebben we het over gehad. De rest hebben we wel besproken.

**Interviewer:** Als we kijken naar je bevoegdheid als teamleider. Kun je aangeven over wat voor zaken jij zou mogen beslissen?

**Respondent:** Dat is natuurlijk heel breed. En daar is ook wel grijs gebied in. Als we het hebben over de aanschaf van dingen. Je hebt dingen die tien euro kosten, waarvan ik denk 'dat doe ik, daar teken ik voor'. Maar ja, een apparaat van 6.000 euro, daar ga ik niet over beslissen. Dus dat weetje, aanschaf van kleine dingen daar kan ik over besluiten. Hoewel >BEDRIJFSLEIDER< daar eindverantwoordelijk in blijft. Maar, dan weet ik wel dat ik dat zonder overleg kan doen. Noem een voorbeeld.

**Interviewer:** Heb je een drempel voor wat je financiële bevoegdheid betreft? Heb je een drempel van 'tot hier teken ik en alles daarboven gaat naar >BEDRIJFSLEIDER<'?

**Respondent:** Nee, daar hebben we geen bedrag voor afgesproken. Maar er komen ook geen dingen uit de lucht vallen van een dure aanschaf, zonder dat we het niet weten. Want voor die tijd bespreken wij van 'goh, volgens mij is het wel wijs dat we een nieuwe douchestoel aanschaffen'. Dan gaan we het daar over hebben en krijgen we daar een offerte van. Dat stuur ik dan naar >BEDRIJFSLEIDER< en zeg ik 'joh, het gaat dit kosten, gaan we dat doen?'. Dus aanschaffen komt niet uit de lucht vallen. Het is ook nooit acuut nodig. In ieder geval niet voor de grote bedragen. Dus het is ook nooit voorgekomen van: mag ik dit nu, of mag ik dit nu niet?

**Interviewer:** Mocht je dan in grijs gebied komen, dan overleg je met >BEDRIJFSLEIDER<?

**Respondent:** Ja, het is ook niet nodig om daar een heel zwart-wit afspraak over te maken.

**Interviewer:** Maar mocht je geen goede werkrelatie hebben met >BEDRIJFSLEIDER<, dan zou een zwart-wit afspraak beter zijn?

**Respondent:** Ja, en zeker als de bedrijfsleider weinig op de afdeling te vinden is, of altijd in overleg zit en zegt 'een keer in de maand hebben we een overleg'. Dan zou ik zeker willen weten wanneer ik iets mag tekenen en wanneer niet. Dan moet je dat weten, maar dat mis ik nu niet.

**Interviewer:** Als je kijk naar jouw rol als teamleider en de HR-afdeling, waar zit hem dan het verschil in.

**Respondent:** Het verschil is dat ik probeer heel laagdrempelig te zijn. Dat collega's heel snel naar mij toekomen en hier in gesprek gaan en hier eruit proberen te komen. En mocht dat niet lukken, of ik denkt 'hé, ik moet hier advies over hebben', dan schakel ik HR in. Dan bel ik HR en zeg ik 'joh, dit is wel ingewikkeld'. Ook wel eens met een ziekmelding, maar meestal komen we daar wel uit, dat kan ik wel zelf. En soms ga ik naar >BEDRIJFSLEIDER< en zeg ik van 'goh, wat moet je hier nu mee, met deze ziekmelding?'. En soms komen we er niet uit en dan bellen we HR en zeggen we 'het zit zus en zo', ik geef nu een voorbeeld van ziekmeldingen, 'en die collega is er nog steeds niet, in hoeverre, hoe gaan we hier nu mee om?'

**Interviewer:** Dus als het je kennen en kunnen overstijgt, ga je naar HR?

**Respondent:** Ja, dan willen we advies hebben. Ja, ook bij langdurige ziekte.is sowieso HR betrokken, dan ga je een traject in en stuur HR advies van 'joh, we gaan een plan van aanpak maken', dat zie je dan ook in het systeem. We hebben dan wat nauwer contact en dat sturen ze ons ook van 'we hebben een gesprek gehad'. Dan wordt HR daar ook bij betrokken. En opzicht, is voor het personeel HR ook wel laagdrempelig en kunnen daar binnen lopen en zeggen 'joh, ik wil het eens even over mijn teamleider hebben, daar kan ik niet mee uit de voeten'. Dus ook voor het personeel is HR uitkomst.

**Interviewer:** Als het aankomt op beleid, maak jij dan je eigen personeelsbeleid of kopieer je eigenlijk wat er uit HR komt, en implementeer je dat hier in de afdeling.

**Respondent:** Ik zit even te denken. We hebben sowieso een maandelijks overleg met HR. Daar hebben niet zo zeer over beleid, maar hebben we het meer over alles wat er loopt, betreffende het personeel. Dus elke maand zien we iemand van HR, spreken we iemand van HR, dus kunnen we bespreken van 'goh, dat loop of goh hier wil ik advies over'. Maar als het hebt over het personeelsbeleid, tuurlijk is er dan vanuit HR een idee of functieprofielen. Maar dan is het aan ons om te kijken 'hoe gaan we dat hier invullen?'. Of advies vragen aan Opleidingen, of aan HR. We hebben hier bijvoorbeeld ook nog twee verzorgenden. Wat is nou wijs, hoe ga je daarmee om, wat voor functieprofiel hebben zij, wat moeten zij doen? Wij hebben natuurlijk een bepaald aantal FTE wat we mogen inzetten, maar opzicht ben je daar wel vrij in hoe je dat dan invult. Dus dat is niet helemaal kopiëren van wat HR vindt. We passen dat aan de behoeften hier en hoe de afdeling er uit ziet.

**Interviewer:** Heb je ook het idee van 'dit overstijgt echt mijn HR-kennis, om het even zo te noemen, dan ga ik naar HR toe?

**Respondent:** Ja, klopt.

**Interviewer:** Heb je het idee dat je bepaalde kennis en kunde moet bezitten jouw rol als teamleider uit te kunnen voeren.

**Respondent:** Ja, zeker. Hoe leg ik dat uit. Een stukje communicatie. Hoe leg ik dat uit. In ieder geval met mensen, daar hoe je helemaal geen HR-kennis voor te hebben, dat je met mensen in gesprek kan als ze tegen dingen aan lopen of dat je feedback kan geven. Echt communicatieve vaardigheden, ik denk dat dat een hele belangrijke is. Volgens mij kom je dan heel ver. Als je maar gewoon met elkaar kan praten en je zegt waar het over gaat. Tuurlijk, als het de oudere verpleegkundigen aangaat. Heel veel kun je zelf verzinnen. We hebben bijvoorbeeld een poli die verpleegkundigen soms draaien. Je kunt soms praktisch kijken van 'joh, helpt het jou een dag in de week die poli gaat doen, even wat anders en niet zoveel prikkels?'. Heel veel kun je wat dat aangaat zelf. Maar ik heb bijvoorbeeld wel, bij langdurige zieken heb je dan een plan van aanpak dat je moet doe. Dat vind ik dan, hoe gaan die regeltjes dan ook weer? Dat zijn meer dingen, die kennis moet je dan hebben, maar dat kan ik prima bij HR vragen. Hoe zit het ook al weer met dat UWV, ingewikkelde dingen. Die heb ik niet zo vaak.

**Interviewer:** Dus eigenlijk heb je hele pragmatische kennis nodig?

**Respondent:** Ja, grotendeels wel. Tuurlijk moet je de procedures wel een beetje weten. Hoe gaat het met ontslag? Mag je zomaar iemand ontslaan? Hoe zit het met die twee maanden opzegtermijn? Of met ziekmelding, wat is er vanuit huis, wat staat er vast. Dat moet je wel weten.

**Interviewer:** Hoe ben je daarvan op de hoogte gekomen. Heb je bepaalde documenten waar je inzicht inhebt?

**Respondent:** Ja, bijvoorbeeld. In het begin heb ik ook gewoon veel aan HR gevraagd. We hebben ook een ontwikkeltraject in het ziekenhuis gehad. Daar krijg je ook veel HR. En het is ook wel initiatief van jezelf om daar ook een actieve rol in te hebben en dingen op te zoeken. Ik heb ook nog wel eens, ik kan even geen voorbeeld noemen, maar iets dat gewoon niet veel voorkomt. Dat je dan denkt van 'hoe moet dat ook al weer'. Dan duik ik even in de CAO of ik duik in protocollen of documenten van HR en dan kom je er uit.

**Interviewer:** Heb je als teamleider ook nog iets waar je voor gaat? Een soort doel die je als teamleider wil bereiken. Bijvoorbeeld tevreden personeel of goede zorg. Waar zet jij je voor in?

**Respondent:** Die vind ik heel belangrijk. Kwaliteit van zorg. Die vind ik heel belangrijk. Tevreden patiënten. Dat bereik je door kwaliteit van zorg, maar ook door tevreden medewerkers. Dus je wil ook gewoon tevreden medewerkers die hele goede zorg leveren. En daar valt eigenlijk alles onder. Alles wat hier reilt en zeilt. Dat zijn de hoofditems wat je wil. Dat ze hier heel blij aan het werk zijn en dat patiënten ook heel blij worden van de zorg.

**Interviewer:** Als we dan kijken naar de uitvoer van je taken, ligt heel strak vast wat je moet doen of wanneer je iets moet doen? Of is het juist dat >BEDRIJFSLEIDER< of HR heel erg loslaten van 'we hebben wel kaders, maar hoe je dat invulling geeft, dat is aan jou'?

**Respondent:** Er zijn wel kaders. Je hebt een functieprofiel en er zijn verwachtingen vanuit de afdeling en verwachtingen vanuit >BEDRIJFSLEIDER<. Maar ik word daar heel vrij in gelaten hoe ik dat invul en wanneer ik dat invul.

**Interviewer:** Hoe ben je van die verwachtingen op de hoogte? Krijg je dat dan via een gesprek door of houd je je vast aan je functieprofiel?

**Respondent:** Nee, ik houd mij niet heel erg vast aan mijn functieprofiel hoor. Ik houd mij vooral vast aan datgene dat vanuit de organisatie van jou verwacht wordt, wat de doelen zijn zeg maar. Bijvoorbeeld het jaarplan van het ziekenhuis, maar ook ons jaarplan. Opzicht is >BEDRIJFSLEIDER< dan ook duidelijk, of wij dan duidelijk, wat de doelen zijn in ons jaarplan. Doelen vanuit >BEDRIJFSLEIDER< en vanuit de organisatie. En soms verandert dat hoor per maand. Dan is dit prioriteit en dan weer dat. Ik krijg wel duidelijk doelen te horen, maar ik kan zelf bepalen hoe ik invulling geef aan het behalen van die doelen. Ik vind dat ik daar redelijk vrij in gelaten wordt.

**Interviewer:** Heb je nog invloed op de doelen, of zijn deze vooral top-down bij jou neergelegd? Of mag jij ook nog, zeg maar, een soort advies over die doelen?

**Respondent:** Ja, zeker. Dat jaarplan doet ik samen met >BEDRIJFSLEIDER<. Dus het wordt nog op mijn bordje gegooid er is samen in overleg van 'waar zijn behoeftes en waar gaan we komend jaar naar toe'. En >BEDRIJFSLEIDER< verwacht dat ik met dingen kom vanuit de afdeling van 'hier kunnen we mee aan de slag en volgens mij moeten we daarmee aan de slag'. Dus vanuit mijn kan komen er ook doelen en die toets ik bij haar. En >BEDRIJFSLEIDER< zeg van 'het is onzin' of 'ga er mee aan de slag'. En tuurlijk vanuit de organisatie en vanuit de IGZ zijn er doelen. Daar moet je aan voldoen, punt. Niet altijd, maar dat zijn soms hele duidelijke kaders waar je aan moet voldoen. En tuurlijk, ben je daar wel een beetje vrij in van 'hoe ga je zorgen dat je dat doel behaalt', maar die doelen zijn gewoon heel strak.

**Interviewer:** Mijn laatste vraag zou eigenlijk zijn 'hoe jij je als teamleider zou moeten gedragen in de ogen van een bedrijfsleider of HR'? Wat zouden van jou gedragingen verwachten?

**Respondent:** Ik moet het goede voorbeeld zijn, sowieso. Ik moet open zijn en laagdrempelig voor personeel, voor verpleegkundigen. Ik moet zichtbaar zijn op de afdeling. Ook een vraagbaak voor de collega's. Ik ben ook een soort tussenpersoon tussen het team en >BEDRIJFSLEIDER<. Dat betekent niet dat ze niet op de afdeling komt en niets zelf ziet, maar toch wel een soort doorgeefluik, klinkt negatief maar dat bedoel ik helemaal niet zo, ook wel iemand die dingen communiceert op de afdeling. Dus collega's motiveert, maar ook informeert. En mijn gedragingen, ja, ik moet het voorbeeld zijn. Communicatie-sterk. Ik denk ook neutraal in je ...

**Interviewer:** Dat je in het team staat?

**Respondent:** Ook wel. Nee, nee. Ik kan het niet goed benoemen. Verdere gedragingen. Dat ik mij houd aan de regels en, wat ik zei, het goede voorbeeld zijn. Een luisterend oor. Goed luisteren en kijken wat voor signalen je krijgt. Die een beetje. Duidelijk.

**Interviewer:** Als ik hetzelfde aan HR zou vragen, zou je weten van ze van jou verwachten als teamleider in de organisatie?

**Respondent:** Ik denk, maar dan vul ik misschien zelf in, denk dat HR meer kijkt naar het functieprofiel, wat goed is hoor, maar daar staan vast dingen in die ik niet genoemd heb nu. Dus die zullen wel echt die dingen noemen, operationeel op de afdeling. Vooral op de afdeling aanwezig. Kijkend naar kwaliteit, naar resultaat, goede zorg, personeel wat blij is zeg maar. Daar ga ik van uit dat zij dat van mij verwachten.

**Interviewer:** Maar in de praktijk is jou rol dus veel breder dan dat zij misschien voor ogen zouden hebben?

**Respondent:** Nee, dat weet ik niet. Geen idee. Ik denk wel, dat als je je doelen wil bereiken, dat je er niet aan ontkomt om breed te gaan. Dat zal ook per teamleider verschillen hoe je je inhoudelijk op dingen stort of heel erg een helicopterview hebt. Dat wisselt bij mij per onderwerp. Ik zit wat meer in de protocollen in het project EVD. Daar stort ik mij wat meer in. Maar sommige dingen laat ik dan aan collega's over en ik hoor dan hoe dat gaat.

**Interviewer:** Je heb mij goed mee kunnen nemen in jouw rol van teamleider. Ik weet niet of er nog dingen zijn die jij toe zou willen voegen aan het interview.

**Respondent:** Nee. Ik weet niet of je nog dingen wil weten, of dat je dingen mist?

**Interviewer:** Eerder uit persoonlijke nieuwsgierigheid. Is het moeilijk om een teamleider te zijn? Ik kan me namelijk zo voorstellen dat, ook omdat je aan bed staat, sta je zowel tussen het personeel en soms moet je hiërarchisch boven het personeel staan. Je bent en blijft een teamleider.

**Respondent:** In het begin vond ik het lastig om mijn plek duidelijk te maken en te vinden wat er bij mij past. Maar ik vind dat nu helemaal niet moeilijk. Maar dan moet ik zeggen dat ik een heel erg prettig team heb om in te werken en die mij ook de ruimte geven van 'joh, laat maar zien hoe jij dat gaat invullen'. Dat heb ik ook wel, dat heeft ook echt wel meegeholpen. En dat ik een hele prettig bedrijfsleider heb om me samen te werken. Die geeft mij de ruimte en heeft mij in het begin ook heel erg de ruimte gegeven van 'kijk hoe je het invult en kijk hoe het bij je past'. Dat voorkomt niet dat het soms wel lastig is hoor. Tuurlijk heb je wel eens dingen, het is wel eens lastig. Over het algemeen gaat het heel goed. Maar dat komt ook door het prettige team. Ik zit hier wel goed op mijn plek.

**Interviewer:** Wat goed, inspirerend.

*Einde transcript.*

# Transcript Interview FLM 4

**Duur interview: 54 minuten en 47 seconden**

**Functie: Teamleider**

**Afdeling(en) onder supervisie: Healthcare**

**Gender: man**

**Werkervaring in functie: 4 jaren**

**Interviewer:** Maar je denk dat er grote verscheidenheid tussen de teamleiders zit?

**Respondent:** Dan denk ik niet, dat weet ik zeker.

**Interviewer:** Waar zit het verschil in?

**Respondent:** Het verschil zit hem met name in opvattingen wat je rol en je positie als teamleider inhoud en hoe je hem invulling geeft. Want ik heb destijds toen ik solliciteerde voor de functie, heb ik ook heel duidelijk gemaakt hoe ik de rol zou invullen. Dat is de manier hoe ik wil werken. Die wijkt af van de wat op dat moment de norm was.

**Interviewer:** In wat voor opzicht zou die afwijken?

**Respondent:** Nou, als je al naar me kijkt zie je al een heel groot verschil. Het is namelijk dat ik in een uniform loop. En dat zie je niet veel.

**Interviewer:** Veel teamleiders die veel in burger lopen?

**Respondent:** Ja, alleen in noodgevallen. Dan moeten ze zich nog omkleden of. Ik ben gewoon oproepbaar en als men mij nodig heeft dan men mij en dan ben ik er.

**Interviewer:** Is er bij jou een scheiding te maken tussen managementfunctie en zorgfunctie?

**Respondent:** Nee. Ik noem mijzelf een operationeel manager waarbij ik, de naam zegt het al en dat vind ik het aller belangrijkste, een teamleider moet een team leiden. En hoe leid je een team? Door contact met ze te houden en niet jezelf af te zonderen van het team. En dat is mijn visie en mijn kijk op de functie. Op het moment dat je jezelf opsluit in een kantoor en je gaat je bezig houden met bellen en cijfers en andere narigheden en je trekt je witte pak uit, dan ben je de verbinding kwijt.

**Interviewer:** Dus jij staat echt midden in het team als ik het goed hoor?

**Respondent:** Ja. Klopt.

**Interviewer:** Is dat dan niet lastig voor jou. Je bent en blijft een teamleider dus er komt ook een moment dat je...

**Respondent:** Harde besluiten moet nemen.

**Interviewer:** ...er boven moet gaan staan. Je bent en blijft verantwoordelijk voor hoe het team doet.

**Respondent:** Dat is hoe je er mee omgaat. Men heeft mij wel een beticht van natuurlijk leiderschap. Ook zeg maar de manier hoe ik opereer op de afdeling en hoe dat ik mij beweeg op de afdeling. Het is namelijk niet zo dat ik de hele dag aan het werk ben op de vloer. Maar als men mij nodig heeft, dan ben ik bereikbaar. De eerste drie maanden. Dat heb ik ook heel bewust gedaan. Want er was ook nooit een teamleider geweest op die afdelingen. Daar werd alleen met seniors gewerkt. Dus ik moest voor het eerst die rol gaan invullen en het team was een beetje verdeeld. Er waren twee teams. >TEAM1< en >TEAM 2<. En van die twee teams, die moesten weer met elkaar in verbinding komen. En uiteindelijk ben ik er tussen gaan staan en heb ik overal gezegd 'zeg maar wat ik moet doen, zeg het maar'. Voor de eerste drie weken heb ik mij helemaal de rambam gewerkt. Tot dat iemand zei '>TEAMLEIDER< heb jij geen ander werk?'. Ik zeg 'jawel, maar als jullie mij nodig hebben, dan ben ik er voor jullie'. Maar wij vinden het nu belangrijk dat jij je met andere taken bezig gaat houden. Want die blijven ook liggen. En ik zeg 'dat klopt'. En het mooie en krachtige ook is, als ik bepaalt wat zij moeten doen, dan is dat minder effectief dan wanneer collectief bepaald wordt wat belangrijk is voor de afdeling. Dat is vele malen krachtiger. En als je het hebt over de effectiviteit van het managen, dan is een collectief uitgedragen besluit honderd malen effectiever dan wanneer iets top down gezegd wordt 'jij gaat dit doen'.

**Interviewer:** Je geeft wat dat betreft medewerkers de vrijheid om zelf besluiten te kunnen maken?

**Respondent:** Ja. Klopt.

**Interviewer:** Is dat niet lastig als men zoveel vrijheid krijgt, dat er besluiten genomen worden, of op een manier gewerkt wordt die je niet aan staat.

**Respondent:** Nee. Ik noem het altijd vrijheid in gebondenheid. In die zin dat de kaders op een afdeling wel helder zijn. En de regels ook. Dat betekent dat als het gaat over invulling geven aan de werkwijze die de NIAS voorstaat of regelzaken, dan is dat leidend. Maar dat zijn de kaders. Maar de wijze waarop je daar invulling aan geeft. Er zijn meerdere wegen die naar Rome leiden. Ik hoef jou als professional niet te gaan vertellen op welke wijze jij dat en dat moet doen als dit de regels zijn.

**Interviewer:** Wordt dat door iedereen fijn gevonden, of merk je dat sommige collega's juist mee houvast nodig hebben.

**Respondent:** Die neem je ook wat mee. Of die zorg je dat in alliantie met een collega kunnen werken dat ze daarin meegenomen kunnen worden. Dat is ook leren zeg maar.

**Interviewer:** Is dat dan ook een rol die bij jou ligt dat enigszins coachen?

**Respondent:** Klopt. Ja. Dat is ook wel waar mijn kracht en specialiteit ligt. Ik doe dat ook privé. Ik heb daar ook een eigen bedrijf in.

**Interviewer:** Als we kijken naar jouw werkzaamheden. Ik hoorde net ook al een stukje meewerken. Hoe zien jouw werkzaamheden eruit?

**Respondent:** Ik heb geen dagroutine.

**Interviewer:** Is er een algemene rode lijn die er doorheen gaat?

**Respondent:** De algemene rode lijn die er doorheen gaat is dat ik in de ochtend de overdrachten aanhoor op de afdeling en de unit waar het het drukste is. Dan kijk ik ook even naar het personeelsoverzicht van hoe die dag en de dagen erna ligt. Dan weet ik ook waar mijn prioriteit ligt. De continuïteit op de afdeling, dat is prio 1. Op het moment dat dat duidelijk is, loop ik altijd even naar de kurenkamer. Want behalve dat we een >AFDELING< hebben en een >AFDELING< hebben, hebben we ook de Kurenkamer en de Dagbehandeling. Ik heb zo een beetje 45 man aan te sturen. Dan ga ik daar even inventariseren of daar nog problemen zijn en hoe de werkdruk is en als ik dat op een gegeven moment in mijn hoofd in kaart heb, dan weet ik of ik actief mee moet helpen op de afdeling en weet ik wanneer ik wat moet doen operationeel. En voor de rest ben ik over dag, wat het werk aan gaat, een troubleshooter. En dat betekent. Mijn voorland is intensive care en spoedeisende hulp. Dus wanneer de lastige infusen te prikken zijn, dan wordt ik altijd opgetrommeld. Dat moet ik altijd doen. Dus dat is eigenlijk een beetje problemen opvangen in praktische zin.

**Interviewer:** Ik heb dan een HR-achtergrond. Ik kan me voorstellen dat, zeker als je 45 man aan te sturen hebt, dat daar ook een stukje personeelsmanagement bij komt kijken. Aan wat **Respondent:** voor taken moet ik dan denken?

**Respondent:** Dan moet je vooral denken aan primaire sollicitatiegesprekken. De verzuimgesprekken. De jaargesprekken. En de functioneringsgesprekken.

**Interviewer:** Dus alle gespreksvoering ligt bij jou?

**Respondent:** Als het gaat over HR-taken wel.

**Interviewer:** Ik heb eens opgezocht waar een teamleider. Wat bij een teamleider zou kunnen liggen qua HR-taken. Zij er nog er nog bepaalde punten die er van dit lijstje voor jou uit springen?

**Respondent:** Op dit moment is dit een hot topic. Werving en selectie.

**Interviewer:** Wat is jouw rol daarin?

**Respondent:** Alles. Van tot de brief binnen komt en tot het moment tijd wij besluiten wel of niet iemand aan te nemen.

**Interviewer:** Jouw stem is wat dat betreft bindend of het houdt de bedrijfsleider het laatste woord?

**Respondent:** We hebben bedrijfsleider die volgt wat ik aangeef.

**Interviewer:** Een ja of nee valt of staat met jou?

**Respondent:** Als ik nee zeg, zegt de bedrijfsleider geen ja. Maar dat ligt ook aan de manier waarop wij samenwerken. Dat nog wel eens verschillend. Wij werken nog net duaal samen. Maar dat scheelt niet veel. Ik heb ook mijn HBO managementopleiding gedaan. Daar speelt het ook in. Het kennisniveau in het ziekenhuis. Dus je hebt wel dezelfde functie maar niet hetzelfde opleidingsniveau. Dus daar zit ook verschil in.

**Interviewer:** Merk je dat jij door jouw HBO managementopleiding meer vrijheid krijgt van je bedrijfsleider?

**Respondent:** Nee, dat had ik al.

**Interviewer:** Waar is dat van afhankelijk. Het lijkt me geen vanzelfsprekendheid?

**Respondent:** Nee. Dat is geen vanzelfsprekendheid. Ik ben acht jaar voorzitter geweest van de OR. En mijn bedrijfsleider heeft in de OR gezeten en onder mijn voorzitterschap. >BEDRIJFSLEIDER< wist hierdoor precies hoe ik handel en wat mijn manier van werken is. En dat is niet vanuit macht maar vanuit kracht. En dat is nog wel een groot verschil. Positionele macht ben ik altijd een beetje dwars van.

**Interviewer:** Het komt aan op de dingen die je doet?

**Respondent:** Als je iets afdwingt bij een medewerker en je zit niet naar hem te luisteren en je gaat op je strepen staan, dan hoef je niet over een week te vragen 'zou jij misschien dit voor mij willen doen?'. Dan krijg je 'zoek het uit, een fijne dag verder'.

**Interviewer:** Als we dan kijken naar de besluiten die jij mag nemen. Je over best veel dingen beslissen. Kun je misschien iets meer vertellen over de dingen waar jij als teamleider over mag beslissen?

**Respondent:** Meestal de meest praktische zaken. Als iets met een student niet goed gaat. We hebben onlangs op de opleiding een studenten die we gewoon dezelfde dag nog de deur uit hebben gezet. Dat soort dingen. Dat wordt letterlijk ook gewoon gedekt. Op dat moment is er gewoon, dat is wederzijds, het volste vertrouwen de besluitvorming die wij nemen. Ik in mijn bedrijfsleider en de bedrijfsleider in mij. En dat is ook denk ik een voorwaarde om te kunnen werken. Want als jij niet volledig jouw teamleider vertrouwt, dus dat je permanent moet controleren en aan het overrulen of aan het afhangen, dan klopt er iets niet. In het takenpakket dat je hebt moet je gewoon vrij kunnen handelen. En kan dat niet, dan is er iets niet goed.

**Interviewer:** We hebben het hier over een bedrijfsleider die jou al heel lang kent en jou heeft zien werken. Stel er zou een nieuw iemand op de stoel van bedrijfsleider komen, zou je dan nog steeds diezelfde verantwoordelijkheden kunnen hebben?

**Respondent:** Dat weet ik niet.

**Interviewer:** Dat is dan afhankelijk van de relatie die je hebt met de nieuwe bedrijfsleider.

**Respondent:** Tuurlijk. De invulling die jij geeft aan je functie en aan je taak is heel erg persoonlijk. De ene gaat er in en draai een keer mijn zijn schouders en heeft het geregeld. En de ander heeft veel meer overleg nodig en afstemming. En meestal heb ik al iets gedaan voor mijn bedrijfsleider voordat de vraag gesteld is. En dan wil >BEDRIJFSLEIDER< dit vragen, dan heb ik dat al gedaan.

**Interviewer:** Denk je dat je meer verantwoordelijkheden hebt in verhouding tot andere teamleiders hier in huis?

**Respondent: I**k weet niet of ik meer verantwoordelijkheden heb. Ik weet wel dat ik meer vrijheden heb. En ik weet ook dat ik meer vertrouwen geniet.

**Interviewer:** Waar komt dat vertrouwen van?

**Respondent:** Puur door wat ik net uitleg.

**Interviewer:** Die opgebouwde relatie over meerdere jaren?

**Respondent:** Ja. En het feit dat wij qua persoonlijkheid ook niet zo veel verschillen. We zijn identiek qua denken en ook qua wat je normen en waardenkader is. We worden over dezelfde dingen boos. Van dezelfde dingen hebben we last. Dus was dat betreft is dat ook identiek. En dat is wat mij betreft ook wel uniek.

**Interviewer:** Denk je dat je daarom ook deze functie hebt gekregen, omdat er zo'n klik is met de bedrijfsleider?

**Respondent:** Dat weet ik niet. Maar het was drie en een half jaar geleden, toen kwam ik de bedrijfsleider op de gang tegen en toen zei ik 'die functie van teamleider, die is zeker al lang vergeven?'. Het was een vacature die al anderhalf jaar uit stond en al een paar keer ververst was. Nee die vacature staat nog steeds vacant omdat we nog steeds niet de goede kandidaat gevonden hebben. Toen zei >BEDRIJFSLEIDER< van 'goh heb je willen solliciteren?'. Daar ben ik toen wel over gaan nadenken ja.

**Interviewer:** En toen uit de OR gestapt?

**Respondent:** Toen uit de OR gestapt en binnen een week was het beklonken. En niet alleen bij de bedrijfsleider geniet ik vertrouwen. Ook bij de medisch specialisten, de voorzitter, het hele kader. En bij het team heb ik het echt moeten verdienen.

**Interviewer:** En hoe deed je dat, dat verdienen?

**Respondent:** Door het team te laten voelen wat de meerwaarde is van een teamleider op de afdeling.

**Interviewer:** Jullie maken gebruik van seniors?

**Respondent:** Ja, maar die zijn operationeel ingedeeld. En als je operationeel bent ingedeeld heb je misschien wel verantwoordelijkheden, maar je hebt geen formele bevoegdheden. En daar heb je last van op het moment dat er dingen besloten moeten worden. Dan moet je altijd weer gaan opschalen. En ik hoef dat niet te doen. Ik kan zelf besluiten nemen. Als ik zeg 'ik wil morgen een uitzendkracht', dan kom hij er. En een senior moet eerst toestemming gaan vragen en gaat het dan regelen. Ik kan het zelfstandig regelen. Dus wat dat betreft maakt het wat dat betreft veel vrijer.

**Interviewer:** Kun je eens onder woorden brengen wat een senior extra doet naar aan het bed staan?

**Respondent:** Puur kwaliteitszaken.

**Interviewer:** Dus puur op zorg gericht?

**Respondent:** Ja. Maar ook kwaliteit. Een BIM-commissie. De voorzitter van de BIM-commissie is een senior. We hebben iemand die verantwoordelijk is voor het maken van de roosters. Dat wordt ook nog gedaan door een senior. Dat zijn allemaal mensen die operationeel zijn. Dus als er een moment komt dat er een probleem is in het rooster, dan komt het op mijn bordje terecht omdat ik veel meer vrijheden heb om op te lossen.

**Interviewer:** Is het dan ook zo dat jij meer dingen gedaan kunt krijgen vanwege jouw hiërarchische positie?

**Respondent:** Ik denk het wel. Bepaalde zaken worden makkelijker geaccepteerd wanneer ik het regels dan wanneer een ander dat zou regelen. Misschien is het ook. Voordat ik die opleiding had gedaan was het ook al zo. Toen ik in de OR zat was het ook al zo. Wellicht heeft het ook te maken met persoonlijkheid of zo of de manier waarop je dingen verteld. Dat het overtuigend is en niet een twijfelverhaal of zoiets.

**Interviewer:** Heb je ook nog een financiële bevoegdheid?

**Respondent:** Zodra het gaat over personeelsmanagementuitgaven, dan is de bedrijfsleider degene die tekenbevoegd is. Dus als iemand een opleiding wil doen, dan is de bedrijfsleider degene die dat initieert. Als ik een aankoop wil doen, dan kan ik dat regelen.

**Interviewer:** Duidelijk. Zeker als we het hebben over personeelsmanagement. Wat vindt jij dat iemand een goede teamleider maakt?

**Respondent:** Dat is iemand die respectvol om gaat met mensen en zichzelf wegcijfert voor het belang van de afdeling.

**Interviewer:** Is dat niet heel moeilijk?

**Respondent:** Het is eigenlijk de meest moeilijke functie in een organisatie die er is. Je zit heel dicht op de werkvloer, maar je hebt te maken met het aansturende beleid van boven. En soms moet je impopulaire maatregelen zien te vertalen naar de werkvloer. En dan moet je ook de credits kunnen behouden. En dat is een kwestie van vroeg mensen in de processen meenemen en zorgen dat zij niet iets op hun schouder gegooid krijgen waar je zelf ook niet gelukkig van wordt. Je moet ook zelf bereid zijn je nek uit te steken. En als je dat niet durft en je gooit het naar binnen en trekt de deur achter je dicht, dan gaat het niet werken.

**Interviewer:** Komt het dan neer op communicatie?

**Respondent:** Ja. Ik maak ook iedere week een nieuwsbrief over dingen die nieuw zijn, of anders zijn of veranderen. Of beleid dat er aan komt of spannende dingen die gaan komen.

**Interviewer:** Vind jij dat je een doorgeeffunctie hebt? Dat dingen die in het team gebeuren weer naar de bedrijfsleider speelt?

**Respondent:** Niet in de zin van klikken. We hebben een open cultuur. Dat is letterlijk zo. De deur staat altijd open. Zowel bij mij als bij de bedrijfsleider. Wat ik wel merk dat sinds ik nu op de afdeling ben is dat is dat men wat minder naar de bedrijfsleider gaat en wat meer naar mij. Omdat ik wat dichter bij de mensen sta. Daar zie je nu wel een verandering in ontstaan sinds afgelopen jaar.

**Interviewer:** En mag jij ook besluiten nemen als het aankomt op het trainen en opleiden van personeel?

**Respondent:** Nee. Dat gebeurt meestal vanuit Opleidingen zelf. Maar als ik zie dat er behoefte is of er nood is, dan kan ik dat wel initiëren.

**Interviewer:** Ik ben wel benieuwd naar de verwachtingen die men heeft van een teamleider. En dan doel ik vooral op wat jij verwacht van jouw functie als teamleider. Als we dan eerst kijken naar jouw bevoegdheden. Waar ligt de grens volgens jou.

**Respondent:** Een bedrijfsleider kijkt vooral naar het organisatorische deel en het managementdeel. Dus als het gaat om het financiële plaatje, de jaarplannen. Daar geef ik wel input voor, maar daar ben ik niet verantwoordelijk voor. Daar ligt de scheidslijn. Ik voed wel, maar ik maak niet.

**Interviewer:** Is het een adviserende rol die ik voor mij moet zien?

**Respondent:** Ja. Qua besluitvorming ben ik niet degene die daar de klap op geeft dat is de voorzitter en de bedrijfsleider. Die zijn degene die de besluiten nemen. En de teamleider, ik zij de gek en de teamleider van de Polikliniek, wij deze degene die gevieren het managementteam van de afdeling vormen. Maar ieder vanuit zijn eigen context en vanuit zijn eigen perceptie.

**Interviewer:** Wat heeft HR voor waarde voor jou als teamleider?

**Respondent:** Ik denkt faciliteren. Dus ik denk of op het moment dat zaken spannend worden wanneer het gaat om arbeidsrechtelijke zaken of om hogeschool HR-werk gaat, dus personeelsmanagement, dat we een loket hebben waar we terecht kunnen.

**Interviewer:** Dus als je hele specifieke kennis nodig hebt, hele specifieke HR kennis?

**Respondent:** Dat niet alleen. Je kunt ook bedenken, als je een confl>AFDELING< hebt met iemand, of iemand heeft met jou een confl>AFDELING<, dat je een soort bedrijfsmediation. Dat HR daar ook een schakel in is.

**Interviewer:** Heb je het idee dat jij als teamleider nog bepaalde kennis nodig hebt om goed te kunnen functioneren?

**Respondent:** Op dit moment? Nee.

**Interviewer:** Of voor als er een nieuwe teamleider zou komen. Waarvan zou je dan hebben 'dit zijn randvoorwaarden'.

**Respondent:** Als voor mezelf spreek. Zou de organisatie er wel bij gebaat zijn om daar een eenduidige visie op die functie komt.

**Interviewer:** Denk je dat dat mogelijk is?

**Respondent:** Ja. De organisatie moet het alleen willen.

**Interviewer:** Ik zeg dat meer met het idee dat de verschillen tussen afdelingen groot kunnen zijn, en de manier waarop deze gestuurd worden, dat deze zo groot zijn dat er moeilijk eenheidsworst van gemaakt kan worden.

**Respondent:**	Het hoeft geen eenheidsworst te worden. Het gaat erom dat je macroscopisch mensen begeleidt in die functie. Want het is echt in alle oprechtheid de lastigste functie in een organisatie. Je zit er echt overal als een sandwich tussen. En dat betekent ook dat dat in je brein en emotioneel wat met je doet. Je zit altijd. Het zijn hele lastige dingen. Waar ben je dan open over en wat leg je in het team? En wat niet? En hoe ga je om waar je bedrijfsleider last van heeft? Ga je dat uitleggen aan je team? Dat zijn allemaal vraagstukken waar een beginnende teamleider mee te maken krijgt. Ik begon destijds in de OR en dat was een grote losgeslagen bende en er was niets meer van over. Allemaal losse individuen. En uiteindelijk is dat in acht jaar tijd een goede groep geworden die in goede cohesie samen kan werken. Maar daar is wel wat voor nodig. Dat is namelijk niet dat het gaat om de voorzitter, het gaat ook niet om de vicevoorzitter. Het gaat om de OR als collectief. En zo probeer ik ook een afdeling aan te sturen. Het gaat niet om jou als verpleegkundige. Het gaat ook niet om jou. Wel als je ergens last van hebt, maar niet als je de producten levert. Ons product is gezondheid en goede zorg leveren aan mensen. En als je je daar als collectief verantwoordelijk voor maakt, dan maakt het geen bal meer uit wie wat doet op de afdeling. En soms wordt onderscheid nog wel gemaakt van 'ik ben een senior, of ik ben de gespecialiseerd verpleegkundige, of ik ben de teamleider, of ik ben de leidinggevende'. Snap je?

**Interviewer:**	Dan ga je gelaagd?

**Respondent:**	Dan ga je gelaagd. Het belangrijkste is dat je het belang van de organisatie voorop gaat stellen. Je trekt het dan breder. Dan betrek je het niet alleen op de afdeling. Op het moment dat er een nieuwe teamleider komt, die gaat die functie naar zijn eigen goedheid invullen en je hebt twee mogelijkheden. Of het gaat goed. Of hij kruipt weg als het spannend wordt en doet de deur achter zich dicht en gaat hij achter zijn computer zitten. Wat ik echt denk vanuit mijn hart. Is dat een goed traineeship een coaching van mensen waarvan je denkt 'dit is de manier van hoe wij vinden dat wij graag willen werken', dat zou ik heel goed vinden. Nu worden mensen aangenomen die vroeger verpleegkundige waren en op die functie van teamleider gezet. Dat is het dan. Succes.

**Interviewer:**	Terwijl je net zeg dat we het hebben over een functie die moeilijk in te vullen is.

**Respondent:**	Het zijn of mensen die binnen een jaar overspannen raken omdat ze de stapeling van problemen, niet alleen van boven maar van beneden, niet aankunnen. Je krijgt alles.

**Interviewer:**	Als ik nu hardop denk, kom je dan uit zoiets als een leiderschapstraining?

**Respondent:**	Het is niet alleen leiderschapstraining. Dan ga je het weer theoretiseren. Het is vooral veel coaching op de werkvloer en intervisie van elkaar 'wat heb jij meegemaakt, waar heb jij last van, waar loop je tegen aan?'. En hoe je daarmee om gaat en hoe je collega's daarmee omgaan.

**Interviewer:**	Heb je destijds ook deelgenomen aan de teamleidertraining in huis?

**Respondent:**	Nee. Toen zat ik midden in mijn managementopleiding. Daar heb ik toen vriendelijk voor bedankt.

**Interviewer:**	Ik was benieuwd of dat stukje dat je dan net noemde terug zou herkennen in de teamleidertraining. Maar, dat kunnen we dan niet weten.

**Respondent:**	Ik herken het niet omdat ik het niet zie. Als ik naar andere collega-teamleiders ook van de zorgafdelingen, bijvoorbeeld vanochtend bij het roosteren, dan ben ik de ene die in uniform ziet. De enige. Maar dan gaan die mensen wel vertellen dat het heel druk is op de afdeling. Dat begrijp ik dan niet. Waar ben jij? Waarom zit jij hier? Terwijl jouw bedrijfsleider er ook bij zit. Waarom ben jij niet bij je mensen?

**Interviewer:**	Ik denk dan even hardop. Dat heeft dan misschien ook te maken met in hoeverre een bedrijfsleider wil dat de teamleider operationeel inzetbaar is. In het functieprofiel staat...

**Respondent:**	25 procent. Maar hoe hard is dat? Ik ben 25 procent inzetbaar. Dat is voor mij een dag in de week. Dan rooster ik mezelf in op dinsdag daar staan er als zes dus dat maakt niet uit. Maar woensdag staan er maar drie in het rooster. Wat doe je dan? Ik heb dan zoiets van 'ik zet me nergens in het rooster en op het moment dat het druk is stap ik in'. Dat is een beetje van 'hoe flexibel ben je zelf?'. Ben je faciliterend bezig of ben je heel star bezig en zeg je 'dinsdag in het rooster, woensdag niet. Als jullie dan een probleem hebben, dan moet je het zelf maar uitzoeken'. Of 'wat denk je daar nu aan te kunnen doen'. Ook zo'n dooddoener managementuitspraak. Ik ben vooral een mensenmens. Ik vind het belangrijk als iemand in de ochtend gelukkig binnenkomt en in de middag ook weer gelukkig de deur uit gaat.

**Interviewer:**	Toen jij dan als teamleider begon destijds. De bedrijfsleider had destijds bepaalde verwachtingen van jou van 'goh, dit zou ik graag willen dat je doet, zo zou ik graag willen dat je het invult'. Hoe ben jij destijds van die verwachtingen op de hoogte gebracht?

**Respondent:**	Ja niet. Ik heb gewoon gezegd hoe dat ik die functie in zou vullen en daar hebben ze ja tegen gezegd.

**Interviewer:**	Er was geen moment van botsing met hoe de bedrijfsleider tegen de functie van teamleider aankeek?

**Respondent:**	Nee. Alleen dat het heel erg gewaardeerd werd. Dat het als prettig werd gezien en als ontlastend.

**Interviewer:**	Is er ook eens een moment geweest dat je HR-dingen moest doen dat je zoiets had van 'hoe moet ik dit in hemelsnaam aanpakken. Wat wordt er nou van mij verwacht?'.

**Respondent:**	Ja, ja. Dat weet ik goed. Mijn allereerste confrontatie met mijn teamleiderschap was een collega die disfunctioneerde en eigenlijk al bij niemand meer terecht kon vanwege gedragingen en uitingen. Toen kwam ik daar en werd gevraagd of ik dat dossier wilde overnemen omdat ik iemand leek die daarmee matchte.

**Interviewer:**	Ok. En toen. Je krijgt een taak, je weet niet hoe je het moet uitvoeren. Dus hoe heb je dat aangepakt?

**Respondent:**	Ik heb vooral met huidige kennis en ervaring gewerkt. Dat heb ik mezelf toen ook opgelegd voordat ik in functie kwam. Ik namelijk eerst een cursus linguïstisch programmeren. Daar heb ik ontzettend veel aan gehad. Ook leren wat er ook in het hoofd van anderen gebeurt als, of het nou positief of negatief is, en wat er ook gebeurt en hoe wij taal geven aan beelden. Wij hebben geen taal in ons hoofd. Wij geven taal aan beelden. Alles wat je zintuigelijk waarneemt, dat maak je woorden van dingetjes en beweginkjes. Dat krijgt taal. Daarom gaat het ook zo vaak mis in communicatie omdat wij vak het idee van elkaar hebben. De ander zou mij wel begrijpen, dat is omgekeerd ook zo. Alleen is het beeld niet altijd even duidelijk. Dat is hetzelfde dat ik jou hier nu iets probeer uit te leggen. En dan moet ik er maar vanuit gaan dat het voor jou helder en duidelijk is wat ik bedoel. Of ik mijn plaatje helder aan jou heb overgedragen.

**Interviewer:**	Maar was het destijds niet heel stressvol? Je moet die confrontatie aangaan. Je hebt wel een cursus gedaan en wel een idee.

**Respondent:**	Ik daarvoor ook leiding gegeven aan de OR. En als iets complex is, dan is dat het wel. Dat zijn vijftien mensen. Vijftien verschillende opleidingsniveaus. Met vijftien verschillende opvattingen. Dat moest teruggebracht worden naar een eenheid die uit een situatie kwamen van verdeel en heers kwamen waarvan de voorzitter de dienst uitmaakte. Die ging toen weg en er moest nieuw vuur komen. Toen zeiden ze van 'er moet nieuw vuur komen, dat moet jij doen'. Ik zei 'dat is prima, maar dan doen we het wel op mijn manier, dus we gaan het anders doen'.

**Interviewer:**	Ik ook duidelijk voor jou hoe je de HR-taken moet uitvoeren?

**Respondent:**	Ook daar heb ik mijn eigen weg in gevonden omdat men vanuit de organisatie het belangrijk vindt dat je elkaar aanspreekt en elkaar opzoekt om dingen uit te leggen en feedback te geven. Heb ik ook voor het type jaargesprek dat we nu doen bedacht toen het nieuwe formulier kwam dat mensen twee collega's mogen aanwijzen voor een feedbackformulier in te vullen. Dat is aan de hand van het functieprofiel. Die heb ik zelf gemaakt die formulieren en ik wijs ook iemand aan. Random. Om feedback te geven. Mensen komen bij mij op gesprek met de feedback van drie personen. En dat is ook de lijddraad van het gesprek. Dus dat maakt het veel waardevoller dan wanneer ik daar het gesprek ga houden vanuit mijn eigen referentiekader.

**Interviewer:**	Een soort peer-to-peercomponent is eraan toegevoegd?

**Respondent:**	Ja. En dat maakt het natuurlijk veel waardevoller. En de grap is dat iedereen in het begin faliekant tegen was en angstbeelden had. En ik zei 'jongens, laat het gewoon eens gebeuren en strakjes over een half jaar mogen jullie ervan zeggen wat jullie ervan vinden'. Vanwege de weerstand heb ik eerst de mensen uitgenodigd die daar geen probleem mee hadden. En wat bleek nou, ze kregen ook complimenten op papier. En wat verbaal eigenlijk nooit gebeurt. En ze zeiden 'ik vind het zo leuk, zo leuk, ik krijg gewoon een compliment'. Hoe fijn is dat? Wat vind je daar van? Dat vind ik harstikke leuk. Zeg dat dan ook eens tegen je collega dan. En uiteindelijk is het een klein olievlekje dat steeds groter is geworden. En nu is het ingesleten en vind iedereen het normaal. Natuurlijk wel lastig als je iets moet opschrijven waar een ander zich in kan verbeteren, maar daar heb ik ze in gecoacht. 'Ik weet niet hoe ik dat moet doen'. Dan moet je niet zeggen wat hij verkeert doet. Dan moet je opschrijven wat je graag wil zien van de ander. Wat heb jij nodig zodat jij beter kan werken in de avonddienst? Als je dat nou eens opschrijft. Dan zeg je niet wat hij verkeerd doet, dan zeg je wat jij nodig hebt. Dan praat je vanuit je eigen context. Veel prettiger. Je geeft dezelfde boodschap, alleen een stuk vriendelijker.

**Interviewer:**	Ik hoor dat je best wel zelfredzaam bent wat dat aangaat. Je kiest er voor om er zelf een weg in te vinden?

**Respondent:**	Ja. Klopt.

**Interviewer:**	Is dat leuk?

**Respondent:**	Soms. Kijk je hebt ook wel eens momenten. Dan loopt het over je schoenen dan is het te veel. Dan komt er te veel van boven en van beneden. Dan is het gewoon heel erg druk. Maar dan heb ik ook een bedrijfsleider die zegt 'zeg maar wat ik van je moet overnemen'.

**Interviewer:**	Is het ook voor jou duidelijk wanneer jij bepaalde dingen gedaan moeten worden?

**Respondent:**	Ja. Die schema's maak ik zelf.

**Interviewer:**	Als een soort eigen draaiboek voor jezelf?

**Respondent:**	Ja. Het schema van vorig jaar is leidend. Het is dus niet zo dat iemand die een jaargesprek heeft gehad in december dat die in maart weer aan de beurt komt. Daar houd ik wel altijd rekening mee.

**Interviewer:**	Heeft de bedrijfsleider daar nog een inspraak in wat je qua dingen moet doen?

**Respondent:**	Als soms mijn agenda te vol zit, dan neemt >BEDRIJFSLEIDER< deze over. Kijk de manier hoe de gesprekken gevoerd worden, dat doen wij ook gewoon allebei hetzelfde. Hoe wij het jaargesprek hebben ontwikkeld is allemaal in samenspraak gedaan met de bedrijfsleider. Die zal dan niet denken 'laat ik eens even lekker wild doen en zelf dingen verzinnen'. Het besluit wordt in de context van de EVR genomen. Het is niet alleen mijn smeltkroes. Het besluit wordt samen genomen.

**Interviewer:**	Heb je het idee dat je als teamleider nog over bepaalde competenties moet beschikken?

**Respondent:**	Zeker wel.

**Interviewer:**	Waarover moet je beschikken?

**Respondent:**	Ik denk dat je enorm flexibel moet zijn. Ik denk dat je enorm grote mate van sociaal EQ moet hebben. Dat mis ik ook soms nog wel eens bij collega-teamleiders zeg maar. En dat heb je voor nodig om te snappen dat wanneer iemand emotioneel is of boos of verdrietig, dat daar altijd iets onder zit en iets achter zit. Niet alleen maar een praktisch dingetje omdat het te druk is. Te makkelijk worden dingen aangenomen. En ik denk dat je goede kennis van besluitvorming moet hebben. Dus hoe de organisatie in elkaar steekt. Hoe de hazen lopen. Hoe de politieke spellen gespeeld worden. Dat gebeurt natuurlijk ook. Wat dat betreft heb ik wel veel voordeel gehad aan mijn OR werk.

**Interviewer:**	Je kent de klappen van de zweep?

**Respondent:**	Ik ben ook niet bang als mij iets dwars zit om bij de bestuurder op de deur te bonzen en naar binnen te lopen.

**Interviewer:**	Heb je het idee dat je als teamleider ook nog bepaald gedrag uit moet stralen richting het team.

**Respondent:**	Ja. Ik denk vooral vertrouwen en het vertrouwen hebben dat zij ook dingen los kunnen laten. Kijk en dat is dus het belangrijkste. Ik zie mezelf als een kapitein van het schip die moet zorgen voor de continuïteit, maar ook moet faciliteren dat de ander goed zijn werk kan doen. Dat is mijn voornaamste taak. Zo zie ik dat. En of dat nou is he om te zorgen dat de jaarplannen goed gevoerd worden en dat de jaarrekening goed is en dat de bedbezetting goed is en dat de personele afstemmen met betrekking tot de productie goed is, al die zaken. Dat gaat allemaal samen. Maar als ik dat tegen mensen op de vloer zeg 'ik ben verantwoordelijk voor dit en dit'. Daar kopen ze niets voor. Ik vind het belangrijk als ik hier loop dat ik genoeg collega's naast mij heb om de werkdruk op te vangen. En daarom wil ik ook op de werkvloer zijn. Zodat ik aanvoel van 'het is nu te druk, bedden dichtgooien' of 'een mannetje erbij zetten'. Kijk en als jij dat aanvoelt en zij voelen dat jij dat aanvoelt, dan heb je een soort goede synergie waarbij iedereen vertrouwen heeft dat het altijd wel oke is.

**Interviewer:**	Heb je het idee dat zoals je nu je functie uitvoert dat dat in lijn ligt met wat de bedrijfsleider en HR van je verwachten?

**Respondent:**	Dat hoop ik wel. Van mijn bedrijfsleider weet ik het van. Of HR dat zo vindt, dat weet ik niet. Ik denk het wel.

**Interviewer:**	Zou jij daar waarde aan hechten, wat HR van jou verwacht? Of is het de bedrijfsleider waar het allemaal om draait?

**Respondent:**	Ik weet ook wel wat HR van mij vindt. Die hebben het idee dat ik mijn functie op een goede manier vormgeef met het uitvoeren.

**Interviewer:**	Dan denk ik dat ik er wel doorheen ben.

**Respondent:**	Was het een beetje een coherent verhaal voor je?

**Interviewer:**	Ik denk dat ik een goed inzicht heb gekregen in jouw functie als teamleider en jouw visie op de functie.

**Respondent:**	Misschien is het wel interessant om ook een andere teamleider te vragen?

**Interviewer:**	Ik heb in totaal iets tien teamleiders in deze studie opgenomen, van over de hele organisatie. Zowel zorg als niet-zorg. Dat geeft een breed beeld. Er zit zeker herkenning in wat jij hebt verteld.

**Respondent:**	Maar is zit zeker verschil. Dat heeft ook te maken met hoe de bedrijfsleider in elkaar zit. De bedrijfsleider moet ook in staat zijn om dingen echt te kunnen delegeren en ook echt met het volste vertrouwen dat het dan ook goedkomt. En dat is ook nog wel eens spannend als je niet weet of je teamleider dat allemaal goed oplost. Als je er met je rug naartoe gaat staan wordt jij er op aangesproken als bedrijfsleider.

**Interviewer:**	Ik kan me voorstellen dat dat best stressvol kan zijn als teamleider als je niet competent bent?

**Respondent:**	Dan denk ik ook dat je eerlijk moet zeggen dat je daar niet competent in bent, tenzij je dat niet durft en dus weer het idee hebt, dus nooit getoetst bij de ander, dat de ander daar iets van vindt. Dat zie je ook wel eens bij verpleegkundigen. De ene zegt 'dat kan ik niet, dat heb ik nog nooit gedaan' en de ander gaat het doen en staat met een rode kop te klungelen. Dat is ook. Geef mij die eerste maar. Zo probeer ik mensen ook op te voeden. Je kunt niet alles weten. Dat is onmogelijk.

**Interviewer:**	Toe willen geven van 'ik kan dit niet'?

**Respondent:**	Dat is ook de macht van het collectief. Samen heb je een enorme bulk aan kennis. Ga er nou niet vanuit dat je alles moet weten en alles moet kunnen. Dat bestaat niet. Ik heb geen verstand van >SPECIALISATIE<. Ik heb geen verstand van >SPECIALISATIE<. Mijn collega's wel. Ik kan bepaalde dingen heel goed. Sommige dingen kan ik helemaal niet. Daar schaam ik mij ook helemaal niet voor.

**Interviewer:**	Dan vertrouw je op de expertise van collega's.

**Respondent:**	Ja. Honderd procent. Maar omgekeerd ook. Dan wordt het collectief heel sterk. Dat maakt het ook dat je een veel sterker team maakt en creëert uiteindelijk. De collectieve macht is groter dan de individuele macht.

**Interviewer:**	Heb je nog een tip voor HR hoe het leven van de teamleider beter gemaakt kan worden in de organisatie?

**Respondent:**	Ja. Ik weet niet of het een tip is voor HR of voor de organisatie ansicht. Ik weet niet of dat binnen het verantwoordelijkheidskader van HR valt.

**Interviewer:**	Wat komt er in je op.

**Respondent:**	Ik denk als je het goed wil laten draaien dan zal er veel meer afstemming moeten komen.

**Interviewer:**	Tussen?

**Respondent:**	Teamleiders. Als je wil dat de organisatie als collectie sterker wordt, dan moet je zorgen dat de individualisering weggenomen wordt. En dat zeg maar de collectieve macht ook weer terug komt. Dat mensen weer vaker met elkaar in gesprek gaan en dat ze niet denken 'daar kom die arrogante kwast weer' of 'met haar of met hem praat ik niet want zij zijn van die en die afdeling die nooit iets voor ons doen'.

**Interviewer:**	Dan krijg je een beetje een eilandjestoestand.

**Respondent:**	Juist. Vorige week zijn er drie mensen van mij het huis in gegaan om te helpen op andere afdelingen en ik krijg vier bedankmailtjes. En dan denk ik 'nou, eigenlijk zou dat helemaal niet hoeven, het zou vanzelfsprekend moeten zijn'. Dat is eigenlijk het meest sprekende voorbeeld dat ik kan bedenken. Dat is het goochem dat je hier niet voor jezelf loopt maar eigenlijk als onderdeel van een organisatie werkt.

**Interviewer:**	Zoiets als een teamleidersoverleg zou al een stap in de goede richting zijn?

**Respondent:**	Dat werkt alleen als mensen gelijk gestemd zijn. Dat is een paar keer van de grond geprobeerd te trekken, maar dan zijn er toch weer mensen die anders over de invulling denken van 'nou, dat vind ik helemaal niet zo belangrijk'. Dan komen van de 40 teamleiders die hier in huis lopen drie of vier. Tien procent. Ik schaam me kapot.

**Interviewer:**	Ja. Dat kan ik mij voorstellen. Het klinkt als een moeilijke vicieuze cirkel waar je in zit...

**Respondent:**	Weet je. Je zult het nooit als eenheid kunnen krijgen. Je kunt wel zorgen dat je het kleiner doet. Dat je gaat clusteren als het ware. De ondersteuners, de zorg, poli. Dat zijn allemaal afdelingen met andere kijk op zaken. Andere belangen. Andere manieren van werken. Andere opleidingsniveau waar je leiding aan geeft. Dat is ook nog wel een verschil. Op een poli wordt meestal, vaker, echt gewoon alleen maar gemopperd door mensen. Maar ook gewoon omdat ze vaak niet begrijpen hoe besluitvorming werkt en hoe dat gaat. Veel hebben persoonlijke begeleiding nodig. Dat is dus een andere manier van leiding geven. Heel anders dan een zorgprofessional die hoog is opgeleid, zoals een verpleegkundige met een oncologische specialisatie, dat is een ander verhaal dan de standaard verpleegkundige.

**Interviewer:**	Hoe zou een teamleider met het verschil omgaan?

**Respondent:**	Nou, dat je je van bewust bent dat er een kennisniveauverschil is en dat mensen daardoor ook positioneel anders kunnen voelen. Ik ben belangrijker. Ik heb meer kennis dan jij. Onze afdeling is belangrijker. Ik heb een >AFDELING< en een >AFDELING< afdeling. Het heeft heel veel moeite gekost om die twee afdelingen met elkaar samen te laten werken.

**Interviewer:**	Er zat een hiërarchisch verschil tussen beiden?

**Respondent:**	Wij, zij. En nu draaien ze diensten voor elkaar. Ruilen ze onderling diensten.

**Interviewer:**	Maar daar heb jij wel een aandeel in gehad als teamleider, of niet?

**Respondent:**	Ja, omdat ik geen onderscheid maak. Wie mijn hulp vraagt, daar ben ik. Dan zeg ik ook 'je moet daar even gaan helpen of desnoods een week of in de zomervakantie dat ze elkaar helpen' en dan blijkt het toch ineens gezellig en leuk te zijn.

**Interviewer:**	Maar dat zal ook over tijd zijn ontstaan, in het begin zal niet iedereen dat even leuk gevonden hebben?

**Respondent:**	Dat heeft dik een jaar gekost voordat het begon zijn vruchten af te werpen.

**Interviewer:**	Je hebt hierin een voorbeeldfunctie? Je kunt het dan wel niet praktisch doen, maar je kunt wel de mindset overdragen?

**Respondent:**	Ja. Klopt. Dat is ook 'hoe ga je met elkaar om?'. En ik zeg 'je kan roepen tegen die ander of over die andere wat je wil, maar aan het einde van het liedje heb je elkaar gewoon nodig'. Dus als je nu drie keer nee roept, hoef je een vierde keer niet iets te gaan vragen. Of je kunt gewoon met elkaar om tafel zitten. Ga eens met elkaar koffie drinken en ga eens eventjes bij elkaar kijken ga elkaar eens helpen. En je zult zien hoe fijn het is. Want op het moment dat jij het druk hebt, komt diegene jou ook helpen.

**Interviewer:**	Dat heb je allemaal voor elkaar gekregen omdat je het respect hebt verdiend van je medewerkers?

**Respondent:**	Ja. Maar ook door het te versimpelen en door het terug te brengen naar de meest basale en normale menselijke dingen en het helemaal niet te verheffen over functie of status of weet ik wat.

**Interviewer:**	Laagdrempelig maken?

**Respondent:**	Ja. Het interesseert mij niet of ik de directeur voor mij heb, of een dokter, of een verpleegkundige. Ik ga met iedereen gelijk op. En al ik boos ben. Dan ben ik net zo boos op de dokter als op de verpleegkundige. Geen onderscheid.

**Interviewer:**	Ik weet niet of er nog bepaalde dingen zijn die je toe zou willen voegen aan dit gesprek.

**Respondent:**	Nee, ik denk het niet. Ik denk dat we aardig. Dat je aardig gevoed bent voor je stuk.

**Interviewer:**	Dat denk ik ook wel ja. Is het leuk om zo'n personeelsfunctie te hebben? Het houden van functioneringsgesprekken of maken van ...

**Respondent:**	Ja dat vind ik wel interessant. Met een jaargesprek is het toch alleen een stuk je heen en terug kijken. Van 'waar kom je vandaan, waar sta je nu, en waar wil je naartoe?' dat een beetje. Het is altijd wel leuk om te horen omdat iedereen in een andere levensfase zit. Kijk de jongelingen hebben een ander perspectief van verleden, heden, en toekomst dan iemand die aan het einde van zijn carrière zit en die gaat er ook anders mee om. En die heeft ook andere vragen. En dat vraagt ook weer wat anders van mij. En dat maakt het natuurlijk interessant. Ik ben altijd wel gek geweest op dat soort dingen een beetje te helpen. Dat we de puzzel zo kunnen maken dat iedereen er weer gelukkig van wordt. En kijk, het lukt niet altijd. Je hebt ook wel eens mensen die teleurgesteld zijn. Ik heb ook mijn beperkingen. Daarin ben ik eerlijk. Ik zeg 'ik ga mijn best doen, maar dat zijn de buitenlijnen, daar tussenin kunnen we kijken wat we kunnen doen'.

**Interviewer:**	Juist. Hoe zijn die buitenlijnen voor jou duidelijk geworden over tijd?

**Respondent:**	Dat is afhankelijk van het onderwerp denk ik. Kijk arbeidsrechtelijk heb ik veel aan mijn tijd bij de OR gehad en weet ik ook vrij veel over arbeidsrecht en waar mensen recht op hebben. En voor de rest is het heel praktisch nadenken en snel in je hoofd dingen kunnen rekenen. Met name als het gaat over de ouder-wordende medewerker die toch zijn jaren moet volmaken en het wordt steeds zwaarder, dan gaan we heel nadrukkelijk kijken van 'hoe kunnen we het rooster zo maken dat jij ook fysiek en mentaal aan het werk kunt blijven, zonder dat jij helemaal stuk draait en er ook nog lol in blijft houden?'. Dan gaan we kijken hoe we die PLB-uren maximaal kunnen verdelen over het jaar waardoor je werklast en de herstelperiode van die persoon beter wordt.

**Interviewer:**	Maak je daarvoor nog veel gebruik van kennis die HR heeft? Dit klink namelijk best als een ARBO onderwerp.

**Respondent:**	Ja. Maar meer als ik dingen moet doorrekenen. Als ze zeggen dat ze er onvoldoende aan hebben en ze zeggen van 'ik ben inkomensafhankelijk'. Dan ga ik wel even praten van 'ik wil dit en dit doorgerekend hebben'. Iemand even een paar uur minder werken, maar hij heeft dit salaris nodig. Als we dat nou zo met die PLB-uren doen en daar een uurtje eraf, komen we daar netto op uit qua werken en dat is net behapbaar. Maar wat houdt deze persoon dan netto over? Daar maak ik dan wel eens gebruik van. Dat salaris vind ik helemaal. Daar ben ik niet voor opgeleid ook.

**Interviewer:**	Dan maak je gebruik van de kennis van anderen?

**Respondent:**	Ja. Dan maak ik een sjabloontje, een blauwdruk van 'nou zo zie ik het, maar wat betekent dat voor die persoon in zijn portemonnee?'.

**Interviewer:**	Dat is een heel specifiek stukje kennis. Maar over het algemeen kun je jezelf wel uit de voeten maken met alles waar het personeel mee te kampen heeft?

**Respondent:**	Ja. Dat is het voordeel als je al dertig jaar meehuppeld in de zorg.

**Interviewer:**	Hartstikke bedankt.

*Einde transcript*

# Transcript interview FLM 5

**Duur interview:	54 minuten en 36 seconden**

**Functie:	Teamleider**

**Afdeling(en) onder supervisie:	support**

**Gender:	man**

**Werkervaring in functie:	0.8**

**Respondent:**	Zal ik gewoon beginnen met iets over mezelf te vertellen?

**Interviewer:**	Ja, goed.

**Respondent:**	Ik ben vanaf half november werk ik hier in het ziekenhuis. Daarvoor. Ik kom eigenlijk uit de sociale zekerheid dus ik heb jarenlang gewerkt voor >SOCIALE INSTANTIES< en voor een zorginstelling. Dus ik ben eigenlijk niet zo thuis in het ziekenhuiswereldje. Dat begin ik nu een beetje te leren. Dus dat verschil kan ik wel goed uitleggen.

**Interviewer:**	Top. Dan is mijn eerste om mij eens mee te nemen in jouw dagelijkse werkzaamheden als teamleider.

**Respondent:**	Zal ik een dag beschrijven?

**Interviewer:**	Ja. Wat doe je zowaar?

**Respondent:**	Wat doe ik allemaal. Ik begin om negen uur in de ochtend. Dan ga ik naar de kamer toe. Doe mijn jas uit en ga naar de kamer toe een kop koffie drinken met de dames en heren binnen. En dat doe ik heel bewust om te zorgen dat ze mij altijd zien. In ieder geval elke dag even de mogelijkheid hebben om de dag door te spreken of de bijzonderheden neer te leggen. Het is een team dat heel veel leidinggevende in de afgelopen periode heeft gehad. Niet allemaal even positieve ervaringen met de leidinggevenden gehad hebben en een behoefte aandacht hebben. Dus dat dus. Dat doe ik nu elke dag. Elke dag ga ik bij hun zitten en dan ga ik even een kopje koffie met ze drinken en dan houd je feeling met je groep. Wat ik dan ga doen, dat is meestal een kwartiertje half uurtje als er niets is ben ik zo weg en anders blijf ik praten, wat ik dan ga doen is roosters, een beetje mail beantwoorden, en dan gaan de afspraken lopen die ik heb gemaakt. Je hebt een MT vergadering. Je hebt een debriefingvergadering. Er worden afspraken met fabrikanten gemaakt voor de beddenmovers. En dan gaat je dag lopen en geen dag is eigenlijk hetzelfde. En om twee uur is het alweer klaar en ga ik naar huis. Het is een vrij korte dag voor mij. Het zijn maar vijf uurtjes. Ik werk parttime. Dus ik probeer mijn tijd zo efficiënt mogelijk in te delen. Hoewel ik wel moet zeggen dat dit nu een van de rustigere banen is die ik heb gehad. Dus ik ben wel op zoek naar uitbreiding van mijn taken. Dat heb ik dan nu ook inmiddels met >HOOFD< geregeld. dus dat ik er wat extra projectjes bij krijg.

**Interviewer:**	Wat voor projecten zou je er dan bijvoorbeeld bij krijgen?

**Respondent:**	Gastvrij met sterk. Ik weet niet of je daar ooit van gehoord hebt, maar dat is een soort keurmerk waarmee je beoordeelt wordt als ziekenhuis hoe gastvrij je bent voor je gasten, patiënten, bezoekers. En dat keurmerk moet dit jaar weer opnieuw uitgedeeld worden. We moeten opnieuw geaccrediteerd worden zoals dat heet. En daar moeten een aantal dingen voor gedaan worden zoals beleid moet ervoor gemaakt worden, er moet geïnventariseerd worden, enzovoorts. Dus dat.

**Interviewer:**	Ik hoorde net als een stukje HR-taken die je benoemt. Zoals de roosters.

**Respondent:**	Ja. Klopt.

**Interviewer:**	Zijn er nog meer dingen die jij doet op het gebied van personeelsmanagement?

**Respondent:**	Ja. HR-taken. De gesprekken met de medewerker, die voer ik altijd. Ik probeer heel laagdrempelig te zijn. Dus ze mogen met alles wat hun bezig houdt, dus niet alleen in het werk maar ook juist in het privé en waar je tegen aan loopt in je ontwikkeling, probeer ik ook zo open mogelijk tegen ze te zijn dat ze dingen aan mij gaan vertellen. Dus ik probeer tegenwoordig, met een aantal is dat inmiddels gelukt en met een aantal nog niet, een vertrouwensband met die mensen te kweken dat ze mij als een praatpaal gebruiken omdat dat vaak oplucht. Dus op die manier probeer ik invloed op mensen uit te oefenen, op het gedrag dat ze vertonen. Dus daar zit wel iets. Dus dat. De roosters dan. Dit is wel een dingetje hier. Ik weet niet of je het meegekregen hebt maar ik heb 29 mensen, 14 vrijwilligers en 15 mensen met kortdurende dienstverbandjes. En dat moet allemaal in een rooster onder elkaar gepland worden en dat is wel een gedoetje. Want als de ene vrij is moet ik de ander inroosteren en nou ja, iedereen is gewend aan zijn vaste tijden en dat moet allemaal wat flexibeler worden. En daarmee ben ik ze afgelopen maanden steeds flexibeler aan het maken.

**Interviewer:**	Hoe pak je dat aan?

**Respondent:**	Door het gewoon te doen. Het voorstel van een rooster leg ik neer bij mensen en daar kunnen ze op reageren en kunnen ze precies zien in het rooster welke diensten ze de maand erop gaan draaien. Zijn ze het er niet mee eens, dan komen ze het vertellen. Maar dat gaat niet van de ene op de andere dag. Veranderingen gaan altijd heel langzaam. De eerste maand heb ik niets veranderd. De tweede maand een beetje. De derde maand iets meer verandert. En ik ben nu bezig met de bezetting aan het terugschroeven. Ik vond dat er te veel mensen te weinig deden voor het werk dat er te doen was. Dus wat ik nu aan het doen ben ik vrijwilligers bijvoorbeeld uitroosteren waardoor de groep die patiënten vervoert kleiner wordt en dat ze het ook wat drukker gaan hebben. En dan krijg je met het groepsproces te maken. Want onderling is er veel haat en nijd geweest de afgelopen periode en dat komt ook omdat ze tijd over hebben om op elkaar te letten. Als de druk wat toeneemt, worden ze wat meer gedwongen om met elkaar samen te werken.

**Interviewer:**	Dat houdt ze uiteindelijk bezig?

**Respondent:**	Juist.

**Interviewer:**	Ik heb eens opgezocht wat voor HR-taken bij een zo'n teamleider zouden kunnen liggen en daar komt dan deze taken rollen er uit. Zou eens kunnen kijken welke taken jou bezighouden en op wat voor manier jij dat dan doet?

**Respondent:**	Ja. Werving en selectie. Ben ik wel mee bezig, maar we zitten hier in een afbouwende sfeer. Dus werving en selectie doe ik wel, maar dan met stagiaire en zo van 'kom binnen, wie ben je, waarom?'. Maar werving en selectie zelf, dat hoort bij mijn taak, vind ik wel, maar doe ik op dit moment niet.

**Interviewer:**	Wat bedoel je met de afbouwende sfeer?

**Respondent:**	Ik heb te veel mensen zitten in totaal. Ik heb 29 mensen en te veel vrijwilligers dus die ben ik aan het uitfaseren zoals dat heet. Dat betekent dat ik ze stimuleer naar andere werkzaamheden. Of hun aanspreek op hun gedrag of hun vraag om een ander dagdeel te gaan draaien. Voor vaste medewerkers, dat is een langduriger traject. Als je die gaat aanspreken op houding en gedrag, moet je ze ook die kans geven zich te ontwikkelen en zich te verbeteren. En voordat je uitkomt in het disfunctioneringstraject ben je eigenlijk alweer een jaar verder. Dat gaat niet heel snel. Wat wel sneller gaat is mensen motiveren om buiten hun werk te gaan kijken van 'goh, misschien zijn er andere mogelijkheden binnen het ziekenhuis en vind je dat leuk om te gaan doen en wat heb je daar dan voor nodig?'. Dat voer ik ook wel. Dat zijn de POP-gesprekken.

**Interviewer:**	Nu hebben we het over een personeelsoverschot. Mocht het dus zo zijn dat er straks weer wat vacant komt te staan, ben jij dan de persoon...

**Respondent:**	Ik weet niet hoe dat precies geregeld is in het ziekenhuis. Maar ik vind wel dat ik daar grootste verantwoordelijkheid in heb en die neem ik dan ook. Het zal ongetwijfeld in samenwerking met HR gaan. Met >HR ADVISEUR< denk ik. Want dat vind ik wel belangrijk. Want die hebben toch een iets andere kijk op de mens. Maar ik ben verantwoordelijk voor hoe mijn team functioneert en ben verantwoordelijk voor de individuele teamleden en ga dat wel kijken van 'wie passen er in mijn team en wat mis ik nog in mijn team?'. Dus daar ga ik wel heel bewust mee om.

**Interviewer:**	Vind je dat je een adviserende stem hebt? Of vind je dat je een hard, ja, nee, stem hebt?

**Respondent:**	Ik vind, ik eis, een beslissende stem. En dat klinkt misschien heel hard. Maar dit is mijn team. Ik ben er verantwoordelijk voor en ben er ook eindverantwoordelijk voor. Dus volgens mij ben ik dan degene die de beslissing neemt.

**Interviewer:**	Zijn er nog meer punten van het lijstje die jou opvallen?

**Respondent:**	Introductie. Natuurlijk. Dat speelt bij nieuwe medewerkers. Ik heb hier een werksetje. Wat ik dan wel doe, dat heb ik dan in het verleden gedaan, een praatje. Ik doe de werving en selectie. Daardoor leer je mensen al beter kennen en doe ik een introductiepraatje welke eisen ik stel aan houding en gedrag enzovoort. En het echt daadwerkelijke inwerken laat ik aan de medewerkers over omdat die gewoon want, ja, gewoon, die zitten op de werkvloer en weten wat ze moeten doen dus he. Binnen het ziekenhuis heb je ook introductieweken of een introductiedag. Dus dan leren ze het ziekenhuis kennen, leren de werkzaamheden kennen, en weten wat ik van heb verwacht. Volgens mij heb je dan het plaatje rond. Dus dat. Bijhouden van personeelsbestanden. Dat is onderdeel van het werk. Als iemand verhuist geef je een mutatie door.

**Interviewer:**	Dat komt allemaal bij jou terecht?

**Respondent:**	Ja. Ze geven aan mij door van 'ik ga verhuizen' en dan vaak is het even een mailtje naar HR sturen of even langswandelen en dat zijn dan de mutaties. Dat is niet heel interessant. Tenminste, dat zijn de administratieve taken. Die horen erbij. Beslissen over en plannen van ontwikkelings en trainingsbehoeften medewerkers. Dat is dat POP-traject waar ik het net over had. Beslissen lig iets anders. We hebben een bepaald opleidingsbudget voor mijn team. En binnen dat opleidingsbudget kan ik samen met HR de route bepalen. Als ik buiten het budget ga heb ik eigenlijk geen beslissingsbevoegdheid en dan ga ik naar >HOOFD< of iemand anders. Dan is het maar afhankelijk van hoe belangrijk >HOOFD< het vindt, of zij over genoeg geld beschikken, of dat soort dingen.

**Interviewer:**	Maar eigenlijk, alles wat binnen het budget valt dan kan jij op je eigen houtje...

**Respondent:**	Dan kan ik doen. Maar dat is niet iets wat ik wil omdat HR met name zicht heeft wie binnen het ziekenhuis dat soort trainingen bijvoorbeeld zou kunnen geven en of er nog andere wegen zijn en vaak zijn er ook contracten afgesloten met opleiders. Dus dat doe ik altijd wel in samenwerking met HR. Ik ben wel degene die zegt 'dit is wel het soort training dat ik wil' en daar kun je in meegaan en laat ik mij graag over adviseren over de mogelijkheden. En er dan ook wel eens is gebeurt is 'misschien is het wel handig als?' of 'heb je daar wel eens over nagedacht?'. Zo maak je het plaatje compleet. Dat is zeg maar de samenwerking. Even kijken hoor. Beslissen over en plannen van trainings en ontwikkelingsbehoeften. Eigenlijk kan dat natuurlijk niet he? Eigenlijk is dat een gekke zin. Ik kan niet beslissen over iemand zijn behoeften want die hebben ze.

**Interviewer:**	Nee. Precies. Maar jij kan wel een training of iets dergelijks aanbieden van 'ga op cursus' of 'ga naar dat symposium'.

**Respondent:**	Ja. Zeker. Geven van formele training. Wat bedoel je daarmee?

**Interviewer:**	Zoiets als dat je voor de groep gaat staan om een uitleg te geven hoe dit werkt of hoe dat werkt.

**Respondent:**	Dat gebeurt natuurlijk in een soort werkoverleg wel. Ik heb daar wel over na zitten denken of ik dat zou gaan doen. In mijn andere werk heb ik dat wel gedaan. Ik ben >FUNCTIE< en kan mensen wel echt heel veel vertellen over houding en gedrag. Maar dat ik vertellen, maar het heeft veel mee impact als iemand anders dat doet omdat ik natuurlijk ook beslis over de dagelijkse gang van zaken. Ze zien me elke dag. En ik weet als een training impact moet hebben dan kun je beter iemand anders erbij halen die buiten de groep staat. Die kan ook makkelijker signaleren wat er aan de hand is en vanuit een specialisatie de training geven. Ik zou het wel kunnen, maar ik doe het in principe niet binnen mijn eigen groep.

**Interviewer:**	De manier van een externe partij is naar jou mening effectiever?

**Respondent:**	Is effectiever. Ja. Ja. Vreemde ogen dwingen.

**Interviewer:**	En formele training dan? Als je bijvoorbeeld iemand ziet duwen of tillen dat je zegt van 'goh, kun je dat niet misschien zo of zo doen?'.

**Respondent:**	Ja dat doe ik wel, maar dat is echt aanspreken op houding en gedrag. Je noemt het informele training maar eigenlijk is het gewoon aanspreken van 'ik zie dat je dit doet, kun je dat niet op een andere manier doen?'. Coachen gaat verder dan dat vind ik. Coachen gaat ook over hoe iemand privé in zijn leven zit. Ik heb hier regelmatig gesprekken over privé. Ik kan je wel een voorbeeld geven. Ik heb iemand zitten die ontzettend in de schulden zit op dit moment. Dit help, op afstand, die help ik van 'heb je daar al aan gedacht, heb je daar al aan gedacht, ben je al naar de gemeente geweest om de WSNP aan te vragen?'. Dat soort dingen. Maar dat komt omdat ik al heel veel ervaring heb met dat soort gebieden van de sociale zekerheid uitkeringen en alles dat daarbij hoort.

**Interviewer:**	Vind je dat dit iets is dat een teamleider standaard zou moeten doen, of is dit iets dat toevallig in jou persoonlijkheid ligt?

**Respondent:**	Beide denk ik. Maar ik ben van de coachende leiderschapsstijlen. Ik vind het belangrijk dat ik weet wat mijn mensen bezighoud. Als ik weet waar mijn mensen mee bezig zijn, kan ik ze ook beter leiding geven en kan ze beter sturen en kan ik ze ook beter helpen hun werk doen. Op het moment dat jij thuis ontzettend in de problemen zit en je belt op van 'ik wil een dag vrij hebben'. Dan kan ik dat snappen als ik weet wat er aan de hand is en kan ik je heel makkelijk vrij geven. Dat is maar een heel simpel voorbeeld, maar zo werkt het natuurlijk wel. Als ik niet weet wat er bij je aan de hand is en ze bellen me van 'kan ik vrij nemen?'. Dan kan ik denken van 'je hebt het niet nodig en ik heb je hier op het werk nodig' en ik weiger. Terwijl het voor die persoon misschien wel heel belangrijk is. Vervolgens komen ze hier gefrustreerd naartoe en ben je nog verder van huis dus. En dat gaat natuurlijk veel verder dan privé. Dat gaat ook om zieke kinderen thuis, mantelzorg, maar ook je eigen behoeften aan ontwikkeling. Waar wil je naartoe en wie ben je? Vind ook belangrijk ander kan ik het gewoon niet goed doen vind ik.

**Interviewer:**	Duidelijk.

**Respondent:**	Mentoren. Wat bedoel je daarmee?

**Interviewer:**	Dat komt dan neer een op soort coachende rol die jij actief invult? Pak je daar dan als eerst moment die POP-gesprekken voor als eerst moment om privé-dingen te signalen.

**Respondent:**	Nee. Dat zeg ik. Dat gebeurt gewoon door het binnenlopen. Maar ook gewoon door de gesprekken in de koffiekamer. Dingen ook bespreekbaar maken. Ik heb elke dag een andere groep mensen zitten. Laat ik het maar even zo zeggen. Als er de dag daarvoor iets is gebeurt in de groep, dan ben ik degene die het de volgende dag tegen de andere groep vertelt 'nou uh houd rekening , Nathalie is ziek want' of 'dit is er aan de hand dus ze neemt vrij'. Weet je en ik ben wel een soort intermediair. Hoe noem je dat. De missing link tussen de groepen. Daarbij informeer ik actie de groepen over de andere mensen. En dat betekent dat ik overal wel van op de hoogte moet zijn en dus ook op de hoogte raak van wat er speelt. Daarvoor zijn die koffiegesprekjes en daar begint het eerste signaleren en als ik zie dat iemand niet lekker in zijn vel zit, dan roep ik ze apart en ga ik apart met ze praten.

**Interviewer:**	Ik kan me namelijk ook voorstellen, als je ergens mee zit, iets met collega's, dat je niet lekker in zo'n koffiemoment bespreekbaar maakt.

**Respondent:**	Ja. Of juist wel he? Dat ik dat juist wel in de groep gooi. Het is ook wel eens gebeurt dat ze tegenover elkaar stonden dat er een incident is aan de hand is geweest in de ochtend en dat vind ik het bij uitstek wel een moment om in de groep te bespreken omdat ik vind dat de groep ook een verantwoordelijkheid heeft in hoe men met elkaar om gaat. En ik kan dan wel de twee spelers bij elkaar roepen, maar eigenlijk vind ik dat de groep zichzelf moet corrigeren en dat vertel ik dan ook. Als twee mensen onderling ruzie hebben, dan kan ik wel een scheidsrechter gaan zijn, maar ik vind eigenlijk belangrijker dat ze het probleem gaan oplossen. Daar hebben ze mij in feite niet voor nodig, het zijn allemaal volwassen mensen kom zeg. Toch?

**Interviewer:**	Het kan diep gaan?

**Respondent:**	Dat is ook zo. Het blijven natuurlijk ook wel mensen. Dan zie ik functioneringsgesprekken en beoordelingsgesprekken. Dat hoort erbij. Dat zijn de jaargesprekken hier. Ik ben nu bezig, toevallig net met >HOOFD< afgesproken dat ik, ik ben 100 dagen, net iets meer dan 100 dagen in dienst. En ik noem het 100-dagen-gesprekken. Eén voor een ga ik ze allemaal binnen halen en ga ik vertellen wat ik van ze vind en mogen ze vertellen wat ze van mij vinden. Dat zijn dan eigenlijk de functioneringsgesprekken. Dus dat. Ik vind dat een beoordelingsgesprek nooit een verassing mag zijn. Want ik vind dat je van mij regelmatig moet horen hoe je functioneert.

**Interviewer:**	Pak je dan ook dat format erbij van het functioneringsgesprek?

**Respondent:**	Nee. Als het. Ik weet nog niet of ik het ga vastleggen. Dat weet ik nog helemaal niet. Op het moment dat er dingen fout gaan dan zijn er wel waarschuwingen geweest in de groep. Die leg ik natuurlijk wel vast. Die leg ik ook vast in het personeelsdossier. Maar dit soort gesprekken weet ik niet. Het zijn niet echt functioneringsgesprekken. Het zijn ook geen beoordelingsgesprekken. En het zijn ook een POP-gesprekken.

**Interviewer:**	Het zijn contactmomenten?

**Respondent:**	Ja. Het zijn van die contactmomenten dat je elkaar eens even goed de waarheid kunt vertellen. En dat hoeft niet altijd op papier.

**Interviewer:**	Als het maar gezegd is uiteindelijk?

**Respondent:**	Als ze maar weten waar ze aan toe zijn. En ik ook natuurlijk. Het gaat twee kanten op. Akkoord geven op ontwikkelingsplannen. Je ontwikkelt een visie als leidinggevende en ja, volgens mij kan ik dat gewoon uitvoeren, maar gaat dat in samenspraak met mijn leidinggevende. Want het moet ook in zijn visie passen. En uiteindelijk moet het weer in de visie van het ziekenhuis passen. Dus ja. Je hebt een richting waarop je wil met je team en dat heb ik nu, voor dit team heb ik dit nu redelijk in beeld 'he, dit is de richting waarop ze gaan'. Ook >HOOFD< heeft dat beeld. Dat hebben we gedeeld met elkaar. En die richting gaan we ook op. En gaandeweg wordt dat steeds verder bijgesteld. Het is niet de visie die op papier staat. Dan heb je disciplineren en corrigeren. Ja. Op het moment dat ze dingen niet mogen, dan krijgen ze een waarschuwing. Officieel gesprek, waarschuwing. Of een officieel gesprek zonder waarschuwing, dat kan natuurlijk ook.

**Interviewer:**	Voer je dat gesprek dan alleen of nodig je een derde uit?

**Respondent:**	Soms voer ik het wel alleen. Het liefste voer ik het alleen. Maar soms is het beter om het met z'n tweeën te doen om het meer gewicht te geven. En dat hebben we bijvoorbeeld wel gedaan. Toen ben ik bij >HOOFD< samen met >HOOFD< hebben we een gesprek gevoerd met één van mijn medewerkers. En dat zit een beetje een psychologisch dingetje achter he, om dat nog meer gewicht te geven. Zo van 'weet dat dit gewoon echt niet geaccepteerd wordt'. En mijn ervaring is vaak, ik kan wel zeggen dat jij dingen mogen. Dan denk je 'pff, ik ga wel naar >HOOFD< toe, die denkt daar toch anders over'. Als je dat met z'n tweeën doet, is die weg afgesloten. Dus dat is een beetje een psychologisch dingetje. Soms haal ik HR erbij.

**Interviewer:**	Maakt het nog uit in wat voor gevallen je voor HR kiest in plaats van >HOOFD<?

**Respondent:**	We hebben een dingetje met seksuele intimidatie. En dan loop ik even naar HR toe van 'ik heb dit nu en ga me adviseren hoe ik hier nu mee om moet gaan'. En goed. Dan hebben we daar op de achtergrond contact mee. Maar ik voel wel de gesprekken. HR is dan beschikbaar om er eventueel bij te komen zitten. Dat is prima als je vindt dat dat nodig is kan dat. Maar tot nu toe heb ik zoiets van 'ik kan het vaak wel alleen af'.

**Interviewer:**	Als je dan bijvoorbeeld voor die seksuele intimidatie contact je HR. Contact je dan HR omdat zo'n onderwerp is dat je even niet wat je moet doen, dat het zo afwijkend is. Als mensen ruzie hebben, dan zou je daar een manier op kunnen bedenken. Maar dit zijn extreme gevallen. Hoe moet ik dit nu aanvliegen.

**Respondent:**	Ja. Deels. Ik vind ook dat HR redelijk op de hoogte moet zijn van hoe het met het personeel gaat. Dus ook met waarschuwingen en dat soort dingen, ook als het goed gaat moeten ze het weten, maar dat doe ik dan tijdens de bilaterale gesprekken met HR. Een keer in de drie vier weken hebben we zo'n gesprek en dan praten we daarover. Maar zeker met dit soort dingen omdat dit ook de integriteit van het ziekenhuis raakt, en als ik het niet signaleer en niet meld aan HR, kunnen zij nooit een compleet plaatje krijgen hoe het gaat in het ziekenhuis. En vaak hebben ze toch ervaringen met seksuele intimidatie. Dat heb ik wel een keertje gehad bij het UWV. Maar hier zijn toch weer andere gedragsregels en andere protocollen. Dat moet je weten en HR weet dat als het goed is. Dus daarom. Ik gebruik ze als informatiebron en als signaalfunctie zeg maar. Afhandelen van tuchtprocedures en klachtprocedures. Klachtprocedures ligt bij mij. Maar dan gaat het vaak over klachten over het gedrag en houding van de medewerkers. Tuchtprocedures dat licht niet bij mij. Absentiemanagement. Ja ik geef toestemming tot verlof of niet.

**Interviewer:**	Als er iets van ziekmeldingen binnenkomt, geef jij daar wel of geen akkoord op?

**Respondent:**	Ik heb met de dames en heren afgesproken dat zij eerst mij bellen. En als het goed is, is het goed dan vind ik het akkoord. Maar ik wil eerst weten wat er aan de hand is. Voor de rest mogen ze dan de secretaresse bellen of ik meld ze dan door. Maar eerst naar mij toe. Het gebeurt niet vaak dat ik weiger is het zo dat ze van 'ik voel me niet lekker, ik heb gisteravond een feestje gehad en voel me niet lekker, ik noem maar even een voorbeeld hoor, ik voel me niet lekker en ik wil mij ziekmelden'. Die meld ik niet ziek, dat vind ik geen ziekmelding. Dan zeg ik van 'weet je wat, neem een aspirientje en duik even je bed in, maar ik wil je om twaalf uur weer terug hebben'. Weet je in de avond een kerel in de ochtend ook een kerel. Dus ik zit daar wel heel strak op. Ik vind ook als je ziek bent ben je ziek, daar is niets aan te doen. Maar vaak zijn het dat grijze gebied met name dat mensen zich makkelijk ziekmelden. Daar ben ik heel vel op. Dat wil ik niet. Ik heb dan liever dat je belt en zegt 'mag ik een dag vrij krijgen?', dan dat je je ziek meld.

**Interviewer:**	En als we het hebben over waardering geven, het volgende item, op wat voor manier doe je dat.

**Respondent:**	Dat is vaak individueel of als groep. Ik weet niet of je dat weet maar ik doe elke vrijdag een vrijdagmail zoals dat heet. Dat is een oude traditie die doe ik nou al twintig jaar inmiddels en daarin vertel ik wat er die week is gebeurt en wat er goed, maar ook wat er fout gaat, wat de plannen zijn. Dat. Daarin geef ik waardering. Als dingen goed zijn gegaan 'harstikke goed'. Dat doe ik in de koffiekamer. Als ik gedrag zie dat ik goed vind, duimpje omhoog, schouderklopje. Dat soort dingetjes. Maar verder dan dat gaat het eigenlijk niet. Het is meer hoe je met de persoon omgaat. Doen ze dingen goed, harstikke goed, doen ze dingen verkeerd dan niet. Beslissen over salarisschalen. Daar ga ik niet over dat is CAO. Mensen worden ingeschaald en klaar. Dat doet HR ook. Die gesprekken worden daar gedaan. Bottom-up communicatie. Alles wat ik signaleer en hoor op de vloer en dat heeft belang voor het hele ziekenhuis, dan gaat het via mij naar >HOOFD< of naar degene die dat betreft. Top-down communicatie eigenlijk ook. Dat loopt dan andersom he?

**Interviewer:**	Ja. Het klinkt misschien negatief, maar een soort doorgeefluik?

**Respondent:**	Ja. Een doorgeefluik. Dat is een heel belangrijk aspect want daar zet je de sfeer in je team neer. Dus op het moment dat ik mensen het idee geef dat ze gehoord worden, dan zullen ze dat ook voelen. Op het moment dat ik informatie achterhoud of niet deel, dan hebben ze dat op een gegeven moment wel door. Die gasten lopen het hele ziekenhuis door. Horen ze het niet van mij, dan horen ze het wel van iemand anders. Het cruciaal voor mijn positie om het vertrouwen van de mensen te hebben en daar kun je de communicatie natuurlijk harstikke goed voor gebruiken. Als ze problemen hebben signaleren ze dat en als ik er dan niets mee doe gaan ze langs me heen naar iemand anders toe en dan ben je het vertrouwen van de mensen kwijt. Dus dat is wel een hele cruciale schakel.

**Interviewer:**	Maar jij zou misschien ook wel eens nieuws moeten delen wat jij helemaal niet leuk vindt om te delen of iets waar je het niet mee eens bent?

**Respondent:**	Dat ligt eraan. Wat ik wel probeer is dat voordat beslissingen genomen worden, probeer ik daar al bij betrokken te zijn en mijn mensen daarbij te betrekken als het hun gaat raken. Dus ik vertel ze van te voren 'dit zijn de beslissingen, dit zijn de argumenten voor, tegen, en dit is een beslissing die genomen moet worden'. Als ze daar eenmaal invloed op uit hebben kunnen oefenen, dan als uiteindelijk de beslissing genomen wordt zijn er argumenten voor en zijn er argumenten tegen. Maar op het moment dat je invloed kunt uitoefenen op een beslissing zal je die later ook wat makkelijker accepteren. Snap je wat ik bedoel? Dus dat probeer ik. Dat is natuurlijk niet altijd zo. Dat ik het personeelsbestand moet afbouwen zijn ze het natuurlijk helemaal niet mee eens en dat begrijp ik ook wel. Dat gaat direct ten koste van hun werk. Aan de andere kant begrijpen ze ook wel dat er een ziekenhuisbelang is die misschien wel voor gaat. Aanhoren en reageren op personeel. Dat is eigenlijk hetzelfde verhaal. Coördineren van het werk van teams. Ik heb maar een team dus.

**Interviewer:**	Misschien dat je aanstuurt wat de ploegen moeten doen, of richten ze dit juist zelf in?

**Respondent:**	Er valt weinig te sturen. Ze worden opgeroepen door een telefoontje van de afdeling en er moet een patiënt van a naar b vervoerd worden en dus er valt heel weinig aan te coördineren. Wat ik nu wel aan het doen ben is om te kijken of ze naast dit werk ook nog ander werk kunnen doen en dat is bijvoorbeeld kledinguitgifte. Dat zijn ze dan nu ook aan het doen. Maar ja ik vind dat niet echt coördineren. Dat gaat overigens wel veranderen want er zijn serieuze plannen om dit team innig te laten samenwerken met een facilitair team. Dat is een beetje een visieplan en daarmee komt er dan ook een stukje coördinatie bij.

**Interviewer:**	Deze mensen worden dan breder inzetbaar?

**Respondent:**	Worden breder inzetbaar, er komen ook want andere mensen bij. Dan zou het zomaar kunnen zijn dat je op een gegeven moment heel druk bent met >AFDELING< en dat je meerdere mensen op >AFDELING< inzet en als er een vrachtwagen gelost moet worden dan zet je daar wat meer mensen op in. Een ook als het aankomt op coördineren dat wordt dan belangrijker. Maar dan heb je ook weer allemaal software voor je die dat regelt. Maar dan wordt dat belangrijker. Maar nu worden ze aangestuurd door het aanbod.

**Interviewer:**	Dat red zichzelf?

**Respondent:**	Dat red zichzelf. Onderhouden van effectief teamwerk. Dat is wel een dingetje. Dat heeft alles ook weer te maken met roosters, hoe je mensen neerzet, welke mensen je bij elkaar zet. En soms is het beter om minder mensen op wat meer weer werk in te zetten dan veel mensen op weinig werk, want dan slaan ze elkaar de hersenen in. Dat is wat ik je net aan het begin verteld. Dus dat moet je wel een beetje slim doen. Maar dat is het roosteren. Personeel consulteren en raadplegen. Elke morgen en anders haal ik ze er gewoon bij. Als ik een fabrikant hier heb dan haal ik ze erbij. Als ik interviews heb, nou ja vandaag toevallig dan niet, maar normaal gesproken dan wel. Vind ik overigens ook een onderwerp dat niet met hen besproken hoeft te worden. Gezondheid en veiligheid. Arbo.

**Interviewer:**	Dit zijn toch mensen die een hoop kilometers maken op een dag. Hoe houd je ze vit en vitaal?

**Respondent:**	Dat is wel een dingetje. Daar zijn ze eigenlijk nooit mee bezig geweest. Ze hebben wel bijvoorbeeld een duw en trekcursus ooit gehad en vanuit HR wordt er af en wel eens iets geadviseerd over de vitaliteitsweken en dat soort dingen. We hebben toevallig afgelopen dinsdag van >ARBO< een instructie, een presentatie, gehad over duurzame inzetbaarheid en hoe je ziekteverzuim kunt aanpakken. Dat het niet alleen ziek zijn is maar dat het ook met houding en gedrag te maken heeft en leefstijlen. Dat begint nu in het ziekenhuis te komen. Zelf ben ik daar al wel verder mee. Op deze manier doe ik het nu al jaren lang. Juist omdat ik die connectie met de mensen wil hebben en wil weten wat er aan de hand is met ze. Maar in het ziekenhuis is dat wel nu en voor hun is het ook nieuw dat ik op die manier met ze om ga.

**Interviewer:**	Maar je houdt gesprekken met ze, om het dan in kaart te brengen als er wat aan de hand is...

**Respondent:**	Ik kan je wel een voorbeeldje geven. We hebben hier iemand zitten die is volgens mij 80. Ietsjes jonger volgens mij. Vrijwilliger is hij. Maar die kan bedden niet meer bewegen. Hij is wat doof aan het worden. Of wat doof, hij is bijna stokdoof. Nou, dat. En eigenlijk vind ik dat dat niet meer kan en dat maak ik dan ook bespreekbaar met die man, waardoor hij al iets meer voor zichzelf de keuze heeft gemaakt om minder te gaan werken. Op die manier probeer ik dat wel. Maar vaak is het, en dat komt omdat het, het is een lagere arbeidsklasse die hier zit. Dus die hebben hun geld hard nodig om hun eigen leven te kunnen onderhouden. Dus op het moment dat je tegen iemand gaat zeggen 'het is misschien verstandiger voor je gezondheid om een stapje terug te doen', dan zit je direct in hun financiën. En dat maakt het moeilijker bespreekbaar voor ze want ze willen daar helemaal niet over nadenken want ze hebben hun geld hard nodig.

**Interviewer:**	Dat is een moeilijk spel?

**Respondent:**	Dat is een moeilijk spel. Maar uiteindelijk weet je als ze zo door blijven gaan blazen ze zichzelf op en dan heb je helemaal geen centen meer. Dus het is maar de wijze waarop je daarmee om gaat.

**Interviewer:**	Het is dus voor die gespreksvoering. Dat is de rol van teamleider. Dingen bespreekbaar maken?

**Respondent:**	Ja. Ja.

**Interviewer:**	Zit daar dan ook een soort doorgeeffunctie aan vast dat je zoiets hebt van 'misschien moet je eens met die gaan praten of eens met die gaan praten?'.

**Respondent:**	Tuurlijk. Je gaat mensen verwijzen naar de specialisten. Dat is wel de bedoeling. Maar je moet als teamleider wel voor ogen hebben wat er mogelijk is. Dus ook zoals >ARBO< nu gedaan heeft, dat is heel goed. Die heeft verteld 'zo zit HR er in'. Dus je weet zodra mensen hier in de problemen raken dat je, en je komt er zelf niet meer uit, dat je HR als hulp in kunt roepen.

**Interviewer:**	Was het voor jou duidelijk wat die mogelijkheden waren?

**Respondent:**	Dat ligt er aan hoe de HR afdeling opgetuigd is. Dat is in elke organisatie weer anders. Je bent nog vrij jong, dus ik weet niet of je dat weet. HR maakt altijd een golfbeweging zeg maar. Op het moment dat er te weinig personeel op de arbeidsmarkt is, wordt HR belangrijker. Op het moment dat het aanbod van het personeel heel hoog, dus de werkeloosheid heel hoog, wordt HR minder belangrijk want dan flikker je er iemand uit en haal je een nieuwe binnen. Bij wijze van spreken. Maar als het personeel wat schaarser wordt, ja dan moet je er zuiniger op zijn en dan wordt HR weer belangrijker. En dat is afgelopen 30 jaar een beetje een golfbeweging aan het worden. Volgens mij wordt het nu weer steeds belangrijker dat HR goed werk levert want er komt weer schaarste aan op de arbeidsmarkt.

**Interviewer:**	Als we eens kijken naar jouw bevoegdheden als teamleider, waarover zou je allemaal mogen beslissen. Over wat voor onderwerpen?

**Respondent:**	Waar doel je dan op?

**Interviewer:**	Ik probeer een beetje in kaart te brengen waar de grens zou liggen als het aankomt op jouw bevoegdheden als teamleider. Dus bijvoorbeeld, vanaf dan zal je met >HOOFD< in gesprek gaan.

**Respondent:**	Ik heb inmiddels geleerd, maar dat is ook door schade en schande, is dat het handig is om je leidinggevende in bijna alles mee te nemen. En dat heeft te maken dat ik >HOOFD< dan in dek. Dat weet >HOOFD< van 'weet wat er speelt, je hoeft er verder niets mee, maar weet dat het in ieder geval speelt' dus ik denk dat dat altijd handig is. En zo moet je ook omgaan met HR met de mensen die hier komen. Ik ben vrij open, vertel altijd alles, dus dat mag gewoon allemaal waardoor, als er ergens een beslissing genomen gaat worden, dat het nooit een verassing voor anderen kan zijn. Vind ik overigens ook met de beoordelingsgesprekken mag ook nooit een verassing zijn. Want als het goed is weet je in de periode daarvoor al wat ik van je vind. Dus zo probeer ik alles van te voren al besproken te hebben. Maar uiteindelijk ben ik verantwoordelijk voor het functioneren van de dames en heren hier en vind ik dat ik moet beslissen  of ze een waarschuwing krijgen of misschien zelfs wel ontslagen moeten worden. Uiteindelijk vind ik dat ik dat moet doen en dat ik de beslissing moet nemen. Maar dat doe ik altijd in samenwerking met anderen en in de voorbereiding op dat traject haal ik mijn informatie.

**Interviewer:**	Heb je ook nog een financiële bevoegdheid, zijn er nog dingen die jij mag kopen? Een bos bloemen of iets dergelijks?

**Respondent:**	Ja. Ik kan de secretaresse altijd opdracht geven om een bos bloemen te bestellen. Dat is verder geen probleem. Dat soort kleine uitgaven is allemaal nooit zo een probleem. Ik heb niet zo een groot budget voor dit team. Maar dat is ook wel eens anders geweest dat je gewoon budgetten van anderhalf miljoen tot je beschikking had om over te beslissen. Dus dat ligt dan ook gewoon wat er gebruikelijk is in een functie. En hier is dat gewoon wat minder.

**Interviewer:**	Je maakt dus niet echt gebruik, dat je geld investeert in het team?

**Respondent:**	Nee. Dat is ook niet echt nodig hoor. Wat er nu gebeurt dan. Vorig jaar zijn er wat investeringsaanvragen geweest voor bijvoorbeeld een beddenmover. Ik heb hem hier toevallig liggen. Dat is een automatisch beddentransport middel. Dat gaat dan via de investeringscommissie, daar moet weer een akkoord op gegeven worden, dan gaat het naar Inkoop, programma van eisen. Een hele bureaucratie er omheen. Maar uiteindelijk mag ik straks wil twintig tot 40.000 euro uitgeven. Dus ook dat gaat weer niet alleen. Ook dat gaat weer in samenwerking met alles en iedereen.

**Interviewer:**	Er zit dus wel een verschil tussen de hele kleine aankopen, die bos bloemen zou je dan in principe zelf kunnen doen...

**Respondent:**	Ja. Dat hoef ik niet eens te overleggen. Dat kan ik gewoon zelf doen. Dat kan ik gewoon opdracht geven om te doen. Dat is verder geen probleem.

**Interviewer:**	En wat vind jij dat een jou een goede teamleider maakt, wanneer levert een teamleider goed werk.

**Respondent:**	Uh... pff... Daar zitten ook weer zoveel nuances aan. Ik denk op het moment dat jij. Een teamleider doet zijn werk goed werk als het werk wordt gedaan door mensen waarvoor die mensen worden aangenomen. Dat is heel kaal. Dat is het. Dat weet je. Die mensen moeten hun werk doen waar ze voor betaald worden. Ze hebben een contract, een arbeidsovereenkomst, krijgen geld en moeten een prestatie leveren. Leveren ze die prestatie niet, dan doen zij het niet goed, dan doet de teamleider het niet goed. In het kort gezegd is dat het. Maar ik vind dat ik moet proberen mijn mensen elke dag een beetje beter moet maken en ik weet niet of dat helemaal de taak van een teamleider is, maar ik vind wel of dat maakt of je een goed leidinggevende bent of niet. Ja dat.

**Interviewer:**	Heb je als teamleider het idee dat je ook nog bepaalde kennis moet hebben om je werk goed te kunnen doen. Dus bijvoorbeeld de taken waar we het over hebben gehad en de beslissingen die je moet nemen?

**Respondent:**	Uh. Ja. Dat vind ik wel. Ik vind wel dat je bepaalde bagage nodig hebt om dat te kunnen doen. Ik ben nu >LEEFTIJD< ben vanaf mijn >LEEFTIJD< leidinggevende al 20 jaar. En de fouten die ik in het begin heb gemaakt, maak ik nu echt niet meer. Maar je hebt het maken van die fouten wel nodig om te weten wat je niet meer moet doen. Of juist wel moet doen. Je kunt dan wel 80.000 van dat soort boekjes lezen, maar uiteindelijk moet je in de praktijk ervaren hoe het echt is. Dus ik zal niet zeggen dat een jonge leidinggevende geen goede leidinggevende kan zijn. Als de intenties maar goed zijn kun je een prima leidinggevende zijn. Maar je gaat fouten maken. En naarmate je wat ouder wordt, zul je de fouten die je in het begin hebt gemaakt zul je niet meer maken. Maar dat is een leerschool. Dat duurt eventjes voor je dat kan. Ik vind ook dat leidinggeven een echt vak is. Niet iedereen kan dat en niet iedereen gaat dat ooit kunnen. Er zijn mensen die fantastische dingen kunnen managen, maar dat maakt ze nog geen goede leidinggevende. Dus een manager en een leidinggevende zijn ook twee verschillende vakken denk ik.

**Interviewer:**	Niet echt specifieke dingen die je moet weten? Hoe het bijvoorbeeld in huis geregeld is of hoe je gesprekken voert?

**Respondent:**	Dat dan weer wel natuurlijk. Ik vind niet dat een leidinggevende inhoudelijk op de hoogte hoeft te zijn van de werkzaamheden. Want als het goed is heb je in je team voldoende professionals zitten die dat werk voor je doen. Jij hoeft dat werk niet te doen uiteindelijk. Je bent er wel verantwoordelijk voor dat die mensen gefaciliteerd worden om het werk te doen, maar dat hoef je niet alleen te doen. Je hoeft niet perse inhoudelijk geschoold te zijn om een goed leidinggevende te zijn. Dat is ook denk ik niet te doen. Misschien met dit wel. Maar ik heb ook leiding gegeven aan juristen, dan moet ik een hele jurstenopleiding gaat volgen, of artsen of zo. Dat is gewoon niet te doen.

**Interviewer:**	Laat je je dan veel adviseren door andere professionals zoals HR? Als er bijvoorbeeld iets met de CAO aan de hand is of de werktijdenwet?

**Respondent:**	Dat bouw je ook op door je ervaring. Dus je weet ook wie voor wat verantwoordelijk is. Dat is in elke organisatie ongeveer wel hetzelfde. Maar je moet wel signaleren. En daar heb je die ervaring voor nodig. Mensen aanspreken op de inhoud van hun werk, met professionals is dat wat moeilijker dan met de ranken die ik om zo maar te zeggen. Maar het werk is niet zo ingewikkeld. Dus dan is het makkelijker om te leren. Maar als je leiding gaat geven aan artsen, dan moet je wegblijven bij die inhoud want daar heb je geen verstand van. Maar je kunt wel wat vinden van hun houding en gedrag en je kunt wel vinden dat het niet in het belang is van het bedrijf. Ik denk dat je heel goed leidinggevende van artsen kunt zijn, zonder zelf arts te zijn.

**Interviewer:**	Dus die intermenselijk kennis die je echt moet hebben?

**Respondent:**	Ja. Sociale kennis.

**Interviewer:**	Dan ben ik wel benieuwd naar jouw visie op de teamleider en voornamelijk wat hoort bij het takenpakket van een teamleider. Wat valt er binnen wat valt er buiten.

**Respondent:**	Dat is een grijs gebied. Alles wat we net besproken hebben van het lijstje, dat hoort er allemaal bij. Maar dat is maar een lijstje. Het gaat er uiteindelijk om of jij je mensen dusdanig gemotiveerd aan de gang krijgt dat zij hun werk gaan doen. Dat is het enige wat ik moet doen. Als zij dat doen ben ik in feite overbodig. En dat klinkt misschien wel raar maar ik heb met al mijn team gezegd 'ik streef ernaar mezelf overbodig te maken'. En dat betekent dus dat zij hun werk goed kunnen doen, zij hun weg binnen de organisatie weten te vinden, en als er wat aan de hand is dat ze de route kennen en dat ze elkaar gaan corrigeren als er dingen niet goed gaan. En als het goed, maar ik heb het nu niet helemaal meegemaakt hoor, als het goed is na een jaar of twee drie zou ik overbodig moeten zijn.

**Interviewer:**	Zijn er dan ook echt nog heel sec dingen waarvan je zoiets hebt 'die horen echt niet bij mij'. Die horen bijvoorbeeld bij >HOOFD< of bij HR?

**Respondent:**	Ja. Dat heeft dan echt te maken met beleid en visie zeg maar. Het hoogoven beleid van het ziekenhuis dat kan ik niet bepalen. Daar heb ik ook genoeg kennis voor. Maar ik vind wel dat ik er wat over te zeggen heb zeg maar. Ik wil er dus wel bij betrokken worden maar ik hoef er niet perse verantwoordelijk voor te zijn.

**Interviewer:**	Een adviserende stem als het ware?

**Respondent:**	Vraag gewoon zoveel mogelijk mensen om iets te maken voor je. Dat is ook wat ik doe op kleinere schaal dan. Ik vraag ook aan mijn team de expertise om een goed plan te kunnen maken. Ik vind dat een bestuurder dat ook moet doen. Die moet ook de mensen zoeken die er verstand van hebben en die hoeft ook niet alles zelf te doen.

**Interviewer:**	Heb je het idee dat een teamleider ook nog op een bepaalde manier zijn taken moet uitvoeren?

**Respondent:**	Hoe bedoel je?

**Interviewer:**	Is er bijvoorbeeld een standaard manier om goed die teamleidertaken uit te voeren of heb je juist zoiets 'je moet het loslaten en de teamleider daarin gewoon zelf een weg daarin te laten vinden?'. Bijvoorbeeld als een nieuwe teamleider zal komen en aan jou zou vragen 'hoe moet ik dit werk nu aanpakken', wat zou je dan zeggen?

**Respondent:**	Dat vind ik wel lastig want de manier waarop ik het je nu vertel is hoe ik er over denk. Maar ik heb ook prima samenwerkt met mensen die er anders over nadenken over leiderschap. Want je hebt verschillende stijlen. Ik ben coachend, mensgericht. Maar je hebt ook hele taakgerichte en resultaatgerichte leidinggevenden of autoritair. Is ook prima als bepaalde groepen mensen bepaalde werkzaamheden vragen een andere soort leiderschapsstijl. Dus ik weet niet waar je nu precies naar op zoek bent maar ik denk dat je dat allemaal zou moeten kunnen. Je zou allemaal een bepaalde rol moeten kunnen vervullen. Het ligt er soms iets dichter tegen je aan als mens zijnde, ligt er een bepaalde rol dichter tegen je aan. Maar als er autoriteit gevraagd wordt, dan moet je dat ook kunnen en moet je eigenlijk je pet kunnen wisselen en eigenlijk een autoritaire leider kunnen worden. Dat is wat lastig en kun je ook wat minder lang volhouden, maar je moet het wel kunnen. Dus nee, eigenlijk weet ik dat niet.

**Interviewer:**	Er is geen standaardformule voor succesvol teamleiderschap?

**Respondent:**	Nee. Dat denk ik niet.

**Interviewer:**	Dat zouden we dus ook kunnen zeggen over wanneer iemand bepaalde werkzaamheden moet uitvoeren. Dat is puur afhankelijk van de context en de mensen die daar aanwezig zijn?

**Respondent:**	Ja.

**Interviewer:**	Kunnen we wel stellen dat het resultaat allesbepalend is?

**Respondent:**	Ja. Uiteindelijk wel.

**Interviewer:**	De weg daar naartoe is dan niet eens het belangrijkste, als het resultaat er maar is?

**Respondent:**	Nou, weet je. De weg daar naartoe bepaalt natuurlijk wat voor leider je bent en wat voor stijl je hebt. Voor mij is dat ontwikkelingsgericht. Anderen kunnen denken 'je moet vanaf het begin die taken goed uit kunnen voeren, doe je dat niet dan ben je niet geschikt voor de functie en kom je er niet voor in aanmerking'. Snap ik ook. Dus de druk op een bedrijf om te presteren is hoog. Dat is in elk bedrijf. Of je nou werkt in de zorg of niet. Je moet altijd presteren en altijd een bepaalde prestatie halen. Ik zelf denk dat je mensen altijd wel kan ontwikkelen naar een bepaald niveau toe. Dat vraagt wat langere tijd, maar uiteindelijk kan dat wel.

**Interviewer:**	Moet een teamleider dan ook nog bepaald gedrag uitstralen?

**Respondent:**	Ja. Die heeft voorbeeldgedrag dat is heel belangrijk. Je kunt wel tegen mensen zeggen dat ze goed moeten letten op hun houding en gedrag, maar op het moment dat je dat zelf niet vertoond. Tja, wat voor voorbeeld geef je dan. Dan is je boodschap ook weg.

**Interviewer:**	Heb je het idee dat de manier waarop jij naar teamleiderschap kijkt dat dat overeenkomt met hoe >HOOFD< erin staat?

**Respondent:**	Ja. Dat denk ik wel. Weet ik niet helemaal zeker. In de sollicitatiegesprekken hebben we het er natuurlijk over gehad over wie ik ben en hoe ik in elkaar steek. Ik ga er vanuit dat >HOOFD< dat herkent en dat gewenst vindt voor het team. Als dat niet zo was geweest, had >HOOFD< mij niet aangenomen. Maar >HOOFD< is als mens natuurlijk al anders en natuurlijk zit >HOOFD< er op een andere manier in dan ik doe.

**Interviewer:**	Ondanks dat je dus niet honderd procent zeker weet dat die verwachtingen gelijk zijn aan elkaar...

**Respondent:**	Nou. Net voordat jij kwam had ik een bilateraal. Dat hebben we een keer in de drie weken. Aan het eind van het gesprek heb ik toevallig gevraagd 'wat vind je er nou van hoe ik het doe?'. Dus ik check dat wel en dat is nog steeds conform wat >HOOFD< wil en wenst want >HOOFD< heeft er geen op of aanmerkingen op. Dus dan ga ik er van uit dat ik wel op de goede manier bezig ben.

**Interviewer:**	Het is niet honderd procent duidelijk wat de verwachting van >HOOFD< is?

**Respondent:**	Dat weet ik niet helemaal. >HOOFD< heeft bepaalde. >HOOFD< vindt dat ik dat zelf moet ontdekken. >HOOFD< laat me ook eigenlijk met rust. Als >HOOFD< ziet dat er iets fout gaat dan komt >HOOFD< me dat vertellen. Dat is verder niet zo een probleem. Maar >HOOFD< vindt voor. Tenminste ik voel. Dat >HOOFD< mij respecteert om wat ik heb gedaan en mij wel vertrouwt dat ik dit tot een goed einde breng en daardoor heb ik geen last van >HOOFD<. Ik weet wel dat het bij andere teamleiders weer anders is. Als >HOOFD< vindt dat het niet goed vertelt >HOOFD< je dat wel. En dat kan soms ook wel eens betekenen dat het soms als vervelend wordt ervaren omdat je aangesproken wordt op dingen die niet goed gaan en het zal wel gek zijn als dat na tweeëneenhalve maand al zo zou zijn toch?

**Interviewer:**	Is het niet enorm stressvol voor jou. >HOOFD< zegt 'je moet je functie als het ware een beetje gaan uitvinden, wat het is'. Is het niet heel stressvol dat de kaders. Het klinkt een beetje vaag namelijk.

**Respondent:**	Ja. Oké. Dat snap ik dat jij dat denkt. Ik denk dat je daar als je beginnend leidinggevende bent meer behoefte aan hebt dan dat je al wat ervaring hebt. Ik weet wel ongeveer wat er van mij verwacht wordt. Het simpelste is '>HOOFD< moet geen last van mij hebben'. Ik moet mijn doelstellingen halen die in het jaarplan afgesproken zijn en hoe ik dat bereik dat is geloof ik lekker mijn eigen probleem. Het personeel moet niet opstandig worden en allerlei mensen in elkaar gaan beuken of zo en er moet een bepaalde ontwikkeling naar de toekomst in zitten. Dus je moet wel een idee hebben van 'oké dit is wat ik met dat team kan gaan doen' en in ieder geval er mee bezig zijn. En ik denk dat dat voldoende is.

**Interviewer:**	Dat jaarplan dat jullie gemaakt hebben is een houvast voor jou?

**Respondent:**	Ja. Dat zijn de afspraken die we gemaakt hebben en dat zijn natuurlijk ook je kaders. En natuurlijk je kaders worden natuurlijk ook bepaald door wat HR vindt weet je over bepaalde ideeën. Kijk ik kan er wel allemaal mensen uit willen schoppen maar je hebt bijvoorbeeld ontslagrecht mee te maken dus je wordt ook wettelijk ingekaderd. Door het bedrijf wordt je ingekaderd van 'zo gaan wij om met onze mensen'. Dus daar zit richting aan. In het begin is het natuurlijk ontzettend uitzoeken als je net begint met leidinggeven 'wat kan wel en wat kan niet?'.

**Interviewer:**	Hoe heb je dat gedaan, dat uitzoeken?

**Respondent:**	Dan loop je soms tegen muren aan van 'ik wil dit' en dat kan dan gewoon niet omdat het niet gebruikelijk is in een bedrijf. Als ik vind dat iemand niet goed functioneert moet ik dat goed kunnen onderbouwen en moeten laten zien wat ik gedaan heb om het beter te maken. Terwijl ik soms naar mensen kijk en denk van 'jij gaat het nooit leren'. Dat ongeduld moet je inperken en dat heeft ook met de jeugd te maken. Hoe jonger je bent hoe sneller je wil en jij denkt wel dat je de wereld kan veranderen maar vaak als je wat ouder bent is dat niet zo.

**Interviewer:**	Duidelijk. Ik ben wel door mijn vragen heen.

**Respondent:**	Heb je er wat aan gehad?

**Interviewer:**	Ja. Zeker. Misschien als laatste vraag. Heb jij het idee dat een teamleider nog bepaalde competenties nodig heeft?

**Respondent:**	Ja. Dat zeker. Ja. Dat zeker. Ja. Ja. Ja.

**Interviewer:**	Wat moet een teamleider kunnen?

**Respondent:**	Wat ik belangrijk vind is dat je sociaalvaardig bent en daar horen wat competenties bij. Sensitiviteit, ontwikkelingsgericht en dat soort termen horen erbij. Dus je moet feeling hebben met je mensen. Dat vind ik belangrijk. En daarbij moet je ook resultaatgericht zijn. Je moet kunnen combineren. Er worden resultaten gevraagd van mensen. Daar worden ze ook voor betaald. Dat is logisch. Maar hoe je die resultaten gaat behalen, dat wordt bepaald door de leidinggevende.

**Interviewer:**	Er zit een harde economische kant, als een zachte sociale kant aan de teamleider?

**Respondent:**	Ja. Als je die goed in balans kunt brengen dan ben je volgens mij een goede leidinggevende.

**Interviewer:**	Top. Ik heb wat ik nodig heb.

**Respondent:**	Alsjeblieft.

**Interviewer:**	Ik weet niet of er nog dingen zijn die je toe zou willen voegen?

**Respondent:**	Nee. Ik denk het niet.

*Einde transcript*

# Transcript Interview FLM 6

**Duur interview:	75 minuten en 17 seconden**

**Functie:	Teamleider**

**Afdeling(en) onder supervisie:	Healthcare**

**Gender:	vrouw**

**Werkervaring in functie:	3.4 jaren**

**Interviewer:**	Misschien leuk om breed te beginnen om eens te kijken wat jouw dagelijkse werkzaamheden als teamleider zijn.

**Respondent:**	Nou.

*Respondent pakt een vel papier met tekst.*

**Interviewer:**	Oh, wauw.

**Respondent:**	Ik kan het je zo voorschotelen. Het is een heleboel en eigenlijk. Het is veel. Het is een grote afdeling> Nu zijn het ongeveer 57 medewerkers en die vallen allemaal onder mij. Het is een afdeling die heel dynamisch is. Heel druk. Pieken en dalen. Verloskunde. Ik heb eigenlijk nooit dezelfde dag. Het is elke dag anders. Ik ben bezig met vanaf binnenkomst, de overdracht, kijken naar de personele bezetting, daardoorheen laveer ik met mijn eigen werkzaamheden. Dus ja al is het allemaal wel een beetje benoemd. Het is niet zo 'vandaag doe ik dit, morgen doe ik dat'. Het is maar net hoe de agenda eruit ziet. De personele planning is wel een van de hoofdmoten, coachen van de medewerkers. Verzuimgesprekken en begeleiding. Jaargesprekken. Verbeterplannen. Kort-cyclische verbeterplannen. Bijvoorbeeld op elke donderdag hebben we een verbeterbordsessie en dan korte dingen die spelen waar ze tegen aanlopen en dan oppakken met elkaar.

**Interviewer:**	Is dat dan op de zorg gericht, of is dat ook op de medewerkers gericht?

**Respondent:**	Op zorg. Waar loop je tegen aan? Bijvoorbeeld apparatuur. Wij zijn een risicovolle afdeling. Is er defecte apparatuur, wie gaat dat oppakken, wat heb je er mee gedaan? Dat soort dingen. De dagelijkse evaluaties tussen half vier en vier. Wat hebben we nog mee op zo'n dag? Nou, om en om de dossierbespreking. Dan zitten de multidisciplinaire artsen er ook bij en de verloskundigen. En de projecten die er lopen. De meeste medewerkers hebben taken en aandachtsgebieden. Dan zorg je ervoor dat je echt een strak plan met ze hebt 'wat koppel je terug, wat heb je nodig, wanneer heb je tijd nodig, hoeveel?'. Om ze zoveel mogelijk de ruimte te geven om aan hun taken te werken. Dat lukt niet altijd. Er is een senior die senior OKE is zoals we dat in huis kennen. Die is ook gewoon de dagcoördinator en als zij er is, dan is het wat rustiger voor mij. Dan lopen ze wat minder vaak hier binnen.

**Interviewer:**	Wat doe zo'n senior OKE in jouw ogen?

**Respondent:**	Ze is meer coördinerend. Dus zoals nu hebben we vandaag twee zieken en dan kom ik in de morgen en dan kijken we met elkaar van 'hoe gaan we dat doen, of moet er meer personeel bij?'. De stand van zaken. Zij gaat naar de grote overdrachten, heeft korte lijnen met de oudste van dienst op de ene kant. Geno is een kant genulde verloskamers. We doen kommetjes. Korte overleg momenten. En daar is zij eigenlijk leading in. Dit is de stand van zaken. Dit hebben we nu nodig en als het spaak loopt trekt zij bij mij aan de bel van 'wij moeten er nu iemand bij hebben'. Daarnaast coördineert ze ook en ziet ze erop toe dat alle taken en aandachtsgebieden dat mensen daar echt iets mee doen. Een actieplan maken, terugkoppeling geven, de weekplanning kijkt ze ook naar. Want ik ben natuurlijk ook een dagje vrij. Dus dan kijkt ze ook 'moet ik personeel regelen'.

**Interviewer:**	Ligt er ook een soort personeelsmanagementfunctie bij de senior?

**Respondent:**	Ze kijkt voornamelijk mee met de weekplanning. We hebben vier categorieën. verpleegkundigen. Kraamverzorgende verpleegkundigen, gespecialiseerd en CCO'ers. In elke dienst doen we ons best om van elk er eentje te hebben. Als eentje ziek is moet dat weer opgevuld worden en kijkt ze mee. Maar dat doen ze overigens allemaal hoor. Alle verpleegkundigen. Want ik vind ook dat ze verantwoordelijk zijn voor de gezamenlijke planning.

**Interviewer:**	Duidelijk. We werk jij zelf ook nog mee?

**Respondent:**	Als het nodig is dan spring ik bij.

**Interviewer:**	Komt het vaak voor?

**Respondent:**	Afhankelijk van drukte. Als het druk is spring ik bij.

**Interviewer:**	Jij hebt een zorgachtergrond?

**Respondent:**	Ja. Ik ben zelf ook gespecialiseerd verpleegkundige.

**Interviewer:**	We hebben het over jouw werkzaamheden gehad en een stukje HR werkzaamheden. Ik heb eens gekeken wat er over het algemeen bij een teamleider zou kunnen liggen qua HR. Zou je eens kunnen kijken welke punten je herkent en op wat voor een manier jij daarmee bezig bent in je functie?

**Respondent:**	Werving. Ja. Werving en selectie dat staat daar ook. Ik heb nu drie nieuwe verpleegkundigen aangenomen en zorg ik ervoor. Ik zit bij de gesprekken. Ik vind het dan belangrijk dat je ook zelf mensen van de werkvloer erbij betrekt. Dus daar zitten daar dan gespecialiseerd verpleegkundigen of kraamverzorgenden. Dat is maar net wat je werft. Ook bij de gesprekken.

**Interviewer:**	Ligt bij jou dan de beslissing 'ik neem iemand wel of iemand niet aan?'.

**Respondent:**	Ja. Wij zijn een gespecialiseerde afdeling. Dus ik weet precies wat we nodig hebben om het hier draaiende te houden. Introductie van nieuwe medewerkers zeker. De senior doet dat ook. Dat is meer in de vorm van. De senior doet bijvoorbeeld degene rondleiden, instructies geven over hoe dat werkt en ik heb een gesprek met diegene over 'wat doe je bijvoorbeeld als je ziek bent, als iets extra's wil doen, hoe zit het met je dienstroosterplanning, waar vind je het rampenplan?'. Dat hele rijtje bespreek ik dan met ze. Wat verwacht je van hun. Eigenlijk het totaalplaatje.

**Interviewer:**	Duidelijk. En als het aankomt op het volgende punt?

**Respondent:**	Ja. We hebben Youforce. Daar doe ik de jaargesprekken in. Ziekteverzuim. Daar hebben we nu een mooi verzuimsignaalsysteem voor. Daar kun je ook je notities maken, je dossier bijhouden, en als ik gesprekken heb. Iedereen heeft bij mij een mapje. En gesprekken, een kort verslagje en in de map. Dat ik het terug kan vinden. Diegene krijgt hem ook. Ze mogen hem tekenen. Maar vaak tekenen ze het niet. Lezen ze hem. Beslissen over planning ontwikkelingsbehoeften medewerkers. Ja zeker. Ik geef input aan het opleidingsplan en scholingsplan. Als ze bij mij in het jaargesprek aangeven dat ze een opleiding willen volgen dan is het. Dan kijk ik ook of de opleiding 'is het relevant voor deze afdeling?'. Dan schrijven zij een motivatiebrief en gaan ze zelf op zoek waar de opleiding wordt gegeven en wat houdt het in, hoe kan ik het hier toepassen? Dat wordt bij mij ingeleverd en dan kijk ik 'goh, we kunnen daar inderdaad wel wat van gaan leren'. Zoals nu bijvoorbeeld heb ik nu iemand die bijvoorbeeld de informaticaopleiding wil doen. Die heeft dat twee jaar geleden aangegeven en die volgt hem nu. Dat is een mooi voorbeeld. Borstvoedingscoaches ook. Die mogen dan een motivatiebrief schrijven en dan houd ik een soort tien minuten kwartier gesprek, samen met iemand van de werkvloer in dit geval dan een lactatiekundige of een applicatiebeheerder, en dan nemen we een beslissing of diegene...

**Interviewer:**	Neem jij dan de uiteindelijke beslissing of diegene wel of niet op training of op cursus gaat of is dat >BEDRIJFSLEIDER<?

**Respondent:**	Dat doet ik samen met >BEDRIJFSLEIDER<. Wij kortsluiten altijd met elkaar van 'goh, joh dit ligt klaar op tafel, hoe kijk jij er tegen aan, wat vind jij ervan, wat heb je nodig, wat gaat het ons opleveren?'. En dan nemen we een besluit. Formele training waar moet ik dan aan denken?

**Interviewer:**	Formele training zou dan echt zijn dat je voor de groep gaat staan, er is bijvoorbeeld iets nieuws, dat jij daar dan uitleg over geeft.

**Respondent:**	Voorheen wel. We zijn natuurlijk een opleidingsziekenhuis dus heel vaak gaat het bij ons om beleidsverandering en protocollen. Dat soort dingen. Wat ik merk is dat degene van de werkvloer het ook prettig vinden, zoals nu een mooi voorbeeld. Een fluxusprotocol dat moet opnieuw geïntroduceerd worden. Dan wordt onze beroepsgroep, dat is de NVOG, en dan gaan er twee verpleegkundigen met een arts-assistent en een verloskundige om tafel zitten. Dat is de werkgroep. Zij nemen dat stukje over zeg maar. Dat wordt helemaal uitgewerkt. En toevallig nu de komende vier dagen is er aankomende vier middagen een presentatie voor het hele team. Ik ben ervan op de hoogte want ik zit altijd in de mailwisseling. Dus ik weet wel wat er gebeurt. Maar zij mogen wel het zelf allemaal doen.

**Interviewer:**	Zij pakken dat dus zelf op?

**Respondent:**	Ja.

**Interviewer:**	Een stukje informele training...

**Respondent:**	Coaching en ondersteuning.

**Interviewer:**	En wat voor manier gaat dat dan, kun je daar wat meer over vertellen?

**Respondent:**	Bijvoorbeeld nu heb ik een verpleegkundige die had een gespecialiseerde opleiding verlieskunde gedaan en die wilde heel graag iets betekenen voor de afdeling. Ik weet niet of het op het journaal gehoord hebt? Wij zijn uniek in Nederland met de *>geschrapt wegens identiteit ziekenhuis<.* Dat kwam van een van onze collega's. Vanaf het begin af aan help ik haar met het maken van een projectplan, het ondersteunen daarin. Het coachen en begeleiden. Tot dit het einddoel er is. Dat is heel mooi. Soms denken ze dat ze iets niet kunnen. Maar als je daar die begeleiding en ondersteuning geeft en samen naar informatie op zoek gaat. Dan zie je, het komt er wel. Ook bij de verloskundige. De verloskundige groep bij ons is best een zelfsturende groep. Als zij dingen hebben dan is het altijd even met elkaar zitten en het erover hebben en duidelijk maken aan elkaar 'wat hebben we nodig en hoe gaan we dit doen? Wie gaat dat dragen, wie is eigenaar daarvan?'.

**Interviewer:**	Jouw rol is daarin ondersteunend?

**Respondent:**	Ja.

**Interviewer:**	Puur vragen 'wat heb je nodig om dit mogelijk te maken?'.

**Respondent:**	Ja. Ik communiceer ook naar het team toe. Dus dan is het altijd wel belangrijk dat ik op de hoogte ben dat als het team vragen heeft, dat ik die ook kan beantwoorden. Mentoren, daar heb ik net al een beetje een voorbeeld van gegeven. Functioneringsgesprekken doe ik, beoordelingsgesprekken doe ik. Maar ook gewoon. Ik doe heel vaak coachingsgesprekken als ik zie dat mensen niet lekker gaan of niet lekker in hun vel zitten.

**Interviewer:**	Hoe neem je dat waar, hoe krijg je dat binnen?

**Respondent:**	Ik ken ze door en door. Ik zie ze ook gewoon op de gang lopen. Dan denk ik 'ziet die een beetje moe?'. Wat ik zelf prettig vind, ze weten mij te vinden. Ze komen wel makkelijk. De drempel is nu. Ik stimuleer ze ook om dat te blijven doen. Want ik ben lang niet altijd op de werkvloer dat ze mij kunnen aanspreken. Maar ze komen wel. Of ze mailen of sturen een berichtje van 'goh, kan ik even met je praten'. Dan maak ik daar tijd voor. Mijn verbeterpunt is op tijd naar huis gaan. Wat nog best last is. Sommige draaien heel veel avonddiensten en nachtdiensten. Dan zie je ze heel weinig. Dan is het contact sowieso al minder. De tijden waarop zij mij nodig hebben en zij mij weinig hebben gezien dan maak ik tijd voor ze.

**Interviewer:**	Dus ook als dat moet in de avonduren?

**Respondent:**	Ik blijf tot vijf uur. En als de avonddienst begint, die begint om kwart over drie. Meestal om half vier gaan ze echt aan het werk na de overdracht. Of er is nog een klinische les of een scholing. Vier uur. Tussen vier en vijf maak ik tijd voor ze. En als iemand gelijk aan de slag gaat omdat er een bevalling is, dan kwartiertje tot kwart over vijf, half zes, dat vind ik niet er.

**Interviewer:**	Wel fijn dat je nog een puntje van ze mee kunt maken van hun dienst?

**Respondent:**	Ja. Ik maar ook echt tijd voor ze. Akkoord geven op ontwikkelingsplannen. Dat doen >BEDRIJFSLEIDER< **en ik samen. Als** >BEDRIJFSLEIDER< beleidsdocumenten maakt en schrijft dan maken we dat daar altijd een onderdeel van. We geven ook input. Dan maakt >BEDRIJFSLEIDER< het plan en dan meestal als we teamleidersoverleg hebben dan kijken we samen op de computers of is er geschreven, kunnen wij ons daar in vinden?

**Interviewer:**	Wie is ons?

**Respondent:**	De teamleider van >AFDELING<. Wij zijn met zijn tweeën. Ik ook op de afdeling >TEAMLEIDER< op de poli. Dan bekijken we dan, dan bespreken we met elkaar van 'goh, zijn we het er mee eens, als we input geven vraagt >BEDRIJFSLEIDER< ook van 'goh, kun je je er in vinden of moet het anders geformuleerd worden?'. Dat soort dingen. Disciplineren een corrigeren. Disciplineren en corrigeren. Ik probeer een beetje voor de hand liggende. Voorbeelden hoef ik niet zo te graven. Als ik het altijd hoor discipline. Nu heb ik dan die protocollen waarvan ik denk 'de IGZ kan altijd binnen stappen' en dan moet ik toch altijd een beetje. Ik moet toch aan de kar gaan trekken dat ze toch op de hoogte zijn van alle informatie. Dus ik moet daarin soms ook gewoon zeggen van 'goh, het is niet omdat het nodig is, gewoon omdat het moet'. Leren. We leren onszelf ook aan de nieuwsbrieven te lezen of intranet op Idoc intranet alle informatie op te zoeken. Zeker omdat ik een keer per kwartaal een veiligheidsronde hier over de afdeling loop. Samen met collega's, maakt niet uit wie er is. Dan komen daar verbeterpunten naar voren en die ik die zoals nu in de nieuwsbrief. Die gaat deze week eruit. Het is misschien een zwaar woord om het te eisen. Maar ik leg de nadruk er wel op dat het hun eigen verantwoordelijk is om het daadwerkelijk die informatie tot zicht te nemen.

**Interviewer:**	Dit is wel echt een moetje?

**Respondent:**	Ja.

**Interviewer:**	En als je dat dan in jouw nieuwsbrief schrijft, schrijf je die dan aan de persoon of is het meer in het algemeen 'jongens, let op ik heb dit en dit gezien, let op'.

**Respondent:**	Ja. Ik heb een stapeltje met verbeterpunten. Die heb ik nu ook geformuleerd. En dan is het algemeen omdat ik denk dat het niet alleen voor de verpleegkundigen is, maar ook voor de artsen en voor de verloskundigen die heel veel niet weten. Want zij krijgen ook de nieuwsbrief.

**Interviewer:**	En het team voelt zich wel aangesproken als het zo in het algemeen wordt geformuleerd?

**Respondent:**	De ene minder dan de ander. Ik merk wel dat als ik die veiligheidsronde loop dat ik dan een keer in de week steekproefsgewijs dat we ook de hot items even doornemen. Vandaar ook dat we het verbeterbord hebben van 'jongens let er op, we kunnen elk moment een onaangekondigd bezoek krijgen, dus awareness voor'. En natuurlijk vinden ze dat soms lastig, niet allemaal hoor. Je hebt een paar diehards die zijn van alles op de hoogte. Die lezen ook keurignetjes alle nieuwsbrieven. Sterker nog, als ik dan vrijdag de nieuwsbrief niet verstuur, dan vragen ze van 'goh, we hebben geen nieuwsbrief gehad'. Maar je hebt ook een deel, dat is niet zo interessant. Dat zijn mensen die werken en gaan weer naar huis. That's it.

**Interviewer:**	Ligt er dan ook nog een rol bij jou dat iemand zich niet aan die awareness dingen houdt?

**Respondent:**	Het is niet alleen gedrag, maar daar corrigeer ik ze ook in. Van 'goh, joh, aanstaande vrijdag gaan we borstvoeding her-certificeren', dan zeg ik ook van 'joh, jullie hebben alle informatie gehad, lees het dan ook'. We kunnen natuurlijk ook toetsen 'heb je het gelezen'. En soms roep ik iets en dan zeg ik van 'goh, wie heeft dat gelezen?'. En dan weet je het al als niemand antwoord geeft. Ik vind dat stukje een lastige. Jezelf voorzien van informatie.

**Interviewer:**	Wat maakt het lastig?

**Respondent:**	Er wordt altijd geklaagd, geroepen, dat er geen tijd is om het te lezen. En in mijn beleving is dat niet zo. Tuurlijk hebben we drukke dagen dat we veertien baby’s dat er geen tijd is voor eten zelfs. Maar er zijn ook dagen waarop iedereen. Je zit op de computer, haalt je pasje er doorheen en je kunt het lezen. Het is echt wel een soort van discipline die jezelf moet leren.

**Interviewer:**	Het moet in je patroon opgenomen worden? Dat als je tijd hebt dat je dat even in het lezen steekt?

**Respondent:**	Het wordt ook op het bord gehangen. Ik print het uit. Ik hang ze ook op het bord. Dat hij ook fysiek gewoon te pakken is.

**Interviewer:**	Een nog lagere drempel?

**Respondent:**	Hmm.

**Interviewer:**	Oke. Ik kan me voorstellen dat het lastige is. Heeft het ook met een stukje opvoeden te maken?

**Respondent:**	Nou. Het is tweeledig. Ik denk dat de jongelui die we nu aannemen. We voeden ze dusdanig op dat het erin gaat zitten. Maar je hebt ook een deel, die is al zo lang op de afdeling. Dat is een soort van starheid. Rigiditeit weet je wel? Van 'pff'.

**Interviewer:**	Ingesleten?

**Respondent:**	Ja, dat is soms wel eens lastig. Afhandelen van tuchtprocedures en klachtprocedures. Tucht nee. Klachtenprocedures wel. Daar ben ik nauw betrokken. Wij hebben onze eigen afdelingsenquêtes en dan krijg ik elke maand krijg ik zo'n analyse. Dat doet een verpleegkundige. Die maakt daar een analyse van. Daar staan ook soms telefoonnummers bij van mensen die teruggebeld willen worden. Soms is het ook een compliment hoor. Dat is wel leuk. Dan bel ik mensen terug. Het is ook zo dat we soms brieven krijgen via de klachtenfunctionaris. Dan speel ik daar een grote rol in om de mensen te bellen. Heel vaak stel ik ze voor de keuze of ze het gesprek telefonisch het gesprek willen of dat ze persoonlijk willen. Dus ik heb heel vaak hier voor de klachtgesprekken. En voor de verpleegkundige die erbij betrokken is die spreek ik, dat klinkt heel gek, maar die spreek ik er dan wel op aan. Zeker als de klacht op naam is worden ze daar ook gewoon mee geconfronteerd kun je zeggen. Gelukkig.

**Interviewer:**	Ik kan me voorstellen, het is niet leuk om te lezen, maar het is wel waardevol om te lezen.

**Respondent:**	Ja. Zeker als het op naam staat. Als het niet op naam staat is het lastiger. Dan gis je een beetje. Gelukkig hebben we ons elektronisch patiëntendossier. Daar staat altijd een digitale handtekening en een naam erachter. Die kun je altijd achterhalen afhankelijk van de situatie die geschetst is 'wie, wat, waar, hoe laat, wat is er gerapporteerd?'. Je hebt altijd wel een aanknopingspunt om terug te koppelen en te proberen zo tactvol mogelijk te brengen. Want ik geloof er in dat elke verpleegkunde die hier werkt de beste intenties heeft om de patiënt zo goed mogelijk te helpen. Helaas hebben we toch in stijgende lijn patiënten die hoge verwachtingen hebben en veel meer eisen stellen. Dus dat moet je dan ook meenemen. Maar soms denk je ook van 'dat kan echt niet'. En dan maak ik dat wel bespreekbaar.

**Interviewer:**	Wat dan uit die klachten komt, gebruik je dat dan ook wel eens voor je nieuwsbrief?

**Respondent:**	Nieuwsbrief soms. Afhankelijk van. Het is een vast agendapunt van onze vergaderingen. Dus altijd klachten en DIM als vast punt. En dan bespreken we de klacht. Kijk je hoeft de persoon niet met naam en toenaam te gebruiken. Je kunt het wel benoemen om er van te leren.

**Interviewer:**	Die teamvergadering is hoe vaak per jaar?

**Respondent:**	Zes keer per jaar.

**Interviewer:**	En dan gewoon met het hele team bij elkaar?

**Respondent:**	Ja. Je krijgt ze lang niet altijd allemaal bij elkaar. Maar over het algemeen is de opkomst wel goed. Even kijken. Absenties. Ja. De verzuimgesprekken. Ik probeer wel als ik vooraf zie dat mensen er al een beetje doorheen zitten om alvast een beetje beleid uit te stippelen samen en in gesprek te gaan van 'goh, hoe kunnen dat ondervangen, wat heb je nodig?'. Ik zet ze wel aan het denken. Zelf aan het werk. Wat heb je nodig, wat voor ondersteuning? Eigenlijk het ziekmelden voor zijn?

**Interviewer:**	Heb je het idee dat je alles goed meekrijgt wat hier op de afdeling gebeurt?

**Respondent:**	Over het algemeen wel. Tuurlijk zijn er onderling wat dingetjes die ze wat eerder met een collega zouden bespreken dan met mij. Dat vind ik prima. Maar als het erop aankomt of iemand het idee heeft uit te vallen of noem het maar op. Dan vinden ze mij wel.

**Interviewer:**	En jij weet hen ook goed te vinden?

**Respondent:**	Ja. Zeker. Ja. Meestal zie ik ze op de gang al van 'hmm. Jij bent niet zo vrolijk vandaag. Speelt er iets?'. En als ik zie 'dit was vorige week ook', dan zeg ik van 'goh, zullen we samen een kopje thee drinken?'.

**Interviewer:**	Is het dan voornamelijk lichaamshouding die jou dat jouw signaalfunctie is van 'zal ik eens doorvragen?'.

**Respondent:**	Ja. Of soms zit je aan de koffie met ze. Dat probeer ik ook zo vaak mogelijk te doen. Met hun regelmatig koffie drinken of tijdens de lunch. Dat lukt mij niet altijd. Maar ik doe wel mijn best. En dan hoor je wel eens wat. En dan moet ik die even onthouden voor als. Maar over het algemeen komen ze wel. Ik heb nu twee die tegen mij gezegd hebben 'ik ben best wel moe'. Dus ik zeg 'goh, hoe zou je het anders willen dan?'. Ik schotel het niet direct voor maar ga eens zelf denken van 'hoe kun je jezelf ontlasten dat je zelf van de moeheid af kan wil komen'. En vaak komen ze wel met een voorstel van 'nou, misschien kan ik daar en PLB-dagje, of misschien twee uurtjes minder. Ja. Waardering geven. Dit puntje is een groot punt. Want ja. Wat versta je onder waardering. Dat is zo lastig.

**Interviewer:**	Wat valt er voor jou onder waardering?

**Respondent:**	Ik denk als je voldoende persoonlijke aandacht geeft, tijd aan besteed. Ze krijgen hier altijd als iemand ziek is een kaartje, een bosje bloemen, een presentje met sinterklaas, we hebben afdelingsuitjes. Er is altijd wel iets. Ik geef ze complimenten. De nieuwsbrief is altijd als een maand druk is geweest, dan is de eerste regel in mijn nieuwsbrief van 'jongens, het is ontzettend druk geweest, complimenten voor jullie inzet. We hebben zoveel babies zijn geboren'. Dat doe ik altijd. Maar ook gewoon persoonlijk en in gesprekken. En zeker ook met de jaargesprekken en tussentijds. Dat ik zeg van 'goh, je bent een goede verpleegkundige, houd vast'. Tuurlijk heb je altijd nog punten waar je aan kunt werken. Maar ook dat is een stukje waardering dat iemand bereid is door te groeien of zich te ontwikkelen, op wel gebied dan ook. En dan laat ik ze ook wel. Ook zoals nu met de POP-gesprekken ook, van 'goh, wat heb je nodig, wat vind je leuk om te doen, wat past bij je, welke kwaliteiten heb je?'. Dat vind ik zelf ook wel een stukje waardering. Dat je mensen vanuit hun eigen kracht, nou ja gebruikt is misschien grot gezet, maar dat je daar gebruikt van maakt. Van de dingen waar mensen goed in zijn. Dat heb ik net gezien met het project Verlieskunde. Het is voor diegene wel heel leuk dat het voor diegene zo'n eindresultaat heeft opgeleverd. Maar ook voor ons. Dan zie je ook wel dat het hele team het waardeert. Dan zeg ik ook tegen ze van 'zo kan het ook'. Dus degene die niet zin hebben om iets te doen die kunnen hiervan ook prikkels krijgen van 'goh, misschien is het ook leuk om eens iets te gaan doen'. Beslissen over salarisschaal. Hmm. Nee. Weet je, we hebben hier in 2008 een bijzonder moment gehad dat toen de functiedifferentiatie der kwam en dat de gespecialiseerde gewogen werden en een schaal omlaag gingen. Dat heeft zoveel tamtam gebracht. Wat we wel besproken hebben, >BEDRIJFSLEIDER< en ik, is om als we de jaargesprekken houden om te kijken 'gaan we als mensen bovengemiddeld scoren, wat doe je dan?'. Geef je ze een bonus of hoe geef je daar aandacht aan?

**Interviewer:**	Dat is wel iets dat je samen doet met >BEDRIJFSLEIDER<? Het is niet zo dat je zelf die bonus uitschrijft?

**Respondent:**	Nee. We hebben het erover gehad en we hebben afgesproken dat we vanaf volgend jaar zullen kijken als mensen bovengemiddeld zijn wat daar tegenover staat.

**Interviewer:**	Maar altijd in samenspraak?

**Respondent:**	Ja. Bottom-up communicatie. Naar >BEDRIJFSLEIDER< toe?

**Interviewer:**	Juist. Zie jij een rol dat je berichten van de medewerkers naar >BEDRIJFSLEIDER< communiceert?

**Respondent:**	Ja. Een keer in de twee weken hebben we een overleg en daar informeer ik haar over eigenlijk alles wat gaande is op de afdeling. Van leukigheden tot minder leuke dingen. Ook qua beleidsverandering. Personeel. Ziekte. Daar informeer ik haar over omdat ik vind dat zij dat wel moet weten.

**Interviewer:**	Werkt dat ook de andere kant op? Dat >BEDRIJFSLEIDER< dingen heeft gehoord van bijvoorbeeld de EVR-voorzitter, dat >BEDRIJFSLEIDER< dat weer deelt met jou en dat jij dat weer terugzet naar het team?

**Respondent:**	Nee. Nou. Kijk. Ik vind zelf dat ik. Ik zit in het midden. Ik gesandwiched tussen twee. Ik vind wel dat ik weinig informatie van dingen die gaande zijn krijg. Ik moet zelf achter de dingen aan. En ik ben wel alert. Want als ik weet dat er dingen staan te gebeuren, dan. Ik ken mezelf. Ik ga daar echt achteraan en dan vraag ik het ook.

**Interviewer:**	Hoe ben je daarvan op de hoogte?

Door het te vragen weet je. In de ochtend lees ik altijd even intranet en daar staan altijd dingen waarvan ik denk 'oh ja, dat staat te gebeuren'. En dan vraag ik ook aan >BEDRIJFSLEIDER< van 'ik lees dat en dat, is dat iets wat ik moet weten. Of is dat geregeld?'.

**Interviewer:**	Je gaat heel actief informatie halen?

**Respondent:**	Ja. Ik moet dat wel doen. Ander heb je kans dat ik informatie mis.

**Interviewer:**	En dat geldt dan ook voor medewerkers?

**Respondent:**	Ja. Er zit wel een soort doorgeeffunctie, maar van medewerker naar >BEDRIJFSLEIDER< makkelijker dan andersom. Omdat ik vind dat >BEDRIJFSLEIDER< het moet weten. Als iemand bijvoorbeeld langer dan twee weken ziek is, dan meld ik >BEDRIJFSLEIDER< van 'diegene is ziek van dat en dat'. Maar ik vind wel als ik een verzuimgesprek ga voeren omdat het dan vier weken is, en ga je of naar de verzuimspecialist of naar de bedrijfsarts? Dan zeg ik ook tegen >BEDRIJFSLEIDER< 'het duurt te lang, ik zie diegene nog niet terugkomen dus'. Dan vraag ik ook aan >BEDRIJFSLEIDER< 'hoe kijk jij hier tegen aan, welk advies of tip heb je?'. Ook als ik lastige jaargesprekken heb, dat ik aan >BEDRIJFSLEIDER< vraag van 'goh, heb jij nog bepaalde punten die ik moet meenemen'. Ik heb nu iemand waarvan ik gezegd heb van 'daar ga ik geen gesprekken meer mee voeren, die is voor jou'.

**Interviewer:**	Hebben jullie de jaargesprekken onderverdeeld of doe je in principe alle jaargesprekken?

**Respondent:**	Ja.

**Interviewer:**	Dit is dan een uitzonderingsgeval die naar >BEDRIJFSLEIDER< gaat?

**Respondent:**	Ik heb mooie jaargesprekken. Maar je hebt altijd wel een iemand die. En weet je kan het blijven doen.

*Telefoon gaat.*

**Interviewer:**	Ligt er ook nog een rol bij jou als het aankomt op het aanhoren van suggesties van medewerkers? Als ze een idee hebben of iets degelijks.

**Respondent:**	Ja. Zeker. Waar ik nu achter ben. We hebben het medewerkerstevredenheidsonderzoek gehad en ga je ook met elkaar om de tafel zitten. We hebben een teamvergadering gehad waar het ook wel gepresenteerd is. En ja, dat zij ook zeggen 'zullen we eerder naar >TEAMLEIDER< gaan, dan dat we naar jou gaan'. Je bent er wel, maar je bent er ook niet. De deur is dicht. >BEDRIJFSLEIDER< is ook met andere dingen bezig. Daarom zeg ik ook 'zij vinden hun weg wel naar mij'. Ik weet dat het wederzijds vertrouwen er is en dat als er dingen spelen, dat ze komen. Het is wel een puntje van de communicatie kan beter. Met name van >BEDRIJFSLEIDER< naar mij. Dat kan beter. Het is ook wel zo namelijk dat ik denk van 'oh, ik heb begrepen dat ik op die en die dag dat moet doen, hoe zit dat dan? Dat weet ik niet'. Ja. Dat kan soms lastig zijn.

**Interviewer:**	Hoe doe je dat dan? Er wordt iets van je verwacht. Maar hoe je dat invult, zeg je net zelf, dat is hmm. Hoe ga je dan vervolgens te werk?

**Respondent:**	Nou meestal. Ik vraag het gewoon. Ik ben altijd heel makkelijk daarin. Van 'goh, joh, laatst in de wandelgangen, het is een klein voorbeeldje hoor, zegt >BEDRIJFSLEIDER< 'die personele bezetting op donderdagmorgen is er donderdagmorgen een overleg. Bedrijfsleiders die zitten daar bij'. Vroeger ging ik daar heen, maar nu is het zo gerold dat het niet meer hoefde. En nu. Per 1 april ga jij dat doen. Oh. Oke. Hoe? Het is zaterdag 1 april, dat wil zeggen dat ik volgende week ergens naartoe moet. En iets moet gaan doen. Ik weet het niet.

**Interviewer:**	Dus nu ga je straks aan >BEDRIJFSLEIDER< vragen van 'oke, wat verwacht je nou precies van mij. Wat wil je dat ik doe?'.

**Respondent:**	Ja. Dat ga ik vanmiddag vragen. Van 'is dat structureel, waar moet ik zijn, wat de reden ervan is dat ik nu er naartoe moet, wat wordt nu van mij verwacht?'. Soms is het best wel lastig. Zoals nu ook. Soms lees ik dingen over de mail. Als >BEDRIJFSLEIDER< dingen naar het team stuurt dan lees ik ook dingen en dan denk ik ook 'oh, het was handig geweest als ik dat ook had geweten'.

**Interviewer:**	Het komt dus ook wel eens voor dat er vanuit >BEDRIJFSLEIDER< direct naar het team wordt gecommuniceerd? Dat >BEDRIJFSLEIDER< jou overstijgt?

**Respondent:**	Ja. >BEDRIJFSLEIDER< stuurt wel eens mailtjes en dan zeg ik ook van 'ik vind dat jij dat moet sturen'. Dan doet >BEDRIJFSLEIDER< dat ook. Er komen ook wel eens mailtjes voorbij dat ik denk. Dan vind ik belangrijk dat ik weet, als zij er niet is, dat ik een back-up bedrijfsleider heb. Ze vervangen elkaar. En ik heb nu in de mail gelezen wie >BEDRIJFSLEIDER< vervangt deze twee weken.

**Interviewer:**	Dus dat is dan een verassing achteraf?

**Respondent:**	Ja de bedrijfsleider van de Kinder, die was altijd de vervanger van >BEDRIJFSLEIDER<. En nu lees ik een totaal andere naam en denk ik van.

**Interviewer:**	Daar moet je het dan mee doen?

**Respondent:**	En nu heb ik even niets gevraagd. Ik kreeg dat mailtje en toen was >BEDRIJFSLEIDER< er niet. Ter kennisgeving zeg maar. Ja. Ja. Je weet ook niet of er iets van een overdracht ligt. Dat weet ik niet.

**Interviewer:**	Dus jij moet nu gaan uitzoeken 'wat is de situatie?'.

**Respondent:**	Dat vind ik soms wel eens lastig. Ik mis een stukje. Ook met simpele dingen. Ook met vrije dagen enzo. Weet je wel. Dan hebben we gezegd 'we stemmen op elkaar af'. Woensdag vrijdag. >BEDRIJFSLEIDER< is er op de woensdag en ik ben vrij. Op een gegeven moment heb ik dat losgelaten want dat liep niet. En natuurlijk denk ik 'ook een beetje naar gelang je agenda'. Als ik denk woensdag vrij te zijn, en er staan heel veel afspraken in mijn agenda. Dan zeg ik van 'vrijdag heb ik minder', die kan ik wel afzeggen. Dan neem ik vrijdag vrij. Dat communiceer ik altijd. Van 'ik ben er vrijdag niet'. Dat het was soepeler verloopt.

**Interviewer:**	Eigenlijk alles wat jij mist, dwingt jou er toe om vragen te stellen en vragen van 'wat verwacht je precies'?

**Respondent:**	Ja. Ja. Kijk. De poli is er natuurlijk ook. Het is een vrije grote EVR. Daar houd je ook rekening mee. Dat je niet over te allen tijde kan zijn. En binnenkort heeft >BEDRIJFSLEIDER< er een andere EVR bij. Dus ik moet mezelf zien hoe dat zal verlopen. We gaan het zien.

**Interviewer:**	Zijn er verder nog punten op het lijstje waarvan je denkt 'daar ben ik mee bezig'?

**Respondent:**	Aanhoren van suggesties, dat heb ik geschetst, die zijn er zat. Coördineren van werk van teams en ploegen. Ja. Phoe. Onderhouden van effectief teamwerk zeker. We zijn een gespecialiseerde afdeling. Je hebt een team verloskunde. Een team zwangere kraamvrouwen. En het blijft een puntje van aandacht om die met elkaar samen te laten werken in een dienst als het druk is.

**Interviewer:**	Ligt daar nog een rol voor jou weggelegd?

**Respondent:**	Ja. Zeker. Het begint bij de planning. Deze mogen ze zelf invullen. Wie waar werkt. Ik vind zelf namelijk ook dat dat iets is wat zij prima zelf kunnen doen. Stuur daar zelf op. Dat ze weten wie daar kan werken en wie wat kan. En soms is de indeling gemaakt en zijn ze het er niet mee eens. En ik zeg altijd van 'joh, ga met je collega's in gesprek en als het niet lukt'. Soms moet ik wel eens bijspringen als ze een clashje met elkaar hebben. Tja. Ik denk dat je dat in ieder team hebt. Het is niet anders. Personeel consulteren.

**Interviewer:**	Dat gaat in de richting van het aanhoren van personeel. Maar het komt er op neer dat als jij naar >BEDRIJFSLEIDER< gaat, dat jij dan eerst nog even aan het personeel vraagt van 'goh, wat vinden jullie er van?'.

**Respondent:**	Ja. Vaak als >BEDRIJFSLEIDER< punten inbrengen bij de evaluatie, dus tijdens het verbeterwoord, maar ook één-op-één. En dan komen ze wel met bepaalde punten waarvan ik denk. Ja. Vaak vraag ik het wel 'wil je dat ik dit met >BEDRIJFSLEIDER< bespreek?'. Het is ook van vrouwen he. Heel gevoelig allemaal.

*Interviewer lacht.*

**Respondent:**	Gezondheid en veiligheid en verbeteren van werklevens. Ja. Daar besteden we zeker aan dacht aan. De oudere garde die hebben bij mij een PLB-plan ingeleverd. En ik stimuleer ze ook om deel te nemen aan workshops over vitaliteit. Dat soort dingen. Dat ze het wel doen. Veiligheid. Kijk we hebben heel veel risicovolle apparatuur hier. En soms merk je ook wel dat het irritatie is van defecte apparatuur. Of als dingen het niet doen. Of als ze dingen moeten versjouwen. Ook inherent aan de leeftijd. De jongeren hoor je iets minder dan de ouderen. Veilig werken op een ander vlak is dat is ik nu in de nieuwsbrief heb opgenomen is een stukje over datalek. De clean desk principe. Dat ze weten wat het inhoudt. Informatiebeveiliging. Hoe ga je daarmee om. Maar ook veiligheid onderling. Als collega's onderling een geborgen en veilig gevoel om dingen tegen elkaar te zetten. Daar hebben ze nu ook de cursus Verbindend communiceren voor. Daarvan hebben we afgesproken 'als we het doen, dan gaat het hele team, inclusief met zelf en inclusief >BEDRIJFSLEIDER<'. Dan gaat iedereen die cursus ook volgen. Dus rond mei, als iedereen de cursus heeft gevolgd, gaan we een vergadering plannen of een sessie plannen om van gedachten te wisselen over hoe dat nu in de praktijk kunnen toepassen. Wat er allemaal geleerd is.

**Interviewer:**	Als we nou eens naar jou bevoegdheden als teamleider. Zou je dan eens aan kunnen geven waarover je wel en waarover je niet zou mogen beslissen.

**Respondent:**	Nou. Eigenlijk beslis ik al een heleboel. Als het maar werkvloer-gerelateerd is. Als het operationeel is. Dat soort dingen. Personeel. Werving en selectie. Over verzuim. Daar heb ik nauw contact mee met de verzuimspecialist. Dan hoef ik ondertussen niet te vragen hoe of wat. De rest plan ik allemaal zelf.

**Interviewer:**	Waar zou je wel toestemming voor vragen?

**Respondent:**	Zoals me de formatie, dat houd ik ook zelf bij. Inkoop doe ik ook tot 2.500 euro mag dat. Ik. Zo een, twee, drie. Zou ik dat niet weten.

**Interviewer:**	Je zegt net vooral operationeel…

Vooral strategisch, organisatorisch beleid. Dat ligt ook bij >BEDRIJFSLEIDER<.. Dat staat ook duidelijk in de taakfunctieomschrijving van >BEDRIJFSLEIDER<. Dat is duidelijk afgescheiden. Ik heb nou het idee dat het een en ander ook weer in kaart werd gebracht. Nou dan noemt >BEDRIJFSLEIDER< ook van 'wat doe jij en wat doe ik?'. En ook van 'nou het team moet toch wel duidelijk wanneer kunnen ze bij jou en wanneer kunnen ze bij mij'. En volgens mij hebben we dat wel redelijk helder gecommuniceerd. Voor het team is het denk ik wel duidelijk. Ik hoor ze er niet over. En als ze een vraag bij mij neerleggen waarvan ik denk 'nou, daar weet ik het antwoord niet op. Dat is niet mijn gebied', dan heb ik zoiets van 'loop even lang bij >BEDRIJFSLEIDER<'. Of als >BEDRIJFSLEIDER< er niet is, dan heb ik zoiets van 'wil je dat ik het meeneem?'.

*Telefoon gaat.*

**Interviewer:**	Zoals je net zei. Die 2.500 euro mag je inkopen. Mag je met die 2.500 euro ook iets voor het personeel doen. Dus in het personeel investeren?

**Respondent:**	Uh. Je bedoelt bijvoorbeeld scholing...

**Interviewer:**	Voor scholing vertelde je dat je een scholingsplan hebt.

**Respondent:**	.. of je bedoelt een cadeautje?

**Interviewer:**	Ja zoiets. Of zoiets als een vrije dag.

**Respondent:**	Oee.

**Interviewer:**	Ligt dat bij jou?

**Respondent:**	Nee.

**Interviewer:**	Ligt dat bij >BEDRIJFSLEIDER<?

**Respondent:**	Als het zou kunnen dat ik daarin meer zou kunnen beslissen, zou dat heel fijn zijn. Ook meer omdat je precies weet wat er reilt en zeilt.

**Interviewer:**	Heb je het idee dat dat nu het geval is?

**Respondent:**	Nee. Nee. Is het niet.

**Interviewer:**	Die 2.500 euro is voor jou meer zorg-gerelateerd?

**Respondent:**	Die 2.500 is meer voor inkoop. Alles wat er hier nodig is aan apperatuur. Zo heb ik net borstvoedingskussens besteld. Daar maak ik dan een bon van. En daar maak ik dan een beslissing over. Dit is echt verloskundig dus dit gebruiken wij. En soms als het ingewikkelde dingen zijn denk ik 'mwa, de dokters en de verloskundigen moeten er ook mee werken dan overleg ik het met twee andere partijen'. Tot dat bedrag kan ik wel het bonnetje tekenen.

**Interviewer:**	Je noemde net bijvoorbeeld dat bosje bloemen en dat kaartje. Doe jij dat dan ook of ligt dat bij de bedrijfsleider?

**Respondent:**	Ik meld het >BEDRIJSLEIDER<. Die en die zijn ziek. Langdurig ziek. En ik wil hen een bosje bloemen sturen.

**Interviewer:**	Dan heb je het wel zelf besloten? Dan zijn het de kleine dingetjes die jij eventueel wel zou kunnen doen?

**Respondent:**	Ja.

**Interviewer:**	Wat maakt naar jou idee een goede teamleider?

**Respondent:**	Ik denk dat je als teamleider niet alleen maar ook moet hebben voor de productie die er gedraaid wordt en dat de bedden vol zijn. Maar ook dat je gewoon naar je personeel kijkt. Dat je een luisterend oor bied. Dat je ze de tijd en ruimte bied en de mogelijkheden. Dat je ondersteunt. Je coacht. Eigenlijk moet je er voor ze zijn in de breedste zin van het woord. En weet je wat mijn werk hier inhoud, dat weten ze wel. Kijk, joh als je wil weten waar ik mee bezig ben, kom en vraag het. Ik probeer zo open mogelijk en zo transparant mogelijk te laten zien waar ik mee bezig ben. En als ik zoiets heb van 'dit moet het team weten', dan zeg ik dat ook. Zoals nu het nieuwe protocol is een mooi voorbeeld. Dan zeg ik ook van 'joh, hebben jullie nieuwe materialen nodig en dan mogen ze zelf aan de slag'. Dan gaan ze tellen en er zijn altijd wel mensen van 'oh, zal ik dit en dit doen?'. Ja. En dan complimenteer ik ze. En daar waar de serieuze gesprekken gevoerd moeten worden van 'denk eventjes aan'. Of het nou houding is of gedrag, inzet. Daar probeer ik op een hele tactvolle manier te benaderen. Ik zie het ook meer dat ik als teamleider alle schakels houd ik open. Ook alle deuren houd ik open voor iedereen. Want de artsen komen, de verloskundigen komen. Er zijn zo veel werkgroepen. Het is eigenlijk best veel in dat bedrijfje.

**Interviewer:**	Heb je ook nog het idee dat jij als teamleider bepaalde dingen moet weten of over bepaalde kennis moet beschikken om je werk naar behoren te kunnen doen?

**Respondent:**	Ja. Zeker.

**Interviewer:**	Wat moet je weten?

**Respondent:**	Ik vind dat je een stukje, vond ik prettig, personeelsmanagement. Dat vond ik prettig. Ik volg altijd alle workshops die er zijn. Ik denk dat je zeker weet hoe je moet weten hoe time management, verzuimmanagment, roosterplanning, hoe je verbeterplannen maakt. Dat soort dingen ik denk dat je over kwaliteit en veiligheid ook voldoende informatie moet hebben. Het hoeft niet dat je een opleiding gaat volgen, maar dat je gewoon cursussen volt of workshops volgt. Je kunt ook zelf een boek lezen. Ik heb zelf de managementopleiding gedaan en dat vind ik wel toegevoegde waarde hebben.

**Interviewer:**	Een externe managementopleiding of een interne opleiding?

**Respondent:**	Nee. Extern. Een tweeëneenhalf jarige opleiding heb ik gedaan.

**Interviewer:**	En voor dat stukje personeelsmanagement kennis, wat helpt jou. Wat voor kennis helpt jou?

**Respondent:**	Ik denk wel dat je weet hoe je omgaat met de mensen maar. Wat ik zelf mooi vind is dat je gedrag kunt herkennen. Dat je ook weet wanneer je ondersteuning moet aanvragen bij HR.

*Telefoon gaat.*

**Respondent:**	Dat je ondersteuning bij HR kan vragen. Binnenkort heb ik zo'n WIA IVA gesprek met een medewerker die ziek is. Dan denk ik 'ja, ik vind wel'. Je hoeft niet alle kennis te hebben, maar ik wil wel voorbereid zijn. Ik wil wel een stukje kennis hebben van 'goh waar gaat het over, wat houdt het in?'. Ja. Dat vind ik wel belangrijk.

**Interviewer:**	Zijn er dan ook nog bepaalde competenties die je zou moeten hebben als teamleider?

**Respondent:**	Dat is een breed scala hè?

**Interviewer:**	Wat helpt jou het meeste?

**Respondent:**	Ik denk je als teamleider ook wel open moet zijn. Je moet benaderbaar zijn. Mensen moeten je kunnen vinden. Ze moeten je in vertrouwen dingen kunnen mededelen. Kijk ook wel communicatie. Dat ik communicatief-vaardig ben. Dat is best lastig. Dan heb je bepaalde termen in je hoofd en dat gebruik je. En dan zegt iemand tegen je 'en nu in Jip en Janneke taal'. Overschakelen. Maar ook gewoon van die organisatie. Daar moet je ook kennis van hebben. Hoe ziet de organisatie eruit en hoe ziet het organogram eruit. Bij wie moet ik zijn? Netwerken. Noem het maar op. Heel breed vind ik zelf. Misschien wel breder dan. Soms denk ik wel eens breder dan dat pakket van mijn bedrijfsleider zelf omdat mijn bedrijfsleider echt een afgebakend iets heeft. Die zorgt voor het meerjarenbeleid, die zorgt voor dat en dat en dat, en  voor de begroting en ja het is dat. En ik heb het idee. Ik zit hier en het is tentakel dit tentakel dat. Tentakels alle kanten op.

**Interviewer:**	Heb je ook het idee dat een teamleider nog bepaald gedrag moet vertonen?

**Respondent:**	Ja. Zeker.

**Interviewer:**	Hoe gedraagt een teamleider zich?

**Respondent:**	Ik denk niet naast je schoenen lopen. Je moet het voorbeeld geven. Ik denk wel dat lachen en humor mag ook. Als het serieus is vind ik dat je ook serieuse gesprekken moet aangaan. Want anders ga je niet corrigeren. Dan kun je ook niet verwachten dat een andere ook. Ik probeer het altijd gelijkwaardig. Maar ja. Dat is natuurlijk een utopie om te denken dat iedereen dat ook voelt. Dat is niet zo. Ik vind wel dat je een voorbeeldfunctie hebt en dat je niet hier moet in het rond gaan schreeuwen en bleren en doen. Ik denk ook dat je eerlijk en oprecht moet zijn. Als je iets niet weet. Als mij iets wordt gevraagd en ik weet het niet, dan zeg ik ook van 'joh, ik weet het niet'. Moet ik het even voor je opzoeken? Of ik kom er op terug. Dat doe ik niet te vaak. Want dan kan het ook tegen je gebruikt worden. Over het algemeen.

**Interviewer:**	Heb je ook nog een kijk op hoe je als teamleider je taken moet uitvoeren?

**Respondent:**	Zelf?

**Interviewer:**	Heb je bijvoorbeeld een idee in je hoofd hoe je als teamleider moeten doen?

**Respondent:**	Eigen invulling. Iedereen is ook wel anders. Het is ook een stuk van jezelf. Ik vind het belangrijk dat ik weet bij wie ik kan aankloppen als ik iets wil weten of als iets onduidelijk is of als ik er de kennis niet voor heb. Dan heb ik zoiets van 'goh, help mij'. Dat vind ik wel belangrijk. En invulling van dingen. En als je in het ziekenhuis kijkt. We hebben regelmatig overleggen met teamleiders. Dat is gaandeweg wel wat minder want iedereen heeft het druk. Merk je ook wel dat we allemaal hetzelfde hebben. Het is druk het is vol. Je hebt geen tijd. Er ligt heel veel op je bordje. Wanneer doe je wat? En hoe doe je het? Wat doe jij? Hoe doe jij het? Oh, ik doe het zo en zo. Dat zijn zulke grote verschillen. Ik heb veel contact met de teamleider van >AFDELING<. We zitten nu ook samen in een project. Er zijn gewoon hele grote verschillen inherent aan de hoe de bedrijfsleider zelf de ruimte geeft om dingen te doen. Daarin met je gewoon dat het verschil. Vind ik zelf in dit ziekenhuis veelste groot.

**Interviewer:**	Mijn laatste vraag. Hoe ben jij op de hoogte van wat jouw functie als teamleider nou wel en niet inhoud? Wat je wel en niet doet. Hoe is dat voor jou duidelijk geworden?

**Respondent:**	Een aantal jaar. In 2008. Ik was eerst een Senior en daarna, volgens mij 2009, werd het Senior Zorgeenheid omdat de Raad van Bestuur het woord teamleider niet wilde. In 2012, nee 2013, werd het echt teamleider. Ja. Weet je hoe haal je informatie. Ja, nee. We hebben gewoon een taakfunctieomschrijving gehad en toen weet ik nog wel dat ik heel brutaal was om bij HR te vragen van 'goh, wat houdt het nou in'? En we hadden toen een interim-bedrijfsleider en ik heb toen gevraagd 'ik wil zien wat er in die taakfunctieomschrijving staat'. Heb ik daar nog wel een stem in? En toen heeft die het naar mij toegestuurd en zo.

**Interviewer:**	Dus heel sterk aan die functieomschrijving vasthouden?

**Respondent:**	Ja. En dat was jammer hoor. Want ik was echt van mening hoor. Dan solliciteer je en dat word je aangenomen. En als je profiel nog niet helemaal, het was toen een facet, dat je samen met je mensen kunt vragen van 'goh, wat is je verwachting?'. Hoe denk je dat in te gaan vullen? Wat is je beeld?

**Interviewer:**	Het is nu op je bord gegooid?

**Respondent:**	Ja. Hier. Hup. En dan ga je gaandeweg daar een weg in vinden van 'goh, hoe doe ik dit, hoe doe ik dat?'. En dan kom je er dan achter van 'oké, zo doe ik dit. Maar het is zoveel'. En hoe?

**Interviewer:**	Was dat niet heel stressvol?

**Respondent:**	Ik heb niet zoveel last van stress, maar ik...

**Interviewer:**	Lastig misschien?

**Respondent:**	Ja. Lastig wel. En toen ben ik het lijstje gaan maken. Ik heb eerder een gesprek gehad met iemand van HR die vroeg 'wat doe je zoal, kun je dat puntsgewijs op papier zetten?'. Toen heb ik dus dit lijstje gemaakt met wat ik allemaal doe en die heeft het toen vergeleken met wat andere teamleiders van de algemene afdelingen deden. Dit was echt drie keer zoveel als wat de andere deden. En dan is uitgegroeid. Dat nu ook de anderen zeggen 'ik verzuip'. Ik werk 36 uur. Maar ik denk dat ik uiteindelijk wel 50 uur per week werk. Want ja. Er moet ook nog een planning gemaakt worden en als er ziekte is dan verandert de planning. En ik krijg hier de rust niet, mijn deur staat dan nu op bezet dus dan komen ze niet, om hier het rooster te maken. Dan doe ik dat thuis.

**Interviewer:**	Zou er dan uiteindelijk meer naar de seniors geschoven moeten worden?

**Respondent:**	Dit hebben ook veel hoor?

**Interviewer:**	Wat maakt voor jou dan een goede oplossing?

**Respondent:**	Dat heb ik ook nu bedacht. Omdat >BEDRIJFSLEIDER< ook bedrijfsleider wordt van >EVR<. Dat is best wel veel.

*Telefoon gaat.*

**Respondent:**	Dan denk ik wel het moet voor mij nog duidelijker zijn 'wat gaat >BEDRIJFSLEIDER< doen? Ik ben van mening, dat is namelijk ook een best grote EVR, hoe ga jij dat doen? Maar ik denk wel, ik ben namelijk ook zo, ga iets maar gewoon proberen. Als je het niet hebt geprobeerd, dan weet je het niet. Ik denk wel van goede communicatie nodig is om verder te gaan.

**Interviewer:**	Dus goed weten vanuit >BEDRIJFSLEIDER< van 'wat verwacht ik nou van jou?'.

**Respondent:**	Dus ik heb wel een opzetje gemaakt van het profiel. Maar als ik dan zie wat er naast het rijtje ook erbij staat. Ik zit ook in ziekenhuisbrede projecten. Per 1 mei gaan we ook QMENTUM uitrollen voor de hele EVR. Dan zegt >BEDRIJFSLEIDER< ook van. Dat is echt wel een kluif. Daar moet ik dan echt goed over nadenken. Het is een groot project. Ik ben van mening dat ik best goed voor ogen heb. De senior heeft een groot takenpakket. Maar je hebt ook mensen die hun eigen taak en aandachtsgebieden hebben. Die wil ik ook in hun kracht laten. Als ik maar de terugkoppeling heb van 'dat heb je nodig om je verbeterpunten, je plannen, bij te stellen'. En recent heb ik bij >COLLEGA< van Kwaliteit en Veilig gevraagd of zij wat lijstjes van PDCA kunnen sturen. Hoe ze PDCA in kunnen zetten. Dan hoe ik daar niet meer achteraan te hobbelen.

**Interviewer:**	Dat je dan ook anderen benadert van 'kun je dat voor mij regelen'?

**Respondent:**	Ja. We hebben echt wel een sterk team. En er zitten ook heel veel kwaliteiten in van mensen die het zelf kunnen alleen ze hebben behoefte aan tijd. Als ik ze dan ook vraag 'wat heb je nodig om hier die toko te draaien'. Ik ga kijken wat ik nodig en ik kom erachter dat als het team echt in hun kracht wil ik ze laten en ruimte wil geven dan heb ik tijd nodig. Als ze acht uur werken gaan ze niet nog twee uur verbeterplannen of een analyse maken. Dus dat zit hem in tijd. Zelf zijn we een van de afdelingen die goed presteren en goed hun zaken op orde hebben. En dat durf ik te zeggen omdat we niets doen, maar juist omdat we hard werken. Dus nee. >BEDRIJFSLEIDER< zei ook van. Ik ga wel met >BEDRIJFSLEIDER< afspreken van 'hoe lang ga ik dat aankijken?'. Even een datum inplannen. Op die tijd evalueren wij en kan ik nog bijstellen wat ik nodig heb om niet te verzuipen.

**Interviewer:**	Over dat functieprofiel van teamleider is dan veelste basaal wat jou betreft? Moet die meer op maat gemaakt moeten worden?

**Respondent:**	Ja. Dat denk ik zeker. We hebben de profielen naast elkaar gezet. Teamleider algemeen. Mijn profiel. En dan de seniors. De senior is niet echt een functie, het is meer een rol. Ik zie wel heel grote verschillen. En eigenlijk zou je hem dus op maat moeten maken en dat is wat we nu een beetje aan het doen zijn. Maar wel met de mogelijkheid om nog te zeggen van 'we moeten een beetje bijsturen, want het wordt nu een beetje veel'. Het is ook inherent aan 'hoe sta je er zelf in'. Ik vind mijn werk leuk en put daar veel energie uit. Ik steek er veel energie in, maar krijg er ook veel energie voor terug. Ik zoek wel. En netwerk. En zoek informatie op. En als er scholing is, dan vol ik die. Want dan weet ik dat ik voldoende bagage heb om het hier draaiende te houden.

**Interviewer:**	Duidelijk. Ik weet niet of je nog zelf dingen aan het interview wil toevoegen?

**Respondent:**	Nee. Wat ik net noem. Ik vind dat het op maat moet. En ben benieuwd hoe de tendens zal gaan als bedrijfsleiders meerdere EVRs zullen aansturen. Ik denk dat het het begin is. Dan...

**Interviewer:**	Wat bedoel je daar precies mee?

**Respondent:**	Ik denk dat je dan echt moet gaan kijken. Kijk het kan zo zijn. Dit is Verloskunde en echt een profiel op maat. Dat is het maar de vraag of ik mij daar in kan vinden. Of dat ook niet te veel is voor het aantal uren dat je moet werken. Hoewel. De ene teamleider werk 24 de ander 32 de ander 36. Dus daar. Ook daar zitten zulke grote verschillen. Dat ik me soms ook wel denk 'oh, jij ben ook. Lekker relaxt baantje'. Heb ik echt wel gezegd. Maar dit is mijn vakgebied. Daar heb ik iets mee. Dus dan blijf je. Ik vind ook wel dat je als teamleider vakinhoudelijk wat moet weten. Je kunt niet een teamleider MDL op Verloskunde laten werken. Dat is eigenlijk not done. Kijk >BEDRIJFSLEIDER< is ook geen verloskundige of verpleegkundige. Dus als >BEDRIJFSLEIDER< inhoudelijke dingen krijgt, worden die naar mij gestuurd van 'oh, wil jij even meelezen?' of 'ik weet het niet'. Dus ja. Dat is net zo goed als dat ik iets heb om met >BEDRIJFSLEIDER< te kortsluiten. Dan ze ik ook 'ik ga liever naar de verloskundige als naar >BEDRIJFSLEIDER<'.

*Telefoon gaat.*

**Respondent:**	Dus ja. Ik ben heel benieuwd naar de uitkomsten van jou en wat de verschillen zijn. Want ik ervaar een heel groot verschil in het ziekenhuis. De teamleiders van de Poli. Ik zou het niet weten wat zij allemaal in hun pakket hebben. Ik zou het niet weten. Ik weet het niet. Dat komt ook omdat je weinig contact hebt. Ik heb meer contact met de teamleiders van Kliniek. Er is toen geopperd van 'misschien is het handig om bijeenkomsten met elkaar te hebben. Dus teamleiders poli, kliniek, iedereen bij elkaar. Ja. Wat kom je halen, wat kom je brengen dan hè? Ik merkte wel dat ik de dingen die ik van de poli hoorde. Dat waren allemaal dingen ja dat wist ik. Dat waren de basis dingen. Ook inherent aan hoe lang ben je teamleider? Heb je een opleiding gevolgd?

**Interviewer:**	Dus die bijeenkomsten hadden niet echt een meerwaarde voor jou?

**Respondent:**	Nee. Mijn advies naar degene die dat organiseert en plande was dan ook 'kun je geen onderscheid maken tussen teamleiders kliniek en teamleiders poli?'. Ik denk dat zij hun vakjargon is hetzelfde. En teamleiders kliniek is om en nabij hetzelfde.

**Interviewer:**	Een soort clustering als het ware?

**Respondent:**	Ja. Waar ik meer behoefte aan had was zeg maar een intervisie. Dat je casuïstiek kunt inbrengen en van elkaar kan leren.

**Interviewer:**	Maar het niveauverschil is te hoog het werkt niet...

**Respondent:**	Dan kan het niet.

**Interviewer:**	De ene vindt het te moeilijk en de ander weer te makkelijk?

**Respondent:**	Ja. Klopt. Dus dat voorstel heb ik ook gedaan van 'misschien moeten we het uit elkaar trekken poli en kliniek' en dan intervisie voor de poli en intervisie voor de kliniek. De bedrijfsleider hebben ook intervisie voor de bedrijfsleiders. Waarom hebben wij dat niet? En dat is ook. Ik zie intervisie niet alleen als leren van elkaar dat je bepaalde situaties met elkaar bespreekt. Maar dat je ook een stukje kennis met elkaar kan delen. Ik merk ook. Ik denk ook dat het komt omdat iedereen druk is als er een scholing is of zo. Dan zitten er maar weinig mensen. Dat ik denk 'hoe kan dat nou?'. De afgelopen week heb ik een workshop over mantelzorg gevolgd. Ik vond dat erg informatief. Nu heb ik een paar handvatten. Die kan ik ook inzetten want ik weet dat er twee medewerkers zijn die echt mantelzorg verlenen. Dan weet je echt hoe je er mee om moet gaan. Dan denk ik 'ja, waarom is het niet een soort verplichting dat elke teamleider dat volgt?'.

**Interviewer:**	Dat lijkt me een moeilijke afweging. Dan wordt het weer een moetje en dan roep je weer weerstand op?

**Respondent:**	Dan kom ik weer terug op jouw vraag over personeelsmanagement. Dat is ook een onderdeel van. Dat je weet hoe je daar op kunt sturen als iemand zegt van 'goh, joh mijn kind is ernstig ziek'. Dat is ook een stukje.

**Interviewer:**	Zou het wat dat aangaat allemaal wat strakker geregeld moeten worden? Dat als er dan een teamleideroverleg komt dat je dan ook echt zegt 'dit is onderdeel van je functie, hier moet je bij aanwezig zijn?'.

**Respondent:**	Ja. Dat zou je dan echt moeten benoemen van 'dit is een verplicht item'. Het heeft een verplicht karakter voor de teamleiders. Want het is nu een beetje vrijblijvend en vrijblijvendheid. Kijk, als je gedisciplineerd bent, dan doe je dat. Als ik vrij ben en er is een workshop, dan denk ik 'daar kan ik wat van opsteken, dat kan ik toepassen', dan kom ik. Dan is het voor mij niet te veel. Maar het is ook een stukje mentaliteit. Dat is dan gewoon zo. En hoe doe je je werk? Ja. Met liefde en passie. Dat is het.

**Interviewer:**	Ontzettend bedankt dat je mij meegenomen hebt in jouw functie als teamleider.

**Respondent:**	Oh, dat was ik nog vergeten te zeggen. Ik ervaar een soort hiërarchie of bureaucratie. Als je als teamleiders wat voor elkaar wil krijgen dat je onderling niet krijgt, dan moet er eerst een bedrijfsleider overheen voordat het lukt. Dan moet je de teamleider overtuigen. Dat moet je voorzichtig benaderen. Soms is niet duidelijk en soms wel of de bedrijfsleider daarin met je meegaat. Dan ben ik wel eens verrast. Soms dien ik het gewoon in en wacht ik af.

*Toevoegingen respondent per mail:*

ik vind dat er qua salaris onderscheid gemaakt mag worden tussen teamleiders met een afgerond management opleiding en zonder Opleiding. Overgroot deel heeft een cursus gevolgd van 6 dagen. Omdat ik tot de eerste groep teamleiders behoorde heb ik toen bewust de cursus naast mijn management opleiding ook gevolgd. Vond het onvoldoende qua leerdoelen en inhoud etc. Daarnaast is het zo dat gespecialiseerd verpleegkundigen die teamleider zijn geen schaal verhoging hebben. Mijn medewerkers die 32 uur werken verdienen dus meer dan ik. Hoe krom is dat. Ziekenhuisbreed zit in salaris en taken/ profiel een enorm verschil. Ik zou nog meer willen bespreken.

Er is nog 1 punt wat mij bezig houdt: er staat aangegeven dat de teamlieder operationeel leidinggeeft en niet hiërarchisch.

Ik vraag mij af of dit wel strookt met het voeren van jaargesprekken, medewerkers aanspreken op gedrag, verzuimgesprekken voeren, coaching etc.

Misschien een vraag om mee te nemen in je verslag?

Teamleiders met een management opleiding zouden een addendum moeten hebben en functieschaal gewogen. Hebben een groter taken pakket en meer verantwoordelijkheid.

Heel veel succes

Ik ben erg benieuwd naar de uitkomsten en of het zal zorgen voor helderheid en eenduidigheid in het >ZIEKENHUIS<.

*Einde transcript*

# Transcript Interview FLM 7

**Duur interview:	43 minuten en 30 seconden**

**Functie:	Teamleider**

**Afdeling(en) onder supervisie:	Healthcare**

**Gender:	vrouw**

**Werkervaring in functie:	1.8**

**Interviewer:**	Misschien kunnen we beginnen met jouw functie naast die van coördinator te leggen. >COÖRDINATOR< is coördinator voor de >AFDELING<. Wat maakt het verschil tussen jou en >COÖRDINATOR< als het aankomt op werkzaamheden?

**Respondent:**	>COÖRDINATOR< is daarin bezig met de direct aansturing van mensen en met de planning, maar ook de planning verwerken in DRP, dat doet >COÖRDINATOR< ook. En ik ben meer een stapje hoger, of hoe zeg je dat? Ik heb bepaalde ideeën over hoe dingen zouden moeten gaan dat bespreek ik dan met ze en dat proberen door te voeren.

**Interviewer:**	Is het dan ook zo dat als er iets op de werkvloer gebeurt dat ze dan eerst naar >COÖRDINATOR< gaan voordat ze naar jou komen?

**Respondent:**	In principe wel ja. >COÖRDINATOR< is gewoon het direct aanspreekpunt voor medewerkers. >COÖRDINATOR< loopt in de ochtend een rondje en >COÖRDINATOR< ontvangt ook mensen en >COÖRDINATOR< geeft aan waar mensen naartoe moeten en stuurt bij op kwaliteit of als er iets op die afdeling wat >COÖRDINATOR< via de mail heeft gekregen dan is >COÖRDINATOR< degene die dat tegen medewerkers zegt.

**Interviewer:**	En doet >COÖRDINATOR< dat fulltime?

**Respondent:**	Nee. Tweeëneenhalve dag in week. 21 uur.

**Interviewer:**	Voor de rest, mocht >COÖRDINATOR< er niet zijn?

**Respondent:**	Ja, ook, kan. Dan hebben we een medewerker kwaliteit. Een kwaliteitsmedewerker en die is er op de andere dagen dat >COÖRDINATOR< er niet is en >MEDEWERKER KWALITEIT< heeft dan ook die aansturende werkzaamheden en de aandachtsgebieden van >MEDEWERKER KWALITEIT< is kwaliteit.

**Interviewer:**	Dan nu naar jou als teamleider. Waar houd jij je mee bezig?

**Respondent:**	Ik houd mij bezig met alles wat niet via >COÖRDINATOR< en >MEDEWERKER KWALITEIT<, direct die operationele zaken. Die sluis ik of wel door naar >COÖRDINATOR< of naar >MEDEWERKER KWALITEIT< of naar >AFDELING<. Maar als het voor lange termijn is en structureel, dan soort zaken neem ik op, pak ik op.

**Interviewer:**	Maar dan nog wel steeds gefocust op de werkvloer, die operationele werkvloer.

**Respondent:**	Ja. Ja. Ja.

**Interviewer:**	Herken je ook dat er een stukje personeelsmanagement in jouw functie van teamleider zit?

**Respondent:**	Ja. Zeker. Ik doe dus wel de jaargesprekken met mensen en daar komt natuurlijk ook een stukje personeelsmanagement bij. En als >COÖRDINATOR< en >MEDEWERKER KWALITEIT< er niet uitkomen met de medewerkers, dan pak ik het op. Een bepaalde manier van samenwerken of als ze ergens ontevreden over zijn dan hebben we het gesprek met z'n drietjes en spreken we daar de medewerker op aan of proberen we daar op een ander manier uit te komen met elkaar. Ja.

**Interviewer:**	Ik heb hier wat gevonden wat misschien bij een teamleider zou kunnen liggen aan onderwerpen. Zijn er bepaalde onderwerpen die naar jouw idee eruit springen van 'hier ben ik mee bezig'? En zo ja, op wat voor een manier?

**Respondent:**	Ja. Werving en selectie. Introductie...

**Interviewer:**	Voor werving en selectie, voer jij dan zelf de gesprekken?

**Respondent:**	Ik wil ze wel zien. Kan zijn dat >COÖRDINATOR< of >MEDEWERKER KWALITEIT< het eerste gesprek voert en ik daarna dan nog een gesprek heb.

**Interviewer:**	Oké. Duidelijk.

**Respondent:**	Bijhouden van personeelsbestanden. Dat doe ik ook. Beslissen over en plannen van trainings en ontwikkelingsbehoeften medewerkers. Ik heb contact met de afdeling Opleidingen.

**Interviewer:**	Jij bent dan degene die het opleidingsplan maakt?

**Respondent:**	Ja. En daar heb ik dan een budget voor dan beslissen we daar dan over samen met Opleidingen. Dat er in ieder geval een budget komt en dat we dit jaar zoveel opleidingen gaan doen. Geven van formele training. Wat bedoel je daarmee?

**Interviewer:**	Sta je ook wel een voor de groep om instructies te geven of nieuwe dingen uit te leggen?

**Respondent:**	Daar hebben we dan >MEDEWERKER KWALITEIT< van kwaliteit. >MEDEWERKER KWALITEIT< doet ook dat stukje van als mensen een bepaalde methodiek niet goed kunnen dan helpt >MEDEWERKER KWALITEIT< daarmee. Dat is de rol van >MEDEWERKER KWALITEIT<. Geven van informele training inclusief coaching, dat is meer wat ik doe.

**Interviewer:**	Op wat voor manier doe je dat?

**Respondent:**	Soms heb je wel een mensen privé wel eens wat zaken in een groep waar zich nog wel eens wat kan afspelen thuis of in hun omgeving. En dat ze dat niet altijd bij >COÖRDINATOR< of >MEDEWERKER KWALITEIT< kwijt kunnen, dan komt het bij mij.

**Interviewer:**	Enig idee waarom ze het daar niet kwijt kunnen, maar wel bij jou?

**Respondent:**	Omdat. Misschien. Ik ben net. Uhm. Ja, weet ik wel. Dan denk ik, dat het daar niet altijd even veilig is.

**Interviewer:**	Dan denk ik hardop hoor, maar is dat dan omdat >COÖRDINATOR< of >MEDEWERKER KWALITEIT< dichter bij de werkvloer staan dan dat jij staat?

**Respondent:**	Ja. Ja. En dat het dan misschien besproken wordt met andere mensen.

**Interviewer:**	Dus dit is dan een soort veilige afgezonderde...

**Respondent:**	Ja. Ja. Ja.

**Interviewer:**	Mentoren die zit er dan ook een beetje in. Ben je ook wel eens op de werkvloer echt mensen aan het aansturen van 'misschien kun je het beter zo doen of beter zo doen?'.

**Respondent:**	Nee. Nee. Nee.

**Interviewer:**	Dus je bent voornamelijk hier.

**Respondent:**	Ja. Hier en eigenlijk de contactpersoon naar de >AFDELING< of andere afdelingen van stafafdelingen.

**Interviewer:**	Duidelijk. Functioneringsgesprekken en beoordelingsgesprekken.

**Respondent:**	Dat doe ik allemaal zelf.

**Interviewer:**	Allemaal? Grote ploeg?

**Respondent:**	Ja. Moet ik allemaal zelf doen. En uitwerken ook.

**Interviewer:**	Ontwikkelingsplannen. Daar heb je ook al wat over gezegd. Ben jij uiteindelijk degene die bepaalt. Jij maakt het plan maar geef jij ook akkoord op het plan van 'we gaan het doorvoeren ja of nee?', of wordt dat gedaan door >HOOFD< of iemand anders?

**Respondent:**	Kijk. Daarin heb ik wel veel samenspraak met >HOOFD<. Dat is ook de manier dat we samen van 'nou' en >HOOFD< kan er een klap op geven of >MANAGER< geeft er een klap op. Even een voorbeeld. Ik heb gevraagd, ik wil deze ruimte hiernaast opheffen, de linnenkamer en daar wil ik een soort pauzeruimte van maken voor de medewerkers hier. Ze moeten allemaal in het kantoortje of ze moeten ergens. Dat is niet helemaal het beleid van het ziekenhuis, maar ik vind wel, deze groep heeft daar wel behoefte aan om gewoon dat ze hier komen en rustig kunnen zitten.

**Interviewer:**	Een eigen plek?

**Respondent:**	Een eigen plek ja. Dat is niet het beleid van het ziekenhuis, want die vinden dan dat de mensen die op de >AFDELING< werken dat ze daar ook pauze houden of bij >RESTAURANT< of in de banken. Dus ja, ik zou daar zo akkoord op geven, maar >HOOFD< en >MANAGER< zijn ook van 'oh, dat moeten we even in de gaten houden'.

**Interviewer:**	Als het aankomt op het disciplineren en corrigeren van medewerkers, is dat jouw rol?

**Respondent:**	Dat is mijn rol. Ik ben die. Ja. Het minder leuke ja. Het aanspreken op gedrag. Of als ze werkzaamheden niet uitvoeren. >COÖRDINATOR< of >MEDEWERKER KWALITEIT< in eerste instantie, maar als dat een probleem dreigt te worden, dan ga ik daarin en. Ja.

**Interviewer:**	Heb je ook het idee dat medewerkers het zo zien dat jij een stapje hoger bent in de hiërarchie?

**Respondent:**	Ja. Dat denk ik wel.

**Interviewer:**	En dat heeft meer impact?

**Respondent:**	Ja.

**Interviewer:**	Misschien iets arbeidsrechtelijke confl>AFDELING<en.

**Respondent:**	Ja, heb ik ook gehad.

**Interviewer:**	Dat ligt ook bij jou?

**Respondent:**	Dat hebben >HR ADVISEUR< en ik hebben daar nog een dingetje over gehad met twee medewerkers. Dat is nog net niet voor de rechter gekomen.

**Interviewer:**	Dat is iets want onder jou, door jou gedaan wordt?

**Respondent:**	Ja.

**Interviewer:**	Managen van absenties?

**Respondent:**	Ik houd ook de absenties bij en zorg er bijvoorbeeld ook voor dat mensen re-integreren en dat soort zaken.

**Interviewer:**	Dat jij de gesprekken voert?

**Respondent:**	Ja. Net als de meneer die nu beneden bij de toiletten zit, is je dat opgevallen? Daar staat nu een meneer bij de toiletten beneden in de hal.

**Interviewer:**	Nee.

**Respondent:**	Moet je dadelijk maar eens even kijken.

**Interviewer:**	Oké.

**Respondent:**	Die is ziek, maar die kan wel kleine stukjes lopen. Die staat nu bij de toiletten. En in eerste instantie is het natuurlijk van 'dat ga ik echt niet doen'. Je bent natuurlijk verplicht om mee te helpen, mee te re-integreren. En nu vinden ze het volgens mij wel leuk.

**Interviewer:**	Mooie ontwikkeling. Waardering geven aan medewerkers?

**Respondent:**	Ja. Ook.

**Interviewer:**	Op wat voor een manier?

**Respondent:**	Net als bijvoorbeeld. We hebben noro gehad hier. Heb je dat meegekregen?

**Interviewer:**	Ja.

**Respondent:**	Nou, daar hebben de mensen natuurlijk best veel mee te maken gehad. En dan zeg ik in de ochtend of in de middag, of geef ze van 'goh, dat hebben jullie wel goed voor elkaar gekregen'. Of in een werkoverleg. Of het ziekteverzuim dat lager is. Het is niet heel laag, maar goed. Dat hebben we vorig jaar wel een paar keer gehad. Dat je mensen daar ook voor waardeert.

**Interviewer:**	Benoemen en een compliment geven?

**Respondent:**	Ja. Ja. Ja. Ik ben daar niet heel sterk in. Moet ik persoonlijk zeggen.

**Interviewer:**	Mag je ook beslissen over salarisschalen van medewerkers?

**Respondent:**	Nee. Want dat ligt allemaal vast. Dat is gewoon. Dat staat in de CAO en daar kan ik niets anders over zeggen. Ga ik ook niet doen want het is, het ligt gewoon vast.

**Interviewer:**	Oké. Als het aankomt op communicatie...

**Respondent:**	Naar boven toe. Ja.

**Respondent:**	En zowel naar beneden toe. Zie jij. Is dat iets dat bij jou ligt?

**Interviewer:**	Ja. Juist ik ben degene die naar de. Ja.

**Respondent:**	En wat voor manieren moet ik dan aan denken dat je dan boodschappen doorgeeft, van beneden naar boven of van boven naar beneden? Wat is jou manier?

**Interviewer:**	Ik probeer het via werkoverleg. Ik heb sinds dit jaar ook een nieuwsbrief en daar wil ik ook wat meer mee doen. Juist ook de leuke dingen gaan vermelden. Vaak is het zo als je niet goed oplet dat je alleen maar de negatieve informatie geeft. Ik vind dat je met zo'n nieuwsbrief ook leuke dingen moet vermelden.

**Interviewer:**	En die stuur je dan per mail toe?

**Respondent:**	Ja. Die is per mail. Omdat ik ook wil dat mensen, en dat is juist met deze groep heel moeilijk, veel meer ook achter de computer gaan zitten. Dat doet deze groep niet. Die vinden dat eng. En elke keer zijn ze hun wachtwoord kwijt of. Dan moet dat weer geregeld worden. Door zo'n nieuwsbrief, er is weer een nieuwsbrief. Kijk in je mail. In je mail komt ook gewoon allemaal andere informatie vanuit het ziekenhuis. Dus ja.

**Interviewer:**	Fysiek ophangen zou wat dat aangaat dat niet bevorderen?

**Respondent:**	Nee. Want dan faciliteer je ze weer. Ze moeten het ook zelf gaan halen.

**Interviewer:**	Oké. Duidelijk. Je pakt dat werkoverleg ook als een moment om te peilen wat er in de groep speelt zodat je dat eventueel weer door kunt spelen richting >HOOFD<?

**Respondent:**	Ja. Ja. Ja.

**Interviewer:**	Hebben jullie vaak overleg?

**Respondent:**	We hadden eerst acht keer per jaar. Dat heb ik nu teruggezet naar zes keer per jaar omdat ik vorig jaar een sessie heb gedaan Verbindende communicatie, daar heeft iedereen een cursus voor gevolgd. Ik weet niet of je dat meegekregen hebt?

**Interviewer:**	Wie is iedereen?

**Respondent:**	Iedereen van het ziekenhuis. Je hebt dan niet gehoord?

**Interviewer:**	Nee.

**Respondent:**	Dat was heel leuk. Het is een workshop Verbindend communiceren, geweldloos communiceren noemen ze het ook wel eens, dat is een hele dag. Drie dagdelen. Vonden ze allemaal best wel een ding. Vervolgens heb ik in kleine groepjes, met een cursusleider erbij, weer met elkaar gaat zitten en gezegd 'spreek elkaar eens aan wat je leuk vindt aan diegene, maar ook waar je je misschien aan stoort'. En dat vond ik eigenlijk veel meer waarde hebben dan zo'n werkoverleg waar iedereen aan het zitten is en denkt 'dat is mooi, een uur pauze'. Dat moeten ze zelf aan de slag...

**Interviewer:**	Een actieve werkvorm?

**Respondent:**	Ja. Ja. Dus dat wil ik daarom in plaats van, twee geschrapt, twee werkoverleggen. En dan doen we in het najaar weer een sessie Verbindend communiceren, weer met een klein groepje, gaan we weer eens met elkaar aan de slag.

**Interviewer:**	Oké. Duidelijk.

**Respondent:**	Dat is wat hier heel erg speelt. Ze vinden alles over elkaar en ze vinden het heel moeilijk elkaar daarover aan te spreken. Ik hoor elke keer weer 'ik hoor die die en die doet dit en die doet dit niet'. En heb je dat ook gezegd? Nee, dat doe ik niet, dat moet jij doen. Ik ga dat niet meer doen.

**Interviewer:**	Ligt daar een rol voor jou om dat...

**Respondent:**	Nee. Dat vind ik niet. Ik vind juist dat je elkaar moet aanspreken.

**Interviewer:**	Zie jij dan. Nou. Je noemt dan de cursus, dat moment geweldloos communiceren, zie je dat echt als een moment om ze daarmee te helpen?

**Respondent:**	Ja.

**Interviewer:**	Doe je dat ook nog op andere manieren of is dat gewoon het moment dat je met elkaar kunt spreken?

**Respondent:**	Hoe bedoel je dat?

**Interviewer:**	Nou zeg maar, als je door het werk heen misschien nog mensen helpt of een soort van coacht, dat ze daarmee werken en daarmee bezig gaan. Als ze bijvoorbeeld moeten wachten tot die sessie tot het uitgesproken hebben...

**Respondent:**	Nee. Dat komt inderdaad. Dat popt wel op. Als mensen. Dan komen ze ook zegen van joh 'dit en dit'. Ze hebben hier allemaal een eigen afdeling en dan hebben ze invallers, maar die doen schijnbaar niet alles en daar vinden ze dan wat van. Spreek mensen daar dan op aan.

**Interviewer:**	Dan geef jij het terug?

**Respondent:**	Ik probeer het wel steeds meer terug te geven ja.

**Interviewer:**	Je zei het net zelf ook al, je reageert op suggesties van het personeel. Ben jij, nee >COÖRDINATOR< is degene die alles regelt met de teams en de ploegen als het aankomt op 'wie werkt wanneer'?

**Respondent:**	Ja.

**Interviewer:**	Is >COÖRDINATOR< degene die ervoor zorgt dat alles in het team goed loopt qua teamwerk en dat alles goed verloopt?

**Respondent:**	Daar heb ik ook een rol in.

**Interviewer:**	Op wat voor een manier?

**Respondent:**	Op budgettair natuurlijk. Ik ben hier ook binnen gekomen een paar jaar geleden en er moest toen van alles bezuinigd worden. Dus ik heb toen samen met >COÖRDINATOR< en >MEDEWERKER KWALITEIT< gekeken van 'hoe kunnen we het efficiënter inzetten?' en toen hebben we een bepaalde verandering doorgevoerd. Nu zijn we daar met >HOOFD< weer mee bezig, dat wordt wel grootser aangepakt.

**Interviewer:**	Dus jij bent heel procesmatig aan het kijken 'waar het nu beter'?

**Respondent:**	Ja. Waar is nu. Die combinatie die ze nu willen is een roomservicemedewerker. Ik weet niet of je daar iets van gehoord hebt?

**Interviewer:**	Nee.

**Respondent:**	Dat is een combinatie van voedingmedewerkers, de voedingsassistenten, die bijvoorbeeld bij >NAAM< zitten, die komen bij de patiënt. Onze >AFDELING<medewerkers komen bij de patiënt, de afdelingsassistent vult nog kasten bij. Zo zijn er drie vier mensen die allemaal rondom de patiënt bezig zijn allemaal facilitaire taken. Dat zou je ook mooi samen kunnen brengen met dat het één persoon is.

**Interviewer:**	Dus één iemand met een heel breed profiel die alle losse taken zou kunnen doen?

**Respondent:**	Ja. Ja. Ja. Dan heeft de patiënt één gezicht. En daar kun je een efficiëntieslag mee maken anders heb je drie vier mensen die telkens naar de patiënt moeten lopen.

**Interviewer:**	Stel het wordt nou doorgevoerd, wat wordt dan van jou gevraagd als teamleider?

**Respondent:**	Nu gaan we kijken van 'welke medewerkers zijn daar mogelijk geschikt voor'. Want binnen mijn team zijn wel een aantal die zoiets hebben van 'nee, ik wil niet veel met de patiënt te maken hebben, ik wil liever niet iets vragen aan de patiënt, ik blijf liever op de achtergrond'. Dus daar moet je dus gaan kijken 'wie is daar geschikt voor, voor wie kunnen we laten opleiden, en wie gaan die productiewerkzaamheden doen?'.

**Interviewer:**	Heb je het idee dat je goed op de hoogte bent van iedere medewerker hoe die staat qua of je zoiets kan vragen van zo iemand.

**Respondent:**	Ja. Dat komt ook in de jaargesprekken en ook, ik zit ook in de pauzes bij de opstart, dan spreek je mensen ook. Die ken ik. Je hoort het natuurlijk ook van >COÖRDINATOR< en >MEDEWERKER KWALITEIT< hoe bepaalde mensen reageren en of ze proactief zijn of minder.

**Interviewer:**	Denk je ook dat onderwerpen als gezondheid en veiligheid en het verbeteren van werklevens bij jou ligt? Echt duurzame inzetbaarheid...

**Respondent:**	Ja. Want juist deze groep is ook inderdaad wat ouder. Ik heb best een grote groep 55 plus en richting 55 plus. Daar zijn we ook mee bezig.

**Interviewer:**	Op wat voor manier ben je daar dan mee bezig?

**Respondent:**	Bedden schoonmaken is best een intensieve bezigheid. Er zijn gesprekken geweest met een bedrijf dat robots kan leveren die bij het schoonmaken, VMARK van het Erasmus ziekenhuis, dat is het eerste ziekenhuis dat een soort beddenwasstraat heeft waar bedden erin gaan en worden daar robots schoongemaakt. Dus daar komt geen mensen arbeid meer aan te pas. Wel het opmaken van de bedden.

**Interviewer:**	Dat zou dan de medewerker van zware arbeid ontlasten?

**Respondent:**	Ja.

**Interviewer:**	Dat ze minder zwaar werk hoeven te doen?

**Respondent:**	Alleen onze bedden zijn er niet voor geschikt.

**Interviewer:**	Oh, dat is balen.

**Respondent:**	Ja. Dat is een beetje jammer.

**Interviewer:**	Dan maar de tuinslang erop zetten?

**Respondent:**	Ja. Daar zijn we ook mee in gesprek van...

**Interviewer:**	Dus echt kijken van 'hoe kunnen we het zware werk verlichten'?

**Respondent:**	Er is al een slag gemaakt een aantal jaar geleden. Ik denk een kleine tien jaar geleden. Toen. Weet je ongeveer hoe ze schoonmaken hier?

**Interviewer:**	Nee niet heel concreet.

**Respondent:**	Als je thuis dan een natte doek zou pakken en die uit zou wringen, dat doen ze hier niet meer. Het is allemaal microvezeldoekjes. Er wordt ook niet meer met sop schoongemaakt. Deze doekjes maak je nat, maar ook niet te nat, je hoeft ze niet wringen. Door het systeem van microvezel hoe je die doekjes te pakken en kun je oppervlaktes schoonmaken. Doordat er allemaal haakjes zitten in die stof, haal je het vuil weg. En dat is...

**Interviewer:**	Scheelt wringen?

**Respondent:**	Ja. Dat wringen. Dat scheelt een hele hoop.

**Interviewer:**	Dus vooral slimmer werken is dan vooral de sleutel?

**Respondent:**	Dus die slag is al gemaakt.

**Interviewer:**	Nou duidelijk. Als we eens kijken naar de beslissingen die jij als teamleider mag nemen. Waarover zou jij allemaal mogen beslissen, over wat voor een onderwerpen?

**Respondent:**	Wie is aanneem. Ik denk als ik binnen budget blijft, mag ik alles zelf wel beslissen. Maar ga ik daar over heen, dan moet ik wel een akkoord hebben van >HOOFD<. Die gaat dan weer naar >MANAGER< toe.

**Interviewer:**	En als we het dan over budget hebben, vallen daar dan ook dingen onder die je aan het personeel geeft? Dus als je in het personeel investeert. Een bos bloemen of nieuwe werkschoenen?

**Respondent:**	Dat is allemaal gebudgetteerd.

**Interviewer:**	Dus dat kun je allemaal zelf. Daarvoor hoef je geen akkoord?

**Respondent:**	Nee. Ja. Als iemand hier pijn in de knieën heeft en zelf geen schoenen kan betalen, fatsoenlijke met zolen. Dan betalen wij dat.

**Interviewer:**	Komen dat soort dingen vaak voor?

**Respondent:**	Toevallig nog een paar maanden geleden hadden we een vrouw die is gescheiden, die heeft gewoon minimum inkomen en die kan gewoon geen nieuwe schoenen kopen met zolen want ja, dat kost ook nog een 50 euro. En als je 1.000 euro in de maand salaris hebt...

**Interviewer:**	Dan blijft er weinig over?

**Respondent:**	Ja, dan blijft er weinig over dan denkt zij 'laat die zooltjes maar zitten'.

**Interviewer:**	Zijn er meer van dat soort soortgelijke situaties? Weliswaar niet een paar schoenen maar misschien iets anders?

**Respondent:**	Ja. Ik probeer dan wel te kijken naar andere werkzaamheden, maar meestal zijn de mensen dan al ziek. Bijvoorbeeld hier is een medewerker die is nu doekjes aan het tellen, maar die zit nu in een mitella dus die kan geen ander werkzaamheden doen. Dus ga je zoeken wat ze wel kan doen. En structureel gewoon. Ja. Soms wisselen we ook wel eens van afdeling. Kijk een kraamafdeling hebben natuurlijk heel veel badkamers en daar kan het heel hectisch zijn. Als je dan medewerkers hebt die moeite hebben met hectiek en flink doorwerken, dan moet je die daar wel van afhouden en soms moet je dat wel eens voor een medewerker beslissen.

**Interviewer:**	Maar als het dus aankomt op budget, zijn er af ten toe wel van die kleine uitgaves, zoals nu een paar schoenen...

**Respondent:**	Als we binnen budget blijven is er niets aan de hand.

**Interviewer:**	Maar dat is echt heel incidenteel?

**Respondent:**	Ja. Ja.

**Interviewer:**	Nog meer dingen waar jij over zou mogen beslissen. Mensen aannemen. Budget. Meer dingen die in je opkomen.

**Respondent:**	Ik vind best dat ik veel vrijheid heb. Als ik binnen budget blijf dan is het prima. En binnen het beleid van het ziekenhuis.

**Interviewer:**	De kader zijn wel...

**Respondent:**	Ja. Ja. *Respondent lacht*.

**Interviewer:**	Wat maakt naar jou idee een goede teamleider, wanneer vind jij dat jij je functie goed uitvoert?

**Respondent:**	Nou ik denk wel dat je snapt hoe de mensen. Hoe het werkt. En beslissingen durft te nemen.

**Interviewer:**	Wat bedoel je precies met 'je snapt hoe het werkt'?

**Respondent:**	Nou. Even denken hoe ik dat uitleg. Dit is ook een groep die, er zitten hele goeie bij. Er zitten ook mensen bij die de kantje er vanaf lopen en ja die moet je wel in het oog hebben en dat je die ook wel echt durft aan te spreken. Er zijn altijd wel dingen die waar mensen niet achter staan, die mensen niet leuk vinden, maar je toch moet doorvoeren voor het groter geheel. Voor het ziekenhuis voor de patiënt.

**Interviewer:**	Dus een kwestie van goed de mensen kennen, een beetje durf hebben, impopulaire beslissingen durven te maken?

**Respondent:**	Ja. Dat je niet de vriendin bent.

**Interviewer:**	Dat je daarboven staat?

**Respondent:**	Ja. Ja. Ja. Je bent hier niet om vriendjes te maken. En dat zie je natuurlijk wel bij >COÖRDINATOR< en >MEDEWERKER KWALITEIT< die zitten heel dicht bij de groep en die vinden dat heel moeilijk om te zeggen van 'joh, jij gaat nu niet naar die afdeling toe, maar naar die andere want dat is voor jou beter'.

**Interviewer:**	Want die zitten er gewoon helemaal, die staan er gewoon helemaal in?

**Respondent:**	Ja. Ja.

**Interviewer:**	Heb jij ook het idee dat je, om je functie van teamleider goed uit te voeren, dat je nog bepaalde kennis nodig hebt?

**Respondent:**	Ja. Altijd.

**Interviewer:**	Wat moet je weten?

**Respondent:**	Wat bedoel je dan met 'wat voor kennis'?

**Interviewer:**	Wat zijn dingen die je weet die jou helpen om je functie goed uit te oefenen?

**Respondent:**	Ik heb zo'n teamleidersessie gedaan met een aantal mensen. Dat heeft mij ook wel geholpen door niet alles. Door ook dingen terug te geven. Mensen proberen altijd alles bij je neer te leggen, maar probeer de verantwoordelijkheden daar neer te leggen waar ze horen.

**Interviewer:**	Het is dus heel belangrijk om te weten 'wat hoort bij mij'...

**Respondent:**	Wat hoort bij de ander. Ja. En niet voor het karretje laten spannen. Wat we natuurlijk gewend zijn een teamleider of leidinggevende die lost het wel voor mij op. Maar nu is het 'ga gewoon zelf het gesprek aan, jij hebt er last van'.

**Interviewer:**	... Wat stom. Dat is dan duidelijk. ... Oh, dat wilde ik vragen. Hoe ben je ervan op de hoogte gekomen wat nou wel en wat nou niet bij jouw functie hoort? Je zegt 'het is voor mij goed om alle dingen terug te leggen die niet bij mij horen', hoe zijn die scheidslijnen duidelijk geworden?

**Respondent:**	Ik denk wel in je functieomschrijving.

**Interviewer:**	Dat is een goede houvast voor jou?

**Respondent:**	Ja en voor andere medewerkers van >COÖRDINATOR< en >MEDEWERKER KWALITEIT< wat daarin staat. Daar staat ook gewoon duidelijk in omschreven wat zij moeten doen en waar ze voor betaald worden.

**Interviewer:**	En zodra er iets binnenkomt waarvan jij hebt 'dit valt buiten mijn functieomschrijving', dan verwijs je door van 'misschien is het handiger om even bij die aan te kloppen'.

**Respondent:**	Ja. Ja. Ja. Er zijn wel grijze gebieden, dus moet je kijken wie dat het beste op kan pakken.

**Interviewer:**	Dan ga je gewoon vragen van 'wie zou dit het beste kunnen doen?'.

**Respondent:**	Ja. Of ik stuur een mail en vraag. Dit zijn vaak van die operationele dingen. Zo groot is het niet.

**Interviewer:**	Heb jij ook nog bepaalde dingen, om dan door te bouwen wat binnen en buiten jouw functie ligt. We weten nu dat het binnen jouw functie heel operationeel ligt, zijn er ook nog dingen die echt heel sec buiten jou vallen waarvan je zoiets hebt 'hier houd ik mij echt niet mee bezig'?

**Respondent:**	Ja. Ik denk langetermijnbeleid van het ziekenhuis. Daar ben ik niet mee bezig. Dat is iets dat wij. ... Nee. ... Nee.

**Interviewer:**	Dus eigenlijk alles dat binnen de muren van deze afdeling valt, daarvoor kan ik naar jou toe. Maar als we het hebben over meerjarenbeleid.

**Respondent:**	Nou, kijk dat is het meerjarenbeleid. Waar we nu bezig zijn om die nieuwe functie van roomservicemedewerker, dat ben ik wel actief bij betrokken. Daar sta ik ook achter. Dus daar gaan we nu ook mee starten. Binnenkort komt er een MMO. Een medewerker meet onderzoek. Op drie verschillende afdelingen, en dan gaan we daar mee verder. Kijken wat eruit komt en je hoop natuurlijk dat het voor het facilitair gunstig uitkomt en dan vind ik het leuk om daar verder mee bezig te gaan. Dat is ook wel iets voor lange termijn.

**Interviewer:**	Maar echt de strategische dingen...

**Respondent:**	Nee. Nee. Strategisch niet.

**Interviewer:**	Als het om het hele ziekenhuis gaat. Dit project is dan een uitzondering? Want dit klinkt meer afdelingsoverstijgend? Eerder een uitzondering dan dat het normaal is?

**Respondent:**	Ja. Ja. Dit soort projecten maakt mijn functie ook leuk omdat je juist weer iets met lange-termijn bezig bent en veranderen en misschien ook een efficiëntieslag behalen.

**Interviewer:**	Als we het over de teamleider hebben, dan is dit een onderdeel dat er echt wel bij hoort?

**Respondent:**	Dat vind ik wel ja. Middellange termijn meedenken.

**Interviewer:**	En dat is dus niet alleen binnen je afdeling, maar ook dus veel daarbuiten als ik het goed begrijp?

**Respondent:**	Ja.

**Interviewer:**	Heb je ook nog een bepaalde manier hoe je werkt. Is het voor jou duidelijk hoe je je taken moet uitvoeren, of heb je daar je eigen draai aan gegeven?

**Respondent:**	... uhm. Je moet inderdaad uitkijken dat je opgeslokt wordt door alle dagelijkse dingen. Dat je uiteindelijk wel ruimte maakt in je agenda om inderdaad bezig bent met langetermijnzaken.

**Interviewer:**	De brandjes moeten je niet voor 40 uur per week bezig houden?

**Respondent:**	Nee. Nee.

**Interviewer:**	Is het dan ook de kunst om het weer terug te geven aan de medewerker of door te verwijzen naar >COÖRDINATOR< en >MEDEWERKER KWALITEIT<, of nee zeggen?

**Respondent:**	Ja. Ja. Ja.

**Interviewer:**	Toen jij hier begon als teamleider, wist jij toen al helemaal wat er van jou verwacht werd? Wat je wel moest doen en wat je niet moest doen?

**Respondent:**	... uhm. Nee, ik ben toen eigenlijk ook in het diepe gegooid.

**Interviewer:**	Hoe ging je daar mee om toen?

**Respondent:**	Uhm Ja. Hoe ging ik daar mee om? Ja, gewoon maar oppakken wat voorbij komt en ... op een gegeven moment komt daar wel structuur in.

**Interviewer:**	Echt iets dat je nu. Je zit hier nu meerdere jaren en heb je als het ware een soort cyclus opgebouwd als het ware van 'dit is normaal', zoals de jaargesprekken, dat is iets dat standaard terug komt?

**Respondent:**	Ja. Dat is het.

**Interviewer:**	Of het medewerkerstevredenheidsonderzoek, dat komt standaard terug.

**Respondent:**	Het werkoverleg.

**Interviewer:**	Dus ja. Je wordt in het diepe gegooid, maar over tijd wordt er een basis gelegd waar je iedere keer weer op terug kunt vallen?

**Respondent:**	Ja. Ja. Ja.

**Interviewer:**	Was dat niet heel stressvol in het begin?

**Respondent:**	... uhm. ... viel opzicht nog wel mee. Viel wel mee.

**Interviewer:**	Heb je ergens nog veel aan gehad die tijd toen je je functie aan het ontdekken was?

**Respondent:**	Ik denk het hele teamleidertraject, waar je ook met andere teamleiders, dan kun je met elkaar eens over hebben van waar je dan tegenaan loopt dan denk ik van 'oh, nou ja dat is het'.

**Interviewer:**	Een stukje kennisdeling?

**Respondent:**	Ja. Ook vooral omdat het dwars door de organisatie was. We hadden een aantal van facilitaire afdelingen en een aantal van >AFDELING<en.

**Interviewer:**	Het is nu ook over tijd duidelijk geworden wanneer je wat moet doen qua taken? Zijn er veel vaste verplichtingen waar je aan moet voldoen. Veel moetjes?

**Respondent:**	De DRP doe ik gelukkig niet, maar dat doen wel veel teamleiders volgens mij. Dat doe ik niet. Dat heb ik eigenlijk ook meteen aan het begin al. Dat deed >COÖRDINATOR< al en daar had ik zoiets van 'als jij daar goed in bent, dan moet je dat vooral blijven doen'. Ik merk wel dat >COÖRDINATOR< daar veel tijd aan kwijt is. Daar vind ik ook iets van. Daar heb ik het wel eens met >HOOFD< over gehad. Volgens mij is het veel handiger als meerdere mensen van de facilitaire afdelingen, teamleiders en coördinatoren, allemaal mee bezig zijn, volgens mij kun je daar dan ook een functie van maken. En houd je hier veel meer afstand van de mensen en dan is toch dat je een keer misschien een vriendje eens een keer hier helpt. Dat is niet helemaal ...

**Interviewer:**	Het rooster is wel echt een moetje...

**Respondent:**	Het rooster doet >COÖRDINATOR< dan. Maar ja, jaargesprekken. Verzuimsignaal bijhouden. Werkoverleggen. En de zieke medewerkers begeleiden. Dat vergt ook wel heel veel energie hoor die zieke medewerkers.

**Interviewer:**	In wat voor opzicht vergt dit veel energie?

**Respondent:**	Ik heb ook een paar bij die in het tweede jaar zitten zijn. Nou en die vinden ook natuurlijk van alles. Dat ze iets niet kunnen. Maar het is toch allemaal re-integreren en dat kan wel eens escaleren.

**Interviewer:**	Jij moet ze in die actieve houding krijgen?

**Respondent:**	Ja.

**Interviewer:**	En dat vreet veel energie?

**Respondent:**	Ja. Dat kost veel energie. Je hebt zieke medewerkers die niet alleen lichamelijk iets mankeren, maar mentaal ook.

**Interviewer:**	Duidelijk. Heb je het idee dat je als teamleider ook nog bepaald gedag moet uitstalen?

**Respondent:**	Ik moet hier niet zo suf gaan zitten van 'nou, ik weet het ook allemaal niet meer'. Ik moet hier wel een beetje power uitstralen. Positiviteit. Ja. Ja.

**Interviewer:**	Nog meer gedragingen waar ik aan moet denken?

**Respondent:**	Ik denk ook de normen van het ziekenhuis waar wij als ziekenhuis voor staan.

**Interviewer:**	Een voorbeeldfunctie zijn?

**Respondent:**	Een voorbeeldfunctie ja.

**Interviewer:**	Heb je zelf nog een visie op de teamleider. Wat is de toekomst van de teamleider in het ziekenhuis?

**Respondent:**	Ik ken die niet zo goed van de >AFDELING<en. Dat is daar natuurlijk ook nog wel een pittige functie. Volgens mij moet je daar ook nog flink in meewerken. Ik zie ze daar ook nog wel eens werkelijk staan. Dat doe ik niet.

**Interviewer:**	Wat vind je daarvan dat teamleider meewerken?

**Respondent:**	Ik vind dat je daar heel erg mee moet uitkijken want anders verlies je gewoon het overzicht.

**Interviewer:**	Omdat er dan gewoon aan alle kanten aan je getrokken wordt, met dat idee?

**Respondent:**	Ja. Als jij gaat meewerken dan kijk je volgens mij veel beter iemand inhuren of inzetten. Soms dan is het niet zo.

**Interviewer:**	Dus eigenlijk ben je wel blij dat je een beetje afgezonderd bent van de werkvloer? Dat je echt naast de werkvloer staat in plaats van er midden in?

**Respondent:**	Ja. Misschien wel ja.

**Interviewer:**	Vind je dat het goed geregeld is op facilitair gebied als het aankomt op de teamleider? Qua functieomschrijving, werkindeling.

**Respondent:**	Volgens mij verschilt het nogal. Als ik bijvoorbeeld kijk wat >TEAMLEIDER< doet, >TEAMLEIDER< is vooral bezig met DRP en plannen, ook met jaargesprekken, die heeft  er wel 80 geloof ik. >TEAMLEIDER< is misschien een ander typ dan ik ben. Ik vind het juist leuk om dingen te vernieuwen en te veranderen, maar >TEAMLEIDER< is dan meer van 'laat maar zo, het is goed'.

**Interviewer:**	Het traditionele behouden?

**Respondent:**	Ja. Ja.

**Interviewer:**	Ik heb in principe alles wel wat ik weten moet. Ik denk wel dat we het er over eens kunnen zijn dat de coördinatoren voor jou heel erg belangrijk zijn om de werkdruk bij jou laag te houden?

**Respondent:**	Ja. Ja. Ja. Dat is ook geweest. Eentje is een tijdje ziek geweest. Dan merk je gewoon dat het op ons alle twee een hele invloed heeft. Ik heb er drie he. Ik werk maar drie dagen als teamleider. Die vierde dag ben ik ook voor >ORGANISATIE<. Dus ik heb twee petten op. En daar is ook een voorwerker-coördinator-rol. En daar ben ik iemand voor aan het zoeken en waarschijnlijk heb ik haar gevonden.

**Interviewer:**	Mooi. Dan mag jij uiteindelijk de ja of de nee zeggen of zij het gaat worden. Dat is aan jou?

**Respondent:**	Ja. Dat ben ik. Dat ga ik doen. Alleen hadden wij net wel een gesprek met >COÖRDINATOR< en >MEDEWERKER KWALITEIT< want zij gaat dus ook samen met, dus er moet wel een klik zijn. Dus het is wel belangrijk dat zij het ook dragen.

**Interviewer:**	Je samenwerking met >HOOFD< hoe is de taakverdeling eigenlijk tussen jullie?

**Respondent:**	>HOOFD< is eigenlijk wat er allemaal speelt in het ziekenhuis en wat de veranderingen zijn en >HOOFD< geeft weer door wat hier speelt aan >MANAGER< en >HOOFD< is degene meer voor de VMARK of voor de roomservicemedewerker, dat project dat trekt >HOOFD< en neemt daar de sturing in.

**Interviewer:**	Heb je het idee dat het goed werkt, >HOOFD< als tussenschakel tussen jou en de manager?

**Respondent:**	Ja. Ik denk dat dat wel prima is.

**Interviewer:**	Ik weet niet of er zelf nog dingen zijn die je toe zou willen voegen?

**Respondent:**	Nee. Niet dat ik zou weten.

*Einde transcript*

# Transcript Interview FLM 8

**Duur interview:	50 minuten en 38 seconden**

**Functie:	Teamleider**

**Afdeling(en) onder supervisie:	Healthcare**

**Gender:	vrouw**

**Werkervaring in functie:	4 jaren**

**Interviewer:**	Laten we dan maar gewoon heel breed beginnen, wat doe jij in het dagelijks leven als teamleider? Waar ben jij verantwoordelijk voor?

**Respondent:**	Oh. Nou ja. Wat ik jou net al noemde. Jij komt mij interviewen. Dan vind ik het ook fijn om het een beetje voor te bereiden. Ik heb hier en daar wat dingetjes voor mijzelf op geschreven.

**Interviewer:**	Wat doe je zoal?

**Respondent:**	In eerste instantie denk ik dat een teamleider toch wel een beetje de spil van een afdeling is. Ik ben nu sinds een jaar weer leidinggevende op poli >AFDELING<. Ik heb dat een aantal jaren ook op verloskunde gedaan. Een jaar of vijf of zes geloof ik dat weet ik niet meer. Maar goed, in ieder geval wel gewend. En nou ja, wat doet een teamleider? Dat zijn echt heel veel taken. *Respondent lacht*. Een teamleider geeft in eerste instantie als je het grofweg wil benoemen, leiding, directe leiding aan het team. Je staat in verhouding met de bedrijfsleider. Dat is dus zeg maar mijn leidinggevende. Een bedrijfsleider stuurt mij aan op bepaalde taken en ik geef dat weer door aan het team. Dat is een klein beetje grofweg echt kort door de bocht verteld, denk ik, wat een teamleiderfunctie inhoud. Maar dan zijn er dus heel veel taken aan gekoppeld.

**Interviewer:**	Werk je ook nog samen met seniors hier op de poli >AFDELING<?

**Respondent:**	Ik heb senior verpleegkundige en dat was al een bestaande functie op deze polikliniek hier en daar werk ik samen mee.

**Interviewer:**	Hoe zijn de taken tussen jou en de senior verdeeld? Hoe zie ik dat voor me?

**Respondent:**	Hier is het zo dat. Kijk de senior hier die heeft een aantal taken die zij eigenlijk al die jaren, als takenpakket heeft. Zij doet heel veel met de planning. Alle dingen daaromheen organiseert zij. Ik heb wel zicht daar op. Maar zij houd zich met name daarmee bezig. Dat ontlast mij weer want dat is vrijwel een groot takenpakket. Ik kan haar daar wel in aansturen maar zij voert het uit. Plus het verlengde van taken waarvan ik denk 'oh, daar zou zij mij in kunnen ondersteunen of mee helpen'. Daar betrek ik haar bij en ik betrek haar natuurlijk ook bij alle dingen die operationeel zijn die lopen zodat zij op de hoogte is van. Ik heb nu ook ingesteld dat ik een keer per week een overleg met haar heb en dat we elkaar communicatief goed op de hoogte houden waar iedereen mee bezig is zodat iedereen op een lijn ligt, op een lengte.

**Interviewer:**	Het is echt een soort bondgenoot?

**Respondent:**	Ja. Zo moet je het zien ja.

**Interviewer:**	En als onderdeel van jouw takenpakket, herken je dan dat daar een stukje HR in zit verscholen?

**Respondent:**	Oh, uiteraard. Een heleboel dingen natuurlijk.

**Interviewer:**	Op wat voor een manier merk je dat? Aan wat voor dingen moet ik denken?

**Respondent:**	Als je nou specifiek naar HR. Ik denk dat sowieso wat betreft alle personele zaken die je aanliggen. Ik maak nu nog redelijk veel gebruik van om te informeren over dingen die er spelen, nou bijvoorbeeld een contractwijziging, aan uren, aan begeleiding in gesprekken daar heb ik nu toevallig iets voor de hand liggen waar ik HR bij betrek. Er zijn heel veel facetten waar ik zeker de weg daar naartoe weet en daar ook heel graag gebruik van wil maken.

**Interviewer:**	Maakt het een groot deel van jouw functie uit?

**Respondent:**	Nou, niet het grootste deel, nee. Het is wel een deel dat heel belangrijk is wat erbij hoort.

**Interviewer:**	Waar gaat het grootste gedeelte van je functie naar uit denk je?

**Respondent:**	Nou, ik denk toch wel naar coachen en motiveren van medewerkers op de werkvloer en daar gaan natuurlijk ook heel veel dingen in zitten als dienstroosters maken, de mensen aansturen op de werkvloer, zorgen voor kwaliteit, dat er een goede samenwerking is, dat was zeker voor deze polikliniek een hot item, dat je met elkaar probeert te werken en niet langs elkaar heen. Hier zitten ook twee drie verschillende clubjes die wat meer samenwerkingen zouden moeten krijgen. Daar ben ik mee bezig. Van elkaar leren feedback te geven. Confl en op te lossen. Open sfeer te creëren. Het houden van teamoverleggen. Ik heb het hier ingevoerd om een keer per week structureel een soort, een opening noem ik het, van de poli te creëren dat iedereen eens wat kan zeggen wat er speelt op de werkvloer en met name gerelateerd aan het werk. Dat doe ik met een gynaecoloog samen. Dat vind ik ook erg belangrijk om te doen. Wat is er nog mee. Er zijn zo veel dingen. Even denken. Je let ook verder op de personele bezetting. Het ziekteverzuim bijvoorbeeld. Dat wordt ook weer via HR. Daar ben ik ook contact mits je collega's hebt waar iets aan de hand is waar je meer begeleiding in wil hebben en het bieden. Die weg heb ik nu niet op het moment, maar ik weet dat het kan, dat wel. Ik heb nu wat langdurig zieke door griep, daar doe je niet zo heel veel mee.

**Interviewer:**	Jij bent wel degene die uiteindelijk dat verzuimgesprek voert als ze echt langdurig ziek zijn?

**Respondent:**	Inderdaad.

**Interviewer:**	Merk je op gebied van werving en selectie dat jij daar nog een rol in speelt?

**Respondent:**	Ja. Ook daarin.

**Interviewer:**	Op wat voor manier?

**Respondent:**	We hebben toevallig vacatureruimte gekregen zodat we daar ook op in kunnen spelen. Daar organiseer ik, samen met HR ook. De mensen worden geselecteerd. De mensen krijgen via jullie een uitnodiging en maken daar de afspraken mee. Ik houd daar de gesprekken mee en uiteindelijk komt er een kandidaat uit die aangenomen wordt. Daar wordt een vervolgtraject mee opgezet. Ook weer via HR zodat ook alles landt. Er worden gesprekken via HR opgezet.

**Interviewer:**	Selecteer jij ook de vacatures die binnen komen van 'die lijkt me geschikt of die lijkt me minder geschikt?'

**Respondent:**	Ja. Ook dat houd ik in de gaten.

**Interviewer:**	En als iemand eenmaal is aangenomen, ben jij dan ook degene die die persoon wegwijs gaat maken op de afdeling of besteed je dat uit?

**Respondent:**	Mede. Ik vind het wel belangrijk dat je eerst een soort van eerste ontvangt hebt. Zodat iemand zich ook een klein beetje welkom voelt. En dan is het zo dat ik een inwerkschema heb. Dat hebben we ook opgezet zodat nieuwe medewerkers hier goed ingewerkt kunnen worden en ook, ik zorg wel dat ik een beetje faciliteer en iemand koppelen dat iemand zich aan het begin een beetje kan oriënteren en een beetje kan inwerken. Dat is natuurlijk belangrijk he? Als je een goede basis biedt, dan pluk je daar later uiteindelijk de vruchten van. Denk ik. *Respondent lacht.*

**Interviewer:**	Je houdt ook alles bij qua personeelsbestanden? Als er iets gemuteerd moet worden dan komt dat allemaal bij jou terecht?

**Respondent:**	Ja, dat klopt en dan te denken aan ziekmeldingen, betermeldingen, dat hele DRP-gebeuren. Aan het einde van de maand heb je hele lijsten die je invult van de uren die medewerkers hebben gemaakt. Dat is ook een best wel verantwoordelijke taak he? Dat je daar ook goed in muteert en dat dat uiteindelijk ook goed wordt uitgevoerd. Ja, dat doe ik ook.

**Interviewer:**	Bijvoorbeeld iets met opleidingen, ben jij ook degene die bepaalt wie welke opleiding gaat doen?

**Respondent:**	Nou kijk, het is zo dat, is gebudgetteerd he. Daar krijgen we een budget voor en ik houd jaargesprekken. Dat heb ik natuurlijk dit jaar ook met alle medewerkers gedaan. En die jaargesprekken daar komt natuurlijk ook een planning uit voort. Om ervoor te zorgen dat je medewerkers gemotiveerd houdt  en vooral dat ze een leerklimaat kunnen verwachten.

*Telefoon gaat.*

**Respondent:**	Dat is ook iets waar ik natuurlijk op toezie en dat is ook iets dat ik bespreek met mijn bedrijfsleider wat er mogelijk is. Ik inventariseer de wensen en ik kijk ook of het relevant is. Kijk mensen kunnen soms ook wensen hebben voor opleidingen die eigenlijk niet relevant zijn voor hun vakgebied of voor de toekomst. En dan moet je ook wel eens zeggen 'dit is eigenlijk iets wat nu niet aansluit op je functie'. Daar moet je een beetje in selecteren. Als ik die planning een beetje voor ogen heb, helder heb, dan bespreek ik die met >BEDRIJFSLEIDER< die kan mij dan wee op weg helpen wat mogelijk is en niet.

**Interviewer:**	Het is dan uiteindelijk de bedrijfsleider die akkoord geeft op jouw plan van 'ik zou die op training willen sturen'? Jij komt met het voorstel...

**Respondent:**	Ik bespreek dat wel. Ik neem daar niet een eigen beslissing in.

**Interviewer:**	Duidelijk. Sta je ook wel eens voor de groep en geef je af en toe iets van een training of iets dergelijks qua personeel?

**Respondent:**	Ja. Ik ga sowieso. Onze poliklieken lopen een beetje achter in bepaalde kwaliteitseisen die gesteld zijn. Andere poliklinieken zijn daar al verder in. Ik ben nu aan het oppakken van, bijvoorbeeld 'weet je convenant, voorbehouden handelingen, enzovoort'. Daar heb ik nu een team voor samengesteld. Ik heb een aantal mensen laten trainen en met deze mensen ga ik een soort avond creëren, of meerdere avonden, ligt eraan hoe snel we er doorheen kunnen, om bijvoorbeeld die mensen te toetsen. Daar hoort natuurlijk ook een soort uitleg, klinische les aan vast. Dat vind ik harstikke leuk om te geven. Ik zit de teamvergaderingen zelf voor. In de toekomst, kijk als er iets op mijn pad ligt waar ik vakinhoudelijk bekwaam voor ben, vind ik het ook heel erg leuk om een klinische les te geven. Maar dat ligt maar net aan het vakkenpakket. Dan moet je ook mijn achtergrond bekijken en of het aansluit op de poli. Van de verloskunde heb ik best wel veel bagage, maar die sluit niet altijd aan op hier. Ik ben betrokken bij de drills. Dat is een instrument dat gebuikt wordt op de afdeling verloskunde daar worden acute verloskundige scenario’s nagespeeld, met echte situaties die zich voor kunnen doen. Daar betrek ik de poli ook bij want dat is een stuk in het verlengde van hier en daar neem ik de mensen ook weer in mee. Die trainingen geef ik zelf ook, dus daar ben ik ook weer bij betrokken. Dus dat zijn eigenlijk een beetje de dingen die ik extra doe aan uitleg, trainingen, hoe je het noemt.

**Interviewer:**	Werk je ook nog wel eens mee op de werkvloer? Want dat zie je af en toe ook nog wel eens bij een teamleider dat ze dus ook af en toe nog in het wit lopen.

**Respondent:**	Ja. Hoort ook he? Kijk ik vind het heel nadelig maar ik heb hier echt een kamertje ergens helemaal achteraf. Ik heb heel weinig, dat is niet waar, ik zoek het contact heel erg maar ik zit hier nog wel, ik moet natuurlijk ook dingen uitwerken. En ik voel ook dat ik heel erg ver weg zit van mijn team. Eigenlijk ga ik veel liever eigenlijk richting het team ergens een plek willen hebben. Dan zien zij mij, ik hen, heb ik ook door wat er speelt. Ik zoek ze echt wel heel veel geregeld op. Maar dat is een groot nadeel. Maar het is ook wel zo dat, ik deel me ook wel eens af en toe een spoedpoli mee te draaien, dat is ook de functie die ik kan vervullen. Kijk, doktersassistentenfunctie, daar ben ik eerlijkgezegd niet voor opgeleid en daar verdiep ik mij ook niet in. Ik ga niet afspraken plannen of zo. Dat doe ik niet. Niet omdat het mij te laag is qua niveau, maar dat kost zoveel energie om mij dat eigen te maken. Maar de spoedpoli zeg, als verpleegkundige, kan ik natuurlijk meedraaien. Dat doe ik bij acute uitval. Stel dat het echt niet meer kan oplossen, en mijn agenda laat het toe, dan doe ik het sowieso. Maar de ene keer zit ik met mijn planning, met name het afgelopen jaar toch wel wat krapper, waardoor ik mezelf ook inplan. Maar dat heeft ook voordelen. Voordelen voor mezelf. Dat ik zelf in de praktijk kan zien hoe het zit en werkt. Dus ook als mensen tegen problemen aan lopen, dan ja 'dan heb je een punt he, daar moeten we misschien wel aan werken of anders organiseren of daar moeten we naar kijken'. Maar ik vind ook dat het meer band geeft naar een afdeling. Dat is echt mijn mening erover.

**Interviewer:**	Hoe recent heb je nog op de werkvloer gestaan, echt in functie?

**Respondent:**	Dat is anderhalve week geleden nog. *Respondent lacht.* Ik doe dat wel geregeld. Niet te veel. Want dan gaat het ook ten koste. Dat is ook een valkuil. Daar moet je ook voor waken. Het kan een valkuil voor jezelf zijn als je te vaak wil dat die gaten worden opgevuld, en je doet dat, dan kan dat ook weer ten koste gaan van je eigen functie. En geloof met het zijn uren, ik zit een minuut stil. *Respondent lacht.*

**Interviewer:**	Het is een soort spanning waar je tussen zit?

**Respondent:**	Ja. Dat kan je. Je moet er een gezonde balans in proberen te vinden.

**Interviewer:**	En als je op de werkvloer staat, geef je dan ook nog wel eens instructies aan de collega's van 'goh ik zie dat je het nu zo aanpakt, zou je dat misschien niet anders kunnen'?

**Respondent:**	Ja. Natuurlijk. Als ik zie dat dingen anders zouden moeten, ja nou ja, uiteraard. Ik benoem het altijd een beetje coachend, voorzichtig. Ik zou niet. Ik ben geen autoritaire leider. Absoluut niet. Ik zou er nooit zo gaan staan van 'wat je nu doet, dat kan niet'. Dat moet je een beetje aanvoelen en op die manier. Maar uiteraard, als ik iets waarvan ik denk dat moet anders of dat kan anders, dan zou ik daar wel wat van zeggen. Tuurlijk.

**Interviewer:**	De manier waarop je dat aanpakt, je hebt wel het gevoel dat dat goede impact heeft?

**Respondent:**	Ja. Ja.

**Interviewer:**	Je ziet iets. Je wil dat het anders gedaan wordt. Uiteindelijk gebeurt het ook anders?

**Respondent:**	Uiteraard. Ja. Ik denk dat we hier nu met elkaar. Kijk wat ik zeg, ik zit hier nu een jaar en heb de mensen ook allemaal moeten leren kennen. Ik denk dat wij een hele, tot nu toe mag ik zeggen, echt zeggen, dat we een open en leuke werk, ruimte, prettige werksfeer hebben gecreëerd waar nu begint te ontstaan. Dat was eerst niet zo. Maar, dat lijkt er naartoe te groeien.

**Interviewer:**	Welke ruimte bedoel je?

**Respondent:**	Dat elkaar bijvoorbeeld feedback kan geven en dat je elkaar aanspreken over iets dat minder plezierig loopt of minder goed loopt of anders moet, zonder dat je daardoor je aangevallen voelt.

**Interviewer:**	Juist. Dus het disciplineren en corrigeren ligt ook wel bij jou, maar dat doe je op een hele coachende manier?

**Respondent:**	Ja. Ja.

**Interviewer:**	Werkt dat ook goed als je hele ernstige incidenten meemaakt met collega's?

**Respondent:**	Dat heb ik hier nog niet meegemaakt. Maar dan praat ik even vanuit ervaringen uit het verleden. Kijk er zijn grenzen he? Je kan proberen om het in harmonie, ik weet niet hoe ik het moet noemen, op de manier waarop jet het doet, proberen zo ver mogelijk te komen. Maar als er iets gebeurt dat een ernstige consequentie heeft of iets anders, dan zijn er andere methodes om gesprekken in te gaan. Dan houdt het op. En dan wordt het ook nog op een correcte manier gedaan.

**Interviewer:**	Dan pak je toch wel een stukje autoritair dan? Dan is het te ver gegaan?

**Respondent:**	Dan ligt het anders. Maar goed ik heb het niet voor handen wat het functioneren betreft op de poli en ja goed, als je met iemand een traject in moet wegens disfunctioneren, ja. Dan weet je, dat gaat. Dat weet ik nu toevallig ben ik daarmee bezig, maar dan zou dat ook via HR moeten. Dan wil ik toch heel graag handvaten en hulp daarbij 'hoe gaan we dit goed aanpakken? '. Dat het op de juiste manier over gaat.

**Interviewer:**	Als jij dan je team waardeert, op wat voor manier doe je dat?

**Respondent:**	Ik complimenteer heel veel. Ik ben vrij, hoe moet ik mezelf omschrijven, ik ben altijd vrolijk. Over het algemeen een enthousiast iemand. Als ik zie hoe dingen worden aangepakt, ook al zijn het maar kleine dingen, ik complimenteer heel graag en heel geregeld. Het moet niet overkomen van 'oh daar komt ze weer', nee zo ook niet. Maar ik denk dat het wel gewaardeerd wordt en dat dat een positieve drive geeft. Als je ziet dat mensen iets aanpakken of doen waarvan je denkt 'jeetje wat leuk' en ik krijg ook de medewerking nu, dat merk je ook. Want ik vraag ook het team, nu ik wat meer personele ruimte heb, we zitten hier niet te uitgebreid maar ik heb iets meer speling. Ik betrek de mensen bij projecten en ze vinden het fantastisch. En dan denk ik 'die vindt dat wel leuk om te doen' en dat vraag ik aan hem. En dan geef ik het enthousiasme over en dan stel ik deadlines van 'zullen we proberen het binnen twee weken op te lossen?'. Dat gaat, dat loopt prima.

**Interviewer:**	Harstikke goed. Ben jij ook de persoon, mocht het uitlopen op arbeidsconfl>AFDELING<en of als er misschien klachten zijn, ben jij dan ook de persoon die dat afhandelt?

**Respondent:**	Bedoel je dan een confl>AFDELING< met een collega?

**Interviewer:**	Ja, bijvoorbeeld.

**Respondent:**	Ja. Heb ik gehad in het verleden.

**Interviewer:**	Of dat er een advocaat bij gehaald moest worden.

Dat heb ik gehad. Dat heb ik gehad in het verleden. Uit de verloskundetijd heb ik, daar was destijds een disfunctionerende medewerker. Die is uiteindelijk op mijn afdeling geplaatst. Die had al een heel dossier opgebouwd en daar was ik destijds bij betrokken en dat ging tot de rechtbank in >PLAATS< en tot ontslag. En daar was ik zeer zeker bij betrokken. Dat vond ik wat minder aangenaam, maar ook dat hoort erbij.

**Interviewer:**	En stond je er toen alleen voor of had je nog hulp van iemand anders?

**Respondent:**	Nee. Daar was ook HR bij betrokken en daar heb ik veel ondersteuning van gekregen en ook gevraagd en aan mijn toenmalig hoofd van de afdeling. Als een soort driespan.

**Interviewer:**	Omdat dit zo serieus was en zoveel gevolgen had?

**Respondent:**	Ja. Een keer heb ik daar een ervaring mee gehad. Ik hoop dat het niet vaker gebeurt. Dat is wat minder leuk he?

**Interviewer:**	Heb je ook beslissingsbevoegdheid als het aankomt op de salarisschalen van medewerkers, of ligt dat allemaal vast?

**Respondent:**	Daar zeg je wat. Nee. Nee. Dat ligt allemaal vast he? Kijk het zijn twee functies hier. Het is een verpleegkundefunctie of een doktersassistentenfunctie. En dan heb je nog wel een verpleegkundig specialist. Die heeft, is ook ooit in een bepaalde salarisschaal gekomen. Daar heb ik eigenlijk nog nooit mee te maken gehad om te moeten bedenken dat het anders moet. Nee. Dat is ook eigenlijk nog nooit. Daar heb ik nog nooit iets mee gehad.

**Interviewer:**	Ligt er ook een belangrijke communicatiefunctie bij jou als teamleider?

**Respondent:**	Heel belangrijk. En dat merk ik zelf ook. Dan voel ik me soms, dan merk ik soms af en toe eens, daar moet ik echt aan wennen. Ik denk wel dat ik alles heel veel communiceer ook door nieuwsbrieven te schrijven. Door dingen die soms plots op je bordje komen, schiet het daar wel eens bij in en denk je 'dat doe ik even zus en zo', maar dan is het net niet gecommuniceerd, waardoor er geen grove dingen gebeuren, maar dan is het van 'oh, dat wisten we helemaal niet'. Dan dek ik 'oh, zie je, had ik moeten melden'. Ik denk dat dat heel essentieel en belangrijk is. Dat je enorm goede communicatie hebt dat je alles wat je moet delen ook daadwerkelijk deelt en wat ik ook belangrijk vind, wat ik ook heb ingesteld, is dat er structurele overlegmomenten die jij net noemde, met de senior. Dat je daar ook de communicatie goed mee hebt lopen en van elkaar weet waar iedereen mee bezig is en ik heb nu ook ingesteld dat een keer in de twee weken met de gynaecoloog, die van de poli >AFDELING< verantwoordelijk is, een overleg mee te hebben om ook daar de zaken te bespreken die spelen op de werkvloer . Van zowel hen uit, als van mij uit. Wat ik voelde was, je had gewoon heel vaak dagelijks van die geïrriteerde kleine dingetjes. Dan denk ik 'kan je wel ad hoc elke keer op inspelen', maar ik denk eerder dat het beter is om te verzamelen en in een overleg structureel een oplossing te zoeken. Die communicatie naar het team of de communicatie naar gynaecologen, onderling, enzovoort. Dat is heel belangrijk. Als dat niet loopt, kan je het denk ik wel vergeten. Ik denk dat dat een van de belangrijkste taken is om een goed samenhangend geheel hier te vormen.

**Interviewer:**	Je geeft heel erg aan 'ik communiceer naar het team', van wat je dan misschien van >BEDRIJFLEIDER< hebt gehoord of van iemand anders, heb je het idee dat je ook de andere kant op moet communiceren? Dus alles wat jij dan vanuit je teamoverleg verzamelt, dat jij dan de persoon bent die dat weer doorspeelt naar...

**Respondent:**	Ja. Uiteraard. Daarom heb ik die overlegmomenten he? Dus ik heb ook aangegeven aan het team. Ze weten dat ik om de twee weken, bijvoorbeeld nu met de gynaecoloog, om tafel zit om dingen te bespreken die op de werkvloer spelen. Heel breed is dat he? Dus niet allemaal iets aan irritaties, daar gaat het juist bijna niet over, echt dingen die belangrijk zijn voor het voortzetten van het werk. Dat weten zij. Dus zij koppelen dingen aan mij 'oh >TEAMLEIDER<, wij lopen hier en hier tegen aan, wil jij dat bespreken?'. Dus vanuit mijn team krijg ik zeker ook input om bij de gynaecoloog iets bespreekbaar te maken. Maar ook krijg ik input vanuit het team om te zeggen 'goh >BEDRIJFSLEIDER<, hoe kunnen we dat oplossen? Heb jij een idee of wat zijn de mogelijkheden?'. Want als bedrijfsleider heeft zij soms over bepaalde dingen meer zicht dan dat ik dat heb. Met dingen waarvan ik denk 'dat is grensoverschrijdend wat mij betreft', ben ik weer bij >BEDRIJFSLEIDER<.

**Interviewer:**	Dit hoor erbij?

**Respondent:**	Ja.

**Interviewer:**	Dit is echt onderdeel van een teamleider?

**Respondent:**	Ja. Een heel belangrijk deel ook. Het valt of staat met communicatie. Als die niet goed is, heb je echt een probleem.

**Interviewer:**	Heb je ook als je iets van een plan hebt, toets je dat dan ook eens bij medewerkers van 'goh, wat zou jij hier van vinden?' of 'ik sta op het punt dit te beslissen, wat is jouw kijk?'.

**Respondent:**	Ja, maar soms heb je dingen waarvan je denkt 'hier moet je geen discussie om willen hebben, dat moet je gewoon voor lief aannemen'. Dan doe je het als een soort mededeling. Maar je geeft ook de ruimte om mee te denken, juist, vind ik, want hoe meer gedragen door het team, hoe meer en hoe groter het succes is. Lijkt mij. Dus dat doe ik zeker. Ik koppel zeker terug en vraag ook aan hen 'wat vind je er van?'.

**Interviewer:**	Ligt er naar jou idee een soort scheidslijn, nu doe ik het als een mededeling en...

**Respondent:**	Ja. Soms zijn er dingen waarvan je denkt 'hier wil ik geen discussie over, want dat leidt misschien tot niets of tot', ja, dat moet je een beetje aanvoelen. Dat moet je ook, als het dingen zijn. Ja. Ik denk dat je het aan moet voelen. Als dingen echt moeten of ja, dan moet je het toch spelen als meer een soort mededeling en proberen het akkoord te winnen, en als het dingen zijn waarvan ik denk 'dit wil ik heel breed gedragen hebben, en daar wil ik echt succesverzekerd zijn' en het is iets waarvan je denkt 'als ik dit in een breder perspectief zet en een betrokkenheid vraag, heb ik meer kans van slagen', dan doe ik dat wel. Maar dan moet ik nu echt concreet voorbeelden voor me hebben om te kunnen zeggen 'ik zou dit in dit geval nu zo aanpakken en dit in geval van dit zo aanpakken'.

**Interviewer:**	Dus echt afhankelijk van het onderwerp?

**Respondent:**	Ja. En dan een beetje op je gevoel afspelen.

**Interviewer:**	Geen zwart-wit...

**Respondent:**	Nee. Ik denk nooit zwartwit.

**Interviewer:**	Heb je ook het idee dat onderwerpen als kwaliteit, veiligheid, duurzame inzetbaarheid, ligt dat ook bij jou?

**Respondent:**	Al maar door. Al maar door. Kom maar door. Zeker omdat het hier nog zo achterloopt. Er is hier nog heel veel werk aan de winkel. Dat is toch ook iets waarvoor je moet waken van 'Rome is ook niet in een dag gebouwd', maar het is wel, hier ligt nog zo veel wat op dat gebied aan kwaliteitseisen vereist is. Zeker nu we de IGZ continu in huis hebben. Daar ben ik volop mee bezig.

**Interviewer:**	Beleid voor aan het maken?

**Respondent:**	Ja. Dus echt wat de organisatie vraagt, wat ook echt moet, dat probeer ik toch dit jaar een heel eind te komen. Zal het nog niet allemaal gered hebben in een jaar, maar dat lijkt mij...

**Interviewer:**	Je hebt een jaarplan voor jezelf opgesteld?

**Respondent:**	Ik heb toevallig. Ook daar weer in. Leuk dat je dat je dat een jaarplan noemt, dat heb ik zeker voor ogen. Dat bespreek ik natuurlijk met >BEDRIJFSLEIDER< want die is ook van het meerjarig beleidsplan. In het meerjarig beleidsplan hebben ze ook het een en ander uitgezet en doelen gesteld waar we aan moeten voldoen. Dat wordt natuurlijk ook met mij besproken en daar moeten we natuurlijk ook weer proberen het team bij te betrekken. Dat zijn trouwens ook alle regelmatig agendapunten op een teamoverleg zodat je ze daarbij ook betrekt. Zodat ze ook weten wat er speelt en ook weten waar ze aan mee moeten werken en waar we naartoe gaan. Sturing en richting geven. Maar zelfs daarmee wordt ook aan de medewerkers gevraagd 'goh, jongens schiet erop, wat vinden jullie ervan, vinden jullie het haalbaar?'. Ik moet wel zeggen dat op zo'n niveaus, zoals het schrijven van een beleidsplan, als je dat hier presenteer aan de medewerkers heb ik toch de indruk dat dat niet helemaal speelt. Dat het toch iets is waarvan ze denken 'ach, het zal wel'. En je moet het ook een klein beetje kunnen plaatsen. Het zijn mensen met een bepaald soort achtergrond, met een bepaald soort opleiding. Doktersassistenten zijn betrokken. Klantgerichtheid staat hier echt op nummer een. Dat doen ze echt werklijkwaar zo fantastisch. Ze buigen en wringen zich in duizend bochten om de patiënt centraal te stellen, maar als het dan afdelingsoverstijgend gaat en gaat richting beleid en organisatiebeleid enzovoort, ik zie ze dan wel een beetje afhaken hoor. Dan ze denken van 'oh, nou het zal wel, dat gaat mijn petje te na'. Heb ik alle begrip voor. Maar je moet ze wel informeren en dan een klein beetje op niveau, niet op niveau, maar als de taken bekend zijn ze daar een beetje in meenemen en het een beetje versimpelen en aangeven wat je ermee bedoelt. Als je zo'n plan daar neerlegt dan schudden ze echt met hun oren.

**Interviewer:**	Als we kijken naar jouw bevoegdheden als teamleider. Over wat voor dingen zou jij allemaal mogen beslissen?

**Respondent:**	Dat is wel moeilijk.

**Interviewer:**	Of misschien is het makkelijker om te vragen waar jij niet zelf over zou mogen beslissen maar waarover jij je laat over adviseren of informeren.

**Respondent:**	Dat is ook lastig. Daar overval je mij mee. Mijn bevoegdheden. *Respondent lacht*. Ik denk niet dat ik gekke dingen uithaal. Ik koppel alles terug. Al is het maar als een soort van mededeling aan mijn bedrijfsleider. Ik doe mijn ding hier en ga zelfstandig aan het werk, maar over gelden, over aanschaf. Dat ga ik allemaal niet doen. Dat ga ik wel voorstellen, maar zeker niet zelfstandig besluiten.

**Interviewer:**	Zijn het dan vooral de dingen die met de werkzaamheden zelf te maken hebben, de kleinere dingen?

**Respondent:**	Ja. Alles wat ik niet benoemde, doe ik zelfstandig.

**Interviewer:**	Zodra het meld geld te maken gaat krijgen, dan haak je af?

**Respondent:**	Ja. Dan haak ik ook af. Of besluiten zomaar nemen of iets invoeren, nee nee, bijvoorbeeld blokkades op de spreekuren zetten, ik noem maar wat. Alles doe ik in overleg. Maar goed, dat komt dan voort uit een soort probleem, dat moet ik dan analyseren, en dan moet ik de juiste persoon vinden die mij kunnen adviseren daarin en dus uiteindelijk, samen met mijn bedrijfsleider, een besluit moet nemen. Maar dat doe ik niet zo even snel. Nee. Ik weet wel echt mijn grenzen. Hoop ik. Ik heb in ieder geval nog niets negatiefs gehoord. *Respondent lacht.*

**Interviewer:**	Maar dat was dus eigenlijk mijn volgende vraag, als het dus op geld aankomt, heb je zoiets 'dat ligt niet bij mij, alles dat met geld te maken heeft, dat ligt bij >BEDRIJFSLEIDER<. Als we het dan hebben over kleinere personeelsuitgaves, zoals een bosje bloemen...

**Respondent:**	Dat is oké. *Respondent lacht.*

**Interviewer:**	Er is dus iets heel kleins. Dat is iets dat kom ik vaker dus tegen. Dat soort kleine uitgaves maak je dan wel zelfstandig?

**Respondent:**	Kijk het is ook zo, je moet ook een balans zien te vinden denk in de. Kijk een bedrijfsleider, en zeker onze >BEDRIJFSLEIDER< die is ontzettend druk, ieder op zijn gebied ook. En ik denk dat je afspreekt. Kijk ik laat haar wel alles weten. Maar als ik inderdaad voor iets een bloemetje moet organiseren of uit een afdeling een borreltje moet komen voor een jubileum, ja dat overleg ik. Dat zeg ik dan ook, maar dat regel ik. Dan is dat oké.

**Interviewer:**	Dan is het meer een mededeling dan echt de vraag 'mag ik?'.

**Respondent:**	Nou in het begin heb ik wel steeds de vraag gesteld 'mag ik' omdat ik ook een klein beetje moet aftasten in hoeverre ik die bevoegdheid heb. Nu ik hier zo over nadenk heb ik daar eigenlijk niet echt een concrete richtlijn van 'dat mag jij wel en dat mag jij niet'. Nooit zo over gehad. Ik merkte wel gaandeweg als je dit soort dingetjes inderdaad even iets moest regelen op kleinschalig niveau, dat het goed was om te regelen. Ik meld het wel. Altijd. Want >BEDRIJFSLEIDER< moet het ook weten.

**Interviewer:**	Het is >BEDRIJFSLEIDER< budget?

**Respondent:**	Ja. >BEDRIJFSLEIDER< doet ook interne en externe budgettering natuurlijk. Daar heb ik eigenlijk weinig mee te maken. In die zin niet mee te maken dat ik het niet onderhoud, maar ik moet natuurlijk wel aangeven waar ik mee bezig ben. En dat doe ik zeker. Maar ik regel het uiteindelijk zelfstandig. Maar ik denk wel in goed overleg en dat is goed. >BEDRIJFSLEIDER< zie het ook vanzelf verschijnen want >BEDRIJFSLEIDER< moet akkoord geven.

**Interviewer:**	Op een of andere manier...

**Respondent:**	Komt het bij >BEDRIJFSLEIDER< op het bordje. *Respondent lacht.* En dan zou >BEDRIJFSLEIDER< op een gegeven moment zeggen '>TEAMLEIDER< je gaat te veel bloemetjes vergeven'. >BEDRIJFSLEIDER< weet gewoon wat hier speelt. Ik licht >BEDRIJFSLEIDER< daarover in. Dat gaat altijd in overleg. Dat is ook logisch. Je gaat even. >BEDRIJFSLEIDER< wil ook graag weten wat er speelt. Niet in de kleinste details, maar in grove lijnen is het voor >BEDRIJFSLEIDER< dat er een jubileum is of dat er een afscheid is of wat dan ook is. Dat >BEDRIJFSLEIDER< betrokken is bij de medewerkers zelf.

**Interviewer:**	Heb jij nog een kijk op hoe jij vindt dat jij goed werk levert?

**Respondent:**	Dat vind ik ook een goede vraag hoor. Hoe merk je dat. Ik vind heel belangrijk eigenlijk dat je binnen zo'n team de sfeer goed hebt. Dat was ook een beetje het kerndoel hier. Want als je nogmaals de basis oké hebt, dan kun je ook opbouwen. Hoe toets je dat. Dat is moeilijk he? Ik heb een instrument en dat is het medewerkerstevredenheidsonderzoek en daar heb ik goed op gescoord?

**Interviewer:**	Dat is jouw graadmeter?

**Respondent:**	Dat heb ik nu voor het eerste meegemaakt. Dat was in toenmalige tijd niet toen ik leidinggevende was. Maar ja toen had ik wel het gevoel 'iedereen is blij met je'. Je moet natuurlijk ook wel eens maatregelen nemen die niet populair zijn. Maar goed het is niet anders, dat hoort ook bij mijn functie. Ik zal ook niet verwachten dat iedereen mij leuk vind. Dat hoeft ook niet. Ik vind het wel heel belangrijk dat je een vertrouwensbasis hebt opgebouwd, dat je een soort van lid bent van het team, maar ook de leiding daarin kunt nemen. Die sfeer dat vind ik fantastisch en dat heb ik hier ook. Dat is wel de graadmeter waarmee je zou kunnen toetsen. Het is een anonieme enquête he? De ze hoeven hun namen niet, en ze hoeven niet te slijmen he naar mij? Als ik dan zie dat ik op drie onderdelen, wat >BEDRIJFSLEIDER< ook zei, echt heel goed scoorde. Dan denk ik 'je doet het niet zo slecht'.

**Interviewer:**	Je hebt niet heel gevoelsmatig, nu heb ik goed werk geleverd?

**Respondent:**	Nee. Ik vind dat heel lastig. Ik hoop dat maar. Ik vind het heel moeilijk om te zeggen. Ik weet het niet te toetsen. Kijk ik zie resultaten. Als er iets gedaan is en je hebt iets voor elkaar en iets, weet ik veel, ze kenden niet eens hun dienstlijst om maar even een begin te maken. Dan hebben ze dienstlijsten op een gegeven moment hier samengesteld en is drie, twee drie maanden vooruit gepland, meer haal ik ook niet omdat de artsen hun roosters dan nog niet klaar hebben.

*Telefoon gaat.*

**Interviewer:**	Wat vind jij belangrijk voor een teamleider om te weten? Aan kennis om goed te functioneren?

**Respondent:**	Ik vind het belangrijk om vakinhoudelijk op de hoogte te blijven van alles. Ik vind ook wel, er wordt heel veel aangeboden, ook via het ziekenhuis, waar ik nu ook regelmatig gebruik van probeer te maken. Nou ja, alle instrumenten die worden aangeboden op het gebied van automatisering bijvoorbeeld. Dat je daarvan op de hoogte blijft. Cursussen en van alles en nog meer kun je volgen. Het is ook maar net waar je je zwakke punten in hebt, dat je dat probeert te verbeteren en te versterken. Dat vind ik voor mezelf heel belangrijk, maar dan ontbreekt het mij aan tijd. Dat merk ik wel. Toen ik hier ingestapt ben. Al die computerprogramma's. Die kende ik allemaal niet. Daar moest ik wel behoorlijk voor investeren om daar een beetje wegwijs in te worden. Maar dat is net zo belangrijk. Als je randvoorwaarden in je werk vind ik een belangrijke taak. Vakinhoudelijk vind ik een taak. Maar ook alles op het gebied van verdere ontwikkeling en leiding geven en wat je aan dingen kunt bedenken. Ik wil wel aan de bal blijven en op de hoogte blijven.

**Interviewer:**	Een stukje HR-kennis die je nodig hebt?

**Respondent:**	Altijd welkom. Want op dat gebied weet ik ook nog niet alles. Maar ik weet natuurlijk wel, je kunt via Ido en Portaal, kan ik zien waar jullie mee bezig zijn. Als ik iets heb waar ik niet uit kom heb ik toch wel iets waar ik op kan zoeken of iemand kan vragen 'helpen jullie mij daarin'. Ik weet wel de wegen te bewandelen. Dat zeker. Dat vind ik wel het allerbelangrijkste.

**Interviewer:**	Je weet altijd wel de persoon te vinden die het weet?

**Respondent:**	Ja. En ook van hen gebruik maken. Als ieder zijn, kijk dat vak, dat HR, dat is al zo'n uitgebreid iets wat jullie bieden voor het ziekenhuis waar wij dan, althans ik dan laat ik voor mezelf spreken, gebruik van kan maken als ik het niet weet.

**Interviewer:**	Heb jij nog voor je hoe de taken zijn verdeeld tussen jou en >BEDRIJFSLEIDER<? Welke taken horen wel bij jou als teamleider en welke taken horen niet bij jou als teamleider?

**Respondent:**	Ik denk dat als je nu naar ons gesprek kijkt en dat is best wel redelijk veel toegelicht waarvan ik vind dat mijn taken zijn en wat bij mij hoort. Dan zou ik daar de functiebeschrijving van de bedrijfsleider bij moeten pakken, die ik dus niet in mijn hoofd heb, maar >BEDRIJFSLEIDER< is meer op macroniveau bezig. En ik denk. Ik weet niet of ik het moet zeggen van 'het hoort er niet bij'. Ik denk dat je altijd over dingen samen overlegt, maar >BEDRIJFSLEIDER< is meer met bedrijfsvoering bezig. Daar ben ik niet zo uitvoerig mee bezig. Ik heb de taakfunctieomschrijving van >BEDRIJFSLEIDER< niet zo goed voor ogen. *Respondent lacht*.

**Interviewer:**	Is >BEDRIJFSLEIDER< dan echt afdeling overstijgend?

**Respondent:**	Meedenkend vind ik. En ook wel meedenkend van mijn kant uit. Dat denk ik wel. Als >BEDRIJFSLEIDER< iets uitzet of iets doet, dan betrekt >BEDRIJFSLEIDER< daar zeker bij en ik uiteindelijk weer het team als dat nodig is.

**Interviewer:**	Is het ook duidelijk voor jou welke dingen er moeten gebeuren? Heb je als teamleider eigenlijk veel vaste verplichtingen?

**Respondent:**	Ja. Hoe moet ik dat nou zien. Je takenpakket bedoel je?

**Interviewer:**	Ik zal je een voorbeeld geven. Het jaargesprek. Die horen erbij.

**Respondent:**	Ja. Die moeten. Daar ben ik verplicht aan.

**Interviewer:**	En je weet ook 'voor het einde van het jaar moet dat'...

**Respondent:**	Moet dat klaar zijn. Ja.

**Interviewer:**	Zijn er nog meer van dat soort vergelijkbare dingen?

**Respondent:**	Die moeten. Ja. Ik kan me dat niet zo bedenken ad hoc. Alles wat ik benoemde moet vind ik eigenlijk.

**Interviewer:**	Hoe houd je dan het overzicht over al je taken en verantwoordelijkheden?

**Respondent:**	Kijk ik heb een jaarplanningagenda. Dit ligt hier onder jouw telefoon. *Respondent lacht.* Het is zo dat ik als volgt heb georganiseerd. Kijkend naar bijvoorbeeld mijn jaarplan of bekijken wat ik wil behalen en moet doen, zet ik zelf gewoon deadlines 'tot dan en dan wil ik dat en dat bereikt hebben en dan en dan wil ik dat en dat hebben behaald'. Bijvoorbeeld het teamoverleg moet zes keer per jaar gehouden worden en zet ik al voor het hele jaar uit. Dat is nu al voor het hele jaar uitgezet met ruimtes erbij. Niet de agenda. Dat is een beetje afhankelijk van wat je wil bespreken. Dit soort dingen zet ik gewoon uit. De jaargesprekken heb ik nog niet uitgezet maar ben ik mij zeker van bewust dat ik voor september de planning heb gemaakt. Dus dan de zomer wil dat. Ik heb, het team hier is niet zo groot. Sommige, bijvoorbeeld als verloskunde, die hebben zoveel mensen die moeten het hele jaar door plannen denk ik. Dat heb ik niet dus eind van het driekwartjaar begin ik met de jaargesprekken. Dat houd ik nu zo aan omdat ik dat vorig jaar ook zo heb gedaan, dan zit er precies een jaar tussen. Maar als er bij een jaargesprek bijvoorbeeld uit komt dat je een onvoldoende beoordeling geeft, dat heb ik ook voor handen en nu, dan doe je dus tussentijdse evaluatie. Alles wat je aan planningen hebt. Bijvoorbeeld nu. Ik heb dan, met jou dus niet, maar er zijn weer afspraken komen daar uit voort. Daar plan ik weer een deadline voor. Die komen allemaal in de agenda. Zo kan ik het overzicht ook houden, wanneer ik precies weet wanneer ik wat af moet hebben. En hoeveel tijd ik nog heb.

**Interviewer:**	Hoe ben je op de hoogte dan van dat soort dingen? Net zoals je zegt, die teamgesprekken moeten zes keer per jaar. Dat is echt zo'n dingetje...

**Respondent:**	Dat zijn ook wel dingen die je uit de verschillende portalen haalt. Dat is ook iets wat een beetje voortvloeit uit de ervaringen die je hebt opgedaan in de loop der jaren. Dat je weet dat dat bij jouw taken hoor. Maar dat haal ik ook van internet, of intranet moet ik zeggen, en dingen die je ziet verschijnen in >INTERN MAGAZINE<. Daar komt natuurlijk ook het een en ander aan dingen, of soms een reminder dat ik denk 'o jeej, daar moet ik ook nog even aandacht aan besteden'. Nieuwsbrieven die ik eigenlijk wekelijks wil versturen, maar die verstuur ik niet om te versturen. Die moeten ook relevant zijn qua inhoud, dat kan soms ook wel eens twee weken duren. Het is eigenlijk gewoon iets opgeslagen in je hersenen dat je gewoon weet, maar ik heb het wel omgezet in een agenda. Want ik vind het iets dat organisatorisch bij mij altijd vrij lastig, ik heb altijd overal briefjes en overal dingen. Ik vind het eigenlijk overzichtelijker allemaal in een agenda te zetten.

**Interviewer:**	Duidelijk. Heb je als teamleider ook nog het idee dat je bepaald gedrag moet uitstralen?

**Respondent:**	Ja. Ik vind dat je zeker een. Hoe noem je dat. Dat iemand zich niet hoeft te spiegelen. Hoe noem je zoiets?

**Interviewer:**	Voorbeeldfunctie?

**Respondent:**	Voorbeeldfunctie. Ja. Ik ben Duitser. Soms weet ik de woorden niet zo goed. Een voorbeeldfunctie uiteraard. Ik kan me voorstellen dat mensen zich ook een beetje aanpassen aan je eigen gedrag. Dus als jij een chagrijnig iemand bent of alles maar ad hoc doet of alles maar. Dan denken zij ook van 'nou, dan doen wij het ook zo ook niet'. Je moet echt wel een goed voorbeeld geven om uiteindelijk te kunnen verwachten dat het team ook zo reageert. Dan moet je alleen duidelijk hebben of jij wel het goede voorbeeld bent he als teamleider?

**Interviewer:**	Heb je dat goed duidelijk voor jezelf?

**Respondent:**	Ik denk voor mezelf wel maar ik heb daar geen toetsmoment voor of ik kan, ik laat me, aan de mensen zelf, dat je feedback krijgt. Maar ook aan je directe leidinggevende die zich >TEAMLEIDER< eigenlijk vind ik dat hoe jij die dingen aanpakt helemaal niet goed. Of dat je daarin sturing krijgt of dat je zelf denkt van 'hier ben ik een beetje zwak in'. Laten we eens kijken of er via Opleidingen iets wordt aangeboden op het gebied van communicatie of zo. Dat je die wegen kunt bewandelen. Maar ik toets mezelf niet. In die zin. Dat is gevoel wat je hebt. Je denkt dat het goed gaat. Je hoopt dat het goed gaat. En je ziet natuurlijk wel resultaten dat je zegt van 'volgens mij gaat het op die manier wel goed'.

**Interviewer:**	Je hebt er wel een positief gevoel bij hoe het nu gaat?

**Respondent:**	Ja.

**Interviewer:**	Heb je het idee dat er ook nog veel verschillen zijn tussen teamleiders hier in huis?

**Respondent:**	Oh ja.

**Interviewer:**	Waar moet ik dan aan denken?

**Respondent:**	De manier van leiderschap. Ja. Dat is dan alleen maar omdat ik, zeker uit mijn vorige leidinggevende positie, natuurlijk ook veel te maken heb gehad met andere teamleiders en leidinggevenden. Ook in overleggen en in die overleg hoor je natuurlijk hoe iets aangepakt moet worden, hoe mensen iets aanpakken. Dan denk ik 'dat zou niet mijn weg zijn'. Ja. Dat zeker. Dat mensen een hele andere leiderschapsstijl hebben een hele andere manier van benaderen hebben waar ik mij niet prettig bij voel.

**Interviewer:**	Zou er meer gelijkheid moeten komen tussen teamleiders?

**Respondent:**	Dat vind ik moeilijk te zeggen.

**Interviewer:**	Zoals je zegt 'iedereen heeft een eigen manier van aanpakken', is dat een goed iets naar jouw idee?

**Respondent:**	Dat vind ik wel iets om over na te denken. Kijk ik kan niet verwachten dat iemand zoals ik ben, dat de ander ook zo is. Dat is een beetje moeilijk he? Ik denk dat je in grove lijnen waar je als je je functieprofiel bijhoudt waar een teamleider aan moet voldoen, denk ik als die taken worden gedaan en je boekt resultaten, is dat denk ik, dan maakt het niet uit op welke manier je dat doet. Maar als je ziet dat iemand dat probeert te doen, en het lukt niet, dan is het misschien iets waarvan je denk 'diegene moet misschien eens aangesproken worden of bijgestuurd worden of zich aan moeten passen aan een ander soort'. Daar geef je mij stof om over na te denken. Moet iedereen hetzelfde zijn? Ik denk het niet.

**Interviewer:**	Het afdeling en het werk dat bepaalt? Het zijn de resultaten die behaald moeten worden?

**Respondent:**	De resultaten moeten wel kloppen. Je moet je taakfunctieomschrijving erbij houden en denken 'voldoe ik hier aan?' en als je dat via weg A voor elkaar krijgt en de andere via weg B, dan maakt eigenlijk niet zo veel uit denk ik, als je je doelen bereikt.

**Interviewer:**	De taakfunctieomschrijving is voor jou een best wel belangrijk middel dan om te weten wat jouw functie precies inhoud?

**Respondent:**	Dat denk ik wel. Daar kijk ik ook geregeld na. Of ten minste geregeld naar, dat is wel fijn om daar even naar te kijken 'hoe zit dat in elkaar en waar moet je aan voldoen en wit zijn je taken?'.

**Interviewer:**	Een goede houvast?

**Respondent:**	Ja. Vind ik wel.

**Interviewer:**	En dan heb je je  MO waar je veel informatie uit haalt als het aankomt op jouw functioneren, hoe tevreden je medewerkers zijn?

**Respondent:**	Wat is MO?

**Interviewer:**	Het medewerkerstevredenheidsonderzoek?

**Respondent:**	Oh. Ja dat. Als afkorting MO. Ja. Dat vind ik ook een heel mooi instrument ja.

**Interviewer:**	En dan heb je je jaarplan die ook veel duidelijkheid schept van 'wat moet ik wanneer doen'?

**Respondent:**	Ja.

**Interviewer:**	Dan heb ik het wel duidelijk.

*Einde transcript*

# Transcript Interview FLM 9

**Duur interview:	30 minuten en 3 seconden**

**Functie:	Teamleider**

**Afdeling(en) onder supervisie:	Support**

**Gender:	Man**

**Werkervaring in functie:	1,5 jaren**

**Interviewer:**	Sinds wanneer ben je teamleider?

**Respondent:**	Phoe, phoe, phoe, dat is een goeie vraag. Ik ben sinds anderhalf jaar gok ik.

**Interviewer:**	Oke. Duidelijk. Als teamleider, wat maakt het anders dan dat je hiervoor als coördinator deed?

**Respondent:**	Voor mij inhoudelijk niet zo heel veel. Want als coördinator deed ik al dezelfde dingetjes als dat ik als teamleider deed. Dus voor mijzelf maakte dat niet heel veel uit. Ik kan me zo voorstellen dat het voor anderen die daar coördinator zijn wel een verschil in zat. Maar ik deed bijvoorbeeld al jaargesprekken en ik beheer al budgetten. Ik weet niet andere teamleiders dat ook doen. Maar dat deed ik toen ook al.

**Interviewer:**	Dus eigenlijk is er alleen maar een financiële bijdrage bijgekomen?

**Respondent:**	Ja. Zo moet je het wel zien. En dat staat dan weer niet hetzelfde als andere teamleiders. Ik heb begrepen, ik zit anders ingeschaald dan anderen. Waarom dat is weet ik niet. *Respondent lacht*. Maar dat schijnt wel zo te wezen. Dus ja voor mij het inderdaad alleen en als teamleider heb je toch net wat meer zeggenschap dan dat je als coördinator natuurlijk heb.

**Interviewer:**	In wat voor gevallen merk je dat dan dat je dan meer zeggenschap hebt?

**Respondent:**	Je mag wel wat meer in beslismomenten meepraten denk ik. Ook naar mijn team toe als coördinator had ik dan wel de coördinerende functie maar was ik wat meer op mijn manager aangewezen als dat ik dan dat ik dat nu eigenlijk ben.

**Interviewer:**	Dus ook meer verantwoordelijkheid nu ook?

**Respondent:**	Buiten het feit dat ik toch die verantwoording al had. Maar die stond toen nog niet op papier, maar die heb ik nu wel. Maar nu wordt natuurlijk ook verwacht dat je meer die verantwoordelijkheid pakt en dat was voorheen iets anders omdat ik toen iemand boven mij had zitten die mee moest beslissen.

**Interviewer:**	En werkt dat voor jouw team nu heel anders? Merken zij het verschil?

**Respondent:**	Nee. Ik denk niet dat ze het verschil. Wel dat de lijntjes nog iets korter zijn als dat ze het eerst aan mij vroegen. Nu kan ik ook de beslissing ten dele nemen. Dat kon vooraf niet. Daarvoor moest ik ook bij mijn manager zijn om toestemming te vragen. En ja nu is dat minder gewoon. Zonder meer is dat minder.

**Interviewer:**	Ze hebben nu niet het idee dat er iemand hiërarchisch boven hun staat?

**Respondent:**	Nee. Ja minder. Nee. Omdat dat daarvoor natuurlijk ook al was. Die overstap voor hun is heel minimaal geweest omdat ik die als coördinator ook al ten dele overnam. Er is niet veel verschil.

**Interviewer:**	Oke. En als teamleider. Hoe ziet je dag eruit? Wat je doe je zowaar?

**Respondent:**	Ja. Goh. Ja. Dat verschilt een beetje per dag. Ik zit bij heel veel overleggen en een teamleider ondersteunende dienst is wat anders dan een zorgteamleider denk ik.

**Interviewer:**	In wat voor opzicht?

**Respondent:**	Nou. Wat wij doen is een andere tak van sport dan als wanneer je met zorg bezig bent natuurlijk. >AFDELING< is iets anders dan zorg. Ik zit best wel veel in overleg. Ik word veel aangesproken op de hal door mensen die dingen willen, die bedacht moeten worden en zo. Ik ben natuurlijk ook met mijn personeel bezig die ik aan de gang moet houden. Het is best breed. Maar ik vind het erg leuk. En ik werk voor x procent ook nog mee. Ik werk voor 25 procent ook nog gewoon mee.

**Interviewer:**	Wat vind je daarvan, van dat meewerken? Is dat een goed iets?

**Respondent:**	Ik denk juist dat het heel positief uitwerkt naar de mensen die ik onder mij heb. Mij maakt het niet uit of ik een doosje moet open of weggooien of verscheuren. Ik doe in die 25 procent eigenlijk exact hetzelfde als wat hun doen.

**Interviewer:**	Juist. En die 25 procent. Te veel, te weinig, precies goed?

**Respondent:**	Ik denk nu voor het moment dat het te veel begint te worden. Doordat wij zo veel in overleg zitten, ik zit bijna overal bij. Verbouwing van de OK, zit ik bij, noem maar op. Alles wat iets te maken heeft met >AFDELING< en waar computers bij komen kijken, ja daar zit ik inmiddels wel bij. En omdat dat best veel is en snel gaat. Die ontwikkeling in de zorg gaat heel snel. Dat ik dat eigenlijk 25 procent meewerken te veel is. Dus ja, of ik dat dagelijks haal of wekelijks haal weet ik niet.

**Interviewer:**	Oke. En dat aantal overleggen. Merk je dat dat nu meer geworden is nu je teamleider bent of was dat ook als coördinator al zo?

**Respondent:**	Daarvoor was het ook al zo. Ik beheer natuurlijk een aparte club van de Helpdesk. Het is wel meer geworden. Ja. Heel veel meer. Het is wel ietsje meer geworden. Maar daarvoor zat ik ook al eigenlijk overal bij. Ik heb ook managementoverleggen, ik heb overleggen met de projectleiders, ik zit bij de eerste gesprekken van projecten, waarna ik ze weer door kan zetten naar een van de jongens.

**Interviewer:**	Je noemde net al een stuk 'het managen van personeel'. Wat valt daar onder voor jou?

**Respondent:**	Wat valt daar onder voor mij? Dat vind ik een hele goeie vraag. Dat is best breed, vind ik, denk ik. Voor mij is het belangrijkste dat de dingen die gedaan moeten worden dat die aan het einde van de dag gedaan zijn. En daar zijn ze opzicht vrij in, buiten het feit dat ik wil dat op bepaalde tijden dingen gebeuren. Dat moet dan ook worden gebeurt.

**Interviewer:**	Ben jij dan ook degene die een soort planning maakt?

**Respondent:**	Wij werken met een vaste planning. Wij zitten natuurlijk met de telefoon die moet altijd bezet zijn. Wij moeten hier om acht uur zijn en om vijf uur gaat die telefoon uit. Maar in de tussentijd moet het niet zo zijn dat er niemand aan die telefoon zit. We hebben een roostertje die wisselt per dag, zodat ze allemaal ongeveer hetzelfde doen. We hebben wel een paar medewerkers die doen andere dingen erbij. Dat is ook omdat, dat is hun kennisgebied. Ze werken via een rooster. De ene dag hebben ze de telefoon, de andere dag lossen ze storingen op, de andere dag zijn met een project bezig, zijn de projectmember waar ze dingen voor moeten doen. Het is heel wisselend. Ik denk ook dat ze dat heel fijn vinden. Dat ze niet alleen maar de telefoon hoeven op te nemen.

**Interviewer:**	Heb je ook het idee dat er een stuk HR bij jou ligt als teamleider?

**Respondent:**	Voor het stukje jaargesprekken wel ja.

**Interviewer:**	En verder dan dat?

**Respondent:**	Goedkeuren van de verloven doe ik. Dus dat is ook weer een stukje HR. Als ze iets willen. Ziektemeldingen. Ja. Ik denk wel van A tot Z. Want als mensen vrij moeten, dat is natuurlijk ook een stukje HR. Dat gebeurt natuurlijk ook.

**Interviewer:**	Ja. Zeker. Zie jij hier nog onderwerpen tussen staan waar jij deel aan neemt als teamleider?

**Respondent:**	Werving en selectie. Ja. En de introductie van een nieuwe medewerker doe ik als er iemand binnenkomt. Bijhouden van personeelsbestanden met betrekking tot je jaarverslagen doe je. Beslissen en plannen van training en ontwikkelingsbehoeften van medewerkers doe ik.

**Interviewer:**	Maak jij dan ook het opleidingsplan?

**Respondent:**	Ja. Samen met mijn manager. Die gaat over de budgetten. Dus die gaat over dat budget. Ik heb zelf dat budget niet. Dus dat doen we samen. Ja. Geven van informele training. Ja. We geven natuurlijk heel veel uitleg, maar dat doen we eigenlijk allemaal, uitleg aan de eindgebruikers. Ik probeer wel een beetje te mentoren. Nou, functioneren dat staat er al op. Ontwikkelplannen. Dat is natuurlijk onderdeel van de training die ze volgen. Disciplineren en corrigeren doen we waar nodig. Maar gelukkig is het niet nodig. Tuchtprocedures en klachtenprocedures. Het is het weinig. Maar het mocht nog wel eens voorkomen dat wij een DIM-meldingkje krijgen en een klachtmelding krijgen waar je iets mee moet doen. Nou ja, absentiemanagement, waarderen. Dat zit weer in het jaargesprek over het algemeen.

**Interviewer:**	En aan wat voor manier moet ik dan aan waarderen denken? Hoe pak je dat aan?

**Respondent:**	Dat ze goed hun werk doen. Als er de mogelijk is, dat je ze een eenmalige bonus geeft omdat ze extra werk hebben geleverd boven op het werk dat ze hoeven te doen. We doen natuurlijk bottom-up en top-down communicatie dat komt omdat ik eigenlijk de schil ben tussen de managementlaag en de medewerker.

**Interviewer:**	Hoe pak je dat aan, dat communiceren?

**Respondent:**	Ik communiceer zo'n beetje alles. Er zijn natuurlijk dingen die niet voor iedereen bestemd zijn, maar over het algemeen communiceer ik alles met ze omdat ik vind dat je open met elkaar om moet gaan. Dat ik voor mij fijn maar dat is voor hun ook fijn. Het reageren van suggesties van personeel doen we. Dat is voor ons heel belangrijk natuurlijk.

**Interviewer:**	En op wat voor manier krijg je dat dan binnen, dat soort suggesties?

**Respondent:**	Nou. Wij werken bijvoorbeeld met templates om bepaalde zaken sneller te laten verlopen. We zitten natuurlijk met HR hebben we heel erg nauw het hele accountproces. Als iemand in dienst komt, wil je natuurlijk ook in kunnen loggen. Als daar dingetjes in verbeterd kunnen worden, dan bespreken we die met elkaar en zetten we de verbeteringen door. Het werk van teams en ploegen, dat heb ik net al gezegd. Dat is het wel zo'n beetje. Het hele lijstje.

**Interviewer:**	Dat klinkt dan alsof er een groot stuk personeelsmanagement bij jou ligt dan?

**Respondent:**	Ja. Maar ik denk ook dat het voor een deel bij de medewerker zelf ligt. JE bent natuurlijk ook zelf verantwoordelijk voor het vragen van als je bepaalde opleidingsbehoeften hebt en zo. Ik denk ook dat je het kenbaar moet maken, dat je het graag wil. Er zijn natuurlijk ook mensen die zeggen 'ik vind het wel best zo'.

**Interviewer:**	Stuur je daar ook actief op?

**Respondent:**	Ik wil wel dat ze bepaalde cursussen doen. Dat moet gewoon. Die markt verandert zo snel. Je bent zelf denk ik student? Dus je merkt zelf wel hoe nodig jij geautomatiseerde systemen hebt tegenwoordig. Laptop. Nou ja, je ziet het al, telefoon. Zonder telefoon kan je al niet meer bij wijze van.

**Interviewer:**	Maar ben jij dan degene die zegt 'ik wil dat jij nu naar die cursus gaat'? Of gaat het juist andersom?

**Respondent:**	Soms vragen ze er naar of ze er een cursus voor kunnen krijgen, dan ga ik kijken of ik dat kan regelen. Er moeten wel de mogelijkheden voor zijn en je werk natuurlijk met een heleboel op basis van 'je moet het dit jaar aanvragen, dan krijg je volgend jaar de cursus'. Maar het zou natuurlijk best zo kunnen zijn dat wij iets nemen waar zij extra moet. Dan is het wel de vraag 'kom het van medewerker of' uh. Dan ga ik wel proberen om te regelend dat ze daar heen kunnen. Maar het is ook andersom dat ik wil dat ze naar bepaalde cursussen gaan. Wij gebruiken ITEL als principe binnen de IT en ik wil allemaal dat ze die cursus volgen. Dat ze ongeveer allemaal hetzelfde hebben. Ik probeer het dan ook nog een beetje evenredig te verdelen. Dat als de ene het ene jaar wel een hele uitgebreide cursus heeft gehad dat hij dan niet de volgende jaren er weer een krijgt. Dat is een beetje wikken en wegen, maar dat is budgettair natuurlijk dus ik probeer dat eerlijk en evenredig te verdelen.

**Interviewer:**	En als we kijken naar jou bevoegdheden. Over wat voor dingen zou jij allemaal mogen beslissen als teamleider?

**Respondent:**	Dat is een hele goede. Voor mij is dat vrij breed. Ik heb weinig vergelijkingsmateriaal met andere teamleiders. Ik doe natuurlijk een stukje budgettering. Als er personeel nodig is dan kan ik daar over beslissen, dus inhuren van uitzendkrachten kan ik over beslissen. Moet ik natuurlijk wel in samenspraak met mijn manager doen. Eigenlijk over alles wel kan ik beslissingen nemen.

**Interviewer:**	Enig idee waar je geen beslissingen over kan nemen?

**Respondent:**	Nee. Dat is een hele goeie. Nee. Daar ben ik nog niet tegen aan gelopen?

**Interviewer:**	Maar het gaat wel vaak in overleg?

**Respondent:**	Ik doe wel vaak dingen in overleg. Dat komt ook gewoon, ik heb wel wat budget, maar dat is meer hardware budget. Het personele budget dat ligt dan eigenlijk. Ik doe dan wel de verloven, de jaargesprekken en de dingen. Maar het echte personele budget voor mijn team, dat ligt bij mijn manager. Ik zal altijd >HOOFD< nodig hebben voor dat budget.

**Interviewer:**	Dat personele budget, waar moet ik dan aan denken. Waar wordt dat aan uitgegeven?

**Respondent:**	Wat zeg je?

**Interviewer:**	Dat personele budget, waar wordt dat aan uitgegeven?

**Respondent:**	Nou dat is een deel opleidingen zit er als het goed is in. Sommigen hebben nog een jaarlijkse verhoging vanuit de functie die ze hebben. Dat je jaarlijks klimt zeg maar. Dat soort dingen zeg maar, maar dat gaat allemaal via mijn manager.

**Interviewer:**	Komt het wel eens voor dat jij een bosje bloemen koopt voor een personeelslid of iets anders klein?

**Respondent:**	Als die binnen komt?

**Interviewer:**	Ja. Als die binnen komt of een collega die al wat langer aan het werk is. Van die kleinere personeelsuitgaves?

**Respondent:**	Ja. Maar dat regelen we meestal zonder dat daar uitgaves aan gedaan moeten worden. We draaien allemaal diensten en natuurlijk komt daar wel een oude apparatuur. Nou ja, dat kan ik of weggeven dat het in de verkreukelaar komt. Ik kan ook wel eens zeggen van 'nou jongen, dat we dan gezamenlijk', dat vraag ik aan mijn Hoofd dan. Het is een keer voorgekomen dat ik die jongens niet een oude laptop mee kon geven, zodat het makkelijk voor hun wordt om diensten te draaien. Dat soort dingetjes. We kopen ook wel eens een x aantal stikkies of een keer koptelefoons. Maar dat is eigenlijk >AFDELING<-breed.

**Interviewer:**	Oke. Maar ben jij dan degene die dat gewoon zelf kan kopen, of vraag je dan eerst nog even toestemming bij...

**Respondent:**	Nou. Voor de kleine dingetjes. Stel, dat we dat voor tijdens ons werk ook nodig hebben. Zoals USB-stickjes, daar beslis ik zelf dan over. Wordt het wat groter, dan wordt dat gewoon besproken.

**Interviewer:**	Enig idee waar voor jou de grens ligt? Of is dat puur gevoelsmatig?

**Respondent:**	Dat is gewoon gevoelsmatig. Als ik 2.000 3.000 euro moet uitgeven om een cadeautje weg te geven op de afdeling. Dat is gewoon een onderbuikgevoel denk ik. Ik vind ook niet dat ik daar zo maar over kan beslissen.

**Interviewer:**	Heb jij ook voor jezelf goed duidelijk wanneer jij als teamleider goed functioneert?

**Respondent:**	Nou ik denk als mijn manager daar tevreden over is.

**Interviewer:**	Zo lang je geen...

**Respondent:**	Nee. Want ik denk zo lang mijn groep mensen, als die tevreden over mij zijn en degene die boven mij staat tevreden over mij is, dan denk ik dat je goed functioneert.

**Interviewer:**	Hoe peil je zoiets?

**Respondent:**	Dat wordt gepeild in je jaargesprekken en ja, als er heel veel aan je wordt gevraagd en je doet bepaalde zaken niet of mijn medewerkers geven aan van 'hij doet niet wat ik vraag' of...

**Interviewer:**	Dan krijgen ze het of van boven of van beneden wel te horen?

**Respondent:**	Van boven of van beneden. Ik ben heel open. Als er wat is, vind ik dat ze dat aan moeten kunnen geven. Zo ben ik ook. Ik ben best heel direct. Voor sommigen iets te veel, maar ik vind dat prettig. Als ik wat heb, dan zeg ik het en ik verwacht van hun ook dat ze dat doen. En dat doen ze ook over het algemeen.

**Interviewer:**	Heb jij het idee dat jij als teamleider ook nog bepaalde dingen moet weten om goed te kunnen functioneren,  bepaalde kennis moet bezitten?

**Respondent:**	Ja. Je heb altijd bepaalde kennis die je moet bezitten, maar de meeste handvatten, daar ben ik een jaar op cursus voor geweest. Daar hebben we wat voor geleerd, in een team van zeven acht mensen zijn we een jaar lang op teamleiderscursus geweest. Dan krijg je natuurlijk wat handvatten aangereikt. Of voor mij op dat moment de juiste waren weet ik niet, want ik denk wel dat het heel belangrijk is, omdat je natuurlijk zeggenschap over een stukje personeel hebt, dat dat meer inhoudelijk naar het personeel toe zou moeten zijn. Bijvoorbeeld 'wat moet ik als ik een nieuwe medewerker krijg?'. 'wat moet ik doen als ik een uitzendkracht in wil huren?'. Dat zijn handige handvatten. Ik kom er nu wel achter, gewoon, ik vraag het gewoon. Maar dat waren voor de cursus handigere handvatten geweest om dat er in ieder geval bij te doen, om het in ieder geval een stukje aan te stippen tijdens de cursus.

**Interviewer:**	Dus hoe je zoiets aanpakt als zoiets plaatsvind? Wat de regels zijn?

**Respondent:**	Ja. Natuurlijk. Als je een medewerker bent en je wordt bijvoorbeeld teamleider, ja, dan krijg je natuurlijk hele andere verantwoordelijkheidsgebieden en dan is het ook wel handig als je weet hoe je dat in moet vullen. Natuurlijk, je kunt het zelf uitzoeken. Maar het ook handig om te weten van 'hoe moet ik dat aanpakken en hoe moet ik dat doen?'.

**Interviewer:**	Had je dat ook voor jezelf, dat je dus niet wist hoe je bepaalde dingen moest invullen?

**Respondent:**	In het begin wel ja. Het aannamebeleid van het ziekenhuis bijvoorbeeld. Hoe kun je nieuw personeel werven, welke stappen moet je nemen. Mag je dat vanuit jezelf doen of moet er een personeelsadviseur bij zitten? Die stappen weet ik nu inmiddels. In den beginne was dat nog wel even zoeken en had gehoopt dat de cursus die wij deden van een jaar, dat daar ook dat soort dingen in besproken werden.

**Interviewer:**	Die cursus zou dus als het ware alle processen moeten verduidelijken?

**Respondent:**	Ik denk wel dat het handig is om te weten 'hoe werkt dat nou hier in het ziekenhuis?'.

**Interviewer:**	Je bent er uiteindelijk dus achter gekomen door het maar gewoon te gaan vragen? Van 'goh, hoe werkt dit'?

**Respondent:**	Ja. Hoe zit het een en ander in elkaar?

**Interviewer:**	Heb je ook duidelijk voor jezelf waar jou verantwoordelijkheid als teamleider eigenlijk ophoud, waar jij niet over. Waar het ophoud?

**Respondent:**	Dat is een goeie. Weet ik eigenlijk niet. Ik denk dat ik altijd wel een bepaalde soort verantwoording heb.

**Interviewer:**	Dus ook als ik, je noemde net al een stukje budget, dat is iets waar je niet over gaat?

**Respondent:**	Dat is niet helemaal, ik heb natuurlijk wel een heel groot hardware budget. Dus ik heb wel een vijf ton aan euro's voor het uitgeven aan spullen. Daar ligt wel degelijk een hele grote verantwoording denk ik.

**Interviewer:**	Enig idee waar het anders op zou kunnen houden. Bijvoorbeeld zoals echt meerjarenbeleid, ben je daarin betrokken?

**Respondent:**	Niet voor het ziekenhuisbrede meerjarenbeleid, wel voor het >AFDELING<. Zijdelinks wordt ik daar wel in betrokken. Wij moeten natuurlijk moet omdat wat zij bedenken wij moeten ondersteunen.

**Interviewer:**	Je zegt als 'gaandeweg ben ik aan het ontdekken hoe ik dat stukje HR moest doen, door het gewoon te vragen'. Maar, is het ook voor jou duidelijk wanneer alles gedaan moet worden qua personele aangelegenheden. Denk bijvoorbeeld aan de jaargesprekken.

**Respondent:**	Ja. Ja. Ja. Wij >AFDELING<ers zijn er heel slecht in he?

**Interviewer:**	Oh ja?

**Respondent:**	Zodat het echt administratie moet worden zijn wij echt. Natuurlijk weet ik dat het gebeuren moet. Maar wij hebben een beperkte groep. We hebben het altijd vrij druk en het eerste dat er dan een beetje in gaat sluipen is natuurlijk, de jaargesprekken doen we. De administratieve afhandeling, dat neemt bij ons altijd wel even de tijd. Het is niet goed, dat weet ik. Het kan zijn dat ik op een gegeven moment de keuze maak 'zal ik nu concretiseren of ga ik eerst doen wat heel belangrijk is om de zorg door te laten gaan?

**Interviewer:**	Zijn er nog meer van dat soort dingen? Zoals jaargesprekken, dat is iets dat ieder jaar gedaan moet worden. Zijn er meer van dat soort dingen naar jou idee?

**Respondent:**	Nee. Eigenlijk niet.

**Interviewer:**	Vooral de jaargesprekken dus?

**Respondent:**	De jaargesprekken is wel een ding. Het is best veel werk. Je moet het gesprek voeren, daar komen dan weer dingen uit, die moet je dan uitschrijven. Dat wat je uitgeschreven hebt moet de medewerker dan eigenlijk weer lezen, die kan daar wat van vinden natuurlijk. Het zou kunnen dat als hij daar wat van vind dat jij dat moet aanpassen. Ik heb er gelukkig maar vijf. Er zijn ook mensen die van januari tot december bezig zijn. Daar zou ik persoonlijk niet blij van worden.

**Interviewer:**	Heb je het idee dat jij als teamleider ook nog bepaald gedrag moet uitstralen?

**Respondent:**	Ik denk dat. Ik ben 'een van de jongens'. En dat zou wat minder moeten zijn. Maar aan de andere kant, als je 'een van de' bent, weet je ook eerder wat er speelt denk ik?

**Interviewer:**	Oh, ja?

**Respondent:**	Dat ik wel. Ik kan wel heel strak gaan zitten van 'ik ben jullie baas'. Ik weet nou niet of dat altijd de juiste keuze is. Met elkaar moeten we het allemaal zien te rooien. Natuurlijk weten ze allemaal wel, dat als het zo ver is dat ik op mijn strepen ga staan.

**Interviewer:**	Is dat geen moeilijk spel?

**Respondent:**	Daar heb ik geen problemen mee.

**Interviewer:**	Kun je onder woorden brengen hoe je zoiets aanpakt?

**Respondent:**	Ik denk als er echt iets moet gebeuren dat ik dan gewoon zeg dat het moet gebeuren. Dan kunnen ze daar een andere mening over hebben, maar wat moet dat moet. Dat heb ik ook vanuit mijn manager zo. Wat moet dat moet.

**Interviewer:**	Dan is het een kwestie van doordrukken?

**Respondent:**	Dan is het een kwestie van doordrukken. Sommige dingen moeten gewoon en het is niet anders. Je kunt veel dingen doen op een vreedzame manier, er zijn ook wel eens dingen die gewoon moeten, die mensen niet leuk vinden. Maar het is niet anders. Soms moeten dingen gewoon. Dat heb je thuis ook, soms moeten dingen.

**Interviewer:**	Nee zeker. Maar eerder omdat je zo midden in het team staat, dat klinkt lastig?

**Respondent:**	Soms is het lastig. In het begin had ik daar best moeite mee, maar je merkt gewoon, daar groei je gewoon in. Zij merken natuurlijk ook, ooit was ik helemaal een van de jongens. Dat ebde langzaam weg omdat ik een coördinerende rol kreeg. Dan krijg je alweer wat andere verantwoordingsgebieden, je zit meer in overleggen. En nu is dat helemaal anders en nu snappen ze ook wel 'als het echt moet, dan moet het en is hij serieus'.

**Interviewer:**	Maar jij hebt dus ook zoiets van 'laat mij daar maar gewoon tussen zitten, het is prima zo'?

**Respondent:**	Ik moet daar tussen zitten. Het is een beetje fingerspitzengefühl en je moet natuurlijk ook allemaal oppakken wat er speelt en wat er bezig is in huis. Het zou best kunnen dat wij dingen moeten escaleren omdat er iets stuk is dat sneller gefixt moet worden. Dus ik denk ook zeker dat ik tussen de mensen moet blijven zitten. Ik zou ook niet in een kantoortje apart willen zitten. Ik wil gewoon feeling met de werkvloer houden.

**Interviewer:**	Heb jij ook nog het idee dat jij als teamleider bepaalde dingen moet kunnen, bepaalde competenties moet bezitten?

**Respondent:**	Ik denk dat je competitief heel goed moet zijn. Zeker met de top-down en bottom-up communicatie goed moet hebben. Om het te delen, maar ook op te kunnen vangen. Ik denk dat dat zeker heel belangrijk is. Wij zijn natuurlijk heel erg open voor het huis. Iedereen kan bij ons langs komen en kunnen altijd dingen vragen. Ik denk dat het communicatief, dat je wel heel goed moet wezen.

**Interviewer:**	Nog meer dingen waarvan je denkt, dat is wel handig om te kunnen?

**Respondent:**	Ja. Omgaan met budgetten omdat je een financiële verantwoordelijkheid hebt. Je kunt natuurlijk een PC kopen die 1.000 euro kost, maar je kunt er bijvoorbeeld ook twee voor 1.000 euro kopen. Dat je ook een beetje een financieel inzicht hebt. Ik vind dat met het geld dat je krijgt dat je daar heel goed mee om moet gaan. Daar moet je het maximale uit zien te halen. Dat is natuurlijk ook een spel tussen mij, inkoop en degene die het aanvraagt. Wij proberen altijd voor het bedrag dat ze gereserveerd hebben, daar zo maximaal mogelijk de spullen voor aan te schaffen.

**Interviewer:**	Juist. Het is eigenlijk heel snel gegaan, maar ik heb eigenlijk geen verdere vragen meer.

**Respondent:**	Ik ook niet. Dus ik ben geslaagd? Ik mag nog een jaartje blijven?

**Interviewer:**	Is het een leuke functie de functie van teamleider?

**Respondent:**	Voor waar ik zit, in mijn vak, wel.

**Interviewer:**	Ja? Als je hier niet had gezeten, was het dan anders geweest denk je?

**Respondent:**	Dat weet ik niet. Ik vind het voor het stukje dat ik doe, >AFDELING< Helpdesk, dat vind ik leuk. Ik heb ook andere functies gehad. Ik ben ook beheerder geweest. Dat zijn dingen die ik nooit meer zou hoeven doen.

**Interviewer:**	Dus het heeft ook echt wel te maken met je vakgebied, waar je passie voor hebt?

**Respondent:**	Ja. Ik vind het gewoon echt heel leuk om mensen te helpen en ik vind het leuk om problemen uit te zoeken. Dit is gewoon mijn ding, ik vind dit leuk.

**Interviewer:**	Super tof?

**Respondent:**	Ja. Voor mij wel ja. Ik vind het een geweldige functie en ik vind het zelfs jammer dat die 25 procent dat ik daar wat minder, dat ik wat verder van de techniek raak. Dat vind ik wel jammer.

**Interviewer:**	Dus die 25 procent in stand houden, dat zou wel een goeie zijn?

**Respondent:**	Ja. Dat wordt gewoon met de werkzaamheden steeds moeilijker. Ik moet ook processen uitdenken, ik doe ook incidentmanagement erbij. Als jij mij belt, dan moet het bijvoorbeeld niet een half jaar duren voordat jij geholpen wordt. Dat soort dingen, maar dat moet je allemaal in de gaten houden. En dat is best veel werk. En als je ook nog de jongen moet aansporen. En gelukkig zijn ze heel erg zelfstandig, maar er zijn ook dagen dat je moet zeggen 'he mannen, aan de slag'.

**Interviewer:**	Ja, precies. Maar het zou dus wel een goede zaak zijn om ervoor te zorgen dat die teamleiders toch mee blijven werken als het ware?

**Respondent:**	Ik denk dat dat opzicht niet verkeerd is. Ik denk dat jij dan ook meemaakt wat jouw medewerker meemaakt. En in welke percentages dat nou zou moeten, dat weet ik niet. Maar ik zeker dat het...

**Interviewer:**	Het zou zonde zijn om de teamleiders af te zonderen van de dagelijkse werkzaamheden?

**Respondent:**	Ik denk het wel. Voor mij is het heel prettig om mee te maken wat hun meemaken en ik denk ook dat het voor hun iets naar de medewerker toe is. Dat het, ondanks dat je hiërarchisch boven hun staat, je niet vies bent om ook hun werk te doen.

**Interviewer:**	Nee, precies. Merk je ook dat je meer begrip krijgt van mensen?

**Respondent:**	Ja, dat denk ik wel. Of dat nou heel erg toetsbaar is, dat weet ik niet. Maar ik heb wel het idee dat ze het fijn vinden dat wat zij doen, dus ook de minder leuke dingen, dat ik die ook gewoon doe. Ik ben er zeker van dat het heel erg van nu is. Ik zou het zelf ook niet anders willen. Ik hoop dat je er wat mee kan.

**Interviewer:**	Heel erg bedankt.

*Einde transcript*

# Transcript Interview FLM 10

**Duur interview:	66 minuten en 7 seconden**

**Functie:	Teamleider**

**Afdeling(en) onder supervisie:	>AFDELING<**

**Gender:	vrouw**

**Werkervaring in functie:	1 jaar**

**Interviewer:**	Eigenlijk mijn eerste vraag is 'wat doe jij zowaar als teamleider?'. Gewoon heel breed.

**Respondent:**	Ik ben nog maar een jaar teamleider. Voorheen was ik senior op de afdeling en dat deed ik vanaf 2009 en had ik al een heel breed takenpakket werd daar wel in woord en in glimlach naar beloond, nooit financieel. Op een gegeven moment is dat wel financieel beloond. Maar dat was iets van 60 euro in de maand voor heel veel verantwoordelijkheden. Toen ben ik gevraagd door een andere afdeling om daar te solliciteren als teamleider. Ik heb open kaart gespeeld en heb ik gezegd van 'ik heb volgende week uitgenodigd voor een gesprek'. En zodoende ben ik, wilde ze mij hier niet kwijt en ben ik hier teamleider geworden. We zijn sinds vorig jaar februari aan het groeien in deze rol. Dus honderd procent wat er van mij wordt verwacht, denk ik globaal te weten, maar dat is nog steeds in ontwikkeling. Dus in die zin is het niet helemaal puntsgewijs vastgelegd wat er van mij verwacht wordt.

**Interviewer:**	Hoe is het voor jou duidelijk aan het worden wat er van jou verwacht wordt?

**Respondent:**	Omdat ik van mijn bedrijfsleider bepaalde taken toegeschreven krijg en we wekelijks overleg hebben waar we dingen bespreken. Dus ik doe voornamelijk alles dat wat ik al deed en heb daar bepaalde taakjes bij gekregen eigenlijk. En dat samen, dat zie ik dan, als mijn werkzaamheden als teamleider.

**Interviewer:**	Zie je een groot verschil tussen jouw functie nu als teamleider en toen rol als senior?

**Respondent:**	Ja en nee. Ik krijg meer gelegenheid, meer tijd, om deze rol te vervullen dus ik krijg ook meer gelegenheid om meer dingen te doen. Het verschil zit hem met name in het organisatorische vlak. Dus dingen borgen zoals kwaliteit, veiligheid om daarmee in ontwikkeling te gaan. De jaargesprekken met de medewerkers. Het is. Jaargesprekken was echt heel anders. Kwaliteit en veiligheid hield ik mij ook mee bezig, maar daar wordt ik nu toch meer mee geconfronteerd. Het moet gewoon rond zijn en op gebouwd worden en het is niet ter ondersteuning van je teamleider van 'geef maar een zetje, dan kan zij hem inkoppen'. Er wordt van mij wel verwacht dat ik dat zou moet doen.

**Interviewer:**	Jij bent nu echt verantwoordelijk?

**Respondent:**	>BEDRIJFSLEIDER< blijft eindverantwoordelijk, maar ook ik vind dat ik wel wat mee moet brengen in deze functie. En voor de rest eigenlijk alles wat ik al deed in die zin van, ik voorzie de agenda's van de spreekuren, dus het spreekuurroostering, personele roostering doe ik, ik houd alle vakantiedagen bij, vrije dagen van personeel en van artsen en maakt daar een rooster op, ik zorg voor de aanschaf van nieuwe materialen en alle stromingen die vanuit inkoop daarbij komen kijken, risico-inventarisaties en dat soort dingen mee. Soms heel vervelend, maar dat hoort er dus ook bij. Ik spreek mensen aan op afspraken die niet gemaakt zijn, zodat we wel een beetje dezelfde richting op kijken. Daar zie ik dan op toe zeg maar. Dat we ons ook aan de afspraken houden. En voor de rest zie ik mijzelf als een soort communicator tussen de artsen en het andere personeel, in die zin dat ik iedereen op de hoogte houd van ontwikkelingen of dingen die er spelen.

**Interviewer:**	Hoe doe je dat, dat communiceren?

**Respondent:**	Dat, een keer in de week heb ik een half uur in de ochtend vroeg een met een dermatoloog, waar we dingetjes uitwissen van 'hoe staat het hiermee, wat vind je daar van?'. Soms gaat het ook tussen neus en lippen door van 'dan komen we er vrijdag op terug' en diezelfde vrijdag heb ik met >BEDRIJFSLEIDER< een overleg, ook wekelijks, daarin bespreken we dan ook de zaken die we vanuit die hoek. Dus ik, ik bemiddel een beetje daartussen. Wat van boven, schuif ik door naar beneden en alles van beneden schuif ik door naar boven. Dus ik ben een beetje, die taak of die functie zie ik ook wel daarin. Ik vind het heel belangrijk want dat is hoe je samen een bent, zonder lange lijnen die niet werken, overleggen waar iedereen bij aanwezig moet zijn en zo. We hebben ook elke dinsdagochtend, aan het eind van de ochtend, een overleg met de assistentes onderling waarbij we ook dingen vragen naar artsen toe van 'hoe zit het nu daar mee?'. Nou ja, dat neem ik dan ook mee. Iedereen krijgt van mij een verslag per mail toegestuurd. Dat zijn alle artsen, alle assistentes, maar ook onze flexibele medewerkers zodat iedereen op de hoogte is van de afspraken die zijn gemaakt. En soms zijn dat bijstellingen om processen sneller te laten lopen. Of nieuwe afspraken van ja factuuroverzichten, eigenlijk alles wat ter sprake komt.

**Interviewer:**	Duidelijk. Werk je ook nog mee?

**Respondent:**	Ik werk twee dagdelen minimaal mee. Afhankelijk van daar waar het roostertechnisch nodig is, gooi ik dat ook nog wel eens om, dan kom ik op een dag dat ik eigenlijk vrij ben. Dan compenseer ik dat met een ander moment of als ik niet nodig ben op de werkvloer, dan wordt ik door een collega, die de indeling, dus de taken verdeelt, die zegt van 'doe jij gewoon maar teamleidertaken', dan krijg ik al die tijd weer terug. Daarentegen als er iemand zeeziek is of er is een calamiteit, dan spring ik er ook meteen weer in om mee te kunnen werken, daar waar nodig is, dus het primaire proces blijft in die zin gegarandeerd doorgaan.

**Interviewer:**	Vind je dat een goed iets? Je bent eigenlijk heel flexibel inzetbaar?

**Respondent:**	Ten eerste kan ik dat gezien mijn privésituatie. Ik heb geen jonge kinderen meer, ik deel dat zelf in. Die vrijheid heb ik, dus dat kan. En ja, ik probeer mij daar in te zetten waar het het hardste nodig is en ik compenseer dat wel weer met een ander moment dus dat. Die vrijheid heb ik en neem ik ook zeker.

**Interviewer:**	Ik hoorde net al een klein stukje HR tussen de regels. Heb jij zelf ook het idee dat jij een personeelsmanager bent?

**Respondent:**	HR. Het blijft sowieso belangrijk en nodig in alles wat ik doe. Ik merk wel bijvoorbeeld als er artsen gasten uitnodigen, en dat soort dingen meer, dat ik moet regelen dat die Cv’s er zijn dat er ook referenties moeten worden opgevraagd. Dat soort dingen meer. Om het dan door te koppelen naar HR zelf toe. Ik probeer natuurlijk collega's te adviseren in de mogelijkheden die er zijn binnen HR. Dan moet je denken aan fietsplannen of een PLB plan. Dat soort dingen meer probeer ik dan een beetje bij te houden, voor zover ik helemaal op de hoogte ben. Voor mij blijven er telkens maar nieuwe dingen komen. Maar die haal ik gewoon bij mijn bedrijfsleider zo van 'ik zit hiermee, wat zal ik doen? Is dat calamiteitenverlof, of wat?'. Dan stuurt >BEDRIJFSLEIDER< mij daar in en dat is dat leermoment waar ik mee bezig ben.

**Interviewer:**	En dan wordt jou functie ook steeds duidelijk, wat de kader...

**Respondent:**	Dat blijft nog een beetje. Toevallig hebben we laatst overleg gehad met het hele team en daarin werd toch wel gezegd 'wanneer moeten we nou naar jou en wanneer moeten we nou naar >BEDRIJFSLEIDER<, moet welke beweging worden gemaakt he?'. Dat blijkt dus nog niet voor iedereen duidelijk te zijn. Voor mijzelf heb ik dat wel redelijk, dat ik denk van 'alles met PR en financiën, daar gaat >BEDRIJFSLEIDER< eigenlijk over' en verder, probeer het maar eerst bij mij, dan kan ik het altijd nog doorspelen naar >BEDRIJFSLEIDER<. Daar is ook niets mis mee. Als je het eerst bij mij neerlegt. Als ik er niets mee kan, stuur ik het vanzelf wel door. Dus ik maak daar ook niet zo'n probleem van. Maar echt heel helder gekaderd. Ik ben hier natuurlijk aangekomen zonder dat er heel duidelijk een profiel lag van 'die doet dit, die doet dat, he?'. Wie waarvoor verantwoordelijk is. Dus echt heel op de letter is dat nooit zo neergelegd.

**Interviewer:**	En nu gaandeweg...

**Respondent:**	Ben ik het een beetje aan het leren ja. En ik merk ook dat mijn bedrijfsleider het prettig vindt dat het hier zo zelfstandig mogelijk loopt allemaal. Want >BEDRIJFSLEIDER< vertrouwt erop dat het hier allemaal goed gaat. Nou ja, dat vertrouwen probeer ik >BEDRIJFSLEIDER< te geven. Zodat >BEDRIJFSLEIDER< tijd en gelegenheid heeft voor andere dingen en voor andere mensen.

**Interviewer:**	Is dat voor jou niet stressvol?

**Respondent:**	Soms. Ja. Soms.

**Interviewer:**	Heb je dan niet het idee waar je aan toe bent?

**Respondent:**	Nee. Dat niet. Het is voor mij in die zin dat er voor mij er heel veel zaken geregeld moeten worden. Dat heeft niet zo zeer over wat er geregeld moet worden, de hoeveelheid dat er geregeld moet worden. Dan merk ik vaak van 'jeej, waar haal ik de tijd vandaan?'. Dan plan je gewoon overdag iets in van 'ik ga vandaag dit doen', maar puntje bij paaltje aan het eind van de dag denk je 'ik ben er weer niet aan toe gekomen'. Dat is het enige waarvan ik denk van, stressvol is het niet heel erg laat ik het zo zeggen. Maar soms frustreert het een beetje dat je niet genoeg tijd neemt, krijgt, voor de dingen die je eigenlijk wil doen. Er zijn altijd net weer andere prioriteiten die om het hoekje komen kijken. Kan ook deels bij mezelf liggen hoor, dat ik niet zo goed het time management beheers. Bij wijze van spreken zeg ik. Dat kan ook. Maar dat zit dan ook een beetje in dat leerproces, om dat in te delen. Zo zie ik het nu zelf op dit moment.

**Interviewer:**	Dus, is het voor jou wel duidelijk, als we dan een jaar zouden plannen, wat jij voor dat jaar zou moeten doen?

**Respondent:**	Nou, zo'n langetermijnplanning heb ik niet zelf. Juist uit dat wekelijkse overleg met mijn bedrijfsleider, daar komen vaak dingen naar voren van 'dit is belangrijk dit moeten we deze maand wel doen', dan probeer ik dat die maand wel voor elkaar te krijgen. Maar echt een langetermijnplanning van 'voor het einde van het jaar moeten we dit rond hebben', dan heb ik **Interviewer:**	eerst heel veel andere dingen die ik eerst af wil maken en tegen die tijd kom ik daar wel aan.

**Interviewer:**	Je hebt wel een paar aantal vaste verplichtingen als teamleider denk ik, zoals bijvoorbeeld de jaargesprekken...

**Respondent:**	Die staan gepland. Een jaar van te voren staan ze ingepland.

**Interviewer:**	Zijn er meer van dat soort dingen die nu al vast staan? Waar je als het ware een beetje naartoe kan leven?

**Respondent:**	Ik weet alleen dat we juni volgend jaar de NIAS audits hier in het ziekenhuis. Dus ik weet dat voor die tijd de lijsten ter verbetering nog allemaal moeten worden afgewerkt. Dat weet ik wel. Daar ben ik dan ook, daar hebben we het regelmatig over van 'hoe staat het hier mee?'. Ik probeer wel heel adequaat elke keer van 'oh, dat zit niet goed. Als we controle krijgen dan'. Dus dan probeer ik dat wel gelijk aan te pakken want dan is een vinkje weg zeg maar van wat gedaan moet worden. Maar ik heb niet in die zin van te voren gepland van 'ik ga dan dit doen en ga dan daar tijd aan doen'. Ik heb een lijst 'oké, dit moet veranderen'. Oké, wat heb ik daar voor nodig? Dan onderneem ik actie, niet gepland. Wel redelijk duidelijk wat er verwacht wordt.

**Interviewer:**	Zijn er misschien nog meer dingen die HR-gerelateerd zijn en die bij jou zouden liggen?

**Respondent:**	Ik zit even te denken hoor.

**Interviewer:**	Ik zou je een stukje op weg kunnen helpen?

**Respondent:**	Met voorbeelden he?

**Interviewer:**	Dit zijn voorbeelden...

**Respondent:**	Nou bij de werving, werving en selectie, maak ik meestal samen met mijn bedrijfsleider een plan op van 'wat zoeken we, wie zoeken we, waar moet het aan voldoen, wat willen we wel en wat willen we beslist niet?'. De tekst wordt samen even opgesteld, voor zover het niet meer dan een standaard tekst is voor vacatures die geplaatst gaan worden. Wij doen samen ook de selectiegesprekken, maar ook wat breder in het team. Er zitten ook altijd artsen en assistentes bij. Meestal doe ik met een dermatoloog en een bedrijfsleider de eerste ronde en daarna volgt nog een ronde met medewerkers en andere collega's zodat die daar ook iets over kunnen zeggen. Introductie. Ik houd met elke twee weken bezig met de introductie van coassistenten en begeleiding daarvan. Dan vertel ik wat de afdeling inhoud, wat hun verwachtingen zijn vraag ik meestal, ik geef ze een rondleiding en geef ze precies aan 'bij die moet je zijn of zo werk het schema en zo, dat valt dan even onder introductie. Natuurlijk nieuwe medewerkers, nieuwe collega's, dar geldt dat ook voor. Natuurlijk nieuwe collega's, die moeten wat breder. Die krijgen ook een introductie via het ziekenhuis zelf een een SAP-training en dat soort dingen meer. Dus ik zorg wel dat ze aan de slag kunnen. Bijhouden personeelsbestanden. In die zin dat ik ook DRP-registratie doe. Dus vakantieurenregistratie. Dat doe ik voor de assistenten, de arts-assistenten zeg maar, voor mijn bedrijfsleider. Een andere collega doet het juist weer voor alle doktersassistentes. Dus we zitten niet in ons eigen bestand te kruizen of zo. De zomervakantieaanvragen verwerk ik dan gelijk mee daarin. Maar mijn collega checkt die dat allemaal nog of dat goed is gedaan. Die dat dat per maand zeg maar. Beslissen over en plannen van trainings en ontwikkelingsbehoeften medewerkers. Ik houd per jaar een lijstje bij van symposia die er zijn en bijscholingen die er zijn, op wondgebied meestal, in dat lijstje houd ik ook bij wie wanneer waar naartoe gaat. Als dat er weer aan zit te komen kijk ik even hoeveel mensen daar naartoe zouden kunnen en aan de hand van dat lijstje zeg ik van 'nou, ga jullie inschrijven. Jullie mogen daar nu naartoe'.

**Interviewer:**	Mag jij dan ook uiteindelijk de beslissing nemen om dan dat groepje daarheen te sturen?

**Respondent:**	Ja. Dat ben ik zelf. Meestal weet ik van 'nou daar gaan twee mensen naartoe' en bij sommige bijeenkomsten die op de >AFDELING< met name toegespitst zijn, daar kunnen er meestal vier naartoe. Dan is het altijd zo plannen van 'het liefst twee mensen die niet te vrij zijn, dat in ieder geval het rooster hier wel door kan draaien'. Want dat blijft altijd wel de boventoon voeren. Het primaire proces moet doorgaan. Dat probeer ik dan in dermate te sturen. Het geven van formele training. Ja het geven van formele training, dat weet ik niet helemaal waar je op doelt, maar ik help mee begeleiden stagiaires waar nodig is. Dat doe ik incidenteel. Ik ben eigenlijk vraagbaak voor collega's.

**Interviewer:**	Dat is meer de informele training die er onder staat. Het is niet zo dat jij voor de groep gaat staan, als er een nieuw protocol is of als er iets nieuws geïnstrueerd moet worden.

**Respondent:**	Ja. Dat soms wel. Dat is afhankelijk ervan. Ik heb meestal. Meestal nodigen we iemand uit die met dat onderwerp te maken heeft. Maar ik heb ook zelf verbeteringen doorgevoerd door een lean-opleiding die ik heb gedaan. En daar hebben we ook veranderingen doorgevoerd op de afdeling en ja, dan laat ik hen ook zien, middels een presentatie, van 'dit betekent het voor jullie, ga nu op deze manier werken, wat vinden jullie ervan?'. Dus in die zin geef ik ook nog wel eens uitleg over vernieuwingen binnen ons SAP. Ons ziekenhuisinformatiesysteem, als daar veranderingen in zijn. Ik ben zeg maar kerngebruiker, dat inhoud dat je het eerste aanspreekpunt bent voor de automatiseringsdienst en bij storingen en bij nieuwe instructies die met dat systeem te maken hebben. Dat koppel ik in die zin altijd wel weer door. Of ik probeer een mooie werkbeschrijving te maken met plaatjes erbij, heel visueel te maken, als een soort handleiding gebruikt te worden.

**Interviewer:**	Vind je dat dat iets is dat bij een teamleider ligt?

**Respondent:**	Niet specifiek. Maar ik vind het leuk om te doen. Het is iets dat ik altijd heb gedaan. Ik vind het wel belangrijk dat er een goede werkinstructie komt. Het is leuk om te doen en ik ben er ook wel goed in. In die zin, ik ben er niet zo lang mee bezig. Het komt ook gewoon omdat je de computerprogramma's die je daarvoor gebruikt beheers. Er gaat mij niet heel veel tijd in zitten. En dan weet je het he. Dan weet je dat het goed gaat. Mentoren. We doen nog wel eens, een collega die langdurig ziek is geweest, die heb ik in gesprekken begeleid. Aangehoord waar haar problemen waren. Af en toe denk ik ook dat ik een soort maatschappelijk werker ben hier. Dus in die zin probeer ik ze persoonlijk te sturen in 'zou je het niet eens zo aanpakken, of denk er ook eens aan ga ook eens in gesprek, geef eens aan waar jouw grenzen liggen en wat jouw wensen zijn, de ander ziet dat niet'. Dus in die zin probeer ik mensen graag handvatten te geven waar ze mee verder kunnen, ook in hun privé.

**Interviewer:**	Vind je dat oké? Is dat iets dat bij jou ligt?

**Respondent:**	Ja. Ik vind dat prettig. Als ik van mensen terug hoor dat zij dat heel prettig vinden en ik vind dat wel, ja, het tevreden zijn en het werkplezier zie ik wel als onderdeel van mijn functie. Dat zie ik persoonlijk wel zo. Dat ik daar mede verantwoordelijk voor ben hoe zo iemand functioneert op de poli. En ik vind het heel prettig als je daar gewoon met een klein beetje levenservaring of ideeën of tips iemand mee op weg kan helpen, waarmee die gewoon weer lekker op zijn werk verschijnt. Ik weet ook dat ik naar maatschappelijk werk kan sturen, maar dit is veel intiemer en werkt net zo goed en ik weet meteen hoe het met mijn medewerker is. Ik vind dat belangrijk dat ik dat ook mee kan nemen in het functioneren en in het ontwikkelen van de persoon zelf.

**Interviewer:**	Hoe ben je dan op de hoogte van dit soort dingen? Iemand is langdurig ziek, dan krijg je een ziekmelding binnen. Maar zijn er ook nog andere manieren waarmee je op de hoogte bent?

**Respondent:**	Wij hebben allemaal een hele laagdrempelige band met elkaar. Er wordt heel vaak gewoon in de ochtend bij het binnen komen 'hoe was gister je dag?'. Dan komen daar dingen ter tafel vaak natuurlijk. We zijn erg hecht ook gewoon met het team. Dus het is niet zo 'we werken gewoon samen, that's it'. Nee we zijn ook heel hecht en dat we van elkaars privésituaties op de hoogte zijn allemaal. Daarom vind ik het gewoon belangrijk die band ook goed te houden. En ik vind, ja, als ze mensen vragen van 'nou, dit gaat echt niet lekker, weet je, ik zit ermee'. Soms, vaak zie je het ook aan de mensen zelf van 'goh, zullen we even gaan zitten of zo, want ik heb het idee dat we even met elkaar moeten gaan kletsen of zo?'. Nou heel vaak komt daar dus iets uit en als dat opluchting geeft bij diegene dan denk ik 'nou, dan kan die weer wat gefunctioneerd, geconcentreerd of gemotiveerd weer aan het werk'. In die zin vind het wel iets bij mij hoort en bij mij past, wat ook bij de functie. Ja. Het welbevinden van je team. Ik ben niet overal verantwoordelijk voor, maar ik zie het wel als toegevoegde waarde als ik dat meeneem. Zo zie ik het.

**Interviewer:**	De jaargesprekken?

**Respondent:**	Ja. De jaargesprekken doe ik jaar voor het eerst.

**Interviewer:**	Voer je met iedereen het jaargesprek?

**Respondent:**	Ik doe het zo veel mogelijk met mijn bedrijfsleider nog op dit moment. De ene keer zegt >BEDRIJFSLEIDER< van 'doe jij de gesprekken zelf maar'. >BEDRIJFSLEIDER< wil ook een deel van de jaargesprekken zelf blijven doen om dat ook in >BEDRIJFSLEIDER< routine te houden zeg maar. >BEDRIJFSLEIDER< wil dus niet alles overgeven en zegt 'ik wil er ook graag een paar zelf doen'. En dan ben ik wel degene die bij het gesprek aanwezig is en, gewoon ja, ik probeer naar >BEDRIJFSLEIDER< te luisteren en kijken hoe >BEDRIJFSLEIDER< het aanpakt. En ja. Met name bij lastige onderwerpen leer ik daarvan en bij andere kan ik laten dat ik het ook best aardig onder de knie heb. Dus we doen dat een beetje om en om en samen en in overleg met elkaar. We overleggen even samen met elkaar van te voren of >BEDRIJFSLEIDER< het gesprek doet of ik en wat er bij de artsen vandaan is gekomen aan feedback. En ja. En zo maakt of >BEDRIJFSLEIDER< het verslag of ik maak het verslag. Dus zo.

**Interviewer:**	Delen jullie de groep bij wijze van in tweeën of is er wel een reden voor dat >BEDRIJFSLEIDER< juist die mensen...

**Respondent:**	Vandaag hadden we toevallig een gesprek en van 'nou ik denk dat jij het maar gaan doen'. Ik zei 'nou, ik loop even naar het kopieerapparaat dan zal ik dat even overdenken', ik was er namelijk in gegaan dat >BEDRIJFSLEIDER< dat zou gaan doen. Ik zeg 'ik vind dit toch een lastige'. Ik denk dat ik het toch fijner vind als jij dat doet, dan luister ik mee. En dan ja. Dan geef ik het aan en zegt >BEDRIJFSLEIDER< van 'oh ja, geen probleem'.

**Interviewer:**	Je ben ook gewoon vrij om te zeggen...

**Respondent:**	Ik zit in een leerfase dus ik, weet je, ik kan beter naar >BEDRIJFSLEIDER< luisteren waar ik dan van leer, dan kan ik dat volgende keer, weet je. Dat heeft ook een beetje te maken met hoe ik, en ja, we zijn makkelijke collega's in die zin dat ze snel gepleased zijn, dat ik ook weet dat ze zich goed ontwikkelen. Ik vraag ook niet zo veel. Ze zijn tevreden met wat ze hebben. Functioneren gaat ook heel goed. Dan gaat het dus meer op de momenten dat je andere mensen toch moet aanspreken van 'ik vind dit toch wel een minpuntje van je, wat vind je er zelf van?'. Ik merk dat ik dat een beetje lastig vind, dat ben ik nu aan het leren.

**Interviewer:**	Want, ik denk dat hij iets verder op het lijstje staat, het disciplineren en corrigeren van medewerkers...

**Respondent:**	Dat ligt ook bij mij.

**Interviewer:**	Hoe pak je dat op?

**Respondent:**	Dat is een kwestie van op de juiste manier feedback geven. Gewoon ook direct vragen, niet doen alsof je neus bloedt. We hebben daar afspraken over gemaakt, ik zie dat jij dat toch doet, vertel eens, hoe komt dat? We hebben samen die afspraak gemaakt en blijk je er toch niet aan te houden. Dus meer op die fiets. We maken ook vaak samen bepaalde afspraken. Dus dan kan je daar makkelijker op terug komen. Ja, we hebben dat samen afgesproken. Wat is nou de reden dat jij je daar niet aan houdt?

**Interviewer:**	Dat klinkt, komt over alsof het nog enigszins coachend is, is er ook wel eens een moment geweest waarop je heel autoritair hebt moeten zijn en er boven hebt moeten staan van 'goh, wat jij nu doet, dat vind ik echt niet oké.

**Respondent:**	Ja. Ja. Niet heel veel. Ik denk dat ook te maken heeft met het team zelf. Hoe ver wel al zijn gekomen. Maar, ik moet ook wel een impopulaire beslissingen maken. Dat komt wel eens voor. Gelukkig komt dat niet heel vaak voor. Of dat ik een bepaalde weg kies waarvan collega's zoiets hebben van 'nou, ik weet niet of dat handig zou zijn'. Nee, we gaan het nu zo doen. Mocht dat niet werken, kunnen we altijd nog. In die zin. Ja. Ik overdenk het wel altijd welke kant ik op wil en de mening van het team te vragen van 'goh, wat vinden jullie?'. Dus in die zin komt het niet zo vaak voor. Ik heb wel eens zaken, ook naar de artsen toe, van 'nou, dat kan gewoon echt niet. Dan krijgt diegene dat te horen van mij. De ene keer wat subtieler dan de andere keer. Maar ik laat het wel weten. Meestal is het zo van 'jongens, we hebben dit afgesproken en nou zie ik dat het alweer niet is gedaan. Nu wil ik gewoon dat je deze week eens een schema maakt en daar ophangt. Dan gaan we het zo doen'. Ja. Het team heeft het niet heel erg nodig. Misschien dat het andere teams wat meer van toepassing is, maar ik hoef eigenlijk niet zo vaak op mijn strepen te gaan. Dat valt wel mee hier. Dat is wel fijn. Ook een fijn team.

**Interviewer:**	Zijn er misschien nog andere punten die je misschien opvallen?

**Respondent:**	Klachtenprocedures doe ik ook. Ik zorg dat het in ieder geval teruggekoppeld wordt en geef wel altijd respons namens de afdeling. Het liefst handel ik het hier te plekke af. Dan neem ik de patiënt apart en bespreken we het meteen want het heeft veel meer effect om het meteen ter plekke op te lossen dan te wachten op een klachtenbrief. Dat proberen we hier wel. Absentiemanagement. Dat is ook iets waar ik dit jaar pas mee begonnen ben. Of eigenlijk vorig jaar. Ben ik ook in gecoacht door mijn teamleider zo van 'dit zou je ook kunnen aanbieden, dat zou je kunnen aanbieden'. Tot nu toe. Het enige is dat het gewoon heel veel administratie vergt. Dat is het enige dat daar vervelend aan is.

**Interviewer:**	Het is wel duidelijk wat er van je gevraagd wordt?

**Respondent:**	Ja. Vaak wel. Niet altijd. Want dan hebben we nu zo dat. Uh. Verzuimregistratie of zo in een programma en dan krijg je een melding dat er weer wat moet. En ja, elke week bel ik mensen of maken we afspraken. Dat vind ik nog wel eens lastig. Je krijgt elke keer weer een melding 'je moet er weer wat mee, je moet er weer wat mee'. Terwijl ik zelf het idee heb 'ik bel die mensen wel, ik heb daar dingen mee afgesproken'. Maar je moet alles dus gaan vastleggen.

**Interviewer:**	Dat klink heel geforceerd?

**Respondent:**	Ja. Nou ja, bijna Youforce, geforceerd zeg maar, maar dat is weer een ander programma.

**Interviewer:**	Maar het is wel fijn dat je die meldingen krijgt...

**Respondent:**	Dan weet je 'dan moet je moet je er weer wat mee'. Met name met langdurig zieken.

**Interviewer:**	Dus ondanks dat je er iets mee moet, is het wel fijn dat je weet waar je aan toe bent?

**Respondent:**	Ja. Nou. Er zijn administratieve zaken nodig, die je later, Wet Poortwachter, wetgeving. Echt heel langdurige zieken. Dat is niet een heel groot percentage. Ik heb nu toevallig een iemand die door ziekte langdurig is uitgeschakeld. Die anderen heb ik dus met persoonlijke gesprekken kunnen helpen en die functioneren nu weer helemaal. Dat is soms nog wel eens lastig, met name dat.

**Interviewer:**	Ik bedoelde meer met het idee van 'als je iets doet dat nieuw voor je is, dan kan zo'n systeem, ondanks dat je dan enigszins geforceerd wordt om dingen te doen, zo'n systeem kan jou dan helpen aan te geven wanneer er dingen van jou verwacht worden'?

**Respondent:**	Ik heb persoonlijk het idee dat iedere week iets moet gaan. Terwijl, als iemand over drie weken naar het ziekenhuis gaat om resultaten te bespreken, dan denk ik 'ja, wat moet ik hier nou tussen gaan zitten?'. Dat over drie weken weet ik pas meer. In die zin denk ik 'pff, weer die melding'. Dat in die zin denk ik meer, dat ik denk van 'moet ik nou elke week opschrijven dat ze over drie weken naar het ziekenhuis kan?'. Dan is er in de tussentijd niets verandert bij wijze van spreken. Dat vind ik gewoon, dat ik denk van.

**Interviewer:**	We zijn wel lekker legaal bezig?

**Respondent:**	Ja. Weer. Waardering geven. Ja.

**Interviewer:**	Hoe doe je dat?

**Respondent:**	Complimenten. Complimenteren. Of zeggen dat ik het fijn vind dat iedereen toch weer bij zo'n vergadering aanwezig is. Dat mensen de moeite hebben genomen, om input te geven, of in die zin. Ik probeer het wel. Men doet het te weinig, weet ik. Zeker als ik wat gemopper heb gehoord in de groep weet ik dat dat heel erg motiverend kan werken, een compliment.

**Interviewer:**	En gaat het soms ook wel eens verder dan een compliment?

**Respondent:**	In de zin van een waardering, iets financieels of iets in die geest?

**Interviewer:**	Bijvoorbeeld.

**Respondent:**	We hebben toevallig een collega gehad in het jaargesprek die heeft aangegeven meer salaris te willen verdienen,  dit is een zijinstromer die het werk enorm goed doet en daarvan hebben we gewoon samen met de bedrijfsleider over gesproken en daar is toch uit gekomen dat diegene een stap hoger zeker verdient. Dus daar hebben we ons hard voor gemaakt om dat voor diegene voor elkaar te krijgen.

**Interviewer:**	Dat is iets over salarisschalen, dat doe je samen met de bedrijfsleider?

**Respondent:**	Ja. Dat beslis ik niet samen. Of, dat beslissen we samen. Dat doe ik niet zelf. Daarom haal ik ook bij >BEDRIJFSLEIDER< de informatie van 'wat is er mogelijk?'. Omdat >BEDRIJFSLEIDER< die ervaring heeft en ik niet. Voor mij was het de eerste keer en ik vind niet dat het aan mij is om die beslissing te maken. Dat is iets wat je gezamenlijk moet doen.

**Interviewer:**	Zou je veel verder van huis zijn als je een bedrijfsleider had die jou niet makkelijk van informatie zou voorzien?

**Respondent:**	In die zin van dat >BEDRIJFSLEIDER< dingen sneller aan mij overlaat?

**Interviewer:**	Nee, zoals met die salarisschalen 'wat kan ik hier nu mee', dan ga je naar de bedrijfsleider om te vragen 'wat kunnen we hiermee', dan heb je een bedrijfsleider die meewerkend is en jou wil helpen omdat jij lerend bent, dan komt er zoiets in me op van 'dan is die relatie tussen teamleider en bedrijfsleider heel belangrijk om goed je werk te kunnen doen'?

**Respondent:**	Voor mij wel. Ja.

**Interviewer:**	Dus als die relatie minder goed zou zijn...

**Respondent:**	Ik denk dat dat wel een voorwaarde is dat je wel goed met elkaar om kan gaan. Aan de andere kant kan ik ook de informatie halen bij een HR of dat soort dingen meer. Mijn bedrijfsleider is iemand die echt meedenkt, die de personen kennen, die de omstandigheden kennen. >BEDRIJFSLEIDER< heeft vorig jaar alle jaargesprekken gehad, dus >BEDRIJFSLEIDER<. Ja. Ik vind het prettig dat ik heel goed met >BEDRIJFSLEIDER< kan samenwerken. Dat vind ik wel belangrijk. En zeker. Je krijg de kans om in zo'n functie te groeien. Voorheen was hier namelijk een heel traject voor om teamleidertrainingen te doen. Dus ik heb >BEDRIJFSLEIDER< wel nodig daarvoor. Er is geen andere optie.

**Interviewer:**	Zou je wel voorstander zijn voor zo'n traject?

**Respondent:**	Ja. Ik wel ja.

**Interviewer:**	Wat zou het voordeel zijn?

**Respondent:**	Nou, kijk sowieso dat je, kijk als ik iets nu, ga ik wel eens bij een andere teamleider wat kletsen of zo he, onderling. Ja, dan weet je wat er speelt. We hebben wel eens teamleideroverleg. Dat is een keer in de twee of drie maanden of zo. Dan worden bepaalde onderwerpen aangesneden en dan merk je toch wel dat niet iedereen op dezelfde golflengte zit. De een pakt het zus aan, de ander pakt het zo aan. En ik denk dat die handvatten die ik nu steeds moet gaan halen, dat als je die in een cursus krijgt, dat je veel meer ruimte voor 'waarom dan, en wanneer zou je dat dan?'. Je krijgt dan in een keer zo'n pakket aan informatie waar je wat mee kan. En nu moet ik er eerst tegen aan lopen en dan ga ik het ter sprake brengen. Maar ja. Die training is er niet meer. Dus ik doe het op mijn eigen manier.

**Interviewer:**	Als je met die andere bedrijfsleider, uh teamleider, spreekt, merk je dan dat er een verschil zit tussen jullie?

**Respondent:**	Qua ervarenheid om maar te beginnen. Ik kom in die zin maar net kijken, in deze functie dan, dat is niet altijd zo 'hoe doe jij dit nou, hoe doe jij dat nou?'. Een heel enkel keertje komt dat tegen en dan merk je toch ook dat je voor verschillende poliklinieken werkt, met hele andere artsen. Dus de verhouding tussen die arts is al heel anders. Kijk, hier tutoieren wij elkaar. Ik ken ze persoonlijk zo ondertussen. Als ik op die andere poli was aangenomen moest ik van beneden af aan beginnen. En dan denk ik 'ja, ik had ook wel met die bedrijfsleider kunnen sparren', maar wat ik dan allemaal moest leren, een heel nieuw vak ik bedoel inhoudelijk, artsen, hele nieuwe collega's, dan was het nog moeilijker geweest. Dan was het wel een uitdaging geweest, maar en ja. Als je al iets meer weet van het teamleiderschap is dat alleen maar mooi meegenomen.

**Interviewer:**	Is het ook voor jou duidelijk wat jouw beslissingsbevoegdheid is? Waarover jij wel en waarover jij niet als teamleider over zou mogen beslissen?

**Respondent:**	Het is grotendeels op gevoel. Een financiële bevoegdheid, die grens is heel duidelijk. Er is voor 1.000 euro, alles wat aangeschaft moet worden, kan ik gewoon zelf beslissen en aanschaffen. Daar heb ik tekenbevoegdheid voor. En voor de duurdere zaken moet ik dat toch doorspelen naar mijn bedrijfsleider. Dus dan doen we altijd 'dit is iets dat aangeschaft moet worden' en dan moet je een pre maken voor de inkoop, de ja of de nee. Soms doen we dat samen. Dan moet je het motiveren en dan is het er niet zo één, twee, drie. Het zijn vaak hele lange trajecten. En dan is het goed dat je iemand, mijn bedrijfsleider heeft een hele zakelijke overview en een bedrijfskundige opleiding gehad. En ik juist de medische dus ik weet hoe het op de werkvloer werkt, maar >BEDRIJFSLEIDER< weet alles hoe het daar boven zo is. Dus in die zin denk ik dat het gewoon een hele goede combi is. Het ligt er net aan op welk vlak er iets moet worden beslist, want als ik iets ga vragen over een behandeling, dat heeft >BEDRIJFSLEIDER< daar totaal geen kennis van. Als ik iets bedrijfsmatig moet weten, is >BEDRIJFSLEIDER< degene. Zo hebben we ieder ons eigen kennisvlak ook. Maar we delen dat. Veel beslissingen delen we wel. Maar als het echt over de operationele zaken gaat, vind ik dat zelf mag en doe ik dat ook.

**Interviewer:**	Is het dan ook wel eens voorgekomen dat je een besluit hebt genomen waarvan je dan achteraf hoort 'dat had ik liever zelf gedaan'?

**Respondent:**	Nee. Tot nu toe niet.

**Interviewer:**	Ook niet bang voor?

**Respondent:**	Nee. Als ik het idee heb dat dat niet aan mij is, dan overleg ik dat automatisch eigenlijk.

**Interviewer:**	Dat is een stukje gevoel dat je hebt?

**Respondent:**	Ja. Daar heb ik nooit echt moeite mee gehad. Bij twijfel dan mailen we of dan bellen we even. Ik heb ook nog een dermatoloog natuurlijk, een van de artsen...

**Interviewer:**	Als een sparringspartner?

**Respondent:**	Ja. Vraag ik ook wel eens 'wat vind jij?'. Daar hebben we de overlegjes ook voor, voor dat soort dingen.

**Interviewer:**	En zeker in een nieuwe functie kan ik me voorstellen, er is geen zwart-wit lijn...

**Respondent:**	Nee. Het is gevoel.

**Interviewer:**	Er is grijs gebied?

**Respondent:**	Voorheen was ik al senior van de afdeling. Daardoor wist ik ook al veel dingen ik eigenlijk niet hoefde, die ook niet zwartopwit lagen of uitgeschreven waren, wat ik wel gewoon deed. Je groeit erin. Af en toe pak je links en rechts wat mee. Dus. Beslissen over salarisschalen. In die zin dus niet. Dat ligt allemaal vast als je binnenkomt. Bottom-up communicatie, via mijn bedrijfsleider dan. Ik zit er een beetje tussen he? Het is de ene keer naar collega's en andere keer naar de artsen of naar de bedrijfsleider.

**Interviewer:**	En daar pak je top-down ook mee?

**Respondent:**	Ja. Suggesties van het personeel aanhoren. Dat komt voornamelijk terug in onze dinsdagoverleggen waar iedereen input kan doen middels een schriftje. Die punten worden op die dinsdag besproken. Dat zijn voornamelijk punten om het operationele proces te verbeteren. Van kunnen we dit niet beter gaan mailen', weet je?. Dan is het daar van 'wat vinden jullie er van?', dan gaan we het uitproberen. Dan gaan we het volgende maand eens evalueren of het goed is gegaan en nou goed idee. Dus iedereen kan eigen ideeën inbrengen en alles wat naar verbetering leid wordt eigenlijk al meteen doorgevoerd. Of er wordt beargumenteer waarom iets toch niet zou werken en wat de andere kant zou zijn. In principe hebben we daar het dinsdagoverleg voor ingesteld. Ook omdat de frequentie van een groot werkoverleg eigenlijk te verlagen. Er liggen korte lijntje en korte lijnen naar verbetering, dat het veel efficiënter is. Dat is ook volgens de leangedachte. Dus vandaar dat ik dat heb doorgevoerd ook. Coördineren van het werk. Ik regel hier op de afdeling wel dat, ik geef aan hoeveel personeel er nodig is op een bepaalde dag. Voorheen deed ik ook de taakindeling. Dat heb ik nu afgestoten naar een collega, die normaal de taakverdeling deed. Die vraagt nu ook onze flexibele collega's. Zij zorgt dat er voldoende personeel is. Ik geef alleen aan hoeveel er nodig is en zij vraagt het aan. Zij maakt ook de indeling wie wat gaat doen. Dat heb ik gedelegeerd. Dus dat scheelt weer. Onderhouden van effectief teamwerk, in de zin van 'of ik meedraai'?

**Interviewer:**	Hoe je ervoor zorgt dat iedereen lekker zijn werk kan doen bijvoorbeeld?

**Respondent:**	Ik denk dat dat ook wel uit die dinsdagoverleggen voortvloeit. Dat als je hebt dat dingen anders kunnen, of beter, of mooier, dan wordt aan die behoefte gehoord gegeven.

**Interviewer:**	Als er storing is en je ziet dat, bijvoorbeeld, dan pak je iemand apart om mee te praten?

**Respondent:**	Ja. Ten eerste gebeurt dat niet heel veel. Ik probeer mensen die al heel negatief zijn, op andere gedachten te brengen dat van 'joh, weet je dat helpt geen sier dat negatief denken, het zou fijn zijn als, houd dat voor ogen, het kan ook zo en zo zijn'. Dat is toch even dat setje kunnen geven om fijn te kunnen werken. En daar waar nodig spring ik altijd zelf in. Dus mocht het ergens niet lopen. Personeel consulteren en raadplegen. In die zin dat ik ze wel een apart neem als ik iets wil bespreken met ze. Bij ziekte ga ik zelfs ook nog consulteren in de thuissituatie. Dus dat. Of als iemand opbelt van 'oh', weet je? Paniek in de thuissituatie. En ik ben vrij, dan neem ik de moeite om op de bus te stappen om daar te gaan praten 'kan ik je ergens mee helpen?'. Een beetje afhankelijk van de situatie en of ik denk dat ik iets kan betekenen voor een ander. Ja. Dat is gewoon meer de verbondenheid met elkaar. Ja. Meestal komen ze aan mijn bureau vragen van 'ik kom hier niet verder mee, of dat lukt niet'. Dus in die zin is het ook een soort consulteren raadplegen en de vraagbaak

**Interviewer:**	Is het ook wel eens andersom? Als jij een besluit wil hebben, maken bedoel ik, dat je een advies wil van het team, gooi je het dan ook wel eens in de groep?

**Respondent:**	Ik schrijf ook in het werkoverleg punten op.

**Interviewer:**	Schiet er eens op, wat vinden jullie?

**Respondent:**	Ja. Ik doe het vaak graag in overleg. Of dat ik iemand, we hebben allemaal bepaalde taken. Als ik denk 'jij bent van de wondzorg, zouden we dat niet beter zus en zo kunnen doen?'. Maar dat bespreek ik dan even met diegene. Dat is ja, een beetje het idee van sparren. Zodat we, daarop gebaseerd, een besluit kunnen nemen. Of ik gooi het daarna in het team wat breder van 'we hebben er over nagedacht, het kan zus of zo', wat vinden jullie daarvan? En dan gaat het ook gebeuren in die zin. We hebben allemaal wel zo mensen in het team zitten die kennis hebben van verschillende zaken. Iedereen heeft ook verschillende taken, dus het is ook maar net 'welke taak raakt dat?'. Met diegene ga je dan. Ik ben erg van het sparren en 'wat vind jij?'. Omdat ik weet dat je dan gewoon een grotere draagkracht krijgt om problemen op te lossen. Gezondheid en veiligheid. In hoeverre dat. Natuurlijk, je bent verantwoordelijk voor de veiligheid om de afdeling. We hebben ook de DIM-commissie daarvoor. We pleiten er ook voor dat er altijd ge-dimt gaat worden, melding gemaakt gaat worden van zo'n incident wat gebeurt. Voorheen zat ik zelf in die commissie. Sinds ik teamleider ben geworden, wordt dat niet wenselijk geacht. Ik heb nu andere mensen die staan daar nu in en die beheren dat nu. Per kwartaal behoort er een rapportage te worden gegeven en die krijg ik dan wel in te zien. Ik heb nog wel eens dat als ik het idee heb dat er iets gebeurt is of iemand roept van 'mooi, heb ik weer bijna dat mesje in mijn vingers'. Dan zeg ik wel 'wil je dat even dimmen?'. Dus in die zin probeer ik dat. En de veiligheid ter plekke komt heel vaak uit audits naar voren van 'dit is niet goed, dat is niet goed' of dat kan beter. Dan is het aan mij om dat ook op te pakken en dat is ook qua veiligheid. Dingen waar je zelf nog wel eens aan voorbij holt, wat goed is dat een ander daar eens naar kijkt en dat je daar dan iets mee kan. In die zin voel ik mij ook wel verantwoordelijk voor de veiligheid. Maar ik vind wel dat dat iets is dat je samen moet delen, want als het een iemand is die dat regisseert, dan vind ik wel dat diegene daar een melding van moet maken.

**Interviewer:**	Dingen zoals gezondheid en het verbeteren van werkleven, dan ga je al snel de Arbo-kant op, van 'duurzame inzetbaarheid' en dat soort onderwerpen.

**Respondent:**	Daar hebben we niet zo veel mee te maken. Maar we hebben wel sinds kort twee mensen die een erchocoachtraining hebben gedaan. Die geven binnenkort een presentatie over wat ze hebben geleerd. Die zijn gecoacht door iemand, een extern bureau, en die zijn gecoacht en die hebben daar een presentatie over gemaakt en willen die bij ons, aan ons laten zien. En zij kijken met name naar het arbo-techinische. De houdingen, de zit, het tillen. Dus dat heb ik nu ook aan twee collega's uitbesteed.

**Interviewer:**	Hebben zij dan nog een verwachting van jou dat jij daar iets mee gaat doen als teamleider?

**Respondent:**	Op dit moment nog niet. In die zin, als er iets aangeschaft moet worden, dat ik dan zeg 'dat kan echt niet', daar ben ik dan ook maar mee bezig. Met een stoel zeg maar die nog op mechanisch werkt of in plaats iets wat hoger moet of waar je bij moet bukken. Ja. Dan ben ik betrokken bij de aanschaf van dat nieuwe product. Zij zijn nog maar net in functie. Er is dan ook nog niet veel naar boven gekomen. Die stoel, dat daar een veilige, degelijke stoel moet komen voor de ruggen beter functioneert, ben ik zeker bij betrokken in de zin dat er iets nieuws aangeschaft moet worden. Krijg ik dan weer op mijn bordje. Neem, c krijg, ik vind dat dat wel bij je functie hoort.

**Interviewer:**	Je zei net ook al iets over die 1.000 euro die je zou mogen investeren. Gebruik je die 1.000 euro ook wel eens om te investeren in het personeel?

**Respondent:**	Nee. Dit is echt voor de aanschaf van materialen en zo. Soms hele kleine dingetjes hoor, dan denk ik aan een chocoladeletter met Sinterklaas of ja, die koop ik gewoon en declareer ik. Dan krijgt iedereen een gedicht erbij bij wijze van spreken. We hebben het ook wel eens gehad, van 'ja we hebben allemaal zo hard gewerkt, zou leuk zijn als iedereen zich eens een half uurtje zou kunnen laten masseren'. Nou ja, dan opper ik dat bij mijn bedrijfsleider en die zegt 'ja prima, doen'. En dan wordt het geïnvesteerd. Niet van die 1.000 euro zo zeer. Maar dat idee wordt dan wel uitgevoerd.

**Interviewer:**	Maar ik hoor nu eigenlijk wel twee verschillende dingen. Want een chocoladeletter doe je dan weer zelf...

**Respondent:**	Omdat dat een jaarlijks terugkerend iets is. Dat is ieder jaar weer.

**Interviewer:**	En die massage was..

**Respondent:**	Dat was echt, toen hadden we zo'n frustrerende maanden achter de rust van weinig personeel, optimale inzet van iedereen. Toen dacht ik 'daar moeten we even iets tegenover zetten als waardering voor die lastige periode die we zijn doorgekomen'. Dus ja, dat is alleen maar waar het zich aandient.

**Interviewer:**	Zijn er nog meer vergelijkbare dingen, zoals die chocoladeletter?

**Respondent:**	Nja. We hebben een verjaardagpot, maar daar betaalt ieder zijn eigen bijdrage en voorzie ik dan in de cadeaubonnen. Dat is niet vanuit. Dat is meer vanuit het team zelf. Hooguit complimentjes hier en daar. Niet met financiële zaken. Daar waar behoefte aan is wordt dat geopperd van 'kan hier niet eens iets nieuws voor komen of iets anders of het zou leuk zijn als we ook dit zouden kunnen doen'. Ja. Als het geen enorme bedragen zijn en het lijkt me een goed plan, of het is voor de patiënt toch wel vriendelijker als dat er staat of het ontvangt vriendelijker met een bos bloemen, ik noem maar wat. Dat doe ik dat, maar niet alleen perse voor het personeel. Nee.

**Interviewer:**	Heb jij ook een beeld voor ogen wanneer jij goed functioneert als teamleider?

**Respondent:**	Zolang ik weet van mezelf dat ik mijn best doe, geen kritiek krijg op dingen die ik laat liggen, ga ik er vanuit dat ik mijn best doe. Ja. Ik ga er vanuit dat als ik niet goed mijn best doe, dan krijg ik dat wel te horen.

**Interviewer:**	Dan het jouw collega's die jouw graadmeter zijn van 'doe ik het goed?'.

**Respondent:**	Ja, of de artsen gewoon. Dus ik krijg daar ook feedback van terug. Dat mag naar mijn idee best wat vaker gebeuren. Maar net als anderen, ik geef dan wel complimenten aan collega's van 'goh, ik vind dat je dat goed aangepakt hebt, ik vind het fijn dat je zo veel initiatief neemt'. Ik weet van mijn bedrijfsleider, die geeft mij dan nog wel een positieve feedback, maar ook als het wat minder gaat krijg ik dat ook te horen. Ik zie dat niet meteen als slecht presteren. Ik zie dat meer als leercurve. Dus uh.

**Interviewer:**	Heb jij het idee dat jij nog over bepaalde kennis moet beschikken om je werk goed te kunnen doen?

**Respondent:**	Ik zou nog wel mee willen weten, maar ik zit ook met alle dingen die nu op mijn bordje liggen, niet zo gemaakt van 'ik ga eens een weekje'...

**Interviewer:**	Wat zou je graag willen weten dan?

**Respondent:**	Ja. Dingen zoals met bijzonder verlof, wanneer bijzonder verlof. Dat staat natuurlijk ook allemaal op Idoc. Dus als ik het nodig heb, kan ik het opzoeken he? Vandaar dat ik ook zeg 'die teamleiderstraining zou mij heel zinvol lijken'. Afijn, hij is er niet meer. Zaken zoals. Af en toe krijg ik dan ook een scholing. Dat jij zegt 'er is een bijeenkomst in het auditorium'. De laatste keer ging het over mantelzorgers. Hoe het gaat bijvoorbeeld met al die regelingen. Dat moet je mij niet vragen. Als het op mijn bordje komt, dan ga ik het vragen. Daar heb ik nog niet mee te maken gehad. En ik denk dat dat voor mij op dit moment gewoon het beste werkt zo van 'jongens, als ik het niet weet, dan ga ik het wel opzoeken, ga halen, of ik ga het vragen'. Ik kan nog niet echt zeggen van 'dit'. Aldoende leer ik eigenlijk. Maar dat is, nogmaals, bij het ontbreken van een bepaald traject. Toen de seniors kwamen was er een heel duidelijk seniorontwikkelingstraject zeg maar. Dat je op de communicatie ging of dat je zegt 'wanneer kom je in mijn persoonlijke omgeving?'. Allerlei dingen. Omgang met agressie. Daar teer ik dan nog maar een beetje op. Dat heb ik dan in ieder geval gehad. En met alle andere dingen die aangeboden worden probeer ik gewoon zo veel mogelijk gebruik te maken en daar iets mee te doen.

**Interviewer:**	Duidelijk. Is het voor jou ook duidelijk waar jouw functie ophoud?

**Respondent:**	Bij mijn bedrijfsleider.

**Interviewer:**	En aan wat voor onderdelen zou ik moeten denken waar jij je niet mee bezig ben als teamleider?

**Respondent:**	Ik ben niet echt bezig met bedrijfsmatige zaken. Dus met jaarplannen maken. Ik houd mij ook niet bezig met financiële zaken. Met investeringen houd ik mij niet bezig. Eigenlijk alles wat niet direct het operationele proces raakt zou ik zeggen.

**Interviewer:**	Zodra het de afdeling overstijgt, of zodra het over financiën gaat?

**Respondent:**	Ja. Daar houdt het voor mij wel een beetje op. En ik doe het ook graag samen met de bedrijfsleider. Maar ja, ik heb ook zoiets 'het is ook >BEDRIJFSLEIDER< taak om personeel aan te nemen en dat soort dingen meer en urenberekeningen en zo'. Dat vind ik >BEDRIJFSLEIDER< pakje aan. Dat vind >BEDRIJFSLEIDER< gelukkig ook tot nu toe. Dus in die zin. >BEDRIJFSLEIDER< probeert wel heel veel dingen naar mij toe te sturen van 'daar leer je van'. Voor het geval 'oh het is een nieuwe teamleider of een nieuwe bedrijfsleider krijg', dat ik dan heel veel zelf kan. Dat idee heb ik. >BEDRIJFSLEIDER< ziet dit ook nog niet tien tot twintig jaar voortduren in >BEDRIJFSLEIDER< rol dus heeft zoiets van 'leer het allemaal maar, dan kun je in ieder geval behelpen en je kunt je daarin ontwikkelen, misschien word je nog eens bedrijfsleider'. Dat is niet mijn prioriteit. Ik wil op de werkvloer blijven.

**Interviewer:**	Het is voor jou gaandeweg duidelijk geworden wat je moet uitvoeren en wanneer je iets moet uitvoeren?

**Respondent:**	En ik word teruggefloten als ik het niet goed doe, denk ik dan. Het is een learning on the job eigenlijk zo.

**Interviewer:**	Heb je het idee dat je als teamleider nog bepaalt gedrag uit moet stralen?

**Respondent:**	Ja. Ik zit, aan de ene kant wordt mij opgelegd, ten minste geadviseerd, houd je meer op met teamleiders en je moet meer afstand nemen, aan de andere kant denk ik 'ik werk ook mee en krijg, naar mijn idee, ook meer dingen van mijn team gedaan als ik mij op redelijk hun niveau verplaats zeg maar gewoon'. Dus ik probeer daar een weg in te vinden, dat ik met iedereen om kan gaan.

**Interviewer:**	Is dat een moeilijk spel?

**Respondent:**	Voor mij is het niet zo zeer een moeilijk spel, omdat ik op deze afdeling als senior gekozen ben, met honderd procent van stemmen. Toen mijn bedrijfsleider in dienst kwam heeft >BEDRIJFSLEIDER< aan iedereen gevraagd. Er moest een senior komen en toen ben ik met honderd procent van de stemmen, nou honderd, mijn stem niet want ik kon niet op mezelf stemmen. Ik had me wel aanbevolen, maar niet op zelf gestemd. Iedereen stond er achter dat ik dat zou worden en toen het duidelijk was dat ik als teamleider op een andere afdeling wilde gaan werken is het team ook naar mijn bedrijfsleider toegestapt van 'dat willen wij niet, we willen dat >TEAMLEIDER< bij ons blijft', heb ik ook gewoon een heel groot draagvlak hier op de poli. Dus heb ik ook geen moeite mij autoritairder op te stellen. Zij weten gewoon dat ik bepaalde beslissingen moet nemen vanuit mijn rol als teamleider en dat ik ook wel eens wat minder populair kan zijn. Dat weten ze.

**Interviewer:**	Heb je het idee dat je een voorbeeldfunctie hebt als teamleider?

**Respondent:**	Ja. Dat had ik ook al toen ik senior was. Toen voelde ik dat ook al. Je moet het goede voorbeeld geven.

**Interviewer:**	Qua dat is er eigenlijk dus niet heel veel veranderd? Er zijn alleen wat taken bij gekomen die het verschil hebben gemaakt?

**Respondent:**	Ja. Dat ik daar meer gelegenheid voor krijg. Meer tijd. Van de 25 procent werk ik nu mee. Dat was 50 procent. In die zin he.

**Interviewer:**	Mijn laatste vraag. Heb je het idee dat een teamleider bepaalde competenties moet hebben, bepaalde dingen moet kunnen?

**Respondent:**	Ik denk wel dat niet iedereen teamleider kan zijn, als ik het even zo stel. Ik denk dat je in eerste instantie zelfverzekerd moet zijn. Dat je dat in ieder geval uitstraalt. Dat je weet waarover je het hebt. Je moet kunnen de-escaleren. Dat vind ik ook wel een puntje. Dat je niet overal vol in gaat. Dat maakt het alleen maar erger en in heel veel situaties is dat onwenselijk. Kennis van je team. Ja, dat kun je ook leren. Daar kun je ook in groeien zoals ik hier nu in groei. Empathisch moet je zijn. Je moet je ook in anderen kunnen verplaatsen, waarom ze dingen doen. Je kunt het ook wel vragen, dat je ook iets met het gevoel kunt doen vind ik ook heel belangrijk. Je moet goed dingen van twee kanten kunnen bekijken vind ik zelf. Niet een blik van 'dit kan niet', maar van 'waarom zou het kunnen, waarom zou het niet kunnen?'. En die twee daar moet je een balans in vinden. Dat geeft je een beslissing. Je moet ook intiem willen werken en niet autoritair, vanuit een troontje allemaal dingen opleggen. Ik vind samenwerken blijft het belangrijkste'.

**Interviewer:**	Heb je het idee dat dat samenwerken bevordert wordt door jouw achtergrond, het medische?

**Respondent:**	Ik heb natuurlijk in eerste instantie hetzelfde niveau als mijn collega's, allemaal doktersassistentes met allemaal specialisaties, dus ja in die zin. Ja, ik heb wel aanvullende opleidingen gedaan. Schriftelijke management in de gezondheidszorg, maar of dat nou de toegevoegde waarde is...

**Interviewer:**	Meer met het idee dat jij een historie hebt in de zorg. Dat helpt wel? Dus als jij mocht kiezen, als bedrijfsleider, tussen twee mensen waar één een achtergrond heeft in de zorg en de ander gewoon bedrijfskundig...

**Respondent:**	Daar ligt het verschil he?

**Interviewer:**	Dan zou degene met de zorgachtergrond een streepje voor hebben?

**Respondent:**	In mijn optiek wel. Ik denk juist dat je als bedrijfsleider, dan heb je het op dat organisatorische vlak en dan denk ik 'als je toch gebruik maakt van iemand die gewoon op de werkvloer aanwezig is, dan is het zeker meegenomen dat degene kennis heeft van die werkvloer, dat die ook weet wat er bij het personeel speelt, en wat er nodig is op die werkvloer'. Dat vind ik een toegevoegde waarde en anders moet je je dat allemaal eigen maken. Ik weet niet of je dat. Tuurlijk zullen er mensen zijn die dat zich wel heel goed kunnen verplaatsen in die collega's, maar voor mij is het makkelijker omdat ik precies hetzelfde werk kan doen als die collega's en dat werk heel lang heb gedaan. Ik weet waar ze tegen aan lopen en dat een verdomd vervelend apparaat is om mee te werken. Terwijl als iemand zegt 'ja, ja daar moet je toch mee om leren gaan. Een apparaat is een apparaat'. Juist omdat je daar zelf mee gewerkt hebt weet je waar het over gaat en snap je ook waar zij het over hebben. Dat vind ik een voordeel. En aan de andere kant, je staat ook dicht bij het team he? Dus het autoritaire dat je af en te moet, dat is bewust iets dat ik een beetje van mij af schuif omdat ik merk dat dit voor mij goed werkt. Ik weet niet of het voor een ander goed werkt, maar ik weet dat het voor mij goed werkt.

**Interviewer:**	Alleen op de momenten dat autoritair gevraagd wordt...

**Respondent:**	Moet je het wel kunnen zijn. Maar gelukkig is dat niet zo vaak. Maar ja dat komt ook omdat ik een beetje Pietje precies ben. Zo hebben we het afgesproken en waarom doe je het nu niet? Dat heb ik ook wel. Een beetje perfectionistisch, soms een beetje te, *respondent lacht*. Maar dan niet zo dat ik het in het autoritaire ga trekken. Daar hou ik niet zo van.

**Interviewer:**	Ik heb verder geen vragen meer. Ik weet niet of er nog dingen zijn die je zelf had willen toevoegen?

**Respondent:**	Nee, ik hoop dat ik aan al je vragen heb voldaan.

*Einde transcript*
